# EvoTol: a protein-sequence based evolutionary intolerance framework for disease-gene prioritization

**DOI:** 10.1093/nar/gku1322

**Published:** 2014-12-29

**Authors:** Owen J. L. Rackham, Hashem A. Shihab, Michael R. Johnson, Enrico Petretto

**Affiliations:** 1 Medical Research Council (MRC) Clinical Sciences Centre, Imperial College London, Hammersmith Hospital, Du Cane Road, London W12 0NN, UK; 2 The Medical Research Council Integrative Epidemiology Unit, University of Bristol, Oakfield House, Oakfield Grove, Bristol BS8 2BN, UK; 3 Division of Brain Sciences, Imperial College London, Hammersmith Hospital Campus, Burlington Danes Building, London W12 0NN, UK; 4 Duke-NUS Graduate Medical School, 8 College Road, Singapore 169857, Singapore

## Abstract

Methods to interpret personal genome sequences are increasingly required. Here, we report a novel framework (EvoTol) to identify disease-causing genes using patient sequence data from within protein coding-regions. EvoTol quantifies a gene's intolerance to mutation using evolutionary conservation of protein sequences and can incorporate tissue-specific gene expression data. We apply this framework to the analysis of whole-exome sequence data in epilepsy and congenital heart disease, and demonstrate EvoTol's ability to identify known disease-causing genes is unmatched by competing methods. Application of EvoTol to the human interactome revealed networks enriched for genes intolerant to protein sequence variation, informing novel polygenic contributions to human disease.

## INTRODUCTION

The application of whole-exome sequencing (WES) to patient cohorts is becoming increasingly widespread. One such use is through trio-sequencing where patients and their parents have their exome sequenced in order to identify disease-associated *de novo* mutations. Already this technology has been used to study a number of disease cohorts including epilepsy (1), schizophrenia (2), congenital heart disease (CHD) (3) and autism (4). While these WES studies identified several new disease genes on the basis of recurrent *de novo* mutation in affected offspring, the high rate of *de novo* mutation in the human genome makes it difficult to distinguish causal mutations from irrelevant random events, particularly for missense mutations, where genes are impacted only singly or where gene mutation falls short of exome-wide significance. Therefore, new tools for prioritization of disease-causing genes are required (5).

Currently, several techniques exist for predicting the pathogenicity of individual sequence variants, for example, PolyPhen (6) or SIFT (7), but these do not generalize their predictions to the gene level. Complementary to these variant-level prediction approaches, a number of techniques for identifying or prioritizing candidate disease-causing genes have been developed, but often these require prior knowledge of known disease-causing genes for a particular disorder (for example, ENDEAVOUR (8) or Prioritizer (9)). This class of methods uses different kind of similarity measures (e.g. functional similarity, sequence similarity, pathways membership, cross-species phenotype similarity, etc.) between known disease genes and the genes to be prioritized (reviewed in (10)). For these approaches, the prioritization accuracy, in the large part, depends on the accuracy, availability and specificity of the prior information used (known disease-causing genes).

Recently, a distinct (and new) class of approaches for gene-level prioritization of disease-causing genes has emerged, which does not require any prior disease-related information. Using the pattern of DNA sequence variation observed in the human population, one recently described method ranks a gene's likelihood of causing disease when mutated by calculating its ‘residual variance intolerance score’ (RVIS) (11). Specifically, using the Exome Variation Server as a source of genetic variation across the population, the RVIS estimates the studentized residuals of rare versus all variations known to occur in a particular gene and this measure is used as a proxy for that gene's ability to tolerate mutations. It has been shown that genes with high intolerance to mutations according to RVIS are more likely to be disease-causing genes than genes that can tolerate mutations (11). Thus, the RVIS uses the pattern of sequence variation observed in the human population to prioritize possible disease-causing genes from sets of genes impacted by *de novo* mutation in disease cohorts (e.g. from WES of family trios). Despite the RVIS approach being relatively new it is already a widely applied technique in the field, being used broadly including studies into the genetics of epilepsy (12), kidney disease (13), autism (14) and familial dyskinesia (15). A second, complementary approach for the analysis of excesses in *de novo* mutation per gene by calibrating a model of *de novo* mutation has been recently introduced by Samocha *et al*. (16). This method uses the absence of rare functional variation in comparison to the expectation within humans to derive constraint scores (or missense *Z* scores), which can be similarly used to evaluate excesses of mutation in gene sets and evaluate the significance for individual genes.

Here we present an alternative method to RVIS (11) and the constraint score (16) for gene-level prioritization of disease genes. Uniquely, our method, EvoTol, combines genic intolerance with evolutionary conservation of whole protein sequences or their constituent protein domains to prioritize disease-causing genes. EvoTol's ability to prioritize disease-causing genes is as a result of utilizing the processes guiding evolution, which have the effect of penalizing mutations that unduly influence the ‘fitness’ of an organism. By studying protein sequences across multiple species it is possible to estimate which protein sequences are more probable given what has been observed. These probabilities are captured by creating sequence profiles, such as those found in PFAM (17) or SUPERFAMILY (18). These profiles provide a rich resource for identifying protein-coding genes that are more intolerant to mutation than others and can be used for predicting a gene's propensity to cause disease. EvoTol builds on and extends the RVIS approach by not looking exclusively at DNA sequence variation in the human population, but instead leveraging the information on protein sequence evolution (via FATHMM (19)) to identify genes where the number of mutations that are likely to be damaging based on evolutionary protein information is higher than expected. We use this strategy to define an alternative proxy measure for intolerance to mutation (i.e. evolutionary intolerance), which can be used to prioritize disease-causing genes.

We show that EvoTol performs better than RVIS at prioritizing disease-causing genes from the Online Mendelian Inheritance in Man (OMIM) database (20) and also at prioritizing possible disease causing genes from previously published WES trio data sets for epilepsy (1) and CHD (3). In order to show that the EvoTol measure can also be integrated with other data types to improve its performance we provide two example applications. In the first case, we integrate the measure with tissue-specific gene expression from >700 CAGE libraries taken from various cell types and tissues (from the FANTOM5 consortium (21)). These are classified into cell-type categories using Uberon cell-type ontology (22). We then illustrate how removing genes that are not expressed in a given tissue can result in a 3- to 7-fold increase in the detection of known disease-causing genes in disorders that are limited to a single cell or tissue type. In the second case, we integrate the measure with the human interactome (STRING data (23)). In doing so we identify specific clusters with increased (and decreased) evolutionary intolerance scores that reflect functionally coherent biological processes relevant to disease aetiology, and which may be of use to identify polygenic contributions to complex disease.

EvoTol is freely available and accessible online at www.evotol.co.uk, where users can input gene lists to be ranked according to the evolutionary intolerance score.

## MATERIALS AND METHODS

### Building the evolutionary intolerance (EvoTol) scores

**Figure 1.**
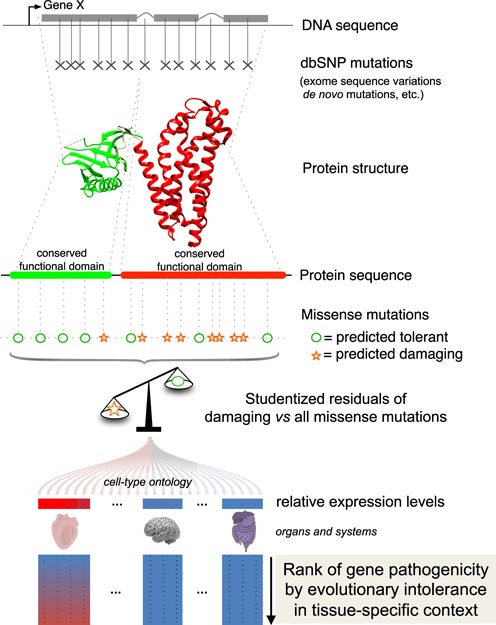
EvoTol uses FATHMM predictions (on the protein space) for dbSNP missense mutations in each gene to derive a gene-level ‘evolutionary intolerance’ score, which corresponds to the studentized residual of damaging versus all mutations. For a given gene, the percentile of the EvoTol score provides a measure of intolerance with low studentized residuals corresponding to high intolerance. Integrating gene expression data from the FANTOM5 consortium, the gene pathogenicity predicted by the evolutionary intolerance score is determined with respect to the specific tissue-context where the gene is expressed.

A graphical summary of the EvoTol approach which builds upon the intuition of RVIS is reported in Figure 1, summarized in the following steps and explained in detail below:

- Taking a set of mutations from dbSNP, each mutation is assessed by FATHMM in order to predict if it is damaging or not.
- The mutation set is then grouped by gene and a linear regression of the total number of mutations against the number of damaging mutations for all genes is calculated.
- The studentized residuals are calculated for each gene and this is used as a proxy for intolerance.
- The genes are then ranked by their studentized residuals and assigned a percentile based on this rank. Those genes that appear in the top 25 percentile are considered to be intolerant, with most intolerant genes being in the top one percentile.

EvoTol utilizes data from FATHMM (19) to predict the functional consequences of missense mutations. To this end we apply FATHMM to a set of 1 068 744 mutations from dbSNP (24), obtaining a FATHMM score for each. Following this, for each gene we derive a new score (the EvoTol score) by taking the studentized residuals of all mutations in a gene and plotting these against the number of predicted damaging mutations according to FATHMM. A negative residual represents a gene evolutionarily intolerance to mutation (i.e. pathogenic) while a positive residual represents a gene tolerant to mutation (i.e. benign).

Specifically, FATHMM exploits the evolutionary information stored in hidden Markov models of protein sequences or their constituent domains and combines this information with a novel weighting scheme in order to predict if a missense mutation will be damaging or not. FATHMM has previously been shown to outperform state-of-the-art prediction methods (i.e. SIFT, PolyPhen, PANTHER, SNPs&GO and MutPred) (19). The hypothesis underlying the EvoTol score is based upon the assumption that if the same amino acid is conserved across species it is likely to have a critical function, and therefore genes that contain a higher proportion of these mutations are those that are the most intolerant to mutation. In order to use FATHMM information to identify intolerance at the gene level (and compare this with RVIS), we used 1 068 744 single nucleotide polymorphisms (SNPs) that appear in dbSNP (24), of which 686 501 are missense and were scored using FATHMM. FATHMM predictions are classified as either ‘TOLERATED’ (535 592 in total) or ‘DAMAGING’ (120 089 in total) and were grouped by gene. Following this we linearly regressed the number of DAMAGING mutations against the total number of mutations for each gene and calculated the studentized residuals. This transformation controls for SNP density and gene length and as such creates the EvoTol score. The studentized scores were converted to percentiles such that the most intolerant gene (i.e. the gene where the number of DAMAGING mutations is highest for its mutational load) falls in the first percentile and vice versa.

The detail of how this method can be benchmarked against existing methods and modified in order to incorporate cell-specific gene expression and protein interaction data are described in the following sections.

### Benchmarking EvoTol and RVIS

In order to assess the performances of both RVIS and EvoTol when prioritizing putative disease genes we used the same sets of OMIM genes employed by Petrovski*et al*. when assessing RVIS (11). In total there are six gene sets; a set containing all of the OMIM genes (with genes linked to search terms resistance, cancer, somatic, susceptibility, carcinoma or tumor removed), and five subsets of genes extracted by identifying genes that matched a search for ‘recessive’, ‘haploinsufficiency’, ‘dominant negative’, ‘*de novo*’ and a combination of ‘*de novo*’ and ‘haploinsufficiency’. The RVIS (0.1% threshold) and EvoTol percentile for each of the genes in a given set is then assigned and a count of the number of disease-causing genes that would be found at each percentile are reported. In order to show the baseline prediction, the result of randomly assigning a percentile to each gene is also shown.

**Figure 2.**
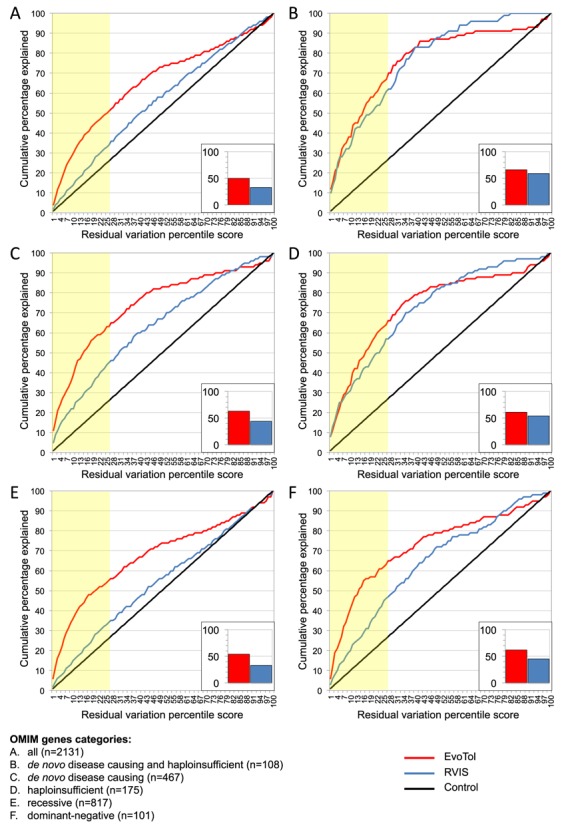
Comparison between EvoTol and RVIS intolerance scores using the OMIM database, showing the cumulative percentage plots for the residual variation intolerance scores for the six OMIM gene lists (A–F, as indicated). Inserts, cumulative percentage of OMIM genes identified within the 25th percentile of intolerance (yellow box) by EvoTol and RVIS.

### Real data analysis: epilepsy and CHD data sets

The Epi4K project (25) analyzed whole-exomes of 264 probands, and their parents, and confirmed 329 *de novo* mutations (26) which were found in 176 genes, each of which we annotated with its EvoTol score as described above. A large study of CHD by Zaidi *et al*. (3) included 362 parent–offspring trios comprising a proband with severe CHD and no first-degree relatives with identified structural heart disease. WES analysis found 184 genes containing *de novo* missense mutations. All missense mutations for epilepsy and CHD used in these studies are available at www.evotol.co.uk, where the EvoTol scores can be retrieved for each gene. For each intolerant gene we consider its protein product and derived known phenotype associations from UniProt db (Universal Protein Resource, www.uniprot.org), as reported in Supplementary Table S3 (epilepsy) and Supplementary Table S5 (CHD), respectively.

### EvoTol analysis of the STRING network

The network analysis was performed using STRING v.9 (27). The network was filtered to include only high-confidence edges with a STRING score greater than 500 and with an experimental score greater than zero. This has the effect of removing most of the low quality (mostly literature-based) edges and ensures that each edge inference is supported by experimental data. The network was clustered using the ‘Molecular Complex Detection’ (MCODE) algorithm (28,29) with the default settings, which have been used from within Cytoscape (30). MCODE identifies discrete subnetworks (or clusters) from within a larger network (e.g. STRING) and has the advantage over other clustering methods to fine-tune directly clusters of interest without relying on the rest of the network (28).

Each of the nodes of the network is a gene and as such can be assigned its EvoTol scores as calculated above. The clusters can be compared by performing a Mann–Whitney U test on the distribution of EvoTol scores from each cluster. A cluster that is significantly more/less intolerant will result in a statistically significant *P*-value for the difference in median EvoTol score compared to that of the background (i.e. the other clusters).

### Functional annotation of intolerant genes and subnetworks

We used DAVID (the database for annotation, visualization and integrated discovery) (31) to investigate functional enrichment for intolerant genes and STRING subnetworks. The DAVID tool uses several sources of gene annotation, including Gene Ontology (GO) and Kyoto Encyclopedia of Genes and Genomes (KEGG) pathways to assess overrepresentation of specific functions and pathways within a given gene set and accounts for the size of the gene set. The background in the case of epilepsy and CHD was the set of *de dovo* mutation containing genes and for the networks it was the whole genome. To annotate human genes with respect to previously known disease phenotype functions we used Ensembl Biomart to retrieve data from the OMIM database for the Mendelian disorders (20), and the Developmental Disorders Gene to Phenotype (DDG2P (32)) and Orphanet (http://www.orpha.net) databases.

### Cell-type-specific evolutionary intolerance by integrating the FANTOM consortium data

The FANTOM5 consortium produced capped analysis of gene expression (CAGE) data from a large number of primary cells, tissues or cell lines (21) that can be used to provide context-specific gene expression information in a manner that is useful for evolutionary analysis, as we previously proposed (33). Each of the FANTOM samples can be annotated as belonging to UBERON cell-type ontology (22), resulting in sets of samples which are ‘anatomically’ related at various levels. For instance, the UBERON term ‘Central Nervous System’ will group CAGE libraries from the FANTOM5 project that correspond to this term, e.g. neuron, hippocampus, adult brain, etc. For each such grouping provided by the UBERON ontology we use the corresponding FANTOM5 data to calculate the mean expression level of each gene and define a given gene to be reliably expressed if the average gene expression is greater than 100 tags per million (TPM) across the group, see Supplementary Figure S2. These sets of expressed genes provide biological (cell-type specific) context and can be used to redefine the percentiles of evolutionary intolerance scores. Specifically, the evolutionary intolerance scores are calculated as described above, providing a studentized residual for each gene based on a linear regression of the number of known mutations for that gene against the number that are predicted as damaging. To produce an UBERON term-specific ranking the genes that are not reliably expressed are removed, and the remaining genes ranked by their residuals. These rankings are then converted to percentiles and it is these percentiles that are used to prioritize those genes that appear the most intolerant in that specific context.

To show that cell-type-specific evolutionary intolerance increases the power to predict disease-causing genes, we retrieve known gene-phenotype associations (using UniProt, see above) and compare these with the predicted most intolerant (<25 percentile score) genes using the cell-type-specific evolutionary intolerance. The fraction of genes predicted by EvoTol using cell-type-specific evolutionary intolerance is then compared with the fraction of genes predicted using non-cell-type-specific evolutionary intolerance and a fold change of enrichment is calculated. For display purposes, we report these fold enrichments by grouping ontological cell-types into six high-level categories (respiratory system (UBERON:0001004), digestive system (UBERON:0001007), circulatory system (UBERON:0001009), central nervous system (UBERON:0001017), musculoskeletal system (UBERON:0002204) and immune system (UBERON:0002405)). The complete UBERON cell-type ontologies with the corresponding samples identifiers retrieved from the FANTOM5 data set are reported in Supplementary Table S7 online.

### Identifying intolerant protein domains using EvoTol

Despite the prevailing direction in the field to concentrate on identifying intolerant genes, EvoTol methodology also permits the identification of intolerant protein domains. This allows for an intermediate resolution between individual SNPs and whole genes, which may be helpful when only part of a gene is truly intolerant to mutation. In order to identify intolerant protein domains we apply the same methodology as described above with the exception of grouping mutations by protein domain rather than gene. In this way the total number of mutations found within a domain is linearly regressed against the number of damaging mutations. A studentized residual is calculated for each domain, which in turn is converted to a percentile. There is more than one domain type included with FATHMM and as such the intolerant domains list contains domains from SUPERFAMILY, PfamA and PFamB. The intolerant domains list is made available online through www.evotol.co.uk.

## RESULTS

First, we set out to benchmark EvoTol performance against RVIS (11) and the gene constraint score (16) with respect of known disease-causing genes. Both of these methods for prioritizing disease genes do so without relying on *a priori* information on the disease of interest, and as such are the only available techniques for comparison. We then apply the EvoTol framework to the analysis of WES data in epilepsy and CHD, and systematically test the utility of EvoTol to prioritize genome-wide candidate disease-causing genes. To illustrate an additional application of EvoTol, we also carried out a network-level evolutionary intolerance analysis of the human interactome. Finally, to demonstrate the increased predictive ability of EvoTol when candidate genes are stratified by their expression in a given tissue relevant to the disease, we carried out separate comparative analysis of EvoTol performance with and without using information on tissue specificity.

### Evolutionary intolerance has better predictive power than genic intolerance to prioritize disease-causing genes

The OMIM database comprises a large repository of known genes and mutations for Mendelian disease (20). The OMIM database can be used to extract functionally coherent gene-sets (for instance, genes containing the keywords ‘*de novo*’ or ‘haplo-insufficuency’) and these sets were previously used to assess the performance of RVIS (11). Here, we use the same OMIM gene-sets to test the ability of EvoTol to discriminate genes that do and do not cause disease in comparison with RVIS. Figure 2 shows the relative performance of RVIS and EvoTol to predict all OMIM genes and specific gene-sets associated with the keywords ‘haploinsufficiency’, ‘*de novo* disease causing’, ‘recessive genes’ and ‘dominant negative’. For each considered gene-set EvoTol provides greater enrichment for disease genes compared with RVIS (see Supplementary Figure S1 for receiver operating characteristic (ROC) curves). It is worth noting that OMIM is far from a complete resource and many disease genes remain unannotated or undiscovered. In this respect the following benchmark provides a good way of comparing techniques but is not a good measure of overall accuracy of each technique since many of the ‘false positive results’ may well involve disease-causing genes that remain unknown. Looking at the full set of OMIM genes, we found that the first 25th percentile of the EvoTol score (previously adopted to prioritize disease genes using RVIS (11)) contained 50% of the OMIM genes while RVIS contained only 35%, Figure 2A (insert). The increased ability of EvoTol to retrieve disease-causing genes from OMIM as compared with RVIS was preserved for all OMIM gene lists investigated and was most apparent for the ‘recessive’ gene-set (54% versus 33% for EvoTol and RVIS at the 25th percentile cutoff, respectively, Figure 2). In addition, we found the recently introduced gene constrain score (16) to have similar performance as RVIS in predicting genes belonging to OMIM terms (Supplementary Figure S5 and Table S6). The performance of EvoTol is still better than existing techniques if the data source is the Exome Variation Server rather than dbSNP, see Supplementary Figure S5.

We looked in detail at the top 10 intolerant genes genome-wide that are predicted by EvoTol and compared the percentile positions with those predicted by RVIS (Supplementary Table S1). We identified several genes (*BRCA1*,*ABCA4*,*LRP2*,*FBN3* and *HBB*) judged to be highly evolutionary intolerant by EvoTol that RVIS placed in the set of genes tolerant to mutation. While *BRCA1* is one of the most intolerant genes predicted by EvoTol (i.e. within the first percentile of residual intolerance) it is only within the 79th percentile according to RVIS. To explain these differences, we investigated *BRCA1* in detail, for which there are 720 single nucleotide mutations found in dbSNP (24) for which a FATHMM score can be obtained. Of these 720 single nucleotide variations (SNVs), 426 (59%) are considered to be damaging according to FATHMM, a ratio of tolerant to intolerant mutations that allowed EvoTol to place *BRCA1* in the list of most intolerant genes. In the same gene there is a total of 237 SNVs documented in the Exome Variant Server, of which 34 (14%) are common variants. Accordingly, RVIS places *BRCA1* in the set of tolerant genes despite over half the SNVs being predicted as damaging using amino acid conservation by FATHMM. For *BRCA1*, PolyPhen (6) predicted 63% of mutations are either ‘possibly’ or ‘probably’ damaging and two phenotypes are associated with mutations in *BRCA1* in the OMIM database (20) (Supplementary Table S1). Using evolutionary intolerance EvoTol predicted *BRCA1* to be highly intolerant to functional variation and therefore highly prioritized as a disease-causing gene. Similar differences in the intolerance predicted by EvoTol and RVIS were observed for *ABCA4*, where only 149 (out of 538) SNVs are considered tolerant to mutation according to FATHMM. The high intolerance score for *ABCA4* by EvoTol is further supported by PolyPhen prediction (56% of either ‘possibly’ or ‘probably’ damaging mutations). There are six phenotypes associated with mutations in *ABCA4* in the OMIM database (20) (Supplementary Table S1), one of which is cone-rod dystrophy; 65% of patients with cone-rod dystrophy carry a mutation in this gene (34) which lends weight to the hypothesis that *ABCA4* is indeed intolerant to mutation.

### Using evolutionary intolerance to prioritize disease genes in epilepsy

A recent study searched for disease-causing mutations in a cohort of patients with severe epilepsy using WES from 264 trios (Epi4k consortium (1)), resulting in a set of 329 *de novo* mutations occurring in 176 different genes in the disease cohort. We re-analyzed this set of mutated genes by first ranking them by their evolutionary intolerance score (and by RVIS for comparative purposes), see Supplementary Table S2. We focused first on genes within the 25th percentile of EvoTol scores and annotated this gene set using the DAVID tool (31) to investigate if this quartile of most intolerant genes was enriched for functional categories relevant to epilepsy. For the EvoTol results this showed significant overrepresentation for ion channel activity genes (*BEST2*,*CACNA1A*,*GABRA1*,*GABRB1*,*GABRB3*,*GRIN1*,*KCNQ2*,*KCNQ3*,*KCNB1*,*SCN8A*,*SCN1A*,*SCN2A*), enrichment *P* = 0.03 with respect to the background of the 176 *de novo* mutations containing genes. The EvoTol predictions for the genes within the 25th percentile of intolerance were supported by data on associations between diseases and phenotypes and human genes from OMIM, DDG2P (32) and Orphanet (http://www.orpha.net) databases, Supplementary Table S2.

**Figure 3.**
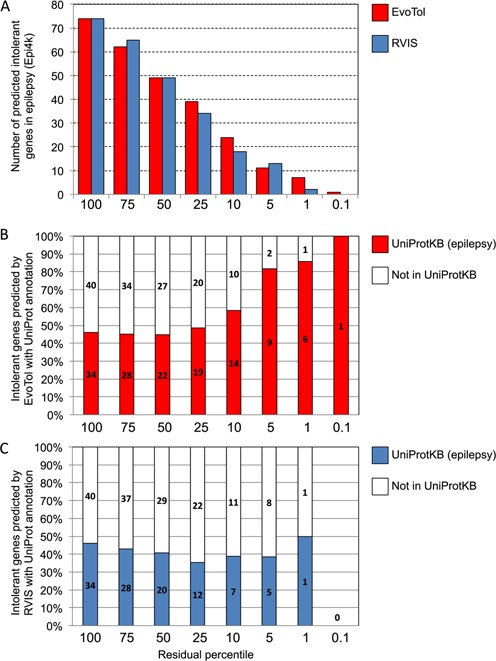
EvoTol and RVIS intolerance scores using WES data from the Epi4K consortium (26) and comparison with functional annotation of disease genes in UniProt. (A) Number of predicted disease genes for different residual percentile scores. For each residual percentile score of EvoTol (B) and RVIS (C) we report the number of predicted disease genes and percentages (*y*-axes) that were annotated as ‘epilepsy’ genes by UniProt (keyword: ‘Epilepsy [KW-0887]’, Supplementary Table S3).

We then compared the ranked gene lists predicted by EvoTol and RVIS with separate functional annotation data from UniProt (http://www.uniprot.org) and found that EvoTol has increased power to detect genes which if defective cause epilepsy. To this aim we retrieved a list of 81 proteins (and corresponding genes) associated with epilepsy by UniProt (keyword: ‘Epilepsy [KW-0887]’, Supplementary Table S3), and investigated the extent to which these were predicted by EvoTol or RVIS at different residual percentile scores. Overall, EvoTol predicted more intolerant genes (i.e. ranked within the 25th percentile) than RVIS (Figure 3A) and among these, a higher fraction of genes were also functionally annotated as epilepsy-causing by UniProt (Figure 3B and C). Notably, for decreasing residual percentile scores, EvoTol identified an increased proportion of genes in agreement with the functional annotation by UniProt, whereas RVIS identified at most 50% of genes in agreement with UniProt (Figure 3B and C). These analyses also show the high sensitivity of the EvoTol score, which predicted more disease-causing genes (according to UniProt functional annotation) for lower residual scores compared to RVIS.

### Annotation of the most evolutionary intolerant genes prioritized by EvoTol in epilepsy

**Figure 4.**
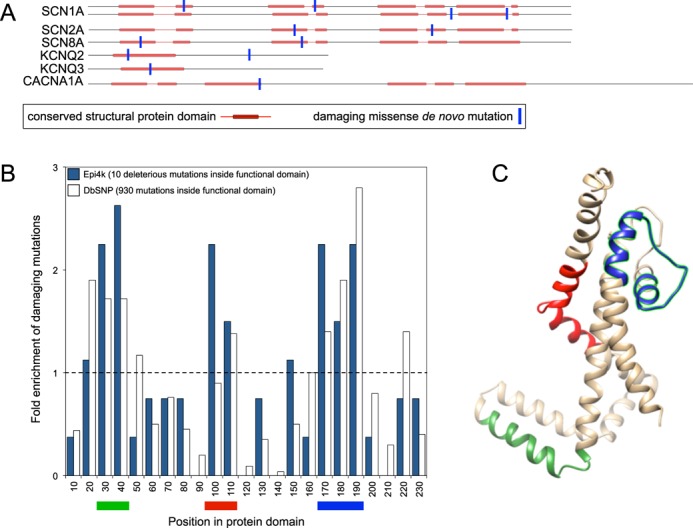
(A) Location and frequency of damaging missense mutations in seven of intolerant genes predicted by EvoTol in epilepsy. (B) Distribution of fold-change enrichments of damaging mutations in the conserved voltage-gated potassium channels protein domain, where each bin in the histogram groups 10 residues of the protein domain. The expected level of enrichment of damaging mutations is indicated by the dotted line. (C) Protein domain model from voltage-gated potassium channels superfamily (SUPERFAMILY ID: 81324, Model ID: 0041998) where the superfamily model is based on the seed structure d1orqc_ (http://scop.berkeley.edu/sunid=87348). The locations of the domain that show the greatest enrichment in deleterious mutations are indicated in (B) and (C) and colored as follows: green (30–50 AA), red (90–110 AA) and blue (170–190 AA), the latter represents the protein location where the ion binds to the channel.

In contrast with the RVIS method, the EvoTol approach identified *SCN1A*, an established epilepsy gene (35), as the most important gene in the Epi4K data set (0.04 percentile rank of evolutionary intolerance, Supplementary Table S2). In the Epi4K data four *de novo* mutations are found in *SCN1A* across all trios. The gene itself encodes for a protein that has four repeated protein domains that form a tetrameric transmembrane channel, as do other highly intolerant genes identified by EvoTol including *KCNQ2* (0.51th percentile rank) and *KCNQ3* (6th percentile rank). Notably, neither of these genes scored ranked as highly on the RVIS measure (Supplementary Table S2), although these genes are known epilepsy genes (36). Among the Epi4K genes that are highly intolerant to missense mutation predicted by EvoTol are *CACNA1A, KCNQ2, KCNQ3, SCN1A, SCN8A* and *SCN2A* which all encode voltage-gated ion channel subunit proteins and all have a protein domain in common. Within these 6 highly intolerant genes there is a total of 13 mutations predicted as damaging and, of these, 10 are found within the same protein domain, whereas the 3 remaining mutations are located between 2 and 30 amino acids from the predicted location of the protein domain (Figure 4A). Given that the average length of the proteins (∼2000 AA) and of the protein domains (∼200 AA) then assuming a uniform mutation rate across the gene, the probability of this clustering of mutations occurring by chance is very low (*P* = 2.9 × 10^−10^). This repeated occurrence of damaging mutations in a specific protein domain suggests this is functionally important for the phenotype. In order to investigate whether the same clustering of mutations is similarly observed in other diseases, we investigated the occurrence of all SNPs from dbSNP occurring in this domain. Figure 4B shows the distribution of fold changes expected for all damaging mutations (according to FATHMM) from dbSNP that map to this domain as well as the occurrence in the epilepsy set alone. Three regions showed more than expected damaging mutations (shown in green, red and blue in Figure 4B). Of particular interest are residues 180–190 (blue region) showing a ∼2-fold enrichment to the expected number of damaging mutations occurring in epilepsy and overall, coinciding to the location in the protein where ions binds to the channel (Figure 4C). Structural effects of mutations to the *SCN1A* gene are well documented (37,38); here, we provide these observations as an example of the follow-on investigation of the EvoTol results alone, and without extensive structural investigation of the protein domains.

### Using evolutionary intolerance to prioritize disease genes in CHD

**Figure 5.**
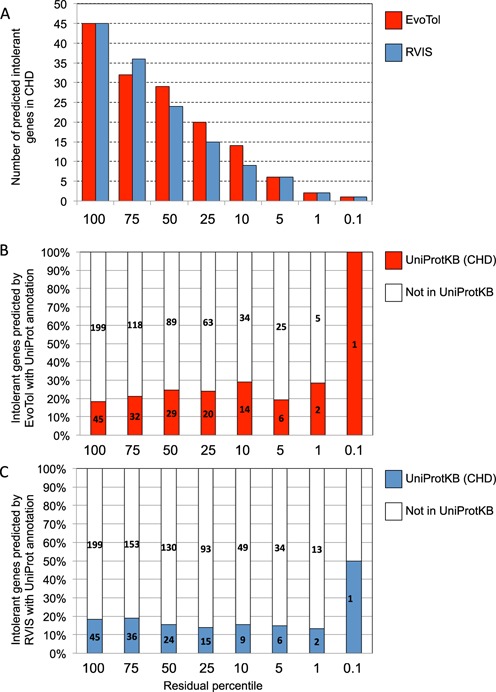
EvoTol and RVIS intolerance scores using WES data in CHD (3) and comparison with functional annotation of disease genes in UniProt. (A) Number of predicted disease genes for different residual percentile scores. For each residual percentile score of EvoTol (B) and RVIS (C) we report the number of predicted disease genes and percentages (*y*-axes) that were annotated as ‘CHD’ genes by UniProt (keyword: ‘congenital heart disease’, Supplementary Table S5).

Zaidi *et al*. performed trio exome sequencing to identify *de novo* mutations that are associated with CHD (3), suggesting these mutations tended to be involved in histone modification. The study found 184 genes containing *de novo* mutations. An EvoTol re-analysis of these data identified 53 genes in the top 25th percentile of EvoTol scores (Supplementary Table S4). Focusing on the most intolerant genes, i.e. those within the second percentile of EvoTol scores, we identify 10 genes (*PTCH1*,*LRP2*,*FBN2*,*KCNH6*,*ABCA13*,*ALPL*,*STAB1*,*GRM8*,*GANAB* and *FGFR4*). The most intolerant gene in CHD predicted by EvoTol (and RVIS) is *PTCH1*, which is involved in sonic hedgehog signaling and mutations in the gene are known to cause congenital disorders (39). Several genes predicted to be intolerant by EvoTol and RVIS have been previously implicated in heart disease, including *FBN2*, linked to congenital contractural arachnodactyly, a disease characterized by contractions in connective tissue (40), and *FGFR4* that encodes the protein Fibroblast growth factor receptor 4, a closely related gene that in mouse contains a mutation causing CHD (41). However, in several cases EvoTol and RVIS provided conflicting rankings of gene intolerance in CHD. To investigate these differences in more detail, we retrieved all genes associated to ‘CHD’ in the UniProt database (Supplementary Table S5). While the proportion of intolerant genes predicted by EvoTol that match the UniProt gene annotation is no greater than 30% (for ranks <1%), Figure 5 shows that on the whole EvoTol has similar or greater power and sensitivity than RVIS in detecting CHD genes. We therefore investigated specific cases where EvoTol classified a gene to be highly intolerant (i.e. <10th percentile rank) and RVIS predicted the same gene to be tolerant. One such case is the low-density lipoprotein-related protein 2 gene (*LRP2*) that is a highly intolerant gene according to EvoTol (0.21th percentile rank), but in contrast was predicted highly tolerant by RVIS (99th percentile rank). However, carriers of *LRP2* mutations suffer from Donnai–Barrow syndrome, a rare, autosomal recessive disorder often characterized by congenital heart anomalies (42) and LRP2-deficient mice are born with severe congenital anomalies affecting multiple tissues (43) due to misregulation of cholesterol levels in the developing embryo. Another intolerant gene according to EvoTol but not RVIS is *KCNJ15* whose expression in the heart is developmentally controlled (44), in keeping with the observation of damaging mutations in this gene in CHD patients. Potassium voltage-gated channels have been extensively investigated as possible therapeutic targets for CHD (45) and other closely related genes, *KCNA5* (46) and *KCNQ1* (47), have been previously linked to heart disease. Another class of highly intolerant genes predicted by EvoTol but not RVIS are those which belong to the ATP-binding cassette (ABC) family of transmembrane transporters, such as ABCA10 a cholesterol responsive gene (48), ABCA13 or ABCB6 previously involved in cardiovascular disease, such as atherosclerosis (49). These highly homologous genes from the same family have been investigated in great depth, with ABCA1 being tightly linked to cholesterol levels and heart disease (50), therefore, suggesting a possible role for the mutated ABC transporters in CHD. While the most intolerant genes solely predicted by EvoTol belong to different protein classes (e.g. potassium voltage-gated channels and ABC transporters), functional enrichment analysis of the genes in the top 25th percentile of EvoTol scores using DAVID shows enrichment for a number of terms including keywords, such as ‘receptor’ (*P* = 0.0013) and ‘negative regulation of nitrogen compound metabolic process’ (*P* = 0.0061) with respect to the other 184 mutation containing genes. The genes within the top 25th percentile by RVIS score were only enriched for a two terms ‘phosphoprotein’ (*P* = 0.0017) and MAPK signaling pathway (*P* = 0.0052). These analyses demonstrate the ability of EvoTol to prioritize both new disease genes and pathways from exome-sequence data over and above those identified by RVIS.

### The identification of highly intolerant protein domains

Since EvoTol incorporates evolutionary information through the inclusion of data on the conservation of protein domains it also allows to comment on which of these domains are in themselves highly intolerant. Since it is not always the case that the entire gene is intolerant but only a small region being able to identify intolerant subregions of a gene (like protein domains) is extremely useful, and not possible in the existing methodologies. The question of whether the whole gene is the correct unit to be judging intolerance is an important one within the field, with some genes clearly being more intolerant in confined regions of the protein. Having calculated both a domain and gene level intolerance we are able to identify genes where this is indeed the case. As an example, if we consider the SUPERFAMILY ‘Voltage-gated potassium channel’ protein domain we find that this is a highly intolerant domain with 930 damaging mutations identified within it. Correspondingly, 85 out of 96 genes that contain this domain are also considered as intolerant, while 11 are not. An example of this is GALNT8, which in its various isoforms (ENSP00000408321 and ENSP00000252318) contains four different SUPERFAMILY protein domains (POZ Domain, Ricin B-like lectins, Nuceotide-diphospho-sugar transferases and Voltage-gated potassium channels). Of these only the Voltage-gated potassium channel is considered intolerant. As this domain only occupies 234 amino acids out of the 1166 reported it is clear why the protein as a whole can be considered tolerant. However, a mutation in the Voltage-gated potassium channel is still highly likely to be disease causing. This demonstrates that despite in the majority of cases the gene-level is a good unit to study intolerance, there are also cases where considering the domain-level analysis can boost detection of disease-causing genes (see Supplementary Figure S6 for further details). In order to allow the community to study both gene and domain level evolutionary intolerance, both measures are accessible at www.evotol.co.uk.

### Integrating evolutionary intolerance with tissue-specific gene expression boosts prediction of disease-causing genes

**Figure 6.**
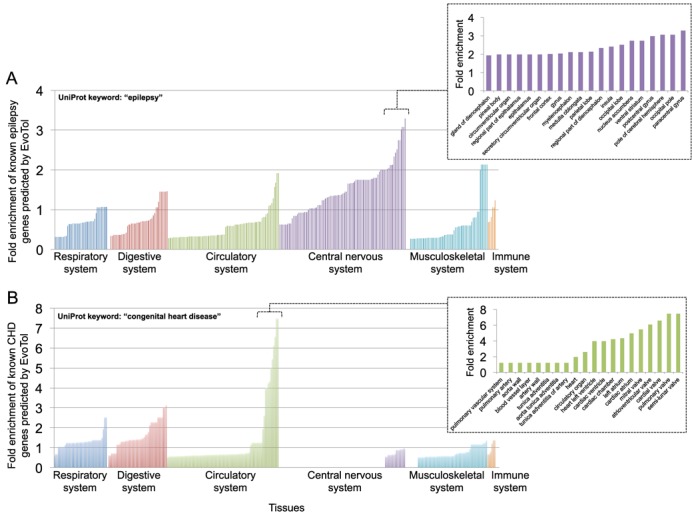
Taking into the context in which genes are expressed increases the enrichment of disease-causing genes within those predicted to be evolutionary intolerant by EvoTol. For each cell type we identified robustly expressed genes as those whose average expression is >100 TPM, therefore defining lists of genes specifically expressed in a given tissue. The ranking of a gene by its evolutionary intolerance score is then calculated with respect to all other genes expressed in the same tissue. Highly evolutionary intolerant genes (in the top 25th percentile score) are then compared with known disease-causing genes as annotated by UniProt keywords: ‘epilepsy’ (A) and ‘CHD’ (B). For each tissue (*x*-axes) we report the fold enrichment of predicting known disease genes by EvoTol when information on tissue-specific gene expression is used (*y*-axes). Fold enrichments are calculated as the ratio between the number of genes predicted by EvoTol using tissue-specific evolutionary intolerance and the number of genes predicted using non-tissue-specific evolutionary intolerance. Inserts, fold enrichments observed for the top 20 tissues from the central nervous system (top) and the circulatory system (bottom).

EvoTol can also be integrated with information on cell-type and tissue-specific gene expression patterns to rank genes with respect to their pathogenicity in tissue-specific context. This layer of information can prove useful to prioritize disease genes when the functional impact of mutations is restricted to a specific tissue or cell-type, as for example, in disorders of the human brain or heart. To illustrate this we retrieved the lists of predicted most intolerant genes (<25th percentile) after removing those genes not expressed in a tissue type of interest (e.g. the central nervous system or circulatory system) and the list of predicted most intolerant genes (<25th percentile) (i) without using and (ii) including, cell-type-specific expression information. We then compared these lists with respect of the UniProt proteins (and corresponding genes) associated with epilepsy by UniProt (keyword: ‘epilepsy’, Supplementary Table S3) and CHD (keyword: ‘congenital heart disease’, Supplementary Table S5) and calculate the number of matching disease-causing genes. We show that by ranking genes by ‘evolutionary intolerance’ with respect to their tissue-specific expression, we increase the power to predict known disease genes from UniProt in the specific tissue-context relevant to the disease, Figure 6. This was achieved by using CAGE data from the FANTOM5 consortium (21) and selecting an average gene expression greater than 100 TPM to identify robustly expressed genes for each category (see Materials and Methods), we found ∼3-fold increase in detection of ‘epilepsy’ UniProt genes in the central nervous system and 6- to 7-fold increase in detection of ‘CHD’ UniProt genes in the circulatory system. We also observe that the fold-increase in detection of UniProt genes associated with ‘epilepsy’ and ‘CHD’ rose when a more stringent thresholding of expression was adopted, reaching a maximum fold-increase for gene expression thresholds between 100 and 200 TPMs (see Supplementary Figure S2).

In some cases the highly intolerant genes predicted by EvoTol were also highly expressed in multiple tissues or cell types. For instance, we found that the *ATP2A2* gene, encoding one of the sarco/endoplasmic reticulum Ca(^2+^)-ATPases (SERCA) (which are intracellular pumps located in the sarcoplasmic or endoplasmic reticula of muscle cells), ranked very highly in the evolutionary intolerance score in adult brain tissue (EvoTol percentile score = 5.7) and was associated with epilepsy by UniProt (Supplementary Table S3). Mutations in *ATP2A2* are known to cause neuropsychiatric phenotypes (including epilepsy) in patients with Darier disease (51). In addition, *ATP2A2* is predicted to be highly intolerant to mutations in a number of other contexts including the cardiovascular system (EvoTol percentile score = 8.2 in adult heart tissue), suggesting an additional pathogenic role for this gene in cardiac disease, as previously reported (52,53). Since *ATP2A2* acts as a Ca^2+^ pump, an essential function in both the brain and heart, its pleiotropic damaging effect in these diseases is not unexpected. Overall, these analyses show that stratifying gene candidates by their expression in a tissue relevant to the disease under consideration boosts EvoTol's ability to predict gene pathogenicity and can be used to reveal pleiotropic gene effects on different diseases. In this proof of principle we integrated EvoTol with CAGE data from the FANTOM5 consortium (21). However, with the increasing accumulation of gene expression data in public repositories (e.g. Gene Expression Omnibus (GEO)) and the systematic annotation of disease processes to specific tissues (54), more comprehensive analyses of the link between tissue- or cell-specific gene expression and a gene's EvoTol score may yield better predictions of a gene's pathogenicity.

### Evolutionary intolerance analysis of the human interactome network

**Figure 7.**
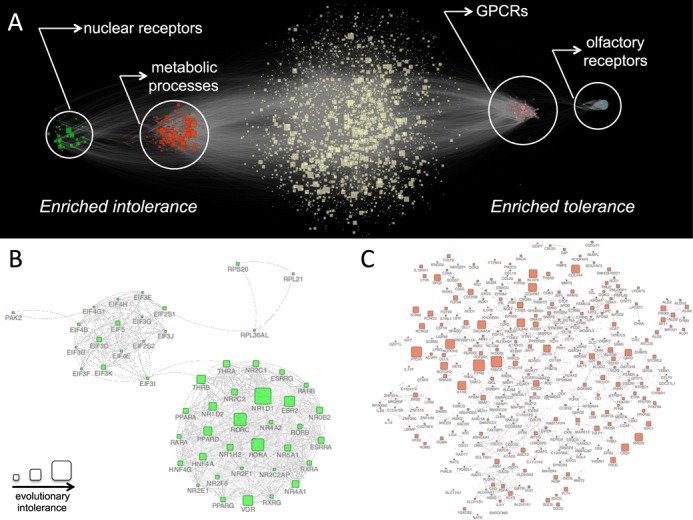
Clustering of the human interactome network revealed functionally coherent subnetworks with high or low evolutionary intolerance to mutations. (A) The whole interactome network where four subnetworks have been highlighted, representing clusters enriched for intolerant (left) and tolerant genes (right). Each node in the network represents a gene where the size of the node is scaled by its evolutionary intolerance score. (B) The subnetwork most enriched for intolerance (MWT, *P* = 7.5 × 10^−8^) that has significant functional enrichment for nuclear receptors. (C) The subnetwork most enriched for intolerance (MWT, *P =* 1.2 × 10^−5^) that has significant functional enrichment for metabolic processes (see Supplementary Table S6 for additional details on functional annotation for subnetworks).

In order to show that EvoTol is also useful when integrated with multiple genes at the network level we set out to investigate whether EvoTol can provide insights into disease susceptibility for multiple members of the same gene (or protein) network or regulatory program. To this aim we extended the EvoTol analysis to the level of protein interaction networks using the STRING database (http://string-db.org/) (27). The whole STRING protein-protein interaction (PPI) network was first analyzed to remove low quality edges and then clustered using the MCODE algorithm (see Materials and Methods) to derive gene networks for EvoTol analysis. Briefly, the MCODE algorithm finds densely connected regions within the large PPI network and therein identifies discrete clusters with above average ‘within-cluster’ connections compared to ‘out of cluster’ connections (28,29). Using this approach we found a total of 146 distinct PPI clusters, where only 41 contained more than 10 nodes. In order to test whether any of these clusters show a higher than expected evolutionary intolerance we used the non-parametric Mann–Whitney U test (MWT) to compare the distributions of intolerance scores for genes within and outside each subnetwork. This identified four subnetworks with significantly increased (or decreased) intolerance using a stringent Bonferroni threshold to account for multiple testing (Supplementary Figure S3). DAVID analysis showed significant functional specialization of these clusters within the larger PPI network (Figure 7A and Supplementary Table S6). The most tolerant PPI subnetworks included olfactory receptor genes and G-protein-coupled receptors (GPCRs) (MWT for tolerance, *P* = 4.6 × 10^−22^ and *P* = 9.7 × 10^−6^, respectively, that were below the threshold of significance after correction for multiple testing, *P* = 6.4 × 10^−5^). Olfactory receptors represent an ancient sensory system allowing an organism to detect chemicals in its environment (55), which have been shown to exhibit increased rates of molecular evolution relative to other (non-chemosensory) GPCRs (56), therefore, suggesting high tolerance to genetic variation for this cluster of genes. The most intolerant PPI subnetwork (MWT for intolerance*, P* = 7.5 × 10^−8^) was highly enriched for ligand-dependent nuclear receptor activity genes (*P* = 1.6 × 10^−59^), Figure 7B and Supplementary Table S6. Specifically, this subnetwork contains hormone-sensing proteins that can act as transcription factors and have a broad functional role from coordinating development to controlling metabolism (57). The other highly intolerant subnetwork (MWT for intolerance*, P* = 1.2 × 10^−5^) was highly enriched for genes with disease-causing mutation (DAVID analysis, SP_PIR_KEYWORDS ‘disease mutation’, *P* = 8.4 × 10^−29^), Figure 7C. Specifically, out of the 334 subnetwork genes, 97 (29%) genes had at least one variant responsible for a disease according to UniProt db, an enrichment that we confirmed by looking at the OMIM database where we found 103 genes (31%) with at least one phenotype-causing mutation representing a significant enrichment with respect of a genome-wide expectation (hypergeometric test *P* = 5.7 × 10^−13^). Notably, this global enrichment for disease causing genes by OMIM was not captured by RVIS analysis: RVIS classified only 8 genes as intolerant (not identified by EvoTol) and conversely EvoTol uniquely classified 34 genes as intolerant (not detected by RVIS) (Supplementary Figure S4). Functionally, this cluster was also enriched for genes involved in metabolic processes, such as glycerolipid metabolism (*P* = 4.7 × 10^−7^) and KEGG pathway ‘alanine, asparate and glutamate metabolism’ (*P* = 3.6 × 10^−8^), and including several genes encoding for transferase proteins (*P* = 3.1 × 10^−16^). Since transferases play an essential enzymatic function in hundreds of biochemical pathways it follows that mutations in this class of proteins are likely to have a large impact on key metabolic functions and related disorders. Similar analyses with RVIS failed to identify significant enrichment for intolerance for any subnetwork but, consistently with EvoTol, RVIS identified the olfactory receptor cluster as highly tolerant (data not shown). These results show that the integration of EvoTol with gene network information can be used to prioritize and annotate both intolerant and tolerant genes as well as gene networks.

### Online tool to prioritize evolutionary intolerant genes

To make the EvoTol method easy to access by the general scientific community we have designed a fast and intuitive web-based tool for evolutionary intolerance analysis, www.evotol.co.uk. We developed an easy to use graphical user interface where the user can input sets of genes (or proteins) to retrieve a ranked list of genes (or proteins) with the corresponding EvoTol score for intolerance. In addition, EvoTol can integrate cell-type-specific gene expression (from the FANTOM5 consortium (21)) classified using the UBERON cell-type ontology (22). Integrating information on cell-type-specific expression allows EvoTol to remove non-expressed genes before the assignment of genes to percentiles of evolutionary intolerance hence taking into account the specific tissue and cellular context where the gene is expressed. In addition, this framework represents a template that is amenable to inclusion of additional sources of data, including gene expression profiles from GEO or protein expression profiles in specific tissue types.

## CONCLUSIONS

In this study we utilized evolutionary information analysis of the predicted functional and phenotypic consequences of amino acid sequence variation to develop an integrated computational framework, EvoTol, to prioritize disease genes on the basis of their intolerance to mutation. Using known gene-phenotype associations and different levels of gene function annotation, we demonstrated that EvoTol uncovers intolerant genes more accurately than RVIS (11), in particular showing increased power and sensitivity to differentiate genes with predicted high pathogenicity. However, of additional interest is that the RVIS and EvoTol scores do not correlate strongly with each other (data not shown) and since these are able to predict disease genes reliably, the application of both scores in parallel will likely be of complimentary benefit. We provide a single, easy-to-use integrated approach to prioritize pathogenic genes (www.evotol.co.uk), which allows the systematic annotation of genomic sequence and mutation data from large-scale sequencing studies. To illustrate this point, we have applied EvoTol to the analysis of two separate WES data sets and showed how EvoTol provides a powerful framework to prioritize candidate disease-causing genes in epilepsy and CHD. We also showed EvoTol can be integrated with other data sources, increasing its ability to prioritize disease-causing genes operating in specific tissues or cellular contexts (when this information is available). For instance, stratifying genes by their expression patterns across more than 700 cell types and tissues results in up to 7-fold increase in EvoTol's ability to classify disease-causing genes. Another gene network-level application of EvoTol to the analysis of the human interactome (STRING (27)) revealed two highly intolerant networks enriched for nuclear receptors and genes involved in the regulation of metabolic processes. These intolerant networks were not classified as such by RVIS, however, the contribution of these network genes to cause Mendelian or complex disease has been confirmed by analysis of separate functional annotation data (e.g. OMIM, UniProt). As we move toward the personalized genomics era and massive genome sequence data from patients are made available to the scientific community, EvoTol can provide a simple and intuitive tool to prioritize new gene discovery.

## SUPPLEMENTARY DATA

Supplementary Data are available at NAR Online.

SUPPLEMENTARY DATA

## References

[1] Allen A.S., Berkovic S.F., Cossette P., Delanty N., Dlugos D., Eichler E.E., Epstein M.P., Glauser T., Goldstein D.B., Han Y. (2013). De novo mutations in epileptic encephalopathies. Nature.

[2] Fromer M., Pocklington A.J., Kavanagh D.H., Williams H.J., Dwyer S., Gormley P., Georgieva L., Rees E., Palta P., Ruderfer D.M. (2014). De novo mutations in schizophrenia implicate synaptic networks. Nature.

[3] Zaidi S., Choi M., Wakimoto H., Ma L., Jiang J., Overton J.D., Romano-Adesman A., Bjornson R.D., Breitbart R.E., Brown K.K. (2013). De novo mutations in histone-modifying genes in congenital heart disease. Nature.

[4] Neale B.M., Kou Y., Liu L., Ma'ayan A., Samocha K.E., Sabo A., Lin C.-F., Stevens C., Wang L.-S., Makarov V. (2012). Patterns and rates of exonic de novo mutations in autism spectrum disorders. Nature.

[5] MacArthur D.G., Manolio T.A., Dimmock D.P., Rehm H.L., Shendure J., Abecasis G.R., Adams D.R., Altman R.B., Antonarakis S.E., Ashley E.A. (2014). Guidelines for investigating causality of sequence variants in human disease. Nature.

[6] Adzhubei I., Jordan D.M., Sunyaev S.R. (2013). Predicting functional effect of human missense mutations using PolyPhen-2. Curr Protoc Hum Genet..

[7] Ng P.C., Henikoff S. (2003). SIFT: predicting amino acid changes that affect protein function. Nucleic Acids Res..

[8] Tranchevent L.-C., Barriot R., Yu S., Van Vooren S., Van Loo P., Coessens B., De Moor B., Aerts S., Moreau Y. (2008). ENDEAVOUR update: a web resource for gene prioritization in multiple species. Nucleic Acids Res..

[9] Franke L., van Bakel H., Fokkens L., de Jong E.D., Egmont-Petersen M., Wijmenga C. (2006). Reconstruction of a functional human gene network, with an application for prioritizing positional candidate genes. Am. J. Hum. Genet..

[10] Bromberg Y. (2013). Chapter 15: disease gene prioritization. PLoS Comput. Biol..

[11] Petrovski S., Wang Q., Heinzen E.L., Allen A.S., Goldstein D.B. (2013). Genic intolerance to functional variation and the interpretation of personal genomes. PLoS Genet..

[12] Thomas R.H., Berkovic S.F. (2014). The hidden genetics of epilepsy-a clinically important new paradigm. Nat. Rev. Neurol..

[13] Renkema K.Y., Stokman M.F., Giles R.H., Knoers N.V.A.M. (2014). Next-generation sequencing for research and diagnostics in kidney disease. Nat. Rev. Nephrol..

[14] Uddin M., Tammimies K., Pellecchia G., Alipanahi B., Hu P., Wang Z., Pinto D., Lau L., Nalpathamkalam T., Marshall C.R. (2014). Brain-expressed exons under purifying selection are enriched for de novo mutations in autism spectrum disorder. Nat. Genet..

[15] Chen Y.-Z., Friedman J.R., Chen D.-H., Chan G.C.-K., Bloss C.S., Hisama F.M., Topol S.E., Carson A.R., Pham P.H., Bonkowski E.S. (2014). Gain-of-function ADCY5 mutations in familial dyskinesia with facial myokymia. Ann. Neurol..

[16] Samocha K.E., Robinson E.B., Sanders S.J., Stevens C., Sabo A., McGrath L.M., Kosmicki J.A., Rehnström K., Mallick S., Kirby A. (2014). A framework for the interpretation of de novo mutation in human disease. Nat. Genet..

[17] Finn R.D., Mistry J., Tate J., Coggill P., Heger A., Pollington J.E., Gavin O.L., Gunasekaran P., Ceric G., Forslund K. (2010). The Pfam protein families database. Nucleic Acids Res..

[18] De Lima Morais D. A., Fang H., Rackham O.J.L., Wilson D., Pethica R., Chothia C., Gough J. (2011). SUPERFAMILY 1.75 including a domain-centric gene ontology method. Nucleic Acids Res..

[19] Shihab H. A., Gough J., Cooper D.N., Stenson P.D., Barker G.L.A., Edwards K.J., Day I.N.M., Gaunt T.R. (2013). Predicting the functional, molecular, and phenotypic consequences of amino acid substitutions using hidden Markov models. Hum. Mutat..

[20] Hamosh A., Scott A.F., Amberger J.S., Bocchini C.A., McKusick V.A. (2005). Online Mendelian Inheritance in Man (OMIM), a knowledgebase of human genes and genetic disorders. Nucleic Acids Res..

[21] Forrest A.R.R., Kawaji H., Rehli M., Kenneth Baillie J., de Hoon M.J.L., Haberle V., Lassmann T., Kulakovskiy I.V., Lizio M., Itoh M. (2014). A promoter-level mammalian expression atlas. Nature.

[22] Mungall C.J., Torniai C., Gkoutos G. V, Lewis S.E., Haendel M.A. (2012). Uberon, an integrative multi-species anatomy ontology. Genome Biol..

[23] Von Mering C., Huynen M., Jaeggi D., Schmidt S., Bork P., Snel B. (2003). STRING: a database of predicted functional associations between proteins. Nucleic Acids Res..

[24] Sherry S.T., Ward M.H., Kholodov M., Baker J., Phan L., Smigielski E.M., Sirotkin K. (2001). dbSNP: the NCBI database of genetic variation. Nucleic Acids Res..

[25] Report S. (2012). Epi4K: gene discovery in 4,000 genomes. Epilepsia.

[26] Allen A.S., Berkovic S.F., Cossette P., Delanty N., Dlugos D., Eichler E.E., Epstein M.P., Glauser T., Goldstein D.B., Han Y. (2013). De novo mutations in epileptic encephalopathies. Nature.

[27] Franceschini A., Szklarczyk D., Frankild S., Kuhn M., Simonovic M., Roth A., Lin J., Minguez P., Bork P., von Mering C. (2013). STRING v9.1: protein-protein interaction networks, with increased coverage and integration. Nucleic Acids Res..

[28] Bader G.D., Hogue C.W.V. (2003). An automated method for finding molecular complexes in large protein interaction networks. BMC Bioinformatics.

[29] Altaf-Ul-Amin M., Shinbo Y., Mihara K., Kurokawa K., Kanaya S. (2006). Development and implementation of an algorithm for detection of protein complexes in large interaction networks. BMC Bioinformatics.

[30] Smoot M.E., Ono K., Ruscheinski J., Wang P.-L., Ideker T. (2011). Cytoscape 2.8: new features for data integration and network visualization. Bioinformatics.

[31] Dennis G., Sherman B.T., Hosack D.A., Yang J., Gao W., Lane H.C., Lempicki R.A. (2003). DAVID: Database for Annotation, Visualization, and Integrated Discovery. Genome Biol..

[32] Bragin E., Chatzimichali E.A., Wright C.F., Hurles M.E., Firth H. V, Bevan A.P., Swaminathan G.J. (2014). DECIPHER: database for the interpretation of phenotype-linked plausibly pathogenic sequence and copy-number variation. Nucleic Acids Res..

[33] Sardar A.J., Oates M.E., Fang H., Forrest A.R.R., Kawaji H., Gough J., Rackham O.J.L. (2014). The evolution of human cells in terms of protein innovation. Mol. Biol. Evol..

[34] Maugeri A., Klevering B.J., Rohrschneider K., Blankenagel A., Brunner H.G., Deutman A.F., Hoyng C.B., Cremers F.P. (2000). Mutations in the ABCA4 (ABCR) gene are the major cause of autosomal recessive cone-rod dystrophy. Am. J. Hum. Genet..

[35] Escayg A., Goldin A.L. (2010). Sodium channel SCN1A and epilepsy: mutations and mechanisms. Epilepsia.

[36] Neubauer B.A., Waldegger S., Heinzinger J., Hahn A., Kurlemann G., Fiedler B., Eberhard F., Muhle H., Stephani U., Garkisch S. (2008). KCNQ2 and KCNQ3 mutations contribute to different idiopathic epilepsy syndromes. Neurology.

[37] Mulley J.C., Scheffer I.E., Petrou S., Dibbens L.M., Berkovic S.F., Harkin L.A. (2005). SCN1A mutations and epilepsy. Hum. Mutat..

[38] Catterall W.A., Kalume F., Oakley J.C. (2010). NaV1.1 channels and epilepsy. J. Physiol..

[39] Nieuwenhuis E., Hui C. (2005). Hedgehog signaling and congenital malformations. Clin. Genet..

[40] Frédéric M.Y., Monino C., Marschall C., Hamroun D., Faivre L., Jondeau G., Klein H.-G., Neumann L., Gautier E., Binquet C. (2009). The FBN2 gene: new mutations, locus-specific database (Universal Mutation Database FBN2), and genotype-phenotype correlations. Hum. Mutat..

[41] Marguerie A., Bajolle F., Zaffran S., Brown N.A., Dickson C., Buckingham M.E., Kelly R.G. (2006). Congenital heart defects in Fgfr2-IIIb and Fgf10 mutant mice. Cardiovasc. Res..

[42] Kantarci S., Al-Gazali L., Hill R.S., Donnai D., Black G.C.M., Bieth E., Chassaing N., Lacombe D., Devriendt K., Teebi A. (2007). Mutations in LRP2, which encodes the multiligand receptor megalin, cause Donnai-Barrow and facio-oculo-acoustico-renal syndromes. Nat. Genet..

[43] Willnow T.E., Hilpert J., Armstrong S.A., Rohlmann A., Hammer R.E., Burns D.K., Herz J. (1996). Defective forebrain development in mice lacking gp330/megalin. Proc. Natl. Acad. Sci. U.S.A..

[44] Thiery E., Gosset P., Damotte D., Delezoide A.L., de Saint-Sauveur N., Vayssettes C., Créau N. (2000). Developmentally regulated expression of the murine ortholog of the potassium channel KIR4.2 (KCNJ15). Mech. Dev..

[45] Wulff H., Castle N.A., Pardo L.A. (2009). Voltage-gated potassium channels as therapeutic targets. Nat. Rev. Drug Discov..

[46] Yang T., Yang P., Roden D.M., Darbar D. (2010). Novel KCNA5 mutation implicates tyrosine kinase signaling in human atrial fibrillation. Heart Rhythm.

[47] Hong K., Piper D.R., Diaz-Valdecantos A., Brugada J., Oliva A., Burashnikov E., Santos-de-Soto J., Grueso-Montero J., Diaz-Enfante E., Brugada P. (2005). De novo KCNQ1 mutation responsible for atrial fibrillation and short QT syndrome in utero. Cardiovasc. Res..

[48] Wenzel J.J., Kaminski W.E., Piehler A., Heimerl S., Langmann T., Schmitz G. (2003). ABCA10, a novel cholesterol-regulated ABCA6-like ABC transporter. Biochem. Biophys. Res. Commun..

[49] Murphy A.J., Sarrazy V., Wang N., Bijl N., Abramowicz S., Westerterp M., Welch C.B., Schuetz J.D., Yvan-Charvet L. (2014). Deficiency of ATP-binding cassette transporter b6 in megakaryocyte progenitors accelerates atherosclerosis in mice. Arterioscler. Thromb. Vasc. Biol..

[50] Oram J.F., Lawn R.M. (2001). ABCA1: the gatekeeper for eliminating excess tissue cholesterol. J. Lipid Res..

[51] Ruiz-Perez V.L., Carter S.A., Healy E., Todd C., Rees J.L., Steijlen P.M., Carmichael A.J., Lewis H.M., Hohl D., Itin P. (1999). ATP2A2 mutations in Darier's disease: variant cutaneous phenotypes are associated with missense mutations, but neuropsychiatric features are independent of mutation class. Hum. Mol. Genet..

[52] Wahlquist C., Jeong D., Rojas-Muñoz A., Kho C., Lee A., Mitsuyama S., van Mil A., Park W.J., Sluijter J.P.G., Doevendans P.A.F. (2014). Inhibition of miR-25 improves cardiac contractility in the failing heart. Nature.

[53] Meyer M., Schillinger W., Pieske B., Holubarsch C., Heilmann C., Posival H., Kuwajima G., Mikoshiba K., Just H., Hasenfuss G. (1995). Alterations of sarcoplasmic reticulum proteins in failing human dilated cardiomyopathy. Circulation.

[54] Lage K., Hansen N.T., Karlberg E.O., Eklund A.C., Roque F.S., Donahoe P.K., Szallasi Z., Jensen T.S., Brunak S. (2008). A large-scale analysis of tissue-specific pathology and gene expression of human disease genes and complexes. Proc. Natl. Acad. Sci. U.S.A..

[55] Gaillard I., Rouquier S., Giorgi D. (2004). Olfactory receptors. Cell. Mol. Life Sci..

[56] Spielman S.J., Wilke C.O. (2013). Membrane environment imposes unique selection pressures on transmembrane domains of G protein-coupled receptors. J. Mol. Evol..

[57] Sonoda J., Pei L., Evans R.M. (2008). Nuclear receptors: decoding metabolic disease. FEBS Lett..

