# Supplementary material for: EvoTol: a protein-sequence based evolutionary intolerance framework for disease-gene prioritization
Source: Nucleic Acids Res. 2014 Dec 29;43(5):e33. doi: 10.1093/nar/gku1322 (PMC4357693; doi:10.1093/nar/gku1322)
Supplement: SUPPLEMENTARY DATA [file supp_43_5_e33__index.html]

EvoTol: a protein-sequence based evolutionary intolerance framework for disease-gene prioritization — SUPPLEMENTARY DATA 

# EvoTol: a protein-sequence based evolutionary intolerance framework for disease-gene prioritization

## SUPPLEMENTARY DATA

**Files in this Data Supplement:**

- SUPPLEMENTARY DATA
