# Supplementary material for: EvoTol: a protein-sequence based evolutionary intolerance framework for disease-gene prioritization
Source: Nucleic Acids Res. 2014 Dec 29;43(5):e33. doi: 10.1093/nar/gku1322 (PMC4357693; doi:10.1093/nar/gku1322)

Supplemental Figure 1. ROC curves for EvoTol and RVIS, showing increased ability of EvoTol (red) to predict disease genes as compared with RVIS (blue) for all OMIM gene lists.

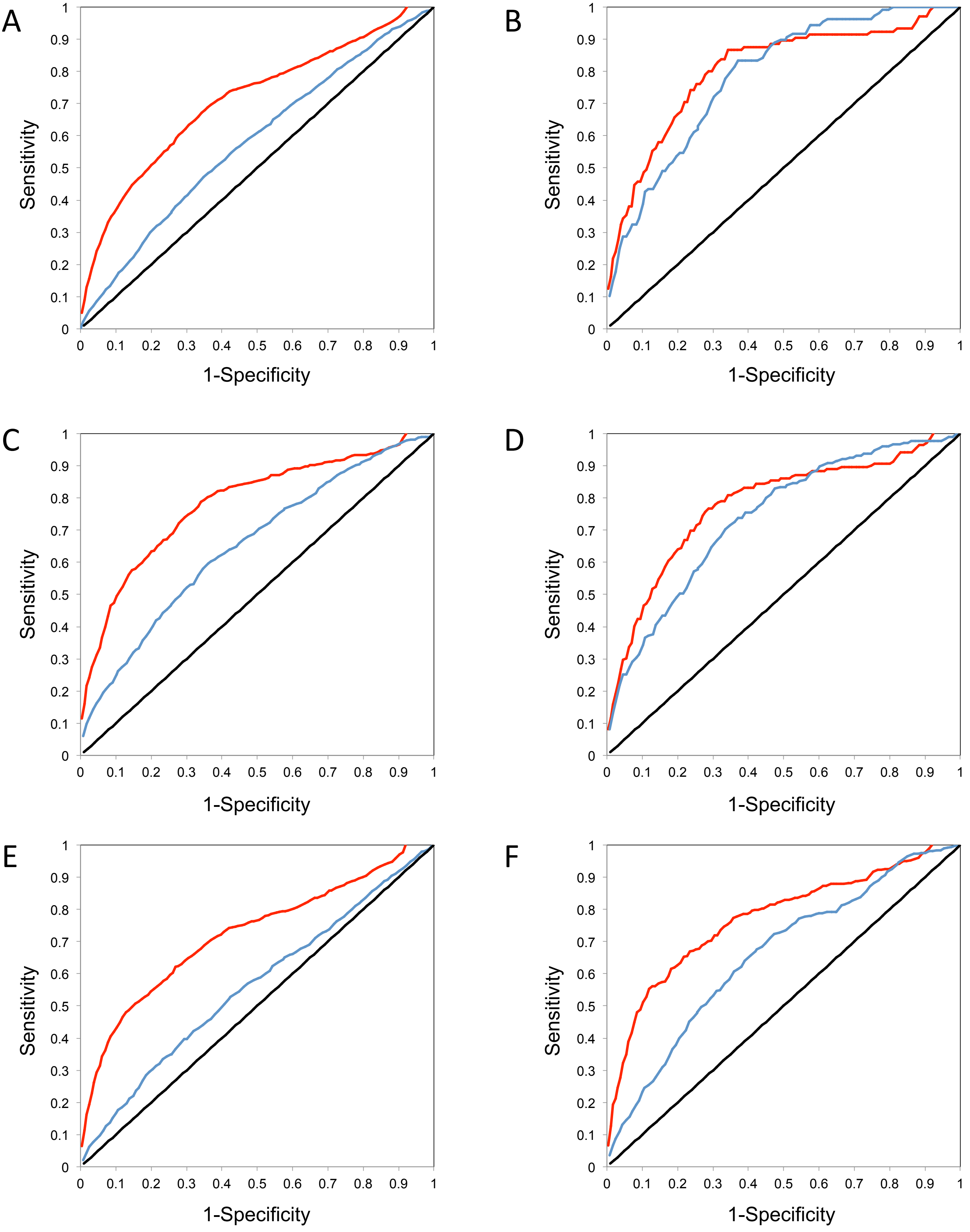

OMIM genes categories:

A. all (n=2131)
B. *de novo* disease causing and haploinsufficient (n=108)
C. *de novo* disease causing (n=467)
D. haploinsufficient (n=175)
E. recessive (n=817)
F. dominant-negative (n=101)

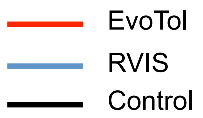

Supplement: SUPPLEMENTARY DATA [file supp_gku1322_nar-02497-met-n-2014-File009.zip › Supp/Supplemental Figure 1.pdf]
