# Supplementary material for: EvoTol: a protein-sequence based evolutionary intolerance framework for disease-gene prioritization
Source: Nucleic Acids Res. 2014 Dec 29;43(5):e33. doi: 10.1093/nar/gku1322 (PMC4357693; doi:10.1093/nar/gku1322)

Supplemental Figure 2. Fold enrichment in prediction of disease genes by EvoTol that was observed by stratifying genes according to their expression levels in different systems. For each of the systems in the Uberon cell-ontology (see Methods) we defined lists of genes detected at different cut-offs of expression (tags per million or TPM, x-axes) and calculated the fold enrichment in predicting disease genes by EvoTol (y-axes) (see Methods). For both the UniProt "epilepsy" (A) and "congenital heart disease" (B) genes, the fold enrichment in prediction of disease causing genes by EvoTol reaches its maximum when expressed genes are selected using a threshold of 100 – 200 TPMs. At higher thresholds (TMP > 500) the number of expressed genes drops significantly (less than hundred) therefore defining is very small lists of detected genes. This decreases the likelihood that any disease gene predicted by EvoTol is represented within the list of expressed genes (i.e., no genes predicted results in fold enrichment = 0). These data suggest that the highest fold enrichments in predicting disease genes were observed by stratifying expressed genes setting the threshold at ~ 100 TPM.

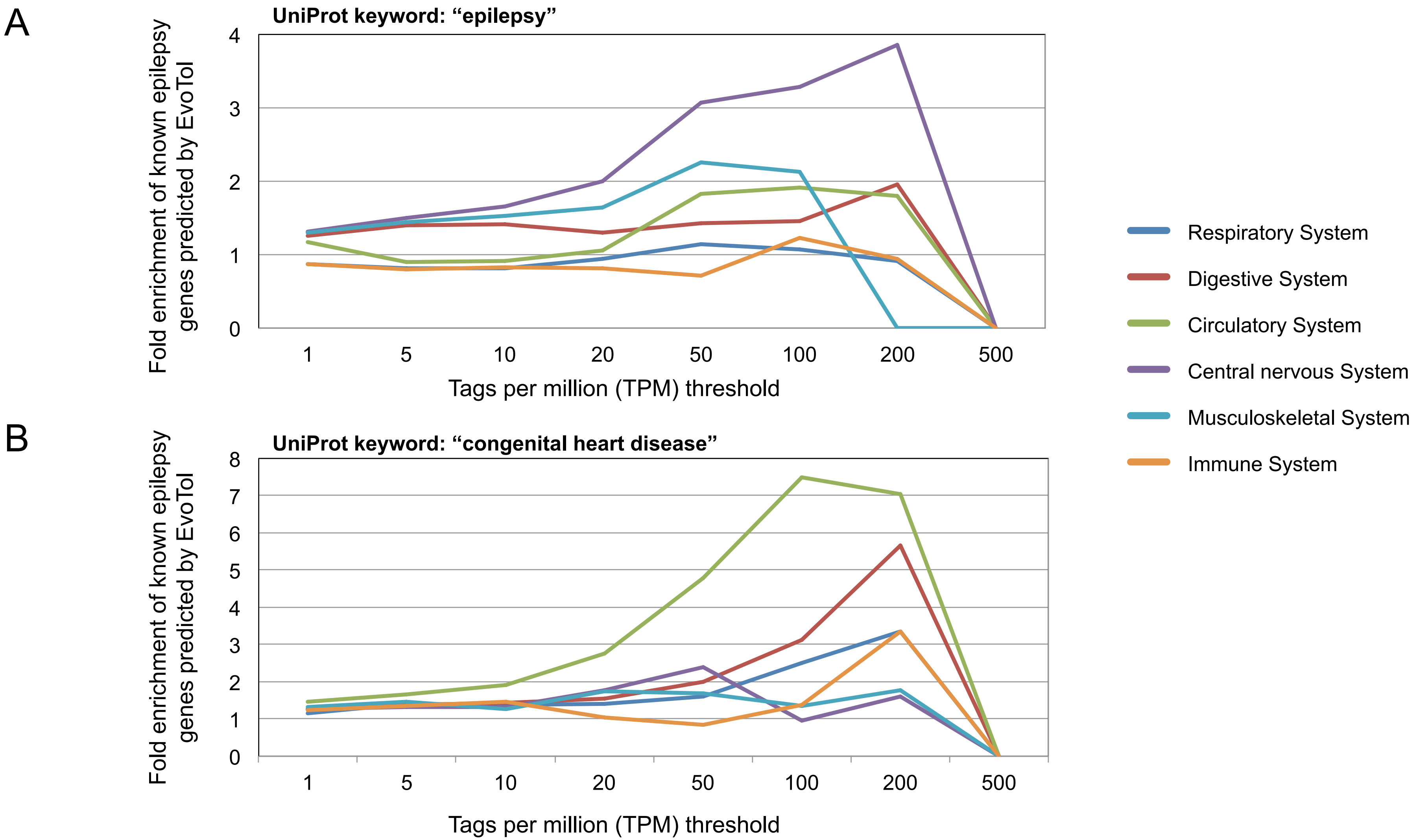

Supplement: SUPPLEMENTARY DATA [file supp_gku1322_nar-02497-met-n-2014-File009.zip › Supp/Supplemental Figure 2.pdf]
