# Supplementary material for: EvoTol: a protein-sequence based evolutionary intolerance framework for disease-gene prioritization
Source: Nucleic Acids Res. 2014 Dec 29;43(5):e33. doi: 10.1093/nar/gku1322 (PMC4357693; doi:10.1093/nar/gku1322)

Supplemental Figure 3. 146 STRING sub-networks identified by MCODE that have above (or below) expected intolerance score with respect to the rest of the STRING network. Red, intolerant PPI sub-network; blue, tolerant PPI sub-network. Red line, Bonferroni corrected threshold of significance (P = 3.4 x 10^{-4}). PPI sub-networks are ordered by decreasing MCODE score (Smoot et al. 2011) which ranks larger, more dense clusters at the top of the list.

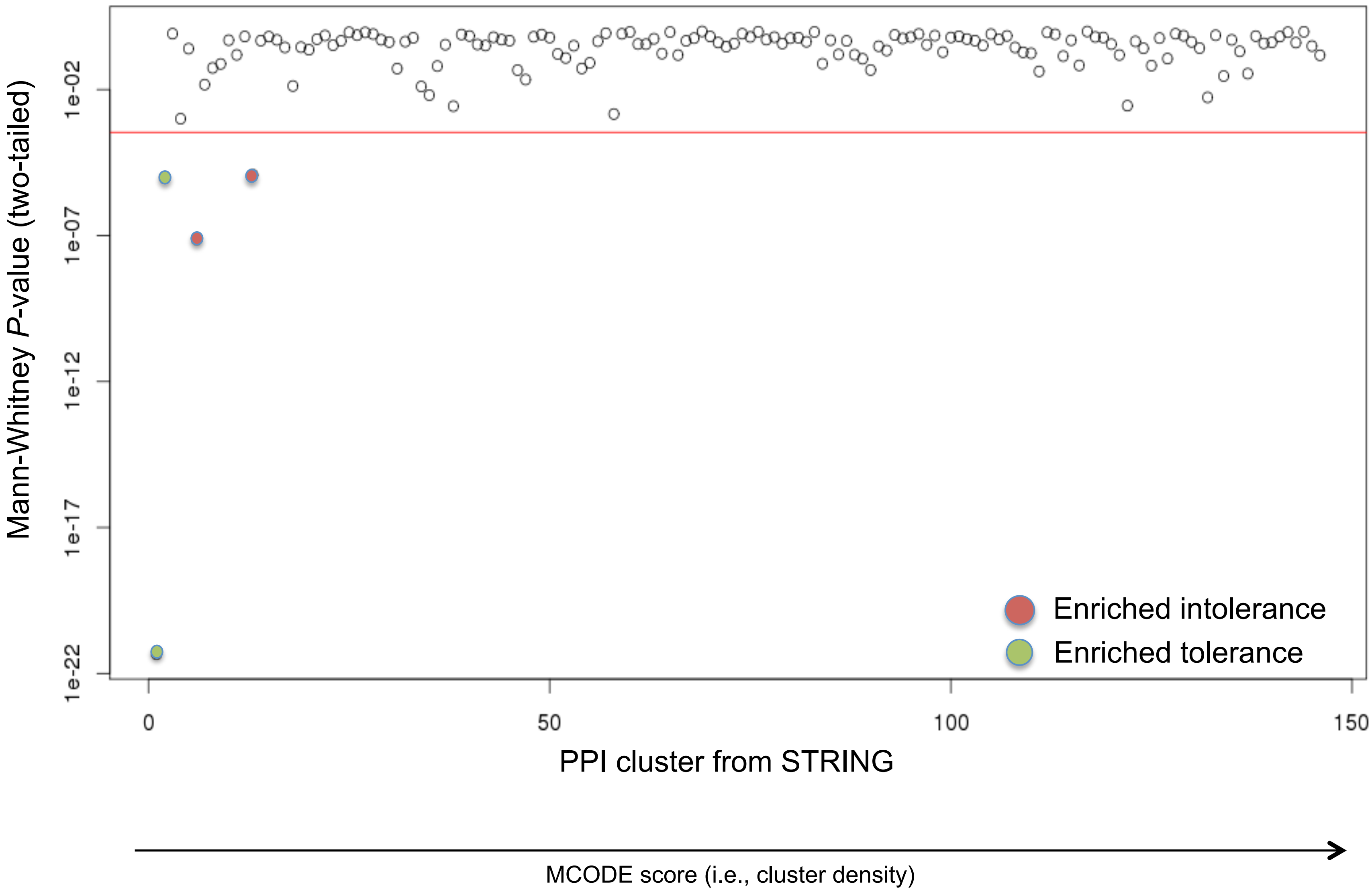

Supplement: SUPPLEMENTARY DATA [file supp_gku1322_nar-02497-met-n-2014-File009.zip › Supp/Supplemental Figure 3.pdf]
