# Supplementary material for: EvoTol: a protein-sequence based evolutionary intolerance framework for disease-gene prioritization
Source: Nucleic Acids Res. 2014 Dec 29;43(5):e33. doi: 10.1093/nar/gku1322 (PMC4357693; doi:10.1093/nar/gku1322)

Supplemental Figure 4. For each of the genes in cluster 4 (Supplementary Table 6), we report the intolerance (y-axes) where high values indicate high-intolerance, and the associated disease phenotype by OMIM database (x-axes). The dotted line indicates the 25 percentile of intolerance, previously adopted to identify disease genes (Petrovski et al. 2013). Apart from 8 genes with OMIM phenotype that have been classified as intolerant only by RVIS (blue bars with intolerance >75% shown at the right-hand side in the graph), EvoTol predictions of intolerance were very highly consistent with the gene-disease phenotype associations from the OMIM database. In comparison, 34 genes (indicated by a star in the graph) with with at least one phenotype-causing mutation by OMIM were predicted to be intolerant by EvoTol but not by RVIS.

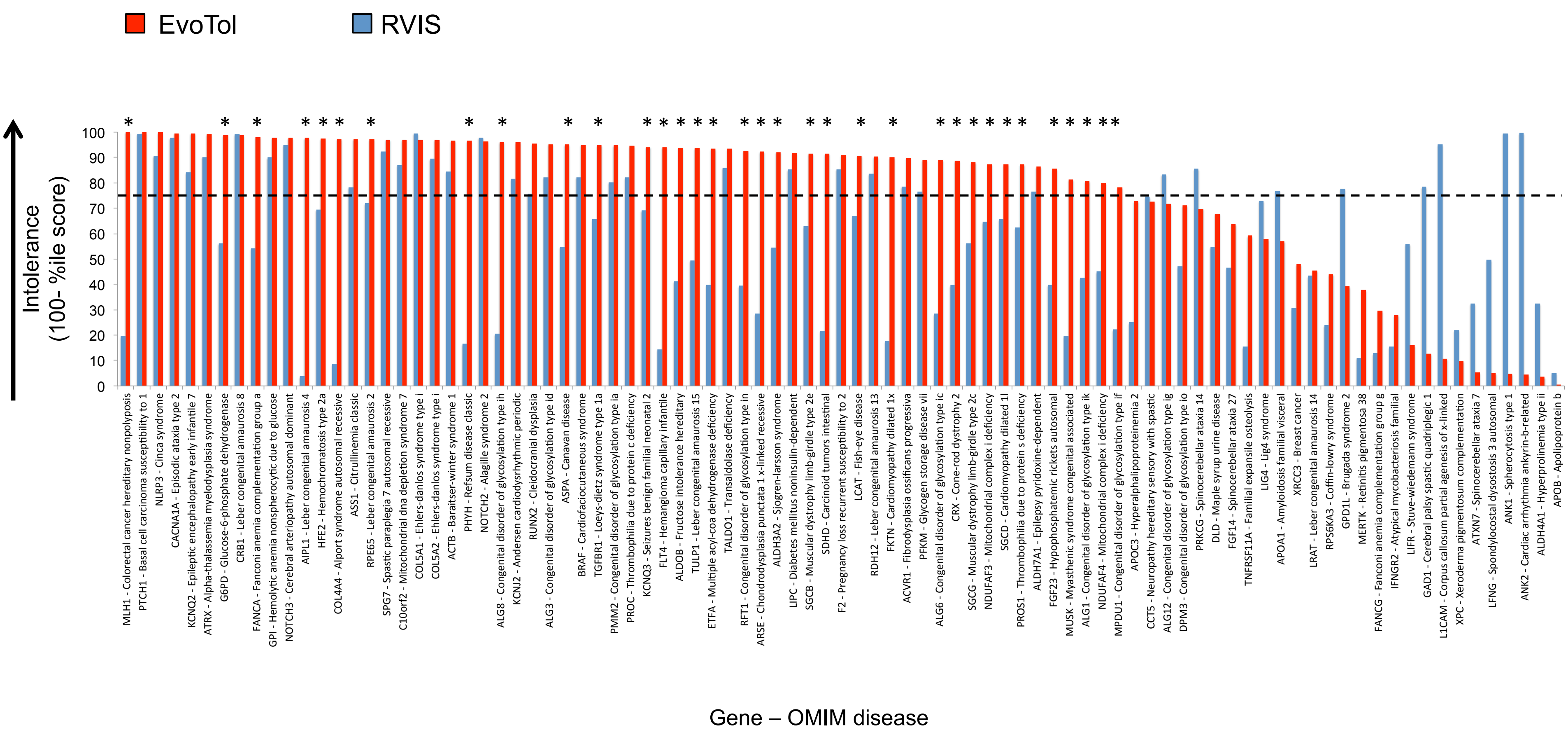

Supplement: SUPPLEMENTARY DATA [file supp_gku1322_nar-02497-met-n-2014-File009.zip › Supp/Supplemental Figure 4 .pdf]
