# Supplementary material for: EvoTol: a protein-sequence based evolutionary intolerance framework for disease-gene prioritization
Source: Nucleic Acids Res. 2014 Dec 29;43(5):e33. doi: 10.1093/nar/gku1322 (PMC4357693; doi:10.1093/nar/gku1322)

Supplementary Figure 5: Comparing RVIS, gene constraint score and Evotol (dbSNP and EVS derived) shows that regardless of the data source the EvoTol approach performs best in the critical region of the ROC curve (ie low FPR).

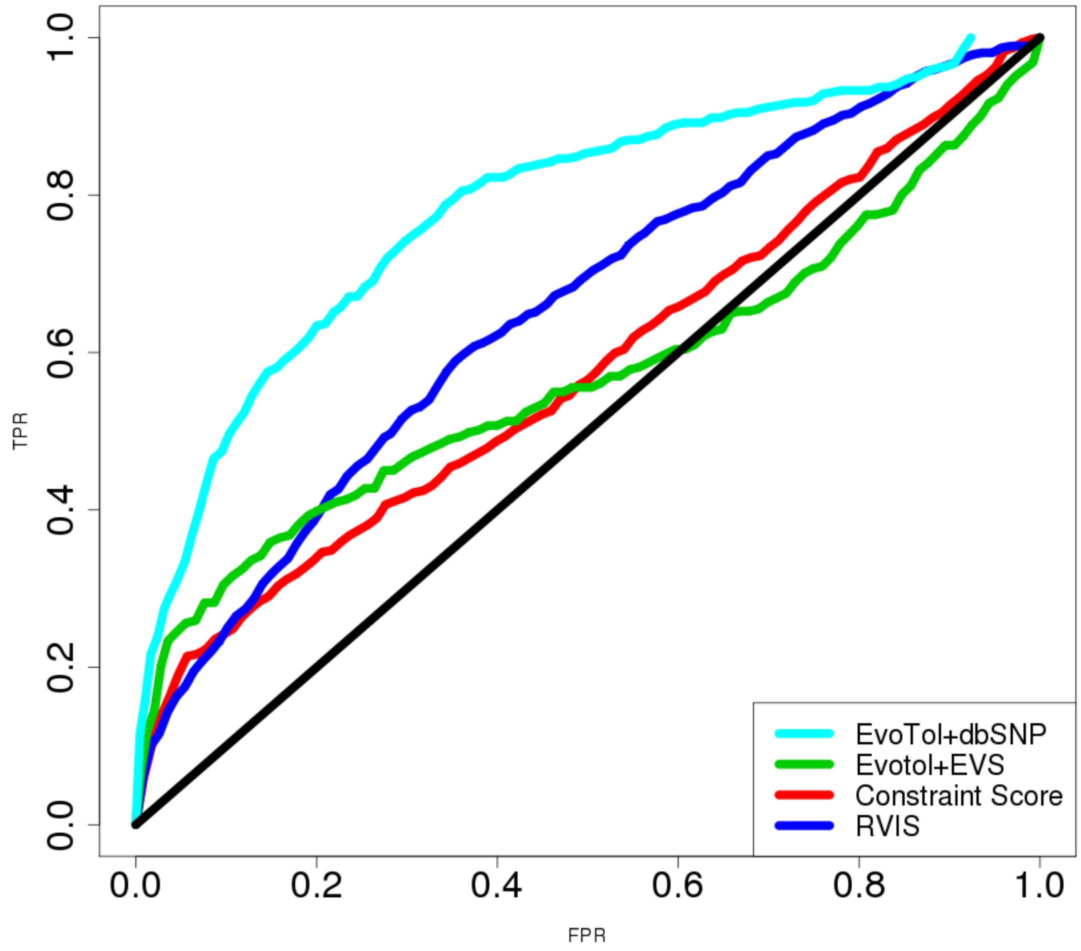

Supplement: SUPPLEMENTARY DATA [file supp_gku1322_nar-02497-met-n-2014-File009.zip › Supp/Supplemental Figure 5.pdf]
