# Supplementary material for: EvoTol: a protein-sequence based evolutionary intolerance framework for disease-gene prioritization
Source: Nucleic Acids Res. 2014 Dec 29;43(5):e33. doi: 10.1093/nar/gku1322 (PMC4357693; doi:10.1093/nar/gku1322)

Supplementary Figure 6: Comparing EvoTol for Genes and Domains shows that using domains is as accurate as genes in the critical region of the ROC (low FDR) and outperforms the gene level elsewhere. The domain level ROC is produced by ranking genes based on their most intolerant protein domain and using this to identify known disease causing genes from OMIM.

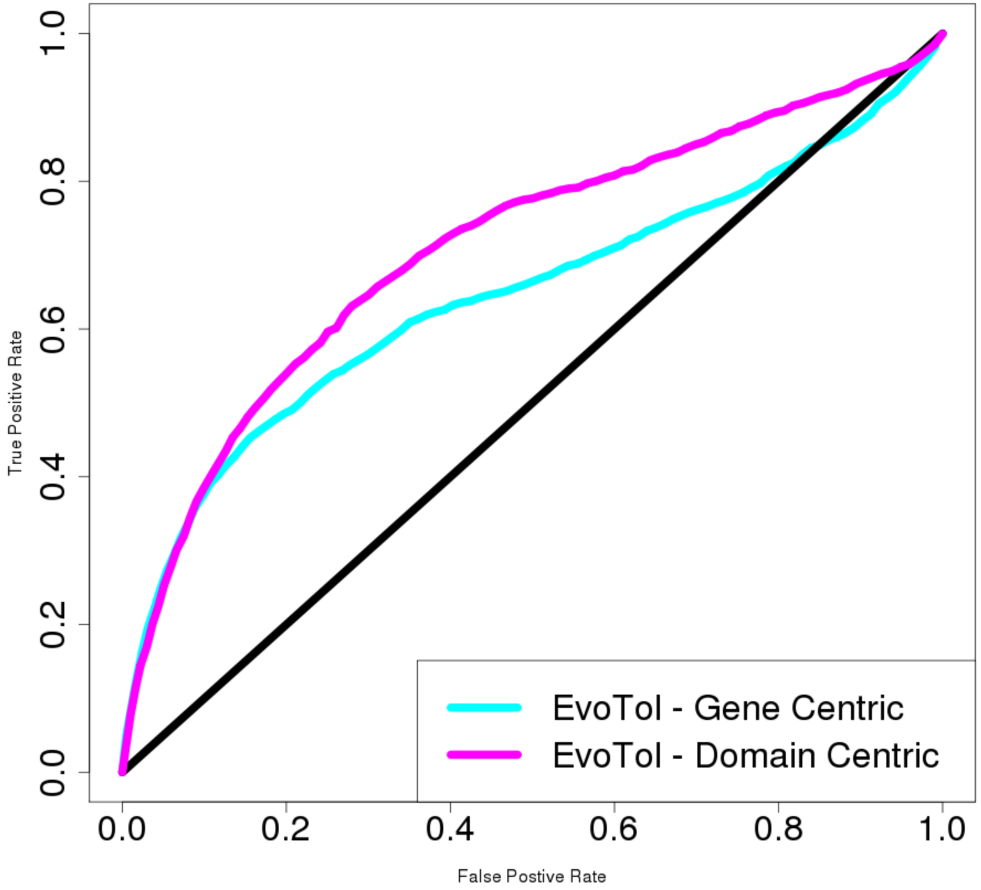

Supplement: SUPPLEMENTARY DATA [file supp_gku1322_nar-02497-met-n-2014-File009.zip › Supp/Supplemental Figure 6.pdf]
