# Supplementary material for: EvoTol: a protein-sequence based evolutionary intolerance framework for disease-gene prioritization
Source: Nucleic Acids Res. 2014 Dec 29;43(5):e33. doi: 10.1093/nar/gku1322 (PMC4357693; doi:10.1093/nar/gku1322)
Supplement: SUPPLEMENTARY DATA [file supp_gku1322_nar-02497-met-n-2014-File009.zip › Supp/Supplemental Table 1.pdf]

Supplemental Table 1. The most 10 intolerant genes according to evolutionary intolerance (EvoTol) and corresponding genic intolerance scores (RVIS, Petrovski et al. *PLoS Genetics* 2013; 9(8):e1003709). (1) http://fathmm.biocompute.org.uk/; (2) http://evs.gs.washington.edu/EVS/; (3) http://genetics.bwh.harvard.edu/pph2/: (4) https://www.ncbi.nlm.nih.gov/clinvar/; (5) http://www.omim.org. Bold font, genes predicted to be highly intolerant (tolerant) by EvoTol but not by RVIS.

				Number of SNVs in each gene and annotation						
Gene symbol	Gene Description	EvoTol score percentile	RVIS percentile	**FATHMM**[1] (total/tolerated)	[damaging/total] (%)	**EVS**[2] (total,0.1%,1%)	**PolyPhen**[3] (benign/possibly damaging/probably damaging/unknown)	[(Possibly damaging+probably damaging/(possibly damaging+probably damaging+benign)] (%)	**ClinVar**[4] (pathogenic variants/Mbp)	**OMIM**[5] (associated phonotypes)
SCN5A	sodium channel, voltage-gated, type V, alpha subunit	<0.001	1.99	594/86	86%	327/52/20	36/13/70/217	70%	0.57	9
RYR1	ryanodine receptor 1 (skeletal)	0.006	0.01	789/343	57%	1074/209/95	46/56/250/723	87%	0.70	5
BRCA1	breast cancer 1, early onset	0.01	78.6	720/294	59%	237/34/16	49/29/54/196	63%	5.23	2
KCNH2	potassium voltage-gated channel, subfamily H (eag-related), member 2	0.02	3.81	469/64	86%	164/29/15	14/13/23/118	72%	1.29	3
ABCA4	ATP-binding cassette, sub-family A (ABC1), member 4	0.02	86.31	538/149	72%	572/270/104	95/27/95/355	56%	0.41	6
MLL3	myeloid/lymphoid or mixed-lineage leukemia 3	0.03	0.91	629/277	56%	616/268/86	116/51/113/336	59%	0.05	-
LRP2	low density lipoprotein receptor-related protein 2	0.03	99.42	536/189	65%	806/405/191	133/66/155/452	62%	0.08	1
PAH	phenylalanine hydroxylase	0.04	14.67	345/31	91%	112/58/24	9/4/20/79	73%	0.83	2
FBN3	fibrillin 3	0.05	99.89	494/183	63%	832/472/242	121/52/160/499	64%	0.04	-
HBB	hemoglobin, beta	0.05	53.51	316/14	96%	28/9/4	2/0/1/25	50%	45.27	9
