# Supplementary material for: EvoTol: a protein-sequence based evolutionary intolerance framework for disease-gene prioritization
Source: Nucleic Acids Res. 2014 Dec 29;43(5):e33. doi: 10.1093/nar/gku1322 (PMC4357693; doi:10.1093/nar/gku1322)
Supplement: SUPPLEMENTARY DATA [file supp_gku1322_nar-02497-met-n-2014-File009.zip › Supp/Supplemental Table 2.pdf]

Supplemental Table 2. EvoTol scores for the 176 genes with de novo mutations by the Epi4k consortium. The table is sorted according to increasing tolerance and for each gene the corresponding RVIS score is reported for comparison. For each gene we also report data on the known associated phenotype, which was retrieved by Ensembl Biomart (CRCh37.p13). The database sources of phenotypic associations are: [1]Online Mendelian Inheritance in Man (OMIM, http://www.omim.org); [2]Developmental Disorders Gene to Phenotype (DDG2P) and [3]Orphanet (http://www.orpha.net). Grey, genes within the 25th percentile of intolerance. Bold, genes encoding proteins with the same protein domain (see Figure 4).

Gene Symbol	Gene description	EvoTol %ile	RVIS %ile	OMIM[1]	DDG2P[2]	Orphanet[3]
SCN1A	calcium channel, voltage-dependent, P/Q type, alpha 1A subunit [Source:HGNC Symbol;Acc:1388]	0.04	4.03	Dravet syndrome	Scn1a-related seizure disorders	Dravet syndrome
CACNA1A	potassium voltage-gated channel, KQT-like subfamily, member 2 [Source:HGNC Symbol;Acc:6296]	0.43	2.27	Episodic ataxia type 2	Epileptic encephalopathy	Alternating hemiplegia of childhood
KCNQ2	filamin C, gamma [Source:HGNC Symbol;Acc:3756]	0.51	15.86	Epileptic encephalopathy early infantile 7	Benign neonatal epilepsy type 1 (ebn1)	Benign familial infantile seizures
FLNC	collagen, type VII, alpha 1 [Source:HGNC Symbol;Acc:2214]	0.59	0.29	Myopathy distal 4	-	Distal myopathy with posterior leg and anterior hand involvement
COL7A1	filamin A, alpha [Source:HGNC Symbol;Acc:3754]	0.80	0.13	Epidermolysis bullosa dystrophica autosomal dominant	-	Acral dystrophic epidermolysis bullosa
FLNA	sodium channel, voltage-gated, type II, alpha subunit [Source:HGNC Symbol;Acc:10588]	0.91	0.44	Cardiac valvular dysplasia x-linked	Fg syndrome type 2 (fgs2)	Chronic intestinal pseudo-obstruction
SCN2A	ATPase, Ca++ transporting, plasma membrane 4 [Source:HGNC Symbol;Acc:817]	0.93	1.77	Epileptic encephalopathy early infantile 11	Benign familial neonatal infantile seizures	Benign familial neonatal-infantile seizures
ATP2B4	ATP-binding cassette, sub-family B (MDR/TAP), member 9 [Source:HGNC Symbol;Acc:50]	1.56	11.51	-	-	-
ABCB9	transmembrane phosphoinositide 3-phosphatase and tensin homolog 2 [Source:HGNC Symbol;Acc:17299]	1.74	2.16	-	-	-
TPTE2	myosin, heavy chain 6, cardiac muscle, alpha [Source:HGNC Symbol;Acc:7576]	2.27	40.45	-	-	-
MYH6	collagen, type IV, alpha 4 [Source:HGNC Symbol;Acc:2206]	2.40	0.66	Atrial septal defect 3	Atrial septal defect type 3 (asd3)	Atrial septal defect ostium secundum type
COL4A4	spastic paraplegia 7 (pure and complicated autosomal recessive) [Source:HGNC Symbol;Acc:11237]	2.70	91.27	Alport syndrome autosomal recessive	Alport syndrome autosomal recessive	Autosomal dominant alport syndrome
SPG7	integrin, beta 4 [Source:HGNC Symbol;Acc:6158]	2.97	7.62	Spastic paraplegia 7 autosomal recessive	-	Autosomal dominant optic atrophy and peripheral neuropathy
ITGB4	sodium channel, voltage gated, type VIII, alpha subunit [Source:HGNC Symbol;Acc:10596]	3.35	0.53	Epidermolysis bullosa junctionalis with pyloric atresia	-	Epidermolysis bullosa simplex with pyloric atresia
SCN8A	potassium voltage-gated channel, KQT-like subfamily, member 3 [Source:HGNC Symbol;Acc:6297]	4.46	2.34	Cognitive impairment with or without cerebellar ataxia	Cognitive impairment with or without cerebellar ataxia	Early infantile epileptic encephalopathy
KCNQ3	myosin VIIB [Source:HGNC Symbol;Acc:7607]	5.82	30.82	Seizures benign familial neonatal 2	-	Benign familial neonatal seizures
MYO7B	keratin 34 [Source:HGNC Symbol;Acc:6452]	6.15	90.92	-	-	-
KRT34	chromodomain helicase DNA binding protein 4 [Source:HGNC Symbol;Acc:1919]	6.24	89.56	-	-	-
CHD4	diaphanous-related formin 3 [Source:HGNC Symbol;Acc:15480]	6.36	2.82	-	-	-
DIAPH3	translin-associated factor X interacting protein 1 [Source:HGNC Symbol;Acc:18586]	6.79	66.27	Auditory neuropathy autosomal dominant 1	-	Autosomal dominant nonsyndromic sensorineural deafness type dfna
TSNAXIP1	myosin IIIA [Source:HGNC Symbol;Acc:7601]	6.93	22.70	-	-	-
MYO3A	heat shock transcription factor 2 [Source:HGNC Symbol;Acc:5225]	7.19	90.66	Deafness autosomal recessive 30	-	Autosomal recessive nonsyndromic sensorineural deafness type dfnb
HSF2	hemopoietic cell kinase [Source:HGNC Symbol;Acc:4840]	7.25	13.33	-	-	-
HCK	chromosome 18 open reading frame 25 [Source:HGNC Symbol;Acc:28172]	7.88	52.09	-	-	-
C18orf25	THAP domain containing 4 [Source:HGNC Symbol;Acc:23187]	8.26	69.21	-	-	-
THAP4	phospholipase A1 member A [Source:HGNC Symbol;Acc:17661]	8.88	40.45	-	-	-
PLA1A	chymotrypsin-like elastase family, member 3B [Source:HGNC Symbol;Acc:15945]	8.89	89.24	-	-	-
CELA3B	galactosidase, beta 1-like 3 [Source:HGNC Symbol;Acc:25147]	8.98	96.60	-	-	-
GLB1L3	Wolf-Hirschhorn syndrome candidate 1-like 1 [Source:HGNC Symbol;Acc:12767]	9.20	10.89	-	-	-
WHSC1L1	proline rich 19 [Source:HGNC Symbol;Acc:33728]	9.46	19.93	-	-	-
PRR19	phosphatase and tensin homolog [Source:HGNC Symbol;Acc:9588]	9.78	53.73	-	-	-
PTEN	glutamate receptor, ionotropic, N-methyl D-aspartate 1 [Source:HGNC Symbol;Acc:4584]	9.86	36.86	Bannayan-riley-ruvalcaba syndrome	Bannayan-zonana syndrome (bzs)	Bannayan-riley-ruvalcaba syndrome
GRIN1	gamma-aminobutyric acid (GABA) A receptor, alpha 1 [Source:HGNC Symbol;Acc:4075]	10.72	6.72	Mental retardation autosomal dominant 8	Epileptic encephalopathy	Autosomal dominant nonsyndromic intellectual deficit
GABRA1	NLR family, pyrin domain containing 8 [Source:HGNC Symbol;Acc:22940]	11.32	24.00	Epilepsy juvenile myoclonic susceptibility to 5	Epileptic encephalopathy	Childhood absence epilepsy
NLRP8	notum pectinacetylesterase homolog (Drosophila) [Source:HGNC Symbol;Acc:27106]	11.57	97.25	-	-	-
NOTUM	gamma-aminobutyric acid (GABA) A receptor, beta 3 [Source:HGNC Symbol;Acc:4083]	12.02	23.43	-	-	-
GABRB3	low density lipoprotein receptor class A domain containing 1 [Source:HGNC Symbol;Acc:32069]	12.85	22.36	Epilepsy childhood absence susceptibility to 5	Childhood absence epilepsy type 5	Childhood absence epilepsy
LDLRAD1	dehydrogenase E1 and transketolase domain containing 1 [Source:HGNC Symbol;Acc:23537]	13.46	67.03	-	-	-
DHTKD1	gamma-aminobutyric acid (GABA) A receptor, beta 1 [Source:HGNC Symbol;Acc:4081]	14.18	90.48	-	2-aminoadipic and 2-oxoadipic aciduria	2-aminoadipic aciduria
GABRB1	mesenchyme homeobox 2 [Source:HGNC Symbol;Acc:7014]	14.65	28.93	-	-	-
MEOX2	dynamin 1 [Source:HGNC Symbol;Acc:2972]	14.68	51.66	-	-	-
DNM1	potassium voltage-gated channel, Shab-related subfamily, member 1 [Source:HGNC Symbol;Acc:6231]	14.79	19.54	-	-	-
KCNB1	guanine nucleotide binding protein (G protein), alpha activating activity polypeptide O [Source:HGNC Symbol;Acc:4389]	17.32	50.34	-	-	-
GNAO1	bestrophin 2 [Source:HGNC Symbol;Acc:17107]	17.72	13.94	-	Epileptic encephalopathy	-
BEST2	HBS1-like (S. cerevisiae) [Source:HGNC Symbol;Acc:4834]	18.46	72.60	-	-	-
HBS1L	exosome component 2 [Source:HGNC Symbol;Acc:17097]	18.51	93.25	-	-	-
EXOSC2	TNNI3 interacting kinase [Source:HGNC Symbol;Acc:19661]	19.29	37.32	-	-	-
TNNI3K	nuclear factor, erythroid 2-like 1 [Source:HGNC Symbol;Acc:7781]	21.62	29.59	-	-	-
NFE2L1	D-amino-acid oxidase [Source:HGNC Symbol;Acc:2671]	24.06	3.78	-	-	-
DAO	WD repeat domain 19 [Source:HGNC Symbol;Acc:18340]	24.39	17.16	-	-	Amyotrophic lateral sclerosis
WDR19	muskelin 1, intracellular mediator containing kelch motifs [Source:HGNC Symbol;Acc:7109]	24.74	81.71	Asphyxiating thoracic dystrophy 5	Asphyxiating thoracic dystrophy 5	Cranioectodermal dysplasia
MKLN1	dipeptidyl-peptidase 7 [Source:HGNC Symbol;Acc:14892]	25.98	25.15	-	-	-
DPP7	deoxythymidylate kinase (thymidylate kinase) [Source:HGNC Symbol;Acc:3061]	26.09	69.57	-	-	-
DTYMK	adenosylhomocysteinase [Source:HGNC Symbol;Acc:343]	26.19	20.26	-	-	-
AHCY	NLR family, pyrin domain containing 5 [Source:HGNC Symbol;Acc:21269]	27.61	48.69	Hypermethioninemia with s-adenosylhomocysteine hydrolase deficiency	-	Psychomotor retardation due to s-adenosylhomocysteine hydrolase deficiency
NLRP5	family with sequence similarity 50, member A [Source:HGNC Symbol;Acc:18786]	28.09	66.14	-	-	-
FAM50A	THO complex 2 [Source:HGNC Symbol;Acc:19073]	28.99	53.19	-	-	-
THOC2	exportin 1 (CRM1 homolog, yeast) [Source:HGNC Symbol;Acc:12825]	29.89	18.59	-	-	-
XPO1	1-acylglycerol-3-phosphate O-acyltransferase 3 [Source:HGNC Symbol;Acc:326]	31.01	11.06	-	-	-
AGPAT3	Ran GTPase activating protein 1 [Source:HGNC Symbol;Acc:9854]	32.51	22.36	-	-	-
RANGAP1	cytochrome P450, family 2, subfamily U, polypeptide 1 [Source:HGNC Symbol;Acc:20582]	32.83	28.11	-	-	-
CYP2U1	protein tyrosine phosphatase, receptor type, R [Source:HGNC Symbol;Acc:9680]	34.24	46.92	-	Hereditary spastic paraplegia	Autosomal recessive spastic paraplegia type 56
PTPRR	syntaxin binding protein 1 [Source:HGNC Symbol;Acc:11444]	34.43	81.01	-	Deafness autosomal recessive 84	-
STXBP1	TRAF-interacting protein with forkhead-associated domain [Source:HGNC Symbol;Acc:19075]	35.48	14.97	Epileptic encephalopathy early infantile 4	Nonspecific severe id	Early infantile epileptic encephalopathy
TIFA	GRAM domain containing 2 [Source:HGNC Symbol;Acc:27287]	38.41	51.40	-	-	-
GRAMD2	unc-5 homolog C (C. elegans)-like [Source:HGNC Symbol;Acc:21203]	39.24	74.37	-	-	-
UNC5CL	family with sequence similarity 102, member A [Source:HGNC Symbol;Acc:31419]	40.23	95.47	-	-	-
FAM102A	tyrosine 3-monooxygenase/tryptophan 5-monooxygenase activation protein, gamma [Source:HGNC Symbol;Acc:12852]	45.00	18.59	-	-	-
YWHAG	PDLIM1 interacting kinase 1 like [Source:HGNC Symbol;Acc:18981]	46.59	32.62	-	-	-
PDIK1L	relaxin/insulin-like family peptide receptor 1 [Source:HGNC Symbol;Acc:19718]	47.58	42.88	-	-	-
RXFP1	keratin associated protein 1-3 [Source:HGNC Symbol;Acc:16771]	49.63	32.06	-	-	-
KRTAP1-3	sorting nexin family member 30 [Source:HGNC Symbol;Acc:23685]	50.13	92.68	-	-	-
SNX30	cartilage acidic protein 1 [Source:HGNC Symbol;Acc:14882]	53.71	24.19	-	-	-
CRTAC1	late cornified envelope 1A [Source:HGNC Symbol;Acc:29459]	54.12	13.16	-	-	-
LCE1A	acyl-CoA thioesterase 4 [Source:HGNC Symbol;Acc:19748]	55.32	56.64	-	-	-
ACOT4	CDP-diacylglycerol synthase (phosphatidate cytidylyltransferase) 2 [Source:HGNC Symbol;Acc:1801]	55.75	71.41	-	-	-
CDS2	RuvB-like AAA ATPase 2 [Source:HGNC Symbol;Acc:10475]	57.06	31.69	-	-	-
RUVBL2	C1q and tumor necrosis factor related protein 6 [Source:HGNC Symbol;Acc:14343]	58.26	7.94	-	-	-
C1QTNF6	receptor (chemosensory) transporter protein 1 [Source:HGNC Symbol;Acc:28580]	58.85	91.58	-	-	-
RTP1	protein kinase, X-linked [Source:HGNC Symbol;Acc:9441]	59.69	58.85	-	-	-
PRKX	growth arrest-specific 2 [Source:HGNC Symbol;Acc:4167]	60.99	23.04	-	-	-
GAS2	ALG13, UDP-N-acetylglucosaminyltransferase subunit [Source:HGNC Symbol;Acc:30881]	61.58	66.82	-	-	-
ALG13	rhotekin 2 [Source:HGNC Symbol;Acc:19364]	62.79	8.37	-	Congenital disorder of glycosylation type is	Alg13-cdg syndrome
RTKN2	casein kinase 1, epsilon [Source:HGNC Symbol;Acc:2453]	62.83	48.78	-	-	-
CSNK1E	neuroligin 2 [Source:HGNC Symbol;Acc:14290]	63.39	30.07	-	-	-
NLGN2	arginyl-tRNA synthetase [Source:HGNC Symbol;Acc:9870]	63.63	7.94	-	-	-
RARS	PWWP domain containing 2A [Source:HGNC Symbol;Acc:29406]	63.66	25.64	-	-	-
PWWP2A	aldo-keto reductase family 1, member C4 [Source:HGNC Symbol;Acc:387]	63.82	53.73	-	-	-
AKR1C4	coenzyme Q3 methyltransferase [Source:HGNC Symbol;Acc:18175]	64.23	53.85	46xy sex reversal 8	-	46xy disorder of sex development due to isolated 17 20 lyase deficiency
COQ3	signaling lymphocytic activation molecule family member 1 [Source:HGNC Symbol;Acc:10903]	64.87	83.25	-	-	-
SLAMF1	coiled-coil domain containing 125 [Source:HGNC Symbol;Acc:28924]	65.21	86.17	-	-	-
CCDC125	regulatory factor X, 3 (influences HLA class II expression) [Source:HGNC Symbol;Acc:9984]	65.54	89.14	-	-	-
RFX3	dehydrodolichyl diphosphate synthase [Source:HGNC Symbol;Acc:20603]	65.65	12.46	-	-	-
DHDDS	RIO kinase 3 [Source:HGNC Symbol;Acc:11451]	66.15	46.74	Retinitis pigmentosa 59	-	Retinitis pigmentosa
RIOK3	cyclin-dependent kinase-like 5 [Source:HGNC Symbol;Acc:11411]	67.20	37.32	-	-	-
CDKL5	histone cluster 1, H2bd [Source:HGNC Symbol;Acc:4747]	67.58	15.86	Epileptic encephalopathy early infantile 2	Epileptic encephalopathy early infantile type 2 (eiee2)	Atypical rett syndrome
HIST1H2BD	olfactory receptor, family 10, subfamily S, member 1 [Source:HGNC Symbol;Acc:14807]	68.59	49.76	-	-	-
OR10S1	centrosomal protein 55kDa [Source:HGNC Symbol;Acc:1161]	69.10	89.47	-	-	-
CEP55	glial cells missing homolog 2 (Drosophila) [Source:HGNC Symbol;Acc:4198]	70.46	96.00	-	-	-
GCM2	mitochondrial translational release factor 1 [Source:HGNC Symbol;Acc:7469]	70.64	92.59	Hypoparathyroidism familial isolated	-	Familial isolated hypoparathyroidism due to agenesis of parathyroid gland

MTRF1	G protein-coupled receptor 108 [Source:HGNC Symbol;Acc:17829]	70.89	83.98	-	-	-
GPR108	DEAD (Asp-Glu-Ala-Asp) box polypeptide 50 [Source:HGNC Symbol;Acc:17906]	70.96	70.64	-	-	-
DDX50	ribosomal RNA processing 1B [Source:HGNC Symbol;Acc:23818]	71.56	19.73	-	-	-
RRP1B	CTTNBP2 N-terminal like [Source:HGNC Symbol;Acc:25330]	73.61	29.43	-	-	-
CTTNBP2NL	mitogen-activated protein kinase 8 interacting protein 1 [Source:HGNC Symbol;Acc:6882]	74.88	48.69	-	-	-
MAPK8IP1	scaffold attachment factor B2 [Source:HGNC Symbol;Acc:21605]	75.22	17.31	Diabetes mellitus noninsulin-dependent	-	-
SAFB2	solute carrier family 1 (glial high affinity glutamate transporter), member 2 [Source:HGNC Symbol;Acc:10940]	75.81	9.68	-	-	-
SLC1A2	cut-like homeobox 2 [Source:HGNC Symbol;Acc:19347]	76.43	5.03	-	-	-
CUX2	spindle and kinetochore associated complex subunit 3 [Source:HGNC Symbol;Acc:20262]	76.73	1.60	-	-	-
SKA3	G protein-coupled receptor 128 [Source:HGNC Symbol;Acc:19241]	77.62	88.30	-	-	-
GPR128	zinc finger protein 839 [Source:HGNC Symbol;Acc:20345]	77.83	87.73	-	-	-
ZNF839	tripartite motif containing 29 [Source:HGNC Symbol;Acc:17274]	78.23	95.38	-	-	-
TRIM29	phosphoinositide-3-kinase adaptor protein 1 [Source:HGNC Symbol;Acc:30034]	78.98	48.84	-	-	-
PIK3AP1	histidine-rich glycoprotein [Source:HGNC Symbol;Acc:5181]	80.87	57.48	-	-	-
HRG	ankyrin repeat domain 12 [Source:HGNC Symbol;Acc:29135]	82.38	99.55	Thrombophilia due to histidine-rich glycoprotein deficiency	-	Hereditary thrombophilia due to congenital histidine-rich (poly-l) glycoprotein deficiency
ANKRD12	syndecan binding protein (syntenin) 2 [Source:HGNC Symbol;Acc:15756]	82.71	45.36	-	-	-
SDCBP2	DEAD (Asp-Glu-Ala-Asp) box polypeptide 58 [Source:HGNC Symbol;Acc:19102]	83.23	94.17	-	-	-
DDX58	phosphofurin acidic cluster sorting protein 2 [Source:HGNC Symbol;Acc:23794]	83.32	57.54	-	-	-
PACS2	microtubule associated serine/threonine kinase 1 [Source:HGNC Symbol;Acc:19034]	83.78	14.24	-	-	-
MAST1	Fc fragment of IgG, low affinity IIb, receptor (CD32) [Source:HGNC Symbol;Acc:3618]	83.78	1.94	-	-	-
FCGR2B	KIAA2018 [Source:HGNC Symbol;Acc:30494]	85.95	58.53	Malaria susceptibility to	-	-
KIAA2018	protocadherin beta 13 [Source:HGNC Symbol;Acc:8684]	86.63	14.50	-	-	-
PCDHB13	chromosome 16 open reading frame 62 [Source:HGNC Symbol;Acc:24641]	86.78	67.50	-	-	-
C16orf62	SMAD specific E3 ubiquitin protein ligase 1 [Source:HGNC Symbol;Acc:16807]	87.59	5.69	-	-	-
SMURF1	paraneoplastic Ma antigen family-like 1 [Source:HGNC Symbol;Acc:25578]	87.79	4.39	-	-	-
PNMAL1	zinc finger and SCAN domain containing 2 [Source:HGNC Symbol;Acc:20994]	89.34	55.61	-	-	-
ZSCAN2	myomesin 3 [Source:HGNC Symbol;Acc:26679]	89.78	5.86	-	-	-
MYOM3	mechanistic target of rapamycin (serine/threonine kinase) [Source:HGNC Symbol;Acc:3942]	90.32	92.65	-	-	-
MTOR	zinc finger homeobox 3 [Source:HGNC Symbol;Acc:777]	90.45	0.34	-	Hemimegalencephaly mtor	-
ZFHX3	Ral GEF with PH domain and SH3 binding motif 1 [Source:HGNC Symbol;Acc:16851]	90.47	0.12	-	-	-
RALGPS1	-	90.61	32.94	-	-	-
SGK223	potassium channel, subfamily T, member 1 [Source:HGNC Symbol;Acc:18865]	90.69	54.13	-	-	-
KCNT1	KIAA1324-like [Source:HGNC Symbol;Acc:21945]	90.93	1.62	-	Malignant migrating partial seizures of infancy	Malignant migrating partial seizures of infancy
KIAA1324L	additional sex combs like 1 (Drosophila) [Source:HGNC Symbol;Acc:18318]	91.28	26.98	-	-	-
ASXL1	plexin A1 [Source:HGNC Symbol;Acc:9099]	91.64	65.77	Bohring-opitz syndrome	Bohring-opitz syndrome	Bohring-opitz syndrome
PLXNA1	serine/threonine kinase 36 [Source:HGNC Symbol;Acc:17209]	91.88	0.09	-	-	-
STK36	neural precursor cell expressed, developmentally down-regulated 4-like, E3 ubiquitin protein ligase [Source:HGNC Symbol;Acc:7728]	92.13	92.86	-	-	-
NEDD4L	glutamate receptor, ionotropic, N-methyl D-aspartate 2B [Source:HGNC Symbol;Acc:4586]	92.39	5.79	-	Epileptic encephalopathy	-
GRIN2B	TAF1 RNA polymerase II, TATA box binding protein (TBP)-associated factor, 250kDa [Source:HGNC Symbol;Acc:11535]	92.81	1.07	-	Autism	-
TAF1	chromosome 17 open reading frame 53 [Source:HGNC Symbol;Acc:28460]	93.04	25.73	Dystonia 3 torsion x-linked	-	X-linked dystonia-parkinsonism
C17orf53	complement component (3d/Epstein Barr virus) receptor 2 [Source:HGNC Symbol;Acc:2336]	93.15	67.43	-	-	-
CR2	chitinase, acidic [Source:HGNC Symbol;Acc:17432]	93.98	57.56	Immunodeficiency common variable 7	-	Common variable immunodeficiency due to an intrinsic b cell defect
CHIA	DIP2 disco-interacting protein 2 homolog C (Drosophila) [Source:HGNC Symbol;Acc:29150]	94.43	97.87	-	-	-
DIP2C	cell division cycle 25B [Source:HGNC Symbol;Acc:1726]	94.77	0.29	-	-	-
CDC25B	trio Rho guanine nucleotide exchange factor [Source:HGNC Symbol;Acc:12303]	94.84	6.48	-	-	-
TRIO	homeodomain interacting protein kinase 3 [Source:HGNC Symbol;Acc:4915]	95.48	0.18	-	-	-
HIPK3	dynein, axonemal, heavy chain 7 [Source:HGNC Symbol;Acc:18661]	96.04	14.24	-	-	-
DNAH7	IQ motif and Sec7 domain 1 [Source:HGNC Symbol;Acc:29112]	96.13	99.82	-	-	-
IQSEC1	A kinase (PRKA) anchor protein 6 [Source:HGNC Symbol;Acc:376]	96.61	5.34	-	-	-
AKAP6	transformation/transcription domain-associated protein [Source:HGNC Symbol;Acc:12347]	97.63	71.65	-	-	-
TRRAP	fatty acid synthase [Source:HGNC Symbol;Acc:3594]	97.88	0.04	-	-	-
FASN	contactin 5 [Source:HGNC Symbol;Acc:2175]	98.37	0.38	-	Autosomal recessive mental retardation	-
CNTN5	ALS2 C-terminal like [Source:HGNC Symbol;Acc:20605]	98.55	47.22	-	-	-
ALS2CL	oxysterol binding protein-like 5 [Source:HGNC Symbol;Acc:16392]	98.56	9.81	-	-	-
OSBPL5	palladin, cytoskeletal associated protein [Source:HGNC Symbol;Acc:17068]	98.90	20.96	-	-	-
PALLD	CUB and Sushi multiple domains 2 [Source:HGNC Symbol;Acc:19290]	98.91	63.67	Pancreatic cancer susceptibility to 1	-	-
CSMD2	Alstrom syndrome 1 [Source:HGNC Symbol;Acc:428]	99.07	1.92	-	-	-
ALMS1	ankyrin 3, node of Ranvier (ankyrin G) [Source:HGNC Symbol;Acc:494]	99.07	98.28	Alstrom syndrome	Alstrom syndrome	Alstrom syndrome
ANK3	G protein-coupled receptor 98 [Source:HGNC Symbol;Acc:17416]	99.14	0.34	-	-	Intellectual deficiency - hypotonia - spasticity - sleep disorder
GPR98	nuclear receptor corepressor 2 [Source:HGNC Symbol;Acc:7673]	99.26	99.95	Febrile seizures familial 4	-	Usher syndrome type 2
NCOR2	neurofascin [Source:HGNC Symbol;Acc:29866]	99.31	0.82	-	-	-
NFASC	titin [Source:HGNC Symbol;Acc:12403]	99.52	2.21	-	-	-
TTN	chromosome 3 open reading frame 22 [Source:HGNC Symbol;Acc:28534]	99.99	98.04	Cardiomyopathy dilated 1g	Cause of early-onset myopathy with fatal cardiomyopathy (eomfc)	Autosomal recessive limb-girdle muscular dystrophy type 2j
C3orf22	calcium/calmodulin-dependent protein kinase IV [Source:HGNC Symbol;Acc:1464]	--	36.86	-	-	-
CAMK4	DENN/MADD domain containing 6B [Source:HGNC Symbol;Acc:32690]	--	34.60	-	-	-
DENND6B	FPGT-TNNI3K readthrough [Source:HGNC Symbol;Acc:42952]	--	--	-	-	-
FPGT-TNNI3K	frequently rearranged in advanced T-cell lymphomas 2 [Source:HGNC Symbol;Acc:16048]	--	27.08	-	-	-
FRAT2	kelch-like family member 11 [Source:HGNC Symbol;Acc:19008]	--	--	-	-	-
KLHL11	lysine (K)-specific methyltransferase 2A [Source:HGNC Symbol;Acc:7132]	--	41.91	-	-	-
KMT2A	lysine (K)-specific methyltransferase 2B [Source:HGNC Symbol;Acc:15840]	--	--	Hairy elbows short stature facial dysmorphism and developmental	Wiedemann-steiner syndrome (wss)	Acute leukemia of ambiguous lineage
KMT2B	nuclear receptor subfamily 1, group H, member 2 [Source:HGNC Symbol;Acc:7965]	--	--	-	-	-
NR1H2	PR domain containing 12 [Source:HGNC Symbol;Acc:13997]	--	22.51	-	-	-
PRDM12	solute carrier family 16 (monocarboxylate transporter), member 3 [Source:HGNC Symbol;Acc:10924]	--	--	-	-	-
SLC16A3	sperm-tail PG-rich repeat containing 2 [Source:HGNC Symbol;Acc:28712]	--	16.68	-	-	-
STPG2	TAF1 RNA polymerase II, TATA box binding protein (TBP)-associated factor, 210kDa-like [Source:HGNC Symbol;Acc:18056]	--	--	-	-	-
TAF1L	-	--	17.75	-	-	-
