# Supplementary material for: EvoTol: a protein-sequence based evolutionary intolerance framework for disease-gene prioritization
Source: Nucleic Acids Res. 2014 Dec 29;43(5):e33. doi: 10.1093/nar/gku1322 (PMC4357693; doi:10.1093/nar/gku1322)
Supplement: SUPPLEMENTARY DATA [file supp_gku1322_nar-02497-met-n-2014-File009.zip › Supp/Supplemental Table 4.pdf]

Supplemental Table 4. EvoTol scores for the 184 genes with de novo mutations by Zaidi et al. The table is sorted according to increasing tolerance and for each gene the corresponding RVIS score is reported for comparison. For each gene we also report data on the knwon associated phenotype, which was retreived by Ensembl Biomart (CRCh37.p13). The database sources of phenotypic associations are: [1]Online Mendelian Inheritance in Man (OMIM, http://www.omim.org); [2]Developmental Disorders Gene to Phenotype (DDG2P) and [3]Orphanet (http://www.orpha.net). Grey, genes within the 25th percentile of intolerance.

Gene Symbol	Gene Description	EvoTol %ile	RVIS %ile	OMIM[1]	DDG2P[2]	Orphanet[3]
PTCH1	patched 1 [Source:HGNC Symbol;Acc:9585]	0.08	0.71	Basal cell carcinoma susceptibility to 1	Basal cell nevus syndrome; bcns	Alobar holoprosencephaly
LRP2	low density lipoprotein receptor-related protein 2 [Source:HGNC Symbol;Acc:6694]	0.21	99.42	Donnai-barrow syndrome	Donnai-barrow syndrome	Donnai-barrow syndrome
FBN2	fibrillin 2 [Source:HGNC Symbol;Acc:3604]	0.48	2.02	Arthrogryposis distal type 9	Congenital contractural arachnodactyly	Congenital contractural arachnodactyly
ABCA13	ATP-binding cassette, sub-family A (ABC1), member 13 [Source:HGNC Symbol;Acc:14638]	0.60	99.79	-	-	-
ALPL	alkaline phosphatase, liver/bone/kidney [Source:HGNC Symbol;Acc:438]	1.03	53.98	Hypophosphatasia adult	Hypophosphatasia	-
STAB1	stabilin 1 [Source:HGNC Symbol;Acc:18628]	1.12	0.16	-	-	-
GRM8	glutamate receptor, metabotropic 8 [Source:HGNC Symbol;Acc:4600]	1.31	19.04	-	-	-
ITPR3	inositol 1,4,5-trisphosphate receptor, type 3 [Source:HGNC Symbol;Acc:6182]	1.67	0.17	-	-	-
GANAB	glucosidase, alpha; neutral AB [Source:HGNC Symbol;Acc:4138]	1.71	16.02	-	-	-
FGFR4	fibroblast growth factor receptor 4 [Source:HGNC Symbol;Acc:3691]	1.96	6.07	-	-	-
KCNH5	potassium voltage-gated channel, subfamily H (eag-related), member 5 [Source:HGNC Symbol;Acc:6254]	2.25	3.81	-	-	-
AIPL1	aryl hydrocarbon receptor interacting protein-like 1 [Source:HGNC Symbol;Acc:359]	2.36	96.17	Leber congenital amaurosis 4	Leber congenital amaurosis 4	-
ABCA10	ATP-binding cassette, sub-family A (ABC1), member 10 [Source:HGNC Symbol;Acc:30]	2.64	91.15	-	-	-
ZNFX1	zinc finger, NFX1-type containing 1 [Source:HGNC Symbol;Acc:29271]	2.75	24.22	-	-	-
KCNJ15	potassium inwardly-rectifying channel, subfamily J, member 15 [Source:HGNC Symbol;Acc:6261]	3.37	58.74	-	-	-
KRT1	keratin 1 [Source:HGNC Symbol;Acc:6412]	3.41	85.68	Epidermolytic hyperkeratosis	-	Annular epidermolytic ichthyosis
ABCB6	ATP-binding cassette, sub-family B (MDR/TAP), member 6 [Source:HGNC Symbol;Acc:47]	3.70	28.26	Blood group langereis system	Microphthalmia isolated with coloboma 7	-
PITX2	paired-like homeodomain 2 [Source:HGNC Symbol;Acc:9005]	4.20	30.37	Axenfeld-rieger syndrome type 1	Axenfeld-rieger syndrome type 1 (rieg1)	Axenfeld's anomaly
UMODL1	uromodulin-like 1 [Source:HGNC Symbol;Acc:12560]	4.25	99.65	-	-	-
KIAA0196	KIAA0196 [Source:HGNC Symbol;Acc:28984]	4.43	4.93	Spastic paraplegia 8 autosomal dominant	-	-
IGFN1	immunoglobulin-like and fibronectin type III domain containing 1 [Source:HGNC Symbol;Acc:24607]	5.14	99.88	-	-	-
CRB2	crumbs homolog 2 (Drosophila) [Source:HGNC Symbol;Acc:18688]	5.98	61.28	-	-	-
SUPT5H	suppressor of Ty 5 homolog (S. cerevisiae) [Source:HGNC Symbol;Acc:11469]	7.00	4.10	-	-	-
SBNO2	strawberry notch homolog 2 (Drosophila) [Source:HGNC Symbol;Acc:29158]	7.68	1.25	-	-	-
MPI	mannose phosphate isomerase [Source:HGNC Symbol;Acc:7216]	7.87	42.06	Congenital disorder of glycosylation type ib	Congenital disorders of glycosylation	Mpi-cdg syndrome
C12orf56	chromosome 12 open reading frame 56 [Source:HGNC Symbol;Acc:26967]	7.92	36.07	-	-	-
RDH5	retinol dehydrogenase 5 (11-cis/9-cis) [Source:HGNC Symbol;Acc:9940]	8.49	60.31	Fundus albipunctatus	-	Fundus albipunctatus
RAB11FIP4	RAB11 family interacting protein 4 (class II) [Source:HGNC Symbol;Acc:30267]	9.59	10.80	-	-	-
NR6A1	nuclear receptor subfamily 6, group A, member 1 [Source:HGNC Symbol;Acc:7985]	9.72	27.69	-	-	-
KDM5A	lysine (K)-specific demethylase 5A [Source:HGNC Symbol;Acc:9886]	10.18	13.79	-	Autosomal recessive mental retardation	-
PFKM	phosphofructokinase, muscle [Source:HGNC Symbol;Acc:8877]	10.91	23.57	Glycogen storage disease vii	-	-
C9orf64	chromosome 9 open reading frame 64 [Source:HGNC Symbol;Acc:28144]	11.03	86.17	-	-	-
DMBX1	diencephalon/mesencephalon homeobox 1 [Source:HGNC Symbol;Acc:19026]	11.15	36.07	-	-	-
DTNA	dystrobrevin, alpha [Source:HGNC Symbol;Acc:3057]	11.30	21.65	Left ventricular noncompaction 1	-	-
TM2D2	TM2 domain containing 2 [Source:HGNC Symbol;Acc:24127]	11.77	53.19	-	-	-
UNC13C	unc-13 homolog C (C. elegans) [Source:HGNC Symbol;Acc:23149]	12.10	2.70	-	-	-
FAM76A	family with sequence similarity 76, member A [Source:HGNC Symbol;Acc:28530]	12.25	41.25	-	-	-
DDO	D-aspartate oxidase [Source:HGNC Symbol;Acc:2727]	13.09	98.20	-	-	-
EEPD1	endonuclease/exonuclease/phosphatase family domain containing 1 [Source:HGNC Symbol;Acc:22223]	13.91	32.15	-	-	-
IL2RB	interleukin 2 receptor, beta [Source:HGNC Symbol;Acc:6009]	15.21	77.91	-	-	-
GPRC5B	G protein-coupled receptor, family C, group 5, member B [Source:HGNC Symbol;Acc:13308]	15.56	11.88	-	-	-
SBNO1	strawberry notch homolog 1 (Drosophila) [Source:HGNC Symbol;Acc:22973]	17.17	12.01	-	-	-
SOS1	son of sevenless homolog 1 (Drosophila) [Source:HGNC Symbol;Acc:11187]	17.22	8.54	Fibromatosis gingival 1	Noonan syndrome 4	-
ALS2CR11	amyotrophic lateral sclerosis 2 (juvenile) chromosome region, candidate 11 [Source:HGNC Symbol;Acc:14438]	17.56	98.71	-	-	-
DGCR2	DiGeorge syndrome critical region gene 2 [Source:HGNC Symbol;Acc:2845]	18.21	6.03	-	-	-
TARS2	threonyl-tRNA synthetase 2, mitochondrial (putative) [Source:HGNC Symbol;Acc:30740]	18.42	34.82	-	-	-
LMBRD2	LMBR1 domain containing 2 [Source:HGNC Symbol;Acc:25287]	18.78	26.73	-	-	-
INTS6	integrator complex subunit 6 [Source:HGNC Symbol;Acc:14879]	19.00	10.73	-	-	-
SMAD4	SMAD family member 4 [Source:HGNC Symbol;Acc:6770]	21.74	31.69	Colorectal cancer	Myhre syndrome	-
MYOG	myogenin (myogenic factor 4) [Source:HGNC Symbol;Acc:7612]	22.66	81.38	-	-	-
TDRD12	tudor domain containing 12 [Source:HGNC Symbol;Acc:25044]	22.95	89.91	-	-	-
IL12RB1	interleukin 12 receptor, beta 1 [Source:HGNC Symbol;Acc:5971]	23.81	76.54	Atypical mycobacteriosis familial	-	-
KIAA1468	KIAA1468 [Source:HGNC Symbol;Acc:29289]	24.94	5.08	-	-	-
CHIC1	cysteine-rich hydrophobic domain 1 [Source:HGNC Symbol;Acc:1934]	28.61	58.00	-	-	-
SMAD2	SMAD family member 2 [Source:HGNC Symbol;Acc:6768]	29.99	27.42	-	Congenital heart disease	-
CYP2J2	cytochrome P450, family 2, subfamily J, polypeptide 2 [Source:HGNC Symbol;Acc:2634]	34.10	15.86	-	-	-
CALML6	calmodulin-like 6 [Source:HGNC Symbol;Acc:24193]	34.84	63.20	-	-	-
LPHN3	latrophilin 3 [Source:HGNC Symbol;Acc:20974]	35.35	28.26	-	-	-
ZNF326	zinc finger protein 326 [Source:HGNC Symbol;Acc:14104]	35.55	23.25	-	-	-
COL4A3BP	collagen, type IV, alpha 3 (Goodpasture antigen) binding protein [Source:HGNC Symbol;Acc:2205]	36.01	17.75	-	-	-
WDR5	WD repeat domain 5 [Source:HGNC Symbol;Acc:12757]	36.18	35.42	-	-	-
MYO16	myosin XVI [Source:HGNC Symbol;Acc:29822]	36.99	58.53	-	-	-
MKRN2	makorin ring finger protein 2 [Source:HGNC Symbol;Acc:7113]	37.29	15.62	-	-	-
TOMM40L	translocase of outer mitochondrial membrane 40 homolog (yeast)-like [Source:HGNC Symbol;Acc:25756]	37.53	46.49	-	-	-
ZNF675	zinc finger protein 675 [Source:HGNC Symbol;Acc:30768]	37.58	53.85	-	-	-
DEFB128	defensin, beta 128 [Source:HGNC Symbol;Acc:18106]	38.06	91.76	-	-	-
UBE2B	ubiquitin-conjugating enzyme E2B [Source:HGNC Symbol;Acc:12473]	38.92	54.63	-	-	-
RAB10	RAB10, member RAS oncogene family [Source:HGNC Symbol;Acc:9759]	41.71	54.63	-	-	-
ZNF576	zinc finger protein 576 [Source:HGNC Symbol;Acc:28357]	41.99	71.08	-	-	-
SIGLEC5	sialic acid binding Ig-like lectin 5 [Source:HGNC Symbol;Acc:10874]	43.51	81.60	-	-	-
NEURL2	neuralized E3 ubiquitin protein ligase 2 [Source:HGNC Symbol;Acc:16156]	43.86	67.92	-	-	-
KPNA1	karyopherin alpha 1 (importin alpha 5) [Source:HGNC Symbol;Acc:6394]	44.34	37.11	-	-	-
HEPACAM	hepatic and glial cell adhesion molecule [Source:HGNC Symbol;Acc:26361]	44.97	41.91	Megalencephalic leukoencephalopathy with subcortical cysts 2a	-	-
FABP2	fatty acid binding protein 2, intestinal [Source:HGNC Symbol;Acc:3556]	46.28	56.64	-	-	-
DST	dystonin [Source:HGNC Symbol;Acc:1090]	46.46	20.71	Neuropathy hereditary sensory and autonomic type vi	-	-
NUCB1	nucleobindin 1 [Source:HGNC Symbol;Acc:8043]	48.92	10.43	-	-	-
EIF3H	eukaryotic translation initiation factor 3, subunit H [Source:HGNC Symbol;Acc:3273]	51.00	27.42	-	-	-
EFHD2	EF-hand domain family, member D2 [Source:HGNC Symbol;Acc:28670]	51.01	41.64	-	-	-
DNAJC5B	DnaJ (Hsp40) homolog, subfamily C, member 5 beta [Source:HGNC Symbol;Acc:24138]	51.03	60.09	-	-	-
CYP11A1	cytochrome P450, family 11, subfamily A, polypeptide 1 [Source:HGNC Symbol;Acc:2590]	51.42	55.61	Adrenal insufficiency congenital with 46xy sex reversal partial	-	-
ATM	ataxia telangiectasia mutated [Source:HGNC Symbol;Acc:795]	51.44	95.51	Ataxia-telangiectasia	Ataxia-telangiectasia; at	-
FADS3	fatty acid desaturase 3 [Source:HGNC Symbol;Acc:3576]	53.56	17.75	-	-	-
MAK16	MAK16 homolog (S. cerevisiae) [Source:HGNC Symbol;Acc:13703]	55.30	30.37	-	-	-
DFFB	DNA fragmentation factor, 40kDa, beta polypeptide (caspase-activated DNase) [Source:HGNC Symbol;Acc:2773]	56.23	48.35	-	-	-
ANKS1B	ankyrin repeat and sterile alpha motif domain containing 1B [Source:HGNC Symbol;Acc:24600]	57.31	5.67	-	-	-
PIK3CD	phosphatidylinositol-4,5-bisphosphate 3-kinase, catalytic subunit delta [Source:HGNC Symbol;Acc:8977]	57.32	2.72	-	-	-

RUFY2	RUN and FYVE domain containing 2 [Source:HGNC Symbol;Acc:19761]	58.05	37.32	-	-	-
PDCD1LG2	programmed cell death 1 ligand 2 [Source:HGNC Symbol;Acc:18731]	58.31	75.29	-	-	-
MAP2K7	mitogen-activated protein kinase kinase 7 [Source:HGNC Symbol;Acc:6847]	59.12	21.41	-	-	-
CNTF	ciliary neurotrophic factor [Source:HGNC Symbol;Acc:2169]	59.25	76.96	-	-	-
ZNF34	zinc finger protein 34 [Source:HGNC Symbol;Acc:13098]	59.59	30.70	-	-	-
GPR1	G protein-coupled receptor 1 [Source:HGNC Symbol;Acc:4463]	60.75	82.08	-	-	-
RAVER1	ribonucleoprotein, PTB-binding 1 [Source:HGNC Symbol;Acc:30296]	63.12	37.54	-	-	-
GTPBP1	GTP binding protein 1 [Source:HGNC Symbol;Acc:4669]	63.32	12.88	-	-	-
TWF2	twinfilin actin-binding protein 2 [Source:HGNC Symbol;Acc:9621]	65.18	12.24	-	-	-
ZNF221	zinc finger protein 221 [Source:HGNC Symbol;Acc:13014]	67.05	98.89	-	-	-
MYEF2	myelin expression factor 2 [Source:HGNC Symbol;Acc:17940]	68.54	48.54	-	-	-
SESTD1	SEC14 and spectrin domains 1 [Source:HGNC Symbol;Acc:18379]	68.99	23.43	-	-	-
ZNF544	zinc finger protein 544 [Source:HGNC Symbol;Acc:16759]	69.50	83.57	-	-	-
HAUS3	HAUS augmin-like complex, subunit 3 [Source:HGNC Symbol;Acc:28719]	69.80	52.09	-	-	-
RNF44	ring finger protein 44 [Source:HGNC Symbol;Acc:19180]	71.32	40.36	-	-	-
XRCC5	X-ray repair complementing defective repair in Chinese hamster cells 5 (double-strand-break rejoining) [Source:HGNC Symbol;Acc:12833]	72.23	23.43	-	-	-
PPWD1	peptidylprolyl isomerase domain and WD repeat containing 1 [Source:HGNC Symbol;Acc:28954]	72.85	15.12	-	-	-
PAPSS1	3'-phosphoadenosine 5'-phosphosulfate synthase 1 [Source:HGNC Symbol;Acc:8603]	74.73	12.46	-	-	-
ZNF79	zinc finger protein 79 [Source:HGNC Symbol;Acc:13153]	75.06	63.57	-	-	-
SUV420H1	suppressor of variegation 4-20 homolog 1 (Drosophila) [Source:HGNC Symbol;Acc:24283]	76.04	9.21	-	-	-
AP3B1	adaptor-related protein complex 3, beta 1 subunit [Source:HGNC Symbol;Acc:566]	76.82	77.29	Hermansky-pudlak syndrome 1	-	-
ANGPT4	angiopoietin 4 [Source:HGNC Symbol;Acc:487]	77.03	60.65	-	-	-
ADH1B	alcohol dehydrogenase 1B (class I), beta polypeptide [Source:HGNC Symbol;Acc:250]	77.03	81.55	-	-	-
DHX38	DEAH (Asp-Glu-Ala-His) box polypeptide 38 [Source:HGNC Symbol;Acc:17211]	77.80	6.36	-	-	-
DSG2	desmoglein 2 [Source:HGNC Symbol;Acc:3049]	78.87	98.32	Arrhythmogenic right ventricular dysplasia familial 10	-	-
PES1	pescadillo ribosomal biogenesis factor 1 [Source:HGNC Symbol;Acc:8848]	80.88	44.03	-	-	-
TBC1D4	TBC1 domain family, member 4 [Source:HGNC Symbol;Acc:19165]	82.00	91.12	-	-	-
CDYL2	chromodomain protein, Y-like 2 [Source:HGNC Symbol;Acc:23030]	82.13	5.99	-	-	-
FAM171B	family with sequence similarity 171, member B [Source:HGNC Symbol;Acc:29412]	82.41	7.15	-	-	-
BACH2	BTB and CNC homology 1, basic leucine zipper transcription factor 2 [Source:HGNC Symbol;Acc:14078]	82.98	17.45	-	-	-
MAP3K1	mitogen-activated protein kinase kinase kinase 1, E3 ubiquitin protein ligase [Source:HGNC Symbol;Acc:6848]	83.27	5.52	46xy sex reversal 6	46xy sex reversal 6	-
LZTR1	leucine-zipper-like transcription regulator 1 [Source:HGNC Symbol;Acc:6742]	83.69	0.73	-	-	-
DDX10	DEAD (Asp-Glu-Ala-Asp) box polypeptide 10 [Source:HGNC Symbol;Acc:2735]	84.75	22.78	-	-	-
FAM65C	family with sequence similarity 65, member C [Source:HGNC Symbol;Acc:16168]	84.86	14.30	-	-	-
TMEM63A	transmembrane protein 63A [Source:HGNC Symbol;Acc:29118]	84.88	82.34	-	-	-
CPD	carboxypeptidase D [Source:HGNC Symbol;Acc:2301]	85.01	52.25	-	-	-
ITGA4	integrin, alpha 4 (antigen CD49D, alpha 4 subunit of VLA-4 receptor) [Source:HGNC Symbol;Acc:6140]	85.48	17.40	-	-	-
ELMO2	engulfment and cell motility 2 [Source:HGNC Symbol;Acc:17233]	86.52	11.77	-	-	-
KIAA2018	KIAA2018 [Source:HGNC Symbol;Acc:30494]	86.63	14.50	-	-	-
NOP2	NOP2 nucleolar protein [Source:HGNC Symbol;Acc:7867]	86.86	75.63	-	-	-
MYBPC2	myosin binding protein C, fast type [Source:HGNC Symbol;Acc:7550]	86.96	98.45	-	-	-
PHIP	pleckstrin homology domain interacting protein [Source:HGNC Symbol;Acc:15673]	87.03	5.49	-	Kallmann syndrome	-
NAA16	N(alpha)-acetyltransferase 16, NatA auxiliary subunit [Source:HGNC Symbol;Acc:26164]	88.20	13.05	-	-	-
CCDC129	coiled-coil domain containing 129 [Source:HGNC Symbol;Acc:27363]	89.25	97.04	-	-	-
SSH2	slingshot protein phosphatase 2 [Source:HGNC Symbol;Acc:30580]	89.63	45.68	-	-	-
LOXL2	lysyl oxidase-like 2 [Source:HGNC Symbol;Acc:6666]	89.98	3.11	-	-	-
ZNF536	zinc finger protein 536 [Source:HGNC Symbol;Acc:29025]	90.34	3.20	-	-	-
TRIM41	tripartite motif containing 41 [Source:HGNC Symbol;Acc:19013]	90.50	29.54	-	-	-
C1orf94	chromosome 1 open reading frame 94 [Source:HGNC Symbol;Acc:28250]	90.82	91.87	-	-	-
USP44	ubiquitin specific peptidase 44 [Source:HGNC Symbol;Acc:20064]	90.94	33.47	-	-	-
BCL9	B-cell CLL/lymphoma 9 [Source:HGNC Symbol;Acc:1008]	91.28	2.88	-	-	-
LRPPRC	leucine-rich pentatricopeptide repeat containing [Source:HGNC Symbol;Acc:15714]	91.78	2.77	Leigh syndrome french canadian type	Leigh syndrome french-canadian type	-
LAMB2	laminin, beta 2 (laminin S) [Source:HGNC Symbol;Acc:6487]	91.90	3.76	Nephrotic syndrome type 5 with or without ocular abnormalities;	-	-
CPSF1	cleavage and polyadenylation specific factor 1, 160kDa [Source:HGNC Symbol;Acc:2324]	92.02	0.37	-	-	-
HIVEP2	human immunodeficiency virus type I enhancer binding protein 2 [Source:HGNC Symbol;Acc:4921]	92.61	1.37	-	-	-
ZC3HAV1	zinc finger CCCH-type, antiviral 1 [Source:HGNC Symbol;Acc:23721]	92.90	85.85	-	-	-
USP34	ubiquitin specific peptidase 34 [Source:HGNC Symbol;Acc:20066]	92.91	0.30	-	-	-
TARBP1	TAR (HIV-1) RNA binding protein 1 [Source:HGNC Symbol;Acc:11568]	93.13	36.36	-	-	-
LAMC1	laminin, gamma 1 (formerly LAMB2) [Source:HGNC Symbol;Acc:6492]	93.16	0.94	-	-	-
SPATA2	spermatogenesis associated 2 [Source:HGNC Symbol;Acc:14681]	93.28	10.85	-	-	-
DSC1	desmocollin 1 [Source:HGNC Symbol;Acc:3035]	93.37	86.85	-	-	-
FAM135A	family with sequence similarity 135, member A [Source:HGNC Symbol;Acc:21084]	93.42	21.84	-	-	-
BTN3A3	butyrophilin, subfamily 3, member A3 [Source:HGNC Symbol;Acc:1140]	93.60	33.49	-	-	-
TLN1	talin 1 [Source:HGNC Symbol;Acc:11845]	93.84	0.37	-	-	-
AFTPH	aftiphilin [Source:HGNC Symbol;Acc:25951]	94.07	66.24	-	-	-
FYCO1	FYVE and coiled-coil domain containing 1 [Source:HGNC Symbol;Acc:14673]	94.09	99.24	Cataract autosomal recessive congenital 2	Cataract autosomal recessive congenital 2	-
TSHZ1	teashirt zinc finger homeobox 1 [Source:HGNC Symbol;Acc:10669]	94.38	5.01	Aural atresia congenital	Aural atresia congenital	-
INPP4B	inositol polyphosphate-4-phosphatase, type II, 105kDa [Source:HGNC Symbol;Acc:6075]	94.53	24.60	-	-	-
MAPK8IP3	mitogen-activated protein kinase 8 interacting protein 3 [Source:HGNC Symbol;Acc:6884]	94.59	0.77	-	-	-
MINK1	misshapen-like kinase 1 [Source:HGNC Symbol;Acc:17565]	94.98	3.86	-	-	-
FAM129C	family with sequence similarity 129, member C [Source:HGNC Symbol;Acc:24130]	95.01	95.79	-	-	-
NFATC2	nuclear factor of activated T-cells, cytoplasmic, calcineurin-dependent 2 [Source:HGNC Symbol;Acc:7776]	95.08	3.18	-	-	-
NCAPD3	non-SMC condensin II complex, subunit D3 [Source:HGNC Symbol;Acc:28952]	95.26	28.12	-	-	-
PCDHGA2	protocadherin gamma subfamily A, 2 [Source:HGNC Symbol;Acc:8700]	95.30	70.78	-	-	-
TFIP11	tuftelin interacting protein 11 [Source:HGNC Symbol;Acc:17165]	95.46	3.42	-	-	-
KNDC1	kinase non-catalytic C-lobe domain (KIND) containing 1 [Source:HGNC Symbol;Acc:29374]	95.70	91.86	-	-	-
NRG3	neuregulin 3 [Source:HGNC Symbol;Acc:7999]	96.16	23.57	-	-	-
NCKAP5	NCK-associated protein 5 [Source:HGNC Symbol;Acc:29847]	96.29	84.13	-	-	-
PCDH1	protocadherin 1 [Source:HGNC Symbol;Acc:8655]	96.46	7.32	-	-	-
USH1C	Usher syndrome 1C (autosomal recessive, severe) [Source:HGNC Symbol;Acc:12597]	96.48	25.87	Deafness autosomal recessive 18a	-	-
MASTL	microtubule associated serine/threonine kinase-like [Source:HGNC Symbol;Acc:19042]	96.66	92.33	Thrombocytopenia 2	-	-
KLHDC4	kelch domain containing 4 [Source:HGNC Symbol;Acc:25272]	96.95	91.76	-	-	-
ITGA7	integrin, alpha 7 [Source:HGNC Symbol;Acc:6143]	97.22	65.35	Muscular dystrophy congenital due to integrin alpha-7 deficiency	Congenital muscular dystrophy	-
NUP62	nucleoporin 62kDa [Source:HGNC Symbol;Acc:8066]	98.02	20.86	Striatonigral degeneration infantile	Infantile striatonigral degeneration (sndi)	-
ALS2CL	ALS2 C-terminal like [Source:HGNC Symbol;Acc:20605]	98.56	9.81	-	-	-
LYST	lysosomal trafficking regulator [Source:HGNC Symbol;Acc:1968]	98.87	0.50	Chediak-higashi syndrome	Chediak-higashi syndrome	-
BICD1	bicaudal D homolog 1 (Drosophila) [Source:HGNC Symbol;Acc:1049]	98.92	3.54	-	-	-
SDK1	sidekick cell adhesion molecule 1 [Source:HGNC Symbol;Acc:19307]	99.01	0.08	-	-	-
FREM2	FRAS1 related extracellular matrix protein 2 [Source:HGNC Symbol;Acc:25396]	99.21	16.77	Fraser syndrome	Fraser syndrome (frass)	-
DCHS2	dachsous cadherin-related 2 [Source:HGNC Symbol;Acc:23111]	99.39	99.94	-	-	-
DNAH9	dynein, axonemal, heavy chain 9 [Source:HGNC Symbol;Acc:2953]	99.59	78.06	-	-	-
LAMA5	laminin, alpha 5 [Source:HGNC Symbol;Acc:6485]	99.62	99.28	-	-	-
VPS13C	vacuolar protein sorting 13 homolog C (S. cerevisiae) [Source:HGNC Symbol;Acc:23594]	99.90	12.08	-	-	-
TTN	titin [Source:HGNC Symbol;Acc:12403]	99.99	98.04	Cardiomyopathy dilated 1g	Cause of early-onset myopathy with fatal cardiomyopathy (eomfc)	-
MYRF	myelin regulatory factor [Source:HGNC Symbol;Acc:1181]	--	--	-	-	-
CLUH	clustered mitochondria (cluA/CLU1) homolog [Source:HGNC Symbol;Acc:29094]	--	--	-	-	-
COLGALT1	collagen beta(1-O)galactosyltransferase 1 [Source:HGNC Symbol;Acc:26182]	--	--	-	-	-
CIPC	CLOCK-interacting pacemaker [Source:HGNC Symbol;Acc:20365]	--	--	-	-	-
