# Supplementary material for: EvoTol: a protein-sequence based evolutionary intolerance framework for disease-gene prioritization
Source: Nucleic Acids Res. 2014 Dec 29;43(5):e33. doi: 10.1093/nar/gku1322 (PMC4357693; doi:10.1093/nar/gku1322)
Supplement: SUPPLEMENTARY DATA [file supp_gku1322_nar-02497-met-n-2014-File009.zip › Supp/Supplemental Table 5.pdf]

Supplemental Table 5. Proteins (and corresponding genes) associated with congenital heart disease retrived from UniProt (search term “congenital heart disease").

Entry	Entry name	Status	Protein names	Associated gene names	Organism	Length
Q15327	ANKR1_HUMAN	reviewed	Ankyrin repeat domain-containing protein 1 (Cardiac ankyrin repeat protein) (Cytokine-inducible gene C-193 protein) (Cytokine-inducible nuclear protein)	ANKRD1 C193 CARP HA1A2	Homo sapiens (Human)	319
Q9H0F7	ARL6_HUMAN	reviewed	ADP-ribosylation factor-like protein 6 (Bardet-Biedl syndrome 3 protein)	ARL6 BBS3	Homo sapiens (Human)	186
Q96L58	B3GT6_HUMAN	reviewed	Beta-1	B3GALT6	Homo sapiens (Human)	329
Q8TAM1	BBS10_HUMAN	reviewed	Bardet-Biedl syndrome 10 protein	BBS10 C12orf58	Homo sapiens (Human)	723
Q6ZW61	BBS12_HUMAN	reviewed	Bardet-Biedl syndrome 12 protein	BBS12 C4orf24	Homo sapiens (Human)	710
Q8NFJ9	BBS1_HUMAN	reviewed	Bardet-Biedl syndrome 1 protein (BBS2-like protein 2)	BBS1 BBS2L2	Homo sapiens (Human)	593
Q9BXC9	BBS2_HUMAN	reviewed	Bardet-Biedl syndrome 2 protein	BBS2	Homo sapiens (Human)	721
Q96RK4	BBS4_HUMAN	reviewed	Bardet-Biedl syndrome 4 protein	BBS4	Homo sapiens (Human)	519
Q8N3I7	BBS5_HUMAN	reviewed	Bardet-Biedl syndrome 5 protein	BBS5	Homo sapiens (Human)	341
Q8IWZ6	BBS7_HUMAN	reviewed	Bardet-Biedl syndrome 7 protein (BBS2-like protein 1)	BBS7 BBS2L1	Homo sapiens (Human)	715
Q13936	CAC1C_HUMAN	reviewed	Voltage-dependent L-type calcium channel subunit alpha-1C	CACNA1C CACH2 CACN2 CACNL1A1 CCHL1A1	Homo sapiens (Human)	2221
Q9BUN5	CC28B_HUMAN	reviewed	Coiled-coil domain-containing protein 28B	CCDC28B	Homo sapiens (Human)	200
O15078	CE290_HUMAN	reviewed	Centrosomal protein of 290 kDa (Cep290) (Bardet-Biedl syndrome 14 protein) (Cancer/testis antigen 87) (CT87) (Nephrocystin-6) (Tumor antigen se2-2)	CEP290 BBS14 KIAA0373 NPHP6	Homo sapiens (Human)	2479
P31327	CPSM_HUMAN	reviewed	Carbamoyl-phosphate synthase [ammonia]	CPS1	Homo sapiens (Human)	1500
P15924	DESP_HUMAN	reviewed	Desmoplakin (DP) (250/210 kDa paraneoplastic pemphigus antigen)	DSP	Homo sapiens (Human)	2871
Q02487	DSC2_HUMAN	reviewed	Desmocollin-2 (Cadherin family member 2) (Desmocollin-3) (Desmosomal glycoprotein II) (Desmosomal glycoprotein III)	DSC2 CDHF2 DSC3	Homo sapiens (Human)	901
Q14126	DSG2_HUMAN	reviewed	Desmoglein-2 (Cadherin family member 5) (HDGC)	DSG2 CDHF5	Homo sapiens (Human)	1118
O95876	FRITZ_HUMAN	reviewed	WD repeat-containing and planar cell polarity effector protein fritz homolog (hFRTZ)	WDPCP BBS15 C2orf86 FRITZ	Homo sapiens (Human)	746
Q92908	GATA6_HUMAN	reviewed	Transcription factor GATA-6 (GATA-binding factor 6)	GATA6	Homo sapiens (Human)	595
P43694	GATA4_HUMAN	reviewed	Transcription factor GATA-4 (GATA-binding factor 4)	GATA4	Homo sapiens (Human)	442
Q8NCW6	GLT11_HUMAN	reviewed	Polypeptide N-acetylgalactosaminyltransferase 11 (EC 2.4.1.41)	GALNT11	Homo sapiens (Human)	608
P54198	HIRA_HUMAN	reviewed	Protein HIRA (TUP1-like enhancer of split protein 1)	HIRA DGCR1 HIR TUPLE1	Homo sapiens (Human)	1017
Q2M1P5	KIF7_HUMAN	reviewed	Kinesin-like protein KIF7	KIF7 UNQ340/PRO539	Homo sapiens (Human)	1343
Q9NQ48	LZTL1_HUMAN	reviewed	Leucine zipper transcription factor-like protein 1	LZTFL1	Homo sapiens (Human)	299
Q7Z7M0	MEGF8_HUMAN	reviewed	Multiple epidermal growth factor-like domains protein 8 (Multiple EGF-like domains protein 8) (Epidermal growth factor-like protein 4) (EGF-like protein 4)	MEGF8 C19orf49 EGFL4 KIAA0817	Homo sapiens (Human)	2845
Q9NPJ1	MKKS_HUMAN	reviewed	McKusick-Kaufman/Bardet-Biedl syndromes putative chaperonin (Bardet-Biedl syndrome 6 protein)	MKKS BBS6	Homo sapiens (Human)	570
Q5HYA8	MKS3_HUMAN	reviewed	Meckelin (Meckel syndrome type 3 protein) (Transmembrane protein 67)	TMEM67 MKS3	Homo sapiens (Human)	995
Q9NXB0	MKS1_HUMAN	reviewed	Meckel syndrome type 1 protein	MKS1	Homo sapiens (Human)	559
P52952	NKX25_HUMAN	reviewed	Homeobox protein Nkx-2.5 (Cardiac-specific homeobox) (Homeobox protein CSX) (Homeobox protein NK-2 homolog E)	NKX2-5 CSX NKX2.5 NKX2E	Homo sapiens (Human)	324
Q5SRE5	NU188_HUMAN	reviewed	Nucleoporin NUP188 homolog (hNup188)	NUP188 KIAA0169	Homo sapiens (Human)	1749
P15428	PGDH_HUMAN	reviewed	15-hydroxyprostaglandin dehydrogenase [NAD(+)] (15-PGDH) (EC 1.1.1.141) (Prostaglandin dehydrogenase 1)	HPGD PGDH1	Homo sapiens (Human)	266
Q9Y2B2	PIGL_HUMAN	reviewed	N-acetylglucosaminyl-phosphatidylinositol de-N-acetylase (EC 3.5.1.89) (Phosphatidylinositol-glycan biosynthesis class L protein) (PIG-L)	PIGL	Homo sapiens (Human)	252
Q99959	PKP2_HUMAN	reviewed	Plakophilin-2	PKP2	Homo sapiens (Human)	881
P14923	PLAK_HUMAN	reviewed	Junction plakoglobin (Catenin gamma) (Desmoplakin III) (Desmoplakin-3)	JUP CTNNG DP3	Homo sapiens (Human)	745
Q3SYG4	PTHB1_HUMAN	reviewed	Protein PTHB1 (Bardet-Biedl syndrome 9 protein) (Parathyroid hormone-responsive B1 gene protein)	BBS9 PTHB1	Homo sapiens (Human)	887
Q92736	RYR2_HUMAN	reviewed	Ryanodine receptor 2 (RYR-2) (RyR2) (hRYR-2) (Cardiac muscle ryanodine receptor) (Cardiac muscle ryanodine receptor-calcium release channel) (Type 2 ryanodine receptor)	RYR2	Homo sapiens (Human)	4967
Q13485	SMAD4_HUMAN	reviewed	Mothers against decapentaplegic homolog 4 (MAD homolog 4)	SMAD4 DPC4 MADH4	Homo sapiens (Human)	552
Q92959	SO2A1_HUMAN	reviewed	Solute carrier organic anion transporter family member 2A1 (Prostaglandin transporter) (PGT) (Solute carrier family 21 member 2)	SLCO2A1 OATP2A1 SLC21A2	Homo sapiens (Human)	643
Q9NYJ8	TAB2_HUMAN	reviewed	TGF-beta-activated kinase 1 and MAP3K7-binding protein 2	TAB2 KIAA0733 MAP3K7IP2	Homo sapiens (Human)	693
P10600	TGFB3_HUMAN	reviewed	Transforming growth factor beta-3 (TGF-beta-3) [Cleaved into: Latency-associated peptide (LAP)]	TGFB3	Homo sapiens (Human)	412
P37173	TGFR2_HUMAN	reviewed	TGF-beta receptor type-2 (TGFR-2) (EC 2.7.11.30) (TGF-beta type II receptor) (Transforming growth factor-beta receptor type II) (TGF-beta receptor type II) (TbetaR-II)	TGFBR2	Homo sapiens (Human)	567
P36897	TGFR1_HUMAN	reviewed	TGF-beta receptor type-1 (TGFR-1) (EC 2.7.11.30)	TGFBR1 ALK5 SKR4	Homo sapiens (Human)	503
Q9BTV4	TMM43_HUMAN	reviewed	Transmembrane protein 43 (Protein LUMA)	TMEM43 UNQ2564/PRO6244	Homo sapiens (Human)	400
Q13049	TRI32_HUMAN	reviewed	E3 ubiquitin-protein ligase TRIM32 (EC 6.3.2.-) (72 kDa Tat-interacting protein) (Tripartite motif-containing protein 32) (Zinc finger protein HT2A)	TRIM32 HT2A	Homo sapiens (Human)	653
Q7Z4L5	TT21B_HUMAN	reviewed	Tetratricopeptide repeat protein 21B (TPR repeat protein 21B)	TTC21B KIAA1992 Nbla10696	Homo sapiens (Human)	1316
Q8TAM2	TTC8_HUMAN	reviewed	Tetratricopeptide repeat protein 8 (TPR repeat protein 8) (Bardet-Biedl syndrome 8 protein)	TTC8 BBS8	Homo sapiens (Human)	541
O00258	WRB_HUMAN	reviewed	Tail-anchored protein insertion receptor WRB (Congenital heart disease 5 protein) (Tryptophan-rich basic protein) (WRB)	WRB CHD5	Homo sapiens (Human)	174
O60481	ZIC3_HUMAN	reviewed	Zinc finger protein ZIC 3 (Zinc finger protein 203) (Zinc finger protein of the cerebellum 3)	ZIC3 ZNF203	Homo sapiens (Human)	467
