# Supplementary material for: EvoTol: a protein-sequence based evolutionary intolerance framework for disease-gene prioritization
Source: Nucleic Acids Res. 2014 Dec 29;43(5):e33. doi: 10.1093/nar/gku1322 (PMC4357693; doi:10.1093/nar/gku1322)
Supplement: SUPPLEMENTARY DATA [file supp_gku1322_nar-02497-met-n-2014-File009.zip › Supp/Supplemental Table 6.pdf]

Supplemental Table 6. Details on the sub-networks (clusters) predicted to be tolerant (cluster 1, cluster 2) ir intolerant (cluster 3, cluster 4) by EvoTol. For each gene in the cluster we report the EvoTol percentile score (EvoTol %ile) and for each cluster we report the Mann-Whitney U test *P*-value for torelance or intolerance and results of the functional enrichment analysis by DAVID (see Methods). Intolerant genes (EvoTol percentile score <25) are highlighted. FDR, false discovery rate.

Cluster 1 - tolerant	Gene Symbol	Gene description	EvoTol %ile
Mann-Whitney U test $P = 4.6 \times 10^{-22}$	*OR5AN1*	olfactory receptor, family 5, subfamily AN, member 1 [Source:HGNC Symbol;Acc:15255]	66.76
	OR51F2	olfactory receptor, family 51, subfamily F, member 2 [Source:HGNC Symbol;Acc:15197]	70.80
	OR8A1	olfactory receptor, family 8, subfamily A, member 1 [Source:HGNC Symbol;Acc:8469]	81.86
	OR8D1	olfactory receptor, family 8, subfamily D, member 1 [Source:HGNC Symbol;Acc:8481]	77.73
	OR51L1	olfactory receptor, family 51, subfamily L, member 1 [Source:HGNC Symbol;Acc:14759]	78.41
	OR2A5	olfactory receptor, family 2, subfamily A, member 5 [Source:HGNC Symbol;Acc:8232]	74.29
	OR2A12	olfactory receptor, family 2, subfamily A, member 12 [Source:HGNC Symbol;Acc:15082]	57.00
	OR6C68	olfactory receptor, family 6, subfamily C, member 68 [Source:HGNC Symbol;Acc:31297]	77.74
	OR2A25	olfactory receptor, family 2, subfamily A, member 25 [Source:HGNC Symbol;Acc:19562]	70.81
	GNGT1	guanine nucleotide binding protein (G protein), gamma transducing activity polypeptide 1 [Source:HGNC Symbol;Acc:4411]	53.49
	OR6C4	olfactory receptor, family 6, subfamily C, member 4 [Source:HGNC Symbol;Acc:19632]	77.75
	OR1K1	olfactory receptor, family 1, subfamily K, member 1 [Source:HGNC Symbol;Acc:8212]	51.88
	OR51G1	olfactory receptor, family 51, subfamily G, member 1 [Source:HGNC Symbol;Acc:14738]	78.42
	OR4F15	olfactory receptor, family 4, subfamily F, member 15 [Source:HGNC Symbol;Acc:15078]	70.81
	OR7G1	olfactory receptor, family 7, subfamily G, member 1 [Source:HGNC Symbol;Acc:8465]	66.75
	OR10J3	olfactory receptor, family 10, subfamily J, member 3 [Source:HGNC Symbol;Acc:14992]	75.04
	OR4N2	olfactory receptor, family 4, subfamily N, member 2 [Source:HGNC Symbol;Acc:14742]	88.03
	OR2B11	olfactory receptor, family 2, subfamily B, member 11 [Source:HGNC Symbol;Acc:31249]	85.12
	OR8K5	olfactory receptor, family 8, subfamily K, member 5 [Source:HGNC Symbol;Acc:15315]	80.26
	OR5B21	olfactory receptor, family 5, subfamily B, member 21 [Source:HGNC Symbol;Acc:19616]	71.47
	OR6C74	olfactory receptor, family 6, subfamily C, member 74 [Source:HGNC Symbol;Acc:31303]	68.46
	OR3A1	olfactory receptor, family 3, subfamily A, member 1 [Source:HGNC Symbol;Acc:8282]	84.70
	OR8K1	olfactory receptor, family 8, subfamily K, member 1 [Source:HGNC Symbol;Acc:14831]	75.71
	OR52N5	olfactory receptor, family 52, subfamily N, member 5 [Source:HGNC Symbol;Acc:15231]	79.62
	OR10K1	olfactory receptor, family 10, subfamily K, member 1 [Source:HGNC Symbol;Acc:14693]	83.34
	OR11G2	olfactory receptor, family 11, subfamily G, member 2 [Source:HGNC Symbol;Acc:15346]	74.30
	OR2W3	olfactory receptor, family 2, subfamily W, member 3 [Source:HGNC Symbol;Acc:15021]	84.71
	OR4D9	olfactory receptor, family 4, subfamily D, member 9 [Source:HGNC Symbol;Acc:15178]	82.42
	OR4C13	olfactory receptor, family 4, subfamily C, member 13 [Source:HGNC Symbol;Acc:15169]	86.22
	OR5AK2	olfactory receptor, family 5, subfamily AK, member 2 [Source:HGNC Symbol;Acc:15251]	79.01
	OR6C6	olfactory receptor, family 6, subfamily C, member 6 [Source:HGNC Symbol;Acc:31293]	79.00
	OR5D13	olfactory receptor, family 5, subfamily D, member 13 [Source:HGNC Symbol;Acc:15280]	70.79
	OR5K3	olfactory receptor, family 5, subfamily K, member 3 [Source:HGNC Symbol;Acc:31290]	75.74
	OR51V1	olfactory receptor, family 51, subfamily V, member 1 [Source:HGNC Symbol;Acc:19597]	50.17
	OR4L1	olfactory receptor, family 4, subfamily L, member 1 [Source:HGNC Symbol;Acc:15356]	80.73
	OR52E4	olfactory receptor, family 52, subfamily E, member 4 [Source:HGNC Symbol;Acc:15213]	80.72
	OR2G6	olfactory receptor, family 2, subfamily G, member 6 [Source:HGNC Symbol;Acc:27019]	85.12
	OR10A6	olfactory receptor, family 10, subfamily A, member 6 [Source:HGNC Symbol;Acc:15132]	81.38
	OR2S2	olfactory receptor, family 2, subfamily S, member 2 [Source:HGNC Symbol;Acc:8276]	83.33
	OR6C76	olfactory receptor, family 6, subfamily C, member 76 [Source:HGNC Symbol;Acc:31305]	71.46
	OR4N4	olfactory receptor, family 4, subfamily N, member 4 [Source:HGNC Symbol;Acc:15375]	90.35
	OR1C1	olfactory receptor, family 1, subfamily C, member 1 [Source:HGNC Symbol;Acc:8182]	66.77
	OR2T1	olfactory receptor, family 2, subfamily T, member 1 [Source:HGNC Symbol;Acc:8277]	85.50
	OR5D16	olfactory receptor, family 5, subfamily D, member 16 [Source:HGNC Symbol;Acc:15283]	72.20
	OR51A7	olfactory receptor, family 51, subfamily A, member 7 [Source:HGNC Symbol;Acc:15188]	47.39
	OR52N1	olfactory receptor, family 52, subfamily N, member 1 [Source:HGNC Symbol;Acc:14853]	73.70
	OR5T2	olfactory receptor, family 5, subfamily T, member 2 [Source:HGNC Symbol;Acc:15296]	82.40
	OR1Q1	olfactory receptor, family 1, subfamily Q, member 1 [Source:HGNC Symbol;Acc:8223]	74.29
	OR52M1	olfactory receptor, family 52, subfamily M, member 1 [Source:HGNC Symbol;Acc:15225]	88.56
	OR51T1	olfactory receptor, family 51, subfamily T, member 1 [Source:HGNC Symbol;Acc:15205]	47.38
	OR5M9	olfactory receptor, family 5, subfamily M, member 9 [Source:HGNC Symbol;Acc:15294]	85.85
	OR4C16	olfactory receptor, family 4, subfamily C, member 16 (gene/pseudogene) [Source:HGNC Symbol;Acc:15172]	90.61
	OR10W1	olfactory receptor, family 10, subfamily W, member 1 [Source:HGNC Symbol;Acc:15139]	70.82
	OR52B4	olfactory receptor, family 52, subfamily B, member 4 [Source:HGNC Symbol;Acc:15209]	63.42
	OR5P2	olfactory receptor, family 5, subfamily P, member 2 [Source:HGNC Symbol;Acc:14783]	79.60
	OR6A2	olfactory receptor, family 6, subfamily A, member 2 [Source:HGNC Symbol;Acc:15301]	83.82
	OR6K2	olfactory receptor, family 6, subfamily K, member 2 [Source:HGNC Symbol;Acc:15029]	84.23
	OR6C3	olfactory receptor, family 6, subfamily C, member 3 [Source:HGNC Symbol;Acc:15437]	80.70
	OR5F1	olfactory receptor, family 5, subfamily F, member 1 [Source:HGNC Symbol;Acc:8343]	80.27
	OR10T2	olfactory receptor, family 10, subfamily T, member 2 [Source:HGNC Symbol;Acc:14816]	84.73
	OR7C2	olfactory receptor, family 7, subfamily C, member 2 [Source:HGNC Symbol;Acc:8374]	77.74
	OR2T6	olfactory receptor, family 2, subfamily T, member 6 [Source:HGNC Symbol;Acc:15018]	84.25
	OR2D3	olfactory receptor, family 2, subfamily D, member 3 [Source:HGNC Symbol;Acc:15146]	77.06
	OR52E2	olfactory receptor, family 52, subfamily E, member 2 [Source:HGNC Symbol;Acc:14769]	83.32
	OR51E1	olfactory receptor, family 51, subfamily E, member 1 [Source:HGNC Symbol;Acc:15194]	47.96
	OR4D11	olfactory receptor, family 4, subfamily D, member 11 [Source:HGNC Symbol;Acc:15174]	74.28
	OR4N5	olfactory receptor, family 4, subfamily N, member 5 [Source:HGNC Symbol;Acc:15358]	85.11
	OR2G3	olfactory receptor, family 2, subfamily G, member 3 [Source:HGNC Symbol;Acc:15008]	77.78
	OR52H1	olfactory receptor, family 52, subfamily H, member 1 [Source:HGNC Symbol;Acc:15218]	79.02
	OR9A4	olfactory receptor, family 9, subfamily A, member 4 [Source:HGNC Symbol;Acc:15095]	65.94
	OR52J3	olfactory receptor, family 52, subfamily J, member 3 [Source:HGNC Symbol;Acc:14799]	84.69
	OR7D2	olfactory receptor, family 7, subfamily D, member 2 [Source:HGNC Symbol;Acc:8378]	75.72
	OR5H2	olfactory receptor, family 5, subfamily H, member 2 [Source:HGNC Symbol;Acc:14752]	75.00
	OR2Z1	olfactory receptor, family 2, subfamily Z, member 1 [Source:HGNC Symbol;Acc:15391]	82.43
	GNAL	guanine nucleotide binding protein (G protein), alpha activating activity polypeptide, olfactory type [Source:HGNC Symbol;Acc:4388]	**17.77**
	OR51F1	olfactory receptor, family 51, subfamily F, member 1 [Source:HGNC Symbol;Acc:15196]	85.86
	OR6N2	olfactory receptor, family 6, subfamily N, member 2 [Source:HGNC Symbol;Acc:15035]	73.03
	OR6X1	olfactory receptor, family 6, subfamily X, member 1 [Source:HGNC Symbol;Acc:14737]	75.72
	OR51S1	olfactory receptor, family 51, subfamily S, member 1 [Source:HGNC Symbol;Acc:15204]	40.96
	OR51G2	olfactory receptor, family 51, subfamily G, member 2 [Source:HGNC Symbol;Acc:15198]	81.34
	OR4Q3	olfactory receptor, family 4, subfamily Q, member 3 [Source:HGNC Symbol;Acc:15426]	85.49
	OR9G1	olfactory receptor, family 9, subfamily G, member 1 [Source:HGNC Symbol;Acc:15319]	91.15
	OR56B4	olfactory receptor, family 56, subfamily B, member 4 [Source:HGNC Symbol;Acc:15248]	69.34
	OR2AG2	olfactory receptor, family 2, subfamily AG, member 2 [Source:HGNC Symbol;Acc:15143]	83.33
	OR2K2	olfactory receptor, family 2, subfamily K, member 2 [Source:HGNC Symbol;Acc:8264]	82.44
	OR8D4	olfactory receptor, family 8, subfamily D, member 4 [Source:HGNC Symbol;Acc:14840]	81.31
	OR9G9	olfactory receptor, family 9, subfamily G, member 9 [Source:HGNC Symbol;Acc:31940]	-
	OR6N1	olfactory receptor, family 6, subfamily N, member 1 [Source:HGNC Symbol;Acc:15034]	48.61
	OR4C15	olfactory receptor, family 4, subfamily C, member 15 [Source:HGNC Symbol;Acc:15171]	88.96
	OR51I2	olfactory receptor, family 51, subfamily I, member 2 [Source:HGNC Symbol;Acc:15201]	80.28
	OR4C3	olfactory receptor, family 4, subfamily C, member 3 [Source:HGNC Symbol;Acc:14697]	93.96
	OR10J1	olfactory receptor, family 10, subfamily J, member 1 [Source:HGNC Symbol;Acc:8175]	81.37
	OR5A2	olfactory receptor, family 5, subfamily A, member 2 [Source:HGNC Symbol;Acc:15249]	76.34
	OR4C12	olfactory receptor, family 4, subfamily C, member 12 [Source:HGNC Symbol;Acc:15168]	69.35
	OR5B2	olfactory receptor, family 5, subfamily B, member 2 [Source:HGNC Symbol;Acc:8323]	80.71
	OR4K13	olfactory receptor, family 4, subfamily K, member 13 [Source:HGNC Symbol;Acc:15351]	77.06
	OR10R2	olfactory receptor, family 10, subfamily R, member 2 [Source:HGNC Symbol;Acc:14820]	79.04
	OR1J2	olfactory receptor, family 1, subfamily J, member 2 [Source:HGNC Symbol;Acc:8209]	85.88
	OR1J4	olfactory receptor, family 1, subfamily J, member 4 [Source:HGNC Symbol;Acc:8211]	73.71
	OR6C1	olfactory receptor, family 6, subfamily C, member 1 [Source:HGNC Symbol;Acc:8355]	78.39
	OR7G2	olfactory receptor, family 7, subfamily G, member 2 [Source:HGNC Symbol;Acc:8466]	74.26
	OR14C36	olfactory receptor, family 14, subfamily C, member 36 [Source:HGNC Symbol;Acc:15026]	70.05
	OR5I1	olfactory receptor, family 5, subfamily I, member 1 [Source:HGNC Symbol;Acc:8347]	78.40
	OR5H14	olfactory receptor, family 5, subfamily H, member 14 [Source:HGNC Symbol;Acc:31286]	88.56
	OR10A7	olfactory receptor, family 10, subfamily A, member 7 [Source:HGNC Symbol;Acc:15329]	78.44
	OR9Q2	olfactory receptor, family 9, subfamily Q, member 2 [Source:HGNC Symbol;Acc:15328]	76.32
	OR5B17	olfactory receptor, family 5, subfamily B, member 17 [Source:HGNC Symbol;Acc:15267]	77.76
	OR51I1	olfactory receptor, family 51, subfamily I, member 1 [Source:HGNC Symbol;Acc:15200]	84.23
	OR5AS1	olfactory receptor, family 5, subfamily AS, member 1 [Source:HGNC Symbol;Acc:15261]	83.83
	OR4K15	olfactory receptor, family 4, subfamily K, member 15 [Source:HGNC Symbol;Acc:15353]	84.70
	OR2G2	olfactory receptor, family 2, subfamily G, member 2 [Source:HGNC Symbol;Acc:15007]	73.05
	OR1N1	olfactory receptor, family 1, subfamily N, member 1 [Source:HGNC Symbol;Acc:8221]	77.07
	OR7E24	olfactory receptor, family 7, subfamily E, member 24 [Source:HGNC Symbol;Acc:8396]	65.94
	OR4X1	olfactory receptor, family 4, subfamily X, member 1 (gene/pseudogene) [Source:HGNC Symbol;Acc:14854]	85.86
	OR1L3	olfactory receptor, family 1, subfamily L, member 3 [Source:HGNC Symbol;Acc:8215]	75.02
	OR10A4	olfactory receptor, family 10, subfamily A, member 4 [Source:HGNC Symbol;Acc:15130]	80.30
	OR2T27	olfactory receptor, family 2, subfamily T, member 27 [Source:HGNC Symbol;Acc:31252]	87.39
	OR56B1	olfactory receptor, family 56, subfamily B, member 1 [Source:HGNC Symbol;Acc:15245]	80.28
	OR1I1	olfactory receptor, family 1, subfamily I, member 1 [Source:HGNC Symbol;Acc:8207]	81.36
	OR51D1	olfactory receptor, family 51, subfamily D, member 1 [Source:HGNC Symbol;Acc:15193]	77.05
	OR10V1	olfactory receptor, family 10, subfamily V, member 1 [Source:HGNC Symbol;Acc:15136]	79.64
	OR6C65	olfactory receptor, family 6, subfamily C, member 65 [Source:HGNC Symbol;Acc:31295]	69.33
	OR10AD1	olfactory receptor, family 10, subfamily AD, member 1 [Source:HGNC Symbol;Acc:14819]	73.72
	OR6C70	olfactory receptor, family 6, subfamily C, member 70 [Source:HGNC Symbol;Acc:31299]	74.26
	OR51B2	olfactory receptor, family 51, subfamily B, member 2 [Source:HGNC Symbol;Acc:14703]	75.01
	OR4C6	olfactory receptor, family 4, subfamily C, member 6 [Source:HGNC Symbol;Acc:14743]	81.88
	OR5AR1	olfactory receptor, family 5, subfamily AR, member 1 (gene/pseudogene) [Source:HGNC Symbol;Acc:15260]	76.33
	OR6K6	olfactory receptor, family 6, subfamily K, member 6 [Source:HGNC Symbol;Acc:15033]	81.33
	OR5T3	olfactory receptor, family 5, subfamily T, member 3 [Source:HGNC Symbol;Acc:15297]	77.02
	OR51M1	olfactory receptor, family 51, subfamily M, member 1 [Source:HGNC Symbol;Acc:14847]	74.28
	OR10AG1	olfactory receptor, family 10, subfamily AG, member 1 [Source:HGNC Symbol;Acc:19607]	77.79
	OR1L8	olfactory receptor, family 1, subfamily L, member 8 [Source:HGNC Symbol;Acc:15110]	81.92
	OR51Q1	olfactory receptor, family 51, subfamily Q, member 1 (gene/pseudogene) [Source:HGNC Symbol;Acc:14851]	83.84
	OR7G3	olfactory receptor, family 7, subfamily G, member 3 [Source:HGNC Symbol;Acc:8467]	74.25
	OR10G3	olfactory receptor, family 10, subfamily G, member 3 [Source:HGNC Symbol;Acc:8171]	80.30
	OR10H3	olfactory receptor, family 10, subfamily H, member 3 [Source:HGNC Symbol;Acc:8174]	76.38
	OR4S2	olfactory receptor, family 4, subfamily S, member 2 [Source:HGNC Symbol;Acc:15183]	80.29
	OR8S1	olfactory receptor, family 8, subfamily S, member 1 [Source:HGNC Symbol;Acc:19628]	83.30
	OR5A1	olfactory receptor, family 5, subfamily A, member 1 [Source:HGNC Symbol;Acc:8319]	79.61
	OR8J1	olfactory receptor, family 8, subfamily J, member 1 [Source:HGNC Symbol;Acc:14855]	85.84
	OR1B1	olfactory receptor, family 1, subfamily B, member 1 [Source:HGNC Symbol;Acc:8181]	76.37
	OR52W1	olfactory receptor, family 52, subfamily W, member 1 [Source:HGNC Symbol;Acc:15239]	74.27
	OR2A14	olfactory receptor, family 2, subfamily A, member 14 [Source:HGNC Symbol;Acc:15084]	65.96
	OR2D2	olfactory receptor, family 2, subfamily D, member 2 [Source:HGNC Symbol;Acc:8244]	84.72
	OR8U1	olfactory receptor, family 8, subfamily U, member 1 [Source:HGNC Symbol;Acc:19611]	97.08
	OR51B4	olfactory receptor, family 51, subfamily B, member 4 [Source:HGNC Symbol;Acc:14708]	81.88
	OR51B6	olfactory receptor, family 51, subfamily B, member 6 [Source:HGNC Symbol;Acc:19600]	83.32
	OR6V1	olfactory receptor, family 6, subfamily V, member 1 [Source:HGNC Symbol;Acc:15090]	64.25
	OR9A2	olfactory receptor, family 9, subfamily A, member 2 [Source:HGNC Symbol;Acc:15093]	73.02
	OR52N4	olfactory receptor, family 52, subfamily N, member 4 (gene/pseudogene) [Source:HGNC Symbol;Acc:15230]	-
	OR4K5	olfactory receptor, family 4, subfamily K, member 5 [Source:HGNC Symbol;Acc:14745]	78.43
	OR7A17	olfactory receptor, family 7, subfamily A, member 17 [Source:HGNC Symbol;Acc:8363]	72.19
	OR5C1	olfactory receptor, family 5, subfamily C, member 1 [Source:HGNC Symbol;Acc:8331]	82.41
	OR4X2	olfactory receptor, family 4, subfamily X, member 2 [Source:HGNC Symbol;Acc:15184]	85.11

Enrichment Type	Enrichment Term	P-value of enrichment	FDR
GOTERM_BP_FAT	GO:0007606~sensory perception of chemical stimulus	$<7 \times 10^{-307}$	$<7 \times 10^{-307}$
GOTERM_BP_FAT	GO:0007608~sensory perception of smell	$<7 \times 10^{-307}$	$<7 \times 10^{-307}$
GOTERM_MF_FAT	GO:0004984~olfactory receptor activity	$<7 \times 10^{-307}$	$<7 \times 10^{-307}$
INTERPRO	IPR000276:7TM GPCR rhodopsin-like	$<7 \times 10^{-307}$	$<7 \times 10^{-307}$
INTERPRO	IPR017452:GPCR rhodopsin-like superfamily	$<7 \times 10^{-307}$	$<7 \times 10^{-307}$
INTERPRO	IPR000725:Olfactory receptor	$<7 \times 10^{-307}$	$<7 \times 10^{-307}$
SP_PIR_KEYWORDS	transducer	$<7 \times 10^{-307}$	$<7 \times 10^{-307}$
SP_PIR_KEYWORDS	olfaction	$<7 \times 10^{-307}$	$<7 \times 10^{-307}$
SP_PIR_KEYWORDS	sensory transduction	$<7 \times 10^{-307}$	$<7 \times 10^{-307}$
SP_PIR_KEYWORDS	g-protein coupled receptor	$<7 \times 10^{-307}$	$<7 \times 10^{-307}$
GOTERM_BP_FAT	GO:0007600~sensory perception	$<7 \times 10^{-307}$	4.7E-307
KEGG_PATHWAY	hsa04740:Olfactory transduction	6.8E-307	3.0E-304
GOTERM_BP_FAT	GO:0050890~cognition	8.4E-296	9.1E-293
GOTERM_BP_FAT	GO:0007186~G-protein coupled receptor protein signaling pathway	2.7E-270	2.9E-267
SP_PIR_KEYWORDS	receptor	1.1E-264	1.0E-261
GOTERM_BP_FAT	GO:0050877~neurological system process	1.8E-261	1.9E-258
PIR_SUPERFAMILY	PIRSF800006:rhodopsin-like G protein-coupled receptors	1.5E-259	7.6E-257
PIR_SUPERFAMILY	PIRSF003152:G protein-coupled olfactory receptor	3.7E-251	1.9E-248
SP_PIR_KEYWORDS	cell membrane	5.4E-228	5.1E-225
GOTERM_BP_FAT	GO:0007166~cell surface receptor linked signal transduction	3.5E-215	3.8E-212
UP_SEQ_FEATURE	topological domain:Extracellular	3.9E-200	5.1E-197
UP_SEQ_FEATURE	topological domain:Cytoplasmic	8.9E-177	1.2E-173
UP_SEQ_FEATURE	disulfide bond	6.1E-152	8.0E-149
SP_PIR_KEYWORDS	disulfide bond	4.0E-148	3.8E-145
UP_SEQ_FEATURE	transmembrane region	1.3E-136	1.7E-133
SP_PIR_KEYWORDS	transmembrane	1.5E-136	1.4E-133
UP_SEQ_FEATURE	glycosylation site:N-linked (GlcNAc...)	2.5E-133	3.3E-130
GOTERM_CC_FAT	GO:0005886~plasma membrane	2.2E-132	1.5E-129
SP_PIR_KEYWORDS	glycoprotein	2.2E-128	2.1E-125
SP_PIR_KEYWORDS	membrane	2.8E-114	2.6E-111
GOTERM_CC_FAT	GO:0016021~integral to membrane	1.2E-88	8.1E-86
GOTERM_CC_FAT	GO:0031224~intrinsic to membrane	6.2E-85	4.1E-82
PIR_SUPERFAMILY	PIRSF038651:G protein-coupled olfactory receptor	1.7E-41	8.8E-39
SP_PIR_KEYWORDS	polymorphism	7.1E-19	6.6E-16
UP_SEQ_FEATURE	sequence variant	1.3E-15	1.6E-12
OMIM_DISEASE	Genome-wide association and linkage analyses of hemostatic factors and hematological phenotypes in the Framingham Heart Study	1.1E-11	3.6E-09
INTERPRO	IPR015544:Olfactory receptor MOR256	1.2E-11	1.0E-08
INTERPRO	IPR015545:Olfactory receptor MOR105-like	6.1E-05	5.3E-02
INTERPRO	IPR015539:Olfactory receptor MOR103	3.1E-03	2.7E+00

OR4B1	olfactory receptor, family 4, subfamily B, member 1 [Source:HGNC Symbol;Acc:8290]	81.35
OR1A1	olfactory receptor, family 1, subfamily A, member 1 [Source:HGNC Symbol;Acc:8179]	47.39
OR4A16	olfactory receptor, family 4, subfamily A, member 16 [Source:HGNC Symbol;Acc:15153]	96.97
OR4E2	olfactory receptor, family 4, subfamily E, member 2 [Source:HGNC Symbol;Acc:8297]	45.80
OR2Y1	olfactory receptor, family 2, subfamily Y, member 1 [Source:HGNC Symbol;Acc:14837]	75.75
OR4K1	olfactory receptor, family 4, subfamily K, member 1 [Source:HGNC Symbol;Acc:14726]	82.42
OR5R1	olfactory receptor, family 5, subfamily R, member 1 [Source:HGNC Symbol;Acc:14841]	81.87
ARRB2	arrestin, beta 2 [Source:HGNC Symbol;Acc:712]	68.05
OR10A3	olfactory receptor, family 10, subfamily A, member 3 [Source:HGNC Symbol;Acc:8162]	77.80
OR52A5	olfactory receptor, family 52, subfamily A, member 5 [Source:HGNC Symbol;Acc:19580]	76.34
OR2T10	olfactory receptor, family 2, subfamily T, member 10 [Source:HGNC Symbol;Acc:19573]	72.21
OR6S1	olfactory receptor, family 6, subfamily S, member 1 [Source:HGNC Symbol;Acc:15363]	83.31
OR7D4	olfactory receptor, family 7, subfamily D, member 4 [Source:HGNC Symbol;Acc:8380]	81.32
OR2C3	olfactory receptor, family 2, subfamily C, member 3 [Source:HGNC Symbol;Acc:15005]	76.35
OR2C1	olfactory receptor, family 2, subfamily C, member 1 [Source:HGNC Symbol;Acc:8242]	80.75
OR4K17	olfactory receptor, family 4, subfamily K, member 17 [Source:HGNC Symbol;Acc:15355]	75.01
OR7A10	olfactory receptor, family 7, subfamily A, member 10 [Source:HGNC Symbol;Acc:8356]	81.32
OR6M1	olfactory receptor, family 6, subfamily M, member 1 [Source:HGNC Symbol;Acc:14711]	74.99
OR5D18	olfactory receptor, family 5, subfamily D, member 18 [Source:HGNC Symbol;Acc:15285]	73.04
OR6F1	olfactory receptor, family 6, subfamily F, member 1 [Source:HGNC Symbol;Acc:15027]	82.89
OR5P3	olfactory receptor, family 5, subfamily P, member 3 [Source:HGNC Symbol;Acc:14784]	82.40
OR14A16	olfactory receptor, family 14, subfamily A, member 16 [Source:HGNC Symbol;Acc:15022]	75.03
OR4D6	olfactory receptor, family 4, subfamily D, member 6 [Source:HGNC Symbol;Acc:15175]	84.24
OR8H1	olfactory receptor, family 8, subfamily H, member 1 [Source:HGNC Symbol;Acc:14824]	80.26
OR7C1	olfactory receptor, family 7, subfamily C, member 1 [Source:HGNC Symbol;Acc:8373]	74.99
OR5D14	olfactory receptor, family 5, subfamily D, member 14 [Source:HGNC Symbol;Acc:15281]	79.60
OR5W2	olfactory receptor, family 5, subfamily W, member 2 [Source:HGNC Symbol;Acc:15299]	77.02
OR8B4	olfactory receptor, family 8, subfamily B, member 4 [Source:HGNC Symbol;Acc:8473]	73.68
OR52B6	olfactory receptor, family 52, subfamily B, member 6 [Source:HGNC Symbol;Acc:15211]	69.34
OR5M8	olfactory receptor, family 5, subfamily M, member 8 [Source:HGNC Symbol;Acc:14846]	77.75
OR4D5	olfactory receptor, family 4, subfamily D, member 5 [Source:HGNC Symbol;Acc:14852]	80.74
OR52E6	olfactory receptor, family 52, subfamily E, member 6 [Source:HGNC Symbol;Acc:15215]	78.41
OR10H2	olfactory receptor, family 10, subfamily H, member 2 [Source:HGNC Symbol;Acc:8173]	88.05
OR1N2	olfactory receptor, family 1, subfamily N, member 2 [Source:HGNC Symbol;Acc:15111]	81.91
OR4K2	olfactory receptor, family 4, subfamily K, member 2 [Source:HGNC Symbol;Acc:14728]	78.43
OR2V2	olfactory receptor, family 2, subfamily V, member 2 [Source:HGNC Symbol;Acc:15341]	81.89
OR5J2	olfactory receptor, family 5, subfamily J, member 2 [Source:HGNC Symbol;Acc:19612]	77.03
OR52D1	olfactory receptor, family 52, subfamily D, member 1 [Source:HGNC Symbol;Acc:15212]	80.72
OR4C11	olfactory receptor, family 4, subfamily C, member 11 [Source:HGNC Symbol;Acc:15167]	81.34
OR4S1	olfactory receptor, family 4, subfamily S, member 1 [Source:HGNC Symbol;Acc:14705]	84.24
OR10K2	olfactory receptor, family 10, subfamily K, member 2 [Source:HGNC Symbol;Acc:14826]	75.03
OR8I2	olfactory receptor, family 8, subfamily I, member 2 [Source:HGNC Symbol;Acc:15310]	83.82
OR5T1	olfactory receptor, family 5, subfamily T, member 1 [Source:HGNC Symbol;Acc:14821]	79.01
OR9I1	olfactory receptor, family 9, subfamily I, member 1 [Source:HGNC Symbol;Acc:14718]	79.00
OR6B1	olfactory receptor, family 6, subfamily B, member 1 [Source:HGNC Symbol;Acc:8354]	63.41
OR5B3	olfactory receptor, family 5, subfamily B, member 3 [Source:HGNC Symbol;Acc:8324]	73.69
OR4A5	olfactory receptor, family 4, subfamily A, member 5 [Source:HGNC Symbol;Acc:15162]	93.77
ADRBK2	adrenergic, beta, receptor kinase 2 [Source:HGNC Symbol;Acc:290]	85.34
OR8D2	olfactory receptor, family 8, subfamily D, member 2 [Source:HGNC Symbol;Acc:8482]	75.71
OR2T11	olfactory receptor, family 2, subfamily T, member 11 (gene/pseudogene) [Source:HGNC Symbol;Acc:19574]	79.03
OR1F1	olfactory receptor, family 1, subfamily F, member 1 [Source:HGNC Symbol;Acc:8194]	63.13
OR10P1	olfactory receptor, family 10, subfamily P, member 1 [Source:HGNC Symbol;Acc:15378]	81.37
OR10Q1	olfactory receptor, family 10, subfamily Q, member 1 [Source:HGNC Symbol;Acc:15134]	73.05
OR2AT4	olfactory receptor, family 2, subfamily AT, member 4 [Source:HGNC Symbol;Acc:19620]	46.16
OR5K4	olfactory receptor, family 5, subfamily K, member 4 [Source:HGNC Symbol;Acc:31291]	80.27
OR4F6	olfactory receptor, family 4, subfamily F, member 6 [Source:HGNC Symbol;Acc:15372]	67.63
OR14I1	olfactory receptor, family 14, subfamily I, member 1 [Source:HGNC Symbol;Acc:19575]	80.76
OR2B2	olfactory receptor, family 2, subfamily B, member 2 [Source:HGNC Symbol;Acc:13966]	68.46
OR56A1	olfactory receptor, family 56, subfamily A, member 1 [Source:HGNC Symbol;Acc:14781]	77.04
OR1D2	olfactory receptor, family 1, subfamily D, member 2 [Source:HGNC Symbol;Acc:8183]	75.76
OR5B12	olfactory receptor, family 5, subfamily B, member 12 [Source:HGNC Symbol;Acc:15432]	77.76
OR56A3	olfactory receptor, family 56, subfamily A, member 3 [Source:HGNC Symbol;Acc:14786]	57.77
OR1J1	olfactory receptor, family 1, subfamily J, member 1 [Source:HGNC Symbol;Acc:8208]	72.23
OR8K3	olfactory receptor, family 8, subfamily K, member 3 [Source:HGNC Symbol;Acc:15313]	77.01
OR8B12	olfactory receptor, family 8, subfamily B, member 12 [Source:HGNC Symbol;Acc:15307]	71.45
OR1A2	olfactory receptor, family 1, subfamily A, member 2 [Source:HGNC Symbol;Acc:8180]	46.61
OR8B8	olfactory receptor, family 8, subfamily B, member 8 [Source:HGNC Symbol;Acc:8477]	78.38
OR9G4	olfactory receptor, family 9, subfamily G, member 4 [Source:HGNC Symbol;Acc:15322]	81.86
OR1M1	olfactory receptor, family 1, subfamily M, member 1 [Source:HGNC Symbol;Acc:8220]	82.44
OR9K2	olfactory receptor, family 9, subfamily K, member 2 [Source:HGNC Symbol;Acc:15339]	80.25
OR5AU1	olfactory receptor, family 5, subfamily AU, member 1 [Source:HGNC Symbol;Acc:15362]	87.09
OR5AC2	olfactory receptor, family 5, subfamily AC, member 2 [Source:HGNC Symbol;Acc:15431]	75.75
OR52N2	olfactory receptor, family 52, subfamily N, member 2 [Source:HGNC Symbol;Acc:15228]	81.33
OR6Q1	olfactory receptor, family 6, subfamily Q, member 1 [Source:HGNC Symbol;Acc:15302]	73.68
OR6T1	olfactory receptor, family 6, subfamily T, member 1 [Source:HGNC Symbol;Acc:14848]	81.87
OR4P4	olfactory receptor, family 4, subfamily P, member 4 [Source:HGNC Symbol;Acc:15180]	65.95
OR6Y1	olfactory receptor, family 6, subfamily Y, member 1 [Source:HGNC Symbol;Acc:14823]	82.89
OR2A2	olfactory receptor, family 2, subfamily A, member 2 [Source:HGNC Symbol;Acc:8230]	73.71
OR6C2	olfactory receptor, family 6, subfamily C, member 2 [Source:HGNC Symbol;Acc:15436]	75.73
OR56A4	olfactory receptor, family 56, subfamily A, member 4 [Source:HGNC Symbol;Acc:14791]	80.71
OR4K14	olfactory receptor, family 4, subfamily K, member 14 [Source:HGNC Symbol;Acc:15352]	77.05
OR10H4	olfactory receptor, family 10, subfamily H, member 4 [Source:HGNC Symbol;Acc:15388]	80.77
OR6C75	olfactory receptor, family 6, subfamily C, member 75 [Source:HGNC Symbol;Acc:31304]	69.32
OR8J3	olfactory receptor, family 8, subfamily J, member 3 [Source:HGNC Symbol;Acc:15312]	79.59
OR1G1	olfactory receptor, family 1, subfamily G, member 1 [Source:HGNC Symbol;Acc:8204]	76.36
OR5AP2	olfactory receptor, family 5, subfamily AP, member 2 [Source:HGNC Symbol;Acc:15258]	77.03
OR4C46	olfactory receptor, family 4, subfamily C, member 46 [Source:HGNC Symbol;Acc:31271]	92.04
OR2AK2	olfactory receptor, family 2, subfamily AK, member 2 [Source:HGNC Symbol;Acc:19569]	80.75
OR2AG1	olfactory receptor, family 2, subfamily AG, member 1 [Source:HGNC Symbol;Acc:15142]	81.91
OR10J5	olfactory receptor, family 10, subfamily J, member 5 [Source:HGNC Symbol;Acc:14993]	70.82
OR2B6	olfactory receptor, family 2, subfamily B, member 6 [Source:HGNC Symbol;Acc:8241]	72.22
OR51E2	olfactory receptor, family 51, subfamily E, member 2 [Source:HGNC Symbol;Acc:15195]	78.42
OR2M4	olfactory receptor, family 2, subfamily M, member 4 [Source:HGNC Symbol;Acc:8270]	66.76

Cluster 2 - tolerant	Gene Symbol	Gene description	EvoTol %ile
Mann-Whitney U test $P = 9.7 \times 10^{-6}$	*EDNRA*	endothelin receptor type A [Source:HGNC Symbol;Acc:3179]	55.89
	GRPR	gastrin-releasing peptide receptor [Source:HGNC Symbol;Acc:4609]	75.88
	PROK2	prokineticin 2 [Source:HGNC Symbol;Acc:18455]	27.36
	AVPR1B	arginine vasopressin receptor 1B [Source:HGNC Symbol;Acc:896]	50.80
	P2RY2	purinergic receptor P2Y, G-protein coupled, 2 [Source:HGNC Symbol;Acc:8541]	53.79
	BDKRB2	bradykinin receptor B2 [Source:HGNC Symbol;Acc:1030]	97.31
	GPR4	G protein-coupled receptor 4 [Source:HGNC Symbol;Acc:4497]	66.11
	NPW	neuropeptide W [Source:HGNC Symbol;Acc:30509]	48.76
	LPAR2	lysophosphatidic acid receptor 2 [Source:HGNC Symbol;Acc:3168]	69.45
	GNA15	guanine nucleotide binding protein (G protein), alpha 15 (Gq class) [Source:HGNC Symbol;Acc:4383]	**11.21**
	DHX9	DEAH (Asp-Glu-Ala-His) box helicase 9 [Source:HGNC Symbol;Acc:2750]	36.37
	FPR2	formyl peptide receptor 2 [Source:HGNC Symbol;Acc:3827]	47.98
	CCL25	chemokine (C-C motif) ligand 25 [Source:HGNC Symbol;Acc:10624]	56.57
	GRP	gastrin-releasing peptide [Source:HGNC Symbol;Acc:4605]	52.85
	CCAR1	cell division cycle and apoptosis regulator 1 [Source:HGNC Symbol;Acc:24236]	90.46
	AGTR1	angiotensin II receptor, type 1 [Source:HGNC Symbol;Acc:336]	48.66
	NPB	neuropeptide B [Source:HGNC Symbol;Acc:30099]	27.14
	C5	complement component 5 [Source:HGNC Symbol;Acc:1331]	98.34
	C5AR1	complement component 5a receptor 1 [Source:HGNC Symbol;Acc:1338]	62.32
	CXCL6	chemokine (C-X-C motif) ligand 6 [Source:HGNC Symbol;Acc:10643]	54.99
	POLR2E	polymerase (RNA) II (DNA directed) polypeptide E, 25kDa [Source:HGNC Symbol;Acc:9192]	39.59
	MLNR	motilin receptor [Source:HGNC Symbol;Acc:4495]	87.71
	HTR1A	5-hydroxytryptamine (serotonin) receptor 1A, G protein-coupled [Source:HGNC Symbol;Acc:5286]	76.11
	CXCL9	chemokine (C-X-C motif) ligand 9 [Source:HGNC Symbol;Acc:7098]	51.14
	EDN3	endothelin 3 [Source:HGNC Symbol;Acc:3178]	**10.38**
	POLR2F	polymerase (RNA) II (DNA directed) polypeptide F [Source:HGNC Symbol;Acc:9193]	25.46
	SNRPG	small nuclear ribonucleoprotein polypeptide G [Source:HGNC Symbol;Acc:11163]	47.68
	CCL28	chemokine (C-C motif) ligand 28 [Source:HGNC Symbol;Acc:17700]	58.00
	NPFF	neuropeptide FF-amide peptide precursor [Source:HGNC Symbol;Acc:7901]	54.09
	PRPF4	pre-mRNA processing factor 4 [Source:HGNC Symbol;Acc:17349]	46.93
	DRD2	dopamine receptor D2 [Source:HGNC Symbol;Acc:3023]	95.89
	GNA14	guanine nucleotide binding protein (G protein), alpha 14 [Source:HGNC Symbol;Acc:4382]	**8.99**
	NPS	neuropeptide S [Source:HGNC Symbol;Acc:33940]	52.16
	POLR2I	polymerase (RNA) II (DNA directed) polypeptide I, 14.5kDa [Source:HGNC Symbol;Acc:9196]	50.94
	CCL20	chemokine (C-C motif) ligand 20 [Source:HGNC Symbol;Acc:10619]	50.57
	S1PR1	sphingosine-1-phosphate receptor 1 [Source:HGNC Symbol;Acc:3165]	85.45
	CHRM4	cholinergic receptor, muscarinic 4 [Source:HGNC Symbol;Acc:1953]	96.28
	ANXA1	annexin A1 [Source:HGNC Symbol;Acc:533]	72.66
	PTGFR	prostaglandin F receptor (FP) [Source:HGNC Symbol;Acc:9600]	30.80
	P2RY6	pyrimidinergic receptor P2Y, G-protein coupled, 6 [Source:HGNC Symbol;Acc:8543]	74.98
	UTS2	urotensin 2 [Source:HGNC Symbol;Acc:12636]	59.98
	DRD3	dopamine receptor D3 [Source:HGNC Symbol;Acc:3024]	94.96
	LPAR6	lysophosphatidic acid receptor 6 [Source:HGNC Symbol;Acc:15520]	74.38
	CXCL5	chemokine (C-X-C motif) ligand 5 [Source:HGNC Symbol;Acc:10642]	**16.78**
	AVPR1A	arginine vasopressin receptor 1A [Source:HGNC Symbol;Acc:895]	74.67
	MTNR1B	melatonin receptor 1B [Source:HGNC Symbol;Acc:7464]	35.54
	ADRA1D	adrenoceptor alpha 1D [Source:HGNC Symbol;Acc:280]	41.84
	CHRM1	cholinergic receptor, muscarinic 1 [Source:HGNC Symbol;Acc:1950]	73.95
	GNRHR	gonadotropin-releasing hormone receptor [Source:HGNC Symbol;Acc:4421]	40.98
	F2RL1	coagulation factor II (thrombin) receptor-like 1 [Source:HGNC Symbol;Acc:3538]	74.50
	GPR17	G protein-coupled receptor 17 [Source:HGNC Symbol;Acc:4471]	32.79
	GPR65	G protein-coupled receptor 65 [Source:HGNC Symbol;Acc:4517]	63.61
	EDNRB	endothelin receptor type B [Source:HGNC Symbol;Acc:3180]	26.88
	S1PR4	sphingosine-1-phosphate receptor 4 [Source:HGNC Symbol;Acc:3170]	28.19
	APP	amyloid beta (A4) precursor protein [Source:HGNC Symbol;Acc:620]	**1.19**
	SSTR4	somatostatin receptor 4 [Source:HGNC Symbol;Acc:11333]	77.60
	UTS2D	-	53.13
	MTNR1A	melatonin receptor 1A [Source:HGNC Symbol;Acc:7463]	80.81
	F2RL2	coagulation factor II (thrombin) receptor-like 2 [Source:HGNC Symbol;Acc:3539]	36.82
	CCL5	chemokine (C-C motif) ligand 5 [Source:HGNC Symbol;Acc:10632]	52.31
	OXTR	oxytocin receptor [Source:HGNC Symbol;Acc:8529]	84.11
	P2RY13	purinergic receptor P2Y, G-protein coupled, 13 [Source:HGNC Symbol;Acc:4537]	69.31
	SSTR3	somatostatin receptor 3 [Source:HGNC Symbol;Acc:11332]	80.03

Enrichment Type	Enrichment Term	P-value of enrichment	FDR
GOTERM_BP_FAT	GO:0007186~G-protein coupled receptor protein signaling pathway	1.69E-113	2.80E-110
GOTERM_BP_FAT	GO:0000377~RNA splicing, via transesterification reactions with bulged adenosine as nucleophile	1.34E-111	2.21E-108
GOTERM_BP_FAT	GO:0000398~nuclear mRNA splicing, via spliceosome	1.34E-111	2.21E-108
GOTERM_BP_FAT	GO:0000375~RNA splicing, via transesterification reactions	1.34E-111	2.21E-108
SP_PIR_KEYWORDS	transducer	2.24E-96	2.84E-93
SP_PIR_KEYWORDS	g-protein coupled receptor	7.47E-95	9.47E-92
INTERPRO	IPR017452:GPCR, rhodopsin-like superfamily	4.94E-92	6.86E-89
INTERPRO	IPR000276:7TM GPCR, rhodopsin-like	5.86E-92	8.14E-89
GOTERM_BP_FAT	GO:0008380~RNA splicing	6.37E-82	1.05E-78
GOTERM_BP_FAT	GO:0007166~cell surface receptor linked signal transduction	1.20E-80	1.99E-77
GOTERM_BP_FAT	GO:0006397~mRNA processing	3.98E-78	6.59E-75
KEGG_PATHWAY	hsa04080:Neuroactive ligand-receptor interaction	4.41E-78	3.90E-75
SP_PIR_KEYWORDS	G protein-coupled receptor	2.09E-77	2.65E-74
GOTERM_BP_FAT	GO:0016071~mRNA metabolic process	3.67E-72	6.08E-69
GOTERM_MF_FAT	GO:0008528~peptide receptor activity, G-protein coupled	4.39E-72	5.87E-69
GOTERM_MF_FAT	GO:0001653~peptide receptor activity	4.39E-72	5.87E-69
PIR_SUPERFAMILY	PIRSF800006:rhodopsin-like G protein-coupled receptors	1.75E-64	2.08E-61
SP_PIR_KEYWORDS	receptor	1.93E-61	2.44E-58
SP_PIR_KEYWORDS	mrna processing	2.33E-60	2.96E-57
GOTERM_BP_FAT	GO:0019932~second-messenger-mediated signaling	6.57E-60	1.09E-56
GOTERM_BP_FAT	GO:0006396~RNA processing	1.30E-56	2.16E-53
GOTERM_MF_FAT	GO:0042277~peptide binding	6.66E-54	8.89E-51
GOTERM_BP_FAT	GO:0007610~behavior	9.89E-50	1.64E-46
SP_PIR_KEYWORDS	mrna splicing	7.71E-49	9.78E-46
SP_PIR_KEYWORDS	disulfide bond	1.02E-47	1.29E-44
UP_SEQ_FEATURE	disulfide bond	6.79E-47	1.03E-43
SP_PIR_KEYWORDS	cell membrane	1.63E-46	2.07E-43
GOTERM_BP_FAT	GO:0007204~elevation of cytosolic calcium ion concentration	4.88E-45	8.09E-42
GOTERM_BP_FAT	GO:0051480~cytosolic calcium ion homeostasis	5.40E-45	8.94E-42
GOTERM_BP_FAT	GO:0006874~cellular calcium ion homeostasis	2.14E-43	3.54E-40
GOTERM_BP_FAT	GO:0055074~calcium ion homeostasis	9.43E-43	1.56E-39
GOTERM_BP_FAT	GO:0006875~cellular metal ion homeostasis	9.16E-42	1.52E-38
GOTERM_BP_FAT	GO:0055065~metal ion homeostasis	1.04E-40	1.72E-37
SP_PIR_KEYWORDS	Spliceosome	1.13E-39	1.43E-36
GOTERM_BP_FAT	GO:0030005~cellular di-, tri-valent inorganic cation homeostasis	2.34E-38	3.87E-35
UP_SEQ_FEATURE	lipid moiety-binding region:S-palmitoyl cysteine	1.04E-37	1.58E-34
GOTERM_BP_FAT	GO:0010863~positive regulation of phospholipase C activity	1.09E-37	1.81E-34
GOTERM_BP_FAT	GO:0007202~activation of phospholipase C activity	1.09E-37	1.81E-34
SP_PIR_KEYWORDS	palmitate	2.33E-37	2.96E-34
GOTERM_BP_FAT	GO:0055066~di-, tri-valent inorganic cation homeostasis	3.45E-37	5.72E-34
GOTERM_BP_FAT	GO:0010518~positive regulation of phospholipase activity	1.33E-36	2.20E-33
UP_SEQ_FEATURE	topological domain:Extracellular	2.19E-36	3.33E-33
GOTERM_BP_FAT	GO:0010517~regulation of phospholipase activity	4.30E-36	7.13E-33
GOTERM_BP_FAT	GO:0030003~cellular cation homeostasis	8.02E-36	1.33E-32
GOTERM_CC_FAT	GO:0005681~spliceosome	3.25E-35	3.90E-32
GOTERM_BP_FAT	GO:0060193~positive regulation of lipase activity	3.95E-35	6.54E-32
GOTERM_BP_FAT	GO:0060191~regulation of lipase activity	3.86E-34	6.39E-31
GOTERM_BP_FAT	GO:0007187~G-protein signaling, coupled to cyclic nucleotide second messenger	1.11E-33	1.84E-30
GOTERM_CC_FAT	GO:0005887~integral to plasma membrane	1.26E-33	1.51E-30
GOTERM_BP_FAT	GO:0055080~cation homeostasis	3.29E-33	5.45E-30
GOTERM_BP_FAT	GO:0019935~cyclic-nucleotide-mediated signaling	7.00E-33	1.16E-29
GOTERM_CC_FAT	GO:0031226~intrinsic to plasma membrane	7.72E-33	9.26E-30
SP_PIR_KEYWORDS	transmembrane protein	2.28E-32	2.89E-29
KEGG_PATHWAY	hsa03040:Spliceosome	1.88E-31	1.66E-28
GOTERM_BP_FAT	GO:0007200~activation of phospholipase C activity by G-protein coupled receptor protein signaling pathway coupled to IP3 second m[illegible]	5.25E-31	8.69E-28
GOTERM_BP_FAT	GO:0007626~locomotory behavior	7.96E-31	1.32E-27
GOTERM_BP_FAT	GO:0048015~phosphoinositide-mediated signaling	1.83E-29	3.03E-26
GOTERM_BP_FAT	GO:0006873~cellular ion homeostasis	1.67E-28	2.76E-25
GOTERM_BP_FAT	GO:0055082~cellular chemical homeostasis	3.61E-28	5.98E-25
GOTERM_BP_FAT	GO:0042330~taxis	4.84E-28	8.01E-25
GOTERM_BP_FAT	GO:0006935~chemotaxis	4.84E-28	8.01E-25
GOTERM_MF_FAT	GO:0008188~neuropeptide receptor activity	7.06E-28	9.43E-25
GOTERM_MF_FAT	GO:0042923~neuropeptide binding	1.50E-27	2.00E-24

QRFP	pyroglutamylated RFamide peptide [Source:HGNC Symbol;Acc:29982]	68.38	UP_SEQ_FEATURE	topological domain:Cytoplasmic	3.90E-27	5.93E-24
GNG2	guanine nucleotide binding protein (G protein), gamma 2 [Source:HGNC Symbol;Acc:4404]	49.52	SP_PIR_KEYWORDS	rna-binding	9.97E-27	1.26E-23
CCR1	chemokine (C-C motif) receptor 1 [Source:HGNC Symbol;Acc:1602]	92.09	GOTERM_BP_FAT	GO:0050801~ion homeostasis	1.23E-26	2.03E-23
KNG1	kininogen 1 [Source:HGNC Symbol;Acc:6383]	85.56	SMART	SM00360:RRM	1.16E-25	1.14E-22
OPN4	opsin 4 [Source:HGNC Symbol;Acc:14449]	66.45	SP_PIR_KEYWORDS	neuropeptide	9.81E-25	1.24E-21
EDN1	endothelin 1 [Source:HGNC Symbol;Acc:3176]	**9.63**	GOTERM_BP_FAT	GO:0048878~chemical homeostasis	1.28E-24	2.13E-21
GCG	glucagon [Source:HGNC Symbol;Acc:4191]	60.41	GOTERM_BP_FAT	GO:0007242~intracellular signaling cascade	5.17E-24	8.57E-21
RXFP3	relaxin/insulin-like family peptide receptor 3 [Source:HGNC Symbol;Acc:24883]	76.23	GOTERM_BP_FAT	GO:0019725~cellular homeostasis	5.52E-24	9.15E-21
SFRS7	-	52.07	GOTERM_BP_FAT	GO:0051345~positive regulation of hydrolase activity	6.04E-24	1.00E-20
SNRPD2	small nuclear ribonucleoprotein D2 polypeptide 16.5kDa [Source:HGNC Symbol;Acc:11159]	34.56	GOTERM_BP_FAT	GO:0043085~positive regulation of catalytic activity	1.86E-23	3.08E-20
CXCR6	chemokine (C-X-C motif) receptor 6 [Source:HGNC Symbol;Acc:16647]	68.76	GOTERM_MF_FAT	GO:0030594~neurotransmitter receptor activity	3.68E-23	4.92E-20
PCBP1	poly(rC) binding protein 1 [Source:HGNC Symbol;Acc:8647]	61.13	SP_PIR_KEYWORDS	chemotaxis	2.42E-22	3.07E-19
CXCL3	chemokine (C-X-C motif) ligand 3 [Source:HGNC Symbol;Acc:4604]	59.59	GOTERM_MF_FAT	GO:0008227~amine receptor activity	2.46E-22	3.29E-19
HNRNPD	heterogeneous nuclear ribonucleoprotein D (AU-rich element RNA binding protein 1, 37kDa) [Source:HGNC Symbol;Acc:5036]	34.58	GOTERM_MF_FAT	GO:0042165~neurotransmitter binding	3.61E-22	4.82E-19
CD2BP2	CD2 (cytoplasmic tail) binding protein 2 [Source:HGNC Symbol;Acc:1656]	72.54	GOTERM_BP_FAT	GO:0030817~regulation of cAMP biosynthetic process	4.08E-22	6.76E-19
HRH3	histamine receptor H3 [Source:HGNC Symbol;Acc:5184]	92.55	GOTERM_BP_FAT	GO:0030814~regulation of cAMP metabolic process	7.06E-22	1.17E-18
TRHR	thyrotropin-releasing hormone receptor [Source:HGNC Symbol;Acc:12299]	67.35	GOTERM_BP_FAT	GO:0030802~regulation of cyclic nucleotide biosynthetic process	4.37E-21	7.23E-18
TACR3	tachykinin receptor 3 [Source:HGNC Symbol;Acc:11528]	47.90	GOTERM_BP_FAT	GO:0030808~regulation of nucleotide biosynthetic process	4.37E-21	7.23E-18
HNRNPR	heterogeneous nuclear ribonucleoprotein R [Source:HGNC Symbol;Acc:5047]	70.21	GOTERM_CC_FAT	GO:0030529~ribonucleoprotein complex	4.55E-21	5.45E-18
FPR3	formyl peptide receptor 3 [Source:HGNC Symbol;Acc:3828]	45.98	GOTERM_BP_FAT	GO:0044093~positive regulation of molecular function	4.76E-21	7.88E-18
XCR1	chemokine (C motif) receptor 1 [Source:HGNC Symbol;Acc:1625]	46.56	INTERPRO	IPR012677:Nucleotide-binding, alpha-beta plait	5.70E-21	7.92E-18
FPR1	formyl peptide receptor 1 [Source:HGNC Symbol;Acc:3826]	53.09	SP_PIR_KEYWORDS	lipoprotein	7.76E-21	9.84E-18
NPBWR1	neuropeptides B/W receptor 1 [Source:HGNC Symbol;Acc:4522]	46.32	GOTERM_BP_FAT	GO:0030799~regulation of cyclic nucleotide metabolic process	9.13E-21	1.51E-17
PPY	pancreatic polypeptide [Source:HGNC Symbol;Acc:9327]	52.13	GOTERM_BP_FAT	GO:0007267~cell-cell signaling	1.39E-20	2.31E-17
P2RY10	purinergic receptor P2Y, G-protein coupled, 10 [Source:HGNC Symbol;Acc:19906]	52.15	GOTERM_BP_FAT	GO:0006140~regulation of nucleotide metabolic process	1.86E-20	3.09E-17
CPSF1	cleavage and polyadenylation specific factor 1, 160kDa [Source:HGNC Symbol;Acc:2324]	91.29	GOTERM_BP_FAT	GO:0019933~cAMP-mediated signaling	2.75E-20	4.56E-17
HEBP1	heme binding protein 1 [Source:HGNC Symbol;Acc:17176]	66.09	GOTERM_BP_FAT	GO:0007188~G-protein signaling, coupled to cAMP nucleotide second messenger	3.14E-20	5.20E-17
PTBP1	polypyrimidine tract binding protein 1 [Source:HGNC Symbol;Acc:9583]	92.87	GOTERM_BP_FAT	GO:0008015~blood circulation	3.74E-20	6.20E-17
ADRA2A	adrenoceptor alpha 2A [Source:HGNC Symbol;Acc:281]	64.67	GOTERM_BP_FAT	GO:0003013~circulatory system process	3.74E-20	6.20E-17
NTSR1	neurotensin receptor 1 (high affinity) [Source:HGNC Symbol;Acc:8039]	93.32	INTERPRO	IPR000504:RNA recognition motif, RNP-1	5.00E-20	6.94E-17
SNRNP200	small nuclear ribonucleoprotein 200kDa (U5) [Source:HGNC Symbol;Acc:30859]	29.07	SMART	SM00199:SCY	8.30E-20	8.16E-17
GAST	gastrin [Source:HGNC Symbol;Acc:4164]	53.51	GOTERM_BP_FAT	GO:0007631~feeding behavior	9.37E-20	1.55E-16
CPSF7	cleavage and polyadenylation specific factor 7, 59kDa [Source:HGNC Symbol;Acc:30098]	**23.08**	GOTERM_BP_FAT	GO:0008217~regulation of blood pressure	1.02E-19	1.69E-16
HNRNPM	heterogeneous nuclear ribonucleoprotein M [Source:HGNC Symbol;Acc:5046]	66.47	GOTERM_MF_FAT	GO:0008009~chemokine activity	1.41E-19	1.88E-16
EDN2	endothelin 2 [Source:HGNC Symbol;Acc:3177]	**9.24**	SP_PIR_KEYWORDS	cleavage on pair of basic residues	1.84E-19	2.33E-16
CXCR7	-	81.54	GOTERM_BP_FAT	GO:0031280~negative regulation of cyclase activity	3.55E-19	5.88E-16
TBXA2R	thromboxane A2 receptor [Source:HGNC Symbol;Acc:11608]	68.97	GOTERM_BP_FAT	GO:0051350~negative regulation of lyase activity	3.55E-19	5.88E-16
PTGER1	prostaglandin E receptor 1 (subtype EP1), 42kDa [Source:HGNC Symbol;Acc:9593]	70.75	GOTERM_BP_FAT	GO:0007194~negative regulation of adenylate cyclase activity	3.55E-19	5.88E-16
GALR2	galanin receptor 2 [Source:HGNC Symbol;Acc:4133]	70.93	GOTERM_MF_FAT	GO:0005179~hormone activity	3.89E-19	5.20E-16
RBM8A	RNA binding motif protein 8A [Source:HGNC Symbol;Acc:9905]	49.43	GOTERM_MF_FAT	GO:0042379~chemokine receptor binding	5.46E-19	7.29E-16
PROKR2	prokineticin receptor 2 [Source:HGNC Symbol;Acc:15836]	54.51	GOTERM_BP_FAT	GO:0006954~inflammatory response	5.67E-19	9.39E-16
UTS2R	urotensin 2 receptor [Source:HGNC Symbol;Acc:4468]	38.78	GOTERM_BP_FAT	GO:0045761~regulation of adenylate cyclase activity	6.35E-19	1.05E-15
SFRS11	-	**18.83**	SP_PIR_KEYWORDS	glycoprotein	8.74E-19	1.11E-15
NPFFR2	neuropeptide FF receptor 2 [Source:HGNC Symbol;Acc:4525]	86.71	UP_SEQ_FEATURE	glycosylation site:N-linked (GlcNAc...)	9.73E-19	1.48E-15
HRH4	histamine receptor H4 [Source:HGNC Symbol;Acc:17383]	46.17	GOTERM_BP_FAT	GO:0031279~regulation of cyclase activity	1.34E-18	2.22E-15
PPYR1	-	47.31	GOTERM_BP_FAT	GO:0051339~regulation of lyase activity	2.17E-18	3.60E-15
SRRM1	serine/arginine repetitive matrix 1 [Source:HGNC Symbol;Acc:16638]	93.26	GOTERM_MF_FAT	GO:0003723~RNA binding	2.24E-18	2.99E-15
SSTR2	somatostatin receptor 2 [Source:HGNC Symbol;Acc:11331]	64.07	GOTERM_BP_FAT	GO:0007218~neuropeptide signaling pathway	5.06E-18	8.37E-15
BDKRB1	bradykinin receptor B1 [Source:HGNC Symbol;Acc:1029]	44.11	GOTERM_BP_FAT	GO:0042592~homeostatic process	1.24E-17	2.06E-14
ADRA1B	adrenoceptor alpha 1B [Source:HGNC Symbol;Acc:278]	39.61	GOTERM_CC_FAT	GO:0044459~plasma membrane part	1.49E-17	1.79E-14
HNRNPF	heterogeneous nuclear ribonucleoprotein F [Source:HGNC Symbol;Acc:5039]	77.90	INTERPRO	IPR001811:Small chemokine, interleukin-8-like	3.60E-17	5.00E-14
P2RY4	pyrimidinergic receptor P2Y, G-protein coupled, 4 [Source:HGNC Symbol;Acc:8542]	40.96	GOTERM_BP_FAT	GO:0044057~regulation of system process	2.96E-16	5.55E-13
PCBP2	poly(rC) binding protein 2 [Source:HGNC Symbol;Acc:8648]	57.76	SP_PIR_KEYWORDS	amidation	6.65E-16	8.44E-13
PROK1	prokineticin 1 [Source:HGNC Symbol;Acc:18454]	**19.52**	GOTERM_BP_FAT	GO:0022618~ribonucleoprotein complex assembly	8.83E-16	1.47E-12
NPFFR1	neuropeptide FF receptor 1 [Source:HGNC Symbol;Acc:17425]	60.22	GOTERM_BP_FAT	GO:0009611~response to wounding	2.37E-15	3.86E-12
OPRM1	opioid receptor, mu 1 [Source:HGNC Symbol;Acc:8156]	71.98	KEGG_PATHWAY	hsa04020:Calcium signaling pathway	2.66E-15	2.36E-12
YBX1	Y box binding protein 1 [Source:HGNC Symbol;Acc:8014]	57.56	GOTERM_BP_FAT	GO:0051336~regulation of hydrolase activity	4.80E-15	7.90E-12
TAC1	tachykinin, precursor 1 [Source:HGNC Symbol;Acc:11517]	31.52	GOTERM_CC_FAT	GO:0030532~small nuclear ribonucleoprotein complex	7.36E-15	8.79E-12
CCL16	chemokine (C-C motif) ligand 16 [Source:HGNC Symbol;Acc:10614]	57.32	GOTERM_BP_FAT	GO:0000245~spliceosome assembly	1.41E-14	2.33E-11
HTR2C	5-hydroxytryptamine (serotonin) receptor 2C, G protein-coupled [Source:HGNC Symbol;Acc:5295]	88.63	GOTERM_BP_FAT	GO:0007193~inhibition of adenylate cyclase activity by G-protein signaling	1.58E-14	2.61E-11
GPRC6A	G protein-coupled receptor, family C, group 6, member A [Source:HGNC Symbol;Acc:18510]	**1.27**	GOTERM_CC_FAT	GO:0005886~plasma membrane	1.69E-14	2.02E-11
CYSLTR1	cysteinyl leukotriene receptor 1 [Source:HGNC Symbol;Acc:17451]	56.74	UP_SEQ_FEATURE	domain:RRM 1	6.31E-14	9.61E-11
LPAR1	lysophosphatidic acid receptor 1 [Source:HGNC Symbol;Acc:3166]	72.31	UP_SEQ_FEATURE	domain:RRM 2	6.31E-14	9.61E-11
C3AR1	complement component 3a receptor 1 [Source:HGNC Symbol;Acc:1319]	81.04	GOTERM_MF_FAT	GO:0031202~RNA splicing factor activity, transesterification mechanism	2.37E-13	3.16E-10
TACR1	tachykinin receptor 1 [Source:HGNC Symbol;Acc:11526]	48.20	GOTERM_BP_FAT	GO:0006952~defense response	4.29E-13	7.10E-10
PPBP	pro-platelet basic protein (chemokine (C-X-C motif) ligand 7) [Source:HGNC Symbol;Acc:9240]	56.15	UP_SEQ_FEATURE	transmembrane region	5.58E-13	8.49E-10
ADRA2C	adrenoceptor alpha 2C [Source:HGNC Symbol;Acc:283]	60.68	SP_PIR_KEYWORDS	transmembrane	8.91E-13	1.13E-09
GALR3	galanin receptor 3 [Source:HGNC Symbol;Acc:4134]	74.48	INTERPRO	IPR018048:Small chemokine, C-X-C, conserved site	1.37E-12	1.91E-09
CASR	calcium-sensing receptor [Source:HGNC Symbol;Acc:1514]	**0.31**	GOTERM_BP_FAT	GO:0051046~regulation of secretion	1.86E-12	3.07E-09
HTR1B	5-hydroxytryptamine (serotonin) receptor 1B, G protein-coupled [Source:HGNC Symbol;Acc:5287]	46.05	GOTERM_MF_FAT	GO:0045028~purinergic nucleotide receptor activity, G-protein coupled	3.01E-12	4.02E-09
GALR1	galanin receptor 1 [Source:HGNC Symbol;Acc:4132]	73.18	GOTERM_MF_FAT	GO:0001608~nucleotide receptor activity, G-protein coupled	3.01E-12	4.02E-09
RXFP4	relaxin/insulin-like family peptide receptor 4 [Source:HGNC Symbol;Acc:14666]	37.90	SP_PIR_KEYWORDS	inflammatory response	3.30E-12	4.19E-09
HTR5A	5-hydroxytryptamine (serotonin) receptor 5A, G protein-coupled [Source:HGNC Symbol;Acc:5300]	41.56	GOTERM_BP_FAT	GO:0002027~regulation of heart rate	4.29E-12	7.10E-09
NPBWR2	neuropeptides B/W receptor 2 [Source:HGNC Symbol;Acc:4530]	56.72	INTERPRO	IPR001089:Small chemokine, C-X-C	4.85E-12	6.74E-09
GAL	galanin/GMAP prepropeptide [Source:HGNC Symbol;Acc:4114]	54.93	GOTERM_MF_FAT	GO:0005184~neuropeptide hormone activity	5.82E-12	7.78E-09
CXCL16	chemokine (C-X-C motif) ligand 16 [Source:HGNC Symbol;Acc:16642]	72.49	GOTERM_CC_FAT	GO:0030530~heterogeneous nuclear ribonucleoprotein complex	9.87E-12	1.18E-08
OXER1	oxoeicosanoid (OXE) receptor 1 [Source:HGNC Symbol;Acc:24884]	90.04	GOTERM_BP_FAT	GO:0045987~positive regulation of smooth muscle contraction	1.77E-11	2.93E-08
NFX1	nuclear transcription factor, X-box binding 1 [Source:HGNC Symbol;Acc:7803]	59.80	SP_PIR_KEYWORDS	neurotransmitter receptor	1.93E-11	2.45E-08
NTS	neurotensin [Source:HGNC Symbol;Acc:8038]	**19.29**	GOTERM_BP_FAT	GO:0006939~smooth muscle contraction	2.06E-11	3.41E-08
F2RL3	coagulation factor II (thrombin) receptor-like 3 [Source:HGNC Symbol;Acc:3540]	77.97	SP_PIR_KEYWORDS	hormone receptor	2.08E-11	2.64E-08
HNRNPA0	heterogeneous nuclear ribonucleoprotein A0 [Source:HGNC Symbol;Acc:5030]	32.00	INTERPRO	IPR002286:P2 purinoceptor	2.62E-11	3.63E-08
S1PR3	sphingosine-1-phosphate receptor 3 [Source:HGNC Symbol;Acc:3167]	48.91	GOTERM_BP_FAT	GO:0019229~regulation of vasoconstriction	2.99E-11	4.95E-08
CYSLTR2	cysteinyl leukotriene receptor 2 [Source:HGNC Symbol;Acc:18274]	76.12	GOTERM_BP_FAT	GO:0003073~regulation of systemic arterial blood pressure	4.29E-11	7.10E-08
HTR1E	5-hydroxytryptamine (serotonin) receptor 1E, G protein-coupled [Source:HGNC Symbol;Acc:5291]	77.19	GOTERM_MF_FAT	GO:0001614~purinergic nucleotide receptor activity	4.64E-11	6.19E-08
NPY1R	neuropeptide Y receptor Y1 [Source:HGNC Symbol;Acc:7956]	69.36	GOTERM_MF_FAT	GO:0016502~nucleotide receptor activity	4.64E-11	6.19E-08
NPSR1	neuropeptide S receptor 1 [Source:HGNC Symbol;Acc:23631]	66.46	GOTERM_CC_FAT	GO:0005665~DNA-directed RNA polymerase II, core complex	6.48E-11	7.78E-08
CCR8	chemokine (C-C motif) receptor 8 [Source:HGNC Symbol;Acc:1609]	40.42	PIR_SUPERFAMILY	PIRSF005435:LPAR1/S1PR1-like lysophospholipid receptor	6.94E-11	8.25E-08
GPR109A	-	95.82	SP_PIR_KEYWORDS	ribonucleoprotein	7.72E-11	9.79E-08
SSTR5	somatostatin receptor 5 [Source:HGNC Symbol;Acc:11334]	79.35	GOTERM_BP_FAT	GO:0051050~positive regulation of transport	1.01E-10	1.68E-07
ADRA2B	adrenoceptor alpha 2B [Source:HGNC Symbol;Acc:282]	62.31	GOTERM_BP_FAT	GO:0045933~positive regulation of muscle contraction	1.01E-10	1.68E-07
TRH	thyrotropin-releasing hormone [Source:HGNC Symbol;Acc:12298]	69.10	BIOCARTA	h_smPathway:Spliceosomal Assembly	1.03E-10	9.78E-08
MLN	motilin [Source:HGNC Symbol;Acc:7141]	54.12	GOTERM_BP_FAT	GO:0007268~synaptic transmission	2.44E-10	4.05E-07
SF3A1	splicing factor 3a, subunit 1, 120kDa [Source:HGNC Symbol;Acc:10765]	80.20	GOTERM_BP_FAT	GO:0045776~negative regulation of blood pressure	2.72E-10	4.51E-07
DRD4	dopamine receptor D4 [Source:HGNC Symbol;Acc:3025]	82.60	GOTERM_BP_FAT	GO:0045777~positive regulation of blood pressure	2.72E-10	4.51E-07
SSTR1	somatostatin receptor 1 [Source:HGNC Symbol;Acc:11330]	45.29	GOTERM_MF_FAT	GO:0004950~chemokine receptor activity	5.28E-10	7.06E-07
S1PR5	sphingosine-1-phosphate receptor 5 [Source:HGNC Symbol;Acc:14299]	30.36	GOTERM_BP_FAT	GO:0045860~positive regulation of protein kinase activity	6.19E-10	1.03E-06
CXCL11	chemokine (C-X-C motif) ligand 11 [Source:HGNC Symbol;Acc:10638]	51.14	KEGG_PATHWAY	hsa04062:Chemokine signaling pathway	6.59E-10	5.84E-07
GNAI2	guanine nucleotide binding protein (G protein), alpha inhibiting activity polypeptide 2 [Source:HGNC Symbol;Acc:4385]	**21.87**	SP_PIR_KEYWORDS	cytokine	8.10E-10	1.03E-06
OPRD1	opioid receptor, delta 1 [Source:HGNC Symbol;Acc:8153]	45.46	GOTERM_BP_FAT	GO:0051482~elevation of cytosolic calcium ion concentration during G-protein signaling, coupled to IP3 second messenger (phosphol	8.27E-10	1.37E-06
SMC1A	structural maintenance of chromosomes 1A [Source:HGNC Symbol;Acc:11111]	26.56	UP_SEQ_FEATURE	compositionally biased region:Arg/Ser-rich (RS domain)	1.09E-09	1.66E-06
NPY5R	neuropeptide Y receptor Y5 [Source:HGNC Symbol;Acc:7958]	46.45	GOTERM_MF_FAT	GO:0019956~chemokine binding	1.17E-09	1.56E-06
SFRS1	-	53.20	GOTERM_BP_FAT	GO:0033674~positive regulation of kinase activity	1.21E-09	2.01E-06
OXT	oxytocin/neurophysin I prepropeptide [Source:HGNC Symbol;Acc:8528]	41.18	GOTERM_BP_FAT	GO:0042127~regulation of cell proliferation	1.27E-09	2.10E-06
PNOC	prepronociceptin [Source:HGNC Symbol;Acc:9163]	43.59	SP_PIR_KEYWORDS	thiolester bond	1.29E-09	1.64E-06
CXCR2	chemokine (C-X-C motif) receptor 2 [Source:HGNC Symbol;Acc:6027]	52.41	GOTERM_BP_FAT	GO:0003044~regulation of systemic arterial blood pressure mediated by a chemical signal	1.46E-09	2.41E-06
FFAR2	free fatty acid receptor 2 [Source:HGNC Symbol;Acc:4501]	40.66	GOTERM_MF_FAT	GO:0001619~lysosphingolipid and lysophosphatidic acid receptor activity	1.48E-09	1.98E-06
CCL21	chemokine (C-C motif) ligand 21 [Source:HGNC Symbol;Acc:10620]	51.76	GOTERM_BP_FAT	GO:0019226~transmission of nerve impulse	1.68E-09	2.79E-06
CHRM2	cholinergic receptor, muscarinic 2 [Source:HGNC Symbol;Acc:1951]	79.26	GOTERM_BP_FAT	GO:0006940~regulation of smooth muscle contraction	2.37E-09	3.92E-06
GNRH2	gonadotropin-releasing hormone 2 [Source:HGNC Symbol;Acc:4420]	56.44	GOTERM_BP_FAT	GO:0051347~positive regulation of transferase activity	2.50E-09	4.13E-06
PTGER3	prostaglandin E receptor 3 (subtype EP3) [Source:HGNC Symbol;Acc:9595]	72.92	GOTERM_BP_FAT	GO:0007586~digestion	2.93E-09	4.86E-06
C3	complement component 3 [Source:HGNC Symbol;Acc:1318]	89.96	GOTERM_BP_FAT	GO:0022613~ribonucleoprotein complex biogenesis	3.09E-09	5.11E-06
DNAJC8	DnaJ (Hsp40) homolog, subfamily C, member 8 [Source:HGNC Symbol;Acc:15470]	52.90	SP_PIR_KEYWORDS	hormone	3.33E-09	4.22E-06
OPRK1	opioid receptor, kappa 1 [Source:HGNC Symbol;Acc:8154]	78.75	GOTERM_BP_FAT	GO:0008016~regulation of heart contraction	3.36E-09	5.57E-06
HNRNPUL1	heterogeneous nuclear ribonucleoprotein U-like 1 [Source:HGNC Symbol;Acc:17011]	**16.73**	SP_PIR_KEYWORDS	viral nucleoprotein	4.65E-09	5.90E-06
NPY	neuropeptide Y [Source:HGNC Symbol;Acc:7955]	51.54	GOTERM_BP_FAT	GO:0043279~response to alkaloid	7.04E-09	1.17E-05
CSTF3	cleavage stimulation factor, 3' pre-RNA, subunit 3, 77kDa [Source:HGNC Symbol;Acc:2485]	44.86	GOTERM_BP_FAT	GO:0051048~negative regulation of secretion	8.64E-09	1.43E-05
CPSF3	cleavage and polyadenylation specific factor 3, 73kDa [Source:HGNC Symbol;Acc:2326]	75.32	GOTERM_BP_FAT	GO:0031644~regulation of neurological system process	9.83E-09	1.63E-05
RNPS1	RNA binding protein S1, serine-rich domain [Source:HGNC Symbol;Acc:10080]	64.15	GOTERM_BP_FAT	GO:0006936~muscle contraction	9.83E-09	1.63E-05
CXCR1	chemokine (C-X-C motif) receptor 1 [Source:HGNC Symbol;Acc:6026]	44.28	GOTERM_BP_FAT	GO:0010460~positive regulation of heart rate	1.15E-08	1.90E-05
TXNL4A	thioredoxin-like 4A [Source:HGNC Symbol;Acc:30551]	41.73	GOTERM_BP_FAT	GO:0043406~positive regulation of MAP kinase activity	1.34E-08	2.23E-05
SF3B3	splicing factor 3b, subunit 3, 130kDa [Source:HGNC Symbol;Acc:10770]	87.36	GOTERM_BP_FAT	GO:0003018~vascular process in circulatory system	1.56E-08	2.58E-05
RBM5	RNA binding motif protein 5 [Source:HGNC Symbol;Acc:9902]	86.64	GOTERM_BP_FAT	GO:0051240~positive regulation of multicellular organismal process	1.79E-08	2.97E-05
SNRNP40	small nuclear ribonucleoprotein 40kDa (U5) [Source:HGNC Symbol;Acc:30857]	35.75	GOTERM_CC_FAT	GO:0000428~DNA-directed RNA polymerase complex	1.93E-08	2.31E-05
GPR44	-	64.42	GOTERM_CC_FAT	GO:0055029~nuclear DNA-directed RNA polymerase complex	1.93E-08	2.31E-05
SF3B1	splicing factor 3b, subunit 1, 155kDa [Source:HGNC Symbol;Acc:10768]	**16.32**	GOTERM_BP_FAT	GO:0060341~regulation of cellular localization	2.37E-08	3.93E-05
S1PR2	sphingosine-1-phosphate receptor 2 [Source:HGNC Symbol;Acc:3169]	50.70	GOTERM_BP_FAT	GO:0006379~mRNA cleavage	2.49E-08	4.12E-05
HNRNPL	heterogeneous nuclear ribonucleoprotein L [Source:HGNC Symbol;Acc:5045]	32.88	PIR_SUPERFAMILY	PIRSF002522:CXC chemokine	2.60E-08	3.09E-05
SF3A3	splicing factor 3a, subunit 3, 60kDa [Source:HGNC Symbol;Acc:10767]	**22.98**	GOTERM_CC_FAT	GO:0030880~RNA polymerase complex	2.74E-08	3.28E-05
P2RY12	purinergic receptor P2Y, G-protein coupled, 12 [Source:HGNC Symbol;Acc:18124]	65.92	GOTERM_BP_FAT	GO:0006368~RNA elongation from RNA polymerase II promoter	2.75E-08	4.55E-05
SF3B2	splicing factor 3b, subunit 2, 145kDa [Source:HGNC Symbol;Acc:10769]	**5.91**	GOTERM_BP_FAT	GO:0046717~acid secretion	3.00E-08	4.97E-05
PENK	proenkephalin [Source:HGNC Symbol;Acc:8831]	42.87	SP_PIR_KEYWORDS	dna-directed rna polymerase	3.79E-08	4.81E-05
NCBP2	nuclear cap binding protein subunit 2, 20kDa [Source:HGNC Symbol;Acc:7659]	51.57	GOTERM_BP_FAT	GO:0003012~muscle system process	4.02E-08	6.66E-05
GTF2F2	general transcription factor IIF, polypeptide 2, 30kDa [Source:HGNC Symbol;Acc:4653]	**19.95**	INTERPRO	IPR002473:Small chemokine, C-X-C/Interleukin 8	4.42E-08	6.14E-05
SFRS4	-	82.32	GOTERM_MF_FAT	GO:0005125~cytokine activity	4.52E-08	6.04E-05
SF3B5	splicing factor 3b, subunit 5, 10kDa [Source:HGNC Symbol;Acc:21083]	37.00	GOTERM_BP_FAT	GO:0014073~response to tropane	4.67E-08	7.74E-05
ADORA3	adenosine A3 receptor [Source:HGNC Symbol;Acc:268]	86.88	GOTERM_BP_FAT	GO:0042220~response to cocaine	4.67E-08	7.74E-05
DHX38	DEAH (Asp-Glu-Ala-His) box polypeptide 38 [Source:HGNC Symbol;Acc:17211]	36.52	GOTERM_BP_FAT	GO:0006354~RNA elongation	5.10E-08	8.44E-05
DDX23	DEAD (Asp-Glu-Ala-Asp) box polypeptide 23 [Source:HGNC Symbol;Acc:17347]	83.97	PIR_SUPERFAMILY	PIRSF038545:chemokine receptor	5.31E-08	6.31E-05
CLP1	cleavage and polyadenylation factor I subunit 1 [Source:HGNC Symbol;Acc:16999]	70.32	GOTERM_BP_FAT	GO:0001990~regulation of systemic arterial blood pressure by hormone	7.07E-08	1.17E-04
CCR7	chemokine (C-C motif) receptor 7 [Source:HGNC Symbol;Acc:1608]	70.34	GOTERM_BP_FAT	GO:0000187~activation of MAPK activity	7.19E-08	1.19E-04
CDC40	cell division cycle 40 [Source:HGNC Symbol;Acc:17350]	39.96	GOTERM_BP_FAT	GO:0050880~regulation of blood vessel size	7.51E-08	1.24E-04
SFRS3	-	52.64	GOTERM_BP_FAT	GO:0035150~regulation of tube size	7.51E-08	1.24E-04
SFRS9	-	57.65	SP_PIR_KEYWORDS	RNA binding	9.38E-08	1.19E-04
FUS	fused in sarcoma [Source:HGNC Symbol;Acc:4010]	**5.95**	INTERPRO	IPR000827:Small chemokine, C-C group, conserved site	9.68E-08	1.34E-04
NCBP1	nuclear cap binding protein subunit 1, 80kDa [Source:HGNC Symbol;Acc:7658]	**10.70**	COG_ONTOLOGY	RNA processing and modification	1.08E-07	3.40E-05
PABPN1	poly(A) binding protein, nuclear 1 [Source:HGNC Symbol;Acc:8565]	43.77	INTERPRO	IPR002231:5-Hydroxytryptamine receptor	1.24E-07	1.72E-04
FFAR1	free fatty acid receptor 1 [Source:HGNC Symbol;Acc:4498]	45.06	UP_SEQ_FEATURE	domain:RRM	1.28E-07	1.94E-04
POLR2L	polymerase (RNA) II (DNA directed) polypeptide L, 7.6kDa [Source:HGNC Symbol;Acc:9199]	41.75	GOTERM_BP_FAT	GO:0045823~positive regulation of heart contraction	1.54E-07	2.54E-04
SF3B4	splicing factor 3b, subunit 4, 49kDa [Source:HGNC Symbol;Acc:10771]	60.08	GOTERM_BP_FAT	GO:0006376~mRNA splice site selection	1.54E-07	2.54E-04
PRPF8	pre-mRNA processing factor 8 [Source:HGNC Symbol;Acc:17340]	**18.52**	GOTERM_BP_FAT	GO:0003078~regulation of natriuresis	1.60E-07	2.65E-04
HNRNPU	heterogeneous nuclear ribonucleoprotein U (scaffold attachment factor A) [Source:HGNC Symbol;Acc:5048]	74.42	GOTERM_BP_FAT	GO:0006937~regulation of muscle contraction	1.62E-07	2.68E-04
PRPF6	pre-mRNA processing factor 6 [Source:HGNC Symbol;Acc:15860]	91.81	BBID	109.Chemokine_families	1.80E-07	1.12E-04
NUDT21	nudix (nucleoside diphosphate linked moiety X)-type motif 21 [Source:HGNC Symbol;Acc:13870]	42.09	GOTERM_BP_FAT	GO:0051952~regulation of amine transport	1.99E-07	3.29E-04
SF3A2	splicing factor 3a, subunit 2, 66kDa [Source:HGNC Symbol;Acc:10766]	39.22	GOTERM_BP_FAT	GO:0050886~endocrine process	2.12E-07	3.52E-04
PHF5A	PHD finger protein 5A [Source:HGNC Symbol;Acc:18000]	47.74	GOTERM_BP_FAT	GO:0051969~regulation of transmission of nerve impulse	2.30E-07	3.81E-04
SF4	-	93.61	GOTERM_BP_FAT	GO:0051047~positive regulation of secretion	2.43E-07	4.02E-04
UPF3B	UPF3 regulator of nonsense transcripts homolog B (yeast) [Source:HGNC Symbol;Acc:20439]	68.24	GOTERM_BP_FAT	GO:0040012~regulation of locomotion	2.82E-07	4.68E-04
SNRPA	small nuclear ribonucleoprotein polypeptide A [Source:HGNC Symbol;Acc:11151]	59.15	INTERPRO	IPR004061:Sphingosine 1-phosphate receptor	3.23E-07	4.49E-04
U2AF1	U2 small nuclear RNA auxiliary factor 1 [Source:HGNC Symbol;Acc:12453]	43.56	INTERPRO	IPR000586:Somatostatin receptor	3.23E-07	4.49E-04
CPSF2	cleavage and polyadenylation specific factor 2, 100kDa [Source:HGNC Symbol;Acc:2325]	47.13	GOTERM_BP_FAT	GO:0051705~behavioral interaction between organisms	3.26E-07	5.39E-04

CCL19	chemokine (C-C motif) ligand 19 [Source:HGNC Symbol;Acc:10617]	49.14
CXCL1	chemokine (C-X-C motif) ligand 1 (melanoma growth stimulating activity, alpha) [Source:HGNC Symbol;Acc:4602]	65.36
SST	somatostatin [Source:HGNC Symbol;Acc:11329]	53.95
CSTF1	cleavage stimulation factor, 3' pre-RNA, subunit 1, 50kDa [Source:HGNC Symbol;Acc:2483]	46.48
GPER	-	51.91
POLR2C	polymerase (RNA) II (DNA directed) polypeptide C, 33kDa [Source:HGNC Symbol;Acc:9189]	**19.73**
SF3B14	-	49.86
CXCR5	chemokine (C-X-C motif) receptor 5 [Source:HGNC Symbol;Acc:1060]	77.32
SNRPA1	small nuclear ribonucleoprotein polypeptide A' [Source:HGNC Symbol;Acc:11152]	58.36
CCL27	chemokine (C-C motif) ligand 27 [Source:HGNC Symbol;Acc:10626]	52.32
SNRNP70	small nuclear ribonucleoprotein 70kDa (U1) [Source:HGNC Symbol;Acc:11150]	61.76
HNRNPK	heterogeneous nuclear ribonucleoprotein K [Source:HGNC Symbol;Acc:5044]	43.99
SFRS6	-	69.20
SNRPB2	small nuclear ribonucleoprotein polypeptide B [Source:HGNC Symbol;Acc:11155]	56.06
SFRS5	-	69.21
HNRNPA2B1	heterogeneous nuclear ribonucleoprotein A2/B1 [Source:HGNC Symbol;Acc:5033]	43.61
PDYN	prodynorphin [Source:HGNC Symbol;Acc:8820]	**14.99**
POLR2G	polymerase (RNA) II (DNA directed) polypeptide G [Source:HGNC Symbol;Acc:9194]	37.83
CXCL13	chemokine (C-X-C motif) ligand 13 [Source:HGNC Symbol;Acc:10639]	51.72
POLR2H	polymerase (RNA) II (DNA directed) polypeptide H [Source:HGNC Symbol;Acc:9195]	27.47
SNRPF	small nuclear ribonucleoprotein polypeptide F [Source:HGNC Symbol;Acc:11162]	48.05
CHRM3	cholinergic receptor, muscarinic 3 [Source:HGNC Symbol;Acc:1952]	97.47
P2RY1	purinergic receptor P2Y, G-protein coupled, 1 [Source:HGNC Symbol;Acc:8539]	66.74
CCKAR	cholecystokinin A receptor [Source:HGNC Symbol;Acc:1570]	68.97
KISS1R	KISS1 receptor [Source:HGNC Symbol;Acc:4510]	65.15
GNRH1	gonadotropin-releasing hormone 1 (luteinizing-releasing hormone) [Source:HGNC Symbol;Acc:4419]	51.05
NMBR	neuromedin B receptor [Source:HGNC Symbol;Acc:7843]	53.80
HTR2B	5-hydroxytryptamine (serotonin) receptor 2B, G protein-coupled [Source:HGNC Symbol;Acc:5294]	80.89
GPR132	G protein-coupled receptor 132 [Source:HGNC Symbol;Acc:17482]	53.09
SNRPD3	small nuclear ribonucleoprotein D3 polypeptide 18kDa [Source:HGNC Symbol;Acc:11160]	49.83
U2AF2	U2 small nuclear RNA auxiliary factor 2 [Source:HGNC Symbol;Acc:23156]	74.81
AGTR2	angiotensin II receptor, type 2 [Source:HGNC Symbol;Acc:338]	68.96
PTAFR	platelet-activating factor receptor [Source:HGNC Symbol;Acc:9582]	69.27
POLR2D	polymerase (RNA) II (DNA directed) polypeptide D [Source:HGNC Symbol;Acc:9191]	27.13
KISS1	KiSS-1 metastasis-suppressor [Source:HGNC Symbol;Acc:6341]	41.90
LPAR3	lysophosphatidic acid receptor 3 [Source:HGNC Symbol;Acc:14298]	74.39
CCKBR	cholecystokinin B receptor [Source:HGNC Symbol;Acc:1571]	82.20
CSTF2	cleavage stimulation factor, 3' pre-RNA, subunit 2, 64kDa [Source:HGNC Symbol;Acc:2484]	66.28
TACR2	tachykinin receptor 2 [Source:HGNC Symbol;Acc:11527]	45.11
LTB4R	leukotriene B4 receptor [Source:HGNC Symbol;Acc:6713]	59.41
POLR2A	polymerase (RNA) II (DNA directed) polypeptide A, 220kDa [Source:HGNC Symbol;Acc:9187]	98.77
PCF11	PCF11 cleavage and polyadenylation factor subunit [Source:HGNC Symbol;Acc:30097]	88.96
F2R	coagulation factor II (thrombin) receptor [Source:HGNC Symbol;Acc:3537]	71.67
P2RY14	purinergic receptor P2Y, G-protein coupled, 14 [Source:HGNC Symbol;Acc:16442]	41.25
GTF2F1	general transcription factor IIF, polypeptide 1, 74kDa [Source:HGNC Symbol;Acc:4652]	91.65
NHP2L1	NHP2 non-histone chromosome protein 2-like 1 (S. cerevisiae) [Source:HGNC Symbol;Acc:7819]	54.78
PYY	peptide YY [Source:HGNC Symbol;Acc:9748]	52.69
CXCL10	chemokine (C-X-C motif) ligand 10 [Source:HGNC Symbol;Acc:10637]	50.54
HCRT	hypocretin (orexin) neuropeptide precursor [Source:HGNC Symbol;Acc:4847]	41.04

GOTERM_MF_FAT	GO:0004435~phosphoinositide phospholipase C activity	3.41E-07	4.55E-04
GOTERM_BP_FAT	GO:0044062~regulation of excretion	3.54E-07	5.86E-04
SP_PIR_KEYWORDS	Secreted	3.62E-07	4.59E-04
GOTERM_BP_FAT	GO:0031124~mRNA 3'-end processing	3.96E-07	6.56E-04
GOTERM_BP_FAT	GO:0014072~response to isoquinoline alkaloid	3.96E-07	6.56E-04
GOTERM_BP_FAT	GO:0043278~response to morphine	3.96E-07	6.56E-04
GOTERM_BP_FAT	GO:0045859~regulation of protein kinase activity	4.06E-07	6.73E-04
GOTERM_BP_FAT	GO:0032844~regulation of homeostatic process	4.12E-07	6.82E-04
GOTERM_BP_FAT	GO:0032103~positive regulation of response to external stimulus	4.81E-07	7.97E-04
GOTERM_BP_FAT	GO:0050804~regulation of synaptic transmission	5.26E-07	8.71E-04
UP_SEQ_FEATURE	compositionally biased region:Gly-rich (hinge region)	5.80E-07	8.82E-04
GOTERM_BP_FAT	GO:0042310~vasoconstriction	6.02E-07	9.97E-04
GOTERM_BP_FAT	GO:0032101~regulation of response to external stimulus	6.39E-07	1.06E-03
GOTERM_MF_FAT	GO:0004994~somatostatin receptor activity	6.83E-07	9.12E-04
GOTERM_BP_FAT	GO:0014821~phasic smooth muscle contraction	6.96E-07	1.15E-03
GOTERM_BP_FAT	GO:0043549~regulation of kinase activity	7.40E-07	1.23E-03
GOTERM_BP_FAT	GO:0000165~MAPKKK cascade	8.12E-07	1.34E-03
GOTERM_BP_FAT	GO:0043405~regulation of MAP kinase activity	8.18E-07	1.36E-03
GOTERM_BP_FAT	GO:0014070~response to organic cyclic substance	8.25E-07	1.37E-03
GOTERM_BP_FAT	GO:0006367~transcription initiation from RNA polymerase II promoter	8.61E-07	1.43E-03
GOTERM_BP_FAT	GO:0003014~renal system process	9.42E-07	1.56E-03
SP_PIR_KEYWORDS	membrane	1.01E-06	1.28E-03
GOTERM_BP_FAT	GO:0032309~icosanoid secretion	1.25E-06	2.08E-03
GOTERM_BP_FAT	GO:0007213~muscarinic acetylcholine receptor signaling pathway	1.25E-06	2.08E-03
GOTERM_BP_FAT	GO:0050482~arachidonic acid secretion	1.25E-06	2.08E-03
GOTERM_BP_FAT	GO:0006378~mRNA polyadenylation	1.25E-06	2.08E-03
SP_PIR_KEYWORDS	pre-mRNA splicing	1.30E-06	1.65E-03
GOTERM_MF_FAT	GO:0003899~DNA-directed RNA polymerase activity	1.45E-06	1.94E-03
GOTERM_MF_FAT	GO:0034062~RNA polymerase activity	1.45E-06	1.94E-03
GOTERM_BP_FAT	GO:0051338~regulation of transferase activity	1.50E-06	2.49E-03
GOTERM_MF_FAT	GO:0004629~phospholipase C activity	1.69E-06	2.25E-03
GOTERM_MF_FAT	GO:0004936~alpha-adrenergic receptor activity	2.02E-06	2.69E-03
INTERPRO	IPR000826:Formyl/methionyl peptide receptor	2.21E-06	3.07E-03
PIR_SUPERFAMILY	PIRSF038568:somatostatin receptor	2.44E-06	2.90E-03
PIR_SUPERFAMILY	PIRSF501018:sphingosine 1-phosphate receptor 1-5	2.44E-06	2.90E-03
GOTERM_BP_FAT	GO:0008284~positive regulation of cell proliferation	2.72E-06	4.51E-03
GOTERM_BP_FAT	GO:0043086~negative regulation of catalytic activity	2.82E-06	4.67E-03
GOTERM_BP_FAT	GO:0051051~negative regulation of transport	2.87E-06	4.75E-03
SP_PIR_KEYWORDS	amidated carboxyl end	2.88E-06	3.66E-03
GOTERM_BP_FAT	GO:0050795~regulation of behavior	2.99E-06	4.95E-03
GOTERM_MF_FAT	GO:0004993~serotonin receptor activity	3.00E-06	4.01E-03
GOTERM_BP_FAT	GO:0043269~regulation of ion transport	4.20E-06	6.96E-03
GOTERM_BP_FAT	GO:0019220~regulation of phosphate metabolic process	4.35E-06	7.21E-03
GOTERM_BP_FAT	GO:0051174~regulation of phosphorus metabolic process	4.35E-06	7.21E-03
INTERPRO	IPR002233:Adrenergic receptor	4.36E-06	6.05E-03
GOTERM_BP_FAT	GO:0042755~eating behavior	4.44E-06	7.35E-03
GOTERM_MF_FAT	GO:0004982~N-formyl peptide receptor activity	4.63E-06	6.19E-03
GOTERM_CC_FAT	GO:0016591~DNA-directed RNA polymerase II, holoenzyme	4.86E-06	5.83E-03
KEGG_PATHWAY	hsa03020:RNA polymerase	5.15E-06	4.56E-03
GOTERM_BP_FAT	GO:0043631~RNA polyadenylation	5.18E-06	8.57E-03
GOTERM_BP_FAT	GO:0051954~positive regulation of amine transport	5.18E-06	8.57E-03
GOTERM_BP_FAT	GO:0006352~transcription initiation	5.54E-06	9.18E-03
GOTERM_BP_FAT	GO:0046883~regulation of hormone secretion	5.84E-06	9.68E-03
GOTERM_BP_FAT	GO:0042325~regulation of phosphorylation	6.63E-06	1.10E-02
GOTERM_BP_FAT	GO:0019722~calcium-mediated signaling	7.03E-06	1.16E-02
GOTERM_BP_FAT	GO:0030334~regulation of cell migration	7.05E-06	1.17E-02
GOTERM_BP_FAT	GO:0006955~immune response	7.06E-06	1.17E-02
GOTERM_BP_FAT	GO:0046903~secretion	8.97E-06	1.48E-02
GOTERM_MF_FAT	GO:0016494~C-X-C chemokine receptor activity	9.13E-06	1.22E-02
GOTERM_MF_FAT	GO:0043176~amine binding	9.28E-06	1.24E-02
KEGG_PATHWAY	hsa04060:Cytokine-cytokine receptor interaction	1.00E-05	8.89E-03
GOTERM_BP_FAT	GO:0030335~positive regulation of cell migration	1.04E-05	1.73E-02
SP_PIR_KEYWORDS	phosphoprotein	1.07E-05	1.36E-02
UP_SEQ_FEATURE	propeptide:Removed for receptor activation	1.12E-05	1.70E-02
SP_PIR_KEYWORDS	vasoactive	1.18E-05	1.49E-02
GOTERM_BP_FAT	GO:0032846~positive regulation of homeostatic process	1.21E-05	2.00E-02
GOTERM_BP_FAT	GO:0007618~mating	1.22E-05	2.02E-02
GOTERM_BP_FAT	GO:0031123~RNA 3'-end processing	1.22E-05	2.02E-02
GOTERM_BP_FAT	GO:0007611~learning or memory	1.28E-05	2.12E-02
GOTERM_MF_FAT	GO:0008144~drug binding	1.31E-05	1.75E-02
GOTERM_BP_FAT	GO:0032228~regulation of synaptic transmission, GABAergic	1.52E-05	2.52E-02
GOTERM_CC_FAT	GO:0005654~nucleoplasm	1.55E-05	1.86E-02
GOTERM_BP_FAT	GO:0007243~protein kinase cascade	1.62E-05	2.68E-02
GOTERM_MF_FAT	GO:0051378~serotonin binding	1.62E-05	2.16E-02
GOTERM_MF_FAT	GO:0004985~opioid receptor activity	1.62E-05	2.16E-02
GOTERM_MF_FAT	GO:0019958~C-X-C chemokine binding	1.62E-05	2.16E-02
INTERPRO	IPR003912:Protease-activated receptor	1.63E-05	2.26E-02
GOTERM_BP_FAT	GO:0014829~vascular smooth muscle contraction	1.78E-05	2.95E-02
SP_PIR_KEYWORDS	inflammation	2.26E-05	2.86E-02
GOTERM_BP_FAT	GO:0065003~macromolecular complex assembly	2.36E-05	3.90E-02
GOTERM_BP_FAT	GO:0040017~positive regulation of locomotion	2.46E-05	4.07E-02
GOTERM_BP_FAT	GO:0051272~positive regulation of cell motion	2.46E-05	4.07E-02
GOTERM_MF_FAT	GO:0004935~adrenoceptor activity	2.65E-05	3.55E-02
GOTERM_MF_FAT	GO:0004954~prostanoid receptor activity	2.65E-05	3.55E-02
GOTERM_MF_FAT	GO:0004953~icosanoid receptor activity	2.65E-05	3.55E-02
GOTERM_BP_FAT	GO:0001975~response to amphetamine	2.77E-05	4.59E-02
GOTERM_BP_FAT	GO:0050433~regulation of catecholamine secretion	2.77E-05	4.59E-02
GOTERM_BP_FAT	GO:0035176~social behavior	2.77E-05	4.59E-02

Cluster 3 - intolerant	Gene Symbol	Gene description	EvoTol %ile
Mann-Whitney U test *P* = 7.5×10^{-8}	*NR5A1*	nuclear receptor subfamily 5, group A, member 1 [Source:HGNC Symbol;Acc:7983]	**7.56**
	HNF4A	hepatocyte nuclear factor 4, alpha [Source:HGNC Symbol;Acc:5024]	**6.59**
	EIF5	eukaryotic translation initiation factor 5 [Source:HGNC Symbol;Acc:3299]	**15.90**
	RPS26	ribosomal protein S26 [Source:HGNC Symbol;Acc:10414]	27.46
	RPL21	ribosomal protein L21 [Source:HGNC Symbol;Acc:10313]	52.11
	NR2E1	nuclear receptor subfamily 2, group E, member 1 [Source:HGNC Symbol;Acc:7973]	32.10
	RORB	RAR-related orphan receptor B [Source:HGNC Symbol;Acc:10259]	**15.09**
	NR2C1	nuclear receptor subfamily 2, group C, member 1 [Source:HGNC Symbol;Acc:7971]	**9.34**
	NR4A1	nuclear receptor subfamily 4, group A, member 1 [Source:HGNC Symbol;Acc:7980]	**6.25**
	NR4A2	nuclear receptor subfamily 4, group A, member 2 [Source:HGNC Symbol;Acc:7981]	**20.39**
	NR2C2	nuclear receptor subfamily 2, group C, member 2 [Source:HGNC Symbol;Acc:7972]	**8.60**
	ESR2	estrogen receptor 2 (ER beta) [Source:HGNC Symbol;Acc:3468]	**2.41**
	EIF2S1	eukaryotic translation initiation factor 2, subunit 1 alpha, 35kDa [Source:HGNC Symbol;Acc:3265]	**22.72**
	RARB	retinoic acid receptor, beta [Source:HGNC Symbol;Acc:9865]	**17.00**
	PPARG	peroxisome proliferator-activated receptor gamma [Source:HGNC Symbol;Acc:9236]	**13.68**
	NR1H2	nuclear receptor subfamily 1, group H, member 2 [Source:HGNC Symbol;Acc:7965]	-
	NR2F1	nuclear receptor subfamily 2, group F, member 1 [Source:HGNC Symbol;Acc:7975]	31.62
	HNF4G	hepatocyte nuclear factor 4, gamma [Source:HGNC Symbol;Acc:5026]	**13.45**
	NR2F6	nuclear receptor subfamily 2, group F, member 6 [Source:HGNC Symbol;Acc:7977]	**18.51**
	EIF4H	eukaryotic translation initiation factor 4H [Source:HGNC Symbol;Acc:12741]	58.73
	NR2C2AP	nuclear receptor 2C2-associated protein [Source:HGNC Symbol;Acc:30763]	33.69
	RXRA	retinoid X receptor, alpha [Source:HGNC Symbol;Acc:10477]	**15.18**
	THRA	thyroid hormone receptor, alpha [Source:HGNC Symbol;Acc:11796]	**7.21**
	THRB	thyroid hormone receptor, beta [Source:HGNC Symbol;Acc:11799]	**4.09**
	EIF2S2	eukaryotic translation initiation factor 2, subunit 2 beta, 38kDa [Source:HGNC Symbol;Acc:3266]	57.96
	ESRRG	estrogen-related receptor gamma [Source:HGNC Symbol;Acc:3474]	**14.11**
	RXRG	retinoid X receptor, gamma [Source:HGNC Symbol;Acc:10479]	**21.71**
	PPARA	peroxisome proliferator-activated receptor alpha [Source:HGNC Symbol;Acc:9232]	**11.31**
	PPARD	peroxisome proliferator-activated receptor delta [Source:HGNC Symbol;Acc:9235]	**4.23**
	EIF3G	eukaryotic translation initiation factor 3, subunit G [Source:HGNC Symbol;Acc:3274]	80.43
	EIF3I	eukaryotic translation initiation factor 3, subunit I [Source:HGNC Symbol;Acc:3272]	35.65
	RORC	RAR-related orphan receptor C [Source:HGNC Symbol;Acc:10260]	**1.54**
	EIF3D	eukaryotic translation initiation factor 3, subunit D [Source:HGNC Symbol;Acc:3278]	**13.46**
	EIF3J	eukaryotic translation initiation factor 3, subunit J [Source:HGNC Symbol;Acc:3270]	54.29
	NR1D2	nuclear receptor subfamily 1, group D, member 2 [Source:HGNC Symbol;Acc:7963]	**4.89**
	NR0B2	nuclear receptor subfamily 0, group B, member 2 [Source:HGNC Symbol;Acc:7961]	**7.87**
	EIF3B	eukaryotic translation initiation factor 3, subunit B [Source:HGNC Symbol;Acc:3280]	89.63
	EIF4B	eukaryotic translation initiation factor 4B [Source:HGNC Symbol;Acc:3285]	30.37
	VDR	vitamin D (1,25- dihydroxyvitamin D3) receptor [Source:HGNC Symbol;Acc:12679]	**2.79**
	EIF3F	eukaryotic translation initiation factor 3, subunit F [Source:HGNC Symbol;Acc:3275]	75.27
	RORA	RAR-related orphan receptor A [Source:HGNC Symbol;Acc:10258]	**1.60**
	RARA	retinoic acid receptor, alpha [Source:HGNC Symbol;Acc:9864]	**17.93**
	PAK2	p21 protein (Cdc42/Rac)-activated kinase 2 [Source:HGNC Symbol;Acc:8591]	40.87
	EIF3K	eukaryotic translation initiation factor 3, subunit K [Source:HGNC Symbol;Acc:24656]	**16.63**
	EIF4G1	eukaryotic translation initiation factor 4 gamma, 1 [Source:HGNC Symbol;Acc:3296]	96.93
	NR1D1	nuclear receptor subfamily 1, group D, member 1 [Source:HGNC Symbol;Acc:7962]	**0.79**
	EIF4E	eukaryotic translation initiation factor 4E [Source:HGNC Symbol;Acc:3287]	53.55
	RPL36AL	ribosomal protein L36a-like [Source:HGNC Symbol;Acc:10346]	52.66
	ESRRA	estrogen-related receptor alpha [Source:HGNC Symbol;Acc:3471]	-
	EIF3E	eukaryotic translation initiation factor 3, subunit E [Source:HGNC Symbol;Acc:3277]	66.24

Enrichment Type	Enrichment Term	P-value of enrichment	FDR
INTERPRO	IPR001723:Steroid hormone receptor	1.75E-66	1.76E-63
INTERPRO	IPR008946:Nuclear hormone receptor, ligand-binding	4.41E-66	4.45E-63
INTERPRO	IPR000536:Nuclear hormone receptor, ligand-binding, core	4.41E-66	4.45E-63
UP_SEQ_FEATURE	zinc finger region:NR C4-type	1.74E-65	2.03E-62
UP_SEQ_FEATURE	DNA-binding region:Nuclear receptor	1.74E-65	2.03E-62
INTERPRO	IPR001628:Zinc finger, nuclear hormone receptor-type	8.96E-64	9.04E-61
GOTERM_MF_FAT	GO:0003707~steroid hormone receptor activity	1.49E-62	1.66E-59
SMART	SM00430:HOLI	3.97E-62	2.56E-59
SMART	SM00399:ZnF_C4	7.31E-60	4.70E-57
GOTERM_MF_FAT	GO:0004879~ligand-dependent nuclear receptor activity	1.56E-59	1.74E-56
INTERPRO	IPR013088:Zinc finger, NHR/GATA-type	3.36E-53	3.39E-50
UP_SEQ_FEATURE	region of interest:Ligand-binding	5.46E-41	6.36E-38
SP_PIR_KEYWORDS	Initiation factor	1.03E-25	1.05E-22
GOTERM_MF_FAT	GO:0043565~sequence-specific DNA binding	2.08E-25	2.32E-22
SP_PIR_KEYWORDS	protein biosynthesis	1.58E-22	1.62E-19
GOTERM_MF_FAT	GO:0003743~translation initiation factor activity	2.18E-22	2.43E-19
GOTERM_MF_FAT	GO:0003700~transcription factor activity	4.57E-21	5.10E-18
SP_PIR_KEYWORDS	receptor	1.45E-19	1.48E-16
GOTERM_MF_FAT	GO:0008135~translation factor activity, nucleic acid binding	2.86E-19	3.19E-16
INTERPRO	IPR000324:Vitamin D receptor	5.57E-19	5.62E-16
SP_PIR_KEYWORDS	zinc-finger	1.36E-18	1.39E-15
UP_SEQ_FEATURE	region of interest:Modulating	3.03E-18	3.53E-15
SP_PIR_KEYWORDS	zinc finger	6.12E-17	1.11E-13
SP_PIR_KEYWORDS	transcription regulation	1.18E-16	1.11E-13
SP_PIR_KEYWORDS	dna-binding	1.73E-16	2.22E-13
SP_PIR_KEYWORDS	Transcription	2.13E-16	2.22E-13
GOTERM_BP_FAT	GO:0006413~translational initiation	3.35E-16	5.11E-13
GOTERM_BP_FAT	GO:0006412~translation	5.49E-16	8.44E-13
GOTERM_CC_FAT	GO:0005852~eukaryotic translation initiation factor 3 complex	6.81E-16	6.33E-13
GOTERM_MF_FAT	GO:0030528~transcription regulator activity	8.59E-16	9.88E-13
SP_PIR_KEYWORDS	zinc	1.05E-15	1.02E-12
GOTERM_BP_FAT	GO:0051252~regulation of RNA metabolic process	3.35E-15	5.06E-12
GOTERM_BP_FAT	GO:0006357~regulation of transcription from RNA polymerase II promoter	9.27E-15	1.42E-11
GOTERM_BP_FAT	GO:0006355~regulation of transcription, DNA-dependent	1.91E-14	2.90E-11
UP_SEQ_FEATURE	region of interest:Hinge	7.71E-14	8.97E-11
INTERPRO	IPR000003:Retinoid X receptor	8.61E-14	8.69E-11
GOTERM_CC_FAT	GO:0005829~cytosol	5.20E-13	4.94E-10
GOTERM_MF_FAT	GO:0016563~transcription activator activity	6.55E-13	7.29E-10
PIR_SUPERFAMILY	PIRSF002534:retinoic X receptor-like nuclear receptor	1.01E-12	8.77E-10
GOTERM_BP_FAT	GO:0006350~transcription	1.63E-12	2.48E-09
SP_PIR_KEYWORDS	metal-binding	3.09E-12	3.16E-09
GOTERM_BP_FAT	GO:0045893~positive regulation of transcription, DNA-dependent	3.55E-12	5.39E-09
GOTERM_BP_FAT	GO:0051254~positive regulation of RNA metabolic process	4.03E-12	6.12E-09
SP_PIR_KEYWORDS	nucleus	1.90E-11	1.94E-08
GOTERM_BP_FAT	GO:0045944~positive regulation of transcription from RNA polymerase II promoter	2.21E-11	3.35E-08
SP_PIR_KEYWORDS	DNA binding	3.94E-11	4.02E-08
GOTERM_BP_FAT	GO:0045941~positive regulation of transcription	4.41E-11	6.70E-08
GOTERM_MF_FAT	GO:0008270~zinc ion binding	5.29E-11	5.90E-08
GOTERM_MF_FAT	GO:0003677~DNA binding	6.58E-11	7.33E-08
GOTERM_BP_FAT	GO:0010628~positive regulation of gene expression	6.86E-11	1.04E-07
GOTERM_BP_FAT	GO:0006446~regulation of translational initiation	9.21E-11	1.40E-07
GOTERM_BP_FAT	GO:0045935~positive regulation of nucleobase, nucleoside, nucleotide and nucleic acid metabolic process	1.97E-10	3.00E-07
GOTERM_BP_FAT	GO:0051173~positive regulation of nitrogen compound metabolic process	3.14E-10	4.77E-07
GOTERM_BP_FAT	GO:0045449~regulation of transcription	3.67E-10	5.57E-07
GOTERM_BP_FAT	GO:0010557~positive regulation of macromolecule biosynthetic process	3.94E-10	5.98E-07
GOTERM_BP_FAT	GO:0031328~positive regulation of cellular biosynthetic process	7.76E-10	1.18E-06
GOTERM_BP_FAT	GO:0009891~positive regulation of biosynthetic process	9.58E-10	1.46E-06
GOTERM_MF_FAT	GO:0005496~steroid binding	3.17E-09	3.53E-06
GOTERM_MF_FAT	GO:0046914~transition metal ion binding	5.36E-09	5.97E-06
GOTERM_BP_FAT	GO:0045934~negative regulation of nucleobase, nucleoside, nucleotide and nucleic acid metabolic process	1.61E-08	2.44E-05
GOTERM_BP_FAT	GO:0010605~negative regulation of macromolecule metabolic process	1.84E-08	2.80E-05
GOTERM_BP_FAT	GO:0051172~negative regulation of nitrogen compound metabolic process	1.89E-08	2.87E-05
GOTERM_BP_FAT	GO:0010604~positive regulation of macromolecule metabolic process	1.94E-08	2.94E-05
GOTERM_BP_FAT	GO:0032583~regulation of gene-specific transcription	2.03E-08	3.09E-05
GOTERM_BP_FAT	GO:0006417~regulation of translation	2.42E-08	3.67E-05
GOTERM_MF_FAT	GO:0008134~transcription factor binding	2.67E-08	2.97E-05
GOTERM_BP_FAT	GO:0010558~negative regulation of macromolecule biosynthetic process	3.52E-08	5.35E-05
GOTERM_BP_FAT	GO:0016481~negative regulation of transcription	4.63E-08	7.03E-05

GOTERM_BP_FAT	GO:0031327~negative regulation of cellular biosynthetic process	4.74E-08	7.20E-05
GOTERM_MF_FAT	GO:0008289~lipid binding	5.82E-08	6.49E-05
GOTERM_BP_FAT	GO:0009890~negative regulation of biosynthetic process	6.07E-08	9.23E-05
GOTERM_BP_FAT	GO:0010629~negative regulation of gene expression	1.29E-07	1.95E-04
GOTERM_BP_FAT	GO:0000122~negative regulation of transcription from RNA polymerase II promoter	3.29E-07	5.00E-04
GOTERM_BP_FAT	GO:0051253~negative regulation of RNA metabolic process	4.40E-07	6.68E-04
SP_PIR_KEYWORDS	transcription factor	6.41E-07	6.55E-04
GOTERM_BP_FAT	GO:0010608~posttranscriptional regulation of gene expression	6.77E-07	1.03E-03
GOTERM_CC_FAT	GO:0016281~eukaryotic translation initiation factor 4F complex	7.84E-07	7.45E-04
GOTERM_BP_FAT	GO:0010551~regulation of specific transcription from RNA polymerase II promoter	9.29E-07	1.41E-03
GOTERM_BP_FAT	GO:0045892~negative regulation of transcription, DNA-dependent	3.71E-06	5.63E-03
GOTERM_BP_FAT	GO:0032268~regulation of cellular protein metabolic process	5.01E-06	7.61E-03
SP_PIR_KEYWORDS	phosphoprotein	6.96E-06	7.11E-03
GOTERM_MF_FAT	GO:0003708~retinoic acid receptor activity	1.02E-05	1.14E-02
BIOCARTA	h_pparPathway:Basic mechanism of action of PPARa, PPARb(d) and PPARg and effects on gene expression	1.63E-05	1.39E-02
INTERPRO	IPR003074:Peroxisome proliferator-activated receptor	2.43E-05	2.46E-02
INTERPRO	IPR003079:Nuclear receptor ROR	2.43E-05	2.46E-02
GOTERM_MF_FAT	GO:0046872~metal ion binding	4.28E-05	4.77E-02
GOTERM_BP_FAT	GO:0019216~regulation of lipid metabolic process	4.58E-05	6.96E-02
SP_PIR_KEYWORDS	activator	4.60E-05	4.70E-02

Cluster 4 - intolerant	Gene Symbol	Gene description	EvoTol %ile
Mann-Whitney U test $P = 1.2 \times 10^{-5}$	*RPS6KB1*	ribosomal protein S6 kinase, 70kDa, polypeptide 1 [Source:HGNC Symbol;Acc:10436]	58.39
	PLEKHG4	pleckstrin homology domain containing, family G (with RhoGef domain) member 4 [Source:HGNC Symbol;Acc:24501]	96.96
	KCNQ2	potassium voltage-gated channel, KQT-like subfamily, member 2 [Source:HGNC Symbol;Acc:6296]	**2.86**
	ALG1	ALG1, chitobiosyldiphosphodolichol beta-mannosyltransferase [Source:HGNC Symbol;Acc:18294]	**14.09**
	CHD3	chromodomain helicase DNA binding protein 3 [Source:HGNC Symbol;Acc:1918]	**2.20**
	PROC	protein C (inactivator of coagulation factors Va and VIIIa) [Source:HGNC Symbol;Acc:9451]	**2.90**
	MMP3	matrix metallopeptidase 3 (stromelysin 1, progelatinase) [Source:HGNC Symbol;Acc:7173]	88.99
	CRABP1	cellular retinoic acid binding protein 1 [Source:HGNC Symbol;Acc:2338]	55.74
	MPDU1	mannose-P-dolichol utilization defect 1 [Source:HGNC Symbol;Acc:7207]	**18.98**
	EVI2A	ecotropic viral integration site 2A [Source:HGNC Symbol;Acc:3499]	**16.56**
	KCNJ2	potassium inwardly-rectifying channel, subfamily J, member 2 [Source:HGNC Symbol;Acc:6263]	**2.17**
	SHROOM3	shroom family member 3 [Source:HGNC Symbol;Acc:30422]	99.45
	ATP5C1	ATP synthase, H+ transporting, mitochondrial F1 complex, gamma polypeptide 1 [Source:HGNC Symbol;Acc:833]	**13.17**
	C12orf49	chromosome 12 open reading frame 49 [Source:HGNC Symbol;Acc:26128]	75.40
	VAMP7	vesicle-associated membrane protein 7 [Source:HGNC Symbol;Acc:11486]	60.85
	CCT6A	chaperonin containing TCP1, subunit 6A (zeta 1) [Source:HGNC Symbol;Acc:1620]	33.75
	DOK2	docking protein 2, 56kDa [Source:HGNC Symbol;Acc:2991]	**21.63**
	NAT8	N-acetyltransferase 8 (GCN5-related, putative) [Source:HGNC Symbol;Acc:18069]	82.46
	MAP2K4	mitogen-activated protein kinase kinase 4 [Source:HGNC Symbol;Acc:6844]	58.57
	CCT8	chaperonin containing TCP1, subunit 8 (theta) [Source:HGNC Symbol;Acc:1623]	31.38
	CHKA	choline kinase alpha [Source:HGNC Symbol;Acc:1937]	71.76
	SMARCD2	SWI/SNF related, matrix associated, actin dependent regulator of chromatin, subfamily d, member 2 [Source:HGNC Symbol;Acc:11107]	65.81
	DLD	dihydrolipoamide dehydrogenase [Source:HGNC Symbol;Acc:2898]	27.95
	HEY2	hes-related family bHLH transcription factor with YRPW motif 2 [Source:HGNC Symbol;Acc:4881]	40.36
	UBA2	ubiquitin-like modifier activating enzyme 2 [Source:HGNC Symbol;Acc:30661]	73.46
	DLL1	delta-like 1 (Drosophila) [Source:HGNC Symbol;Acc:2908]	**4.25**
	RGMA	repulsive guidance molecule family member a [Source:HGNC Symbol;Acc:30308]	**9.96**
	STRN	striatin, calmodulin binding protein [Source:HGNC Symbol;Acc:11424]	42.41
	ATRX	alpha thalassemia/mental retardation syndrome X-linked [Source:HGNC Symbol;Acc:886]	**0.92**
	ALG3	ALG3, alpha-1,3- mannosyltransferase [Source:HGNC Symbol;Acc:23056]	**7.90**
	PPP2R4	protein phosphatase 2A activator, regulatory subunit 4 [Source:HGNC Symbol;Acc:9308]	76.97
	XPC	xeroderma pigmentosum, complementation group C [Source:HGNC Symbol;Acc:12816]	66.43
	SGSM2	small G protein signaling modulator 2 [Source:HGNC Symbol;Acc:29026]	98.70
	FBXO22	F-box protein 22 [Source:HGNC Symbol;Acc:13593]	**12.52**
	CNIH	--	41.91
	SIP1	--	**13.94**
	CCT5	chaperonin containing TCP1, subunit 5 (epsilon) [Source:HGNC Symbol;Acc:1618]	**22.85**
	PPP2R2B	protein phosphatase 2, regulatory subunit B, beta [Source:HGNC Symbol;Acc:9305]	72.94
	SMARCD1	SWI/SNF related, matrix associated, actin dependent regulator of chromatin, subfamily d, member 1 [Source:HGNC Symbol;Acc:11106]	26.41
	IL1R2	interleukin 1 receptor, type II [Source:HGNC Symbol;Acc:5994]	85.18
	CRLF2	cytokine receptor-like factor 2 [Source:HGNC Symbol;Acc:14281]	-
	IL19	interleukin 19 [Source:HGNC Symbol;Acc:5990]	53.44
	ALDH7A1	aldehyde dehydrogenase 7 family, member A1 [Source:HGNC Symbol;Acc:877]	**10.13**
	ALG6	ALG6, alpha-1,3-glucosyltransferase [Source:HGNC Symbol;Acc:23157]	**7.69**
	ALDH3A2	aldehyde dehydrogenase 3 family, member A2 [Source:HGNC Symbol;Acc:403]	**10.78**
	GAD2	glutamate decarboxylase 2 (pancreatic islets and brain, 65kDa) [Source:HGNC Symbol;Acc:4093]	87.15
	PRKX	protein kinase, X-linked [Source:HGNC Symbol;Acc:9441]	72.16
	IL13RA1	interleukin 13 receptor, alpha 1 [Source:HGNC Symbol;Acc:5974]	**10.31**
	NEUROG1	neurogenin 1 [Source:HGNC Symbol;Acc:7764]	**22.36**
	SYNGR3	synaptogyrin 3 [Source:HGNC Symbol;Acc:11501]	57.62
	LY96	lymphocyte antigen 96 [Source:HGNC Symbol;Acc:17156]	-
	IL17F	interleukin 17F [Source:HGNC Symbol;Acc:16404]	60.37
	SGCG	sarcoglycan, gamma (35kDa dystrophin-associated glycoprotein) [Source:HGNC Symbol;Acc:10809]	**8.93**
	TPD52L1	tumor protein D52-like 1 [Source:HGNC Symbol;Acc:12006]	64.86
	ATN1	atrophin 1 [Source:HGNC Symbol;Acc:3033]	96.19
	KCNQ3	potassium voltage-gated channel, KQT-like subfamily, member 3 [Source:HGNC Symbol;Acc:6297]	**2.99**
	ALDH1B1	aldehyde dehydrogenase 1 family, member B1 [Source:HGNC Symbol;Acc:407]	30.43
	H2AFZ	H2A histone family, member Z [Source:HGNC Symbol;Acc:4741]	27.65
	DGKH	diacylglycerol kinase, eta [Source:HGNC Symbol;Acc:2854]	**17.51**
	CHFR	checkpoint with forkhead and ring finger domains, E3 ubiquitin protein ligase [Source:HGNC Symbol;Acc:20455]	40.32
	RAB11FIP4	RAB11 family interacting protein 4 (class II) [Source:HGNC Symbol;Acc:30267]	**6.56**
	PAPD5	PAP associated domain containing 5 [Source:HGNC Symbol;Acc:30758]	53.31
	ACTB	actin, beta [Source:HGNC Symbol;Acc:132]	**1.39**
	EPHA4	EPH receptor A4 [Source:HGNC Symbol;Acc:3388]	25.12
	COX7B	cytochrome c oxidase subunit VIIb [Source:HGNC Symbol;Acc:2291]	48.15
	MERTK	c-mer proto-oncogene tyrosine kinase [Source:HGNC Symbol;Acc:7027]	32.56
	ARHGAP1	Rho GTPase activating protein 1 [Source:HGNC Symbol;Acc:673]	-
	DDO	D-aspartate oxidase [Source:HGNC Symbol;Acc:2727]	**17.37**
	LDB2	LIM domain binding 2 [Source:HGNC Symbol;Acc:6533]	28.42
	IL4I1	interleukin 4 induced 1 [Source:HGNC Symbol;Acc:19094]	**17.37**
	CBLC	Cbl proto-oncogene C, E3 ubiquitin protein ligase [Source:HGNC Symbol;Acc:15961]	**8.14**
	ANK2	ankyrin 2, neuronal [Source:HGNC Symbol;Acc:493]	28.00
	IVL	involucrin [Source:HGNC Symbol;Acc:6187]	95.39
	GATM	glycine amidinotransferase (L-arginine:glycine amidinotransferase) [Source:HGNC Symbol;Acc:4175]	67.81
	ITPR3	inositol 1,4,5-trisphosphate receptor, type 3 [Source:HGNC Symbol;Acc:6182]	**0.45**
	CHPT1	choline phosphotransferase 1 [Source:HGNC Symbol;Acc:17852]	45.69
	C10orf10	chromosome 10 open reading frame 10 [Source:HGNC Symbol;Acc:23355]	70.42
	SMAD6	SMAD family member 6 [Source:HGNC Symbol;Acc:6772]	**8.79**
	APBA1	amyloid beta (A4) precursor protein-binding, family A, member 1 [Source:HGNC Symbol;Acc:578]	89.66
	ATP5F1	ATP synthase, H+ transporting, mitochondrial Fo complex, subunit B1 [Source:HGNC Symbol;Acc:840]	71.86
	LANCL1	LanC lantibiotic synthetase component C-like 1 (bacterial) [Source:HGNC Symbol;Acc:6508]	41.82
	RAD17	RAD17 homolog (S. pombe) [Source:HGNC Symbol;Acc:9807]	63.94
	COL4A4	collagen, type IV, alpha 4 [Source:HGNC Symbol;Acc:2206]	**0.95**
	NOTCH2	notch 2 [Source:HGNC Symbol;Acc:7882]	**1.23**
	SGCB	sarcoglycan, beta (43kDa dystrophin-associated glycoprotein) [Source:HGNC Symbol;Acc:10806]	**6.00**
	GPD1L	glycerol-3-phosphate dehydrogenase 1-like [Source:HGNC Symbol;Acc:28956]	49.29
	IKBKE	inhibitor of kappa light polypeptide gene enhancer in B-cells, kinase epsilon [Source:HGNC Symbol;Acc:14552]	92.22
	RPS6KA6	ribosomal protein S6 kinase, 90kDa, polypeptide 6 [Source:HGNC Symbol;Acc:10435]	61.01
	PTPN12	protein tyrosine phosphatase, non-receptor type 12 [Source:HGNC Symbol;Acc:9645]	37.79
	ALDOC	aldolase C, fructose-bisphosphate [Source:HGNC Symbol;Acc:418]	**9.94**
	CTGF	connective tissue growth factor [Source:HGNC Symbol;Acc:2500]	35.40
	TXNDC9	thioredoxin domain containing 9 [Source:HGNC Symbol;Acc:24110]	53.15
	ALG12	ALG12, alpha-1,6-mannosyltransferase [Source:HGNC Symbol;Acc:19358]	**23.11**
	SNRPE	small nuclear ribonucleoprotein polypeptide E [Source:HGNC Symbol;Acc:11161]	-
	GPD2	glycerol-3-phosphate dehydrogenase 2 (mitochondrial) [Source:HGNC Symbol;Acc:4456]	**15.14**
	APOA1	apolipoprotein A-I [Source:HGNC Symbol;Acc:600]	37.38
	EXOC6	exocyst complex component 6 [Source:HGNC Symbol;Acc:23196]	89.62
	LIPF	lipase, gastric [Source:HGNC Symbol;Acc:6622]	32.93
	PMM2	phosphomannomutase 2 [Source:HGNC Symbol;Acc:9115]	**2.87**
	ALDOB	aldolase B, fructose-bisphosphate [Source:HGNC Symbol;Acc:417]	**3.55**
	SNHG3-RCC1	--	-
	C1QTNF6	C1q and tumor necrosis factor related protein 6 [Source:HGNC Symbol;Acc:14343]	37.69
	RCC1	regulator of chromosome condensation 1 [Source:HGNC Symbol;Acc:1913]	**23.40**
	TKT	transketolase [Source:HGNC Symbol;Acc:11834]	**14.14**
	DGKD	diacylglycerol kinase, delta 130kDa [Source:HGNC Symbol;Acc:2851]	**18.68**
	RFT1	RFT1 homolog (S. cerevisiae) [Source:HGNC Symbol;Acc:30220]	**4.70**
	BAG4	BCL2-associated athanogene 4 [Source:HGNC Symbol;Acc:940]	**21.75**
	MT1G	metallothionein 1G [Source:HGNC Symbol;Acc:7399]	-
	EPHA3	EPH receptor A3 [Source:HGNC Symbol;Acc:3387]	**19.11**
	WDR77	WD repeat domain 77 [Source:HGNC Symbol;Acc:29652]	57.57
	PA2G4	proliferation-associated 2G4, 38kDa [Source:HGNC Symbol;Acc:8550]	29.87
	TAX1BP1	Tax1 (human T-cell leukemia virus type I) binding protein 1 [Source:HGNC Symbol;Acc:11575]	84.62
	PGAM4	phosphoglycerate mutase family member 4 [Source:HGNC Symbol;Acc:21731]	34.73
	CRX	cone-rod homeobox [Source:HGNC Symbol;Acc:2383]	**8.41**
	PFKFB1	6-phosphofructo-2-kinase/fructose-2,6-biphosphatase 1 [Source:HGNC Symbol;Acc:8872]	**15.09**
	PFKFB4	6-phosphofructo-2-kinase/fructose-2,6-biphosphatase 4 [Source:HGNC Symbol;Acc:8875]	**10.54**
	PFKM	phosphofructokinase, muscle [Source:HGNC Symbol;Acc:8877]	**19.69**
	ITGB7	integrin, beta 7 [Source:HGNC Symbol;Acc:6162]	**3.12**
	G6PD	glucose-6-phosphate dehydrogenase [Source:HGNC Symbol;Acc:4057]	**1.56**
	NRIP1	nuclear receptor interacting protein 1 [Source:HGNC Symbol;Acc:8001]	96.58
	SGCD	sarcoglycan, delta (35kDa dystrophin-associated glycoprotein) [Source:HGNC Symbol;Acc:10807]	**22.92**
	APOA2	apolipoprotein A-II [Source:HGNC Symbol;Acc:601]	**19.34**
	ALG2	ALG2, alpha-1,3/1,6-mannosyltransferase [Source:HGNC Symbol;Acc:23159]	33.21
	PFKFB3	6-phosphofructo-2-kinase/fructose-2,6-biphosphatase 3 [Source:HGNC Symbol;Acc:8874]	**10.17**
	G6PC2	glucose-6-phosphatase, catalytic, 2 [Source:HGNC Symbol;Acc:28906]	37.59
	BANF1	barrier to autointegration factor 1 [Source:HGNC Symbol;Acc:17397]	50.07
	STOML2	stomatin (EPB72)-like 2 [Source:HGNC Symbol;Acc:14559]	-
	FLT4	fms-related tyrosine kinase 4 [Source:HGNC Symbol;Acc:3767]	**5.96**
	GPI	glucose-6-phosphate isomerase [Source:HGNC Symbol;Acc:4458]	**2.71**
	RAPGEF1	Rap guanine nucleotide exchange factor (GEF) 1 [Source:HGNC Symbol;Acc:4568]	88.31
	COX5B	cytochrome c oxidase subunit Vb [Source:HGNC Symbol;Acc:2269]	25.40
	UTP6	UTP6, small subunit (SSU) processome component, homolog (yeast) [Source:HGNC Symbol;Acc:18279]	84.14
	KIFC3	kinesin family member C3 [Source:HGNC Symbol;Acc:6326]	94.54

Enrichment Type	Enrichment Term	P-value of enrichment	FDR
SP_PIR_KEYWORDS	disease mutation	8.44E-29	1.18E-25
SP_PIR_KEYWORDS	transferase	3.07E-16	4.66E-13
SP_PIR_KEYWORDS	atp-binding	4.26E-14	5.96E-11
UP_SEQ_FEATURE	nucleotide phosphate-binding region:ATP	9.48E-13	1.51E-09
UP_SEQ_FEATURE	active site:Proton acceptor	2.26E-11	3.61E-08
SP_PIR_KEYWORDS	kinase	7.14E-11	9.97E-08
SP_PIR_KEYWORDS	nucleotide-binding	5.11E-10	7.14E-07
SP_PIR_KEYWORDS	congenital disorder of glycosylation	1.21E-09	1.69E-06
GOTERM_MF_FAT	GO:0005524~ATP binding	1.52E-09	2.24E-06
UP_SEQ_FEATURE	binding site:ATP	1.76E-09	2.81E-06
GOTERM_MF_FAT	GO:0032559~adenyl ribonucleotide binding	2.65E-09	3.92E-06
GOTERM_MF_FAT	GO:0030554~adenyl nucleotide binding	3.70E-09	5.46E-06
SP_PIR_KEYWORDS	spinocerebellar ataxia	4.29E-09	5.99E-06
GOTERM_MF_FAT	GO:0001883~purine nucleoside binding	6.94E-09	1.03E-05
GOTERM_MF_FAT	GO:0001882~nucleoside binding	9.22E-09	1.36E-05
SP_PIR_KEYWORDS	neurodegeneration	1.21E-08	1.69E-05
KEGG_PATHWAY	hsa00561:Glycerolipid metabolism	1.66E-08	1.95E-05
GOTERM_BP_FAT	GO:0006000~fructose metabolic process	3.44E-08	5.95E-05
KEGG_PATHWAY	hsa00250:Alanine, aspartate and glutamate metabolism	3.62E-08	4.25E-05
GOTERM_BP_FAT	GO:0006007~glucose catabolic process	3.89E-08	6.73E-05
SP_PIR_KEYWORDS	liver	6.09E-08	8.50E-05
SP_PIR_KEYWORDS	phosphotransferase	8.07E-08	1.13E-04
GOTERM_BP_FAT	GO:0043933~macromolecular complex subunit organization	8.38E-08	1.45E-04
SP_PIR_KEYWORDS	phosphoprotein	1.07E-07	1.50E-04
GOTERM_BP_FAT	GO:0007167~enzyme linked receptor protein signaling pathway	1.18E-07	2.04E-04
GOTERM_BP_FAT	GO:0046164~alcohol catabolic process	1.65E-07	2.85E-04
GOTERM_BP_FAT	GO:0019318~hexose metabolic process	2.00E-07	3.46E-04
GOTERM_BP_FAT	GO:0019320~hexose catabolic process	2.52E-07	4.36E-04
GOTERM_BP_FAT	GO:0044275~cellular carbohydrate catabolic process	2.84E-07	4.90E-04
GOTERM_BP_FAT	GO:0046365~monosaccharide catabolic process	3.41E-07	5.89E-04
SP_PIR_KEYWORDS	acetylation	4.20E-07	5.87E-04
GOTERM_BP_FAT	GO:0046486~glycerolipid metabolic process	4.75E-07	8.22E-04
UP_SEQ_FEATURE	sequence variant	6.10E-07	9.74E-04
GOTERM_MF_FAT	GO:0004806~triacylglycerol lipase activity	6.24E-07	9.21E-04
SP_PIR_KEYWORDS	leber congenital amaurosis	6.31E-07	8.81E-04
INTERPRO	IPR017441:Protein kinase, ATP binding site	9.35E-07	0.001404339
GOTERM_BP_FAT	GO:0015918~sterol transport	1.01E-06	0.001745935
GOTERM_BP_FAT	GO:0030301~cholesterol transport	1.01E-06	0.001745935
GOTERM_BP_FAT	GO:0065003~macromolecular complex assembly	1.12E-06	0.001934562
GOTERM_BP_FAT	GO:0006006~glucose metabolic process	1.20E-06	0.002069845
GOTERM_MF_FAT	GO:0000166~nucleotide binding	1.35E-06	0.001994259
GOTERM_BP_FAT	GO:0005996~monosaccharide metabolic process	1.70E-06	0.002937604
GOTERM_BP_FAT	GO:0007169~transmembrane receptor protein tyrosine kinase signaling pathway	1.93E-06	0.003344158
GOTERM_MF_FAT	GO:0032555~purine ribonucleotide binding	3.00E-06	0.004433741
GOTERM_MF_FAT	GO:0032553~ribonucleotide binding	3.00E-06	0.004433741
GOTERM_BP_FAT	GO:0006468~protein amino acid phosphorylation	3.25E-06	0.005611776
GOTERM_MF_FAT	GO:0017076~purine nucleotide binding	3.46E-06	0.00511204
SP_PIR_KEYWORDS	ATP	3.70E-06	0.005169175
KEGG_PATHWAY	hsa00010:Glycolysis / Gluconeogenesis	3.91E-06	0.00458167
GOTERM_BP_FAT	GO:0016052~carbohydrate catabolic process	4.24E-06	0.007337707
GOTERM_BP_FAT	GO:0016310~phosphorylation	4.42E-06	0.007637519
INTERPRO	IPR000719:Protein kinase, core	6.77E-06	0.010160687
GOTERM_BP_FAT	GO:0006096~glycolysis	6.81E-06	0.011775332
GOTERM_MF_FAT	GO:0016620~oxidoreductase activity, acting on the aldehyde or oxo group of donors, NAD or NADP as acceptor	6.93E-06	0.010230758
GOTERM_MF_FAT	GO:0008443~phosphofructokinase activity	6.94E-06	0.010256137
GOTERM_BP_FAT	GO:0019637~organophosphate metabolic process	7.67E-06	0.013264535
UP_SEQ_FEATURE	domain:Protein kinase	8.49E-06	0.013554554
GOTERM_BP_FAT	GO:0006639~acylglycerol metabolic process	9.41E-06	0.016262752
SP_PIR_KEYWORDS	glycolysis	9.48E-06	0.013247451
GOTERM_BP_FAT	GO:0006638~neutral lipid metabolic process	1.10E-05	0.018998969
GOTERM_BP_FAT	GO:0006662~glycerol ether metabolic process	1.28E-05	0.022113017
GOTERM_MF_FAT	GO:0004029~aldehyde dehydrogenase (NAD) activity	1.37E-05	0.020164335
GOTERM_BP_FAT	GO:0006796~phosphate metabolic process	1.43E-05	0.024796369
GOTERM_BP_FAT	GO:0006793~phosphorus metabolic process	1.43E-05	0.024796369
GOTERM_MF_FAT	GO:0000030~mannosyltransferase activity	1.59E-05	0.023491842
GOTERM_BP_FAT	GO:0043691~reverse cholesterol transport	1.68E-05	0.029063449
GOTERM_BP_FAT	GO:0018904~organic ether metabolic process	1.71E-05	0.02964129
GOTERM_MF_FAT	GO:0004672~protein kinase activity	1.77E-05	0.026199859
UP_SEQ_FEATURE	region of interest:Heparin-binding	1.92E-05	0.030646492
UP_SEQ_FEATURE	binding site:Fructose-6-phosphate	2.05E-05	0.032741742
UP_SEQ_FEATURE	region of interest:6-phosphofructo-2-kinase	2.05E-05	0.032741742
UP_SEQ_FEATURE	region of interest:Fructose-2,6-bisphosphatase	2.05E-05	0.032741742
GOTERM_MF_FAT	GO:0019955~cytokine binding	2.20E-05	0.032536046
SP_PIR_KEYWORDS	tyrosine-protein kinase	2.26E-05	0.031576853
GOTERM_BP_FAT	GO:0044269~glycerol ether catabolic process	2.53E-05	0.043675165
GOTERM_BP_FAT	GO:0046464~acylglycerol catabolic process	2.53E-05	0.043675165
GOTERM_BP_FAT	GO:0046461~neutral lipid catabolic process	2.53E-05	0.043675165
INTERPRO	IPR001345:Phosphoglycerate/bisphosphoglycerate mutase	2.66E-05	0.039891612
INTERPRO	IPR002194:Chaperonin TCP-1, conserved site	2.66E-05	0.039891612
UP_SEQ_FEATURE	active site:Tele-phosphohistidine intermediate	2.68E-05	0.04277092
INTERPRO	IPR003094:Fructose-2,6-bisphosphatase	2.85E-05	0.042757211
INTERPRO	IPR013079:6-phosphofructo-2-kinase	2.85E-05	0.042757211
INTERPRO	IPR016260:Bifunctional 6-phosphofructo-2-kinase/fructose-2, 6-bisphosphate 2-phosphatase	2.85E-05	0.042757211

TM9SF3	transmembrane 9 superfamily member 3 [Source:HGNC Symbol;Acc:21529]	62.49
CDKN2D	cyclin-dependent kinase inhibitor 2D (p19, inhibits CDK4) [Source:HGNC Symbol;Acc:1790]	-
NAT2	N-acetyltransferase 2 (arylamine N-acetyltransferase) [Source:HGNC Symbol;Acc:7646]	87.11
ANKRD57	--	65.57
TALDO1	transaldolase 1 [Source:HGNC Symbol;Acc:11559]	**4.69**
FGF23	fibroblast growth factor 23 [Source:HGNC Symbol;Acc:3680]	**11.79**
TGFBR1	transforming growth factor, beta receptor 1 [Source:HGNC Symbol;Acc:11772]	**7.81**
LIG4	ligase IV, DNA, ATP-dependent [Source:HGNC Symbol;Acc:6601]	27.85
GBAS	glioblastoma amplified sequence [Source:HGNC Symbol;Acc:4179]	58.70
IFNGR2	interferon gamma receptor 2 (interferon gamma transducer 1) [Source:HGNC Symbol;Acc:5440]	82.95
SCGB2A2	secretoglobin, family 2A, member 2 [Source:HGNC Symbol;Acc:7050]	34.87
OGG1	8-oxoguanine DNA glycosylase [Source:HGNC Symbol;Acc:8125]	38.58
RGL4	ral guanine nucleotide dissociation stimulator-like 4 [Source:HGNC Symbol;Acc:31911]	92.36
CHMP5	charged multivesicular body protein 5 [Source:HGNC Symbol;Acc:26942]	42.68
AIPL1	aryl hydrocarbon receptor interacting protein-like 1 [Source:HGNC Symbol;Acc:359]	**4.00**
CCL23	chemokine (C-C motif) ligand 23 [Source:HGNC Symbol;Acc:10622]	60.56
NCOA2	nuclear receptor coactivator 2 [Source:HGNC Symbol;Acc:7669]	86.71
NCK1	NCK adaptor protein 1 [Source:HGNC Symbol;Acc:7664]	39.24
C17orf79	--	61.51
CRADD	CASP2 and RIPK1 domain containing adaptor with death domain [Source:HGNC Symbol;Acc:2340]	28.31
EVI2B	ecotropic viral integration site 2B [Source:HGNC Symbol;Acc:3500]	76.54
STRN3	striatin, calmodulin binding protein 3 [Source:HGNC Symbol;Acc:15720]	**12.17**
ARID1A	AT rich interactive domain 1A (SWI-like) [Source:HGNC Symbol;Acc:11110]	98.53
C20orf7	--	**18.07**
VAV3	vav 3 guanine nucleotide exchange factor [Source:HGNC Symbol;Acc:12659]	**24.83**
RAD50	RAD50 homolog (S. cerevisiae) [Source:HGNC Symbol;Acc:9816]	96.47
LSAMP	limbic system-associated membrane protein [Source:HGNC Symbol;Acc:6705]	57.08
FANCG	Fanconi anemia, complementation group G [Source:HGNC Symbol;Acc:3588]	81.48
SMOC2	SPARC related modular calcium binding 2 [Source:HGNC Symbol;Acc:20323]	75.46
IL26	interleukin 26 [Source:HGNC Symbol;Acc:17119]	42.51
PABPC1	poly(A) binding protein, cytoplasmic 1 [Source:HGNC Symbol;Acc:8554]	**20.41**
NDUFAF3	NADH dehydrogenase (ubiquinone) complex I, assembly factor 3 [Source:HGNC Symbol;Acc:29918]	**24.38**
CACNA1A	calcium channel, voltage-dependent, P/Q type, alpha 1A subunit [Source:HGNC Symbol;Acc:1388]	**0.96**
F2	coagulation factor II (thrombin) [Source:HGNC Symbol;Acc:3535]	**5.66**
FAM40A	--	84.38
IRAK3	interleukin-1 receptor-associated kinase 3 [Source:HGNC Symbol;Acc:17020]	32.97
APOC3	apolipoprotein C-III [Source:HGNC Symbol;Acc:610]	27.60
MOBKL3	--	34.07
LIFR	leukemia inhibitory factor receptor alpha [Source:HGNC Symbol;Acc:6597]	37.23
GOT1	glutamic-oxaloacetic transaminase 1, soluble [Source:HGNC Symbol;Acc:4432]	**23.21**
PDK1	pyruvate dehydrogenase kinase, isozyme 1 [Source:HGNC Symbol;Acc:8809]	80.67
ETFA	electron-transfer-flavoprotein, alpha polypeptide [Source:HGNC Symbol;Acc:3481]	**10.23**
ZNF615	zinc finger protein 615 [Source:HGNC Symbol;Acc:24740]	88.85
LPCAT2	lysophosphatidylcholine acyltransferase 2 [Source:HGNC Symbol;Acc:26032]	**20.47**
FAP	fibroblast activation protein, alpha [Source:HGNC Symbol;Acc:3590]	**15.12**
ITK	IL2-inducible T-cell kinase [Source:HGNC Symbol;Acc:6171]	**10.14**
SNRPD1	small nuclear ribonucleoprotein D1 polypeptide 16kDa [Source:HGNC Symbol;Acc:11158]	48.37
ASNS	asparagine synthetase (glutamine-hydrolyzing) [Source:HGNC Symbol;Acc:753]	39.74
XRCC3	X-ray repair complementing defective repair in Chinese hamster cells 3 [Source:HGNC Symbol;Acc:12830]	46.41
IL1RL1	interleukin 1 receptor-like 1 [Source:HGNC Symbol;Acc:5998]	51.83
ZNF432	zinc finger protein 432 [Source:HGNC Symbol;Acc:20810]	38.77
KIF7	kinesin family member 7 [Source:HGNC Symbol;Acc:30497]	97.27
STRN4	striatin, calmodulin binding protein 4 [Source:HGNC Symbol;Acc:15721]	35.43
ZNF350	zinc finger protein 350 [Source:HGNC Symbol;Acc:16656]	52.42
ITGB5	integrin, beta 5 [Source:HGNC Symbol;Acc:6160]	**2.24**
TTBK2	tau tubulin kinase 2 [Source:HGNC Symbol;Acc:19141]	89.97
DSCC1	DNA replication and sister chromatid cohesion 1 [Source:HGNC Symbol;Acc:24453]	62.89
SDHD	succinate dehydrogenase complex, subunit D, integral membrane protein [Source:HGNC Symbol;Acc:10683]	**5.70**
FGF14	fibroblast growth factor 14 [Source:HGNC Symbol;Acc:3671]	35.99
PLD2	phospholipase D2 [Source:HGNC Symbol;Acc:9068]	92.61
CD164	CD164 molecule, sialomucin [Source:HGNC Symbol;Acc:1632]	62.98
MRGPRF	MAS-related GPR, member F [Source:HGNC Symbol;Acc:24828]	67.71
CKB	creatine kinase, brain [Source:HGNC Symbol;Acc:1991]	74.56
C17orf42	--	53.68
GNA12	guanine nucleotide binding protein (G protein) alpha 12 [Source:HGNC Symbol;Acc:4380]	**10.55**
MAP3K1	mitogen-activated protein kinase kinase kinase 1, E3 ubiquitin protein ligase [Source:HGNC Symbol;Acc:6848]	92.69
C10orf26	--	73.99
HAT1	histone acetyltransferase 1 [Source:HGNC Symbol;Acc:4821]	**13.26**
LRRC37B	leucine rich repeat containing 37B [Source:HGNC Symbol;Acc:29070]	53.03
CDIPT	CDP-diacylglycerol--inositol 3-phosphatidyltransferase [Source:HGNC Symbol;Acc:1769]	55.76
SULT6B1	sulfotransferase family, cytosolic, 6B, member 1 [Source:HGNC Symbol;Acc:33433]	**20.42**
ATAD5	ATPase family, AAA domain containing 5 [Source:HGNC Symbol;Acc:25752]	31.50
PTPRA	protein tyrosine phosphatase, receptor type, A [Source:HGNC Symbol;Acc:9664]	**17.82**
CSF1R	colony stimulating factor 1 receptor [Source:HGNC Symbol;Acc:2433]	**11.88**
PRMT5	protein arginine methyltransferase 5 [Source:HGNC Symbol;Acc:10894]	**20.28**
TESK1	testis-specific kinase 1 [Source:HGNC Symbol;Acc:11731]	78.25
CYB5R1	cytochrome b5 reductase 1 [Source:HGNC Symbol;Acc:13397]	**11.03**
LPCAT1	lysophosphatidylcholine acyltransferase 1 [Source:HGNC Symbol;Acc:25718]	**13.99**
RDH12	retinol dehydrogenase 12 (all-trans/9-cis/11-cis) [Source:HGNC Symbol;Acc:19977]	**7.51**
BRAF	v-raf murine sarcoma viral oncogene homolog B [Source:HGNC Symbol;Acc:1097]	**3.65**
ALCAM	activated leukocyte cell adhesion molecule [Source:HGNC Symbol;Acc:400]	85.33
FANCA	Fanconi anemia, complementation group A [Source:HGNC Symbol;Acc:3582]	**0.47**
WFIKKN1	WAP, follistatin/kazal, immunoglobulin, kunitz and netrin domain containing 1 [Source:HGNC Symbol;Acc:30912]	86.54
ZNF574	zinc finger protein 574 [Source:HGNC Symbol;Acc:26166]	95.32
ARSE	arylsulfatase E (chondrodysplasia punctata 1) [Source:HGNC Symbol;Acc:719]	**4.93**
ABCF2	ATP-binding cassette, sub-family F (GCN20), member 2 [Source:HGNC Symbol;Acc:71]	**4.79**
NGEF	neuronal guanine nucleotide exchange factor [Source:HGNC Symbol;Acc:7807]	36.09
GAD1	glutamate decarboxylase 1 (brain, 67kDa) [Source:HGNC Symbol;Acc:4092]	88.68
NUBPL	nucleotide binding protein-like [Source:HGNC Symbol;Acc:20278]	57.01
SCP2	sterol carrier protein 2 [Source:HGNC Symbol;Acc:10606]	**23.53**
CSF2RB	colony stimulating factor 2 receptor, beta, low-affinity (granulocyte-macrophage) [Source:HGNC Symbol;Acc:2436]	**2.77**
LCAT	lecithin-cholesterol acyltransferase [Source:HGNC Symbol;Acc:6522]	**6.35**
SOCS7	suppressor of cytokine signaling 7 [Source:HGNC Symbol;Acc:29846]	45.91
NDUFAF4	NADH dehydrogenase (ubiquinone) complex I, assembly factor 4 [Source:HGNC Symbol;Acc:21034]	**18.37**
CRLS1	cardiolipin synthase 1 [Source:HGNC Symbol;Acc:16148]	**22.79**
SUZ12	SUZ12 polycomb repressive complex 2 subunit [Source:HGNC Symbol;Acc:17101]	40.18
NDRG1	N-myc downstream regulated 1 [Source:HGNC Symbol;Acc:7679]	79.67
SKIL	SKI-like oncogene [Source:HGNC Symbol;Acc:10897]	**9.49**
RPE65	retinal pigment epithelium-specific protein 65kDa [Source:HGNC Symbol;Acc:10294]	**1.13**
DPM3	dolichyl-phosphate mannosyltransferase polypeptide 3 [Source:HGNC Symbol;Acc:3007]	34.11
ADPGK	ADP-dependent glucokinase [Source:HGNC Symbol;Acc:25250]	61.58
IL5RA	interleukin 5 receptor, alpha [Source:HGNC Symbol;Acc:6017]	**23.58**
TCP1	t-complex 1 [Source:HGNC Symbol;Acc:11655]	47.06
HOXA9	homeobox A9 [Source:HGNC Symbol;Acc:5109]	**11.64**
GAPDH	glyceraldehyde-3-phosphate dehydrogenase [Source:HGNC Symbol;Acc:4141]	89.61
CAD	carbamoyl-phosphate synthetase 2, aspartate transcarbamylase, and dihydroorotase [Source:HGNC Symbol;Acc:1424]	-
COL5A2	collagen, type V, alpha 2 [Source:HGNC Symbol;Acc:2210]	**1.08**
LIPG	lipase, endothelial [Source:HGNC Symbol;Acc:6623]	**7.74**
TNFRSF11A	tumor necrosis factor receptor superfamily, member 11a, NFKB activator [Source:HGNC Symbol;Acc:11908]	32.64
MLH1	mutL homolog 1 [Source:HGNC Symbol;Acc:7127]	**0.09**
PDC	phosducin [Source:HGNC Symbol;Acc:8759]	56.97
CIB1	calcium and integrin binding 1 (calmyrin) [Source:HGNC Symbol;Acc:16920]	64.54
ABCD3	ATP-binding cassette, sub-family D (ALD), member 3 [Source:HGNC Symbol;Acc:67]	**10.62**
PIK3C3	phosphatidylinositol 3-kinase, catalytic subunit type 3 [Source:HGNC Symbol;Acc:8974]	25.98
FGFR4	fibroblast growth factor receptor 4 [Source:HGNC Symbol;Acc:3691]	**4.37**
LRAT	lecithin retinol acyltransferase (phosphatidylcholine--retinol O-acyltransferase) [Source:HGNC Symbol;Acc:6685]	64.32
PROS1	protein S (alpha) [Source:HGNC Symbol;Acc:9456]	**8.42**
KHDRBS1	KH domain containing, RNA binding, signal transduction associated 1 [Source:HGNC Symbol;Acc:18116]	55.55
CCT7	chaperonin containing TCP1, subunit 7 (eta) [Source:HGNC Symbol;Acc:1622]	46.69
CRLF3	cytokine receptor-like factor 3 [Source:HGNC Symbol;Acc:17177]	73.92
SMN1	survival of motor neuron 1, telomeric [Source:HGNC Symbol;Acc:11117]	-
CRB1	crumbs homolog 1 (Drosophila) [Source:HGNC Symbol;Acc:2343]	**1.28**
ACVR1	activin A receptor, type I [Source:HGNC Symbol;Acc:171]	**15.08**
FKTN	fukutin [Source:HGNC Symbol;Acc:3622]	**6.86**
PFKFB2	6-phosphofructo-2-kinase/fructose-2,6-biphosphatase 2 [Source:HGNC Symbol;Acc:8873]	**10.62**
L1CAM	L1 cell adhesion molecule [Source:HGNC Symbol;Acc:6470]	**23.01**
PRKCG	protein kinase C, gamma [Source:HGNC Symbol;Acc:9402]	25.89
STAP2	signal transducing adaptor family member 2 [Source:HGNC Symbol;Acc:30430]	80.15
PRKACG	protein kinase, cAMP-dependent, catalytic, gamma [Source:HGNC Symbol;Acc:9382]	46.93
ASPA	aspartoacylase [Source:HGNC Symbol;Acc:756]	**8.27**
CXCR4	chemokine (C-X-C motif) receptor 4 [Source:HGNC Symbol;Acc:2561]	67.90
FGR	feline Gardner-Rasheed sarcoma viral oncogene homolog [Source:HGNC Symbol;Acc:3697]	25.04
ACY3	aspartoacylase (aminocyclase) 3 [Source:HGNC Symbol;Acc:24104]	**5.56**
NDRG4	NDRG family member 4 [Source:HGNC Symbol;Acc:14466]	79.66
USP22	ubiquitin specific peptidase 22 [Source:HGNC Symbol;Acc:12621]	53.87
AATF	apoptosis antagonizing transcription factor [Source:HGNC Symbol;Acc:19235]	**7.32**
RGMB	repulsive guidance molecule family member b [Source:HGNC Symbol;Acc:26896]	**13.06**
CELF3	CUGBP, Elav-like family member 3 [Source:HGNC Symbol;Acc:11967]	43.82
MBD3L1	methyl-CpG binding domain protein 3-like 1 [Source:HGNC Symbol;Acc:15774]	61.21
APOB	apolipoprotein B [Source:HGNC Symbol;Acc:603]	99.83
TULP1	tubby like protein 1 [Source:HGNC Symbol;Acc:12423]	**3.63**
ADAP2	ArfGAP with dual PH domains 2 [Source:HGNC Symbol;Acc:16487]	44.89
FKRP	fukutin related protein [Source:HGNC Symbol;Acc:17997]	**5.73**
C10orf2	chromosome 10 open reading frame 2 [Source:HGNC Symbol;Acc:1160]	**4.14**
SLC17A7	solute carrier family 17 (vesicular glutamate transporter), member 7 [Source:HGNC Symbol;Acc:16704]	77.63
SIN3A	SIN3 transcription regulator family member A [Source:HGNC Symbol;Acc:19353]	94.35
RPS6KA4	ribosomal protein S6 kinase, 90kDa, polypeptide 4 [Source:HGNC Symbol;Acc:10433]	83.25
TRIM61	tripartite motif containing 61 [Source:HGNC Symbol;Acc:24339]	50.84
ALDH4A1	aldehyde dehydrogenase 4 family, member A1 [Source:HGNC Symbol;Acc:406]	53.74
NFASC	neurofascin [Source:HGNC Symbol;Acc:29866]	86.90
LCA5	Leber congenital amaurosis 5 [Source:HGNC Symbol;Acc:31923]	70.51
CPB2	carboxypeptidase B2 (plasma) [Source:HGNC Symbol;Acc:2300]	80.46
GAPDHS	glyceraldehyde-3-phosphate dehydrogenase, spermatogenic [Source:HGNC Symbol;Acc:24864]	84.34
TIMP2	TIMP metallopeptidase inhibitor 2 [Source:HGNC Symbol;Acc:11821]	25.90
ABI1	abl-interactor 1 [Source:HGNC Symbol;Acc:11320]	**21.95**
LCP2	lymphocyte cytosolic protein 2 (SH2 domain containing leukocyte protein of 76kDa) [Source:HGNC Symbol;Acc:6529]	30.22
ALDH2	aldehyde dehydrogenase 2 family (mitochondrial) [Source:HGNC Symbol;Acc:404]	44.11
SYNCRIP	synaptotagmin binding, cytoplasmic RNA interacting protein [Source:HGNC Symbol;Acc:16918]	70.62
NOTCH3	notch 3 [Source:HGNC Symbol;Acc:7883]	**0.72**
PNPLA3	patatin-like phospholipase domain containing 3 [Source:HGNC Symbol;Acc:18590]	39.36

NLRP3	NLR family, pyrin domain containing 3 [Source:HGNC Symbol;Acc:16400]	**1.02**
ALG8	ALG8, alpha-1,3-glucosyltransferase [Source:HGNC Symbol;Acc:23161]	**5.32**
CDC37L1	cell division cycle 37-like 1 [Source:HGNC Symbol;Acc:17179]	65.42
HDAC1	histone deacetylase 1 [Source:HGNC Symbol;Acc:4852]	41.40
PDHX	pyruvate dehydrogenase complex, component X [Source:HGNC Symbol;Acc:21350]	93.29
PVALB	parvalbumin [Source:HGNC Symbol;Acc:9704]	53.27
DECR1	2,4-dienoyl CoA reductase 1, mitochondrial [Source:HGNC Symbol;Acc:2753]	30.33
WNT2	wingless-type MMTV integration site family member 2 [Source:HGNC Symbol;Acc:12780]	43.86
DEK	DEK oncogene [Source:HGNC Symbol;Acc:2768]	71.71
RPAP2	RNA polymerase II associated protein 2 [Source:HGNC Symbol;Acc:25791]	**7.42**
ANK1	ankyrin 1, erythrocytic [Source:HGNC Symbol;Acc:492]	**20.52**
PTCH1	patched 1 [Source:HGNC Symbol;Acc:9585]	**0.52**
CCL15	chemokine (C-C motif) ligand 15 [Source:HGNC Symbol;Acc:10613]	56.57
ATXN10	ataxin 10 [Source:HGNC Symbol;Acc:10549]	**13.57**
RUNX2	runt-related transcription factor 2 [Source:HGNC Symbol;Acc:10472]	**11.27**
LIPC	lipase, hepatic [Source:HGNC Symbol;Acc:6619]	**4.99**
ASS1	argininosuccinate synthase 1 [Source:HGNC Symbol;Acc:758]	**4.12**
RPS6KA3	ribosomal protein S6 kinase, 90kDa, polypeptide 3 [Source:HGNC Symbol;Acc:10432]	66.66
MUSK	muscle, skeletal, receptor tyrosine kinase [Source:HGNC Symbol;Acc:7525]	**21.43**
BAMBI	BMP and activin membrane-bound inhibitor [Source:HGNC Symbol;Acc:30251]	**13.84**
SMARCD3	SWI/SNF related, matrix associated, actin dependent regulator of chromatin, subfamily d, member 3 [Source:HGNC Symbol;Acc:11108]	45.91
COL5A1	collagen, type V, alpha 1 [Source:HGNC Symbol;Acc:2209]	**0.94**
THBD	thrombomodulin [Source:HGNC Symbol;Acc:11784]	**21.86**
TRAF3	TNF receptor-associated factor 3 [Source:HGNC Symbol;Acc:12033]	82.27
SPG7	spastic paraplegia 7 (pure and complicated autosomal recessive) [Source:HGNC Symbol;Acc:11237]	**1.86**
PNLIP	pancreatic lipase [Source:HGNC Symbol;Acc:9155]	**9.11**
CDK5	cyclin-dependent kinase 5 [Source:HGNC Symbol;Acc:1774]	51.18
DZIP1L	DAZ interacting zinc finger protein 1-like [Source:HGNC Symbol;Acc:26551]	95.52
PHYH	phytanoyl-CoA 2-hydroxylase [Source:HGNC Symbol;Acc:8940]	**4.87**
MBD3	methyl-CpG binding domain protein 3 [Source:HGNC Symbol;Acc:6918]	**14.05**
CKM	creatine kinase, muscle [Source:HGNC Symbol;Acc:1994]	48.32
MAPK13	mitogen-activated protein kinase 13 [Source:HGNC Symbol;Acc:6875]	81.95
DLL3	delta-like 3 (Drosophila) [Source:HGNC Symbol;Acc:2909]	**8.13**
LFNG	LFNG O-fucosylpeptide 3-beta-N-acetylglucosaminyltransferase [Source:HGNC Symbol;Acc:6560]	81.97
CEL	carboxyl ester lipase [Source:HGNC Symbol;Acc:1848]	64.72
OMG	oligodendrocyte myelin glycoprotein [Source:HGNC Symbol;Acc:8135]	61.14
NOD1	nucleotide-binding oligomerization domain containing 1 [Source:HGNC Symbol;Acc:16390]	81.13
PNLIPRP1	pancreatic lipase-related protein 1 [Source:HGNC Symbol;Acc:9156]	-
THBS2	thrombospondin 2 [Source:HGNC Symbol;Acc:11786]	**6.62**
HFE2	hemochromatosis type 2 (juvenile) [Source:HGNC Symbol;Acc:4887]	**7.49**
ATXN7	ataxin 7 [Source:HGNC Symbol;Acc:10560]	86.86
