# Supplementary material for: EvoTol: a protein-sequence based evolutionary intolerance framework for disease-gene prioritization
Source: Nucleic Acids Res. 2014 Dec 29;43(5):e33. doi: 10.1093/nar/gku1322 (PMC4357693; doi:10.1093/nar/gku1322)
Supplement: SUPPLEMENTARY DATA [file supp_gku1322_nar-02497-met-n-2014-File009.zip › Supp/Supplemental Table 7.pdf]

Supplemental table 7: The Uberon ontologies used for the cell-type specific gene expression enrichment of EvoTol with the corresponding FANTOM IDs.

Grouping Uberon ID	Grouping Uberon Name	Member Uberon ID	Member Uberon Name	FANTOM ID	FANTOM sample name
UBERON:0001004	Respiratory system	UBERON:0000065	respiratory tract	CNhs10625	lung, adult, pool1
UBERON:0001004	Respiratory system	UBERON:0000065	respiratory tract	CNhs10635	trachea, adult, pool1
UBERON:0001004	Respiratory system	UBERON:0000065	respiratory tract	CNhs11766	trachea, fetal, donor1
UBERON:0001004	Respiratory system	UBERON:0000065	respiratory tract	CNhs11680	lung, fetal, donor1
UBERON:0001004	Respiratory system	UBERON:0000065	respiratory tract	CNhs11786	lung, right lower lobe, adult, donor1
UBERON:0001004	Respiratory system	UBERON:0000065	respiratory tract	CNhs10884	Small Airway Epithelial Cells, donor1
UBERON:0001004	Respiratory system	UBERON:0000065	respiratory tract	CNhs12500	Fibroblast - Lung, donor1
UBERON:0001004	Respiratory system	UBERON:0000065	respiratory tract	CNhs11092	Tracheal Epithelial Cells, donor1
UBERON:0001004	Respiratory system	UBERON:0000065	respiratory tract	CNhs11975	Small Airway Epithelial Cells, donor2
UBERON:0001004	Respiratory system	UBERON:0000065	respiratory tract	CNhs11380	Fibroblast - Lung, donor2
UBERON:0001004	Respiratory system	UBERON:0000065	respiratory tract	CNhs11993	Tracheal Epithelial Cells, donor2
UBERON:0001004	Respiratory system	UBERON:0000065	respiratory tract	CNhs12016	Small Airway Epithelial Cells, donor3
UBERON:0001004	Respiratory system	UBERON:0000065	respiratory tract	CNhs12029	Fibroblast - Lung, donor3
UBERON:0001004	Respiratory system	UBERON:0000065	respiratory tract	CNhs12051	Tracheal Epithelial Cells, donor3
UBERON:0001004	Respiratory system	UBERON:0000065	respiratory tract	CNhs12054	Bronchial Epithelial Cell, donor4
UBERON:0001004	Respiratory system	UBERON:0000065	respiratory tract	CNhs12058	Bronchial Epithelial Cell, donor5
UBERON:0001004	Respiratory system	UBERON:0000065	respiratory tract	CNhs12062	Bronchial Epithelial Cell, donor6
UBERON:0001004	Respiratory system	UBERON:0000065	respiratory tract	CNhs11325	Alveolar Epithelial Cells, donor1
UBERON:0001004	Respiratory system	UBERON:0000065	respiratory tract	CNhs11327	Bronchial Epithelial Cell, donor1
UBERON:0001004	Respiratory system	UBERON:0000065	respiratory tract	CNhs11328	Smooth Muscle Cells - Bronchial, donor1
UBERON:0001004	Respiratory system	UBERON:0000065	respiratory tract	CNhs11329	Smooth Muscle Cells - Tracheal, donor1
UBERON:0001004	Respiratory system	UBERON:0000065	respiratory tract	CNhs12085	Bronchial Epithelial Cell, donor2
UBERON:0001004	Respiratory system	UBERON:0000065	respiratory tract	CNhs12348	Smooth Muscle Cells - Bronchial, donor2
UBERON:0001004	Respiratory system	UBERON:0000065	respiratory tract	CNhs12567	Smooth Muscle Cells - Tracheal, donor2
UBERON:0001004	Respiratory system	UBERON:0000065	respiratory tract	CNhs12119	Alveolar Epithelial Cells, donor3
UBERON:0001004	Respiratory system	UBERON:0000065	respiratory tract	CNhs12623	Bronchial Epithelial Cell, donor3
UBERON:0001004	Respiratory system	UBERON:0000065	respiratory tract	CNhs12894	Smooth Muscle Cells - Tracheal, donor3
UBERON:0001004	Respiratory system	UBERON:0000065	respiratory tract	CNhs12642	Bronchial Epithelial Cell, donor7
UBERON:0001004	Respiratory system	UBERON:0000072	segment of respiratory tract	CNhs10635	trachea, adult, pool1
UBERON:0001004	Respiratory system	UBERON:0000072	segment of respiratory tract	CNhs11766	trachea, fetal, donor1
UBERON:0001004	Respiratory system	UBERON:0000072	segment of respiratory tract	CNhs11092	Tracheal Epithelial Cells, donor1
UBERON:0001004	Respiratory system	UBERON:0000072	segment of respiratory tract	CNhs11993	Tracheal Epithelial Cells, donor2
UBERON:0001004	Respiratory system	UBERON:0000072	segment of respiratory tract	CNhs12051	Tracheal Epithelial Cells, donor3
UBERON:0001004	Respiratory system	UBERON:0000072	segment of respiratory tract	CNhs12054	Bronchial Epithelial Cell, donor4
UBERON:0001004	Respiratory system	UBERON:0000072	segment of respiratory tract	CNhs12058	Bronchial Epithelial Cell, donor5
UBERON:0001004	Respiratory system	UBERON:0000072	segment of respiratory tract	CNhs12062	Bronchial Epithelial Cell, donor6
UBERON:0001004	Respiratory system	UBERON:0000072	segment of respiratory tract	CNhs11325	Alveolar Epithelial Cells, donor1
UBERON:0001004	Respiratory system	UBERON:0000072	segment of respiratory tract	CNhs11327	Bronchial Epithelial Cell, donor1
UBERON:0001004	Respiratory system	UBERON:0000072	segment of respiratory tract	CNhs11328	Smooth Muscle Cells - Bronchial, donor1
UBERON:0001004	Respiratory system	UBERON:0000072	segment of respiratory tract	CNhs11329	Smooth Muscle Cells - Tracheal, donor1
UBERON:0001004	Respiratory system	UBERON:0000072	segment of respiratory tract	CNhs12085	Bronchial Epithelial Cell, donor2
UBERON:0001004	Respiratory system	UBERON:0000072	segment of respiratory tract	CNhs12348	Smooth Muscle Cells - Bronchial, donor2
UBERON:0001004	Respiratory system	UBERON:0000072	segment of respiratory tract	CNhs12567	Smooth Muscle Cells - Tracheal, donor2
UBERON:0001004	Respiratory system	UBERON:0000072	segment of respiratory tract	CNhs12119	Alveolar Epithelial Cells, donor3
UBERON:0001004	Respiratory system	UBERON:0000072	segment of respiratory tract	CNhs12623	Bronchial Epithelial Cell, donor3
UBERON:0001004	Respiratory system	UBERON:0000072	segment of respiratory tract	CNhs12894	Smooth Muscle Cells - Tracheal, donor3
UBERON:0001004	Respiratory system	UBERON:0000072	segment of respiratory tract	CNhs12642	Bronchial Epithelial Cell, donor7
UBERON:0001004	Respiratory system	UBERON:0000101	lobe of lung	CNhs11786	lung, right lower lobe, adult, donor1
UBERON:0001004	Respiratory system	UBERON:0000115	lung epithelium	CNhs12054	Bronchial Epithelial Cell, donor4
UBERON:0001004	Respiratory system	UBERON:0000115	lung epithelium	CNhs12058	Bronchial Epithelial Cell, donor5
UBERON:0001004	Respiratory system	UBERON:0000115	lung epithelium	CNhs12062	Bronchial Epithelial Cell, donor6
UBERON:0001004	Respiratory system	UBERON:0000115	lung epithelium	CNhs11325	Alveolar Epithelial Cells, donor1
UBERON:0001004	Respiratory system	UBERON:0000115	lung epithelium	CNhs11327	Bronchial Epithelial Cell, donor1
UBERON:0001004	Respiratory system	UBERON:0000115	lung epithelium	CNhs12085	Bronchial Epithelial Cell, donor2
UBERON:0001004	Respiratory system	UBERON:0000115	lung epithelium	CNhs12119	Alveolar Epithelial Cells, donor3
UBERON:0001004	Respiratory system	UBERON:0000115	lung epithelium	CNhs12623	Bronchial Epithelial Cell, donor3
UBERON:0001004	Respiratory system	UBERON:0000115	lung epithelium	CNhs12642	Bronchial Epithelial Cell, donor7
UBERON:0001004	Respiratory system	UBERON:0000117	respiratory tube	CNhs10625	lung, adult, pool1
UBERON:0001004	Respiratory system	UBERON:0000117	respiratory tube	CNhs11680	lung, fetal, donor1
UBERON:0001004	Respiratory system	UBERON:0000117	respiratory tube	CNhs11786	lung, right lower lobe, adult, donor1
UBERON:0001004	Respiratory system	UBERON:0000117	respiratory tube	CNhs12500	Fibroblast - Lung, donor1
UBERON:0001004	Respiratory system	UBERON:0000117	respiratory tube	CNhs11380	Fibroblast - Lung, donor2
UBERON:0001004	Respiratory system	UBERON:0000117	respiratory tube	CNhs12029	Fibroblast - Lung, donor3
UBERON:0001004	Respiratory system	UBERON:0000117	respiratory tube	CNhs12054	Bronchial Epithelial Cell, donor4
UBERON:0001004	Respiratory system	UBERON:0000117	respiratory tube	CNhs12058	Bronchial Epithelial Cell, donor5
UBERON:0001004	Respiratory system	UBERON:0000117	respiratory tube	CNhs12062	Bronchial Epithelial Cell, donor6
UBERON:0001004	Respiratory system	UBERON:0000117	respiratory tube	CNhs11325	Alveolar Epithelial Cells, donor1
UBERON:0001004	Respiratory system	UBERON:0000117	respiratory tube	CNhs11327	Bronchial Epithelial Cell, donor1
UBERON:0001004	Respiratory system	UBERON:0000117	respiratory tube	CNhs12085	Bronchial Epithelial Cell, donor2
UBERON:0001004	Respiratory system	UBERON:0000117	respiratory tube	CNhs12119	Alveolar Epithelial Cells, donor3
UBERON:0001004	Respiratory system	UBERON:0000117	respiratory tube	CNhs12623	Bronchial Epithelial Cell, donor3
UBERON:0001004	Respiratory system	UBERON:0000117	respiratory tube	CNhs12642	Bronchial Epithelial Cell, donor7
UBERON:0001004	Respiratory system	UBERON:0000166	oral opening	CNhs10654	tonsil, adult, pool1
UBERON:0001004	Respiratory system	UBERON:0000166	oral opening	CNhs11677	salivary gland, adult, pool1
UBERON:0001004	Respiratory system	UBERON:0000166	oral opening	CNhs12849	parotid gland, adult
UBERON:0001004	Respiratory system	UBERON:0000166	oral opening	CNhs12852	submaxillary gland, adult
UBERON:0001004	Respiratory system	UBERON:0000166	oral opening	CNhs11061	Gingival epithelial cells, donor1 (GEA11)
UBERON:0001004	Respiratory system	UBERON:0000166	oral opening	CNhs10848	Fibroblast - Gingival, donor4 (GFH2)
UBERON:0001004	Respiratory system	UBERON:0000166	oral opening	CNhs10866	Fibroblast - Gingival, donor1
UBERON:0001004	Respiratory system	UBERON:0000166	oral opening	CNhs11896	Gingival epithelial cells, donor2 (GEA14)
UBERON:0001004	Respiratory system	UBERON:0000166	oral opening	CNhs11952	Fibroblast - Gingival, donor5 (GFH3)
UBERON:0001004	Respiratory system	UBERON:0000166	oral opening	CNhs11961	Fibroblast - Gingival, donor2
UBERON:0001004	Respiratory system	UBERON:0000166	oral opening	CNhs11903	Gingival epithelial cells, donor3 (GEA15)
UBERON:0001004	Respiratory system	UBERON:0000166	oral opening	CNhs12006	Fibroblast - Gingival, donor3
UBERON:0001004	Respiratory system	UBERON:0000166	oral opening	CNhs12810	salivary acinar cells, donor1
UBERON:0001004	Respiratory system	UBERON:0000166	oral opening	CNhs12811	salivary acinar cells, donor2
UBERON:0001004	Respiratory system	UBERON:0000166	oral opening	CNhs12812	salivary acinar cells, donor3
UBERON:0001004	Respiratory system	UBERON:0000166	oral opening	CNhs14128	Fibroblast - Gingival, donor6 (aggressive periodontitis)
UBERON:0001004	Respiratory system	UBERON:0000166	oral opening	CNhs14129	Fibroblast - Gingival, donor6 (control)
UBERON:0001004	Respiratory system	UBERON:0000166	oral opening	CNhs14130	Fibroblast - Gingival, donor7 (aggressive periodontitis)
UBERON:0001004	Respiratory system	UBERON:0000166	oral opening	CNhs14131	Fibroblast - Gingival, donor7 (control)
UBERON:0001004	Respiratory system	UBERON:0000166	oral opening	CNhs14132	Fibroblast - Gingival, donor8 (chronic periodontitis)
UBERON:0001004	Respiratory system	UBERON:0000166	oral opening	CNhs14133	Fibroblast - Gingival, donor8 (control)

UBERON:0001004	Respiratory system	UBERON:0000166	oral opening	CNhs14134	Fibroblast - Gingival, donor9 (control)
UBERON:0001004	Respiratory system	UBERON:0000166	oral opening	CNhs14135	Fibroblast - Gingival, donor10 (periodontitis)
UBERON:0001004	Respiratory system	UBERON:0000171	respiration organ	CNhs10625	lung, adult, pool1
UBERON:0001004	Respiratory system	UBERON:0000171	respiration organ	CNhs11680	lung, fetal, donor1
UBERON:0001004	Respiratory system	UBERON:0000171	respiration organ	CNhs11786	lung, right lower lobe, adult, donor1
UBERON:0001004	Respiratory system	UBERON:0000171	respiration organ	CNhs12500	Fibroblast - Lung, donor1
UBERON:0001004	Respiratory system	UBERON:0000171	respiration organ	CNhs11380	Fibroblast - Lung, donor2
UBERON:0001004	Respiratory system	UBERON:0000171	respiration organ	CNhs12029	Fibroblast - Lung, donor3
UBERON:0001004	Respiratory system	UBERON:0000171	respiration organ	CNhs12054	Bronchial Epithelial Cell, donor4
UBERON:0001004	Respiratory system	UBERON:0000171	respiration organ	CNhs12058	Bronchial Epithelial Cell, donor5
UBERON:0001004	Respiratory system	UBERON:0000171	respiration organ	CNhs12062	Bronchial Epithelial Cell, donor6
UBERON:0001004	Respiratory system	UBERON:0000171	respiration organ	CNhs11325	Alveolar Epithelial Cells, donor1
UBERON:0001004	Respiratory system	UBERON:0000171	respiration organ	CNhs11327	Bronchial Epithelial Cell, donor1
UBERON:0001004	Respiratory system	UBERON:0000171	respiration organ	CNhs12085	Bronchial Epithelial Cell, donor2
UBERON:0001004	Respiratory system	UBERON:0000171	respiration organ	CNhs12119	Alveolar Epithelial Cells, donor3
UBERON:0001004	Respiratory system	UBERON:0000171	respiration organ	CNhs12623	Bronchial Epithelial Cell, donor3
UBERON:0001004	Respiratory system	UBERON:0000171	respiration organ	CNhs12642	Bronchial Epithelial Cell, donor7
UBERON:0001004	Respiratory system	UBERON:0001004	respiratory system	CNhs10625	lung, adult, pool1
UBERON:0001004	Respiratory system	UBERON:0001004	respiratory system	CNhs10635	trachea, adult, pool1
UBERON:0001004	Respiratory system	UBERON:0001004	respiratory system	CNhs10654	tonsil, adult, pool1
UBERON:0001004	Respiratory system	UBERON:0001004	respiratory system	CNhs11766	trachea, fetal, donor1
UBERON:0001004	Respiratory system	UBERON:0001004	respiratory system	CNhs11680	lung, fetal, donor1
UBERON:0001004	Respiratory system	UBERON:0001004	respiratory system	CNhs11779	diaphragm, fetal, donor1
UBERON:0001004	Respiratory system	UBERON:0001004	respiratory system	CNhs11786	lung, right lower lobe, adult, donor1
UBERON:0001004	Respiratory system	UBERON:0001004	respiratory system	CNhs11677	salivary gland, adult, pool1
UBERON:0001004	Respiratory system	UBERON:0001004	respiratory system	CNhs12849	parotid gland, adult
UBERON:0001004	Respiratory system	UBERON:0001004	respiratory system	CNhs12852	submaxillary gland, adult
UBERON:0001004	Respiratory system	UBERON:0001004	respiratory system	CNhs11061	Gingival epithelial cells, donor1 (GEA11)
UBERON:0001004	Respiratory system	UBERON:0001004	respiratory system	CNhs10848	Fibroblast - Gingival, donor4 (GFH2)
UBERON:0001004	Respiratory system	UBERON:0001004	respiratory system	CNhs10866	Fibroblast - Gingival, donor1
UBERON:0001004	Respiratory system	UBERON:0001004	respiratory system	CNhs10884	Small Airway Epithelial Cells, donor1
UBERON:0001004	Respiratory system	UBERON:0001004	respiratory system	CNhs12500	Fibroblast - Lung, donor1
UBERON:0001004	Respiratory system	UBERON:0001004	respiratory system	CNhs11092	Tracheal Epithelial Cells, donor1
UBERON:0001004	Respiratory system	UBERON:0001004	respiratory system	CNhs11896	Gingival epithelial cells, donor2 (GEA14)
UBERON:0001004	Respiratory system	UBERON:0001004	respiratory system	CNhs11952	Fibroblast - Gingival, donor5 (GFH3)
UBERON:0001004	Respiratory system	UBERON:0001004	respiratory system	CNhs11961	Fibroblast - Gingival, donor2
UBERON:0001004	Respiratory system	UBERON:0001004	respiratory system	CNhs11975	Small Airway Epithelial Cells, donor2
UBERON:0001004	Respiratory system	UBERON:0001004	respiratory system	CNhs11380	Fibroblast - Lung, donor2
UBERON:0001004	Respiratory system	UBERON:0001004	respiratory system	CNhs11993	Tracheal Epithelial Cells, donor2
UBERON:0001004	Respiratory system	UBERON:0001004	respiratory system	CNhs11903	Gingival epithelial cells, donor3 (GEA15)
UBERON:0001004	Respiratory system	UBERON:0001004	respiratory system	CNhs12006	Fibroblast - Gingival, donor3
UBERON:0001004	Respiratory system	UBERON:0001004	respiratory system	CNhs12016	Small Airway Epithelial Cells, donor3
UBERON:0001004	Respiratory system	UBERON:0001004	respiratory system	CNhs12029	Fibroblast - Lung, donor3
UBERON:0001004	Respiratory system	UBERON:0001004	respiratory system	CNhs12051	Tracheal Epithelial Cells, donor3
UBERON:0001004	Respiratory system	UBERON:0001004	respiratory system	CNhs12054	Bronchial Epithelial Cell, donor4
UBERON:0001004	Respiratory system	UBERON:0001004	respiratory system	CNhs12058	Bronchial Epithelial Cell, donor5
UBERON:0001004	Respiratory system	UBERON:0001004	respiratory system	CNhs12062	Bronchial Epithelial Cell, donor6
UBERON:0001004	Respiratory system	UBERON:0001004	respiratory system	CNhs11325	Alveolar Epithelial Cells, donor1
UBERON:0001004	Respiratory system	UBERON:0001004	respiratory system	CNhs11327	Bronchial Epithelial Cell, donor1
UBERON:0001004	Respiratory system	UBERON:0001004	respiratory system	CNhs11328	Smooth Muscle Cells - Bronchial, donor1
UBERON:0001004	Respiratory system	UBERON:0001004	respiratory system	CNhs11329	Smooth Muscle Cells - Tracheal, donor1
UBERON:0001004	Respiratory system	UBERON:0001004	respiratory system	CNhs12085	Bronchial Epithelial Cell, donor2
UBERON:0001004	Respiratory system	UBERON:0001004	respiratory system	CNhs12348	Smooth Muscle Cells - Bronchial, donor2
UBERON:0001004	Respiratory system	UBERON:0001004	respiratory system	CNhs12567	Smooth Muscle Cells - Tracheal, donor2
UBERON:0001004	Respiratory system	UBERON:0001004	respiratory system	CNhs12119	Alveolar Epithelial Cells, donor3
UBERON:0001004	Respiratory system	UBERON:0001004	respiratory system	CNhs12623	Bronchial Epithelial Cell, donor3
UBERON:0001004	Respiratory system	UBERON:0001004	respiratory system	CNhs12894	Smooth Muscle Cells - Tracheal, donor3
UBERON:0001004	Respiratory system	UBERON:0001004	respiratory system	CNhs12642	Bronchial Epithelial Cell, donor7
UBERON:0001004	Respiratory system	UBERON:0001004	respiratory system	CNhs12810	salivary acinar cells, donor1
UBERON:0001004	Respiratory system	UBERON:0001004	respiratory system	CNhs12811	salivary acinar cells, donor2
UBERON:0001004	Respiratory system	UBERON:0001004	respiratory system	CNhs12812	salivary acinar cells, donor3
UBERON:0001004	Respiratory system	UBERON:0001004	respiratory system	CNhs14128	Fibroblast - Gingival, donor6 (aggressive periodontitis)
UBERON:0001004	Respiratory system	UBERON:0001004	respiratory system	CNhs14129	Fibroblast - Gingival, donor6 (control)
UBERON:0001004	Respiratory system	UBERON:0001004	respiratory system	CNhs14130	Fibroblast - Gingival, donor7 (aggressive periodontitis)
UBERON:0001004	Respiratory system	UBERON:0001004	respiratory system	CNhs14131	Fibroblast - Gingival, donor7 (control)
UBERON:0001004	Respiratory system	UBERON:0001004	respiratory system	CNhs14132	Fibroblast - Gingival, donor8 (chronic periodontitis)
UBERON:0001004	Respiratory system	UBERON:0001004	respiratory system	CNhs14133	Fibroblast - Gingival, donor8 (control)
UBERON:0001004	Respiratory system	UBERON:0001004	respiratory system	CNhs14134	Fibroblast - Gingival, donor9 (control)
UBERON:0001004	Respiratory system	UBERON:0001004	respiratory system	CNhs14135	Fibroblast - Gingival, donor10 (periodontitis)
UBERON:0001004	Respiratory system	UBERON:0001004	respiratory system	CNhs14183	Smooth muscle cells - airway, asthmatic, donor1
UBERON:0001004	Respiratory system	UBERON:0001004	respiratory system	CNhs14184	Smooth muscle cells - airway, asthmatic, donor2
UBERON:0001004	Respiratory system	UBERON:0001004	respiratory system	CNhs14186	Smooth muscle cells - airway, asthmatic, donor3
UBERON:0001004	Respiratory system	UBERON:0001004	respiratory system	CNhs14187	Smooth muscle cells - airway, asthmatic, donor4
UBERON:0001004	Respiratory system	UBERON:0001004	respiratory system	CNhs14188	Smooth muscle cells - airway, asthmatic, donor5
UBERON:0001004	Respiratory system	UBERON:0001004	respiratory system	CNhs14189	Smooth muscle cells - airway, asthmatic, donor6
UBERON:0001004	Respiratory system	UBERON:0001004	respiratory system	CNhs14190	Smooth muscle cells - airway, control, donor1
UBERON:0001004	Respiratory system	UBERON:0001004	respiratory system	CNhs14191	Smooth muscle cells - airway, control, donor2
UBERON:0001004	Respiratory system	UBERON:0001004	respiratory system	CNhs14192	Smooth muscle cells - airway, control, donor3
UBERON:0001004	Respiratory system	UBERON:0001004	respiratory system	CNhs14193	Smooth muscle cells - airway, control, donor4
UBERON:0001004	Respiratory system	UBERON:0001005	respiratory airway	CNhs10635	trachea, adult, pool1
UBERON:0001004	Respiratory system	UBERON:0001005	respiratory airway	CNhs11766	trachea, fetal, donor1
UBERON:0001004	Respiratory system	UBERON:0001005	respiratory airway	CNhs11092	Tracheal Epithelial Cells, donor1
UBERON:0001004	Respiratory system	UBERON:0001005	respiratory airway	CNhs11993	Tracheal Epithelial Cells, donor2
UBERON:0001004	Respiratory system	UBERON:0001005	respiratory airway	CNhs12051	Tracheal Epithelial Cells, donor3
UBERON:0001004	Respiratory system	UBERON:0001005	respiratory airway	CNhs11329	Smooth Muscle Cells - Tracheal, donor1
UBERON:0001004	Respiratory system	UBERON:0001005	respiratory airway	CNhs12567	Smooth Muscle Cells - Tracheal, donor2
UBERON:0001004	Respiratory system	UBERON:0001005	respiratory airway	CNhs12894	Smooth Muscle Cells - Tracheal, donor3
UBERON:0001004	Respiratory system	UBERON:0001042	chordate pharynx	CNhs10654	tonsil, adult, pool1
UBERON:0001004	Respiratory system	UBERON:0001044	salivary gland	CNhs11677	salivary gland, adult, pool1
UBERON:0001004	Respiratory system	UBERON:0001044	salivary gland	CNhs12849	parotid gland, adult
UBERON:0001004	Respiratory system	UBERON:0001044	salivary gland	CNhs12852	submaxillary gland, adult
UBERON:0001004	Respiratory system	UBERON:0001044	salivary gland	CNhs12810	salivary acinar cells, donor1
UBERON:0001004	Respiratory system	UBERON:0001044	salivary gland	CNhs12811	salivary acinar cells, donor2
UBERON:0001004	Respiratory system	UBERON:0001044	salivary gland	CNhs12812	salivary acinar cells, donor3
UBERON:0001004	Respiratory system	UBERON:0001103	diaphragm	CNhs11779	diaphragm, fetal, donor1
UBERON:0001004	Respiratory system	UBERON:0001558	lower respiratory tract	CNhs10635	trachea, adult, pool1
UBERON:0001004	Respiratory system	UBERON:0001558	lower respiratory tract	CNhs11766	trachea, fetal, donor1
UBERON:0001004	Respiratory system	UBERON:0001558	lower respiratory tract	CNhs11092	Tracheal Epithelial Cells, donor1

UBERON:0001004	Respiratory system	UBERON:0001558	lower respiratory tract	CNhs11993	Tracheal Epithelial Cells, donor2
UBERON:0001004	Respiratory system	UBERON:0001558	lower respiratory tract	CNhs12051	Tracheal Epithelial Cells, donor3
UBERON:0001004	Respiratory system	UBERON:0001558	lower respiratory tract	CNhs12054	Bronchial Epithelial Cell, donor4
UBERON:0001004	Respiratory system	UBERON:0001558	lower respiratory tract	CNhs12058	Bronchial Epithelial Cell, donor5
UBERON:0001004	Respiratory system	UBERON:0001558	lower respiratory tract	CNhs12062	Bronchial Epithelial Cell, donor6
UBERON:0001004	Respiratory system	UBERON:0001558	lower respiratory tract	CNhs11325	Alveolar Epithelial Cells, donor1
UBERON:0001004	Respiratory system	UBERON:0001558	lower respiratory tract	CNhs11327	Bronchial Epithelial Cell, donor1
UBERON:0001004	Respiratory system	UBERON:0001558	lower respiratory tract	CNhs11328	Smooth Muscle Cells - Bronchial, donor1
UBERON:0001004	Respiratory system	UBERON:0001558	lower respiratory tract	CNhs11329	Smooth Muscle Cells - Tracheal, donor1
UBERON:0001004	Respiratory system	UBERON:0001558	lower respiratory tract	CNhs12085	Bronchial Epithelial Cell, donor2
UBERON:0001004	Respiratory system	UBERON:0001558	lower respiratory tract	CNhs12348	Smooth Muscle Cells - Bronchial, donor2
UBERON:0001004	Respiratory system	UBERON:0001558	lower respiratory tract	CNhs12567	Smooth Muscle Cells - Tracheal, donor2
UBERON:0001004	Respiratory system	UBERON:0001558	lower respiratory tract	CNhs12119	Alveolar Epithelial Cells, donor3
UBERON:0001004	Respiratory system	UBERON:0001558	lower respiratory tract	CNhs12623	Bronchial Epithelial Cell, donor3
UBERON:0001004	Respiratory system	UBERON:0001558	lower respiratory tract	CNhs12894	Smooth Muscle Cells - Tracheal, donor3
UBERON:0001004	Respiratory system	UBERON:0001558	lower respiratory tract	CNhs12642	Bronchial Epithelial Cell, donor7
UBERON:0001004	Respiratory system	UBERON:0001723	tongue	CNhs11768	tongue, fetal, donor1
UBERON:0001004	Respiratory system	UBERON:0001723	tongue	CNhs12853	tongue, adult
UBERON:0001004	Respiratory system	UBERON:0001723	tongue	CNhs13460	tongue epidermis (fungiform papillae), donor1
UBERON:0001004	Respiratory system	UBERON:0001726	papilla of tongue	CNhs13460	tongue epidermis (fungiform papillae), donor1
UBERON:0001004	Respiratory system	UBERON:0001735	tonsillar ring	CNhs10654	tonsil, adult, pool1
UBERON:0001004	Respiratory system	UBERON:0001736	submandibular gland	CNhs12852	submaxillary gland, adult
UBERON:0001004	Respiratory system	UBERON:0001828	gingiva	CNhs11061	Gingival epithelial cells, donor1 (GEA11)
UBERON:0001004	Respiratory system	UBERON:0001828	gingiva	CNhs10848	Fibroblast - Gingival, donor4 (GFH2)
UBERON:0001004	Respiratory system	UBERON:0001828	gingiva	CNhs10866	Fibroblast - Gingival, donor1
UBERON:0001004	Respiratory system	UBERON:0001828	gingiva	CNhs11896	Gingival epithelial cells, donor2 (GEA14)
UBERON:0001004	Respiratory system	UBERON:0001828	gingiva	CNhs11952	Fibroblast - Gingival, donor5 (GFH3)
UBERON:0001004	Respiratory system	UBERON:0001828	gingiva	CNhs11961	Fibroblast - Gingival, donor2
UBERON:0001004	Respiratory system	UBERON:0001828	gingiva	CNhs11903	Gingival epithelial cells, donor3 (GEA15)
UBERON:0001004	Respiratory system	UBERON:0001828	gingiva	CNhs12006	Fibroblast - Gingival, donor3
UBERON:0001004	Respiratory system	UBERON:0001828	gingiva	CNhs14128	Fibroblast - Gingival, donor6 (aggressive periodontitis)
UBERON:0001004	Respiratory system	UBERON:0001828	gingiva	CNhs14129	Fibroblast - Gingival, donor6 (control)
UBERON:0001004	Respiratory system	UBERON:0001828	gingiva	CNhs14130	Fibroblast - Gingival, donor7 (aggressive periodontitis)
UBERON:0001004	Respiratory system	UBERON:0001828	gingiva	CNhs14131	Fibroblast - Gingival, donor7 (control)
UBERON:0001004	Respiratory system	UBERON:0001828	gingiva	CNhs14132	Fibroblast - Gingival, donor8 (chronic periodontitis)
UBERON:0001004	Respiratory system	UBERON:0001828	gingiva	CNhs14133	Fibroblast - Gingival, donor8 (control)
UBERON:0001004	Respiratory system	UBERON:0001828	gingiva	CNhs14134	Fibroblast - Gingival, donor9 (control)
UBERON:0001004	Respiratory system	UBERON:0001828	gingiva	CNhs14135	Fibroblast - Gingival, donor10 (periodontitis)
UBERON:0001004	Respiratory system	UBERON:0001829	major salivary gland	CNhs12849	parotid gland, adult
UBERON:0001004	Respiratory system	UBERON:0001829	major salivary gland	CNhs12852	submaxillary gland, adult
UBERON:0001004	Respiratory system	UBERON:0001831	parotid gland	CNhs12849	parotid gland, adult
UBERON:0001004	Respiratory system	UBERON:0001949	gingival epithelium	CNhs11061	Gingival epithelial cells, donor1 (GEA11)
UBERON:0001004	Respiratory system	UBERON:0001949	gingival epithelium	CNhs11896	Gingival epithelial cells, donor2 (GEA14)
UBERON:0001004	Respiratory system	UBERON:0001949	gingival epithelium	CNhs11903	Gingival epithelial cells, donor3 (GEA15)
UBERON:0001004	Respiratory system	UBERON:0002012	pulmonary artery	CNhs10878	Fibroblast - Pulmonary Artery, donor1
UBERON:0001004	Respiratory system	UBERON:0002012	pulmonary artery	CNhs11089	Smooth Muscle Cells - Pulmonary Artery, donor1
UBERON:0001004	Respiratory system	UBERON:0002012	pulmonary artery	CNhs11989	Smooth Muscle Cells - Pulmonary Artery, donor2
UBERON:0001004	Respiratory system	UBERON:0002012	pulmonary artery	CNhs12047	Smooth Muscle Cells - Pulmonary Artery, donor3
UBERON:0001004	Respiratory system	UBERON:0002031	epithelium of bronchus	CNhs12054	Bronchial Epithelial Cell, donor4
UBERON:0001004	Respiratory system	UBERON:0002031	epithelium of bronchus	CNhs12058	Bronchial Epithelial Cell, donor5
UBERON:0001004	Respiratory system	UBERON:0002031	epithelium of bronchus	CNhs12062	Bronchial Epithelial Cell, donor6
UBERON:0001004	Respiratory system	UBERON:0002031	epithelium of bronchus	CNhs11327	Bronchial Epithelial Cell, donor1
UBERON:0001004	Respiratory system	UBERON:0002031	epithelium of bronchus	CNhs12085	Bronchial Epithelial Cell, donor2
UBERON:0001004	Respiratory system	UBERON:0002031	epithelium of bronchus	CNhs12623	Bronchial Epithelial Cell, donor3
UBERON:0001004	Respiratory system	UBERON:0002031	epithelium of bronchus	CNhs12642	Bronchial Epithelial Cell, donor7
UBERON:0001004	Respiratory system	UBERON:0002048	lung	CNhs10625	lung, adult, pool1
UBERON:0001004	Respiratory system	UBERON:0002048	lung	CNhs11680	lung, fetal, donor1
UBERON:0001004	Respiratory system	UBERON:0002048	lung	CNhs11786	lung, right lower lobe, adult, donor1
UBERON:0001004	Respiratory system	UBERON:0002048	lung	CNhs12500	Fibroblast - Lung, donor1
UBERON:0001004	Respiratory system	UBERON:0002048	lung	CNhs11380	Fibroblast - Lung, donor2
UBERON:0001004	Respiratory system	UBERON:0002048	lung	CNhs12029	Fibroblast - Lung, donor3
UBERON:0001004	Respiratory system	UBERON:0002048	lung	CNhs12054	Bronchial Epithelial Cell, donor4
UBERON:0001004	Respiratory system	UBERON:0002048	lung	CNhs12058	Bronchial Epithelial Cell, donor5
UBERON:0001004	Respiratory system	UBERON:0002048	lung	CNhs12062	Bronchial Epithelial Cell, donor6
UBERON:0001004	Respiratory system	UBERON:0002048	lung	CNhs11325	Alveolar Epithelial Cells, donor1
UBERON:0001004	Respiratory system	UBERON:0002048	lung	CNhs11327	Bronchial Epithelial Cell, donor1
UBERON:0001004	Respiratory system	UBERON:0002048	lung	CNhs12085	Bronchial Epithelial Cell, donor2
UBERON:0001004	Respiratory system	UBERON:0002048	lung	CNhs12119	Alveolar Epithelial Cells, donor3
UBERON:0001004	Respiratory system	UBERON:0002048	lung	CNhs12623	Bronchial Epithelial Cell, donor3
UBERON:0001004	Respiratory system	UBERON:0002048	lung	CNhs12642	Bronchial Epithelial Cell, donor7
UBERON:0001004	Respiratory system	UBERON:0002167	right lung	CNhs11786	lung, right lower lobe, adult, donor1
UBERON:0001004	Respiratory system	UBERON:0002169	alveolar sac	CNhs11325	Alveolar Epithelial Cells, donor1
UBERON:0001004	Respiratory system	UBERON:0002169	alveolar sac	CNhs12119	Alveolar Epithelial Cells, donor3
UBERON:0001004	Respiratory system	UBERON:0002171	lower lobe of right lung	CNhs11786	lung, right lower lobe, adult, donor1
UBERON:0001004	Respiratory system	UBERON:0002185	bronchus	CNhs12054	Bronchial Epithelial Cell, donor4
UBERON:0001004	Respiratory system	UBERON:0002185	bronchus	CNhs12058	Bronchial Epithelial Cell, donor5
UBERON:0001004	Respiratory system	UBERON:0002185	bronchus	CNhs12062	Bronchial Epithelial Cell, donor6
UBERON:0001004	Respiratory system	UBERON:0002185	bronchus	CNhs11327	Bronchial Epithelial Cell, donor1
UBERON:0001004	Respiratory system	UBERON:0002185	bronchus	CNhs11328	Smooth Muscle Cells - Bronchial, donor1
UBERON:0001004	Respiratory system	UBERON:0002185	bronchus	CNhs12085	Bronchial Epithelial Cell, donor2
UBERON:0001004	Respiratory system	UBERON:0002185	bronchus	CNhs12348	Smooth Muscle Cells - Bronchial, donor2
UBERON:0001004	Respiratory system	UBERON:0002185	bronchus	CNhs12623	Bronchial Epithelial Cell, donor3
UBERON:0001004	Respiratory system	UBERON:0002185	bronchus	CNhs12642	Bronchial Epithelial Cell, donor7
UBERON:0001004	Respiratory system	UBERON:0002299	alveolus of lung	CNhs11325	Alveolar Epithelial Cells, donor1
UBERON:0001004	Respiratory system	UBERON:0002299	alveolus of lung	CNhs12119	Alveolar Epithelial Cells, donor3
UBERON:0001004	Respiratory system	UBERON:0002370	thymus	CNhs10633	thymus, adult, pool1
UBERON:0001004	Respiratory system	UBERON:0002370	thymus	CNhs10650	thymus, fetal, pool1
UBERON:0001004	Respiratory system	UBERON:0002372	tonsil	CNhs10654	tonsil, adult, pool1
UBERON:0001004	Respiratory system	UBERON:0002448	fungiform papilla	CNhs13460	tongue epidermis (fungiform papillae), donor1
UBERON:0001004	Respiratory system	UBERON:0003126	trachea	CNhs10635	trachea, adult, pool1
UBERON:0001004	Respiratory system	UBERON:0003126	trachea	CNhs11766	trachea, fetal, donor1
UBERON:0001004	Respiratory system	UBERON:0003126	trachea	CNhs11092	Tracheal Epithelial Cells, donor1
UBERON:0001004	Respiratory system	UBERON:0003126	trachea	CNhs11993	Tracheal Epithelial Cells, donor2
UBERON:0001004	Respiratory system	UBERON:0003126	trachea	CNhs12051	Tracheal Epithelial Cells, donor3
UBERON:0001004	Respiratory system	UBERON:0003126	trachea	CNhs11329	Smooth Muscle Cells - Tracheal, donor1
UBERON:0001004	Respiratory system	UBERON:0003126	trachea	CNhs12567	Smooth Muscle Cells - Tracheal, donor2
UBERON:0001004	Respiratory system	UBERON:0003126	trachea	CNhs12894	Smooth Muscle Cells - Tracheal, donor3
UBERON:0001004	Respiratory system	UBERON:0003127	open tracheal system trachea	CNhs10635	trachea, adult, pool1

UBERON:0001004	Respiratory system	UBERON:0003127	open tracheal system trachea	CNhs11766	trachea, fetal, donor1
UBERON:0001004	Respiratory system	UBERON:0003293	gland of oral region	CNhs11677	salivary gland, adult, pool1
UBERON:0001004	Respiratory system	UBERON:0003293	gland of oral region	CNhs12849	parotid gland, adult
UBERON:0001004	Respiratory system	UBERON:0003293	gland of oral region	CNhs12852	submaxillary gland, adult
UBERON:0001004	Respiratory system	UBERON:0003293	gland of oral region	CNhs12810	salivary acinar cells, donor1
UBERON:0001004	Respiratory system	UBERON:0003293	gland of oral region	CNhs12811	salivary acinar cells, donor2
UBERON:0001004	Respiratory system	UBERON:0003293	gland of oral region	CNhs12812	salivary acinar cells, donor3
UBERON:0001004	Respiratory system	UBERON:0003831	respiratory system muscle	CNhs11779	diaphragm, fetal, donor1
UBERON:0001004	Respiratory system	UBERON:0004225	respiratory system smooth muscle	CNhs14183	Smooth muscle cells - airway, asthmatic, donor1
UBERON:0001004	Respiratory system	UBERON:0004225	respiratory system smooth muscle	CNhs14184	Smooth muscle cells - airway, asthmatic, donor2
UBERON:0001004	Respiratory system	UBERON:0004225	respiratory system smooth muscle	CNhs14186	Smooth muscle cells - airway, asthmatic, donor3
UBERON:0001004	Respiratory system	UBERON:0004225	respiratory system smooth muscle	CNhs14187	Smooth muscle cells - airway, asthmatic, donor4
UBERON:0001004	Respiratory system	UBERON:0004225	respiratory system smooth muscle	CNhs14188	Smooth muscle cells - airway, asthmatic, donor5
UBERON:0001004	Respiratory system	UBERON:0004225	respiratory system smooth muscle	CNhs14189	Smooth muscle cells - airway, asthmatic, donor6
UBERON:0001004	Respiratory system	UBERON:0004225	respiratory system smooth muscle	CNhs14190	Smooth muscle cells - airway, control, donor1
UBERON:0001004	Respiratory system	UBERON:0004225	respiratory system smooth muscle	CNhs14191	Smooth muscle cells - airway, control, donor2
UBERON:0001004	Respiratory system	UBERON:0004225	respiratory system smooth muscle	CNhs14192	Smooth muscle cells - airway, control, donor3
UBERON:0001004	Respiratory system	UBERON:0004225	respiratory system smooth muscle	CNhs14193	Smooth muscle cells - airway, control, donor4
UBERON:0001004	Respiratory system	UBERON:0004802	respiratory tract epithelium	CNhs12054	Bronchial Epithelial Cell, donor4
UBERON:0001004	Respiratory system	UBERON:0004802	respiratory tract epithelium	CNhs12058	Bronchial Epithelial Cell, donor5
UBERON:0001004	Respiratory system	UBERON:0004802	respiratory tract epithelium	CNhs12062	Bronchial Epithelial Cell, donor6
UBERON:0001004	Respiratory system	UBERON:0004802	respiratory tract epithelium	CNhs11325	Alveolar Epithelial Cells, donor1
UBERON:0001004	Respiratory system	UBERON:0004802	respiratory tract epithelium	CNhs11327	Bronchial Epithelial Cell, donor1
UBERON:0001004	Respiratory system	UBERON:0004802	respiratory tract epithelium	CNhs12085	Bronchial Epithelial Cell, donor2
UBERON:0001004	Respiratory system	UBERON:0004802	respiratory tract epithelium	CNhs12119	Alveolar Epithelial Cells, donor3
UBERON:0001004	Respiratory system	UBERON:0004802	respiratory tract epithelium	CNhs12623	Bronchial Epithelial Cell, donor3
UBERON:0001004	Respiratory system	UBERON:0004802	respiratory tract epithelium	CNhs12642	Bronchial Epithelial Cell, donor7
UBERON:0001004	Respiratory system	UBERON:0004807	respiratory system epithelium	CNhs11061	Gingival epithelial cells, donor1 (GEA11)
UBERON:0001004	Respiratory system	UBERON:0004807	respiratory system epithelium	CNhs11896	Gingival epithelial cells, donor2 (GEA14)
UBERON:0001004	Respiratory system	UBERON:0004807	respiratory system epithelium	CNhs11903	Gingival epithelial cells, donor3 (GEA15)
UBERON:0001004	Respiratory system	UBERON:0004807	respiratory system epithelium	CNhs12054	Bronchial Epithelial Cell, donor4
UBERON:0001004	Respiratory system	UBERON:0004807	respiratory system epithelium	CNhs12058	Bronchial Epithelial Cell, donor5
UBERON:0001004	Respiratory system	UBERON:0004807	respiratory system epithelium	CNhs12062	Bronchial Epithelial Cell, donor6
UBERON:0001004	Respiratory system	UBERON:0004807	respiratory system epithelium	CNhs11325	Alveolar Epithelial Cells, donor1
UBERON:0001004	Respiratory system	UBERON:0004807	respiratory system epithelium	CNhs11327	Bronchial Epithelial Cell, donor1
UBERON:0001004	Respiratory system	UBERON:0004807	respiratory system epithelium	CNhs12085	Bronchial Epithelial Cell, donor2
UBERON:0001004	Respiratory system	UBERON:0004807	respiratory system epithelium	CNhs12119	Alveolar Epithelial Cells, donor3
UBERON:0001004	Respiratory system	UBERON:0004807	respiratory system epithelium	CNhs12623	Bronchial Epithelial Cell, donor3
UBERON:0001004	Respiratory system	UBERON:0004807	respiratory system epithelium	CNhs12642	Bronchial Epithelial Cell, donor7
UBERON:0001004	Respiratory system	UBERON:0004815	lower respiratory tract epithelium	CNhs12054	Bronchial Epithelial Cell, donor4
UBERON:0001004	Respiratory system	UBERON:0004815	lower respiratory tract epithelium	CNhs12058	Bronchial Epithelial Cell, donor5
UBERON:0001004	Respiratory system	UBERON:0004815	lower respiratory tract epithelium	CNhs12062	Bronchial Epithelial Cell, donor6
UBERON:0001004	Respiratory system	UBERON:0004815	lower respiratory tract epithelium	CNhs11327	Bronchial Epithelial Cell, donor1
UBERON:0001004	Respiratory system	UBERON:0004815	lower respiratory tract epithelium	CNhs12085	Bronchial Epithelial Cell, donor2
UBERON:0001004	Respiratory system	UBERON:0004815	lower respiratory tract epithelium	CNhs12623	Bronchial Epithelial Cell, donor3
UBERON:0001004	Respiratory system	UBERON:0004815	lower respiratory tract epithelium	CNhs12642	Bronchial Epithelial Cell, donor7
UBERON:0001004	Respiratory system	UBERON:0004821	pulmonary alveolus epithelium	CNhs11325	Alveolar Epithelial Cells, donor1
UBERON:0001004	Respiratory system	UBERON:0004821	pulmonary alveolus epithelium	CNhs12119	Alveolar Epithelial Cells, donor3
UBERON:0001004	Respiratory system	UBERON:0004830	respiratory system skeletal muscle	CNhs11779	diaphragm, fetal, donor1
UBERON:0001004	Respiratory system	UBERON:0004894	alveolar wall	CNhs11325	Alveolar Epithelial Cells, donor1
UBERON:0001004	Respiratory system	UBERON:0004894	alveolar wall	CNhs12119	Alveolar Epithelial Cells, donor3
UBERON:0001004	Respiratory system	UBERON:0006518	right lung lobe	CNhs11786	lung, right lower lobe, adult, donor1
UBERON:0001004	Respiratory system	UBERON:0006524	alveolar system	CNhs11325	Alveolar Epithelial Cells, donor1
UBERON:0001004	Respiratory system	UBERON:0006524	alveolar system	CNhs12119	Alveolar Epithelial Cells, donor3
UBERON:0001004	Respiratory system	UBERON:0007196	tracheobronchial tree	CNhs10635	trachea, adult, pool1
UBERON:0001004	Respiratory system	UBERON:0007196	tracheobronchial tree	CNhs11766	trachea, fetal, donor1
UBERON:0001004	Respiratory system	UBERON:0007196	tracheobronchial tree	CNhs11092	Tracheal Epithelial Cells, donor1
UBERON:0001004	Respiratory system	UBERON:0007196	tracheobronchial tree	CNhs11993	Tracheal Epithelial Cells, donor2
UBERON:0001004	Respiratory system	UBERON:0007196	tracheobronchial tree	CNhs12051	Tracheal Epithelial Cells, donor3
UBERON:0001004	Respiratory system	UBERON:0007196	tracheobronchial tree	CNhs12054	Bronchial Epithelial Cell, donor4
UBERON:0001004	Respiratory system	UBERON:0007196	tracheobronchial tree	CNhs12058	Bronchial Epithelial Cell, donor5
UBERON:0001004	Respiratory system	UBERON:0007196	tracheobronchial tree	CNhs12062	Bronchial Epithelial Cell, donor6
UBERON:0001004	Respiratory system	UBERON:0007196	tracheobronchial tree	CNhs11325	Alveolar Epithelial Cells, donor1
UBERON:0001004	Respiratory system	UBERON:0007196	tracheobronchial tree	CNhs11327	Bronchial Epithelial Cell, donor1
UBERON:0001004	Respiratory system	UBERON:0007196	tracheobronchial tree	CNhs11328	Smooth Muscle Cells - Bronchial, donor1
UBERON:0001004	Respiratory system	UBERON:0007196	tracheobronchial tree	CNhs11329	Smooth Muscle Cells - Tracheal, donor1
UBERON:0001004	Respiratory system	UBERON:0007196	tracheobronchial tree	CNhs12085	Bronchial Epithelial Cell, donor2
UBERON:0001004	Respiratory system	UBERON:0007196	tracheobronchial tree	CNhs12348	Smooth Muscle Cells - Bronchial, donor2
UBERON:0001004	Respiratory system	UBERON:0007196	tracheobronchial tree	CNhs12567	Smooth Muscle Cells - Tracheal, donor2
UBERON:0001004	Respiratory system	UBERON:0007196	tracheobronchial tree	CNhs12119	Alveolar Epithelial Cells, donor3
UBERON:0001004	Respiratory system	UBERON:0007196	tracheobronchial tree	CNhs12623	Bronchial Epithelial Cell, donor3
UBERON:0001004	Respiratory system	UBERON:0007196	tracheobronchial tree	CNhs12894	Smooth Muscle Cells - Tracheal, donor3
UBERON:0001004	Respiratory system	UBERON:0007196	tracheobronchial tree	CNhs12642	Bronchial Epithelial Cell, donor7
UBERON:0001004	Respiratory system	UBERON:0008874	pulmonary acinus	CNhs11325	Alveolar Epithelial Cells, donor1
UBERON:0001004	Respiratory system	UBERON:0008874	pulmonary acinus	CNhs12119	Alveolar Epithelial Cells, donor3
UBERON:0001004	Respiratory system	UBERON:0008949	lower lobe of lung	CNhs11786	lung, right lower lobe, adult, donor1
UBERON:0001004	Respiratory system	UBERON:0009471	dorsum of tongue	CNhs13460	tongue epidermis (fungiform papillae), donor1
UBERON:0001004	Respiratory system	UBERON:0010368	pulmonary lobule	CNhs11325	Alveolar Epithelial Cells, donor1
UBERON:0001004	Respiratory system	UBERON:0010368	pulmonary lobule	CNhs12119	Alveolar Epithelial Cells, donor3
UBERON:0001007	Digestive System	UBERON:0000057	urethra	CNhs13464	Urethra, donor2
UBERON:0001007	Digestive System	UBERON:0000059	large intestine	CNhs10619	colon, adult, pool1
UBERON:0001007	Digestive System	UBERON:0000059	large intestine	CNhs11777	rectum, fetal, donor1
UBERON:0001007	Digestive System	UBERON:0000059	large intestine	CNhs11780	colon, fetal, donor1
UBERON:0001007	Digestive System	UBERON:0000059	large intestine	CNhs11794	colon, adult, donor1
UBERON:0001007	Digestive System	UBERON:0000059	large intestine	CNhs12842	appendix, adult
UBERON:0001007	Digestive System	UBERON:0000059	large intestine	CNhs10868	Smooth Muscle Cells - Colonic, donor1
UBERON:0001007	Digestive System	UBERON:0000059	large intestine	CNhs11963	Smooth Muscle Cells - Colonic, donor2
UBERON:0001007	Digestive System	UBERON:0000059	large intestine	CNhs12007	Smooth Muscle Cells - Colonic, donor3
UBERON:0001007	Digestive System	UBERON:0000101	lobe of lung	CNhs11786	lung, right lower lobe, adult, donor1
UBERON:0001007	Digestive System	UBERON:0000115	lung epithelium	CNhs12054	Bronchial Epithelial Cell, donor4
UBERON:0001007	Digestive System	UBERON:0000115	lung epithelium	CNhs12058	Bronchial Epithelial Cell, donor5
UBERON:0001007	Digestive System	UBERON:0000115	lung epithelium	CNhs12062	Bronchial Epithelial Cell, donor6
UBERON:0001007	Digestive System	UBERON:0000115	lung epithelium	CNhs11325	Alveolar Epithelial Cells, donor1
UBERON:0001007	Digestive System	UBERON:0000115	lung epithelium	CNhs11327	Bronchial Epithelial Cell, donor1
UBERON:0001007	Digestive System	UBERON:0000115	lung epithelium	CNhs12085	Bronchial Epithelial Cell, donor2
UBERON:0001007	Digestive System	UBERON:0000115	lung epithelium	CNhs12119	Alveolar Epithelial Cells, donor3
UBERON:0001007	Digestive System	UBERON:0000115	lung epithelium	CNhs12623	Bronchial Epithelial Cell, donor3
UBERON:0001007	Digestive System	UBERON:0000115	lung epithelium	CNhs12642	Bronchial Epithelial Cell, donor7

UBERON:0001007	Digestive System	UBERON:0000160	intestine	CNhs10619	colon, adult, pool1
UBERON:0001007	Digestive System	UBERON:0000160	intestine	CNhs10630	small intestine, adult, pool1
UBERON:0001007	Digestive System	UBERON:0000160	intestine	Cnhs11772	temporal lobe, fetal, donor1
UBERON:0001007	Digestive System	UBERON:0000160	intestine	CNhs11773	small intestine, fetal, donor1
UBERON:0001007	Digestive System	UBERON:0000160	intestine	CNhs11777	rectum, fetal, donor1
UBERON:0001007	Digestive System	UBERON:0000160	intestine	CNhs11780	colon, fetal, donor1
UBERON:0001007	Digestive System	UBERON:0000160	intestine	CNhs11794	colon, adult, donor1
UBERON:0001007	Digestive System	UBERON:0000160	intestine	CNhs12842	appendix, adult
UBERON:0001007	Digestive System	UBERON:0000160	intestine	CNhs10868	Smooth Muscle Cells - Colonic, donor1
UBERON:0001007	Digestive System	UBERON:0000160	intestine	CNhs10875	Intestinal epithelial cells (polarized), donor1
UBERON:0001007	Digestive System	UBERON:0000160	intestine	CNhs11963	Smooth Muscle Cells - Colonic, donor2
UBERON:0001007	Digestive System	UBERON:0000160	intestine	CNhs12007	Smooth Muscle Cells - Colonic, donor3
UBERON:0001007	Digestive System	UBERON:0000160	intestine	CNhs12595	Smooth Muscle Cells - Intestinal, donor1
UBERON:0001007	Digestive System	UBERON:0000165	mouth	CNhs10654	tonsil, adult, pool1
UBERON:0001007	Digestive System	UBERON:0000165	mouth	CNhs11768	tongue, fetal, donor1
UBERON:0001007	Digestive System	UBERON:0000165	mouth	CNhs11677	salivary gland, adult, pool1
UBERON:0001007	Digestive System	UBERON:0000165	mouth	CNhs12849	parotid gland, adult
UBERON:0001007	Digestive System	UBERON:0000165	mouth	CNhs12852	submaxillary gland, adult
UBERON:0001007	Digestive System	UBERON:0000165	mouth	CNhs12853	tongue, adult
UBERON:0001007	Digestive System	UBERON:0000165	mouth	CNhs13460	tongue epidermis (fungiform papillae), donor1
UBERON:0001007	Digestive System	UBERON:0000165	mouth	CNhs11061	Gingival epithelial cells, donor1 (GEA11)
UBERON:0001007	Digestive System	UBERON:0000165	mouth	CNhs10848	Fibroblast - Gingival, donor4 (GFH2)
UBERON:0001007	Digestive System	UBERON:0000165	mouth	CNhs12493	Fibroblast - Periodontal Ligament, donor4 (PL29)
UBERON:0001007	Digestive System	UBERON:0000165	mouth	CNhs10866	Fibroblast - Gingival, donor1
UBERON:0001007	Digestive System	UBERON:0000165	mouth	CNhs10867	Fibroblast - Periodontal Ligament, donor1
UBERON:0001007	Digestive System	UBERON:0000165	mouth	CNhs10879	Keratinocyte - oral, donor1
UBERON:0001007	Digestive System	UBERON:0000165	mouth	CNhs11896	Gingival epithelial cells, donor2 (GEA14)
UBERON:0001007	Digestive System	UBERON:0000165	mouth	CNhs11952	Fibroblast - Gingival, donor5 (GFH3)
UBERON:0001007	Digestive System	UBERON:0000165	mouth	CNhs11953	Fibroblast - Periodontal Ligament, donor5 (PL30)
UBERON:0001007	Digestive System	UBERON:0000165	mouth	CNhs11961	Fibroblast - Gingival, donor2
UBERON:0001007	Digestive System	UBERON:0000165	mouth	CNhs11962	Fibroblast - Periodontal Ligament, donor2
UBERON:0001007	Digestive System	UBERON:0000165	mouth	CNhs11903	Gingival epithelial cells, donor3 (GEA15)
UBERON:0001007	Digestive System	UBERON:0000165	mouth	CNhs11996	Fibroblast - Periodontal Ligament, donor6 (PLH3)
UBERON:0001007	Digestive System	UBERON:0000165	mouth	CNhs12006	Fibroblast - Gingival, donor3
UBERON:0001007	Digestive System	UBERON:0000165	mouth	CNhs11907	Fibroblast - Periodontal Ligament, donor3
UBERON:0001007	Digestive System	UBERON:0000165	mouth	CNhs12810	salivary acinar cells, donor1
UBERON:0001007	Digestive System	UBERON:0000165	mouth	CNhs12811	salivary acinar cells, donor2
UBERON:0001007	Digestive System	UBERON:0000165	mouth	CNhs12812	salivary acinar cells, donor3
UBERON:0001007	Digestive System	UBERON:0000165	mouth	CNhs14128	Fibroblast - Gingival, donor6 (aggressive periodontitis)
UBERON:0001007	Digestive System	UBERON:0000165	mouth	CNhs14129	Fibroblast - Gingival, donor6 (control)
UBERON:0001007	Digestive System	UBERON:0000165	mouth	CNhs14130	Fibroblast - Gingival, donor7 (aggressive periodontitis)
UBERON:0001007	Digestive System	UBERON:0000165	mouth	CNhs14131	Fibroblast - Gingival, donor7 (control)
UBERON:0001007	Digestive System	UBERON:0000165	mouth	CNhs14132	Fibroblast - Gingival, donor8 (chronic periodontitis)
UBERON:0001007	Digestive System	UBERON:0000165	mouth	CNhs14133	Fibroblast - Gingival, donor8 (control)
UBERON:0001007	Digestive System	UBERON:0000165	mouth	CNhs14134	Fibroblast - Gingival, donor9 (control)
UBERON:0001007	Digestive System	UBERON:0000165	mouth	CNhs14135	Fibroblast - Gingival, donor10 (periodontitis)
UBERON:0001007	Digestive System	UBERON:0000166	oral opening	CNhs10654	tonsil, adult, pool1
UBERON:0001007	Digestive System	UBERON:0000166	oral opening	CNhs11677	salivary gland, adult, pool1
UBERON:0001007	Digestive System	UBERON:0000166	oral opening	CNhs12849	parotid gland, adult
UBERON:0001007	Digestive System	UBERON:0000166	oral opening	CNhs12852	submaxillary gland, adult
UBERON:0001007	Digestive System	UBERON:0000166	oral opening	CNhs11061	Gingival epithelial cells, donor1 (GEA11)
UBERON:0001007	Digestive System	UBERON:0000166	oral opening	CNhs10848	Fibroblast - Gingival, donor4 (GFH2)
UBERON:0001007	Digestive System	UBERON:0000166	oral opening	CNhs10866	Fibroblast - Gingival, donor1
UBERON:0001007	Digestive System	UBERON:0000166	oral opening	CNhs11896	Gingival epithelial cells, donor2 (GEA14)
UBERON:0001007	Digestive System	UBERON:0000166	oral opening	CNhs11952	Fibroblast - Gingival, donor5 (GFH3)
UBERON:0001007	Digestive System	UBERON:0000166	oral opening	CNhs11961	Fibroblast - Gingival, donor2
UBERON:0001007	Digestive System	UBERON:0000166	oral opening	CNhs11903	Gingival epithelial cells, donor3 (GEA15)
UBERON:0001007	Digestive System	UBERON:0000166	oral opening	CNhs12006	Fibroblast - Gingival, donor3
UBERON:0001007	Digestive System	UBERON:0000166	oral opening	CNhs12810	salivary acinar cells, donor1
UBERON:0001007	Digestive System	UBERON:0000166	oral opening	CNhs12811	salivary acinar cells, donor2
UBERON:0001007	Digestive System	UBERON:0000166	oral opening	CNhs12812	salivary acinar cells, donor3
UBERON:0001007	Digestive System	UBERON:0000166	oral opening	CNhs14128	Fibroblast - Gingival, donor6 (aggressive periodontitis)
UBERON:0001007	Digestive System	UBERON:0000166	oral opening	CNhs14129	Fibroblast - Gingival, donor6 (control)
UBERON:0001007	Digestive System	UBERON:0000166	oral opening	CNhs14130	Fibroblast - Gingival, donor7 (aggressive periodontitis)
UBERON:0001007	Digestive System	UBERON:0000166	oral opening	CNhs14131	Fibroblast - Gingival, donor7 (control)
UBERON:0001007	Digestive System	UBERON:0000166	oral opening	CNhs14132	Fibroblast - Gingival, donor8 (chronic periodontitis)
UBERON:0001007	Digestive System	UBERON:0000166	oral opening	CNhs14133	Fibroblast - Gingival, donor8 (control)
UBERON:0001007	Digestive System	UBERON:0000166	oral opening	CNhs14134	Fibroblast - Gingival, donor9 (control)
UBERON:0001007	Digestive System	UBERON:0000166	oral opening	CNhs14135	Fibroblast - Gingival, donor10 (periodontitis)
UBERON:0001007	Digestive System	UBERON:0000167	oral cavity	CNhs11677	salivary gland, adult, pool1
UBERON:0001007	Digestive System	UBERON:0000167	oral cavity	CNhs12849	parotid gland, adult
UBERON:0001007	Digestive System	UBERON:0000167	oral cavity	CNhs12852	submaxillary gland, adult
UBERON:0001007	Digestive System	UBERON:0000167	oral cavity	CNhs12810	salivary acinar cells, donor1
UBERON:0001007	Digestive System	UBERON:0000167	oral cavity	CNhs12811	salivary acinar cells, donor2
UBERON:0001007	Digestive System	UBERON:0000167	oral cavity	CNhs12812	salivary acinar cells, donor3
UBERON:0001007	Digestive System	UBERON:0000945	stomach	CNhs11771	stomach, fetal, donor1
UBERON:0001007	Digestive System	UBERON:0000996	vagina	CNhs12854	vagina, adult
UBERON:0001007	Digestive System	UBERON:0001007	digestive system	CNhs10619	colon, adult, pool1
UBERON:0001007	Digestive System	UBERON:0001007	digestive system	CNhs10620	esophagus, adult, pool1
UBERON:0001007	Digestive System	UBERON:0001007	digestive system	CNhs10624	liver, adult, pool1
UBERON:0001007	Digestive System	UBERON:0001007	digestive system	CNhs10630	small intestine, adult, pool1
UBERON:0001007	Digestive System	UBERON:0001007	digestive system	CNhs10631	spleen, adult, pool1
UBERON:0001007	Digestive System	UBERON:0001007	digestive system	CNhs10651	spleen, fetal, pool1
UBERON:0001007	Digestive System	UBERON:0001007	digestive system	CNhs10654	tonsil, adult, pool1
UBERON:0001007	Digestive System	UBERON:0001007	digestive system	CNhs11768	tongue, fetal, donor1
UBERON:0001007	Digestive System	UBERON:0001007	digestive system	CNhs11771	stomach, fetal, donor1
UBERON:0001007	Digestive System	UBERON:0001007	digestive system	Cnhs11772	temporal lobe, fetal, donor1
UBERON:0001007	Digestive System	UBERON:0001007	digestive system	CNhs11773	small intestine, fetal, donor1
UBERON:0001007	Digestive System	UBERON:0001007	digestive system	CNhs11777	rectum, fetal, donor1
UBERON:0001007	Digestive System	UBERON:0001007	digestive system	CNhs11780	colon, fetal, donor1
UBERON:0001007	Digestive System	UBERON:0001007	digestive system	CNhs11794	colon, adult, donor1
UBERON:0001007	Digestive System	UBERON:0001007	digestive system	CNhs11798	liver, fetal, pool1
UBERON:0001007	Digestive System	UBERON:0001007	digestive system	CNhs11677	salivary gland, adult, pool1
UBERON:0001007	Digestive System	UBERON:0001007	digestive system	CNhs12842	appendix, adult
UBERON:0001007	Digestive System	UBERON:0001007	digestive system	CNhs12848	gall bladder, adult
UBERON:0001007	Digestive System	UBERON:0001007	digestive system	CNhs12849	parotid gland, adult
UBERON:0001007	Digestive System	UBERON:0001007	digestive system	CNhs12852	submaxillary gland, adult
UBERON:0001007	Digestive System	UBERON:0001007	digestive system	CNhs12853	tongue, adult

UBERON:0001007	Digestive System	UBERON:0001007	digestive system	CNhs13460	tongue epidermis (fungiform papillae), donor1
UBERON:0001007	Digestive System	UBERON:0001007	digestive system	CNhs10845	Mesenchymal stem cells - hepatic, donor0
UBERON:0001007	Digestive System	UBERON:0001007	digestive system	CNhs11061	Gingival epithelial cells, donor1 (GEA11)
UBERON:0001007	Digestive System	UBERON:0001007	digestive system	CNhs10848	Fibroblast - Gingival, donor4 (GFH2)
UBERON:0001007	Digestive System	UBERON:0001007	digestive system	CNhs12493	Fibroblast - Periodontal Ligament, donor4 (PL29)
UBERON:0001007	Digestive System	UBERON:0001007	digestive system	CNhs10866	Fibroblast - Gingival, donor1
UBERON:0001007	Digestive System	UBERON:0001007	digestive system	CNhs10867	Fibroblast - Periodontal Ligament, donor1
UBERON:0001007	Digestive System	UBERON:0001007	digestive system	CNhs10868	Smooth Muscle Cells - Colonic, donor1
UBERON:0001007	Digestive System	UBERON:0001007	digestive system	CNhs10875	Intestinal epithelial cells (polarized), donor1
UBERON:0001007	Digestive System	UBERON:0001007	digestive system	CNhs10879	Keratinocyte - oral, donor1
UBERON:0001007	Digestive System	UBERON:0001007	digestive system	CNhs11896	Gingival epithelial cells, donor2 (GEA14)
UBERON:0001007	Digestive System	UBERON:0001007	digestive system	CNhs11952	Fibroblast - Gingival, donor5 (GFH3)
UBERON:0001007	Digestive System	UBERON:0001007	digestive system	CNhs11953	Fibroblast - Periodontal Ligament, donor5 (PL30)
UBERON:0001007	Digestive System	UBERON:0001007	digestive system	CNhs11961	Fibroblast - Gingival, donor2
UBERON:0001007	Digestive System	UBERON:0001007	digestive system	CNhs11962	Fibroblast - Periodontal Ligament, donor2
UBERON:0001007	Digestive System	UBERON:0001007	digestive system	CNhs11963	Smooth Muscle Cells - Colonic, donor2
UBERON:0001007	Digestive System	UBERON:0001007	digestive system	CNhs11903	Gingival epithelial cells, donor3 (GEA15)
UBERON:0001007	Digestive System	UBERON:0001007	digestive system	CNhs11996	Fibroblast - Periodontal Ligament, donor6 (PLH3)
UBERON:0001007	Digestive System	UBERON:0001007	digestive system	CNhs12006	Fibroblast - Gingival, donor3
UBERON:0001007	Digestive System	UBERON:0001007	digestive system	CNhs11907	Fibroblast - Periodontal Ligament, donor3
UBERON:0001007	Digestive System	UBERON:0001007	digestive system	CNhs12007	Smooth Muscle Cells - Colonic, donor3
UBERON:0001007	Digestive System	UBERON:0001007	digestive system	CNhs11323	Esophageal Epithelial Cells, donor1
UBERON:0001007	Digestive System	UBERON:0001007	digestive system	CNhs11324	Smooth Muscle Cells - Esophageal, donor1
UBERON:0001007	Digestive System	UBERON:0001007	digestive system	CNhs12595	Smooth Muscle Cells - Intestinal, donor1
UBERON:0001007	Digestive System	UBERON:0001007	digestive system	CNhs12075	Hepatic Sinusoidal Endothelial Cells, donor1
UBERON:0001007	Digestive System	UBERON:0001007	digestive system	CNhs12340	Hepatocyte, donor1
UBERON:0001007	Digestive System	UBERON:0001007	digestive system	CNhs11335	Hepatic Stellate Cells (lipocyte), donor1
UBERON:0001007	Digestive System	UBERON:0001007	digestive system	CNhs11346	Mesenchymal Stem Cells - hepatic, donor1
UBERON:0001007	Digestive System	UBERON:0001007	digestive system	CNhs12083	Esophageal Epithelial Cells, donor2
UBERON:0001007	Digestive System	UBERON:0001007	digestive system	CNhs12727	Smooth Muscle Cells - Esophageal, donor2
UBERON:0001007	Digestive System	UBERON:0001007	digestive system	CNhs12092	Hepatic Sinusoidal Endothelial Cells, donor2
UBERON:0001007	Digestive System	UBERON:0001007	digestive system	CNhs12349	Hepatocyte, donor2
UBERON:0001007	Digestive System	UBERON:0001007	digestive system	CNhs12093	Hepatic Stellate Cells (lipocyte), donor2
UBERON:0001007	Digestive System	UBERON:0001007	digestive system	CNhs12730	Mesenchymal Stem Cells - hepatic, donor2
UBERON:0001007	Digestive System	UBERON:0001007	digestive system	CNhs12622	Esophageal Epithelial Cells, donor3
UBERON:0001007	Digestive System	UBERON:0001007	digestive system	CNhs12625	Hepatic Sinusoidal Endothelial Cells, donor3
UBERON:0001007	Digestive System	UBERON:0001007	digestive system	CNhs12626	Hepatocyte, donor3
UBERON:0001007	Digestive System	UBERON:0001007	digestive system	CNhs12627	Hepatic Stellate Cells (lipocyte), donor3
UBERON:0001007	Digestive System	UBERON:0001007	digestive system	CNhs12810	salivary acinar cells, donor1
UBERON:0001007	Digestive System	UBERON:0001007	digestive system	CNhs12811	salivary acinar cells, donor2
UBERON:0001007	Digestive System	UBERON:0001007	digestive system	CNhs12812	salivary acinar cells, donor3
UBERON:0001007	Digestive System	UBERON:0001007	digestive system	CNhs14128	Fibroblast - Gingival, donor6 (aggressive periodontitis)
UBERON:0001007	Digestive System	UBERON:0001007	digestive system	CNhs14129	Fibroblast - Gingival, donor6 (control)
UBERON:0001007	Digestive System	UBERON:0001007	digestive system	CNhs14130	Fibroblast - Gingival, donor7 (aggressive periodontitis)
UBERON:0001007	Digestive System	UBERON:0001007	digestive system	CNhs14131	Fibroblast - Gingival, donor7 (control)
UBERON:0001007	Digestive System	UBERON:0001007	digestive system	CNhs14132	Fibroblast - Gingival, donor8 (chronic periodontitis)
UBERON:0001007	Digestive System	UBERON:0001007	digestive system	CNhs14133	Fibroblast - Gingival, donor8 (control)
UBERON:0001007	Digestive System	UBERON:0001007	digestive system	CNhs14134	Fibroblast - Gingival, donor9 (control)
UBERON:0001007	Digestive System	UBERON:0001007	digestive system	CNhs14135	Fibroblast - Gingival, donor10 (periodontitis)
UBERON:0001007	Digestive System	UBERON:0001041	foregut	CNhs10620	esophagus, adult, pool1
UBERON:0001007	Digestive System	UBERON:0001041	foregut	CNhs11771	stomach, fetal, donor1
UBERON:0001007	Digestive System	UBERON:0001041	foregut	CNhs11677	salivary gland, adult, pool1
UBERON:0001007	Digestive System	UBERON:0001041	foregut	CNhs12849	parotid gland, adult
UBERON:0001007	Digestive System	UBERON:0001041	foregut	CNhs12852	submaxillary gland, adult
UBERON:0001007	Digestive System	UBERON:0001041	foregut	CNhs11323	Esophageal Epithelial Cells, donor1
UBERON:0001007	Digestive System	UBERON:0001041	foregut	CNhs11324	Smooth Muscle Cells - Esophageal, donor1
UBERON:0001007	Digestive System	UBERON:0001041	foregut	CNhs12083	Esophageal Epithelial Cells, donor2
UBERON:0001007	Digestive System	UBERON:0001041	foregut	CNhs12727	Smooth Muscle Cells - Esophageal, donor2
UBERON:0001007	Digestive System	UBERON:0001041	foregut	CNhs12622	Esophageal Epithelial Cells, donor3
UBERON:0001007	Digestive System	UBERON:0001041	foregut	CNhs12810	salivary acinar cells, donor1
UBERON:0001007	Digestive System	UBERON:0001041	foregut	CNhs12811	salivary acinar cells, donor2
UBERON:0001007	Digestive System	UBERON:0001041	foregut	CNhs12812	salivary acinar cells, donor3
UBERON:0001007	Digestive System	UBERON:0001042	chordate pharynx	CNhs10654	tonsil, adult, pool1
UBERON:0001007	Digestive System	UBERON:0001043	esophagus	CNhs10620	esophagus, adult, pool1
UBERON:0001007	Digestive System	UBERON:0001043	esophagus	CNhs11323	Esophageal Epithelial Cells, donor1
UBERON:0001007	Digestive System	UBERON:0001043	esophagus	CNhs11324	Smooth Muscle Cells - Esophageal, donor1
UBERON:0001007	Digestive System	UBERON:0001043	esophagus	CNhs12083	Esophageal Epithelial Cells, donor2
UBERON:0001007	Digestive System	UBERON:0001043	esophagus	CNhs12727	Smooth Muscle Cells - Esophageal, donor2
UBERON:0001007	Digestive System	UBERON:0001043	esophagus	CNhs12622	Esophageal Epithelial Cells, donor3
UBERON:0001007	Digestive System	UBERON:0001044	salivary gland	CNhs11677	salivary gland, adult, pool1
UBERON:0001007	Digestive System	UBERON:0001044	salivary gland	CNhs12849	parotid gland, adult
UBERON:0001007	Digestive System	UBERON:0001044	salivary gland	CNhs12852	submaxillary gland, adult
UBERON:0001007	Digestive System	UBERON:0001044	salivary gland	CNhs12810	salivary acinar cells, donor1
UBERON:0001007	Digestive System	UBERON:0001044	salivary gland	CNhs12811	salivary acinar cells, donor2
UBERON:0001007	Digestive System	UBERON:0001044	salivary gland	CNhs12812	salivary acinar cells, donor3
UBERON:0001007	Digestive System	UBERON:0001052	rectum	CNhs11777	rectum, fetal, donor1
UBERON:0001007	Digestive System	UBERON:0001153	caecum	CNhs12842	appendix, adult
UBERON:0001007	Digestive System	UBERON:0001154	appendix	CNhs12842	appendix, adult
UBERON:0001007	Digestive System	UBERON:0001155	colon	CNhs10619	colon, adult, pool1
UBERON:0001007	Digestive System	UBERON:0001155	colon	CNhs11780	colon, fetal, donor1
UBERON:0001007	Digestive System	UBERON:0001155	colon	CNhs11794	colon, adult, donor1
UBERON:0001007	Digestive System	UBERON:0001155	colon	CNhs12842	appendix, adult
UBERON:0001007	Digestive System	UBERON:0001155	colon	CNhs10868	Smooth Muscle Cells - Colonic, donor1
UBERON:0001007	Digestive System	UBERON:0001155	colon	CNhs11963	Smooth Muscle Cells - Colonic, donor2
UBERON:0001007	Digestive System	UBERON:0001155	colon	CNhs12007	Smooth Muscle Cells - Colonic, donor3
UBERON:0001007	Digestive System	UBERON:0001173	biliary tree	CNhs12848	gall bladder, adult
UBERON:0001007	Digestive System	UBERON:0001242	intestinal mucosa	CNhs10875	Intestinal epithelial cells (polarized), donor1
UBERON:0001007	Digestive System	UBERON:0001255	urinary bladder	CNhs10616	bladder, adult, pool1
UBERON:0001007	Digestive System	UBERON:0001255	urinary bladder	CNhs12893	Smooth Muscle Cells - Bladder, donor1
UBERON:0001007	Digestive System	UBERON:0001262	wall of intestine	CNhs10875	Intestinal epithelial cells (polarized), donor1
UBERON:0001007	Digestive System	UBERON:0001277	intestinal epithelium	CNhs10875	Intestinal epithelial cells (polarized), donor1
UBERON:0001007	Digestive System	UBERON:0001280	liver parenchyma	CNhs12075	Hepatic Sinusoidal Endothelial Cells, donor1
UBERON:0001007	Digestive System	UBERON:0001280	liver parenchyma	CNhs12092	Hepatic Sinusoidal Endothelial Cells, donor2
UBERON:0001007	Digestive System	UBERON:0001280	liver parenchyma	CNhs12625	Hepatic Sinusoidal Endothelial Cells, donor3
UBERON:0001007	Digestive System	UBERON:0001281	hepatic sinusoid	CNhs12075	Hepatic Sinusoidal Endothelial Cells, donor1
UBERON:0001007	Digestive System	UBERON:0001281	hepatic sinusoid	CNhs12092	Hepatic Sinusoidal Endothelial Cells, donor2
UBERON:0001007	Digestive System	UBERON:0001281	hepatic sinusoid	CNhs12625	Hepatic Sinusoidal Endothelial Cells, donor3
UBERON:0001007	Digestive System	UBERON:0001555	digestive tract	CNhs10619	colon, adult, pool1

UBERON:0001007	Digestive System	UBERON:0001555	digestive tract	CNhs10620	esophagus, adult, pool1
UBERON:0001007	Digestive System	UBERON:0001555	digestive tract	CNhs10630	small intestine, adult, pool1
UBERON:0001007	Digestive System	UBERON:0001555	digestive tract	CNhs10654	tonsil, adult, pool1
UBERON:0001007	Digestive System	UBERON:0001555	digestive tract	CNhs11768	tongue, fetal, donor1
UBERON:0001007	Digestive System	UBERON:0001555	digestive tract	CNhs11771	stomach, fetal, donor1
UBERON:0001007	Digestive System	UBERON:0001555	digestive tract	Cnhs11772	temporal lobe, fetal, donor1
UBERON:0001007	Digestive System	UBERON:0001555	digestive tract	CNhs11773	small intestine, fetal, donor1
UBERON:0001007	Digestive System	UBERON:0001555	digestive tract	CNhs11777	rectum, fetal, donor1
UBERON:0001007	Digestive System	UBERON:0001555	digestive tract	CNhs11780	colon, fetal, donor1
UBERON:0001007	Digestive System	UBERON:0001555	digestive tract	CNhs11794	colon, adult, donor1
UBERON:0001007	Digestive System	UBERON:0001555	digestive tract	CNhs11677	salivary gland, adult, pool1
UBERON:0001007	Digestive System	UBERON:0001555	digestive tract	CNhs12842	appendix, adult
UBERON:0001007	Digestive System	UBERON:0001555	digestive tract	CNhs12849	parotid gland, adult
UBERON:0001007	Digestive System	UBERON:0001555	digestive tract	CNhs12852	submaxillary gland, adult
UBERON:0001007	Digestive System	UBERON:0001555	digestive tract	CNhs12853	tongue, adult
UBERON:0001007	Digestive System	UBERON:0001555	digestive tract	CNhs13460	tongue epidermis (fungiform papillae), donor1
UBERON:0001007	Digestive System	UBERON:0001555	digestive tract	CNhs11061	Gingival epithelial cells, donor1 (GEA11)
UBERON:0001007	Digestive System	UBERON:0001555	digestive tract	CNhs10848	Fibroblast - Gingival, donor4 (GFH2)
UBERON:0001007	Digestive System	UBERON:0001555	digestive tract	CNhs12493	Fibroblast - Periodontal Ligament, donor4 (PL29)
UBERON:0001007	Digestive System	UBERON:0001555	digestive tract	CNhs10866	Fibroblast - Gingival, donor1
UBERON:0001007	Digestive System	UBERON:0001555	digestive tract	CNhs10867	Fibroblast - Periodontal Ligament, donor1
UBERON:0001007	Digestive System	UBERON:0001555	digestive tract	CNhs10868	Smooth Muscle Cells - Colonic, donor1
UBERON:0001007	Digestive System	UBERON:0001555	digestive tract	CNhs10875	Intestinal epithelial cells (polarized), donor1
UBERON:0001007	Digestive System	UBERON:0001555	digestive tract	CNhs10879	Keratinocyte - oral, donor1
UBERON:0001007	Digestive System	UBERON:0001555	digestive tract	CNhs11896	Gingival epithelial cells, donor2 (GEA14)
UBERON:0001007	Digestive System	UBERON:0001555	digestive tract	CNhs11952	Fibroblast - Gingival, donor5 (GFH3)
UBERON:0001007	Digestive System	UBERON:0001555	digestive tract	CNhs11953	Fibroblast - Periodontal Ligament, donor5 (PL30)
UBERON:0001007	Digestive System	UBERON:0001555	digestive tract	CNhs11961	Fibroblast - Gingival, donor2
UBERON:0001007	Digestive System	UBERON:0001555	digestive tract	CNhs11962	Fibroblast - Periodontal Ligament, donor2
UBERON:0001007	Digestive System	UBERON:0001555	digestive tract	CNhs11963	Smooth Muscle Cells - Colonic, donor2
UBERON:0001007	Digestive System	UBERON:0001555	digestive tract	CNhs11903	Gingival epithelial cells, donor3 (GEA15)
UBERON:0001007	Digestive System	UBERON:0001555	digestive tract	CNhs11996	Fibroblast - Periodontal Ligament, donor6 (PLH3)
UBERON:0001007	Digestive System	UBERON:0001555	digestive tract	CNhs12006	Fibroblast - Gingival, donor3
UBERON:0001007	Digestive System	UBERON:0001555	digestive tract	CNhs11907	Fibroblast - Periodontal Ligament, donor3
UBERON:0001007	Digestive System	UBERON:0001555	digestive tract	CNhs12007	Smooth Muscle Cells - Colonic, donor3
UBERON:0001007	Digestive System	UBERON:0001555	digestive tract	CNhs11323	Esophageal Epithelial Cells, donor1
UBERON:0001007	Digestive System	UBERON:0001555	digestive tract	CNhs11324	Smooth Muscle Cells - Esophageal, donor1
UBERON:0001007	Digestive System	UBERON:0001555	digestive tract	CNhs12595	Smooth Muscle Cells - Intestinal, donor1
UBERON:0001007	Digestive System	UBERON:0001555	digestive tract	CNhs12083	Esophageal Epithelial Cells, donor2
UBERON:0001007	Digestive System	UBERON:0001555	digestive tract	CNhs12727	Smooth Muscle Cells - Esophageal, donor2
UBERON:0001007	Digestive System	UBERON:0001555	digestive tract	CNhs12622	Esophageal Epithelial Cells, donor3
UBERON:0001007	Digestive System	UBERON:0001555	digestive tract	CNhs12810	salivary acinar cells, donor1
UBERON:0001007	Digestive System	UBERON:0001555	digestive tract	CNhs12811	salivary acinar cells, donor2
UBERON:0001007	Digestive System	UBERON:0001555	digestive tract	CNhs12812	salivary acinar cells, donor3
UBERON:0001007	Digestive System	UBERON:0001555	digestive tract	CNhs14128	Fibroblast - Gingival, donor6 (aggressive periodontitis)
UBERON:0001007	Digestive System	UBERON:0001555	digestive tract	CNhs14129	Fibroblast - Gingival, donor6 (control)
UBERON:0001007	Digestive System	UBERON:0001555	digestive tract	CNhs14130	Fibroblast - Gingival, donor7 (aggressive periodontitis)
UBERON:0001007	Digestive System	UBERON:0001555	digestive tract	CNhs14131	Fibroblast - Gingival, donor7 (control)
UBERON:0001007	Digestive System	UBERON:0001555	digestive tract	CNhs14132	Fibroblast - Gingival, donor8 (chronic periodontitis)
UBERON:0001007	Digestive System	UBERON:0001555	digestive tract	CNhs14133	Fibroblast - Gingival, donor8 (control)
UBERON:0001007	Digestive System	UBERON:0001555	digestive tract	CNhs14134	Fibroblast - Gingival, donor9 (control)
UBERON:0001007	Digestive System	UBERON:0001555	digestive tract	CNhs14135	Fibroblast - Gingival, donor10 (periodontitis)
UBERON:0001007	Digestive System	UBERON:0001723	tongue	CNhs11768	tongue, fetal, donor1
UBERON:0001007	Digestive System	UBERON:0001723	tongue	CNhs12853	tongue, adult
UBERON:0001007	Digestive System	UBERON:0001723	tongue	CNhs13460	tongue epidermis (fungiform papillae), donor1
UBERON:0001007	Digestive System	UBERON:0001726	papilla of tongue	CNhs13460	tongue epidermis (fungiform papillae), donor1
UBERON:0001007	Digestive System	UBERON:0001735	tonsillar ring	CNhs10654	tonsil, adult, pool1
UBERON:0001007	Digestive System	UBERON:0001736	submandibular gland	CNhs12852	submaxillary gland, adult
UBERON:0001007	Digestive System	UBERON:0001758	periodontium	CNhs12493	Fibroblast - Periodontal Ligament, donor4 (PL29)
UBERON:0001007	Digestive System	UBERON:0001758	periodontium	CNhs10867	Fibroblast - Periodontal Ligament, donor1
UBERON:0001007	Digestive System	UBERON:0001758	periodontium	CNhs11953	Fibroblast - Periodontal Ligament, donor5 (PL30)
UBERON:0001007	Digestive System	UBERON:0001758	periodontium	CNhs11962	Fibroblast - Periodontal Ligament, donor2
UBERON:0001007	Digestive System	UBERON:0001758	periodontium	CNhs11996	Fibroblast - Periodontal Ligament, donor6 (PLH3)
UBERON:0001007	Digestive System	UBERON:0001758	periodontium	CNhs11907	Fibroblast - Periodontal Ligament, donor3
UBERON:0001007	Digestive System	UBERON:0001828	gingiva	CNhs11061	Gingival epithelial cells, donor1 (GEA11)
UBERON:0001007	Digestive System	UBERON:0001828	gingiva	CNhs10848	Fibroblast - Gingival, donor4 (GFH2)
UBERON:0001007	Digestive System	UBERON:0001828	gingiva	CNhs10866	Fibroblast - Gingival, donor1
UBERON:0001007	Digestive System	UBERON:0001828	gingiva	CNhs11896	Gingival epithelial cells, donor2 (GEA14)
UBERON:0001007	Digestive System	UBERON:0001828	gingiva	CNhs11952	Fibroblast - Gingival, donor5 (GFH3)
UBERON:0001007	Digestive System	UBERON:0001828	gingiva	CNhs11961	Fibroblast - Gingival, donor2
UBERON:0001007	Digestive System	UBERON:0001828	gingiva	CNhs11903	Gingival epithelial cells, donor3 (GEA15)
UBERON:0001007	Digestive System	UBERON:0001828	gingiva	CNhs12006	Fibroblast - Gingival, donor3
UBERON:0001007	Digestive System	UBERON:0001828	gingiva	CNhs14128	Fibroblast - Gingival, donor6 (aggressive periodontitis)
UBERON:0001007	Digestive System	UBERON:0001828	gingiva	CNhs14129	Fibroblast - Gingival, donor6 (control)
UBERON:0001007	Digestive System	UBERON:0001828	gingiva	CNhs14130	Fibroblast - Gingival, donor7 (aggressive periodontitis)
UBERON:0001007	Digestive System	UBERON:0001828	gingiva	CNhs14131	Fibroblast - Gingival, donor7 (control)
UBERON:0001007	Digestive System	UBERON:0001828	gingiva	CNhs14132	Fibroblast - Gingival, donor8 (chronic periodontitis)
UBERON:0001007	Digestive System	UBERON:0001828	gingiva	CNhs14133	Fibroblast - Gingival, donor8 (control)
UBERON:0001007	Digestive System	UBERON:0001828	gingiva	CNhs14134	Fibroblast - Gingival, donor9 (control)
UBERON:0001007	Digestive System	UBERON:0001828	gingiva	CNhs14135	Fibroblast - Gingival, donor10 (periodontitis)
UBERON:0001007	Digestive System	UBERON:0001829	major salivary gland	CNhs12849	parotid gland, adult
UBERON:0001007	Digestive System	UBERON:0001829	major salivary gland	CNhs12852	submaxillary gland, adult
UBERON:0001007	Digestive System	UBERON:0001831	parotid gland	CNhs12849	parotid gland, adult
UBERON:0001007	Digestive System	UBERON:0001949	gingival epithelium	CNhs11061	Gingival epithelial cells, donor1 (GEA11)
UBERON:0001007	Digestive System	UBERON:0001949	gingival epithelium	CNhs11896	Gingival epithelial cells, donor2 (GEA14)
UBERON:0001007	Digestive System	UBERON:0001949	gingival epithelium	CNhs11903	Gingival epithelial cells, donor3 (GEA15)
UBERON:0001007	Digestive System	UBERON:0002012	pulmonary artery	CNhs10878	Fibroblast - Pulmonary Artery, donor1
UBERON:0001007	Digestive System	UBERON:0002012	pulmonary artery	CNhs11089	Smooth Muscle Cells - Pulmonary Artery, donor1
UBERON:0001007	Digestive System	UBERON:0002012	pulmonary artery	CNhs11989	Smooth Muscle Cells - Pulmonary Artery, donor2
UBERON:0001007	Digestive System	UBERON:0002012	pulmonary artery	CNhs12047	Smooth Muscle Cells - Pulmonary Artery, donor3
UBERON:0001007	Digestive System	UBERON:0002046	thyroid gland	CNhs10634	thyroid, adult, pool1
UBERON:0001007	Digestive System	UBERON:0002046	thyroid gland	CNhs11769	thyroid, fetal, donor1
UBERON:0001007	Digestive System	UBERON:0002048	lung	CNhs10625	lung, adult, pool1
UBERON:0001007	Digestive System	UBERON:0002048	lung	CNhs11680	lung, fetal, donor1
UBERON:0001007	Digestive System	UBERON:0002048	lung	CNhs11786	lung, right lower lobe, adult, donor1
UBERON:0001007	Digestive System	UBERON:0002048	lung	CNhs12500	Fibroblast - Lung, donor1
UBERON:0001007	Digestive System	UBERON:0002048	lung	CNhs11380	Fibroblast - Lung, donor2
UBERON:0001007	Digestive System	UBERON:0002048	lung	CNhs12029	Fibroblast - Lung, donor3

UBERON:0001007	Digestive System	UBERON:0002048	lung	CNhs12054	Bronchial Epithelial Cell, donor4
UBERON:0001007	Digestive System	UBERON:0002048	lung	CNhs12058	Bronchial Epithelial Cell, donor5
UBERON:0001007	Digestive System	UBERON:0002048	lung	CNhs12062	Bronchial Epithelial Cell, donor6
UBERON:0001007	Digestive System	UBERON:0002048	lung	CNhs11325	Alveolar Epithelial Cells, donor1
UBERON:0001007	Digestive System	UBERON:0002048	lung	CNhs11327	Bronchial Epithelial Cell, donor1
UBERON:0001007	Digestive System	UBERON:0002048	lung	CNhs12085	Bronchial Epithelial Cell, donor2
UBERON:0001007	Digestive System	UBERON:0002048	lung	CNhs12119	Alveolar Epithelial Cells, donor3
UBERON:0001007	Digestive System	UBERON:0002048	lung	CNhs12623	Bronchial Epithelial Cell, donor3
UBERON:0001007	Digestive System	UBERON:0002048	lung	CNhs12642	Bronchial Epithelial Cell, donor7
UBERON:0001007	Digestive System	UBERON:0002106	spleen	CNhs10631	spleen, adult, pool1
UBERON:0001007	Digestive System	UBERON:0002106	spleen	CNhs10651	spleen, fetal, pool1
UBERON:0001007	Digestive System	UBERON:0002107	liver	CNhs10624	liver, adult, pool1
UBERON:0001007	Digestive System	UBERON:0002107	liver	CNhs11798	liver, fetal, pool1
UBERON:0001007	Digestive System	UBERON:0002107	liver	CNhs10845	Mesenchymal stem cells - hepatic, donor0
UBERON:0001007	Digestive System	UBERON:0002107	liver	CNhs12075	Hepatic Sinusoidal Endothelial Cells, donor1
UBERON:0001007	Digestive System	UBERON:0002107	liver	CNhs12340	Hepatocyte, donor1
UBERON:0001007	Digestive System	UBERON:0002107	liver	CNhs11335	Hepatic Stellate Cells (lipocyte), donor1
UBERON:0001007	Digestive System	UBERON:0002107	liver	CNhs11346	Mesenchymal Stem Cells - hepatic, donor1
UBERON:0001007	Digestive System	UBERON:0002107	liver	CNhs12092	Hepatic Sinusoidal Endothelial Cells, donor2
UBERON:0001007	Digestive System	UBERON:0002107	liver	CNhs12349	Hepatocyte, donor2
UBERON:0001007	Digestive System	UBERON:0002107	liver	CNhs12093	Hepatic Stellate Cells (lipocyte), donor2
UBERON:0001007	Digestive System	UBERON:0002107	liver	CNhs12730	Mesenchymal Stem Cells - hepatic, donor2
UBERON:0001007	Digestive System	UBERON:0002107	liver	CNhs12625	Hepatic Sinusoidal Endothelial Cells, donor3
UBERON:0001007	Digestive System	UBERON:0002107	liver	CNhs12626	Hepatocyte, donor3
UBERON:0001007	Digestive System	UBERON:0002107	liver	CNhs12627	Hepatic Stellate Cells (lipocyte), donor3
UBERON:0001007	Digestive System	UBERON:0002108	small intestine	CNhs10630	small intestine, adult, pool1
UBERON:0001007	Digestive System	UBERON:0002108	small intestine	Cnhs11772	temporal lobe, fetal, donor1
UBERON:0001007	Digestive System	UBERON:0002108	small intestine	CNhs11773	small intestine, fetal, donor1
UBERON:0001007	Digestive System	UBERON:0002110	gallbladder	CNhs12848	gall bladder, adult
UBERON:0001007	Digestive System	UBERON:0002114	duodenum	Cnhs11772	temporal lobe, fetal, donor1
UBERON:0001007	Digestive System	UBERON:0002167	right lung	CNhs11786	lung, right lower lobe, adult, donor1
UBERON:0001007	Digestive System	UBERON:0002169	alveolar sac	CNhs11325	Alveolar Epithelial Cells, donor1
UBERON:0001007	Digestive System	UBERON:0002169	alveolar sac	CNhs12119	Alveolar Epithelial Cells, donor3
UBERON:0001007	Digestive System	UBERON:0002171	lower lobe of right lung	CNhs11786	lung, right lower lobe, adult, donor1
UBERON:0001007	Digestive System	UBERON:0002294	biliary system	CNhs12848	gall bladder, adult
UBERON:0001007	Digestive System	UBERON:0002299	alveolus of lung	CNhs11325	Alveolar Epithelial Cells, donor1
UBERON:0001007	Digestive System	UBERON:0002299	alveolus of lung	CNhs12119	Alveolar Epithelial Cells, donor3
UBERON:0001007	Digestive System	UBERON:0002367	prostate gland	CNhs10628	prostate, adult, pool1
UBERON:0001007	Digestive System	UBERON:0002367	prostate gland	CNhs10882	Prostate Epithelial Cells (polarized), donor1
UBERON:0001007	Digestive System	UBERON:0002367	prostate gland	CNhs10883	Prostate Stromal Cells, donor1
UBERON:0001007	Digestive System	UBERON:0002367	prostate gland	CNhs11920	Smooth Muscle Cells - Prostate, donor1
UBERON:0001007	Digestive System	UBERON:0002367	prostate gland	CNhs11972	Prostate Epithelial Cells, donor2
UBERON:0001007	Digestive System	UBERON:0002367	prostate gland	CNhs11973	Prostate Stromal Cells, donor2
UBERON:0001007	Digestive System	UBERON:0002367	prostate gland	CNhs11976	Smooth Muscle Cells - Prostate, donor2
UBERON:0001007	Digestive System	UBERON:0002367	prostate gland	CNhs12014	Prostate Epithelial Cells, donor3
UBERON:0001007	Digestive System	UBERON:0002367	prostate gland	CNhs12015	Prostate Stromal Cells, donor3
UBERON:0001007	Digestive System	UBERON:0002367	prostate gland	CNhs11910	Smooth Muscle Cells - Prostate, donor3
UBERON:0001007	Digestive System	UBERON:0002370	thymus	CNhs10633	thymus, adult, pool1
UBERON:0001007	Digestive System	UBERON:0002370	thymus	CNhs10650	thymus, fetal, pool1
UBERON:0001007	Digestive System	UBERON:0002372	tonsil	CNhs10654	tonsil, adult, pool1
UBERON:0001007	Digestive System	UBERON:0002423	hepatobiliary system	CNhs10624	liver, adult, pool1
UBERON:0001007	Digestive System	UBERON:0002423	hepatobiliary system	CNhs11798	liver, fetal, pool1
UBERON:0001007	Digestive System	UBERON:0002423	hepatobiliary system	CNhs12848	gall bladder, adult
UBERON:0001007	Digestive System	UBERON:0002423	hepatobiliary system	CNhs10845	Mesenchymal stem cells - hepatic, donor0
UBERON:0001007	Digestive System	UBERON:0002423	hepatobiliary system	CNhs12075	Hepatic Sinusoidal Endothelial Cells, donor1
UBERON:0001007	Digestive System	UBERON:0002423	hepatobiliary system	CNhs12340	Hepatocyte, donor1
UBERON:0001007	Digestive System	UBERON:0002423	hepatobiliary system	CNhs11335	Hepatic Stellate Cells (lipocyte), donor1
UBERON:0001007	Digestive System	UBERON:0002423	hepatobiliary system	CNhs11346	Mesenchymal Stem Cells - hepatic, donor1
UBERON:0001007	Digestive System	UBERON:0002423	hepatobiliary system	CNhs12092	Hepatic Sinusoidal Endothelial Cells, donor2
UBERON:0001007	Digestive System	UBERON:0002423	hepatobiliary system	CNhs12349	Hepatocyte, donor2
UBERON:0001007	Digestive System	UBERON:0002423	hepatobiliary system	CNhs12093	Hepatic Stellate Cells (lipocyte), donor2
UBERON:0001007	Digestive System	UBERON:0002423	hepatobiliary system	CNhs12730	Mesenchymal Stem Cells - hepatic, donor2
UBERON:0001007	Digestive System	UBERON:0002423	hepatobiliary system	CNhs12625	Hepatic Sinusoidal Endothelial Cells, donor3
UBERON:0001007	Digestive System	UBERON:0002423	hepatobiliary system	CNhs12626	Hepatocyte, donor3
UBERON:0001007	Digestive System	UBERON:0002423	hepatobiliary system	CNhs12627	Hepatic Stellate Cells (lipocyte), donor3
UBERON:0001007	Digestive System	UBERON:0002448	fungiform papilla	CNhs13460	tongue epidermis (fungiform papillae), donor1
UBERON:0001007	Digestive System	UBERON:0003126	trachea	CNhs10635	trachea, adult, pool1
UBERON:0001007	Digestive System	UBERON:0003126	trachea	CNhs11766	trachea, fetal, donor1
UBERON:0001007	Digestive System	UBERON:0003126	trachea	CNhs11092	Tracheal Epithelial Cells, donor1
UBERON:0001007	Digestive System	UBERON:0003126	trachea	CNhs11993	Tracheal Epithelial Cells, donor2
UBERON:0001007	Digestive System	UBERON:0003126	trachea	CNhs12051	Tracheal Epithelial Cells, donor3
UBERON:0001007	Digestive System	UBERON:0003126	trachea	CNhs11329	Smooth Muscle Cells - Tracheal, donor1
UBERON:0001007	Digestive System	UBERON:0003126	trachea	CNhs12567	Smooth Muscle Cells - Tracheal, donor2
UBERON:0001007	Digestive System	UBERON:0003126	trachea	CNhs12894	Smooth Muscle Cells - Tracheal, donor3
UBERON:0001007	Digestive System	UBERON:0003293	gland of oral region	CNhs11677	salivary gland, adult, pool1
UBERON:0001007	Digestive System	UBERON:0003293	gland of oral region	CNhs12849	parotid gland, adult
UBERON:0001007	Digestive System	UBERON:0003293	gland of oral region	CNhs12852	submaxillary gland, adult
UBERON:0001007	Digestive System	UBERON:0003293	gland of oral region	CNhs12810	salivary acinar cells, donor1
UBERON:0001007	Digestive System	UBERON:0003293	gland of oral region	CNhs12811	salivary acinar cells, donor2
UBERON:0001007	Digestive System	UBERON:0003293	gland of oral region	CNhs12812	salivary acinar cells, donor3
UBERON:0001007	Digestive System	UBERON:0003294	gland of foregut	CNhs11677	salivary gland, adult, pool1
UBERON:0001007	Digestive System	UBERON:0003294	gland of foregut	CNhs12849	parotid gland, adult
UBERON:0001007	Digestive System	UBERON:0003294	gland of foregut	CNhs12852	submaxillary gland, adult
UBERON:0001007	Digestive System	UBERON:0003294	gland of foregut	CNhs12810	salivary acinar cells, donor1
UBERON:0001007	Digestive System	UBERON:0003294	gland of foregut	CNhs12811	salivary acinar cells, donor2
UBERON:0001007	Digestive System	UBERON:0003294	gland of foregut	CNhs12812	salivary acinar cells, donor3
UBERON:0001007	Digestive System	UBERON:0003408	gland of gut	CNhs11677	salivary gland, adult, pool1
UBERON:0001007	Digestive System	UBERON:0003408	gland of gut	CNhs12849	parotid gland, adult
UBERON:0001007	Digestive System	UBERON:0003408	gland of gut	CNhs12852	submaxillary gland, adult
UBERON:0001007	Digestive System	UBERON:0003408	gland of gut	CNhs12810	salivary acinar cells, donor1
UBERON:0001007	Digestive System	UBERON:0003408	gland of gut	CNhs12811	salivary acinar cells, donor2
UBERON:0001007	Digestive System	UBERON:0003408	gland of gut	CNhs12812	salivary acinar cells, donor3
UBERON:0001007	Digestive System	UBERON:0003672	dentition	CNhs12493	Fibroblast - Periodontal Ligament, donor4 (PL29)
UBERON:0001007	Digestive System	UBERON:0003672	dentition	CNhs10867	Fibroblast - Periodontal Ligament, donor1
UBERON:0001007	Digestive System	UBERON:0003672	dentition	CNhs11953	Fibroblast - Periodontal Ligament, donor5 (PL30)
UBERON:0001007	Digestive System	UBERON:0003672	dentition	CNhs11962	Fibroblast - Periodontal Ligament, donor2
UBERON:0001007	Digestive System	UBERON:0003672	dentition	CNhs11996	Fibroblast - Periodontal Ligament, donor6 (PLH3)
UBERON:0001007	Digestive System	UBERON:0003672	dentition	CNhs11907	Fibroblast - Periodontal Ligament, donor3

UBERON:0001007	Digestive System	UBERON:0003729	mouth mucosa	CNhs11061	Gingival epithelial cells, donor1 (GEA11)
UBERON:0001007	Digestive System	UBERON:0003729	mouth mucosa	CNhs10848	Fibroblast - Gingival, donor4 (GFH2)
UBERON:0001007	Digestive System	UBERON:0003729	mouth mucosa	CNhs10866	Fibroblast - Gingival, donor1
UBERON:0001007	Digestive System	UBERON:0003729	mouth mucosa	CNhs10879	Keratinocyte - oral, donor1
UBERON:0001007	Digestive System	UBERON:0003729	mouth mucosa	CNhs11896	Gingival epithelial cells, donor2 (GEA14)
UBERON:0001007	Digestive System	UBERON:0003729	mouth mucosa	CNhs11952	Fibroblast - Gingival, donor5 (GFH3)
UBERON:0001007	Digestive System	UBERON:0003729	mouth mucosa	CNhs11961	Fibroblast - Gingival, donor2
UBERON:0001007	Digestive System	UBERON:0003729	mouth mucosa	CNhs11903	Gingival epithelial cells, donor3 (GEA15)
UBERON:0001007	Digestive System	UBERON:0003729	mouth mucosa	CNhs12006	Fibroblast - Gingival, donor3
UBERON:0001007	Digestive System	UBERON:0003729	mouth mucosa	CNhs14128	Fibroblast - Gingival, donor6 (aggressive periodontitis)
UBERON:0001007	Digestive System	UBERON:0003729	mouth mucosa	CNhs14129	Fibroblast - Gingival, donor6 (control)
UBERON:0001007	Digestive System	UBERON:0003729	mouth mucosa	CNhs14130	Fibroblast - Gingival, donor7 (aggressive periodontitis)
UBERON:0001007	Digestive System	UBERON:0003729	mouth mucosa	CNhs14131	Fibroblast - Gingival, donor7 (control)
UBERON:0001007	Digestive System	UBERON:0003729	mouth mucosa	CNhs14132	Fibroblast - Gingival, donor8 (chronic periodontitis)
UBERON:0001007	Digestive System	UBERON:0003729	mouth mucosa	CNhs14133	Fibroblast - Gingival, donor8 (control)
UBERON:0001007	Digestive System	UBERON:0003729	mouth mucosa	CNhs14134	Fibroblast - Gingival, donor9 (control)
UBERON:0001007	Digestive System	UBERON:0003729	mouth mucosa	CNhs14135	Fibroblast - Gingival, donor10 (periodontitis)
UBERON:0001007	Digestive System	UBERON:0003929	gut epithelium	CNhs11061	Gingival epithelial cells, donor1 (GEA11)
UBERON:0001007	Digestive System	UBERON:0003929	gut epithelium	CNhs10875	Intestinal epithelial cells (polarized), donor1
UBERON:0001007	Digestive System	UBERON:0003929	gut epithelium	CNhs11896	Gingival epithelial cells, donor2 (GEA14)
UBERON:0001007	Digestive System	UBERON:0003929	gut epithelium	CNhs11903	Gingival epithelial cells, donor3 (GEA15)
UBERON:0001007	Digestive System	UBERON:0004647	liver lobule	CNhs12075	Hepatic Sinusoidal Endothelial Cells, donor1
UBERON:0001007	Digestive System	UBERON:0004647	liver lobule	CNhs12092	Hepatic Sinusoidal Endothelial Cells, donor2
UBERON:0001007	Digestive System	UBERON:0004647	liver lobule	CNhs12625	Hepatic Sinusoidal Endothelial Cells, donor3
UBERON:0001007	Digestive System	UBERON:0004786	gastrointestinal system mucosa	CNhs10875	Intestinal epithelial cells (polarized), donor1
UBERON:0001007	Digestive System	UBERON:0004808	gastrointestinal system epithelium	CNhs10875	Intestinal epithelial cells (polarized), donor1
UBERON:0001007	Digestive System	UBERON:0004821	pulmonary alveolus epithelium	CNhs11325	Alveolar Epithelial Cells, donor1
UBERON:0001007	Digestive System	UBERON:0004821	pulmonary alveolus epithelium	CNhs12119	Alveolar Epithelial Cells, donor3
UBERON:0001007	Digestive System	UBERON:0004894	alveolar wall	CNhs11325	Alveolar Epithelial Cells, donor1
UBERON:0001007	Digestive System	UBERON:0004894	alveolar wall	CNhs12119	Alveolar Epithelial Cells, donor3
UBERON:0001007	Digestive System	UBERON:0004907	lower digestive tract	CNhs11777	rectum, fetal, donor1
UBERON:0001007	Digestive System	UBERON:0004907	lower digestive tract	CNhs12842	appendix, adult
UBERON:0001007	Digestive System	UBERON:0004921	subdivision of digestive tract	CNhs10619	colon, adult, pool1
UBERON:0001007	Digestive System	UBERON:0004921	subdivision of digestive tract	CNhs10620	esophagus, adult, pool1
UBERON:0001007	Digestive System	UBERON:0004921	subdivision of digestive tract	CNhs10630	small intestine, adult, pool1
UBERON:0001007	Digestive System	UBERON:0004921	subdivision of digestive tract	CNhs10654	tonsil, adult, pool1
UBERON:0001007	Digestive System	UBERON:0004921	subdivision of digestive tract	CNhs11771	stomach, fetal, donor1
UBERON:0001007	Digestive System	UBERON:0004921	subdivision of digestive tract	Cnhs11772	temporal lobe, fetal, donor1
UBERON:0001007	Digestive System	UBERON:0004921	subdivision of digestive tract	CNhs11773	small intestine, fetal, donor1
UBERON:0001007	Digestive System	UBERON:0004921	subdivision of digestive tract	CNhs11777	rectum, fetal, donor1
UBERON:0001007	Digestive System	UBERON:0004921	subdivision of digestive tract	CNhs11780	colon, fetal, donor1
UBERON:0001007	Digestive System	UBERON:0004921	subdivision of digestive tract	CNhs11794	colon, adult, donor1
UBERON:0001007	Digestive System	UBERON:0004921	subdivision of digestive tract	CNhs11677	salivary gland, adult, pool1
UBERON:0001007	Digestive System	UBERON:0004921	subdivision of digestive tract	CNhs12842	appendix, adult
UBERON:0001007	Digestive System	UBERON:0004921	subdivision of digestive tract	CNhs12849	parotid gland, adult
UBERON:0001007	Digestive System	UBERON:0004921	subdivision of digestive tract	CNhs12852	submaxillary gland, adult
UBERON:0001007	Digestive System	UBERON:0004921	subdivision of digestive tract	CNhs10868	Smooth Muscle Cells - Colonic, donor1
UBERON:0001007	Digestive System	UBERON:0004921	subdivision of digestive tract	CNhs10875	Intestinal epithelial cells (polarized), donor1
UBERON:0001007	Digestive System	UBERON:0004921	subdivision of digestive tract	CNhs11963	Smooth Muscle Cells - Colonic, donor2
UBERON:0001007	Digestive System	UBERON:0004921	subdivision of digestive tract	CNhs12007	Smooth Muscle Cells - Colonic, donor3
UBERON:0001007	Digestive System	UBERON:0004921	subdivision of digestive tract	CNhs11323	Esophageal Epithelial Cells, donor1
UBERON:0001007	Digestive System	UBERON:0004921	subdivision of digestive tract	CNhs11324	Smooth Muscle Cells - Esophageal, donor1
UBERON:0001007	Digestive System	UBERON:0004921	subdivision of digestive tract	CNhs12595	Smooth Muscle Cells - Intestinal, donor1
UBERON:0001007	Digestive System	UBERON:0004921	subdivision of digestive tract	CNhs12083	Esophageal Epithelial Cells, donor2
UBERON:0001007	Digestive System	UBERON:0004921	subdivision of digestive tract	CNhs12727	Smooth Muscle Cells - Esophageal, donor2
UBERON:0001007	Digestive System	UBERON:0004921	subdivision of digestive tract	CNhs12622	Esophageal Epithelial Cells, donor3
UBERON:0001007	Digestive System	UBERON:0004921	subdivision of digestive tract	CNhs12810	salivary acinar cells, donor1
UBERON:0001007	Digestive System	UBERON:0004921	subdivision of digestive tract	CNhs12811	salivary acinar cells, donor2
UBERON:0001007	Digestive System	UBERON:0004921	subdivision of digestive tract	CNhs12812	salivary acinar cells, donor3
UBERON:0001007	Digestive System	UBERON:0005409	gastrointestinal system	CNhs10619	colon, adult, pool1
UBERON:0001007	Digestive System	UBERON:0005409	gastrointestinal system	CNhs10630	small intestine, adult, pool1
UBERON:0001007	Digestive System	UBERON:0005409	gastrointestinal system	CNhs11771	stomach, fetal, donor1
UBERON:0001007	Digestive System	UBERON:0005409	gastrointestinal system	Cnhs11772	temporal lobe, fetal, donor1
UBERON:0001007	Digestive System	UBERON:0005409	gastrointestinal system	CNhs11773	small intestine, fetal, donor1
UBERON:0001007	Digestive System	UBERON:0005409	gastrointestinal system	CNhs11777	rectum, fetal, donor1
UBERON:0001007	Digestive System	UBERON:0005409	gastrointestinal system	CNhs11780	colon, fetal, donor1
UBERON:0001007	Digestive System	UBERON:0005409	gastrointestinal system	CNhs11794	colon, adult, donor1
UBERON:0001007	Digestive System	UBERON:0005409	gastrointestinal system	CNhs12842	appendix, adult
UBERON:0001007	Digestive System	UBERON:0005409	gastrointestinal system	CNhs10868	Smooth Muscle Cells - Colonic, donor1
UBERON:0001007	Digestive System	UBERON:0005409	gastrointestinal system	CNhs10875	Intestinal epithelial cells (polarized), donor1
UBERON:0001007	Digestive System	UBERON:0005409	gastrointestinal system	CNhs11963	Smooth Muscle Cells - Colonic, donor2
UBERON:0001007	Digestive System	UBERON:0005409	gastrointestinal system	CNhs12007	Smooth Muscle Cells - Colonic, donor3
UBERON:0001007	Digestive System	UBERON:0005409	gastrointestinal system	CNhs12595	Smooth Muscle Cells - Intestinal, donor1
UBERON:0001007	Digestive System	UBERON:0006518	right lung lobe	CNhs11786	lung, right lower lobe, adult, donor1
UBERON:0001007	Digestive System	UBERON:0006524	alveolar system	CNhs11325	Alveolar Epithelial Cells, donor1
UBERON:0001007	Digestive System	UBERON:0006524	alveolar system	CNhs12119	Alveolar Epithelial Cells, donor3
UBERON:0001007	Digestive System	UBERON:0006562	pharynx	CNhs10654	tonsil, adult, pool1
UBERON:0001007	Digestive System	UBERON:0006925	digestive gland	CNhs10624	liver, adult, pool1
UBERON:0001007	Digestive System	UBERON:0006925	digestive gland	CNhs11798	liver, fetal, pool1
UBERON:0001007	Digestive System	UBERON:0006925	digestive gland	CNhs10845	Mesenchymal stem cells - hepatic, donor0
UBERON:0001007	Digestive System	UBERON:0006925	digestive gland	CNhs12075	Hepatic Sinusoidal Endothelial Cells, donor1
UBERON:0001007	Digestive System	UBERON:0006925	digestive gland	CNhs12340	Hepatocyte, donor1
UBERON:0001007	Digestive System	UBERON:0006925	digestive gland	CNhs11335	Hepatic Stellate Cells (lipocyte), donor1
UBERON:0001007	Digestive System	UBERON:0006925	digestive gland	CNhs11346	Mesenchymal Stem Cells - hepatic, donor1
UBERON:0001007	Digestive System	UBERON:0006925	digestive gland	CNhs12092	Hepatic Sinusoidal Endothelial Cells, donor2
UBERON:0001007	Digestive System	UBERON:0006925	digestive gland	CNhs12349	Hepatocyte, donor2
UBERON:0001007	Digestive System	UBERON:0006925	digestive gland	CNhs12093	Hepatic Stellate Cells (lipocyte), donor2
UBERON:0001007	Digestive System	UBERON:0006925	digestive gland	CNhs12730	Mesenchymal Stem Cells - hepatic, donor2
UBERON:0001007	Digestive System	UBERON:0006925	digestive gland	CNhs12625	Hepatic Sinusoidal Endothelial Cells, donor3
UBERON:0001007	Digestive System	UBERON:0006925	digestive gland	CNhs12626	Hepatocyte, donor3
UBERON:0001007	Digestive System	UBERON:0006925	digestive gland	CNhs12627	Hepatic Stellate Cells (lipocyte), donor3
UBERON:0001007	Digestive System	UBERON:0008874	pulmonary acinus	CNhs11325	Alveolar Epithelial Cells, donor1
UBERON:0001007	Digestive System	UBERON:0008874	pulmonary acinus	CNhs12119	Alveolar Epithelial Cells, donor3
UBERON:0001007	Digestive System	UBERON:0008949	lower lobe of lung	CNhs11786	lung, right lower lobe, adult, donor1
UBERON:0001007	Digestive System	UBERON:0009471	dorsum of tongue	CNhs13460	tongue epidermis (fungiform papillae), donor1
UBERON:0001007	Digestive System	UBERON:0009854	digestive tract diverticulum	CNhs12842	appendix, adult
UBERON:0001007	Digestive System	UBERON:0010039	food storage organ	CNhs11771	stomach, fetal, donor1
UBERON:0001007	Digestive System	UBERON:0010047	oral gland	CNhs11677	salivary gland, adult, pool1

UBERON:0001007	Digestive System	UBERON:0010047	oral gland	CNhs12849	parotid gland, adult
UBERON:0001007	Digestive System	UBERON:0010047	oral gland	CNhs12852	submaxillary gland, adult
UBERON:0001007	Digestive System	UBERON:0010047	oral gland	CNhs12810	salivary acinar cells, donor1
UBERON:0001007	Digestive System	UBERON:0010047	oral gland	CNhs12811	salivary acinar cells, donor2
UBERON:0001007	Digestive System	UBERON:0010047	oral gland	CNhs12812	salivary acinar cells, donor3
UBERON:0001007	Digestive System	UBERON:0010368	pulmonary lobule	CNhs11325	Alveolar Epithelial Cells, donor1
UBERON:0001007	Digestive System	UBERON:0010368	pulmonary lobule	CNhs12119	Alveolar Epithelial Cells, donor3
UBERON:0001009	Circulatory System	UBERON:0000178	blood	CNhs11761	blood, adult, pool1
UBERON:0001009	Circulatory System	UBERON:0000178	blood	CNhs10860	Peripheral Blood Mononuclear Cells, donor1
UBERON:0001009	Circulatory System	UBERON:0000178	blood	CNhs11958	Peripheral Blood Mononuclear Cells, donor2
UBERON:0001009	Circulatory System	UBERON:0000178	blood	CNhs12002	Peripheral Blood Mononuclear Cells, donor3
UBERON:0001009	Circulatory System	UBERON:0000178	blood	CNhs11075	Whole blood (ribopure), donor090325, donation1
UBERON:0001009	Circulatory System	UBERON:0000178	blood	CNhs11076	Whole blood (ribopure), donor090325, donation2
UBERON:0001009	Circulatory System	UBERON:0000178	blood	CNhs11675	Whole blood (ribopure), donor090309, donation1
UBERON:0001009	Circulatory System	UBERON:0000178	blood	CNhs11671	Whole blood (ribopure), donor090309, donation2
UBERON:0001009	Circulatory System	UBERON:0000178	blood	CNhs11948	Whole blood (ribopure), donor090309, donation3
UBERON:0001009	Circulatory System	UBERON:0000178	blood	CNhs11672	Whole blood (ribopure), donor090612, donation1
UBERON:0001009	Circulatory System	UBERON:0000178	blood	CNhs11673	Whole blood (ribopure), donor090612, donation2
UBERON:0001009	Circulatory System	UBERON:0000178	blood	CNhs11949	Whole blood (ribopure), donor090612, donation3
UBERON:0001009	Circulatory System	UBERON:0000415	artery wall	CNhs10874	Fibroblast - Aortic Adventitial, donor1
UBERON:0001009	Circulatory System	UBERON:0000415	artery wall	CNhs11968	Fibroblast - Aortic Adventitial, donor2
UBERON:0001009	Circulatory System	UBERON:0000415	artery wall	CNhs12011	Fibroblast - Aortic Adventitial, donor3
UBERON:0001009	Circulatory System	UBERON:0000946	cardial valve	CNhs12855	heart - mitral valve, adult
UBERON:0001009	Circulatory System	UBERON:0000946	cardial valve	CNhs12856	heart - pulmonic valve, adult
UBERON:0001009	Circulatory System	UBERON:0000946	cardial valve	CNhs12857	heart - tricuspid valve, adult
UBERON:0001009	Circulatory System	UBERON:0000947	aorta	CNhs11760	aorta, adult, pool1
UBERON:0001009	Circulatory System	UBERON:0000947	aorta	CNhs10837	Endothelial Cells - Aortic, donor0
UBERON:0001009	Circulatory System	UBERON:0000947	aorta	CNhs10838	Smooth Muscle Cells - Aortic, donor0
UBERON:0001009	Circulatory System	UBERON:0000947	aorta	CNhs10874	Fibroblast - Aortic Adventitial, donor1
UBERON:0001009	Circulatory System	UBERON:0000947	aorta	CNhs12495	Endothelial Cells - Aortic, donor1
UBERON:0001009	Circulatory System	UBERON:0000947	aorta	CNhs11926	Endothelial Cells - Thoracic, donor1
UBERON:0001009	Circulatory System	UBERON:0000947	aorta	CNhs11085	Smooth Muscle Cells - Aortic, donor1
UBERON:0001009	Circulatory System	UBERON:0000947	aorta	CNhs11968	Fibroblast - Aortic Adventitial, donor2
UBERON:0001009	Circulatory System	UBERON:0000947	aorta	CNhs11375	Endothelial Cells - Aortic, donor2
UBERON:0001009	Circulatory System	UBERON:0000947	aorta	CNhs11978	Endothelial Cells - Thoracic, donor2
UBERON:0001009	Circulatory System	UBERON:0000947	aorta	CNhs11305	Smooth Muscle Cells - Aortic, donor2
UBERON:0001009	Circulatory System	UBERON:0000947	aorta	CNhs12011	Fibroblast - Aortic Adventitial, donor3
UBERON:0001009	Circulatory System	UBERON:0000947	aorta	CNhs12022	Endothelial Cells - Aortic, donor3
UBERON:0001009	Circulatory System	UBERON:0000947	aorta	CNhs11309	Smooth Muscle Cells - Aortic, donor3
UBERON:0001009	Circulatory System	UBERON:0000948	heart	CNhs10653	heart, fetal, pool1
UBERON:0001009	Circulatory System	UBERON:0000948	heart	CNhs11789	left ventricle, adult, donor1
UBERON:0001009	Circulatory System	UBERON:0000948	heart	CNhs11790	left atrium, adult, donor1
UBERON:0001009	Circulatory System	UBERON:0000948	heart	CNhs12855	heart - mitral valve, adult
UBERON:0001009	Circulatory System	UBERON:0000948	heart	CNhs12856	heart - pulmonic valve, adult
UBERON:0001009	Circulatory System	UBERON:0000948	heart	CNhs12857	heart - tricuspid valve, adult
UBERON:0001009	Circulatory System	UBERON:0000948	heart	CNhs12498	Fibroblast - Cardiac, donor1
UBERON:0001009	Circulatory System	UBERON:0000948	heart	CNhs11088	Smooth Muscle Cells - Coronary Artery, donor1
UBERON:0001009	Circulatory System	UBERON:0000948	heart	CNhs11378	Fibroblast - Cardiac, donor2
UBERON:0001009	Circulatory System	UBERON:0000948	heart	CNhs11987	Smooth Muscle Cells - Coronary Artery, donor2
UBERON:0001009	Circulatory System	UBERON:0000948	heart	CNhs12027	Fibroblast - Cardiac, donor3
UBERON:0001009	Circulatory System	UBERON:0000948	heart	CNhs12045	Smooth Muscle Cells - Coronary Artery, donor3
UBERON:0001009	Circulatory System	UBERON:0000948	heart	CNhs11909	Fibroblast - Cardiac, donor4
UBERON:0001009	Circulatory System	UBERON:0000948	heart	CNhs12057	Fibroblast - Cardiac, donor5
UBERON:0001009	Circulatory System	UBERON:0000948	heart	CNhs12061	Fibroblast - Cardiac, donor6
UBERON:0001009	Circulatory System	UBERON:0000948	heart	CNhs12341	Cardiac Myocyte, donor1
UBERON:0001009	Circulatory System	UBERON:0000948	heart	CNhs12350	Cardiac Myocyte, donor2
UBERON:0001009	Circulatory System	UBERON:0000948	heart	CNhs12571	Cardiac Myocyte, donor3
UBERON:0001009	Circulatory System	UBERON:0000948	heart	CNhs12368	mesenchymal precursor cell - cardiac, donor1
UBERON:0001009	Circulatory System	UBERON:0000948	heart	CNhs12369	mesenchymal precursor cell - cardiac, donor2
UBERON:0001009	Circulatory System	UBERON:0000948	heart	CNhs12370	mesenchymal precursor cell - cardiac, donor3
UBERON:0001009	Circulatory System	UBERON:0000948	heart	CNhs12371	mesenchymal precursor cell - cardiac, donor4
UBERON:0001009	Circulatory System	UBERON:0001009	circulatory system	CNhs10621	heart, adult, pool1
UBERON:0001009	Circulatory System	UBERON:0001009	circulatory system	CNhs10653	heart, fetal, pool1
UBERON:0001009	Circulatory System	UBERON:0001009	circulatory system	CNhs11757	heart, adult, diseased post-infarction, donor1
UBERON:0001009	Circulatory System	UBERON:0001009	circulatory system	CNhs11758	heart, adult, diseased, donor1
UBERON:0001009	Circulatory System	UBERON:0001009	circulatory system	CNhs11760	aorta, adult, pool1
UBERON:0001009	Circulatory System	UBERON:0001009	circulatory system	CNhs11761	blood, adult, pool1
UBERON:0001009	Circulatory System	UBERON:0001009	circulatory system	CNhs11789	left ventricle, adult, donor1
UBERON:0001009	Circulatory System	UBERON:0001009	circulatory system	CNhs11790	left atrium, adult, donor1
UBERON:0001009	Circulatory System	UBERON:0001009	circulatory system	CNhs12843	artery, adult
UBERON:0001009	Circulatory System	UBERON:0001009	circulatory system	CNhs12855	heart - mitral valve, adult
UBERON:0001009	Circulatory System	UBERON:0001009	circulatory system	CNhs12856	heart - pulmonic valve, adult
UBERON:0001009	Circulatory System	UBERON:0001009	circulatory system	CNhs12857	heart - tricuspid valve, adult
UBERON:0001009	Circulatory System	UBERON:0001009	circulatory system	CNhs13440	eye - vitreous humor, donor1
UBERON:0001009	Circulatory System	UBERON:0001009	circulatory system	CNhs10837	Endothelial Cells - Aortic, donor0
UBERON:0001009	Circulatory System	UBERON:0001009	circulatory system	CNhs10838	Smooth Muscle Cells - Aortic, donor0
UBERON:0001009	Circulatory System	UBERON:0001009	circulatory system	CNhs10839	Smooth Muscle Cells - Umbilical artery, donor0
UBERON:0001009	Circulatory System	UBERON:0001009	circulatory system	CNhs10860	Peripheral Blood Mononuclear Cells, donor1
UBERON:0001009	Circulatory System	UBERON:0001009	circulatory system	CNhs10863	Smooth Muscle Cells - Brain Vascular, donor1
UBERON:0001009	Circulatory System	UBERON:0001009	circulatory system	CNhs10865	Endothelial Cells - Lymphatic, donor1
UBERON:0001009	Circulatory System	UBERON:0001009	circulatory system	CNhs10871	Ciliary Epithelial Cells, donor1
UBERON:0001009	Circulatory System	UBERON:0001009	circulatory system	CNhs10872	Endothelial Cells - Umbilical vein, donor1
UBERON:0001009	Circulatory System	UBERON:0001009	circulatory system	CNhs10874	Fibroblast - Aortic Adventitial, donor1
UBERON:0001009	Circulatory System	UBERON:0001009	circulatory system	CNhs10878	Fibroblast - Pulmonary Artery, donor1
UBERON:0001009	Circulatory System	UBERON:0001009	circulatory system	CNhs12495	Endothelial Cells - Aortic, donor1
UBERON:0001009	Circulatory System	UBERON:0001009	circulatory system	CNhs12496	Endothelial Cells - Artery, donor1
UBERON:0001009	Circulatory System	UBERON:0001009	circulatory system	CNhs11925	Endothelial Cells - Microvascular, donor1
UBERON:0001009	Circulatory System	UBERON:0001009	circulatory system	CNhs11926	Endothelial Cells - Thoracic, donor1
UBERON:0001009	Circulatory System	UBERON:0001009	circulatory system	CNhs12497	Endothelial Cells - Vein, donor1
UBERON:0001009	Circulatory System	UBERON:0001009	circulatory system	CNhs12498	Fibroblast - Cardiac, donor1
UBERON:0001009	Circulatory System	UBERON:0001009	circulatory system	CNhs11085	Smooth Muscle Cells - Aortic, donor1
UBERON:0001009	Circulatory System	UBERON:0001009	circulatory system	CNhs11086	Smooth Muscle Cells - Brachiocephalic, donor1
UBERON:0001009	Circulatory System	UBERON:0001009	circulatory system	CNhs11087	Smooth Muscle Cells - Carotid, donor1
UBERON:0001009	Circulatory System	UBERON:0001009	circulatory system	CNhs11088	Smooth Muscle Cells - Coronary Artery, donor1
UBERON:0001009	Circulatory System	UBERON:0001009	circulatory system	CNhs11067	Smooth Muscle Cells - Internal Thoracic Artery, donor1
UBERON:0001009	Circulatory System	UBERON:0001009	circulatory system	CNhs11089	Smooth Muscle Cells - Pulmonary Artery, donor1
UBERON:0001009	Circulatory System	UBERON:0001009	circulatory system	CNhs11090	Smooth Muscle Cells - Subclavian Artery, donor1
UBERON:0001009	Circulatory System	UBERON:0001009	circulatory system	CNhs11091	Smooth Muscle Cells - Umbilical Artery, donor1

UBERON:0001009	Circulatory System	UBERON:0001009	circulatory system	CNhs11958	Peripheral Blood Mononuclear Cells, donor2
UBERON:0001009	Circulatory System	UBERON:0001009	circulatory system	CNhs11900	Smooth Muscle Cells - Brain Vascular, donor2
UBERON:0001009	Circulatory System	UBERON:0001009	circulatory system	CNhs11901	Endothelial Cells - Lymphatic, donor2
UBERON:0001009	Circulatory System	UBERON:0001009	circulatory system	CNhs11966	Ciliary Epithelial Cells, donor2
UBERON:0001009	Circulatory System	UBERON:0001009	circulatory system	CNhs11967	Endothelial Cells - Umbilical vein, donor2
UBERON:0001009	Circulatory System	UBERON:0001009	circulatory system	CNhs11968	Fibroblast - Aortic Adventitial, donor2
UBERON:0001009	Circulatory System	UBERON:0001009	circulatory system	CNhs11375	Endothelial Cells - Aortic, donor2
UBERON:0001009	Circulatory System	UBERON:0001009	circulatory system	CNhs11977	Endothelial Cells - Artery, donor2
UBERON:0001009	Circulatory System	UBERON:0001009	circulatory system	CNhs11376	Endothelial Cells - Microvascular, donor2
UBERON:0001009	Circulatory System	UBERON:0001009	circulatory system	CNhs11978	Endothelial Cells - Thoracic, donor2
UBERON:0001009	Circulatory System	UBERON:0001009	circulatory system	CNhs11377	Endothelial Cells - Vein, donor2
UBERON:0001009	Circulatory System	UBERON:0001009	circulatory system	CNhs11378	Fibroblast - Cardiac, donor2
UBERON:0001009	Circulatory System	UBERON:0001009	circulatory system	CNhs11305	Smooth Muscle Cells - Aortic, donor2
UBERON:0001009	Circulatory System	UBERON:0001009	circulatory system	CNhs11985	Smooth Muscle Cells - Brachiocephalic, donor2
UBERON:0001009	Circulatory System	UBERON:0001009	circulatory system	CNhs11986	Smooth Muscle Cells - Carotid, donor2
UBERON:0001009	Circulatory System	UBERON:0001009	circulatory system	CNhs11987	Smooth Muscle Cells - Coronary Artery, donor2
UBERON:0001009	Circulatory System	UBERON:0001009	circulatory system	CNhs11988	Smooth Muscle Cells - Internal Thoracic Artery, donor2
UBERON:0001009	Circulatory System	UBERON:0001009	circulatory system	CNhs11989	Smooth Muscle Cells - Pulmonary Artery, donor2
UBERON:0001009	Circulatory System	UBERON:0001009	circulatory system	CNhs11990	Smooth Muscle Cells - Subclavian Artery, donor2
UBERON:0001009	Circulatory System	UBERON:0001009	circulatory system	CNhs11991	Smooth Muscle Cells - Umbilical Artery, donor2
UBERON:0001009	Circulatory System	UBERON:0001009	circulatory system	CNhs12002	Peripheral Blood Mononuclear Cells, donor3
UBERON:0001009	Circulatory System	UBERON:0001009	circulatory system	CNhs12004	Smooth Muscle Cells - Brain Vascular, donor3
UBERON:0001009	Circulatory System	UBERON:0001009	circulatory system	CNhs11906	Endothelial Cells - Lymphatic, donor3
UBERON:0001009	Circulatory System	UBERON:0001009	circulatory system	CNhs12009	Ciliary Epithelial Cells, donor3
UBERON:0001009	Circulatory System	UBERON:0001009	circulatory system	CNhs12010	Endothelial Cells - Umbilical vein, donor3
UBERON:0001009	Circulatory System	UBERON:0001009	circulatory system	CNhs12011	Fibroblast - Aortic Adventitial, donor3
UBERON:0001009	Circulatory System	UBERON:0001009	circulatory system	CNhs12022	Endothelial Cells - Aortic, donor3
UBERON:0001009	Circulatory System	UBERON:0001009	circulatory system	CNhs12023	Endothelial Cells - Artery, donor3
UBERON:0001009	Circulatory System	UBERON:0001009	circulatory system	CNhs12024	Endothelial Cells - Microvascular, donor3
UBERON:0001009	Circulatory System	UBERON:0001009	circulatory system	CNhs12026	Endothelial Cells - Vein, donor3
UBERON:0001009	Circulatory System	UBERON:0001009	circulatory system	CNhs12027	Fibroblast - Cardiac, donor3
UBERON:0001009	Circulatory System	UBERON:0001009	circulatory system	CNhs11309	Smooth Muscle Cells - Aortic, donor3
UBERON:0001009	Circulatory System	UBERON:0001009	circulatory system	CNhs12043	Smooth Muscle Cells - Brachiocephalic, donor3
UBERON:0001009	Circulatory System	UBERON:0001009	circulatory system	CNhs12044	Smooth Muscle Cells - Carotid, donor3
UBERON:0001009	Circulatory System	UBERON:0001009	circulatory system	CNhs12045	Smooth Muscle Cells - Coronary Artery, donor3
UBERON:0001009	Circulatory System	UBERON:0001009	circulatory system	CNhs12046	Smooth Muscle Cells - Internal Thoracic Artery, donor3
UBERON:0001009	Circulatory System	UBERON:0001009	circulatory system	CNhs12047	Smooth Muscle Cells - Pulmonary Artery, donor3
UBERON:0001009	Circulatory System	UBERON:0001009	circulatory system	CNhs12048	Smooth Muscle Cells - Subclavian Artery, donor3
UBERON:0001009	Circulatory System	UBERON:0001009	circulatory system	CNhs12049	Smooth Muscle Cells - Umbilical Artery, donor3
UBERON:0001009	Circulatory System	UBERON:0001009	circulatory system	CNhs11909	Fibroblast - Cardiac, donor4
UBERON:0001009	Circulatory System	UBERON:0001009	circulatory system	CNhs12057	Fibroblast - Cardiac, donor5
UBERON:0001009	Circulatory System	UBERON:0001009	circulatory system	CNhs12061	Fibroblast - Cardiac, donor6
UBERON:0001009	Circulatory System	UBERON:0001009	circulatory system	CNhs11319	Fibroblast - Choroid Plexus, donor1
UBERON:0001009	Circulatory System	UBERON:0001009	circulatory system	CNhs11322	Fibroblast - Lymphatic, donor1
UBERON:0001009	Circulatory System	UBERON:0001009	circulatory system	CNhs12075	Hepatic Sinusoidal Endothelial Cells, donor1
UBERON:0001009	Circulatory System	UBERON:0001009	circulatory system	CNhs12341	Cardiac Myocyte, donor1
UBERON:0001009	Circulatory System	UBERON:0001009	circulatory system	CNhs12596	Iris Pigment Epithelial Cells, donor1
UBERON:0001009	Circulatory System	UBERON:0001009	circulatory system	CNhs11340	Trabecular Meshwork Cells, donor1
UBERON:0001009	Circulatory System	UBERON:0001009	circulatory system	CNhs12597	Smooth Muscle Cells - Umbilical Vein, donor1
UBERON:0001009	Circulatory System	UBERON:0001009	circulatory system	CNhs12344	Fibroblast - Choroid Plexus, donor2
UBERON:0001009	Circulatory System	UBERON:0001009	circulatory system	CNhs12082	Fibroblast - Lymphatic, donor2
UBERON:0001009	Circulatory System	UBERON:0001009	circulatory system	CNhs12092	Hepatic Sinusoidal Endothelial Cells, donor2
UBERON:0001009	Circulatory System	UBERON:0001009	circulatory system	CNhs12350	Cardiac Myocyte, donor2
UBERON:0001009	Circulatory System	UBERON:0001009	circulatory system	CNhs12097	Trabecular Meshwork Cells, donor2
UBERON:0001009	Circulatory System	UBERON:0001009	circulatory system	CNhs12569	Smooth Muscle Cells - Umbilical Vein, donor2
UBERON:0001009	Circulatory System	UBERON:0001009	circulatory system	CNhs12620	Fibroblast - Choroid Plexus, donor3
UBERON:0001009	Circulatory System	UBERON:0001009	circulatory system	CNhs12118	Fibroblast - Lymphatic, donor3
UBERON:0001009	Circulatory System	UBERON:0001009	circulatory system	CNhs12625	Hepatic Sinusoidal Endothelial Cells, donor3
UBERON:0001009	Circulatory System	UBERON:0001009	circulatory system	CNhs12571	Cardiac Myocyte, donor3
UBERON:0001009	Circulatory System	UBERON:0001009	circulatory system	CNhs12124	Trabecular Meshwork Cells, donor3
UBERON:0001009	Circulatory System	UBERON:0001009	circulatory system	CNhs13076	Smooth Muscle Cells - Umbilical Vein, donor3
UBERON:0001009	Circulatory System	UBERON:0001009	circulatory system	CNhs12368	mesenchymal precursor cell - cardiac, donor1
UBERON:0001009	Circulatory System	UBERON:0001009	circulatory system	CNhs12369	mesenchymal precursor cell - cardiac, donor2
UBERON:0001009	Circulatory System	UBERON:0001009	circulatory system	CNhs12370	mesenchymal precursor cell - cardiac, donor3
UBERON:0001009	Circulatory System	UBERON:0001009	circulatory system	CNhs12371	mesenchymal precursor cell - cardiac, donor4
UBERON:0001009	Circulatory System	UBERON:0001009	circulatory system	CNhs11075	Whole blood (ribopure), donor090325, donation1
UBERON:0001009	Circulatory System	UBERON:0001009	circulatory system	CNhs11076	Whole blood (ribopure), donor090325, donation2
UBERON:0001009	Circulatory System	UBERON:0001009	circulatory system	CNhs11675	Whole blood (ribopure), donor090309, donation1
UBERON:0001009	Circulatory System	UBERON:0001009	circulatory system	CNhs11671	Whole blood (ribopure), donor090309, donation2
UBERON:0001009	Circulatory System	UBERON:0001009	circulatory system	CNhs11948	Whole blood (ribopure), donor090309, donation3
UBERON:0001009	Circulatory System	UBERON:0001009	circulatory system	CNhs11672	Whole blood (ribopure), donor090612, donation1
UBERON:0001009	Circulatory System	UBERON:0001009	circulatory system	CNhs11673	Whole blood (ribopure), donor090612, donation2
UBERON:0001009	Circulatory System	UBERON:0001009	circulatory system	CNhs11949	Whole blood (ribopure), donor090612, donation3
UBERON:0001009	Circulatory System	UBERON:0001281	hepatic sinusoid	CNhs12075	Hepatic Sinusoidal Endothelial Cells, donor1
UBERON:0001009	Circulatory System	UBERON:0001281	hepatic sinusoid	CNhs12092	Hepatic Sinusoidal Endothelial Cells, donor2
UBERON:0001009	Circulatory System	UBERON:0001281	hepatic sinusoid	CNhs12625	Hepatic Sinusoidal Endothelial Cells, donor3
UBERON:0001009	Circulatory System	UBERON:0001310	umbilical artery	CNhs10839	Smooth Muscle Cells - Umbilical artery, donor0
UBERON:0001009	Circulatory System	UBERON:0001310	umbilical artery	CNhs11091	Smooth Muscle Cells - Umbilical Artery, donor1
UBERON:0001009	Circulatory System	UBERON:0001310	umbilical artery	CNhs11991	Smooth Muscle Cells - Umbilical Artery, donor2
UBERON:0001009	Circulatory System	UBERON:0001310	umbilical artery	CNhs12049	Smooth Muscle Cells - Umbilical Artery, donor3
UBERON:0001009	Circulatory System	UBERON:0001473	lymphatic vessel	CNhs10865	Endothelial Cells - Lymphatic, donor1
UBERON:0001009	Circulatory System	UBERON:0001473	lymphatic vessel	CNhs11901	Endothelial Cells - Lymphatic, donor2
UBERON:0001009	Circulatory System	UBERON:0001473	lymphatic vessel	CNhs11906	Endothelial Cells - Lymphatic, donor3
UBERON:0001009	Circulatory System	UBERON:0001473	lymphatic vessel	CNhs11322	Fibroblast - Lymphatic, donor1
UBERON:0001009	Circulatory System	UBERON:0001473	lymphatic vessel	CNhs12082	Fibroblast - Lymphatic, donor2
UBERON:0001009	Circulatory System	UBERON:0001473	lymphatic vessel	CNhs12118	Fibroblast - Lymphatic, donor3
UBERON:0001009	Circulatory System	UBERON:0001515	thoracic aorta	CNhs11926	Endothelial Cells - Thoracic, donor1
UBERON:0001009	Circulatory System	UBERON:0001515	thoracic aorta	CNhs11978	Endothelial Cells - Thoracic, donor2
UBERON:0001009	Circulatory System	UBERON:0001529	brachiocephalic artery	CNhs11086	Smooth Muscle Cells - Brachiocephalic, donor1
UBERON:0001009	Circulatory System	UBERON:0001529	brachiocephalic artery	CNhs11985	Smooth Muscle Cells - Brachiocephalic, donor2
UBERON:0001009	Circulatory System	UBERON:0001529	brachiocephalic artery	CNhs12043	Smooth Muscle Cells - Brachiocephalic, donor3
UBERON:0001009	Circulatory System	UBERON:0001533	subclavian artery	CNhs11067	Smooth Muscle Cells - Internal Thoracic Artery, donor1
UBERON:0001009	Circulatory System	UBERON:0001533	subclavian artery	CNhs11090	Smooth Muscle Cells - Subclavian Artery, donor1
UBERON:0001009	Circulatory System	UBERON:0001533	subclavian artery	CNhs11988	Smooth Muscle Cells - Internal Thoracic Artery, donor2
UBERON:0001009	Circulatory System	UBERON:0001533	subclavian artery	CNhs11990	Smooth Muscle Cells - Subclavian Artery, donor2
UBERON:0001009	Circulatory System	UBERON:0001533	subclavian artery	CNhs12046	Smooth Muscle Cells - Internal Thoracic Artery, donor3
UBERON:0001009	Circulatory System	UBERON:0001533	subclavian artery	CNhs12048	Smooth Muscle Cells - Subclavian Artery, donor3
UBERON:0001009	Circulatory System	UBERON:0001621	coronary artery	CNhs11088	Smooth Muscle Cells - Coronary Artery, donor1

UBERON:0001009	Circulatory System	UBERON:0001621	coronary artery	CNhs11987	Smooth Muscle Cells - Coronary Artery, donor2
UBERON:0001009	Circulatory System	UBERON:0001621	coronary artery	CNhs12045	Smooth Muscle Cells - Coronary Artery, donor3
UBERON:0001009	Circulatory System	UBERON:0001637	artery	CNhs11760	aorta, adult, pool1
UBERON:0001009	Circulatory System	UBERON:0001637	artery	CNhs12843	artery, adult
UBERON:0001009	Circulatory System	UBERON:0001637	artery	CNhs10837	Endothelial Cells - Aortic, donor0
UBERON:0001009	Circulatory System	UBERON:0001637	artery	CNhs10838	Smooth Muscle Cells - Aortic, donor0
UBERON:0001009	Circulatory System	UBERON:0001637	artery	CNhs10839	Smooth Muscle Cells - Umbilical artery, donor0
UBERON:0001009	Circulatory System	UBERON:0001637	artery	CNhs10874	Fibroblast - Aortic Adventitial, donor1
UBERON:0001009	Circulatory System	UBERON:0001637	artery	CNhs10878	Fibroblast - Pulmonary Artery, donor1
UBERON:0001009	Circulatory System	UBERON:0001637	artery	CNhs12495	Endothelial Cells - Aortic, donor1
UBERON:0001009	Circulatory System	UBERON:0001637	artery	CNhs12496	Endothelial Cells - Artery, donor1
UBERON:0001009	Circulatory System	UBERON:0001637	artery	CNhs11926	Endothelial Cells - Thoracic, donor1
UBERON:0001009	Circulatory System	UBERON:0001637	artery	CNhs11085	Smooth Muscle Cells - Aortic, donor1
UBERON:0001009	Circulatory System	UBERON:0001637	artery	CNhs11086	Smooth Muscle Cells - Brachiocephalic, donor1
UBERON:0001009	Circulatory System	UBERON:0001637	artery	CNhs11087	Smooth Muscle Cells - Carotid, donor1
UBERON:0001009	Circulatory System	UBERON:0001637	artery	CNhs11088	Smooth Muscle Cells - Coronary Artery, donor1
UBERON:0001009	Circulatory System	UBERON:0001637	artery	CNhs11067	Smooth Muscle Cells - Internal Thoracic Artery, donor1
UBERON:0001009	Circulatory System	UBERON:0001637	artery	CNhs11089	Smooth Muscle Cells - Pulmonary Artery, donor1
UBERON:0001009	Circulatory System	UBERON:0001637	artery	CNhs11090	Smooth Muscle Cells - Subclavian Artery, donor1
UBERON:0001009	Circulatory System	UBERON:0001637	artery	CNhs11091	Smooth Muscle Cells - Umbilical Artery, donor1
UBERON:0001009	Circulatory System	UBERON:0001637	artery	CNhs11968	Fibroblast - Aortic Adventitial, donor2
UBERON:0001009	Circulatory System	UBERON:0001637	artery	CNhs11375	Endothelial Cells - Aortic, donor2
UBERON:0001009	Circulatory System	UBERON:0001637	artery	CNhs11977	Endothelial Cells - Artery, donor2
UBERON:0001009	Circulatory System	UBERON:0001637	artery	CNhs11978	Endothelial Cells - Thoracic, donor2
UBERON:0001009	Circulatory System	UBERON:0001637	artery	CNhs11305	Smooth Muscle Cells - Aortic, donor2
UBERON:0001009	Circulatory System	UBERON:0001637	artery	CNhs11985	Smooth Muscle Cells - Brachiocephalic, donor2
UBERON:0001009	Circulatory System	UBERON:0001637	artery	CNhs11986	Smooth Muscle Cells - Carotid, donor2
UBERON:0001009	Circulatory System	UBERON:0001637	artery	CNhs11987	Smooth Muscle Cells - Coronary Artery, donor2
UBERON:0001009	Circulatory System	UBERON:0001637	artery	CNhs11988	Smooth Muscle Cells - Internal Thoracic Artery, donor2
UBERON:0001009	Circulatory System	UBERON:0001637	artery	CNhs11989	Smooth Muscle Cells - Pulmonary Artery, donor2
UBERON:0001009	Circulatory System	UBERON:0001637	artery	CNhs11990	Smooth Muscle Cells - Subclavian Artery, donor2
UBERON:0001009	Circulatory System	UBERON:0001637	artery	CNhs11991	Smooth Muscle Cells - Umbilical Artery, donor2
UBERON:0001009	Circulatory System	UBERON:0001637	artery	CNhs12011	Fibroblast - Aortic Adventitial, donor3
UBERON:0001009	Circulatory System	UBERON:0001637	artery	CNhs12022	Endothelial Cells - Aortic, donor3
UBERON:0001009	Circulatory System	UBERON:0001637	artery	CNhs12023	Endothelial Cells - Artery, donor3
UBERON:0001009	Circulatory System	UBERON:0001637	artery	CNhs11309	Smooth Muscle Cells - Aortic, donor3
UBERON:0001009	Circulatory System	UBERON:0001637	artery	CNhs12043	Smooth Muscle Cells - Brachiocephalic, donor3
UBERON:0001009	Circulatory System	UBERON:0001637	artery	CNhs12044	Smooth Muscle Cells - Carotid, donor3
UBERON:0001009	Circulatory System	UBERON:0001637	artery	CNhs12045	Smooth Muscle Cells - Coronary Artery, donor3
UBERON:0001009	Circulatory System	UBERON:0001637	artery	CNhs12046	Smooth Muscle Cells - Internal Thoracic Artery, donor3
UBERON:0001009	Circulatory System	UBERON:0001637	artery	CNhs12047	Smooth Muscle Cells - Pulmonary Artery, donor3
UBERON:0001009	Circulatory System	UBERON:0001637	artery	CNhs12048	Smooth Muscle Cells - Subclavian Artery, donor3
UBERON:0001009	Circulatory System	UBERON:0001637	artery	CNhs12049	Smooth Muscle Cells - Umbilical Artery, donor3
UBERON:0001009	Circulatory System	UBERON:0001638	vein	CNhs10872	Endothelial Cells - Umbilical vein, donor1
UBERON:0001009	Circulatory System	UBERON:0001638	vein	CNhs12497	Endothelial Cells - Vein, donor1
UBERON:0001009	Circulatory System	UBERON:0001638	vein	CNhs11967	Endothelial Cells - Umbilical vein, donor2
UBERON:0001009	Circulatory System	UBERON:0001638	vein	CNhs11377	Endothelial Cells - Vein, donor2
UBERON:0001009	Circulatory System	UBERON:0001638	vein	CNhs12010	Endothelial Cells - Umbilical vein, donor3
UBERON:0001009	Circulatory System	UBERON:0001638	vein	CNhs12026	Endothelial Cells - Vein, donor3
UBERON:0001009	Circulatory System	UBERON:0001638	vein	CNhs12597	Smooth Muscle Cells - Umbilical Vein, donor1
UBERON:0001009	Circulatory System	UBERON:0001638	vein	CNhs12569	Smooth Muscle Cells - Umbilical Vein, donor2
UBERON:0001009	Circulatory System	UBERON:0001638	vein	CNhs13076	Smooth Muscle Cells - Umbilical Vein, donor3
UBERON:0001009	Circulatory System	UBERON:0001768	vascular layer of eyeball	CNhs10871	Ciliary Epithelial Cells, donor1
UBERON:0001009	Circulatory System	UBERON:0001768	vascular layer of eyeball	CNhs11966	Ciliary Epithelial Cells, donor2
UBERON:0001009	Circulatory System	UBERON:0001768	vascular layer of eyeball	CNhs12009	Ciliary Epithelial Cells, donor3
UBERON:0001009	Circulatory System	UBERON:0001768	vascular layer of eyeball	CNhs12596	Iris Pigment Epithelial Cells, donor1
UBERON:0001009	Circulatory System	UBERON:0001768	vascular layer of eyeball	CNhs11340	Trabecular Meshwork Cells, donor1
UBERON:0001009	Circulatory System	UBERON:0001768	vascular layer of eyeball	CNhs12097	Trabecular Meshwork Cells, donor2
UBERON:0001009	Circulatory System	UBERON:0001768	vascular layer of eyeball	CNhs12124	Trabecular Meshwork Cells, donor3
UBERON:0001009	Circulatory System	UBERON:0001769	iris	CNhs12596	Iris Pigment Epithelial Cells, donor1
UBERON:0001009	Circulatory System	UBERON:0001775	ciliary body	CNhs10871	Ciliary Epithelial Cells, donor1
UBERON:0001009	Circulatory System	UBERON:0001775	ciliary body	CNhs11966	Ciliary Epithelial Cells, donor2
UBERON:0001009	Circulatory System	UBERON:0001775	ciliary body	CNhs12009	Ciliary Epithelial Cells, donor3
UBERON:0001009	Circulatory System	UBERON:0001778	ciliary epithelium	CNhs10871	Ciliary Epithelial Cells, donor1
UBERON:0001009	Circulatory System	UBERON:0001778	ciliary epithelium	CNhs11966	Ciliary Epithelial Cells, donor2
UBERON:0001009	Circulatory System	UBERON:0001778	ciliary epithelium	CNhs12009	Ciliary Epithelial Cells, donor3
UBERON:0001009	Circulatory System	UBERON:0001797	vitreous humor	CNhs13440	eye - vitreous humor, donor1
UBERON:0001009	Circulatory System	UBERON:0001886	choroid plexus	CNhs11319	Fibroblast - Choroid Plexus, donor1
UBERON:0001009	Circulatory System	UBERON:0001886	choroid plexus	CNhs12344	Fibroblast - Choroid Plexus, donor2
UBERON:0001009	Circulatory System	UBERON:0001886	choroid plexus	CNhs12620	Fibroblast - Choroid Plexus, donor3
UBERON:0001009	Circulatory System	UBERON:0001915	endothelium of capillary	CNhs11925	Endothelial Cells - Microvascular, donor1
UBERON:0001009	Circulatory System	UBERON:0001915	endothelium of capillary	CNhs11376	Endothelial Cells - Microvascular, donor2
UBERON:0001009	Circulatory System	UBERON:0001915	endothelium of capillary	CNhs12024	Endothelial Cells - Microvascular, donor3
UBERON:0001009	Circulatory System	UBERON:0001917	endothelium of artery	CNhs10837	Endothelial Cells - Aortic, donor0
UBERON:0001009	Circulatory System	UBERON:0001917	endothelium of artery	CNhs12495	Endothelial Cells - Aortic, donor1
UBERON:0001009	Circulatory System	UBERON:0001917	endothelium of artery	CNhs12496	Endothelial Cells - Artery, donor1
UBERON:0001009	Circulatory System	UBERON:0001917	endothelium of artery	CNhs11926	Endothelial Cells - Thoracic, donor1
UBERON:0001009	Circulatory System	UBERON:0001917	endothelium of artery	CNhs11375	Endothelial Cells - Aortic, donor2
UBERON:0001009	Circulatory System	UBERON:0001917	endothelium of artery	CNhs11977	Endothelial Cells - Artery, donor2
UBERON:0001009	Circulatory System	UBERON:0001917	endothelium of artery	CNhs11978	Endothelial Cells - Thoracic, donor2
UBERON:0001009	Circulatory System	UBERON:0001917	endothelium of artery	CNhs12022	Endothelial Cells - Aortic, donor3
UBERON:0001009	Circulatory System	UBERON:0001917	endothelium of artery	CNhs12023	Endothelial Cells - Artery, donor3
UBERON:0001009	Circulatory System	UBERON:0001981	blood vessel	CNhs11760	aorta, adult, pool1
UBERON:0001009	Circulatory System	UBERON:0001981	blood vessel	CNhs12843	artery, adult
UBERON:0001009	Circulatory System	UBERON:0001981	blood vessel	CNhs10837	Endothelial Cells - Aortic, donor0
UBERON:0001009	Circulatory System	UBERON:0001981	blood vessel	CNhs10838	Smooth Muscle Cells - Aortic, donor0
UBERON:0001009	Circulatory System	UBERON:0001981	blood vessel	CNhs10839	Smooth Muscle Cells - Umbilical artery, donor0
UBERON:0001009	Circulatory System	UBERON:0001981	blood vessel	CNhs10872	Endothelial Cells - Umbilical vein, donor1
UBERON:0001009	Circulatory System	UBERON:0001981	blood vessel	CNhs10874	Fibroblast - Aortic Adventitial, donor1
UBERON:0001009	Circulatory System	UBERON:0001981	blood vessel	CNhs10878	Fibroblast - Pulmonary Artery, donor1
UBERON:0001009	Circulatory System	UBERON:0001981	blood vessel	CNhs12495	Endothelial Cells - Aortic, donor1
UBERON:0001009	Circulatory System	UBERON:0001981	blood vessel	CNhs12496	Endothelial Cells - Artery, donor1
UBERON:0001009	Circulatory System	UBERON:0001981	blood vessel	CNhs11925	Endothelial Cells - Microvascular, donor1
UBERON:0001009	Circulatory System	UBERON:0001981	blood vessel	CNhs11926	Endothelial Cells - Thoracic, donor1
UBERON:0001009	Circulatory System	UBERON:0001981	blood vessel	CNhs12497	Endothelial Cells - Vein, donor1
UBERON:0001009	Circulatory System	UBERON:0001981	blood vessel	CNhs11085	Smooth Muscle Cells - Aortic, donor1
UBERON:0001009	Circulatory System	UBERON:0001981	blood vessel	CNhs11086	Smooth Muscle Cells - Brachiocephalic, donor1
UBERON:0001009	Circulatory System	UBERON:0001981	blood vessel	CNhs11087	Smooth Muscle Cells - Carotid, donor1

UBERON:0001009	Circulatory System	UBERON:0001981	blood vessel	CNhs11088	Smooth Muscle Cells - Coronary Artery, donor1
UBERON:0001009	Circulatory System	UBERON:0001981	blood vessel	CNhs11067	Smooth Muscle Cells - Internal Thoracic Artery, donor1
UBERON:0001009	Circulatory System	UBERON:0001981	blood vessel	CNhs11089	Smooth Muscle Cells - Pulmonary Artery, donor1
UBERON:0001009	Circulatory System	UBERON:0001981	blood vessel	CNhs11090	Smooth Muscle Cells - Subclavian Artery, donor1
UBERON:0001009	Circulatory System	UBERON:0001981	blood vessel	CNhs11091	Smooth Muscle Cells - Umbilical Artery, donor1
UBERON:0001009	Circulatory System	UBERON:0001981	blood vessel	CNhs11967	Endothelial Cells - Umbilical vein, donor2
UBERON:0001009	Circulatory System	UBERON:0001981	blood vessel	CNhs11968	Fibroblast - Aortic Adventitial, donor2
UBERON:0001009	Circulatory System	UBERON:0001981	blood vessel	CNhs11375	Endothelial Cells - Aortic, donor2
UBERON:0001009	Circulatory System	UBERON:0001981	blood vessel	CNhs11977	Endothelial Cells - Artery, donor2
UBERON:0001009	Circulatory System	UBERON:0001981	blood vessel	CNhs11376	Endothelial Cells - Microvascular, donor2
UBERON:0001009	Circulatory System	UBERON:0001981	blood vessel	CNhs11978	Endothelial Cells - Thoracic, donor2
UBERON:0001009	Circulatory System	UBERON:0001981	blood vessel	CNhs11377	Endothelial Cells - Vein, donor2
UBERON:0001009	Circulatory System	UBERON:0001981	blood vessel	CNhs11305	Smooth Muscle Cells - Aortic, donor2
UBERON:0001009	Circulatory System	UBERON:0001981	blood vessel	CNhs11985	Smooth Muscle Cells - Brachiocephalic, donor2
UBERON:0001009	Circulatory System	UBERON:0001981	blood vessel	CNhs11986	Smooth Muscle Cells - Carotid, donor2
UBERON:0001009	Circulatory System	UBERON:0001981	blood vessel	CNhs11987	Smooth Muscle Cells - Coronary Artery, donor2
UBERON:0001009	Circulatory System	UBERON:0001981	blood vessel	CNhs11988	Smooth Muscle Cells - Internal Thoracic Artery, donor2
UBERON:0001009	Circulatory System	UBERON:0001981	blood vessel	CNhs11989	Smooth Muscle Cells - Pulmonary Artery, donor2
UBERON:0001009	Circulatory System	UBERON:0001981	blood vessel	CNhs11990	Smooth Muscle Cells - Subclavian Artery, donor2
UBERON:0001009	Circulatory System	UBERON:0001981	blood vessel	CNhs11991	Smooth Muscle Cells - Umbilical Artery, donor2
UBERON:0001009	Circulatory System	UBERON:0001981	blood vessel	CNhs12010	Endothelial Cells - Umbilical vein, donor3
UBERON:0001009	Circulatory System	UBERON:0001981	blood vessel	CNhs12011	Fibroblast - Aortic Adventitial, donor3
UBERON:0001009	Circulatory System	UBERON:0001981	blood vessel	CNhs12022	Endothelial Cells - Aortic, donor3
UBERON:0001009	Circulatory System	UBERON:0001981	blood vessel	CNhs12023	Endothelial Cells - Artery, donor3
UBERON:0001009	Circulatory System	UBERON:0001981	blood vessel	CNhs12024	Endothelial Cells - Microvascular, donor3
UBERON:0001009	Circulatory System	UBERON:0001981	blood vessel	CNhs12026	Endothelial Cells - Vein, donor3
UBERON:0001009	Circulatory System	UBERON:0001981	blood vessel	CNhs11309	Smooth Muscle Cells - Aortic, donor3
UBERON:0001009	Circulatory System	UBERON:0001981	blood vessel	CNhs12043	Smooth Muscle Cells - Brachiocephalic, donor3
UBERON:0001009	Circulatory System	UBERON:0001981	blood vessel	CNhs12044	Smooth Muscle Cells - Carotid, donor3
UBERON:0001009	Circulatory System	UBERON:0001981	blood vessel	CNhs12045	Smooth Muscle Cells - Coronary Artery, donor3
UBERON:0001009	Circulatory System	UBERON:0001981	blood vessel	CNhs12046	Smooth Muscle Cells - Internal Thoracic Artery, donor3
UBERON:0001009	Circulatory System	UBERON:0001981	blood vessel	CNhs12047	Smooth Muscle Cells - Pulmonary Artery, donor3
UBERON:0001009	Circulatory System	UBERON:0001981	blood vessel	CNhs12048	Smooth Muscle Cells - Subclavian Artery, donor3
UBERON:0001009	Circulatory System	UBERON:0001981	blood vessel	CNhs12049	Smooth Muscle Cells - Umbilical Artery, donor3
UBERON:0001009	Circulatory System	UBERON:0001981	blood vessel	CNhs12075	Hepatic Sinusoidal Endothelial Cells, donor1
UBERON:0001009	Circulatory System	UBERON:0001981	blood vessel	CNhs12597	Smooth Muscle Cells - Umbilical Vein, donor1
UBERON:0001009	Circulatory System	UBERON:0001981	blood vessel	CNhs12092	Hepatic Sinusoidal Endothelial Cells, donor2
UBERON:0001009	Circulatory System	UBERON:0001981	blood vessel	CNhs12569	Smooth Muscle Cells - Umbilical Vein, donor2
UBERON:0001009	Circulatory System	UBERON:0001981	blood vessel	CNhs12625	Hepatic Sinusoidal Endothelial Cells, donor3
UBERON:0001009	Circulatory System	UBERON:0001981	blood vessel	CNhs13076	Smooth Muscle Cells - Umbilical Vein, donor3
UBERON:0001009	Circulatory System	UBERON:0001982	capillary	CNhs11925	Endothelial Cells - Microvascular, donor1
UBERON:0001009	Circulatory System	UBERON:0001982	capillary	CNhs11376	Endothelial Cells - Microvascular, donor2
UBERON:0001009	Circulatory System	UBERON:0001982	capillary	CNhs12024	Endothelial Cells - Microvascular, donor3
UBERON:0001009	Circulatory System	UBERON:0002012	pulmonary artery	CNhs10878	Fibroblast - Pulmonary Artery, donor1
UBERON:0001009	Circulatory System	UBERON:0002012	pulmonary artery	CNhs11089	Smooth Muscle Cells - Pulmonary Artery, donor1
UBERON:0001009	Circulatory System	UBERON:0002012	pulmonary artery	CNhs11989	Smooth Muscle Cells - Pulmonary Artery, donor2
UBERON:0001009	Circulatory System	UBERON:0002012	pulmonary artery	CNhs12047	Smooth Muscle Cells - Pulmonary Artery, donor3
UBERON:0001009	Circulatory System	UBERON:0002049	vasculature	CNhs11760	aorta, adult, pool1
UBERON:0001009	Circulatory System	UBERON:0002049	vasculature	CNhs12843	artery, adult
UBERON:0001009	Circulatory System	UBERON:0002049	vasculature	CNhs13440	eye - vitreous humor, donor1
UBERON:0001009	Circulatory System	UBERON:0002049	vasculature	CNhs10837	Endothelial Cells - Aortic, donor0
UBERON:0001009	Circulatory System	UBERON:0002049	vasculature	CNhs10838	Smooth Muscle Cells - Aortic, donor0
UBERON:0001009	Circulatory System	UBERON:0002049	vasculature	CNhs10839	Smooth Muscle Cells - Umbilical artery, donor0
UBERON:0001009	Circulatory System	UBERON:0002049	vasculature	CNhs10863	Smooth Muscle Cells - Brain Vascular, donor1
UBERON:0001009	Circulatory System	UBERON:0002049	vasculature	CNhs10865	Endothelial Cells - Lymphatic, donor1
UBERON:0001009	Circulatory System	UBERON:0002049	vasculature	CNhs10871	Ciliary Epithelial Cells, donor1
UBERON:0001009	Circulatory System	UBERON:0002049	vasculature	CNhs10872	Endothelial Cells - Umbilical vein, donor1
UBERON:0001009	Circulatory System	UBERON:0002049	vasculature	CNhs10874	Fibroblast - Aortic Adventitial, donor1
UBERON:0001009	Circulatory System	UBERON:0002049	vasculature	CNhs10878	Fibroblast - Pulmonary Artery, donor1
UBERON:0001009	Circulatory System	UBERON:0002049	vasculature	CNhs12495	Endothelial Cells - Aortic, donor1
UBERON:0001009	Circulatory System	UBERON:0002049	vasculature	CNhs12496	Endothelial Cells - Artery, donor1
UBERON:0001009	Circulatory System	UBERON:0002049	vasculature	CNhs11925	Endothelial Cells - Microvascular, donor1
UBERON:0001009	Circulatory System	UBERON:0002049	vasculature	CNhs11926	Endothelial Cells - Thoracic, donor1
UBERON:0001009	Circulatory System	UBERON:0002049	vasculature	CNhs12497	Endothelial Cells - Vein, donor1
UBERON:0001009	Circulatory System	UBERON:0002049	vasculature	CNhs11085	Smooth Muscle Cells - Aortic, donor1
UBERON:0001009	Circulatory System	UBERON:0002049	vasculature	CNhs11086	Smooth Muscle Cells - Brachiocephalic, donor1
UBERON:0001009	Circulatory System	UBERON:0002049	vasculature	CNhs11087	Smooth Muscle Cells - Carotid, donor1
UBERON:0001009	Circulatory System	UBERON:0002049	vasculature	CNhs11088	Smooth Muscle Cells - Coronary Artery, donor1
UBERON:0001009	Circulatory System	UBERON:0002049	vasculature	CNhs11067	Smooth Muscle Cells - Internal Thoracic Artery, donor1
UBERON:0001009	Circulatory System	UBERON:0002049	vasculature	CNhs11089	Smooth Muscle Cells - Pulmonary Artery, donor1
UBERON:0001009	Circulatory System	UBERON:0002049	vasculature	CNhs11090	Smooth Muscle Cells - Subclavian Artery, donor1
UBERON:0001009	Circulatory System	UBERON:0002049	vasculature	CNhs11091	Smooth Muscle Cells - Umbilical Artery, donor1
UBERON:0001009	Circulatory System	UBERON:0002049	vasculature	CNhs11900	Smooth Muscle Cells - Brain Vascular, donor2
UBERON:0001009	Circulatory System	UBERON:0002049	vasculature	CNhs11901	Endothelial Cells - Lymphatic, donor2
UBERON:0001009	Circulatory System	UBERON:0002049	vasculature	CNhs11966	Ciliary Epithelial Cells, donor2
UBERON:0001009	Circulatory System	UBERON:0002049	vasculature	CNhs11967	Endothelial Cells - Umbilical vein, donor2
UBERON:0001009	Circulatory System	UBERON:0002049	vasculature	CNhs11968	Fibroblast - Aortic Adventitial, donor2
UBERON:0001009	Circulatory System	UBERON:0002049	vasculature	CNhs11375	Endothelial Cells - Aortic, donor2
UBERON:0001009	Circulatory System	UBERON:0002049	vasculature	CNhs11977	Endothelial Cells - Artery, donor2
UBERON:0001009	Circulatory System	UBERON:0002049	vasculature	CNhs11376	Endothelial Cells - Microvascular, donor2
UBERON:0001009	Circulatory System	UBERON:0002049	vasculature	CNhs11978	Endothelial Cells - Thoracic, donor2
UBERON:0001009	Circulatory System	UBERON:0002049	vasculature	CNhs11377	Endothelial Cells - Vein, donor2
UBERON:0001009	Circulatory System	UBERON:0002049	vasculature	CNhs11305	Smooth Muscle Cells - Aortic, donor2
UBERON:0001009	Circulatory System	UBERON:0002049	vasculature	CNhs11985	Smooth Muscle Cells - Brachiocephalic, donor2
UBERON:0001009	Circulatory System	UBERON:0002049	vasculature	CNhs11986	Smooth Muscle Cells - Carotid, donor2
UBERON:0001009	Circulatory System	UBERON:0002049	vasculature	CNhs11987	Smooth Muscle Cells - Coronary Artery, donor2
UBERON:0001009	Circulatory System	UBERON:0002049	vasculature	CNhs11988	Smooth Muscle Cells - Internal Thoracic Artery, donor2
UBERON:0001009	Circulatory System	UBERON:0002049	vasculature	CNhs11989	Smooth Muscle Cells - Pulmonary Artery, donor2
UBERON:0001009	Circulatory System	UBERON:0002049	vasculature	CNhs11990	Smooth Muscle Cells - Subclavian Artery, donor2
UBERON:0001009	Circulatory System	UBERON:0002049	vasculature	CNhs11991	Smooth Muscle Cells - Umbilical Artery, donor2
UBERON:0001009	Circulatory System	UBERON:0002049	vasculature	CNhs12004	Smooth Muscle Cells - Brain Vascular, donor3
UBERON:0001009	Circulatory System	UBERON:0002049	vasculature	CNhs11906	Endothelial Cells - Lymphatic, donor3
UBERON:0001009	Circulatory System	UBERON:0002049	vasculature	CNhs12009	Ciliary Epithelial Cells, donor3
UBERON:0001009	Circulatory System	UBERON:0002049	vasculature	CNhs12010	Endothelial Cells - Umbilical vein, donor3
UBERON:0001009	Circulatory System	UBERON:0002049	vasculature	CNhs12011	Fibroblast - Aortic Adventitial, donor3
UBERON:0001009	Circulatory System	UBERON:0002049	vasculature	CNhs12022	Endothelial Cells - Aortic, donor3
UBERON:0001009	Circulatory System	UBERON:0002049	vasculature	CNhs12023	Endothelial Cells - Artery, donor3
UBERON:0001009	Circulatory System	UBERON:0002049	vasculature	CNhs12024	Endothelial Cells - Microvascular, donor3

UBERON:0001009	Circulatory System	UBERON:0002049	vasculature	CNhs12026	Endothelial Cells - Vein, donor3
UBERON:0001009	Circulatory System	UBERON:0002049	vasculature	CNhs11309	Smooth Muscle Cells - Aortic, donor3
UBERON:0001009	Circulatory System	UBERON:0002049	vasculature	CNhs12043	Smooth Muscle Cells - Brachiocephalic, donor3
UBERON:0001009	Circulatory System	UBERON:0002049	vasculature	CNhs12044	Smooth Muscle Cells - Carotid, donor3
UBERON:0001009	Circulatory System	UBERON:0002049	vasculature	CNhs12045	Smooth Muscle Cells - Coronary Artery, donor3
UBERON:0001009	Circulatory System	UBERON:0002049	vasculature	CNhs12046	Smooth Muscle Cells - Internal Thoracic Artery, donor3
UBERON:0001009	Circulatory System	UBERON:0002049	vasculature	CNhs12047	Smooth Muscle Cells - Pulmonary Artery, donor3
UBERON:0001009	Circulatory System	UBERON:0002049	vasculature	CNhs12048	Smooth Muscle Cells - Subclavian Artery, donor3
UBERON:0001009	Circulatory System	UBERON:0002049	vasculature	CNhs12049	Smooth Muscle Cells - Umbilical Artery, donor3
UBERON:0001009	Circulatory System	UBERON:0002049	vasculature	CNhs11319	Fibroblast - Choroid Plexus, donor1
UBERON:0001009	Circulatory System	UBERON:0002049	vasculature	CNhs11322	Fibroblast - Lymphatic, donor1
UBERON:0001009	Circulatory System	UBERON:0002049	vasculature	CNhs12075	Hepatic Sinusoidal Endothelial Cells, donor1
UBERON:0001009	Circulatory System	UBERON:0002049	vasculature	CNhs12596	Iris Pigment Epithelial Cells, donor1
UBERON:0001009	Circulatory System	UBERON:0002049	vasculature	CNhs11340	Trabecular Meshwork Cells, donor1
UBERON:0001009	Circulatory System	UBERON:0002049	vasculature	CNhs12597	Smooth Muscle Cells - Umbilical Vein, donor1
UBERON:0001009	Circulatory System	UBERON:0002049	vasculature	CNhs12344	Fibroblast - Choroid Plexus, donor2
UBERON:0001009	Circulatory System	UBERON:0002049	vasculature	CNhs12082	Fibroblast - Lymphatic, donor2
UBERON:0001009	Circulatory System	UBERON:0002049	vasculature	CNhs12092	Hepatic Sinusoidal Endothelial Cells, donor2
UBERON:0001009	Circulatory System	UBERON:0002049	vasculature	CNhs12097	Trabecular Meshwork Cells, donor2
UBERON:0001009	Circulatory System	UBERON:0002049	vasculature	CNhs12569	Smooth Muscle Cells - Umbilical Vein, donor2
UBERON:0001009	Circulatory System	UBERON:0002049	vasculature	CNhs12620	Fibroblast - Choroid Plexus, donor3
UBERON:0001009	Circulatory System	UBERON:0002049	vasculature	CNhs12118	Fibroblast - Lymphatic, donor3
UBERON:0001009	Circulatory System	UBERON:0002049	vasculature	CNhs12625	Hepatic Sinusoidal Endothelial Cells, donor3
UBERON:0001009	Circulatory System	UBERON:0002049	vasculature	CNhs12124	Trabecular Meshwork Cells, donor3
UBERON:0001009	Circulatory System	UBERON:0002049	vasculature	CNhs13076	Smooth Muscle Cells - Umbilical Vein, donor3
UBERON:0001009	Circulatory System	UBERON:0002066	umbilical vein	CNhs10872	Endothelial Cells - Umbilical vein, donor1
UBERON:0001009	Circulatory System	UBERON:0002066	umbilical vein	CNhs11967	Endothelial Cells - Umbilical vein, donor2
UBERON:0001009	Circulatory System	UBERON:0002066	umbilical vein	CNhs12010	Endothelial Cells - Umbilical vein, donor3
UBERON:0001009	Circulatory System	UBERON:0002066	umbilical vein	CNhs12597	Smooth Muscle Cells - Umbilical Vein, donor1
UBERON:0001009	Circulatory System	UBERON:0002066	umbilical vein	CNhs12569	Smooth Muscle Cells - Umbilical Vein, donor2
UBERON:0001009	Circulatory System	UBERON:0002066	umbilical vein	CNhs13076	Smooth Muscle Cells - Umbilical Vein, donor3
UBERON:0001009	Circulatory System	UBERON:0002078	right atrium	CNhs12857	heart - tricuspid valve, adult
UBERON:0001009	Circulatory System	UBERON:0002079	left atrium	CNhs11790	left atrium, adult, donor1
UBERON:0001009	Circulatory System	UBERON:0002081	cardiac atrium	CNhs11790	left atrium, adult, donor1
UBERON:0001009	Circulatory System	UBERON:0002081	cardiac atrium	CNhs12857	heart - tricuspid valve, adult
UBERON:0001009	Circulatory System	UBERON:0002082	cardiac ventricle	CNhs11789	left ventricle, adult, donor1
UBERON:0001009	Circulatory System	UBERON:0002084	heart left ventricle	CNhs11789	left ventricle, adult, donor1
UBERON:0001009	Circulatory System	UBERON:0002111	artery smooth muscle tissue	CNhs10838	Smooth Muscle Cells - Aortic, donor0
UBERON:0001009	Circulatory System	UBERON:0002111	artery smooth muscle tissue	CNhs11085	Smooth Muscle Cells - Aortic, donor1
UBERON:0001009	Circulatory System	UBERON:0002111	artery smooth muscle tissue	CNhs11305	Smooth Muscle Cells - Aortic, donor2
UBERON:0001009	Circulatory System	UBERON:0002111	artery smooth muscle tissue	CNhs11309	Smooth Muscle Cells - Aortic, donor3
UBERON:0001009	Circulatory System	UBERON:0002133	atrioventricular valve	CNhs12855	heart - mitral valve, adult
UBERON:0001009	Circulatory System	UBERON:0002133	atrioventricular valve	CNhs12857	heart - tricuspid valve, adult
UBERON:0001009	Circulatory System	UBERON:0002134	tricuspid valve	CNhs12857	heart - tricuspid valve, adult
UBERON:0001009	Circulatory System	UBERON:0002135	mitral valve	CNhs12855	heart - mitral valve, adult
UBERON:0001009	Circulatory System	UBERON:0002146	pulmonary valve	CNhs12856	heart - pulmonic valve, adult
UBERON:0001009	Circulatory System	UBERON:0002200	vasculature of head	CNhs13440	eye - vitreous humor, donor1
UBERON:0001009	Circulatory System	UBERON:0002200	vasculature of head	CNhs10871	Ciliary Epithelial Cells, donor1
UBERON:0001009	Circulatory System	UBERON:0002200	vasculature of head	CNhs11966	Ciliary Epithelial Cells, donor2
UBERON:0001009	Circulatory System	UBERON:0002200	vasculature of head	CNhs12009	Ciliary Epithelial Cells, donor3
UBERON:0001009	Circulatory System	UBERON:0002200	vasculature of head	CNhs12596	Iris Pigment Epithelial Cells, donor1
UBERON:0001009	Circulatory System	UBERON:0002200	vasculature of head	CNhs11340	Trabecular Meshwork Cells, donor1
UBERON:0001009	Circulatory System	UBERON:0002200	vasculature of head	CNhs12097	Trabecular Meshwork Cells, donor2
UBERON:0001009	Circulatory System	UBERON:0002200	vasculature of head	CNhs12124	Trabecular Meshwork Cells, donor3
UBERON:0001009	Circulatory System	UBERON:0002203	vasculature of eye	CNhs13440	eye - vitreous humor, donor1
UBERON:0001009	Circulatory System	UBERON:0002203	vasculature of eye	CNhs10871	Ciliary Epithelial Cells, donor1
UBERON:0001009	Circulatory System	UBERON:0002203	vasculature of eye	CNhs11966	Ciliary Epithelial Cells, donor2
UBERON:0001009	Circulatory System	UBERON:0002203	vasculature of eye	CNhs12009	Ciliary Epithelial Cells, donor3
UBERON:0001009	Circulatory System	UBERON:0002203	vasculature of eye	CNhs12596	Iris Pigment Epithelial Cells, donor1
UBERON:0001009	Circulatory System	UBERON:0002203	vasculature of eye	CNhs11340	Trabecular Meshwork Cells, donor1
UBERON:0001009	Circulatory System	UBERON:0002203	vasculature of eye	CNhs12097	Trabecular Meshwork Cells, donor2
UBERON:0001009	Circulatory System	UBERON:0002203	vasculature of eye	CNhs12124	Trabecular Meshwork Cells, donor3
UBERON:0001009	Circulatory System	UBERON:0002456	internal thoracic artery	CNhs11067	Smooth Muscle Cells - Internal Thoracic Artery, donor1
UBERON:0001009	Circulatory System	UBERON:0002456	internal thoracic artery	CNhs11988	Smooth Muscle Cells - Internal Thoracic Artery, donor2
UBERON:0001009	Circulatory System	UBERON:0002456	internal thoracic artery	CNhs12046	Smooth Muscle Cells - Internal Thoracic Artery, donor3
UBERON:0001009	Circulatory System	UBERON:0002506	iris epithelium	CNhs12596	Iris Pigment Epithelial Cells, donor1
UBERON:0001009	Circulatory System	UBERON:0003497	abdomen blood vessel	CNhs12075	Hepatic Sinusoidal Endothelial Cells, donor1
UBERON:0001009	Circulatory System	UBERON:0003497	abdomen blood vessel	CNhs12092	Hepatic Sinusoidal Endothelial Cells, donor2
UBERON:0001009	Circulatory System	UBERON:0003497	abdomen blood vessel	CNhs12625	Hepatic Sinusoidal Endothelial Cells, donor3
UBERON:0001009	Circulatory System	UBERON:0003498	heart blood vessel	CNhs11088	Smooth Muscle Cells - Coronary Artery, donor1
UBERON:0001009	Circulatory System	UBERON:0003498	heart blood vessel	CNhs11987	Smooth Muscle Cells - Coronary Artery, donor2
UBERON:0001009	Circulatory System	UBERON:0003498	heart blood vessel	CNhs12045	Smooth Muscle Cells - Coronary Artery, donor3
UBERON:0001009	Circulatory System	UBERON:0003509	arterial blood vessel	CNhs11760	aorta, adult, pool1
UBERON:0001009	Circulatory System	UBERON:0003509	arterial blood vessel	CNhs12843	artery, adult
UBERON:0001009	Circulatory System	UBERON:0003509	arterial blood vessel	CNhs10837	Endothelial Cells - Aortic, donor0
UBERON:0001009	Circulatory System	UBERON:0003509	arterial blood vessel	CNhs10838	Smooth Muscle Cells - Aortic, donor0
UBERON:0001009	Circulatory System	UBERON:0003509	arterial blood vessel	CNhs10839	Smooth Muscle Cells - Umbilical artery, donor0
UBERON:0001009	Circulatory System	UBERON:0003509	arterial blood vessel	CNhs10874	Fibroblast - Aortic Adventitial, donor1
UBERON:0001009	Circulatory System	UBERON:0003509	arterial blood vessel	CNhs10878	Fibroblast - Pulmonary Artery, donor1
UBERON:0001009	Circulatory System	UBERON:0003509	arterial blood vessel	CNhs12495	Endothelial Cells - Aortic, donor1
UBERON:0001009	Circulatory System	UBERON:0003509	arterial blood vessel	CNhs12496	Endothelial Cells - Artery, donor1
UBERON:0001009	Circulatory System	UBERON:0003509	arterial blood vessel	CNhs11926	Endothelial Cells - Thoracic, donor1
UBERON:0001009	Circulatory System	UBERON:0003509	arterial blood vessel	CNhs11085	Smooth Muscle Cells - Aortic, donor1
UBERON:0001009	Circulatory System	UBERON:0003509	arterial blood vessel	CNhs11086	Smooth Muscle Cells - Brachiocephalic, donor1
UBERON:0001009	Circulatory System	UBERON:0003509	arterial blood vessel	CNhs11087	Smooth Muscle Cells - Carotid, donor1
UBERON:0001009	Circulatory System	UBERON:0003509	arterial blood vessel	CNhs11088	Smooth Muscle Cells - Coronary Artery, donor1
UBERON:0001009	Circulatory System	UBERON:0003509	arterial blood vessel	CNhs11067	Smooth Muscle Cells - Internal Thoracic Artery, donor1
UBERON:0001009	Circulatory System	UBERON:0003509	arterial blood vessel	CNhs11089	Smooth Muscle Cells - Pulmonary Artery, donor1
UBERON:0001009	Circulatory System	UBERON:0003509	arterial blood vessel	CNhs11090	Smooth Muscle Cells - Subclavian Artery, donor1
UBERON:0001009	Circulatory System	UBERON:0003509	arterial blood vessel	CNhs11091	Smooth Muscle Cells - Umbilical Artery, donor1
UBERON:0001009	Circulatory System	UBERON:0003509	arterial blood vessel	CNhs11968	Fibroblast - Aortic Adventitial, donor2
UBERON:0001009	Circulatory System	UBERON:0003509	arterial blood vessel	CNhs11375	Endothelial Cells - Aortic, donor2
UBERON:0001009	Circulatory System	UBERON:0003509	arterial blood vessel	CNhs11977	Endothelial Cells - Artery, donor2
UBERON:0001009	Circulatory System	UBERON:0003509	arterial blood vessel	CNhs11978	Endothelial Cells - Thoracic, donor2
UBERON:0001009	Circulatory System	UBERON:0003509	arterial blood vessel	CNhs11305	Smooth Muscle Cells - Aortic, donor2
UBERON:0001009	Circulatory System	UBERON:0003509	arterial blood vessel	CNhs11985	Smooth Muscle Cells - Brachiocephalic, donor2
UBERON:0001009	Circulatory System	UBERON:0003509	arterial blood vessel	CNhs11986	Smooth Muscle Cells - Carotid, donor2
UBERON:0001009	Circulatory System	UBERON:0003509	arterial blood vessel	CNhs11987	Smooth Muscle Cells - Coronary Artery, donor2

UBERON:0001009	Circulatory System	UBERON:0003509	arterial blood vessel	CNhs11988	Smooth Muscle Cells - Internal Thoracic Artery, donor2
UBERON:0001009	Circulatory System	UBERON:0003509	arterial blood vessel	CNhs11989	Smooth Muscle Cells - Pulmonary Artery, donor2
UBERON:0001009	Circulatory System	UBERON:0003509	arterial blood vessel	CNhs11990	Smooth Muscle Cells - Subclavian Artery, donor2
UBERON:0001009	Circulatory System	UBERON:0003509	arterial blood vessel	CNhs11991	Smooth Muscle Cells - Umbilical Artery, donor2
UBERON:0001009	Circulatory System	UBERON:0003509	arterial blood vessel	CNhs12011	Fibroblast - Aortic Adventitial, donor3
UBERON:0001009	Circulatory System	UBERON:0003509	arterial blood vessel	CNhs12022	Endothelial Cells - Aortic, donor3
UBERON:0001009	Circulatory System	UBERON:0003509	arterial blood vessel	CNhs12023	Endothelial Cells - Artery, donor3
UBERON:0001009	Circulatory System	UBERON:0003509	arterial blood vessel	CNhs11309	Smooth Muscle Cells - Aortic, donor3
UBERON:0001009	Circulatory System	UBERON:0003509	arterial blood vessel	CNhs12043	Smooth Muscle Cells - Brachiocephalic, donor3
UBERON:0001009	Circulatory System	UBERON:0003509	arterial blood vessel	CNhs12044	Smooth Muscle Cells - Carotid, donor3
UBERON:0001009	Circulatory System	UBERON:0003509	arterial blood vessel	CNhs12045	Smooth Muscle Cells - Coronary Artery, donor3
UBERON:0001009	Circulatory System	UBERON:0003509	arterial blood vessel	CNhs12046	Smooth Muscle Cells - Internal Thoracic Artery, donor3
UBERON:0001009	Circulatory System	UBERON:0003509	arterial blood vessel	CNhs12047	Smooth Muscle Cells - Pulmonary Artery, donor3
UBERON:0001009	Circulatory System	UBERON:0003509	arterial blood vessel	CNhs12048	Smooth Muscle Cells - Subclavian Artery, donor3
UBERON:0001009	Circulatory System	UBERON:0003509	arterial blood vessel	CNhs12049	Smooth Muscle Cells - Umbilical Artery, donor3
UBERON:0001009	Circulatory System	UBERON:0003513	trunk blood vessel	CNhs11067	Smooth Muscle Cells - Internal Thoracic Artery, donor1
UBERON:0001009	Circulatory System	UBERON:0003513	trunk blood vessel	CNhs11090	Smooth Muscle Cells - Subclavian Artery, donor1
UBERON:0001009	Circulatory System	UBERON:0003513	trunk blood vessel	CNhs11988	Smooth Muscle Cells - Internal Thoracic Artery, donor2
UBERON:0001009	Circulatory System	UBERON:0003513	trunk blood vessel	CNhs11990	Smooth Muscle Cells - Subclavian Artery, donor2
UBERON:0001009	Circulatory System	UBERON:0003513	trunk blood vessel	CNhs12046	Smooth Muscle Cells - Internal Thoracic Artery, donor3
UBERON:0001009	Circulatory System	UBERON:0003513	trunk blood vessel	CNhs12048	Smooth Muscle Cells - Subclavian Artery, donor3
UBERON:0001009	Circulatory System	UBERON:0003513	trunk blood vessel	CNhs12075	Hepatic Sinusoidal Endothelial Cells, donor1
UBERON:0001009	Circulatory System	UBERON:0003513	trunk blood vessel	CNhs12092	Hepatic Sinusoidal Endothelial Cells, donor2
UBERON:0001009	Circulatory System	UBERON:0003513	trunk blood vessel	CNhs12625	Hepatic Sinusoidal Endothelial Cells, donor3
UBERON:0001009	Circulatory System	UBERON:0003834	thoracic segment blood vessel	CNhs11067	Smooth Muscle Cells - Internal Thoracic Artery, donor1
UBERON:0001009	Circulatory System	UBERON:0003834	thoracic segment blood vessel	CNhs11090	Smooth Muscle Cells - Subclavian Artery, donor1
UBERON:0001009	Circulatory System	UBERON:0003834	thoracic segment blood vessel	CNhs11988	Smooth Muscle Cells - Internal Thoracic Artery, donor2
UBERON:0001009	Circulatory System	UBERON:0003834	thoracic segment blood vessel	CNhs11990	Smooth Muscle Cells - Subclavian Artery, donor2
UBERON:0001009	Circulatory System	UBERON:0003834	thoracic segment blood vessel	CNhs12046	Smooth Muscle Cells - Internal Thoracic Artery, donor3
UBERON:0001009	Circulatory System	UBERON:0003834	thoracic segment blood vessel	CNhs12048	Smooth Muscle Cells - Subclavian Artery, donor3
UBERON:0001009	Circulatory System	UBERON:0003835	abdominal segment blood vessel	CNhs12075	Hepatic Sinusoidal Endothelial Cells, donor1
UBERON:0001009	Circulatory System	UBERON:0003835	abdominal segment blood vessel	CNhs12092	Hepatic Sinusoidal Endothelial Cells, donor2
UBERON:0001009	Circulatory System	UBERON:0003835	abdominal segment blood vessel	CNhs12625	Hepatic Sinusoidal Endothelial Cells, donor3
UBERON:0001009	Circulatory System	UBERON:0003909	sinusoid	CNhs12075	Hepatic Sinusoidal Endothelial Cells, donor1
UBERON:0001009	Circulatory System	UBERON:0003909	sinusoid	CNhs12092	Hepatic Sinusoidal Endothelial Cells, donor2
UBERON:0001009	Circulatory System	UBERON:0003909	sinusoid	CNhs12625	Hepatic Sinusoidal Endothelial Cells, donor3
UBERON:0001009	Circulatory System	UBERON:0003920	venous blood vessel	CNhs10872	Endothelial Cells - Umbilical vein, donor1
UBERON:0001009	Circulatory System	UBERON:0003920	venous blood vessel	CNhs12497	Endothelial Cells - Vein, donor1
UBERON:0001009	Circulatory System	UBERON:0003920	venous blood vessel	CNhs11967	Endothelial Cells - Umbilical vein, donor2
UBERON:0001009	Circulatory System	UBERON:0003920	venous blood vessel	CNhs11377	Endothelial Cells - Vein, donor2
UBERON:0001009	Circulatory System	UBERON:0003920	venous blood vessel	CNhs12010	Endothelial Cells - Umbilical vein, donor3
UBERON:0001009	Circulatory System	UBERON:0003920	venous blood vessel	CNhs12026	Endothelial Cells - Vein, donor3
UBERON:0001009	Circulatory System	UBERON:0003920	venous blood vessel	CNhs12597	Smooth Muscle Cells - Umbilical Vein, donor1
UBERON:0001009	Circulatory System	UBERON:0003920	venous blood vessel	CNhs12569	Smooth Muscle Cells - Umbilical Vein, donor2
UBERON:0001009	Circulatory System	UBERON:0003920	venous blood vessel	CNhs13076	Smooth Muscle Cells - Umbilical Vein, donor3
UBERON:0001009	Circulatory System	UBERON:0003956	aqueous drainage system	CNhs11340	Trabecular Meshwork Cells, donor1
UBERON:0001009	Circulatory System	UBERON:0003956	aqueous drainage system	CNhs12097	Trabecular Meshwork Cells, donor2
UBERON:0001009	Circulatory System	UBERON:0003956	aqueous drainage system	CNhs12124	Trabecular Meshwork Cells, donor3
UBERON:0001009	Circulatory System	UBERON:0004145	outflow tract	CNhs12857	heart - tricuspid valve, adult
UBERON:0001009	Circulatory System	UBERON:0004151	cardiac chamber	CNhs11789	left ventricle, adult, donor1
UBERON:0001009	Circulatory System	UBERON:0004151	cardiac chamber	CNhs11790	left atrium, adult, donor1
UBERON:0001009	Circulatory System	UBERON:0004151	cardiac chamber	CNhs12857	heart - tricuspid valve, adult
UBERON:0001009	Circulatory System	UBERON:0004178	aorta smooth muscle tissue	CNhs10838	Smooth Muscle Cells - Aortic, donor0
UBERON:0001009	Circulatory System	UBERON:0004178	aorta smooth muscle tissue	CNhs11085	Smooth Muscle Cells - Aortic, donor1
UBERON:0001009	Circulatory System	UBERON:0004178	aorta smooth muscle tissue	CNhs11305	Smooth Muscle Cells - Aortic, donor2
UBERON:0001009	Circulatory System	UBERON:0004178	aorta smooth muscle tissue	CNhs11309	Smooth Muscle Cells - Aortic, donor3
UBERON:0001009	Circulatory System	UBERON:0004237	blood vessel smooth muscle	CNhs10838	Smooth Muscle Cells - Aortic, donor0
UBERON:0001009	Circulatory System	UBERON:0004237	blood vessel smooth muscle	CNhs11085	Smooth Muscle Cells - Aortic, donor1
UBERON:0001009	Circulatory System	UBERON:0004237	blood vessel smooth muscle	CNhs11305	Smooth Muscle Cells - Aortic, donor2
UBERON:0001009	Circulatory System	UBERON:0004237	blood vessel smooth muscle	CNhs11309	Smooth Muscle Cells - Aortic, donor3
UBERON:0001009	Circulatory System	UBERON:0004535	cardiovascular system	CNhs10653	heart, fetal, pool1
UBERON:0001009	Circulatory System	UBERON:0004535	cardiovascular system	CNhs11760	aorta, adult, pool1
UBERON:0001009	Circulatory System	UBERON:0004535	cardiovascular system	CNhs11761	blood, adult, pool1
UBERON:0001009	Circulatory System	UBERON:0004535	cardiovascular system	CNhs11789	left ventricle, adult, donor1
UBERON:0001009	Circulatory System	UBERON:0004535	cardiovascular system	CNhs11790	left atrium, adult, donor1
UBERON:0001009	Circulatory System	UBERON:0004535	cardiovascular system	CNhs12843	artery, adult
UBERON:0001009	Circulatory System	UBERON:0004535	cardiovascular system	CNhs12855	heart - mitral valve, adult
UBERON:0001009	Circulatory System	UBERON:0004535	cardiovascular system	CNhs12856	heart - pulmonic valve, adult
UBERON:0001009	Circulatory System	UBERON:0004535	cardiovascular system	CNhs12857	heart - tricuspid valve, adult
UBERON:0001009	Circulatory System	UBERON:0004535	cardiovascular system	CNhs13440	eye - vitreous humor, donor1
UBERON:0001009	Circulatory System	UBERON:0004535	cardiovascular system	CNhs10837	Endothelial Cells - Aortic, donor0
UBERON:0001009	Circulatory System	UBERON:0004535	cardiovascular system	CNhs10838	Smooth Muscle Cells - Aortic, donor0
UBERON:0001009	Circulatory System	UBERON:0004535	cardiovascular system	CNhs10839	Smooth Muscle Cells - Umbilical artery, donor0
UBERON:0001009	Circulatory System	UBERON:0004535	cardiovascular system	CNhs10860	Peripheral Blood Mononuclear Cells, donor1
UBERON:0001009	Circulatory System	UBERON:0004535	cardiovascular system	CNhs10863	Smooth Muscle Cells - Brain Vascular, donor1
UBERON:0001009	Circulatory System	UBERON:0004535	cardiovascular system	CNhs10865	Endothelial Cells - Lymphatic, donor1
UBERON:0001009	Circulatory System	UBERON:0004535	cardiovascular system	CNhs10871	Ciliary Epithelial Cells, donor1
UBERON:0001009	Circulatory System	UBERON:0004535	cardiovascular system	CNhs10872	Endothelial Cells - Umbilical vein, donor1
UBERON:0001009	Circulatory System	UBERON:0004535	cardiovascular system	CNhs10874	Fibroblast - Aortic Adventitial, donor1
UBERON:0001009	Circulatory System	UBERON:0004535	cardiovascular system	CNhs10878	Fibroblast - Pulmonary Artery, donor1
UBERON:0001009	Circulatory System	UBERON:0004535	cardiovascular system	CNhs12495	Endothelial Cells - Aortic, donor1
UBERON:0001009	Circulatory System	UBERON:0004535	cardiovascular system	CNhs12496	Endothelial Cells - Artery, donor1
UBERON:0001009	Circulatory System	UBERON:0004535	cardiovascular system	CNhs11925	Endothelial Cells - Microvascular, donor1
UBERON:0001009	Circulatory System	UBERON:0004535	cardiovascular system	CNhs11926	Endothelial Cells - Thoracic, donor1
UBERON:0001009	Circulatory System	UBERON:0004535	cardiovascular system	CNhs12497	Endothelial Cells - Vein, donor1
UBERON:0001009	Circulatory System	UBERON:0004535	cardiovascular system	CNhs12498	Fibroblast - Cardiac, donor1
UBERON:0001009	Circulatory System	UBERON:0004535	cardiovascular system	CNhs11085	Smooth Muscle Cells - Aortic, donor1
UBERON:0001009	Circulatory System	UBERON:0004535	cardiovascular system	CNhs11086	Smooth Muscle Cells - Brachiocephalic, donor1
UBERON:0001009	Circulatory System	UBERON:0004535	cardiovascular system	CNhs11087	Smooth Muscle Cells - Carotid, donor1
UBERON:0001009	Circulatory System	UBERON:0004535	cardiovascular system	CNhs11088	Smooth Muscle Cells - Coronary Artery, donor1
UBERON:0001009	Circulatory System	UBERON:0004535	cardiovascular system	CNhs11067	Smooth Muscle Cells - Internal Thoracic Artery, donor1
UBERON:0001009	Circulatory System	UBERON:0004535	cardiovascular system	CNhs11089	Smooth Muscle Cells - Pulmonary Artery, donor1
UBERON:0001009	Circulatory System	UBERON:0004535	cardiovascular system	CNhs11090	Smooth Muscle Cells - Subclavian Artery, donor1
UBERON:0001009	Circulatory System	UBERON:0004535	cardiovascular system	CNhs11091	Smooth Muscle Cells - Umbilical Artery, donor1
UBERON:0001009	Circulatory System	UBERON:0004535	cardiovascular system	CNhs11958	Peripheral Blood Mononuclear Cells, donor2
UBERON:0001009	Circulatory System	UBERON:0004535	cardiovascular system	CNhs11900	Smooth Muscle Cells - Brain Vascular, donor2
UBERON:0001009	Circulatory System	UBERON:0004535	cardiovascular system	CNhs11901	Endothelial Cells - Lymphatic, donor2
UBERON:0001009	Circulatory System	UBERON:0004535	cardiovascular system	CNhs11966	Ciliary Epithelial Cells, donor2

UBERON:0001009	Circulatory System	UBERON:0004535	cardiovascular system	CNhs11967	Endothelial Cells - Umbilical vein, donor2
UBERON:0001009	Circulatory System	UBERON:0004535	cardiovascular system	CNhs11968	Fibroblast - Aortic Adventitial, donor2
UBERON:0001009	Circulatory System	UBERON:0004535	cardiovascular system	CNhs11375	Endothelial Cells - Aortic, donor2
UBERON:0001009	Circulatory System	UBERON:0004535	cardiovascular system	CNhs11977	Endothelial Cells - Artery, donor2
UBERON:0001009	Circulatory System	UBERON:0004535	cardiovascular system	CNhs11376	Endothelial Cells - Microvascular, donor2
UBERON:0001009	Circulatory System	UBERON:0004535	cardiovascular system	CNhs11978	Endothelial Cells - Thoracic, donor2
UBERON:0001009	Circulatory System	UBERON:0004535	cardiovascular system	CNhs11377	Endothelial Cells - Vein, donor2
UBERON:0001009	Circulatory System	UBERON:0004535	cardiovascular system	CNhs11378	Fibroblast - Cardiac, donor2
UBERON:0001009	Circulatory System	UBERON:0004535	cardiovascular system	CNhs11305	Smooth Muscle Cells - Aortic, donor2
UBERON:0001009	Circulatory System	UBERON:0004535	cardiovascular system	CNhs11985	Smooth Muscle Cells - Brachiocephalic, donor2
UBERON:0001009	Circulatory System	UBERON:0004535	cardiovascular system	CNhs11986	Smooth Muscle Cells - Carotid, donor2
UBERON:0001009	Circulatory System	UBERON:0004535	cardiovascular system	CNhs11987	Smooth Muscle Cells - Coronary Artery, donor2
UBERON:0001009	Circulatory System	UBERON:0004535	cardiovascular system	CNhs11988	Smooth Muscle Cells - Internal Thoracic Artery, donor2
UBERON:0001009	Circulatory System	UBERON:0004535	cardiovascular system	CNhs11989	Smooth Muscle Cells - Pulmonary Artery, donor2
UBERON:0001009	Circulatory System	UBERON:0004535	cardiovascular system	CNhs11990	Smooth Muscle Cells - Subclavian Artery, donor2
UBERON:0001009	Circulatory System	UBERON:0004535	cardiovascular system	CNhs11991	Smooth Muscle Cells - Umbilical Artery, donor2
UBERON:0001009	Circulatory System	UBERON:0004535	cardiovascular system	CNhs12002	Peripheral Blood Mononuclear Cells, donor3
UBERON:0001009	Circulatory System	UBERON:0004535	cardiovascular system	CNhs12004	Smooth Muscle Cells - Brain Vascular, donor3
UBERON:0001009	Circulatory System	UBERON:0004535	cardiovascular system	CNhs11906	Endothelial Cells - Lymphatic, donor3
UBERON:0001009	Circulatory System	UBERON:0004535	cardiovascular system	CNhs12009	Ciliary Epithelial Cells, donor3
UBERON:0001009	Circulatory System	UBERON:0004535	cardiovascular system	CNhs12010	Endothelial Cells - Umbilical vein, donor3
UBERON:0001009	Circulatory System	UBERON:0004535	cardiovascular system	CNhs12011	Fibroblast - Aortic Adventitial, donor3
UBERON:0001009	Circulatory System	UBERON:0004535	cardiovascular system	CNhs12022	Endothelial Cells - Aortic, donor3
UBERON:0001009	Circulatory System	UBERON:0004535	cardiovascular system	CNhs12023	Endothelial Cells - Artery, donor3
UBERON:0001009	Circulatory System	UBERON:0004535	cardiovascular system	CNhs12024	Endothelial Cells - Microvascular, donor3
UBERON:0001009	Circulatory System	UBERON:0004535	cardiovascular system	CNhs12026	Endothelial Cells - Vein, donor3
UBERON:0001009	Circulatory System	UBERON:0004535	cardiovascular system	CNhs12027	Fibroblast - Cardiac, donor3
UBERON:0001009	Circulatory System	UBERON:0004535	cardiovascular system	CNhs11309	Smooth Muscle Cells - Aortic, donor3
UBERON:0001009	Circulatory System	UBERON:0004535	cardiovascular system	CNhs12043	Smooth Muscle Cells - Brachiocephalic, donor3
UBERON:0001009	Circulatory System	UBERON:0004535	cardiovascular system	CNhs12044	Smooth Muscle Cells - Carotid, donor3
UBERON:0001009	Circulatory System	UBERON:0004535	cardiovascular system	CNhs12045	Smooth Muscle Cells - Coronary Artery, donor3
UBERON:0001009	Circulatory System	UBERON:0004535	cardiovascular system	CNhs12046	Smooth Muscle Cells - Internal Thoracic Artery, donor3
UBERON:0001009	Circulatory System	UBERON:0004535	cardiovascular system	CNhs12047	Smooth Muscle Cells - Pulmonary Artery, donor3
UBERON:0001009	Circulatory System	UBERON:0004535	cardiovascular system	CNhs12048	Smooth Muscle Cells - Subclavian Artery, donor3
UBERON:0001009	Circulatory System	UBERON:0004535	cardiovascular system	CNhs12049	Smooth Muscle Cells - Umbilical Artery, donor3
UBERON:0001009	Circulatory System	UBERON:0004535	cardiovascular system	CNhs11909	Fibroblast - Cardiac, donor4
UBERON:0001009	Circulatory System	UBERON:0004535	cardiovascular system	CNhs12057	Fibroblast - Cardiac, donor5
UBERON:0001009	Circulatory System	UBERON:0004535	cardiovascular system	CNhs12061	Fibroblast - Cardiac, donor6
UBERON:0001009	Circulatory System	UBERON:0004535	cardiovascular system	CNhs11319	Fibroblast - Choroid Plexus, donor1
UBERON:0001009	Circulatory System	UBERON:0004535	cardiovascular system	CNhs11322	Fibroblast - Lymphatic, donor1
UBERON:0001009	Circulatory System	UBERON:0004535	cardiovascular system	CNhs12075	Hepatic Sinusoidal Endothelial Cells, donor1
UBERON:0001009	Circulatory System	UBERON:0004535	cardiovascular system	CNhs12341	Cardiac Myocyte, donor1
UBERON:0001009	Circulatory System	UBERON:0004535	cardiovascular system	CNhs12596	Iris Pigment Epithelial Cells, donor1
UBERON:0001009	Circulatory System	UBERON:0004535	cardiovascular system	CNhs11340	Trabecular Meshwork Cells, donor1
UBERON:0001009	Circulatory System	UBERON:0004535	cardiovascular system	CNhs12597	Smooth Muscle Cells - Umbilical Vein, donor1
UBERON:0001009	Circulatory System	UBERON:0004535	cardiovascular system	CNhs12344	Fibroblast - Choroid Plexus, donor2
UBERON:0001009	Circulatory System	UBERON:0004535	cardiovascular system	CNhs12082	Fibroblast - Lymphatic, donor2
UBERON:0001009	Circulatory System	UBERON:0004535	cardiovascular system	CNhs12092	Hepatic Sinusoidal Endothelial Cells, donor2
UBERON:0001009	Circulatory System	UBERON:0004535	cardiovascular system	CNhs12350	Cardiac Myocyte, donor2
UBERON:0001009	Circulatory System	UBERON:0004535	cardiovascular system	CNhs12097	Trabecular Meshwork Cells, donor2
UBERON:0001009	Circulatory System	UBERON:0004535	cardiovascular system	CNhs12569	Smooth Muscle Cells - Umbilical Vein, donor2
UBERON:0001009	Circulatory System	UBERON:0004535	cardiovascular system	CNhs12620	Fibroblast - Choroid Plexus, donor3
UBERON:0001009	Circulatory System	UBERON:0004535	cardiovascular system	CNhs12118	Fibroblast - Lymphatic, donor3
UBERON:0001009	Circulatory System	UBERON:0004535	cardiovascular system	CNhs12625	Hepatic Sinusoidal Endothelial Cells, donor3
UBERON:0001009	Circulatory System	UBERON:0004535	cardiovascular system	CNhs12571	Cardiac Myocyte, donor3
UBERON:0001009	Circulatory System	UBERON:0004535	cardiovascular system	CNhs12124	Trabecular Meshwork Cells, donor3
UBERON:0001009	Circulatory System	UBERON:0004535	cardiovascular system	CNhs13076	Smooth Muscle Cells - Umbilical Vein, donor3
UBERON:0001009	Circulatory System	UBERON:0004535	cardiovascular system	CNhs12368	mesenchymal precursor cell - cardiac, donor1
UBERON:0001009	Circulatory System	UBERON:0004535	cardiovascular system	CNhs12369	mesenchymal precursor cell - cardiac, donor2
UBERON:0001009	Circulatory System	UBERON:0004535	cardiovascular system	CNhs12370	mesenchymal precursor cell - cardiac, donor3
UBERON:0001009	Circulatory System	UBERON:0004535	cardiovascular system	CNhs12371	mesenchymal precursor cell - cardiac, donor4
UBERON:0001009	Circulatory System	UBERON:0004535	cardiovascular system	CNhs11075	Whole blood (ribopure), donor090325, donation1
UBERON:0001009	Circulatory System	UBERON:0004535	cardiovascular system	CNhs11076	Whole blood (ribopure), donor090325, donation2
UBERON:0001009	Circulatory System	UBERON:0004535	cardiovascular system	CNhs11675	Whole blood (ribopure), donor090309, donation1
UBERON:0001009	Circulatory System	UBERON:0004535	cardiovascular system	CNhs11671	Whole blood (ribopure), donor090309, donation2
UBERON:0001009	Circulatory System	UBERON:0004535	cardiovascular system	CNhs11948	Whole blood (ribopure), donor090309, donation3
UBERON:0001009	Circulatory System	UBERON:0004535	cardiovascular system	CNhs11672	Whole blood (ribopure), donor090612, donation1
UBERON:0001009	Circulatory System	UBERON:0004535	cardiovascular system	CNhs11673	Whole blood (ribopure), donor090612, donation2
UBERON:0001009	Circulatory System	UBERON:0004535	cardiovascular system	CNhs11949	Whole blood (ribopure), donor090612, donation3
UBERON:0001009	Circulatory System	UBERON:0004536	lymph vasculature	CNhs10865	Endothelial Cells - Lymphatic, donor1
UBERON:0001009	Circulatory System	UBERON:0004536	lymph vasculature	CNhs11901	Endothelial Cells - Lymphatic, donor2
UBERON:0001009	Circulatory System	UBERON:0004536	lymph vasculature	CNhs11906	Endothelial Cells - Lymphatic, donor3
UBERON:0001009	Circulatory System	UBERON:0004536	lymph vasculature	CNhs11322	Fibroblast - Lymphatic, donor1
UBERON:0001009	Circulatory System	UBERON:0004536	lymph vasculature	CNhs12082	Fibroblast - Lymphatic, donor2
UBERON:0001009	Circulatory System	UBERON:0004536	lymph vasculature	CNhs12118	Fibroblast - Lymphatic, donor3
UBERON:0001009	Circulatory System	UBERON:0004537	blood vasculature	CNhs11760	aorta, adult, pool1
UBERON:0001009	Circulatory System	UBERON:0004537	blood vasculature	CNhs12843	artery, adult
UBERON:0001009	Circulatory System	UBERON:0004537	blood vasculature	CNhs10837	Endothelial Cells - Aortic, donor0
UBERON:0001009	Circulatory System	UBERON:0004537	blood vasculature	CNhs10838	Smooth Muscle Cells - Aortic, donor0
UBERON:0001009	Circulatory System	UBERON:0004537	blood vasculature	CNhs10839	Smooth Muscle Cells - Umbilical artery, donor0
UBERON:0001009	Circulatory System	UBERON:0004537	blood vasculature	CNhs10872	Endothelial Cells - Umbilical vein, donor1
UBERON:0001009	Circulatory System	UBERON:0004537	blood vasculature	CNhs10874	Fibroblast - Aortic Adventitial, donor1
UBERON:0001009	Circulatory System	UBERON:0004537	blood vasculature	CNhs10878	Fibroblast - Pulmonary Artery, donor1
UBERON:0001009	Circulatory System	UBERON:0004537	blood vasculature	CNhs12495	Endothelial Cells - Aortic, donor1
UBERON:0001009	Circulatory System	UBERON:0004537	blood vasculature	CNhs12496	Endothelial Cells - Artery, donor1
UBERON:0001009	Circulatory System	UBERON:0004537	blood vasculature	CNhs11925	Endothelial Cells - Microvascular, donor1
UBERON:0001009	Circulatory System	UBERON:0004537	blood vasculature	CNhs11926	Endothelial Cells - Thoracic, donor1
UBERON:0001009	Circulatory System	UBERON:0004537	blood vasculature	CNhs12497	Endothelial Cells - Vein, donor1
UBERON:0001009	Circulatory System	UBERON:0004537	blood vasculature	CNhs11085	Smooth Muscle Cells - Aortic, donor1
UBERON:0001009	Circulatory System	UBERON:0004537	blood vasculature	CNhs11086	Smooth Muscle Cells - Brachiocephalic, donor1
UBERON:0001009	Circulatory System	UBERON:0004537	blood vasculature	CNhs11087	Smooth Muscle Cells - Carotid, donor1
UBERON:0001009	Circulatory System	UBERON:0004537	blood vasculature	CNhs11088	Smooth Muscle Cells - Coronary Artery, donor1
UBERON:0001009	Circulatory System	UBERON:0004537	blood vasculature	CNhs11067	Smooth Muscle Cells - Internal Thoracic Artery, donor1
UBERON:0001009	Circulatory System	UBERON:0004537	blood vasculature	CNhs11089	Smooth Muscle Cells - Pulmonary Artery, donor1
UBERON:0001009	Circulatory System	UBERON:0004537	blood vasculature	CNhs11090	Smooth Muscle Cells - Subclavian Artery, donor1
UBERON:0001009	Circulatory System	UBERON:0004537	blood vasculature	CNhs11091	Smooth Muscle Cells - Umbilical Artery, donor1
UBERON:0001009	Circulatory System	UBERON:0004537	blood vasculature	CNhs11967	Endothelial Cells - Umbilical vein, donor2
UBERON:0001009	Circulatory System	UBERON:0004537	blood vasculature	CNhs11968	Fibroblast - Aortic Adventitial, donor2

UBERON:0001009	Circulatory System	UBERON:0004537	blood vasculature	CNhs11375	Endothelial Cells - Aortic, donor2
UBERON:0001009	Circulatory System	UBERON:0004537	blood vasculature	CNhs11977	Endothelial Cells - Artery, donor2
UBERON:0001009	Circulatory System	UBERON:0004537	blood vasculature	CNhs11376	Endothelial Cells - Microvascular, donor2
UBERON:0001009	Circulatory System	UBERON:0004537	blood vasculature	CNhs11978	Endothelial Cells - Thoracic, donor2
UBERON:0001009	Circulatory System	UBERON:0004537	blood vasculature	CNhs11377	Endothelial Cells - Vein, donor2
UBERON:0001009	Circulatory System	UBERON:0004537	blood vasculature	CNhs11305	Smooth Muscle Cells - Aortic, donor2
UBERON:0001009	Circulatory System	UBERON:0004537	blood vasculature	CNhs11985	Smooth Muscle Cells - Brachiocephalic, donor2
UBERON:0001009	Circulatory System	UBERON:0004537	blood vasculature	CNhs11986	Smooth Muscle Cells - Carotid, donor2
UBERON:0001009	Circulatory System	UBERON:0004537	blood vasculature	CNhs11987	Smooth Muscle Cells - Coronary Artery, donor2
UBERON:0001009	Circulatory System	UBERON:0004537	blood vasculature	CNhs11988	Smooth Muscle Cells - Internal Thoracic Artery, donor2
UBERON:0001009	Circulatory System	UBERON:0004537	blood vasculature	CNhs11989	Smooth Muscle Cells - Pulmonary Artery, donor2
UBERON:0001009	Circulatory System	UBERON:0004537	blood vasculature	CNhs11990	Smooth Muscle Cells - Subclavian Artery, donor2
UBERON:0001009	Circulatory System	UBERON:0004537	blood vasculature	CNhs11991	Smooth Muscle Cells - Umbilical Artery, donor2
UBERON:0001009	Circulatory System	UBERON:0004537	blood vasculature	CNhs12010	Endothelial Cells - Umbilical vein, donor3
UBERON:0001009	Circulatory System	UBERON:0004537	blood vasculature	CNhs12011	Fibroblast - Aortic Adventitial, donor3
UBERON:0001009	Circulatory System	UBERON:0004537	blood vasculature	CNhs12022	Endothelial Cells - Aortic, donor3
UBERON:0001009	Circulatory System	UBERON:0004537	blood vasculature	CNhs12023	Endothelial Cells - Artery, donor3
UBERON:0001009	Circulatory System	UBERON:0004537	blood vasculature	CNhs12024	Endothelial Cells - Microvascular, donor3
UBERON:0001009	Circulatory System	UBERON:0004537	blood vasculature	CNhs12026	Endothelial Cells - Vein, donor3
UBERON:0001009	Circulatory System	UBERON:0004537	blood vasculature	CNhs11309	Smooth Muscle Cells - Aortic, donor3
UBERON:0001009	Circulatory System	UBERON:0004537	blood vasculature	CNhs12043	Smooth Muscle Cells - Brachiocephalic, donor3
UBERON:0001009	Circulatory System	UBERON:0004537	blood vasculature	CNhs12044	Smooth Muscle Cells - Carotid, donor3
UBERON:0001009	Circulatory System	UBERON:0004537	blood vasculature	CNhs12045	Smooth Muscle Cells - Coronary Artery, donor3
UBERON:0001009	Circulatory System	UBERON:0004537	blood vasculature	CNhs12046	Smooth Muscle Cells - Internal Thoracic Artery, donor3
UBERON:0001009	Circulatory System	UBERON:0004537	blood vasculature	CNhs12047	Smooth Muscle Cells - Pulmonary Artery, donor3
UBERON:0001009	Circulatory System	UBERON:0004537	blood vasculature	CNhs12048	Smooth Muscle Cells - Subclavian Artery, donor3
UBERON:0001009	Circulatory System	UBERON:0004537	blood vasculature	CNhs12049	Smooth Muscle Cells - Umbilical Artery, donor3
UBERON:0001009	Circulatory System	UBERON:0004537	blood vasculature	CNhs12075	Hepatic Sinusoidal Endothelial Cells, donor1
UBERON:0001009	Circulatory System	UBERON:0004537	blood vasculature	CNhs12597	Smooth Muscle Cells - Umbilical Vein, donor1
UBERON:0001009	Circulatory System	UBERON:0004537	blood vasculature	CNhs12092	Hepatic Sinusoidal Endothelial Cells, donor2
UBERON:0001009	Circulatory System	UBERON:0004537	blood vasculature	CNhs12569	Smooth Muscle Cells - Umbilical Vein, donor2
UBERON:0001009	Circulatory System	UBERON:0004537	blood vasculature	CNhs12625	Hepatic Sinusoidal Endothelial Cells, donor3
UBERON:0001009	Circulatory System	UBERON:0004537	blood vasculature	CNhs13076	Smooth Muscle Cells - Umbilical Vein, donor3
UBERON:0001009	Circulatory System	UBERON:0004571	systemic arterial system	CNhs11760	aorta, adult, pool1
UBERON:0001009	Circulatory System	UBERON:0004571	systemic arterial system	CNhs10837	Endothelial Cells - Aortic, donor0
UBERON:0001009	Circulatory System	UBERON:0004571	systemic arterial system	CNhs10838	Smooth Muscle Cells - Aortic, donor0
UBERON:0001009	Circulatory System	UBERON:0004571	systemic arterial system	CNhs10874	Fibroblast - Aortic Adventitial, donor1
UBERON:0001009	Circulatory System	UBERON:0004571	systemic arterial system	CNhs12495	Endothelial Cells - Aortic, donor1
UBERON:0001009	Circulatory System	UBERON:0004571	systemic arterial system	CNhs11926	Endothelial Cells - Thoracic, donor1
UBERON:0001009	Circulatory System	UBERON:0004571	systemic arterial system	CNhs11085	Smooth Muscle Cells - Aortic, donor1
UBERON:0001009	Circulatory System	UBERON:0004571	systemic arterial system	CNhs11086	Smooth Muscle Cells - Brachiocephalic, donor1
UBERON:0001009	Circulatory System	UBERON:0004571	systemic arterial system	CNhs11087	Smooth Muscle Cells - Carotid, donor1
UBERON:0001009	Circulatory System	UBERON:0004571	systemic arterial system	CNhs11088	Smooth Muscle Cells - Coronary Artery, donor1
UBERON:0001009	Circulatory System	UBERON:0004571	systemic arterial system	CNhs11067	Smooth Muscle Cells - Internal Thoracic Artery, donor1
UBERON:0001009	Circulatory System	UBERON:0004571	systemic arterial system	CNhs11090	Smooth Muscle Cells - Subclavian Artery, donor1
UBERON:0001009	Circulatory System	UBERON:0004571	systemic arterial system	CNhs11968	Fibroblast - Aortic Adventitial, donor2
UBERON:0001009	Circulatory System	UBERON:0004571	systemic arterial system	CNhs11375	Endothelial Cells - Aortic, donor2
UBERON:0001009	Circulatory System	UBERON:0004571	systemic arterial system	CNhs11978	Endothelial Cells - Thoracic, donor2
UBERON:0001009	Circulatory System	UBERON:0004571	systemic arterial system	CNhs11305	Smooth Muscle Cells - Aortic, donor2
UBERON:0001009	Circulatory System	UBERON:0004571	systemic arterial system	CNhs11985	Smooth Muscle Cells - Brachiocephalic, donor2
UBERON:0001009	Circulatory System	UBERON:0004571	systemic arterial system	CNhs11986	Smooth Muscle Cells - Carotid, donor2
UBERON:0001009	Circulatory System	UBERON:0004571	systemic arterial system	CNhs11987	Smooth Muscle Cells - Coronary Artery, donor2
UBERON:0001009	Circulatory System	UBERON:0004571	systemic arterial system	CNhs11988	Smooth Muscle Cells - Internal Thoracic Artery, donor2
UBERON:0001009	Circulatory System	UBERON:0004571	systemic arterial system	CNhs11990	Smooth Muscle Cells - Subclavian Artery, donor2
UBERON:0001009	Circulatory System	UBERON:0004571	systemic arterial system	CNhs12011	Fibroblast - Aortic Adventitial, donor3
UBERON:0001009	Circulatory System	UBERON:0004571	systemic arterial system	CNhs12022	Endothelial Cells - Aortic, donor3
UBERON:0001009	Circulatory System	UBERON:0004571	systemic arterial system	CNhs11309	Smooth Muscle Cells - Aortic, donor3
UBERON:0001009	Circulatory System	UBERON:0004571	systemic arterial system	CNhs12043	Smooth Muscle Cells - Brachiocephalic, donor3
UBERON:0001009	Circulatory System	UBERON:0004571	systemic arterial system	CNhs12044	Smooth Muscle Cells - Carotid, donor3
UBERON:0001009	Circulatory System	UBERON:0004571	systemic arterial system	CNhs12045	Smooth Muscle Cells - Coronary Artery, donor3
UBERON:0001009	Circulatory System	UBERON:0004571	systemic arterial system	CNhs12046	Smooth Muscle Cells - Internal Thoracic Artery, donor3
UBERON:0001009	Circulatory System	UBERON:0004571	systemic arterial system	CNhs12048	Smooth Muscle Cells - Subclavian Artery, donor3
UBERON:0001009	Circulatory System	UBERON:0004572	arterial system	CNhs11760	aorta, adult, pool1
UBERON:0001009	Circulatory System	UBERON:0004572	arterial system	CNhs12843	artery, adult
UBERON:0001009	Circulatory System	UBERON:0004572	arterial system	CNhs10837	Endothelial Cells - Aortic, donor0
UBERON:0001009	Circulatory System	UBERON:0004572	arterial system	CNhs10838	Smooth Muscle Cells - Aortic, donor0
UBERON:0001009	Circulatory System	UBERON:0004572	arterial system	CNhs10839	Smooth Muscle Cells - Umbilical artery, donor0
UBERON:0001009	Circulatory System	UBERON:0004572	arterial system	CNhs10874	Fibroblast - Aortic Adventitial, donor1
UBERON:0001009	Circulatory System	UBERON:0004572	arterial system	CNhs10878	Fibroblast - Pulmonary Artery, donor1
UBERON:0001009	Circulatory System	UBERON:0004572	arterial system	CNhs12495	Endothelial Cells - Aortic, donor1
UBERON:0001009	Circulatory System	UBERON:0004572	arterial system	CNhs12496	Endothelial Cells - Artery, donor1
UBERON:0001009	Circulatory System	UBERON:0004572	arterial system	CNhs11926	Endothelial Cells - Thoracic, donor1
UBERON:0001009	Circulatory System	UBERON:0004572	arterial system	CNhs11085	Smooth Muscle Cells - Aortic, donor1
UBERON:0001009	Circulatory System	UBERON:0004572	arterial system	CNhs11086	Smooth Muscle Cells - Brachiocephalic, donor1
UBERON:0001009	Circulatory System	UBERON:0004572	arterial system	CNhs11087	Smooth Muscle Cells - Carotid, donor1
UBERON:0001009	Circulatory System	UBERON:0004572	arterial system	CNhs11088	Smooth Muscle Cells - Coronary Artery, donor1
UBERON:0001009	Circulatory System	UBERON:0004572	arterial system	CNhs11067	Smooth Muscle Cells - Internal Thoracic Artery, donor1
UBERON:0001009	Circulatory System	UBERON:0004572	arterial system	CNhs11089	Smooth Muscle Cells - Pulmonary Artery, donor1
UBERON:0001009	Circulatory System	UBERON:0004572	arterial system	CNhs11090	Smooth Muscle Cells - Subclavian Artery, donor1
UBERON:0001009	Circulatory System	UBERON:0004572	arterial system	CNhs11091	Smooth Muscle Cells - Umbilical Artery, donor1
UBERON:0001009	Circulatory System	UBERON:0004572	arterial system	CNhs11968	Fibroblast - Aortic Adventitial, donor2
UBERON:0001009	Circulatory System	UBERON:0004572	arterial system	CNhs11375	Endothelial Cells - Aortic, donor2
UBERON:0001009	Circulatory System	UBERON:0004572	arterial system	CNhs11977	Endothelial Cells - Artery, donor2
UBERON:0001009	Circulatory System	UBERON:0004572	arterial system	CNhs11978	Endothelial Cells - Thoracic, donor2
UBERON:0001009	Circulatory System	UBERON:0004572	arterial system	CNhs11305	Smooth Muscle Cells - Aortic, donor2
UBERON:0001009	Circulatory System	UBERON:0004572	arterial system	CNhs11985	Smooth Muscle Cells - Brachiocephalic, donor2
UBERON:0001009	Circulatory System	UBERON:0004572	arterial system	CNhs11986	Smooth Muscle Cells - Carotid, donor2
UBERON:0001009	Circulatory System	UBERON:0004572	arterial system	CNhs11987	Smooth Muscle Cells - Coronary Artery, donor2
UBERON:0001009	Circulatory System	UBERON:0004572	arterial system	CNhs11988	Smooth Muscle Cells - Internal Thoracic Artery, donor2
UBERON:0001009	Circulatory System	UBERON:0004572	arterial system	CNhs11989	Smooth Muscle Cells - Pulmonary Artery, donor2
UBERON:0001009	Circulatory System	UBERON:0004572	arterial system	CNhs11990	Smooth Muscle Cells - Subclavian Artery, donor2
UBERON:0001009	Circulatory System	UBERON:0004572	arterial system	CNhs11991	Smooth Muscle Cells - Umbilical Artery, donor2
UBERON:0001009	Circulatory System	UBERON:0004572	arterial system	CNhs12011	Fibroblast - Aortic Adventitial, donor3
UBERON:0001009	Circulatory System	UBERON:0004572	arterial system	CNhs12022	Endothelial Cells - Aortic, donor3
UBERON:0001009	Circulatory System	UBERON:0004572	arterial system	CNhs12023	Endothelial Cells - Artery, donor3
UBERON:0001009	Circulatory System	UBERON:0004572	arterial system	CNhs11309	Smooth Muscle Cells - Aortic, donor3
UBERON:0001009	Circulatory System	UBERON:0004572	arterial system	CNhs12043	Smooth Muscle Cells - Brachiocephalic, donor3
UBERON:0001009	Circulatory System	UBERON:0004572	arterial system	CNhs12044	Smooth Muscle Cells - Carotid, donor3

UBERON:0001009	Circulatory System	UBERON:0004572	arterial system	CNhs12045	Smooth Muscle Cells - Coronary Artery, donor3
UBERON:0001009	Circulatory System	UBERON:0004572	arterial system	CNhs12046	Smooth Muscle Cells - Internal Thoracic Artery, donor3
UBERON:0001009	Circulatory System	UBERON:0004572	arterial system	CNhs12047	Smooth Muscle Cells - Pulmonary Artery, donor3
UBERON:0001009	Circulatory System	UBERON:0004572	arterial system	CNhs12048	Smooth Muscle Cells - Subclavian Artery, donor3
UBERON:0001009	Circulatory System	UBERON:0004572	arterial system	CNhs12049	Smooth Muscle Cells - Umbilical Artery, donor3
UBERON:0001009	Circulatory System	UBERON:0004573	systemic artery	CNhs11760	aorta, adult, pool1
UBERON:0001009	Circulatory System	UBERON:0004573	systemic artery	CNhs10837	Endothelial Cells - Aortic, donor0
UBERON:0001009	Circulatory System	UBERON:0004573	systemic artery	CNhs10838	Smooth Muscle Cells - Aortic, donor0
UBERON:0001009	Circulatory System	UBERON:0004573	systemic artery	CNhs10874	Fibroblast - Aortic Adventitial, donor1
UBERON:0001009	Circulatory System	UBERON:0004573	systemic artery	CNhs12495	Endothelial Cells - Aortic, donor1
UBERON:0001009	Circulatory System	UBERON:0004573	systemic artery	CNhs11926	Endothelial Cells - Thoracic, donor1
UBERON:0001009	Circulatory System	UBERON:0004573	systemic artery	CNhs11085	Smooth Muscle Cells - Aortic, donor1
UBERON:0001009	Circulatory System	UBERON:0004573	systemic artery	CNhs11086	Smooth Muscle Cells - Brachiocephalic, donor1
UBERON:0001009	Circulatory System	UBERON:0004573	systemic artery	CNhs11087	Smooth Muscle Cells - Carotid, donor1
UBERON:0001009	Circulatory System	UBERON:0004573	systemic artery	CNhs11088	Smooth Muscle Cells - Coronary Artery, donor1
UBERON:0001009	Circulatory System	UBERON:0004573	systemic artery	CNhs11067	Smooth Muscle Cells - Internal Thoracic Artery, donor1
UBERON:0001009	Circulatory System	UBERON:0004573	systemic artery	CNhs11090	Smooth Muscle Cells - Subclavian Artery, donor1
UBERON:0001009	Circulatory System	UBERON:0004573	systemic artery	CNhs11968	Fibroblast - Aortic Adventitial, donor2
UBERON:0001009	Circulatory System	UBERON:0004573	systemic artery	CNhs11375	Endothelial Cells - Aortic, donor2
UBERON:0001009	Circulatory System	UBERON:0004573	systemic artery	CNhs11978	Endothelial Cells - Thoracic, donor2
UBERON:0001009	Circulatory System	UBERON:0004573	systemic artery	CNhs11305	Smooth Muscle Cells - Aortic, donor2
UBERON:0001009	Circulatory System	UBERON:0004573	systemic artery	CNhs11985	Smooth Muscle Cells - Brachiocephalic, donor2
UBERON:0001009	Circulatory System	UBERON:0004573	systemic artery	CNhs11986	Smooth Muscle Cells - Carotid, donor2
UBERON:0001009	Circulatory System	UBERON:0004573	systemic artery	CNhs11987	Smooth Muscle Cells - Coronary Artery, donor2
UBERON:0001009	Circulatory System	UBERON:0004573	systemic artery	CNhs11988	Smooth Muscle Cells - Internal Thoracic Artery, donor2
UBERON:0001009	Circulatory System	UBERON:0004573	systemic artery	CNhs11990	Smooth Muscle Cells - Subclavian Artery, donor2
UBERON:0001009	Circulatory System	UBERON:0004573	systemic artery	CNhs12011	Fibroblast - Aortic Adventitial, donor3
UBERON:0001009	Circulatory System	UBERON:0004573	systemic artery	CNhs12022	Endothelial Cells - Aortic, donor3
UBERON:0001009	Circulatory System	UBERON:0004573	systemic artery	CNhs11309	Smooth Muscle Cells - Aortic, donor3
UBERON:0001009	Circulatory System	UBERON:0004573	systemic artery	CNhs12043	Smooth Muscle Cells - Brachiocephalic, donor3
UBERON:0001009	Circulatory System	UBERON:0004573	systemic artery	CNhs12044	Smooth Muscle Cells - Carotid, donor3
UBERON:0001009	Circulatory System	UBERON:0004573	systemic artery	CNhs12045	Smooth Muscle Cells - Coronary Artery, donor3
UBERON:0001009	Circulatory System	UBERON:0004573	systemic artery	CNhs12046	Smooth Muscle Cells - Internal Thoracic Artery, donor3
UBERON:0001009	Circulatory System	UBERON:0004573	systemic artery	CNhs12048	Smooth Muscle Cells - Subclavian Artery, donor3
UBERON:0001009	Circulatory System	UBERON:0004582	venous system	CNhs10872	Endothelial Cells - Umbilical vein, donor1
UBERON:0001009	Circulatory System	UBERON:0004582	venous system	CNhs12497	Endothelial Cells - Vein, donor1
UBERON:0001009	Circulatory System	UBERON:0004582	venous system	CNhs11967	Endothelial Cells - Umbilical vein, donor2
UBERON:0001009	Circulatory System	UBERON:0004582	venous system	CNhs11377	Endothelial Cells - Vein, donor2
UBERON:0001009	Circulatory System	UBERON:0004582	venous system	CNhs12010	Endothelial Cells - Umbilical vein, donor3
UBERON:0001009	Circulatory System	UBERON:0004582	venous system	CNhs12026	Endothelial Cells - Vein, donor3
UBERON:0001009	Circulatory System	UBERON:0004582	venous system	CNhs12597	Smooth Muscle Cells - Umbilical Vein, donor1
UBERON:0001009	Circulatory System	UBERON:0004582	venous system	CNhs12569	Smooth Muscle Cells - Umbilical Vein, donor2
UBERON:0001009	Circulatory System	UBERON:0004582	venous system	CNhs13076	Smooth Muscle Cells - Umbilical Vein, donor3
UBERON:0001009	Circulatory System	UBERON:0004638	blood vessel endothelium	CNhs10837	Endothelial Cells - Aortic, donor0
UBERON:0001009	Circulatory System	UBERON:0004638	blood vessel endothelium	CNhs10872	Endothelial Cells - Umbilical vein, donor1
UBERON:0001009	Circulatory System	UBERON:0004638	blood vessel endothelium	CNhs12495	Endothelial Cells - Aortic, donor1
UBERON:0001009	Circulatory System	UBERON:0004638	blood vessel endothelium	CNhs12496	Endothelial Cells - Artery, donor1
UBERON:0001009	Circulatory System	UBERON:0004638	blood vessel endothelium	CNhs11925	Endothelial Cells - Microvascular, donor1
UBERON:0001009	Circulatory System	UBERON:0004638	blood vessel endothelium	CNhs11926	Endothelial Cells - Thoracic, donor1
UBERON:0001009	Circulatory System	UBERON:0004638	blood vessel endothelium	CNhs12497	Endothelial Cells - Vein, donor1
UBERON:0001009	Circulatory System	UBERON:0004638	blood vessel endothelium	CNhs11967	Endothelial Cells - Umbilical vein, donor2
UBERON:0001009	Circulatory System	UBERON:0004638	blood vessel endothelium	CNhs11375	Endothelial Cells - Aortic, donor2
UBERON:0001009	Circulatory System	UBERON:0004638	blood vessel endothelium	CNhs11977	Endothelial Cells - Artery, donor2
UBERON:0001009	Circulatory System	UBERON:0004638	blood vessel endothelium	CNhs11376	Endothelial Cells - Microvascular, donor2
UBERON:0001009	Circulatory System	UBERON:0004638	blood vessel endothelium	CNhs11978	Endothelial Cells - Thoracic, donor2
UBERON:0001009	Circulatory System	UBERON:0004638	blood vessel endothelium	CNhs11377	Endothelial Cells - Vein, donor2
UBERON:0001009	Circulatory System	UBERON:0004638	blood vessel endothelium	CNhs12010	Endothelial Cells - Umbilical vein, donor3
UBERON:0001009	Circulatory System	UBERON:0004638	blood vessel endothelium	CNhs12022	Endothelial Cells - Aortic, donor3
UBERON:0001009	Circulatory System	UBERON:0004638	blood vessel endothelium	CNhs12023	Endothelial Cells - Artery, donor3
UBERON:0001009	Circulatory System	UBERON:0004638	blood vessel endothelium	CNhs12024	Endothelial Cells - Microvascular, donor3
UBERON:0001009	Circulatory System	UBERON:0004638	blood vessel endothelium	CNhs12026	Endothelial Cells - Vein, donor3
UBERON:0001009	Circulatory System	UBERON:0004663	aorta wall	CNhs10874	Fibroblast - Aortic Adventitial, donor1
UBERON:0001009	Circulatory System	UBERON:0004663	aorta wall	CNhs11968	Fibroblast - Aortic Adventitial, donor2
UBERON:0001009	Circulatory System	UBERON:0004663	aorta wall	CNhs12011	Fibroblast - Aortic Adventitial, donor3
UBERON:0001009	Circulatory System	UBERON:0004664	aorta tunica adventitia	CNhs10874	Fibroblast - Aortic Adventitial, donor1
UBERON:0001009	Circulatory System	UBERON:0004664	aorta tunica adventitia	CNhs11968	Fibroblast - Aortic Adventitial, donor2
UBERON:0001009	Circulatory System	UBERON:0004664	aorta tunica adventitia	CNhs12011	Fibroblast - Aortic Adventitial, donor3
UBERON:0001009	Circulatory System	UBERON:0004695	arterial system smooth muscle	CNhs10838	Smooth Muscle Cells - Aortic, donor0
UBERON:0001009	Circulatory System	UBERON:0004695	arterial system smooth muscle	CNhs11085	Smooth Muscle Cells - Aortic, donor1
UBERON:0001009	Circulatory System	UBERON:0004695	arterial system smooth muscle	CNhs11305	Smooth Muscle Cells - Aortic, donor2
UBERON:0001009	Circulatory System	UBERON:0004695	arterial system smooth muscle	CNhs11309	Smooth Muscle Cells - Aortic, donor3
UBERON:0001009	Circulatory System	UBERON:0004700	arterial system endothelium	CNhs10837	Endothelial Cells - Aortic, donor0
UBERON:0001009	Circulatory System	UBERON:0004700	arterial system endothelium	CNhs12495	Endothelial Cells - Aortic, donor1
UBERON:0001009	Circulatory System	UBERON:0004700	arterial system endothelium	CNhs12496	Endothelial Cells - Artery, donor1
UBERON:0001009	Circulatory System	UBERON:0004700	arterial system endothelium	CNhs11926	Endothelial Cells - Thoracic, donor1
UBERON:0001009	Circulatory System	UBERON:0004700	arterial system endothelium	CNhs11375	Endothelial Cells - Aortic, donor2
UBERON:0001009	Circulatory System	UBERON:0004700	arterial system endothelium	CNhs11977	Endothelial Cells - Artery, donor2
UBERON:0001009	Circulatory System	UBERON:0004700	arterial system endothelium	CNhs11978	Endothelial Cells - Thoracic, donor2
UBERON:0001009	Circulatory System	UBERON:0004700	arterial system endothelium	CNhs12022	Endothelial Cells - Aortic, donor3
UBERON:0001009	Circulatory System	UBERON:0004700	arterial system endothelium	CNhs12023	Endothelial Cells - Artery, donor3
UBERON:0001009	Circulatory System	UBERON:0004797	blood vessel layer	CNhs10874	Fibroblast - Aortic Adventitial, donor1
UBERON:0001009	Circulatory System	UBERON:0004797	blood vessel layer	CNhs11968	Fibroblast - Aortic Adventitial, donor2
UBERON:0001009	Circulatory System	UBERON:0004797	blood vessel layer	CNhs12011	Fibroblast - Aortic Adventitial, donor3
UBERON:0001009	Circulatory System	UBERON:0004852	cardiovascular system endothelium	CNhs10837	Endothelial Cells - Aortic, donor0
UBERON:0001009	Circulatory System	UBERON:0004852	cardiovascular system endothelium	CNhs10872	Endothelial Cells - Umbilical vein, donor1
UBERON:0001009	Circulatory System	UBERON:0004852	cardiovascular system endothelium	CNhs12495	Endothelial Cells - Aortic, donor1
UBERON:0001009	Circulatory System	UBERON:0004852	cardiovascular system endothelium	CNhs12496	Endothelial Cells - Artery, donor1
UBERON:0001009	Circulatory System	UBERON:0004852	cardiovascular system endothelium	CNhs11925	Endothelial Cells - Microvascular, donor1
UBERON:0001009	Circulatory System	UBERON:0004852	cardiovascular system endothelium	CNhs11926	Endothelial Cells - Thoracic, donor1
UBERON:0001009	Circulatory System	UBERON:0004852	cardiovascular system endothelium	CNhs12497	Endothelial Cells - Vein, donor1
UBERON:0001009	Circulatory System	UBERON:0004852	cardiovascular system endothelium	CNhs11967	Endothelial Cells - Umbilical vein, donor2
UBERON:0001009	Circulatory System	UBERON:0004852	cardiovascular system endothelium	CNhs11375	Endothelial Cells - Aortic, donor2
UBERON:0001009	Circulatory System	UBERON:0004852	cardiovascular system endothelium	CNhs11977	Endothelial Cells - Artery, donor2
UBERON:0001009	Circulatory System	UBERON:0004852	cardiovascular system endothelium	CNhs11376	Endothelial Cells - Microvascular, donor2
UBERON:0001009	Circulatory System	UBERON:0004852	cardiovascular system endothelium	CNhs11978	Endothelial Cells - Thoracic, donor2
UBERON:0001009	Circulatory System	UBERON:0004852	cardiovascular system endothelium	CNhs11377	Endothelial Cells - Vein, donor2
UBERON:0001009	Circulatory System	UBERON:0004852	cardiovascular system endothelium	CNhs12010	Endothelial Cells - Umbilical vein, donor3
UBERON:0001009	Circulatory System	UBERON:0004852	cardiovascular system endothelium	CNhs12022	Endothelial Cells - Aortic, donor3

UBERON:0001009	Circulatory System	UBERON:0004852	cardiovascular system endothelium	CNhs12023	Endothelial Cells - Artery, donor3
UBERON:0001009	Circulatory System	UBERON:0004852	cardiovascular system endothelium	CNhs12024	Endothelial Cells - Microvascular, donor3
UBERON:0001009	Circulatory System	UBERON:0004852	cardiovascular system endothelium	CNhs12026	Endothelial Cells - Vein, donor3
UBERON:0001009	Circulatory System	UBERON:0005208	right atrium valve	CNhs12857	heart - tricuspid valve, adult
UBERON:0001009	Circulatory System	UBERON:0005284	brain vasculature	CNhs10863	Smooth Muscle Cells - Brain Vascular, donor1
UBERON:0001009	Circulatory System	UBERON:0005284	brain vasculature	CNhs11900	Smooth Muscle Cells - Brain Vascular, donor2
UBERON:0001009	Circulatory System	UBERON:0005284	brain vasculature	CNhs12004	Smooth Muscle Cells - Brain Vascular, donor3
UBERON:0001009	Circulatory System	UBERON:0005284	brain vasculature	CNhs11319	Fibroblast - Choroid Plexus, donor1
UBERON:0001009	Circulatory System	UBERON:0005284	brain vasculature	CNhs12344	Fibroblast - Choroid Plexus, donor2
UBERON:0001009	Circulatory System	UBERON:0005284	brain vasculature	CNhs12620	Fibroblast - Choroid Plexus, donor3
UBERON:0001009	Circulatory System	UBERON:0005396	carotid artery segment	CNhs11087	Smooth Muscle Cells - Carotid, donor1
UBERON:0001009	Circulatory System	UBERON:0005396	carotid artery segment	CNhs11986	Smooth Muscle Cells - Carotid, donor2
UBERON:0001009	Circulatory System	UBERON:0005396	carotid artery segment	CNhs12044	Smooth Muscle Cells - Carotid, donor3
UBERON:0001009	Circulatory System	UBERON:0005606	hyaloid cavity	CNhs13440	eye - vitreous humor, donor1
UBERON:0001009	Circulatory System	UBERON:0005623	semi-lunar valve	CNhs12856	heart - pulmonic valve, adult
UBERON:0001009	Circulatory System	UBERON:0005629	vascular plexus	CNhs11319	Fibroblast - Choroid Plexus, donor1
UBERON:0001009	Circulatory System	UBERON:0005629	vascular plexus	CNhs12344	Fibroblast - Choroid Plexus, donor2
UBERON:0001009	Circulatory System	UBERON:0005629	vascular plexus	CNhs12620	Fibroblast - Choroid Plexus, donor3
UBERON:0001009	Circulatory System	UBERON:0005734	tunica adventitia	CNhs10874	Fibroblast - Aortic Adventitial, donor1
UBERON:0001009	Circulatory System	UBERON:0005734	tunica adventitia	CNhs11968	Fibroblast - Aortic Adventitial, donor2
UBERON:0001009	Circulatory System	UBERON:0005734	tunica adventitia	CNhs12011	Fibroblast - Aortic Adventitial, donor3
UBERON:0001009	Circulatory System	UBERON:0005800	segment of aorta	CNhs11926	Endothelial Cells - Thoracic, donor1
UBERON:0001009	Circulatory System	UBERON:0005800	segment of aorta	CNhs11978	Endothelial Cells - Thoracic, donor2
UBERON:0001009	Circulatory System	UBERON:0005946	outflow tract of atrium	CNhs12857	heart - tricuspid valve, adult
UBERON:0001009	Circulatory System	UBERON:0005965	outflow part of right atrium	CNhs12857	heart - tricuspid valve, adult
UBERON:0001009	Circulatory System	UBERON:0005969	reticulum trabeculare	CNhs11340	Trabecular Meshwork Cells, donor1
UBERON:0001009	Circulatory System	UBERON:0005969	reticulum trabeculare	CNhs12097	Trabecular Meshwork Cells, donor2
UBERON:0001009	Circulatory System	UBERON:0005969	reticulum trabeculare	CNhs12124	Trabecular Meshwork Cells, donor3
UBERON:0001009	Circulatory System	UBERON:0005985	coronary vessel	CNhs11088	Smooth Muscle Cells - Coronary Artery, donor1
UBERON:0001009	Circulatory System	UBERON:0005985	coronary vessel	CNhs11987	Smooth Muscle Cells - Coronary Artery, donor2
UBERON:0001009	Circulatory System	UBERON:0005985	coronary vessel	CNhs12045	Smooth Muscle Cells - Coronary Artery, donor3
UBERON:0001009	Circulatory System	UBERON:0006558	lymphatic part of lymphoid system	CNhs10865	Endothelial Cells - Lymphatic, donor1
UBERON:0001009	Circulatory System	UBERON:0006558	lymphatic part of lymphoid system	CNhs11901	Endothelial Cells - Lymphatic, donor2
UBERON:0001009	Circulatory System	UBERON:0006558	lymphatic part of lymphoid system	CNhs11906	Endothelial Cells - Lymphatic, donor3
UBERON:0001009	Circulatory System	UBERON:0006558	lymphatic part of lymphoid system	CNhs11322	Fibroblast - Lymphatic, donor1
UBERON:0001009	Circulatory System	UBERON:0006558	lymphatic part of lymphoid system	CNhs12082	Fibroblast - Lymphatic, donor2
UBERON:0001009	Circulatory System	UBERON:0006558	lymphatic part of lymphoid system	CNhs12118	Fibroblast - Lymphatic, donor3
UBERON:0001009	Circulatory System	UBERON:0006876	vasculature of organ	CNhs13440	eye - vitreous humor, donor1
UBERON:0001009	Circulatory System	UBERON:0006876	vasculature of organ	CNhs10863	Smooth Muscle Cells - Brain Vascular, donor1
UBERON:0001009	Circulatory System	UBERON:0006876	vasculature of organ	CNhs10871	Ciliary Epithelial Cells, donor1
UBERON:0001009	Circulatory System	UBERON:0006876	vasculature of organ	CNhs11900	Smooth Muscle Cells - Brain Vascular, donor2
UBERON:0001009	Circulatory System	UBERON:0006876	vasculature of organ	CNhs11966	Ciliary Epithelial Cells, donor2
UBERON:0001009	Circulatory System	UBERON:0006876	vasculature of organ	CNhs12004	Smooth Muscle Cells - Brain Vascular, donor3
UBERON:0001009	Circulatory System	UBERON:0006876	vasculature of organ	CNhs12009	Ciliary Epithelial Cells, donor3
UBERON:0001009	Circulatory System	UBERON:0006876	vasculature of organ	CNhs11319	Fibroblast - Choroid Plexus, donor1
UBERON:0001009	Circulatory System	UBERON:0006876	vasculature of organ	CNhs12596	Iris Pigment Epithelial Cells, donor1
UBERON:0001009	Circulatory System	UBERON:0006876	vasculature of organ	CNhs11340	Trabecular Meshwork Cells, donor1
UBERON:0001009	Circulatory System	UBERON:0006876	vasculature of organ	CNhs12344	Fibroblast - Choroid Plexus, donor2
UBERON:0001009	Circulatory System	UBERON:0006876	vasculature of organ	CNhs12097	Trabecular Meshwork Cells, donor2
UBERON:0001009	Circulatory System	UBERON:0006876	vasculature of organ	CNhs12620	Fibroblast - Choroid Plexus, donor3
UBERON:0001009	Circulatory System	UBERON:0006876	vasculature of organ	CNhs12124	Trabecular Meshwork Cells, donor3
UBERON:0001009	Circulatory System	UBERON:0007100	circulatory organ	CNhs10621	heart, adult, pool1
UBERON:0001009	Circulatory System	UBERON:0007100	circulatory organ	CNhs10653	heart, fetal, pool1
UBERON:0001009	Circulatory System	UBERON:0007100	circulatory organ	CNhs11757	heart, adult, diseased post-infarction, donor1
UBERON:0001009	Circulatory System	UBERON:0007100	circulatory organ	CNhs11758	heart, adult, diseased, donor1
UBERON:0001009	Circulatory System	UBERON:0007100	circulatory organ	CNhs11789	left ventricle, adult, donor1
UBERON:0001009	Circulatory System	UBERON:0007100	circulatory organ	CNhs11790	left atrium, adult, donor1
UBERON:0001009	Circulatory System	UBERON:0007100	circulatory organ	CNhs12855	heart - mitral valve, adult
UBERON:0001009	Circulatory System	UBERON:0007100	circulatory organ	CNhs12856	heart - pulmonic valve, adult
UBERON:0001009	Circulatory System	UBERON:0007100	circulatory organ	CNhs12857	heart - tricuspid valve, adult
UBERON:0001009	Circulatory System	UBERON:0007100	circulatory organ	CNhs12498	Fibroblast - Cardiac, donor1
UBERON:0001009	Circulatory System	UBERON:0007100	circulatory organ	CNhs11088	Smooth Muscle Cells - Coronary Artery, donor1
UBERON:0001009	Circulatory System	UBERON:0007100	circulatory organ	CNhs11378	Fibroblast - Cardiac, donor2
UBERON:0001009	Circulatory System	UBERON:0007100	circulatory organ	CNhs11987	Smooth Muscle Cells - Coronary Artery, donor2
UBERON:0001009	Circulatory System	UBERON:0007100	circulatory organ	CNhs12027	Fibroblast - Cardiac, donor3
UBERON:0001009	Circulatory System	UBERON:0007100	circulatory organ	CNhs12045	Smooth Muscle Cells - Coronary Artery, donor3
UBERON:0001009	Circulatory System	UBERON:0007100	circulatory organ	CNhs11909	Fibroblast - Cardiac, donor4
UBERON:0001009	Circulatory System	UBERON:0007100	circulatory organ	CNhs12057	Fibroblast - Cardiac, donor5
UBERON:0001009	Circulatory System	UBERON:0007100	circulatory organ	CNhs12061	Fibroblast - Cardiac, donor6
UBERON:0001009	Circulatory System	UBERON:0007100	circulatory organ	CNhs12341	Cardiac Myocyte, donor1
UBERON:0001009	Circulatory System	UBERON:0007100	circulatory organ	CNhs12350	Cardiac Myocyte, donor2
UBERON:0001009	Circulatory System	UBERON:0007100	circulatory organ	CNhs12571	Cardiac Myocyte, donor3
UBERON:0001009	Circulatory System	UBERON:0007100	circulatory organ	CNhs12368	mesenchymal precursor cell - cardiac, donor1
UBERON:0001009	Circulatory System	UBERON:0007100	circulatory organ	CNhs12369	mesenchymal precursor cell - cardiac, donor2
UBERON:0001009	Circulatory System	UBERON:0007100	circulatory organ	CNhs12370	mesenchymal precursor cell - cardiac, donor3
UBERON:0001009	Circulatory System	UBERON:0007100	circulatory organ	CNhs12371	mesenchymal precursor cell - cardiac, donor4
UBERON:0001009	Circulatory System	UBERON:0007204	brachiocephalic vasculature	CNhs11086	Smooth Muscle Cells - Brachiocephalic, donor1
UBERON:0001009	Circulatory System	UBERON:0007204	brachiocephalic vasculature	CNhs11985	Smooth Muscle Cells - Brachiocephalic, donor2
UBERON:0001009	Circulatory System	UBERON:0007204	brachiocephalic vasculature	CNhs12043	Smooth Muscle Cells - Brachiocephalic, donor3
UBERON:0001009	Circulatory System	UBERON:0007240	tunica adventitia of artery	CNhs10874	Fibroblast - Aortic Adventitial, donor1
UBERON:0001009	Circulatory System	UBERON:0007240	tunica adventitia of artery	CNhs11968	Fibroblast - Aortic Adventitial, donor2
UBERON:0001009	Circulatory System	UBERON:0007240	tunica adventitia of artery	CNhs12011	Fibroblast - Aortic Adventitial, donor3
UBERON:0001009	Circulatory System	UBERON:0007798	vascular system	CNhs11760	aorta, adult, pool1
UBERON:0001009	Circulatory System	UBERON:0007798	vascular system	CNhs12843	artery, adult
UBERON:0001009	Circulatory System	UBERON:0007798	vascular system	CNhs13440	eye - vitreous humor, donor1
UBERON:0001009	Circulatory System	UBERON:0007798	vascular system	CNhs10837	Endothelial Cells - Aortic, donor0
UBERON:0001009	Circulatory System	UBERON:0007798	vascular system	CNhs10838	Smooth Muscle Cells - Aortic, donor0
UBERON:0001009	Circulatory System	UBERON:0007798	vascular system	CNhs10839	Smooth Muscle Cells - Umbilical artery, donor0
UBERON:0001009	Circulatory System	UBERON:0007798	vascular system	CNhs10863	Smooth Muscle Cells - Brain Vascular, donor1
UBERON:0001009	Circulatory System	UBERON:0007798	vascular system	CNhs10865	Endothelial Cells - Lymphatic, donor1
UBERON:0001009	Circulatory System	UBERON:0007798	vascular system	CNhs10871	Ciliary Epithelial Cells, donor1
UBERON:0001009	Circulatory System	UBERON:0007798	vascular system	CNhs10872	Endothelial Cells - Umbilical vein, donor1
UBERON:0001009	Circulatory System	UBERON:0007798	vascular system	CNhs10874	Fibroblast - Aortic Adventitial, donor1
UBERON:0001009	Circulatory System	UBERON:0007798	vascular system	CNhs10878	Fibroblast - Pulmonary Artery, donor1
UBERON:0001009	Circulatory System	UBERON:0007798	vascular system	CNhs12495	Endothelial Cells - Aortic, donor1
UBERON:0001009	Circulatory System	UBERON:0007798	vascular system	CNhs12496	Endothelial Cells - Artery, donor1
UBERON:0001009	Circulatory System	UBERON:0007798	vascular system	CNhs11925	Endothelial Cells - Microvascular, donor1
UBERON:0001009	Circulatory System	UBERON:0007798	vascular system	CNhs11926	Endothelial Cells - Thoracic, donor1

UBERON:0001009	Circulatory System	UBERON:0007798	vascular system	CNhs12497	Endothelial Cells - Vein, donor1
UBERON:0001009	Circulatory System	UBERON:0007798	vascular system	CNhs11085	Smooth Muscle Cells - Aortic, donor1
UBERON:0001009	Circulatory System	UBERON:0007798	vascular system	CNhs11086	Smooth Muscle Cells - Brachiocephalic, donor1
UBERON:0001009	Circulatory System	UBERON:0007798	vascular system	CNhs11087	Smooth Muscle Cells - Carotid, donor1
UBERON:0001009	Circulatory System	UBERON:0007798	vascular system	CNhs11088	Smooth Muscle Cells - Coronary Artery, donor1
UBERON:0001009	Circulatory System	UBERON:0007798	vascular system	CNhs11067	Smooth Muscle Cells - Internal Thoracic Artery, donor1
UBERON:0001009	Circulatory System	UBERON:0007798	vascular system	CNhs11089	Smooth Muscle Cells - Pulmonary Artery, donor1
UBERON:0001009	Circulatory System	UBERON:0007798	vascular system	CNhs11090	Smooth Muscle Cells - Subclavian Artery, donor1
UBERON:0001009	Circulatory System	UBERON:0007798	vascular system	CNhs11091	Smooth Muscle Cells - Umbilical Artery, donor1
UBERON:0001009	Circulatory System	UBERON:0007798	vascular system	CNhs11900	Smooth Muscle Cells - Brain Vascular, donor2
UBERON:0001009	Circulatory System	UBERON:0007798	vascular system	CNhs11901	Endothelial Cells - Lymphatic, donor2
UBERON:0001009	Circulatory System	UBERON:0007798	vascular system	CNhs11966	Ciliary Epithelial Cells, donor2
UBERON:0001009	Circulatory System	UBERON:0007798	vascular system	CNhs11967	Endothelial Cells - Umbilical vein, donor2
UBERON:0001009	Circulatory System	UBERON:0007798	vascular system	CNhs11968	Fibroblast - Aortic Adventitial, donor2
UBERON:0001009	Circulatory System	UBERON:0007798	vascular system	CNhs11375	Endothelial Cells - Aortic, donor2
UBERON:0001009	Circulatory System	UBERON:0007798	vascular system	CNhs11977	Endothelial Cells - Artery, donor2
UBERON:0001009	Circulatory System	UBERON:0007798	vascular system	CNhs11376	Endothelial Cells - Microvascular, donor2
UBERON:0001009	Circulatory System	UBERON:0007798	vascular system	CNhs11978	Endothelial Cells - Thoracic, donor2
UBERON:0001009	Circulatory System	UBERON:0007798	vascular system	CNhs11377	Endothelial Cells - Vein, donor2
UBERON:0001009	Circulatory System	UBERON:0007798	vascular system	CNhs11305	Smooth Muscle Cells - Aortic, donor2
UBERON:0001009	Circulatory System	UBERON:0007798	vascular system	CNhs11985	Smooth Muscle Cells - Brachiocephalic, donor2
UBERON:0001009	Circulatory System	UBERON:0007798	vascular system	CNhs11986	Smooth Muscle Cells - Carotid, donor2
UBERON:0001009	Circulatory System	UBERON:0007798	vascular system	CNhs11987	Smooth Muscle Cells - Coronary Artery, donor2
UBERON:0001009	Circulatory System	UBERON:0007798	vascular system	CNhs11988	Smooth Muscle Cells - Internal Thoracic Artery, donor2
UBERON:0001009	Circulatory System	UBERON:0007798	vascular system	CNhs11989	Smooth Muscle Cells - Pulmonary Artery, donor2
UBERON:0001009	Circulatory System	UBERON:0007798	vascular system	CNhs11990	Smooth Muscle Cells - Subclavian Artery, donor2
UBERON:0001009	Circulatory System	UBERON:0007798	vascular system	CNhs11991	Smooth Muscle Cells - Umbilical Artery, donor2
UBERON:0001009	Circulatory System	UBERON:0007798	vascular system	CNhs12004	Smooth Muscle Cells - Brain Vascular, donor3
UBERON:0001009	Circulatory System	UBERON:0007798	vascular system	CNhs11906	Endothelial Cells - Lymphatic, donor3
UBERON:0001009	Circulatory System	UBERON:0007798	vascular system	CNhs12009	Ciliary Epithelial Cells, donor3
UBERON:0001009	Circulatory System	UBERON:0007798	vascular system	CNhs12010	Endothelial Cells - Umbilical vein, donor3
UBERON:0001009	Circulatory System	UBERON:0007798	vascular system	CNhs12011	Fibroblast - Aortic Adventitial, donor3
UBERON:0001009	Circulatory System	UBERON:0007798	vascular system	CNhs12022	Endothelial Cells - Aortic, donor3
UBERON:0001009	Circulatory System	UBERON:0007798	vascular system	CNhs12023	Endothelial Cells - Artery, donor3
UBERON:0001009	Circulatory System	UBERON:0007798	vascular system	CNhs12024	Endothelial Cells - Microvascular, donor3
UBERON:0001009	Circulatory System	UBERON:0007798	vascular system	CNhs12026	Endothelial Cells - Vein, donor3
UBERON:0001009	Circulatory System	UBERON:0007798	vascular system	CNhs11309	Smooth Muscle Cells - Aortic, donor3
UBERON:0001009	Circulatory System	UBERON:0007798	vascular system	CNhs12043	Smooth Muscle Cells - Brachiocephalic, donor3
UBERON:0001009	Circulatory System	UBERON:0007798	vascular system	CNhs12044	Smooth Muscle Cells - Carotid, donor3
UBERON:0001009	Circulatory System	UBERON:0007798	vascular system	CNhs12045	Smooth Muscle Cells - Coronary Artery, donor3
UBERON:0001009	Circulatory System	UBERON:0007798	vascular system	CNhs12046	Smooth Muscle Cells - Internal Thoracic Artery, donor3
UBERON:0001009	Circulatory System	UBERON:0007798	vascular system	CNhs12047	Smooth Muscle Cells - Pulmonary Artery, donor3
UBERON:0001009	Circulatory System	UBERON:0007798	vascular system	CNhs12048	Smooth Muscle Cells - Subclavian Artery, donor3
UBERON:0001009	Circulatory System	UBERON:0007798	vascular system	CNhs12049	Smooth Muscle Cells - Umbilical Artery, donor3
UBERON:0001009	Circulatory System	UBERON:0007798	vascular system	CNhs11319	Fibroblast - Choroid Plexus, donor1
UBERON:0001009	Circulatory System	UBERON:0007798	vascular system	CNhs11322	Fibroblast - Lymphatic, donor1
UBERON:0001009	Circulatory System	UBERON:0007798	vascular system	CNhs12075	Hepatic Sinusoidal Endothelial Cells, donor1
UBERON:0001009	Circulatory System	UBERON:0007798	vascular system	CNhs12596	Iris Pigment Epithelial Cells, donor1
UBERON:0001009	Circulatory System	UBERON:0007798	vascular system	CNhs11340	Trabecular Meshwork Cells, donor1
UBERON:0001009	Circulatory System	UBERON:0007798	vascular system	CNhs12597	Smooth Muscle Cells - Umbilical Vein, donor1
UBERON:0001009	Circulatory System	UBERON:0007798	vascular system	CNhs12344	Fibroblast - Choroid Plexus, donor2
UBERON:0001009	Circulatory System	UBERON:0007798	vascular system	CNhs12082	Fibroblast - Lymphatic, donor2
UBERON:0001009	Circulatory System	UBERON:0007798	vascular system	CNhs12092	Hepatic Sinusoidal Endothelial Cells, donor2
UBERON:0001009	Circulatory System	UBERON:0007798	vascular system	CNhs12097	Trabecular Meshwork Cells, donor2
UBERON:0001009	Circulatory System	UBERON:0007798	vascular system	CNhs12569	Smooth Muscle Cells - Umbilical Vein, donor2
UBERON:0001009	Circulatory System	UBERON:0007798	vascular system	CNhs12620	Fibroblast - Choroid Plexus, donor3
UBERON:0001009	Circulatory System	UBERON:0007798	vascular system	CNhs12118	Fibroblast - Lymphatic, donor3
UBERON:0001009	Circulatory System	UBERON:0007798	vascular system	CNhs12625	Hepatic Sinusoidal Endothelial Cells, donor3
UBERON:0001009	Circulatory System	UBERON:0007798	vascular system	CNhs12124	Trabecular Meshwork Cells, donor3
UBERON:0001009	Circulatory System	UBERON:0007798	vascular system	CNhs13076	Smooth Muscle Cells - Umbilical Vein, donor3
UBERON:0001009	Circulatory System	UBERON:0008886	pulmonary vascular system	CNhs10878	Fibroblast - Pulmonary Artery, donor1
UBERON:0001009	Circulatory System	UBERON:0008886	pulmonary vascular system	CNhs11089	Smooth Muscle Cells - Pulmonary Artery, donor1
UBERON:0001009	Circulatory System	UBERON:0008886	pulmonary vascular system	CNhs11989	Smooth Muscle Cells - Pulmonary Artery, donor2
UBERON:0001009	Circulatory System	UBERON:0008886	pulmonary vascular system	CNhs12047	Smooth Muscle Cells - Pulmonary Artery, donor3
UBERON:0001009	Circulatory System	UBERON:0010260	umbilical blood vessel	CNhs10839	Smooth Muscle Cells - Umbilical artery, donor0
UBERON:0001009	Circulatory System	UBERON:0010260	umbilical blood vessel	CNhs10872	Endothelial Cells - Umbilical vein, donor1
UBERON:0001009	Circulatory System	UBERON:0010260	umbilical blood vessel	CNhs11091	Smooth Muscle Cells - Umbilical Artery, donor1
UBERON:0001009	Circulatory System	UBERON:0010260	umbilical blood vessel	CNhs11967	Endothelial Cells - Umbilical vein, donor2
UBERON:0001009	Circulatory System	UBERON:0010260	umbilical blood vessel	CNhs11991	Smooth Muscle Cells - Umbilical Artery, donor2
UBERON:0001009	Circulatory System	UBERON:0010260	umbilical blood vessel	CNhs12010	Endothelial Cells - Umbilical vein, donor3
UBERON:0001009	Circulatory System	UBERON:0010260	umbilical blood vessel	CNhs12049	Smooth Muscle Cells - Umbilical Artery, donor3
UBERON:0001009	Circulatory System	UBERON:0010260	umbilical blood vessel	CNhs12597	Smooth Muscle Cells - Umbilical Vein, donor1
UBERON:0001009	Circulatory System	UBERON:0010260	umbilical blood vessel	CNhs12569	Smooth Muscle Cells - Umbilical Vein, donor2
UBERON:0001009	Circulatory System	UBERON:0010260	umbilical blood vessel	CNhs13076	Smooth Muscle Cells - Umbilical Vein, donor3
UBERON:0001009	Circulatory System	UBERON:0010523	microcirculatory vessel	CNhs11925	Endothelial Cells - Microvascular, donor1
UBERON:0001009	Circulatory System	UBERON:0010523	microcirculatory vessel	CNhs11376	Endothelial Cells - Microvascular, donor2
UBERON:0001009	Circulatory System	UBERON:0010523	microcirculatory vessel	CNhs12024	Endothelial Cells - Microvascular, donor3
UBERON:0001017	Central Nervous System	UBERON:0000007	pituitary gland	CNhs12229	pituitary gland, adult, donor10252
UBERON:0001017	Central Nervous System	UBERON:0000007	pituitary gland	CNhs13805	pituitary gland - adult, donor10196
UBERON:0001017	Central Nervous System	UBERON:0000007	pituitary gland	CNhs14231	pituitary gland, adult, donor10258
UBERON:0001017	Central Nervous System	UBERON:0000200	gyrus	CNhs10638	postcentral gyrus, adult, pool1
UBERON:0001017	Central Nervous System	UBERON:0000200	gyrus	CNhs10642	paracentral gyrus, adult, pool1
UBERON:0001017	Central Nervous System	UBERON:0000200	gyrus	CNhs12310	medial frontal gyrus, adult, donor10252
UBERON:0001017	Central Nervous System	UBERON:0000200	gyrus	CNhs12316	medial temporal gyrus, adult, donor10252
UBERON:0001017	Central Nervous System	UBERON:0000200	gyrus	CNhs13796	medial frontal gyrus - adult, donor10196
UBERON:0001017	Central Nervous System	UBERON:0000200	gyrus	CNhs13809	medial temporal gyrus - adult, donor10196
UBERON:0001017	Central Nervous System	UBERON:0000200	gyrus	CNhs14069	medial frontal gyrus, newborn, donor10223
UBERON:0001017	Central Nervous System	UBERON:0000200	gyrus	CNhs14070	medial temporal gyrus, newborn, donor10223
UBERON:0001017	Central Nervous System	UBERON:0000200	gyrus	CNhs14221	medial frontal gyrus, adult, donor10258
UBERON:0001017	Central Nervous System	UBERON:0000200	gyrus	Cnhs14229	medial temporal gyrus, adult, donor10258
UBERON:0001017	Central Nervous System	UBERON:0000203	pallium	CNhs10637	temporal lobe, adult, pool1
UBERON:0001017	Central Nervous System	UBERON:0000203	pallium	CNhs10638	postcentral gyrus, adult, pool1
UBERON:0001017	Central Nervous System	UBERON:0000203	pallium	CNhs10641	parietal lobe, adult, pool1
UBERON:0001017	Central Nervous System	UBERON:0000203	pallium	CNhs10643	occipital pole, adult, pool1
UBERON:0001017	Central Nervous System	UBERON:0000203	pallium	CNhs10646	insula, adult, pool1
UBERON:0001017	Central Nervous System	UBERON:0000203	pallium	CNhs10647	frontal lobe, adult, pool1
UBERON:0001017	Central Nervous System	UBERON:0000203	pallium	Cnhs11781	duodenum, fetal, donor1
UBERON:0001017	Central Nervous System	UBERON:0000203	pallium	CNhs11782	parietal lobe, fetal, donor1

UBERON:0001017	Central Nervous System	UBERON:0000203	pallium	CNhs11784	occipital lobe, fetal, donor1
UBERON:0001017	Central Nervous System	UBERON:0000203	pallium	CNhs11787	occipital lobe, adult, donor1
UBERON:0001017	Central Nervous System	UBERON:0000203	pallium	CNhs12310	medial frontal gyrus, adult, donor10252
UBERON:0001017	Central Nervous System	UBERON:0000203	pallium	CNhs12311	amygdala, adult, donor10252
UBERON:0001017	Central Nervous System	UBERON:0000203	pallium	CNhs12312	hippocampus, adult, donor10252
UBERON:0001017	Central Nervous System	UBERON:0000203	pallium	CNhs12316	medial temporal gyrus, adult, donor10252
UBERON:0001017	Central Nervous System	UBERON:0000203	pallium	CNhs12317	parietal lobe, adult, donor10252
UBERON:0001017	Central Nervous System	UBERON:0000203	pallium	CNhs12320	occipital cortex, adult, donor10252
UBERON:0001017	Central Nervous System	UBERON:0000203	pallium	CNhs13793	amygdala - adult, donor10196
UBERON:0001017	Central Nervous System	UBERON:0000203	pallium	CNhs13795	hippocampus - adult, donor10196
UBERON:0001017	Central Nervous System	UBERON:0000203	pallium	CNhs13796	medial frontal gyrus - adult, donor10196
UBERON:0001017	Central Nervous System	UBERON:0000203	pallium	CNhs13797	parietal lobe - adult, donor10196
UBERON:0001017	Central Nervous System	UBERON:0000203	pallium	CNhs13798	occipital cortex - adult, donor10196
UBERON:0001017	Central Nervous System	UBERON:0000203	pallium	CNhs13809	medial temporal gyrus - adult, donor10196
UBERON:0001017	Central Nervous System	UBERON:0000203	pallium	CNhs14069	medial frontal gyrus, newborn, donor10223
UBERON:0001017	Central Nervous System	UBERON:0000203	pallium	CNhs14070	medial temporal gyrus, newborn, donor10223
UBERON:0001017	Central Nervous System	UBERON:0000203	pallium	CNhs14073	occipital cortex, newborn, donor10223
UBERON:0001017	Central Nervous System	UBERON:0000203	pallium	CNhs14074	parietal lobe, newborn, donor10223
UBERON:0001017	Central Nervous System	UBERON:0000203	pallium	CNhs14078	amygdala, newborn, donor10223
UBERON:0001017	Central Nervous System	UBERON:0000203	pallium	CNhs14081	hippocampus, newborn, donor10223
UBERON:0001017	Central Nervous System	UBERON:0000203	pallium	CNhs14221	medial frontal gyrus, adult, donor10258
UBERON:0001017	Central Nervous System	UBERON:0000203	pallium	CNhs14226	parietal cortex, adult, donor10258
UBERON:0001017	Central Nervous System	UBERON:0000203	pallium	CNhs14227	hippocampus, adult, donor10258
UBERON:0001017	Central Nervous System	UBERON:0000203	pallium	Cnhs14229	medial temporal gyrus, adult, donor10258
UBERON:0001017	Central Nervous System	UBERON:0000203	pallium	CNhs10864	Astrocyte - cerebral cortex, donor1
UBERON:0001017	Central Nervous System	UBERON:0000203	pallium	CNhs11960	Astrocyte - cerebral cortex, donor2
UBERON:0001017	Central Nervous System	UBERON:0000203	pallium	CNhs12005	Astrocyte - cerebral cortex, donor3
UBERON:0001017	Central Nervous System	UBERON:0000204	ventral part of telencephalon	CNhs10644	nucleus accumbens, adult, pool1
UBERON:0001017	Central Nervous System	UBERON:0000204	ventral part of telencephalon	CNhs13912	putamen, adult, donor10252
UBERON:0001017	Central Nervous System	UBERON:0000204	ventral part of telencephalon	CNhs12321	caudate nucleus, adult, donor10252
UBERON:0001017	Central Nervous System	UBERON:0000204	ventral part of telencephalon	CNhs12324	putamen, adult, donor10196
UBERON:0001017	Central Nervous System	UBERON:0000204	ventral part of telencephalon	CNhs13802	caudate nucleus - adult, donor10196
UBERON:0001017	Central Nervous System	UBERON:0000204	ventral part of telencephalon	CNhs14071	caudate nucleus, newborn, donor10223
UBERON:0001017	Central Nervous System	UBERON:0000204	ventral part of telencephalon	CNhs14083	putamen, newborn, donor10223
UBERON:0001017	Central Nervous System	UBERON:0000204	ventral part of telencephalon	Cnhs14225	putamen, adult, donor10258
UBERON:0001017	Central Nervous System	UBERON:0000349	limbic system	CNhs10644	nucleus accumbens, adult, pool1
UBERON:0001017	Central Nervous System	UBERON:0000349	limbic system	CNhs12311	amygdala, adult, donor10252
UBERON:0001017	Central Nervous System	UBERON:0000349	limbic system	CNhs12312	hippocampus, adult, donor10252
UBERON:0001017	Central Nervous System	UBERON:0000349	limbic system	CNhs13793	amygdala - adult, donor10196
UBERON:0001017	Central Nervous System	UBERON:0000349	limbic system	CNhs13795	hippocampus - adult, donor10196
UBERON:0001017	Central Nervous System	UBERON:0000349	limbic system	CNhs14078	amygdala, newborn, donor10223
UBERON:0001017	Central Nervous System	UBERON:0000349	limbic system	CNhs14081	hippocampus, newborn, donor10223
UBERON:0001017	Central Nervous System	UBERON:0000349	limbic system	CNhs14227	hippocampus, adult, donor10258
UBERON:0001017	Central Nervous System	UBERON:0000369	corpus striatum	CNhs10644	nucleus accumbens, adult, pool1
UBERON:0001017	Central Nervous System	UBERON:0000369	corpus striatum	CNhs13912	putamen, adult, donor10252
UBERON:0001017	Central Nervous System	UBERON:0000369	corpus striatum	CNhs12321	caudate nucleus, adult, donor10252
UBERON:0001017	Central Nervous System	UBERON:0000369	corpus striatum	CNhs12324	putamen, adult, donor10196
UBERON:0001017	Central Nervous System	UBERON:0000369	corpus striatum	CNhs13802	caudate nucleus - adult, donor10196
UBERON:0001017	Central Nervous System	UBERON:0000369	corpus striatum	CNhs14071	caudate nucleus, newborn, donor10223
UBERON:0001017	Central Nervous System	UBERON:0000369	corpus striatum	CNhs14083	putamen, newborn, donor10223
UBERON:0001017	Central Nervous System	UBERON:0000369	corpus striatum	Cnhs14225	putamen, adult, donor10258
UBERON:0001017	Central Nervous System	UBERON:0000955	brain	CNhs10617	brain, adult, pool1
UBERON:0001017	Central Nervous System	UBERON:0000955	brain	CNhs10637	temporal lobe, adult, pool1
UBERON:0001017	Central Nervous System	UBERON:0000955	brain	CNhs10638	postcentral gyrus, adult, pool1
UBERON:0001017	Central Nervous System	UBERON:0000955	brain	CNhs10640	pons, adult, pool1
UBERON:0001017	Central Nervous System	UBERON:0000955	brain	CNhs10641	parietal lobe, adult, pool1
UBERON:0001017	Central Nervous System	UBERON:0000955	brain	CNhs10642	paracentral gyrus, adult, pool1
UBERON:0001017	Central Nervous System	UBERON:0000955	brain	CNhs10643	occipital pole, adult, pool1
UBERON:0001017	Central Nervous System	UBERON:0000955	brain	CNhs10644	nucleus accumbens, adult, pool1
UBERON:0001017	Central Nervous System	UBERON:0000955	brain	CNhs10645	medulla oblongata, adult, pool1
UBERON:0001017	Central Nervous System	UBERON:0000955	brain	CNhs10646	insula, adult, pool1
UBERON:0001017	Central Nervous System	UBERON:0000955	brain	CNhs10647	frontal lobe, adult, pool1
UBERON:0001017	Central Nervous System	UBERON:0000955	brain	CNhs10649	corpus callosum, adult, pool1
UBERON:0001017	Central Nervous System	UBERON:0000955	brain	Cnhs11781	duodenum, fetal, donor1
UBERON:0001017	Central Nervous System	UBERON:0000955	brain	CNhs11782	parietal lobe, fetal, donor1
UBERON:0001017	Central Nervous System	UBERON:0000955	brain	CNhs11784	occipital lobe, fetal, donor1
UBERON:0001017	Central Nervous System	UBERON:0000955	brain	CNhs11787	occipital lobe, adult, donor1
UBERON:0001017	Central Nervous System	UBERON:0000955	brain	CNhs11795	cerebellum, adult, pool1
UBERON:0001017	Central Nervous System	UBERON:0000955	brain	CNhs11796	brain, adult, donor1
UBERON:0001017	Central Nervous System	UBERON:0000955	brain	CNhs11797	brain, fetal, pool1
UBERON:0001017	Central Nervous System	UBERON:0000955	brain	CNhs12310	medial frontal gyrus, adult, donor10252
UBERON:0001017	Central Nervous System	UBERON:0000955	brain	CNhs12311	amygdala, adult, donor10252
UBERON:0001017	Central Nervous System	UBERON:0000955	brain	CNhs13912	putamen, adult, donor10252
UBERON:0001017	Central Nervous System	UBERON:0000955	brain	CNhs12312	hippocampus, adult, donor10252
UBERON:0001017	Central Nervous System	UBERON:0000955	brain	CNhs12314	thalamus, adult, donor10252
UBERON:0001017	Central Nervous System	UBERON:0000955	brain	CNhs12315	medulla oblongata, adult, donor10252
UBERON:0001017	Central Nervous System	UBERON:0000955	brain	CNhs12316	medial temporal gyrus, adult, donor10252
UBERON:0001017	Central Nervous System	UBERON:0000955	brain	CNhs12317	parietal lobe, adult, donor10252
UBERON:0001017	Central Nervous System	UBERON:0000955	brain	CNhs12318	substantia nigra, adult, donor10252
UBERON:0001017	Central Nervous System	UBERON:0000955	brain	CNhs12228	pineal gland, adult, donor10252
UBERON:0001017	Central Nervous System	UBERON:0000955	brain	CNhs12319	globus pallidus, adult, donor10252
UBERON:0001017	Central Nervous System	UBERON:0000955	brain	CNhs12229	pituitary gland, adult, donor10252
UBERON:0001017	Central Nervous System	UBERON:0000955	brain	CNhs12320	occipital cortex, adult, donor10252
UBERON:0001017	Central Nervous System	UBERON:0000955	brain	CNhs12321	caudate nucleus, adult, donor10252
UBERON:0001017	Central Nervous System	UBERON:0000955	brain	CNhs12322	locus coeruleus, adult, donor10252
UBERON:0001017	Central Nervous System	UBERON:0000955	brain	CNhs12323	cerebellum, adult, donor10252
UBERON:0001017	Central Nervous System	UBERON:0000955	brain	CNhs13793	amygdala - adult, donor10196
UBERON:0001017	Central Nervous System	UBERON:0000955	brain	CNhs13794	thalamus - adult, donor10196
UBERON:0001017	Central Nervous System	UBERON:0000955	brain	CNhs13795	hippocampus - adult, donor10196
UBERON:0001017	Central Nervous System	UBERON:0000955	brain	CNhs13796	medial frontal gyrus - adult, donor10196
UBERON:0001017	Central Nervous System	UBERON:0000955	brain	CNhs13797	parietal lobe - adult, donor10196
UBERON:0001017	Central Nervous System	UBERON:0000955	brain	CNhs13798	occipital cortex - adult, donor10196
UBERON:0001017	Central Nervous System	UBERON:0000955	brain	CNhs13799	cerebellum - adult, donor10196
UBERON:0001017	Central Nervous System	UBERON:0000955	brain	CNhs13800	medulla oblongata - adult, donor10196
UBERON:0001017	Central Nervous System	UBERON:0000955	brain	CNhs13801	globus pallidus - adult, donor10196
UBERON:0001017	Central Nervous System	UBERON:0000955	brain	CNhs12324	putamen, adult, donor10196
UBERON:0001017	Central Nervous System	UBERON:0000955	brain	CNhs13802	caudate nucleus - adult, donor10196
UBERON:0001017	Central Nervous System	UBERON:0000955	brain	CNhs13803	substantia nigra - adult, donor10196

UBERON:0001017	Central Nervous System	UBERON:0000955	brain	CNhs13804	pineal gland - adult, donor10196
UBERON:0001017	Central Nervous System	UBERON:0000955	brain	CNhs13805	pituitary gland - adult, donor10196
UBERON:0001017	Central Nervous System	UBERON:0000955	brain	CNhs13808	locus coeruleus - adult, donor10196
UBERON:0001017	Central Nervous System	UBERON:0000955	brain	CNhs13809	medial temporal gyrus - adult, donor10196
UBERON:0001017	Central Nervous System	UBERON:0000955	brain	CNhs12610	diencephalon, adult
UBERON:0001017	Central Nervous System	UBERON:0000955	brain	CNhs14069	medial frontal gyrus, newborn, donor10223
UBERON:0001017	Central Nervous System	UBERON:0000955	brain	CNhs14070	medial temporal gyrus, newborn, donor10223
UBERON:0001017	Central Nervous System	UBERON:0000955	brain	CNhs14071	caudate nucleus, newborn, donor10223
UBERON:0001017	Central Nervous System	UBERON:0000955	brain	CNhs14073	occipital cortex, newborn, donor10223
UBERON:0001017	Central Nervous System	UBERON:0000955	brain	CNhs14074	parietal lobe, newborn, donor10223
UBERON:0001017	Central Nervous System	UBERON:0000955	brain	CNhs14075	cerebellum, newborn, donor10223
UBERON:0001017	Central Nervous System	UBERON:0000955	brain	CNhs14076	substantia nigra, newborn, donor10223
UBERON:0001017	Central Nervous System	UBERON:0000955	brain	CNhs14078	amygdala, newborn, donor10223
UBERON:0001017	Central Nervous System	UBERON:0000955	brain	CNhs14079	medulla oblongata, newborn, donor10223
UBERON:0001017	Central Nervous System	UBERON:0000955	brain	CNhs14080	locus coeruleus, newborn, donor10223
UBERON:0001017	Central Nervous System	UBERON:0000955	brain	CNhs14081	hippocampus, newborn, donor10223
UBERON:0001017	Central Nervous System	UBERON:0000955	brain	CNhs14082	globus pallidus, newborn, donor10223
UBERON:0001017	Central Nervous System	UBERON:0000955	brain	CNhs14083	putamen, newborn, donor10223
UBERON:0001017	Central Nervous System	UBERON:0000955	brain	CNhs14084	thalamus, newborn, donor10223
UBERON:0001017	Central Nervous System	UBERON:0000955	brain	CNhs14549	globus pallidus, adult, donor10258
UBERON:0001017	Central Nervous System	UBERON:0000955	brain	CNhs14221	medial frontal gyrus, adult, donor10258
UBERON:0001017	Central Nervous System	UBERON:0000955	brain	Cnhs14223	thalamus, adult, donor10258
UBERON:0001017	Central Nervous System	UBERON:0000955	brain	CNhs14224	substantia nigra, adult, donor10258
UBERON:0001017	Central Nervous System	UBERON:0000955	brain	Cnhs14225	putamen, adult, donor10258
UBERON:0001017	Central Nervous System	UBERON:0000955	brain	CNhs14226	parietal cortex, adult, donor10258
UBERON:0001017	Central Nervous System	UBERON:0000955	brain	CNhs14227	hippocampus, adult, donor10258
UBERON:0001017	Central Nervous System	UBERON:0000955	brain	CNhs14550	locus coeruleus, adult, donor10258
UBERON:0001017	Central Nervous System	UBERON:0000955	brain	Cnhs14229	medial temporal gyrus, adult, donor10258
UBERON:0001017	Central Nervous System	UBERON:0000955	brain	CNhs14230	pineal gland, adult, donor10258
UBERON:0001017	Central Nervous System	UBERON:0000955	brain	CNhs14231	pituitary gland, adult, donor10258
UBERON:0001017	Central Nervous System	UBERON:0000955	brain	CNhs10863	Smooth Muscle Cells - Brain Vascular, donor1
UBERON:0001017	Central Nervous System	UBERON:0000955	brain	CNhs10864	Astrocyte - cerebral cortex, donor1
UBERON:0001017	Central Nervous System	UBERON:0000955	brain	CNhs11900	Smooth Muscle Cells - Brain Vascular, donor2
UBERON:0001017	Central Nervous System	UBERON:0000955	brain	CNhs11960	Astrocyte - cerebral cortex, donor2
UBERON:0001017	Central Nervous System	UBERON:0000955	brain	CNhs12004	Smooth Muscle Cells - Brain Vascular, donor3
UBERON:0001017	Central Nervous System	UBERON:0000955	brain	CNhs12005	Astrocyte - cerebral cortex, donor3
UBERON:0001017	Central Nervous System	UBERON:0000955	brain	CNhs11319	Fibroblast - Choroid Plexus, donor1
UBERON:0001017	Central Nervous System	UBERON:0000955	brain	CNhs11321	Astrocyte - cerebellum, donor1
UBERON:0001017	Central Nervous System	UBERON:0000955	brain	CNhs12344	Fibroblast - Choroid Plexus, donor2
UBERON:0001017	Central Nervous System	UBERON:0000955	brain	CNhs12081	Astrocyte - cerebellum, donor2
UBERON:0001017	Central Nervous System	UBERON:0000955	brain	CNhs12620	Fibroblast - Choroid Plexus, donor3
UBERON:0001017	Central Nervous System	UBERON:0000955	brain	CNhs12117	Astrocyte - cerebellum, donor3
UBERON:0001017	Central Nervous System	UBERON:0000956	cerebral cortex	CNhs10637	temporal lobe, adult, pool1
UBERON:0001017	Central Nervous System	UBERON:0000956	cerebral cortex	CNhs10638	postcentral gyrus, adult, pool1
UBERON:0001017	Central Nervous System	UBERON:0000956	cerebral cortex	CNhs10641	parietal lobe, adult, pool1
UBERON:0001017	Central Nervous System	UBERON:0000956	cerebral cortex	CNhs10643	occipital pole, adult, pool1
UBERON:0001017	Central Nervous System	UBERON:0000956	cerebral cortex	CNhs10646	insula, adult, pool1
UBERON:0001017	Central Nervous System	UBERON:0000956	cerebral cortex	CNhs10647	frontal lobe, adult, pool1
UBERON:0001017	Central Nervous System	UBERON:0000956	cerebral cortex	Cnhs11781	duodenum, fetal, donor1
UBERON:0001017	Central Nervous System	UBERON:0000956	cerebral cortex	CNhs11782	parietal lobe, fetal, donor1
UBERON:0001017	Central Nervous System	UBERON:0000956	cerebral cortex	CNhs11784	occipital lobe, fetal, donor1
UBERON:0001017	Central Nervous System	UBERON:0000956	cerebral cortex	CNhs11787	occipital lobe, adult, donor1
UBERON:0001017	Central Nervous System	UBERON:0000956	cerebral cortex	CNhs12310	medial frontal gyrus, adult, donor10252
UBERON:0001017	Central Nervous System	UBERON:0000956	cerebral cortex	CNhs12311	amygdala, adult, donor10252
UBERON:0001017	Central Nervous System	UBERON:0000956	cerebral cortex	CNhs12312	hippocampus, adult, donor10252
UBERON:0001017	Central Nervous System	UBERON:0000956	cerebral cortex	CNhs12316	medial temporal gyrus, adult, donor10252
UBERON:0001017	Central Nervous System	UBERON:0000956	cerebral cortex	CNhs12317	parietal lobe, adult, donor10252
UBERON:0001017	Central Nervous System	UBERON:0000956	cerebral cortex	CNhs12320	occipital cortex, adult, donor10252
UBERON:0001017	Central Nervous System	UBERON:0000956	cerebral cortex	CNhs13793	amygdala - adult, donor10196
UBERON:0001017	Central Nervous System	UBERON:0000956	cerebral cortex	CNhs13795	hippocampus - adult, donor10196
UBERON:0001017	Central Nervous System	UBERON:0000956	cerebral cortex	CNhs13796	medial frontal gyrus - adult, donor10196
UBERON:0001017	Central Nervous System	UBERON:0000956	cerebral cortex	CNhs13797	parietal lobe - adult, donor10196
UBERON:0001017	Central Nervous System	UBERON:0000956	cerebral cortex	CNhs13798	occipital cortex - adult, donor10196
UBERON:0001017	Central Nervous System	UBERON:0000956	cerebral cortex	CNhs13809	medial temporal gyrus - adult, donor10196
UBERON:0001017	Central Nervous System	UBERON:0000956	cerebral cortex	CNhs14069	medial frontal gyrus, newborn, donor10223
UBERON:0001017	Central Nervous System	UBERON:0000956	cerebral cortex	CNhs14070	medial temporal gyrus, newborn, donor10223
UBERON:0001017	Central Nervous System	UBERON:0000956	cerebral cortex	CNhs14073	occipital cortex, newborn, donor10223
UBERON:0001017	Central Nervous System	UBERON:0000956	cerebral cortex	CNhs14074	parietal lobe, newborn, donor10223
UBERON:0001017	Central Nervous System	UBERON:0000956	cerebral cortex	CNhs14078	amygdala, newborn, donor10223
UBERON:0001017	Central Nervous System	UBERON:0000956	cerebral cortex	CNhs14081	hippocampus, newborn, donor10223
UBERON:0001017	Central Nervous System	UBERON:0000956	cerebral cortex	CNhs14221	medial frontal gyrus, adult, donor10258
UBERON:0001017	Central Nervous System	UBERON:0000956	cerebral cortex	CNhs14226	parietal cortex, adult, donor10258
UBERON:0001017	Central Nervous System	UBERON:0000956	cerebral cortex	CNhs14227	hippocampus, adult, donor10258
UBERON:0001017	Central Nervous System	UBERON:0000956	cerebral cortex	Cnhs14229	medial temporal gyrus, adult, donor10258
UBERON:0001017	Central Nervous System	UBERON:0000956	cerebral cortex	CNhs10864	Astrocyte - cerebral cortex, donor1
UBERON:0001017	Central Nervous System	UBERON:0000956	cerebral cortex	CNhs11960	Astrocyte - cerebral cortex, donor2
UBERON:0001017	Central Nervous System	UBERON:0000956	cerebral cortex	CNhs12005	Astrocyte - cerebral cortex, donor3
UBERON:0001017	Central Nervous System	UBERON:0000988	pons	CNhs10640	pons, adult, pool1
UBERON:0001017	Central Nervous System	UBERON:0000988	pons	CNhs12322	locus coeruleus, adult, donor10252
UBERON:0001017	Central Nervous System	UBERON:0000988	pons	CNhs13808	locus coeruleus - adult, donor10196
UBERON:0001017	Central Nervous System	UBERON:0000988	pons	CNhs14080	locus coeruleus, newborn, donor10223
UBERON:0001017	Central Nervous System	UBERON:0000988	pons	CNhs14550	locus coeruleus, adult, donor10258
UBERON:0001017	Central Nervous System	UBERON:0001017	central nervous system	CNhs10617	brain, adult, pool1
UBERON:0001017	Central Nervous System	UBERON:0001017	central nervous system	CNhs10637	temporal lobe, adult, pool1
UBERON:0001017	Central Nervous System	UBERON:0001017	central nervous system	CNhs10638	postcentral gyrus, adult, pool1
UBERON:0001017	Central Nervous System	UBERON:0001017	central nervous system	CNhs10640	pons, adult, pool1
UBERON:0001017	Central Nervous System	UBERON:0001017	central nervous system	CNhs10641	parietal lobe, adult, pool1
UBERON:0001017	Central Nervous System	UBERON:0001017	central nervous system	CNhs10642	paracentral gyrus, adult, pool1
UBERON:0001017	Central Nervous System	UBERON:0001017	central nervous system	CNhs10643	occipital pole, adult, pool1
UBERON:0001017	Central Nervous System	UBERON:0001017	central nervous system	CNhs10644	nucleus accumbens, adult, pool1
UBERON:0001017	Central Nervous System	UBERON:0001017	central nervous system	CNhs10645	medulla oblongata, adult, pool1
UBERON:0001017	Central Nervous System	UBERON:0001017	central nervous system	CNhs10646	insula, adult, pool1
UBERON:0001017	Central Nervous System	UBERON:0001017	central nervous system	CNhs10647	frontal lobe, adult, pool1
UBERON:0001017	Central Nervous System	UBERON:0001017	central nervous system	CNhs10648	dura mater, adult, donor1
UBERON:0001017	Central Nervous System	UBERON:0001017	central nervous system	CNhs10649	corpus callosum, adult, pool1
UBERON:0001017	Central Nervous System	UBERON:0001017	central nervous system	CNhs11764	spinal cord, fetal, donor1
UBERON:0001017	Central Nervous System	UBERON:0001017	central nervous system	Cnhs11781	duodenum, fetal, donor1
UBERON:0001017	Central Nervous System	UBERON:0001017	central nervous system	CNhs11782	parietal lobe, fetal, donor1

UBERON:0001017	Central Nervous System	UBERON:0001017	central nervous system	CNhs11784	occipital lobe, fetal, donor1
UBERON:0001017	Central Nervous System	UBERON:0001017	central nervous system	CNhs11787	occipital lobe, adult, donor1
UBERON:0001017	Central Nervous System	UBERON:0001017	central nervous system	CNhs11795	cerebellum, adult, pool1
UBERON:0001017	Central Nervous System	UBERON:0001017	central nervous system	CNhs11796	brain, adult, donor1
UBERON:0001017	Central Nervous System	UBERON:0001017	central nervous system	CNhs11797	brain, fetal, pool1
UBERON:0001017	Central Nervous System	UBERON:0001017	central nervous system	CNhs12310	medial frontal gyrus, adult, donor10252
UBERON:0001017	Central Nervous System	UBERON:0001017	central nervous system	CNhs12311	amygdala, adult, donor10252
UBERON:0001017	Central Nervous System	UBERON:0001017	central nervous system	CNhs13912	putamen, adult, donor10252
UBERON:0001017	Central Nervous System	UBERON:0001017	central nervous system	CNhs12312	hippocampus, adult, donor10252
UBERON:0001017	Central Nervous System	UBERON:0001017	central nervous system	CNhs12314	thalamus, adult, donor10252
UBERON:0001017	Central Nervous System	UBERON:0001017	central nervous system	CNhs12315	medulla oblongata, adult, donor10252
UBERON:0001017	Central Nervous System	UBERON:0001017	central nervous system	CNhs12316	medial temporal gyrus, adult, donor10252
UBERON:0001017	Central Nervous System	UBERON:0001017	central nervous system	CNhs12317	parietal lobe, adult, donor10252
UBERON:0001017	Central Nervous System	UBERON:0001017	central nervous system	CNhs12318	substantia nigra, adult, donor10252
UBERON:0001017	Central Nervous System	UBERON:0001017	central nervous system	CNhs12227	spinal cord, adult, donor10252
UBERON:0001017	Central Nervous System	UBERON:0001017	central nervous system	CNhs12228	pineal gland, adult, donor10252
UBERON:0001017	Central Nervous System	UBERON:0001017	central nervous system	CNhs12319	globus pallidus, adult, donor10252
UBERON:0001017	Central Nervous System	UBERON:0001017	central nervous system	CNhs12229	pituitary gland, adult, donor10252
UBERON:0001017	Central Nervous System	UBERON:0001017	central nervous system	CNhs12320	occipital cortex, adult, donor10252
UBERON:0001017	Central Nervous System	UBERON:0001017	central nervous system	CNhs12321	caudate nucleus, adult, donor10252
UBERON:0001017	Central Nervous System	UBERON:0001017	central nervous system	CNhs12322	locus coeruleus, adult, donor10252
UBERON:0001017	Central Nervous System	UBERON:0001017	central nervous system	CNhs12323	cerebellum, adult, donor10252
UBERON:0001017	Central Nervous System	UBERON:0001017	central nervous system	CNhs13793	amygdala - adult, donor10196
UBERON:0001017	Central Nervous System	UBERON:0001017	central nervous system	CNhs13794	thalamus - adult, donor10196
UBERON:0001017	Central Nervous System	UBERON:0001017	central nervous system	CNhs13795	hippocampus - adult, donor10196
UBERON:0001017	Central Nervous System	UBERON:0001017	central nervous system	CNhs13796	medial frontal gyrus - adult, donor10196
UBERON:0001017	Central Nervous System	UBERON:0001017	central nervous system	CNhs13797	parietal lobe - adult, donor10196
UBERON:0001017	Central Nervous System	UBERON:0001017	central nervous system	CNhs13798	occipital cortex - adult, donor10196
UBERON:0001017	Central Nervous System	UBERON:0001017	central nervous system	CNhs13799	cerebellum - adult, donor10196
UBERON:0001017	Central Nervous System	UBERON:0001017	central nervous system	CNhs13800	medulla oblongata - adult, donor10196
UBERON:0001017	Central Nervous System	UBERON:0001017	central nervous system	CNhs13801	globus pallidus - adult, donor10196
UBERON:0001017	Central Nervous System	UBERON:0001017	central nervous system	CNhs12324	putamen, adult, donor10196
UBERON:0001017	Central Nervous System	UBERON:0001017	central nervous system	CNhs13802	caudate nucleus - adult, donor10196
UBERON:0001017	Central Nervous System	UBERON:0001017	central nervous system	CNhs13803	substantia nigra - adult, donor10196
UBERON:0001017	Central Nervous System	UBERON:0001017	central nervous system	CNhs13804	pineal gland - adult, donor10196
UBERON:0001017	Central Nervous System	UBERON:0001017	central nervous system	CNhs13805	pituitary gland - adult, donor10196
UBERON:0001017	Central Nervous System	UBERON:0001017	central nervous system	CNhs13807	spinal cord - adult, donor10196
UBERON:0001017	Central Nervous System	UBERON:0001017	central nervous system	CNhs13808	locus coeruleus - adult, donor10196
UBERON:0001017	Central Nervous System	UBERON:0001017	central nervous system	CNhs13809	medial temporal gyrus - adult, donor10196
UBERON:0001017	Central Nervous System	UBERON:0001017	central nervous system	CNhs12840	cerebral meninges, adult
UBERON:0001017	Central Nervous System	UBERON:0001017	central nervous system	CNhs12610	diencephalon, adult
UBERON:0001017	Central Nervous System	UBERON:0001017	central nervous system	CNhs14069	medial frontal gyrus, newborn, donor10223
UBERON:0001017	Central Nervous System	UBERON:0001017	central nervous system	CNhs14070	medial temporal gyrus, newborn, donor10223
UBERON:0001017	Central Nervous System	UBERON:0001017	central nervous system	CNhs14071	caudate nucleus, newborn, donor10223
UBERON:0001017	Central Nervous System	UBERON:0001017	central nervous system	CNhs14073	occipital cortex, newborn, donor10223
UBERON:0001017	Central Nervous System	UBERON:0001017	central nervous system	CNhs14074	parietal lobe, newborn, donor10223
UBERON:0001017	Central Nervous System	UBERON:0001017	central nervous system	CNhs14075	cerebellum, newborn, donor10223
UBERON:0001017	Central Nervous System	UBERON:0001017	central nervous system	CNhs14076	substantia nigra, newborn, donor10223
UBERON:0001017	Central Nervous System	UBERON:0001017	central nervous system	CNhs14077	spinal cord, newborn, donor10223
UBERON:0001017	Central Nervous System	UBERON:0001017	central nervous system	CNhs14078	amygdala, newborn, donor10223
UBERON:0001017	Central Nervous System	UBERON:0001017	central nervous system	CNhs14079	medulla oblongata, newborn, donor10223
UBERON:0001017	Central Nervous System	UBERON:0001017	central nervous system	CNhs14080	locus coeruleus, newborn, donor10223
UBERON:0001017	Central Nervous System	UBERON:0001017	central nervous system	CNhs14081	hippocampus, newborn, donor10223
UBERON:0001017	Central Nervous System	UBERON:0001017	central nervous system	CNhs14082	globus pallidus, newborn, donor10223
UBERON:0001017	Central Nervous System	UBERON:0001017	central nervous system	CNhs14083	putamen, newborn, donor10223
UBERON:0001017	Central Nervous System	UBERON:0001017	central nervous system	CNhs14084	thalamus, newborn, donor10223
UBERON:0001017	Central Nervous System	UBERON:0001017	central nervous system	CNhs14549	globus pallidus, adult, donor10258
UBERON:0001017	Central Nervous System	UBERON:0001017	central nervous system	CNhs14221	medial frontal gyrus, adult, donor10258
UBERON:0001017	Central Nervous System	UBERON:0001017	central nervous system	CNhs14222	spinal cord, adult, donor10258
UBERON:0001017	Central Nervous System	UBERON:0001017	central nervous system	Cnhs14223	thalamus, adult, donor10258
UBERON:0001017	Central Nervous System	UBERON:0001017	central nervous system	CNhs14224	substantia nigra, adult, donor10258
UBERON:0001017	Central Nervous System	UBERON:0001017	central nervous system	Cnhs14225	putamen, adult, donor10258
UBERON:0001017	Central Nervous System	UBERON:0001017	central nervous system	CNhs14226	parietal cortex, adult, donor10258
UBERON:0001017	Central Nervous System	UBERON:0001017	central nervous system	CNhs14227	hippocampus, adult, donor10258
UBERON:0001017	Central Nervous System	UBERON:0001017	central nervous system	CNhs14550	locus coeruleus, adult, donor10258
UBERON:0001017	Central Nervous System	UBERON:0001017	central nervous system	Cnhs14229	medial temporal gyrus, adult, donor10258
UBERON:0001017	Central Nervous System	UBERON:0001017	central nervous system	CNhs14230	pineal gland, adult, donor10258
UBERON:0001017	Central Nervous System	UBERON:0001017	central nervous system	CNhs14231	pituitary gland, adult, donor10258
UBERON:0001017	Central Nervous System	UBERON:0001017	central nervous system	CNhs10863	Smooth Muscle Cells - Brain Vascular, donor1
UBERON:0001017	Central Nervous System	UBERON:0001017	central nervous system	CNhs10864	Astrocyte - cerebral cortex, donor1
UBERON:0001017	Central Nervous System	UBERON:0001017	central nervous system	CNhs11063	Neural stem cells, donor1
UBERON:0001017	Central Nervous System	UBERON:0001017	central nervous system	CNhs11900	Smooth Muscle Cells - Brain Vascular, donor2
UBERON:0001017	Central Nervous System	UBERON:0001017	central nervous system	CNhs11960	Astrocyte - cerebral cortex, donor2
UBERON:0001017	Central Nervous System	UBERON:0001017	central nervous system	CNhs11384	Neural stem cells, donor2
UBERON:0001017	Central Nervous System	UBERON:0001017	central nervous system	CNhs12004	Smooth Muscle Cells - Brain Vascular, donor3
UBERON:0001017	Central Nervous System	UBERON:0001017	central nervous system	CNhs12005	Astrocyte - cerebral cortex, donor3
UBERON:0001017	Central Nervous System	UBERON:0001017	central nervous system	CNhs11319	Fibroblast - Choroid Plexus, donor1
UBERON:0001017	Central Nervous System	UBERON:0001017	central nervous system	CNhs12586	Oligodendrocyte - precursors, donor1
UBERON:0001017	Central Nervous System	UBERON:0001017	central nervous system	CNhs11321	Astrocyte - cerebellum, donor1
UBERON:0001017	Central Nervous System	UBERON:0001017	central nervous system	CNhs12344	Fibroblast - Choroid Plexus, donor2
UBERON:0001017	Central Nervous System	UBERON:0001017	central nervous system	CNhs12081	Astrocyte - cerebellum, donor2
UBERON:0001017	Central Nervous System	UBERON:0001017	central nervous system	CNhs12620	Fibroblast - Choroid Plexus, donor3
UBERON:0001017	Central Nervous System	UBERON:0001017	central nervous system	CNhs12117	Astrocyte - cerebellum, donor3
UBERON:0001017	Central Nervous System	UBERON:0001018	axon tract	CNhs10649	corpus callosum, adult, pool1
UBERON:0001017	Central Nervous System	UBERON:0001020	commissure	CNhs10649	corpus callosum, adult, pool1
UBERON:0001017	Central Nervous System	UBERON:0001869	cerebral hemisphere	CNhs10637	temporal lobe, adult, pool1
UBERON:0001017	Central Nervous System	UBERON:0001869	cerebral hemisphere	CNhs10638	postcentral gyrus, adult, pool1
UBERON:0001017	Central Nervous System	UBERON:0001869	cerebral hemisphere	CNhs10641	parietal lobe, adult, pool1
UBERON:0001017	Central Nervous System	UBERON:0001869	cerebral hemisphere	CNhs10642	paracentral gyrus, adult, pool1
UBERON:0001017	Central Nervous System	UBERON:0001869	cerebral hemisphere	CNhs10643	occipital pole, adult, pool1
UBERON:0001017	Central Nervous System	UBERON:0001869	cerebral hemisphere	CNhs10644	nucleus accumbens, adult, pool1
UBERON:0001017	Central Nervous System	UBERON:0001869	cerebral hemisphere	CNhs10646	insula, adult, pool1
UBERON:0001017	Central Nervous System	UBERON:0001869	cerebral hemisphere	CNhs10647	frontal lobe, adult, pool1
UBERON:0001017	Central Nervous System	UBERON:0001869	cerebral hemisphere	Cnhs11781	duodenum, fetal, donor1
UBERON:0001017	Central Nervous System	UBERON:0001869	cerebral hemisphere	CNhs11782	parietal lobe, fetal, donor1
UBERON:0001017	Central Nervous System	UBERON:0001869	cerebral hemisphere	CNhs11784	occipital lobe, fetal, donor1
UBERON:0001017	Central Nervous System	UBERON:0001869	cerebral hemisphere	CNhs11787	occipital lobe, adult, donor1
UBERON:0001017	Central Nervous System	UBERON:0001869	cerebral hemisphere	CNhs12310	medial frontal gyrus, adult, donor10252

UBERON:0001017	Central Nervous System	UBERON:0001869	cerebral hemisphere	CNhs12311	amygdala, adult, donor10252
UBERON:0001017	Central Nervous System	UBERON:0001869	cerebral hemisphere	CNhs13912	putamen, adult, donor10252
UBERON:0001017	Central Nervous System	UBERON:0001869	cerebral hemisphere	CNhs12312	hippocampus, adult, donor10252
UBERON:0001017	Central Nervous System	UBERON:0001869	cerebral hemisphere	CNhs12316	medial temporal gyrus, adult, donor10252
UBERON:0001017	Central Nervous System	UBERON:0001869	cerebral hemisphere	CNhs12317	parietal lobe, adult, donor10252
UBERON:0001017	Central Nervous System	UBERON:0001869	cerebral hemisphere	CNhs12319	globus pallidus, adult, donor10252
UBERON:0001017	Central Nervous System	UBERON:0001869	cerebral hemisphere	CNhs12320	occipital cortex, adult, donor10252
UBERON:0001017	Central Nervous System	UBERON:0001869	cerebral hemisphere	CNhs12321	caudate nucleus, adult, donor10252
UBERON:0001017	Central Nervous System	UBERON:0001869	cerebral hemisphere	CNhs13793	amygdala - adult, donor10196
UBERON:0001017	Central Nervous System	UBERON:0001869	cerebral hemisphere	CNhs13795	hippocampus - adult, donor10196
UBERON:0001017	Central Nervous System	UBERON:0001869	cerebral hemisphere	CNhs13796	medial frontal gyrus - adult, donor10196
UBERON:0001017	Central Nervous System	UBERON:0001869	cerebral hemisphere	CNhs13797	parietal lobe - adult, donor10196
UBERON:0001017	Central Nervous System	UBERON:0001869	cerebral hemisphere	CNhs13798	occipital cortex - adult, donor10196
UBERON:0001017	Central Nervous System	UBERON:0001869	cerebral hemisphere	CNhs13801	globus pallidus - adult, donor10196
UBERON:0001017	Central Nervous System	UBERON:0001869	cerebral hemisphere	CNhs12324	putamen, adult, donor10196
UBERON:0001017	Central Nervous System	UBERON:0001869	cerebral hemisphere	CNhs13802	caudate nucleus - adult, donor10196
UBERON:0001017	Central Nervous System	UBERON:0001869	cerebral hemisphere	CNhs13809	medial temporal gyrus - adult, donor10196
UBERON:0001017	Central Nervous System	UBERON:0001869	cerebral hemisphere	CNhs14069	medial frontal gyrus, newborn, donor10223
UBERON:0001017	Central Nervous System	UBERON:0001869	cerebral hemisphere	CNhs14070	medial temporal gyrus, newborn, donor10223
UBERON:0001017	Central Nervous System	UBERON:0001869	cerebral hemisphere	CNhs14071	caudate nucleus, newborn, donor10223
UBERON:0001017	Central Nervous System	UBERON:0001869	cerebral hemisphere	CNhs14073	occipital cortex, newborn, donor10223
UBERON:0001017	Central Nervous System	UBERON:0001869	cerebral hemisphere	CNhs14074	parietal lobe, newborn, donor10223
UBERON:0001017	Central Nervous System	UBERON:0001869	cerebral hemisphere	CNhs14078	amygdala, newborn, donor10223
UBERON:0001017	Central Nervous System	UBERON:0001869	cerebral hemisphere	CNhs14081	hippocampus, newborn, donor10223
UBERON:0001017	Central Nervous System	UBERON:0001869	cerebral hemisphere	CNhs14082	globus pallidus, newborn, donor10223
UBERON:0001017	Central Nervous System	UBERON:0001869	cerebral hemisphere	CNhs14083	putamen, newborn, donor10223
UBERON:0001017	Central Nervous System	UBERON:0001869	cerebral hemisphere	CNhs14549	globus pallidus, adult, donor10258
UBERON:0001017	Central Nervous System	UBERON:0001869	cerebral hemisphere	CNhs14221	medial frontal gyrus, adult, donor10258
UBERON:0001017	Central Nervous System	UBERON:0001869	cerebral hemisphere	Cnhs14225	putamen, adult, donor10258
UBERON:0001017	Central Nervous System	UBERON:0001869	cerebral hemisphere	CNhs14226	parietal cortex, adult, donor10258
UBERON:0001017	Central Nervous System	UBERON:0001869	cerebral hemisphere	CNhs14227	hippocampus, adult, donor10258
UBERON:0001017	Central Nervous System	UBERON:0001869	cerebral hemisphere	Cnhs14229	medial temporal gyrus, adult, donor10258
UBERON:0001017	Central Nervous System	UBERON:0001869	cerebral hemisphere	CNhs10864	Astrocyte - cerebral cortex, donor1
UBERON:0001017	Central Nervous System	UBERON:0001869	cerebral hemisphere	CNhs11960	Astrocyte - cerebral cortex, donor2
UBERON:0001017	Central Nervous System	UBERON:0001869	cerebral hemisphere	CNhs12005	Astrocyte - cerebral cortex, donor3
UBERON:0001017	Central Nervous System	UBERON:0001870	frontal cortex	CNhs10647	frontal lobe, adult, pool1
UBERON:0001017	Central Nervous System	UBERON:0001870	frontal cortex	CNhs12310	medial frontal gyrus, adult, donor10252
UBERON:0001017	Central Nervous System	UBERON:0001870	frontal cortex	CNhs13796	medial frontal gyrus - adult, donor10196
UBERON:0001017	Central Nervous System	UBERON:0001870	frontal cortex	CNhs14069	medial frontal gyrus, newborn, donor10223
UBERON:0001017	Central Nervous System	UBERON:0001870	frontal cortex	CNhs14221	medial frontal gyrus, adult, donor10258
UBERON:0001017	Central Nervous System	UBERON:0001871	temporal lobe	CNhs10637	temporal lobe, adult, pool1
UBERON:0001017	Central Nervous System	UBERON:0001871	temporal lobe	Cnhs11781	duodenum, fetal, donor1
UBERON:0001017	Central Nervous System	UBERON:0001871	temporal lobe	CNhs12311	amygdala, adult, donor10252
UBERON:0001017	Central Nervous System	UBERON:0001871	temporal lobe	CNhs12316	medial temporal gyrus, adult, donor10252
UBERON:0001017	Central Nervous System	UBERON:0001871	temporal lobe	CNhs13793	amygdala - adult, donor10196
UBERON:0001017	Central Nervous System	UBERON:0001871	temporal lobe	CNhs13809	medial temporal gyrus - adult, donor10196
UBERON:0001017	Central Nervous System	UBERON:0001871	temporal lobe	CNhs14070	medial temporal gyrus, newborn, donor10223
UBERON:0001017	Central Nervous System	UBERON:0001871	temporal lobe	CNhs14078	amygdala, newborn, donor10223
UBERON:0001017	Central Nervous System	UBERON:0001871	temporal lobe	Cnhs14229	medial temporal gyrus, adult, donor10258
UBERON:0001017	Central Nervous System	UBERON:0001872	parietal lobe	CNhs10638	postcentral gyrus, adult, pool1
UBERON:0001017	Central Nervous System	UBERON:0001872	parietal lobe	CNhs10641	parietal lobe, adult, pool1
UBERON:0001017	Central Nervous System	UBERON:0001872	parietal lobe	CNhs11782	parietal lobe, fetal, donor1
UBERON:0001017	Central Nervous System	UBERON:0001872	parietal lobe	CNhs12317	parietal lobe, adult, donor10252
UBERON:0001017	Central Nervous System	UBERON:0001872	parietal lobe	CNhs13797	parietal lobe - adult, donor10196
UBERON:0001017	Central Nervous System	UBERON:0001872	parietal lobe	CNhs14074	parietal lobe, newborn, donor10223
UBERON:0001017	Central Nervous System	UBERON:0001872	parietal lobe	CNhs14226	parietal cortex, adult, donor10258
UBERON:0001017	Central Nervous System	UBERON:0001873	caudate nucleus	CNhs12321	caudate nucleus, adult, donor10252
UBERON:0001017	Central Nervous System	UBERON:0001873	caudate nucleus	CNhs13802	caudate nucleus - adult, donor10196
UBERON:0001017	Central Nervous System	UBERON:0001873	caudate nucleus	CNhs14071	caudate nucleus, newborn, donor10223
UBERON:0001017	Central Nervous System	UBERON:0001874	putamen	CNhs13912	putamen, adult, donor10252
UBERON:0001017	Central Nervous System	UBERON:0001874	putamen	CNhs12324	putamen, adult, donor10196
UBERON:0001017	Central Nervous System	UBERON:0001874	putamen	CNhs14083	putamen, newborn, donor10223
UBERON:0001017	Central Nervous System	UBERON:0001874	putamen	Cnhs14225	putamen, adult, donor10258
UBERON:0001017	Central Nervous System	UBERON:0001875	globus pallidus	CNhs12319	globus pallidus, adult, donor10252
UBERON:0001017	Central Nervous System	UBERON:0001875	globus pallidus	CNhs13801	globus pallidus - adult, donor10196
UBERON:0001017	Central Nervous System	UBERON:0001875	globus pallidus	CNhs14082	globus pallidus, newborn, donor10223
UBERON:0001017	Central Nervous System	UBERON:0001875	globus pallidus	CNhs14549	globus pallidus, adult, donor10258
UBERON:0001017	Central Nervous System	UBERON:0001876	amygdala	CNhs12311	amygdala, adult, donor10252
UBERON:0001017	Central Nervous System	UBERON:0001876	amygdala	CNhs13793	amygdala - adult, donor10196
UBERON:0001017	Central Nervous System	UBERON:0001876	amygdala	CNhs14078	amygdala, newborn, donor10223
UBERON:0001017	Central Nervous System	UBERON:0001882	nucleus accumbens	CNhs10644	nucleus accumbens, adult, pool1
UBERON:0001017	Central Nervous System	UBERON:0001886	choroid plexus	CNhs11319	Fibroblast - Choroid Plexus, donor1
UBERON:0001017	Central Nervous System	UBERON:0001886	choroid plexus	CNhs12344	Fibroblast - Choroid Plexus, donor2
UBERON:0001017	Central Nervous System	UBERON:0001886	choroid plexus	CNhs12620	Fibroblast - Choroid Plexus, donor3
UBERON:0001017	Central Nervous System	UBERON:0001890	forebrain	CNhs10637	temporal lobe, adult, pool1
UBERON:0001017	Central Nervous System	UBERON:0001890	forebrain	CNhs10638	postcentral gyrus, adult, pool1
UBERON:0001017	Central Nervous System	UBERON:0001890	forebrain	CNhs10641	parietal lobe, adult, pool1
UBERON:0001017	Central Nervous System	UBERON:0001890	forebrain	CNhs10642	paracentral gyrus, adult, pool1
UBERON:0001017	Central Nervous System	UBERON:0001890	forebrain	CNhs10643	occipital pole, adult, pool1
UBERON:0001017	Central Nervous System	UBERON:0001890	forebrain	CNhs10644	nucleus accumbens, adult, pool1
UBERON:0001017	Central Nervous System	UBERON:0001890	forebrain	CNhs10646	insula, adult, pool1
UBERON:0001017	Central Nervous System	UBERON:0001890	forebrain	CNhs10647	frontal lobe, adult, pool1
UBERON:0001017	Central Nervous System	UBERON:0001890	forebrain	CNhs10649	corpus callosum, adult, pool1
UBERON:0001017	Central Nervous System	UBERON:0001890	forebrain	Cnhs11781	duodenum, fetal, donor1
UBERON:0001017	Central Nervous System	UBERON:0001890	forebrain	CNhs11782	parietal lobe, fetal, donor1
UBERON:0001017	Central Nervous System	UBERON:0001890	forebrain	CNhs11784	occipital lobe, fetal, donor1
UBERON:0001017	Central Nervous System	UBERON:0001890	forebrain	CNhs11787	occipital lobe, adult, donor1
UBERON:0001017	Central Nervous System	UBERON:0001890	forebrain	CNhs12310	medial frontal gyrus, adult, donor10252
UBERON:0001017	Central Nervous System	UBERON:0001890	forebrain	CNhs12311	amygdala, adult, donor10252
UBERON:0001017	Central Nervous System	UBERON:0001890	forebrain	CNhs13912	putamen, adult, donor10252
UBERON:0001017	Central Nervous System	UBERON:0001890	forebrain	CNhs12312	hippocampus, adult, donor10252
UBERON:0001017	Central Nervous System	UBERON:0001890	forebrain	CNhs12314	thalamus, adult, donor10252
UBERON:0001017	Central Nervous System	UBERON:0001890	forebrain	CNhs12316	medial temporal gyrus, adult, donor10252
UBERON:0001017	Central Nervous System	UBERON:0001890	forebrain	CNhs12317	parietal lobe, adult, donor10252
UBERON:0001017	Central Nervous System	UBERON:0001890	forebrain	CNhs12318	substantia nigra, adult, donor10252
UBERON:0001017	Central Nervous System	UBERON:0001890	forebrain	CNhs12228	pineal gland, adult, donor10252
UBERON:0001017	Central Nervous System	UBERON:0001890	forebrain	CNhs12319	globus pallidus, adult, donor10252
UBERON:0001017	Central Nervous System	UBERON:0001890	forebrain	CNhs12229	pituitary gland, adult, donor10252

UBERON:0001017	Central Nervous System	UBERON:0001890	forebrain	CNhs12320	occipital cortex, adult, donor10252
UBERON:0001017	Central Nervous System	UBERON:0001890	forebrain	CNhs12321	caudate nucleus, adult, donor10252
UBERON:0001017	Central Nervous System	UBERON:0001890	forebrain	CNhs13793	amygdala - adult, donor10196
UBERON:0001017	Central Nervous System	UBERON:0001890	forebrain	CNhs13794	thalamus - adult, donor10196
UBERON:0001017	Central Nervous System	UBERON:0001890	forebrain	CNhs13795	hippocampus - adult, donor10196
UBERON:0001017	Central Nervous System	UBERON:0001890	forebrain	CNhs13796	medial frontal gyrus - adult, donor10196
UBERON:0001017	Central Nervous System	UBERON:0001890	forebrain	CNhs13797	parietal lobe - adult, donor10196
UBERON:0001017	Central Nervous System	UBERON:0001890	forebrain	CNhs13798	occipital cortex - adult, donor10196
UBERON:0001017	Central Nervous System	UBERON:0001890	forebrain	CNhs13801	globus pallidus - adult, donor10196
UBERON:0001017	Central Nervous System	UBERON:0001890	forebrain	CNhs12324	putamen, adult, donor10196
UBERON:0001017	Central Nervous System	UBERON:0001890	forebrain	CNhs13802	caudate nucleus - adult, donor10196
UBERON:0001017	Central Nervous System	UBERON:0001890	forebrain	CNhs13803	substantia nigra - adult, donor10196
UBERON:0001017	Central Nervous System	UBERON:0001890	forebrain	CNhs13804	pineal gland - adult, donor10196
UBERON:0001017	Central Nervous System	UBERON:0001890	forebrain	CNhs13805	pituitary gland - adult, donor10196
UBERON:0001017	Central Nervous System	UBERON:0001890	forebrain	CNhs13809	medial temporal gyrus - adult, donor10196
UBERON:0001017	Central Nervous System	UBERON:0001890	forebrain	CNhs12610	diencephalon, adult
UBERON:0001017	Central Nervous System	UBERON:0001890	forebrain	CNhs14069	medial frontal gyrus, newborn, donor10223
UBERON:0001017	Central Nervous System	UBERON:0001890	forebrain	CNhs14070	medial temporal gyrus, newborn, donor10223
UBERON:0001017	Central Nervous System	UBERON:0001890	forebrain	CNhs14071	caudate nucleus, newborn, donor10223
UBERON:0001017	Central Nervous System	UBERON:0001890	forebrain	CNhs14073	occipital cortex, newborn, donor10223
UBERON:0001017	Central Nervous System	UBERON:0001890	forebrain	CNhs14074	parietal lobe, newborn, donor10223
UBERON:0001017	Central Nervous System	UBERON:0001890	forebrain	CNhs14076	substantia nigra, newborn, donor10223
UBERON:0001017	Central Nervous System	UBERON:0001890	forebrain	CNhs14078	amygdala, newborn, donor10223
UBERON:0001017	Central Nervous System	UBERON:0001890	forebrain	CNhs14081	hippocampus, newborn, donor10223
UBERON:0001017	Central Nervous System	UBERON:0001890	forebrain	CNhs14082	globus pallidus, newborn, donor10223
UBERON:0001017	Central Nervous System	UBERON:0001890	forebrain	CNhs14083	putamen, newborn, donor10223
UBERON:0001017	Central Nervous System	UBERON:0001890	forebrain	CNhs14084	thalamus, newborn, donor10223
UBERON:0001017	Central Nervous System	UBERON:0001890	forebrain	CNhs14549	globus pallidus, adult, donor10258
UBERON:0001017	Central Nervous System	UBERON:0001890	forebrain	CNhs14221	medial frontal gyrus, adult, donor10258
UBERON:0001017	Central Nervous System	UBERON:0001890	forebrain	Cnhs14223	thalamus, adult, donor10258
UBERON:0001017	Central Nervous System	UBERON:0001890	forebrain	CNhs14224	substantia nigra, adult, donor10258
UBERON:0001017	Central Nervous System	UBERON:0001890	forebrain	Cnhs14225	putamen, adult, donor10258
UBERON:0001017	Central Nervous System	UBERON:0001890	forebrain	CNhs14226	parietal cortex, adult, donor10258
UBERON:0001017	Central Nervous System	UBERON:0001890	forebrain	CNhs14227	hippocampus, adult, donor10258
UBERON:0001017	Central Nervous System	UBERON:0001890	forebrain	Cnhs14229	medial temporal gyrus, adult, donor10258
UBERON:0001017	Central Nervous System	UBERON:0001890	forebrain	CNhs14230	pineal gland, adult, donor10258
UBERON:0001017	Central Nervous System	UBERON:0001890	forebrain	CNhs14231	pituitary gland, adult, donor10258
UBERON:0001017	Central Nervous System	UBERON:0001890	forebrain	CNhs10864	Astrocyte - cerebral cortex, donor1
UBERON:0001017	Central Nervous System	UBERON:0001890	forebrain	CNhs11960	Astrocyte - cerebral cortex, donor2
UBERON:0001017	Central Nervous System	UBERON:0001890	forebrain	CNhs12005	Astrocyte - cerebral cortex, donor3
UBERON:0001017	Central Nervous System	UBERON:0001891	midbrain	CNhs12318	substantia nigra, adult, donor10252
UBERON:0001017	Central Nervous System	UBERON:0001891	midbrain	CNhs13803	substantia nigra - adult, donor10196
UBERON:0001017	Central Nervous System	UBERON:0001891	midbrain	CNhs14076	substantia nigra, newborn, donor10223
UBERON:0001017	Central Nervous System	UBERON:0001891	midbrain	CNhs14224	substantia nigra, adult, donor10258
UBERON:0001017	Central Nervous System	UBERON:0001893	telencephalon	CNhs10637	temporal lobe, adult, pool1
UBERON:0001017	Central Nervous System	UBERON:0001893	telencephalon	CNhs10638	postcentral gyrus, adult, pool1
UBERON:0001017	Central Nervous System	UBERON:0001893	telencephalon	CNhs10641	parietal lobe, adult, pool1
UBERON:0001017	Central Nervous System	UBERON:0001893	telencephalon	CNhs10642	paracentral gyrus, adult, pool1
UBERON:0001017	Central Nervous System	UBERON:0001893	telencephalon	CNhs10643	occipital pole, adult, pool1
UBERON:0001017	Central Nervous System	UBERON:0001893	telencephalon	CNhs10644	nucleus accumbens, adult, pool1
UBERON:0001017	Central Nervous System	UBERON:0001893	telencephalon	CNhs10646	insula, adult, pool1
UBERON:0001017	Central Nervous System	UBERON:0001893	telencephalon	CNhs10647	frontal lobe, adult, pool1
UBERON:0001017	Central Nervous System	UBERON:0001893	telencephalon	CNhs10649	corpus callosum, adult, pool1
UBERON:0001017	Central Nervous System	UBERON:0001893	telencephalon	Cnhs11781	duodenum, fetal, donor1
UBERON:0001017	Central Nervous System	UBERON:0001893	telencephalon	CNhs11782	parietal lobe, fetal, donor1
UBERON:0001017	Central Nervous System	UBERON:0001893	telencephalon	CNhs11784	occipital lobe, fetal, donor1
UBERON:0001017	Central Nervous System	UBERON:0001893	telencephalon	CNhs11787	occipital lobe, adult, donor1
UBERON:0001017	Central Nervous System	UBERON:0001893	telencephalon	CNhs12310	medial frontal gyrus, adult, donor10252
UBERON:0001017	Central Nervous System	UBERON:0001893	telencephalon	CNhs12311	amygdala, adult, donor10252
UBERON:0001017	Central Nervous System	UBERON:0001893	telencephalon	CNhs13912	putamen, adult, donor10252
UBERON:0001017	Central Nervous System	UBERON:0001893	telencephalon	CNhs12312	hippocampus, adult, donor10252
UBERON:0001017	Central Nervous System	UBERON:0001893	telencephalon	CNhs12316	medial temporal gyrus, adult, donor10252
UBERON:0001017	Central Nervous System	UBERON:0001893	telencephalon	CNhs12317	parietal lobe, adult, donor10252
UBERON:0001017	Central Nervous System	UBERON:0001893	telencephalon	CNhs12318	substantia nigra, adult, donor10252
UBERON:0001017	Central Nervous System	UBERON:0001893	telencephalon	CNhs12319	globus pallidus, adult, donor10252
UBERON:0001017	Central Nervous System	UBERON:0001893	telencephalon	CNhs12320	occipital cortex, adult, donor10252
UBERON:0001017	Central Nervous System	UBERON:0001893	telencephalon	CNhs12321	caudate nucleus, adult, donor10252
UBERON:0001017	Central Nervous System	UBERON:0001893	telencephalon	CNhs13793	amygdala - adult, donor10196
UBERON:0001017	Central Nervous System	UBERON:0001893	telencephalon	CNhs13795	hippocampus - adult, donor10196
UBERON:0001017	Central Nervous System	UBERON:0001893	telencephalon	CNhs13796	medial frontal gyrus - adult, donor10196
UBERON:0001017	Central Nervous System	UBERON:0001893	telencephalon	CNhs13797	parietal lobe - adult, donor10196
UBERON:0001017	Central Nervous System	UBERON:0001893	telencephalon	CNhs13798	occipital cortex - adult, donor10196
UBERON:0001017	Central Nervous System	UBERON:0001893	telencephalon	CNhs13801	globus pallidus - adult, donor10196
UBERON:0001017	Central Nervous System	UBERON:0001893	telencephalon	CNhs12324	putamen, adult, donor10196
UBERON:0001017	Central Nervous System	UBERON:0001893	telencephalon	CNhs13802	caudate nucleus - adult, donor10196
UBERON:0001017	Central Nervous System	UBERON:0001893	telencephalon	CNhs13803	substantia nigra - adult, donor10196
UBERON:0001017	Central Nervous System	UBERON:0001893	telencephalon	CNhs13809	medial temporal gyrus - adult, donor10196
UBERON:0001017	Central Nervous System	UBERON:0001893	telencephalon	CNhs14069	medial frontal gyrus, newborn, donor10223
UBERON:0001017	Central Nervous System	UBERON:0001893	telencephalon	CNhs14070	medial temporal gyrus, newborn, donor10223
UBERON:0001017	Central Nervous System	UBERON:0001893	telencephalon	CNhs14071	caudate nucleus, newborn, donor10223
UBERON:0001017	Central Nervous System	UBERON:0001893	telencephalon	CNhs14073	occipital cortex, newborn, donor10223
UBERON:0001017	Central Nervous System	UBERON:0001893	telencephalon	CNhs14074	parietal lobe, newborn, donor10223
UBERON:0001017	Central Nervous System	UBERON:0001893	telencephalon	CNhs14076	substantia nigra, newborn, donor10223
UBERON:0001017	Central Nervous System	UBERON:0001893	telencephalon	CNhs14078	amygdala, newborn, donor10223
UBERON:0001017	Central Nervous System	UBERON:0001893	telencephalon	CNhs14081	hippocampus, newborn, donor10223
UBERON:0001017	Central Nervous System	UBERON:0001893	telencephalon	CNhs14082	globus pallidus, newborn, donor10223
UBERON:0001017	Central Nervous System	UBERON:0001893	telencephalon	CNhs14083	putamen, newborn, donor10223
UBERON:0001017	Central Nervous System	UBERON:0001893	telencephalon	CNhs14549	globus pallidus, adult, donor10258
UBERON:0001017	Central Nervous System	UBERON:0001893	telencephalon	CNhs14221	medial frontal gyrus, adult, donor10258
UBERON:0001017	Central Nervous System	UBERON:0001893	telencephalon	CNhs14224	substantia nigra, adult, donor10258
UBERON:0001017	Central Nervous System	UBERON:0001893	telencephalon	Cnhs14225	putamen, adult, donor10258
UBERON:0001017	Central Nervous System	UBERON:0001893	telencephalon	CNhs14226	parietal cortex, adult, donor10258
UBERON:0001017	Central Nervous System	UBERON:0001893	telencephalon	CNhs14227	hippocampus, adult, donor10258
UBERON:0001017	Central Nervous System	UBERON:0001893	telencephalon	Cnhs14229	medial temporal gyrus, adult, donor10258
UBERON:0001017	Central Nervous System	UBERON:0001893	telencephalon	CNhs10864	Astrocyte - cerebral cortex, donor1
UBERON:0001017	Central Nervous System	UBERON:0001893	telencephalon	CNhs11960	Astrocyte - cerebral cortex, donor2
UBERON:0001017	Central Nervous System	UBERON:0001893	telencephalon	CNhs12005	Astrocyte - cerebral cortex, donor3
UBERON:0001017	Central Nervous System	UBERON:0001894	diencephalon	CNhs12314	thalamus, adult, donor10252

UBERON:0001017	Central Nervous System	UBERON:0001894	diencephalon	CNhs12228	pineal gland, adult, donor10252
UBERON:0001017	Central Nervous System	UBERON:0001894	diencephalon	CNhs12229	pituitary gland, adult, donor10252
UBERON:0001017	Central Nervous System	UBERON:0001894	diencephalon	CNhs13794	thalamus - adult, donor10196
UBERON:0001017	Central Nervous System	UBERON:0001894	diencephalon	CNhs13804	pineal gland - adult, donor10196
UBERON:0001017	Central Nervous System	UBERON:0001894	diencephalon	CNhs13805	pituitary gland - adult, donor10196
UBERON:0001017	Central Nervous System	UBERON:0001894	diencephalon	CNhs12610	diencephalon, adult
UBERON:0001017	Central Nervous System	UBERON:0001894	diencephalon	CNhs14084	thalamus, newborn, donor10223
UBERON:0001017	Central Nervous System	UBERON:0001894	diencephalon	Cnhs14223	thalamus, adult, donor10258
UBERON:0001017	Central Nervous System	UBERON:0001894	diencephalon	CNhs14230	pineal gland, adult, donor10258
UBERON:0001017	Central Nervous System	UBERON:0001894	diencephalon	CNhs14231	pituitary gland, adult, donor10258
UBERON:0001017	Central Nervous System	UBERON:0001895	metencephalon	CNhs10640	pons, adult, pool1
UBERON:0001017	Central Nervous System	UBERON:0001895	metencephalon	CNhs11795	cerebellum, adult, pool1
UBERON:0001017	Central Nervous System	UBERON:0001895	metencephalon	CNhs12322	locus coeruleus, adult, donor10252
UBERON:0001017	Central Nervous System	UBERON:0001895	metencephalon	CNhs12323	cerebellum, adult, donor10252
UBERON:0001017	Central Nervous System	UBERON:0001895	metencephalon	CNhs13799	cerebellum - adult, donor10196
UBERON:0001017	Central Nervous System	UBERON:0001895	metencephalon	CNhs13808	locus coeruleus - adult, donor10196
UBERON:0001017	Central Nervous System	UBERON:0001895	metencephalon	CNhs14075	cerebellum, newborn, donor10223
UBERON:0001017	Central Nervous System	UBERON:0001895	metencephalon	CNhs14080	locus coeruleus, newborn, donor10223
UBERON:0001017	Central Nervous System	UBERON:0001895	metencephalon	CNhs14550	locus coeruleus, adult, donor10258
UBERON:0001017	Central Nervous System	UBERON:0001895	metencephalon	CNhs11321	Astrocyte - cerebellum, donor1
UBERON:0001017	Central Nervous System	UBERON:0001895	metencephalon	CNhs12081	Astrocyte - cerebellum, donor2
UBERON:0001017	Central Nervous System	UBERON:0001895	metencephalon	CNhs12117	Astrocyte - cerebellum, donor3
UBERON:0001017	Central Nervous System	UBERON:0001896	medulla oblongata	CNhs10645	medulla oblongata, adult, pool1
UBERON:0001017	Central Nervous System	UBERON:0001896	medulla oblongata	CNhs12315	medulla oblongata, adult, donor10252
UBERON:0001017	Central Nervous System	UBERON:0001896	medulla oblongata	CNhs13800	medulla oblongata - adult, donor10196
UBERON:0001017	Central Nervous System	UBERON:0001896	medulla oblongata	CNhs14079	medulla oblongata, newborn, donor10223
UBERON:0001017	Central Nervous System	UBERON:0001897	thalamus	CNhs12314	thalamus, adult, donor10252
UBERON:0001017	Central Nervous System	UBERON:0001897	thalamus	CNhs13794	thalamus - adult, donor10196
UBERON:0001017	Central Nervous System	UBERON:0001897	thalamus	CNhs14084	thalamus, newborn, donor10223
UBERON:0001017	Central Nervous System	UBERON:0001897	thalamus	Cnhs14223	thalamus, adult, donor10258
UBERON:0001017	Central Nervous System	UBERON:0001899	epithalamus	CNhs12228	pineal gland, adult, donor10252
UBERON:0001017	Central Nervous System	UBERON:0001899	epithalamus	CNhs13804	pineal gland - adult, donor10196
UBERON:0001017	Central Nervous System	UBERON:0001899	epithalamus	CNhs14230	pineal gland, adult, donor10258
UBERON:0001017	Central Nervous System	UBERON:0001905	pineal body	CNhs12228	pineal gland, adult, donor10252
UBERON:0001017	Central Nervous System	UBERON:0001905	pineal body	CNhs13804	pineal gland - adult, donor10196
UBERON:0001017	Central Nervous System	UBERON:0001905	pineal body	CNhs14230	pineal gland, adult, donor10258
UBERON:0001017	Central Nervous System	UBERON:0001950	neocortex	CNhs10637	temporal lobe, adult, pool1
UBERON:0001017	Central Nervous System	UBERON:0001950	neocortex	CNhs10638	postcentral gyrus, adult, pool1
UBERON:0001017	Central Nervous System	UBERON:0001950	neocortex	CNhs10641	parietal lobe, adult, pool1
UBERON:0001017	Central Nervous System	UBERON:0001950	neocortex	CNhs10643	occipital pole, adult, pool1
UBERON:0001017	Central Nervous System	UBERON:0001950	neocortex	CNhs10646	insula, adult, pool1
UBERON:0001017	Central Nervous System	UBERON:0001950	neocortex	CNhs10647	frontal lobe, adult, pool1
UBERON:0001017	Central Nervous System	UBERON:0001950	neocortex	Cnhs11781	duodenum, fetal, donor1
UBERON:0001017	Central Nervous System	UBERON:0001950	neocortex	CNhs11782	parietal lobe, fetal, donor1
UBERON:0001017	Central Nervous System	UBERON:0001950	neocortex	CNhs11784	occipital lobe, fetal, donor1
UBERON:0001017	Central Nervous System	UBERON:0001950	neocortex	CNhs11787	occipital lobe, adult, donor1
UBERON:0001017	Central Nervous System	UBERON:0001950	neocortex	CNhs12310	medial frontal gyrus, adult, donor10252
UBERON:0001017	Central Nervous System	UBERON:0001950	neocortex	CNhs12311	amygdala, adult, donor10252
UBERON:0001017	Central Nervous System	UBERON:0001950	neocortex	CNhs12316	medial temporal gyrus, adult, donor10252
UBERON:0001017	Central Nervous System	UBERON:0001950	neocortex	CNhs12317	parietal lobe, adult, donor10252
UBERON:0001017	Central Nervous System	UBERON:0001950	neocortex	CNhs12320	occipital cortex, adult, donor10252
UBERON:0001017	Central Nervous System	UBERON:0001950	neocortex	CNhs13793	amygdala - adult, donor10196
UBERON:0001017	Central Nervous System	UBERON:0001950	neocortex	CNhs13796	medial frontal gyrus - adult, donor10196
UBERON:0001017	Central Nervous System	UBERON:0001950	neocortex	CNhs13797	parietal lobe - adult, donor10196
UBERON:0001017	Central Nervous System	UBERON:0001950	neocortex	CNhs13798	occipital cortex - adult, donor10196
UBERON:0001017	Central Nervous System	UBERON:0001950	neocortex	CNhs13809	medial temporal gyrus - adult, donor10196
UBERON:0001017	Central Nervous System	UBERON:0001950	neocortex	CNhs14069	medial frontal gyrus, newborn, donor10223
UBERON:0001017	Central Nervous System	UBERON:0001950	neocortex	CNhs14070	medial temporal gyrus, newborn, donor10223
UBERON:0001017	Central Nervous System	UBERON:0001950	neocortex	CNhs14073	occipital cortex, newborn, donor10223
UBERON:0001017	Central Nervous System	UBERON:0001950	neocortex	CNhs14074	parietal lobe, newborn, donor10223
UBERON:0001017	Central Nervous System	UBERON:0001950	neocortex	CNhs14078	amygdala, newborn, donor10223
UBERON:0001017	Central Nervous System	UBERON:0001950	neocortex	CNhs14221	medial frontal gyrus, adult, donor10258
UBERON:0001017	Central Nervous System	UBERON:0001950	neocortex	CNhs14226	parietal cortex, adult, donor10258
UBERON:0001017	Central Nervous System	UBERON:0001950	neocortex	Cnhs14229	medial temporal gyrus, adult, donor10258
UBERON:0001017	Central Nervous System	UBERON:0001954	Ammon's horn	CNhs12312	hippocampus, adult, donor10252
UBERON:0001017	Central Nervous System	UBERON:0001954	Ammon's horn	CNhs13795	hippocampus - adult, donor10196
UBERON:0001017	Central Nervous System	UBERON:0001954	Ammon's horn	CNhs14081	hippocampus, newborn, donor10223
UBERON:0001017	Central Nervous System	UBERON:0001954	Ammon's horn	CNhs14227	hippocampus, adult, donor10258
UBERON:0001017	Central Nervous System	UBERON:0002020	gray matter of neuraxis	CNhs10637	temporal lobe, adult, pool1
UBERON:0001017	Central Nervous System	UBERON:0002020	gray matter of neuraxis	CNhs10638	postcentral gyrus, adult, pool1
UBERON:0001017	Central Nervous System	UBERON:0002020	gray matter of neuraxis	CNhs10641	parietal lobe, adult, pool1
UBERON:0001017	Central Nervous System	UBERON:0002020	gray matter of neuraxis	CNhs10643	occipital pole, adult, pool1
UBERON:0001017	Central Nervous System	UBERON:0002020	gray matter of neuraxis	CNhs10644	nucleus accumbens, adult, pool1
UBERON:0001017	Central Nervous System	UBERON:0002020	gray matter of neuraxis	CNhs10646	insula, adult, pool1
UBERON:0001017	Central Nervous System	UBERON:0002020	gray matter of neuraxis	CNhs10647	frontal lobe, adult, pool1
UBERON:0001017	Central Nervous System	UBERON:0002020	gray matter of neuraxis	Cnhs11781	duodenum, fetal, donor1
UBERON:0001017	Central Nervous System	UBERON:0002020	gray matter of neuraxis	CNhs11782	parietal lobe, fetal, donor1
UBERON:0001017	Central Nervous System	UBERON:0002020	gray matter of neuraxis	CNhs11784	occipital lobe, fetal, donor1
UBERON:0001017	Central Nervous System	UBERON:0002020	gray matter of neuraxis	CNhs11787	occipital lobe, adult, donor1
UBERON:0001017	Central Nervous System	UBERON:0002020	gray matter of neuraxis	CNhs12310	medial frontal gyrus, adult, donor10252
UBERON:0001017	Central Nervous System	UBERON:0002020	gray matter of neuraxis	CNhs12311	amygdala, adult, donor10252
UBERON:0001017	Central Nervous System	UBERON:0002020	gray matter of neuraxis	CNhs13912	putamen, adult, donor10252
UBERON:0001017	Central Nervous System	UBERON:0002020	gray matter of neuraxis	CNhs12312	hippocampus, adult, donor10252
UBERON:0001017	Central Nervous System	UBERON:0002020	gray matter of neuraxis	CNhs12316	medial temporal gyrus, adult, donor10252
UBERON:0001017	Central Nervous System	UBERON:0002020	gray matter of neuraxis	CNhs12317	parietal lobe, adult, donor10252
UBERON:0001017	Central Nervous System	UBERON:0002020	gray matter of neuraxis	CNhs12318	substantia nigra, adult, donor10252
UBERON:0001017	Central Nervous System	UBERON:0002020	gray matter of neuraxis	CNhs12319	globus pallidus, adult, donor10252
UBERON:0001017	Central Nervous System	UBERON:0002020	gray matter of neuraxis	CNhs12320	occipital cortex, adult, donor10252
UBERON:0001017	Central Nervous System	UBERON:0002020	gray matter of neuraxis	CNhs12321	caudate nucleus, adult, donor10252
UBERON:0001017	Central Nervous System	UBERON:0002020	gray matter of neuraxis	CNhs12322	locus coeruleus, adult, donor10252
UBERON:0001017	Central Nervous System	UBERON:0002020	gray matter of neuraxis	CNhs13793	amygdala - adult, donor10196
UBERON:0001017	Central Nervous System	UBERON:0002020	gray matter of neuraxis	CNhs13795	hippocampus - adult, donor10196
UBERON:0001017	Central Nervous System	UBERON:0002020	gray matter of neuraxis	CNhs13796	medial frontal gyrus - adult, donor10196
UBERON:0001017	Central Nervous System	UBERON:0002020	gray matter of neuraxis	CNhs13797	parietal lobe - adult, donor10196
UBERON:0001017	Central Nervous System	UBERON:0002020	gray matter of neuraxis	CNhs13798	occipital cortex - adult, donor10196
UBERON:0001017	Central Nervous System	UBERON:0002020	gray matter of neuraxis	CNhs13801	globus pallidus - adult, donor10196
UBERON:0001017	Central Nervous System	UBERON:0002020	gray matter of neuraxis	CNhs12324	putamen, adult, donor10196
UBERON:0001017	Central Nervous System	UBERON:0002020	gray matter of neuraxis	CNhs13802	caudate nucleus - adult, donor10196

UBERON:0001017	Central Nervous System	UBERON:0002020	gray matter of neuraxis	CNhs13803	substantia nigra - adult, donor10196
UBERON:0001017	Central Nervous System	UBERON:0002020	gray matter of neuraxis	CNhs13808	locus coeruleus - adult, donor10196
UBERON:0001017	Central Nervous System	UBERON:0002020	gray matter of neuraxis	CNhs13809	medial temporal gyrus - adult, donor10196
UBERON:0001017	Central Nervous System	UBERON:0002020	gray matter of neuraxis	CNhs14069	medial frontal gyrus, newborn, donor10223
UBERON:0001017	Central Nervous System	UBERON:0002020	gray matter of neuraxis	CNhs14070	medial temporal gyrus, newborn, donor10223
UBERON:0001017	Central Nervous System	UBERON:0002020	gray matter of neuraxis	CNhs14071	caudate nucleus, newborn, donor10223
UBERON:0001017	Central Nervous System	UBERON:0002020	gray matter of neuraxis	CNhs14073	occipital cortex, newborn, donor10223
UBERON:0001017	Central Nervous System	UBERON:0002020	gray matter of neuraxis	CNhs14074	parietal lobe, newborn, donor10223
UBERON:0001017	Central Nervous System	UBERON:0002020	gray matter of neuraxis	CNhs14076	substantia nigra, newborn, donor10223
UBERON:0001017	Central Nervous System	UBERON:0002020	gray matter of neuraxis	CNhs14078	amygdala, newborn, donor10223
UBERON:0001017	Central Nervous System	UBERON:0002020	gray matter of neuraxis	CNhs14080	locus coeruleus, newborn, donor10223
UBERON:0001017	Central Nervous System	UBERON:0002020	gray matter of neuraxis	CNhs14081	hippocampus, newborn, donor10223
UBERON:0001017	Central Nervous System	UBERON:0002020	gray matter of neuraxis	CNhs14082	globus pallidus, newborn, donor10223
UBERON:0001017	Central Nervous System	UBERON:0002020	gray matter of neuraxis	CNhs14083	putamen, newborn, donor10223
UBERON:0001017	Central Nervous System	UBERON:0002020	gray matter of neuraxis	CNhs14549	globus pallidus, adult, donor10258
UBERON:0001017	Central Nervous System	UBERON:0002020	gray matter of neuraxis	CNhs14221	medial frontal gyrus, adult, donor10258
UBERON:0001017	Central Nervous System	UBERON:0002020	gray matter of neuraxis	CNhs14224	substantia nigra, adult, donor10258
UBERON:0001017	Central Nervous System	UBERON:0002020	gray matter of neuraxis	Cnhs14225	putamen, adult, donor10258
UBERON:0001017	Central Nervous System	UBERON:0002020	gray matter of neuraxis	CNhs14226	parietal cortex, adult, donor10258
UBERON:0001017	Central Nervous System	UBERON:0002020	gray matter of neuraxis	CNhs14227	hippocampus, adult, donor10258
UBERON:0001017	Central Nervous System	UBERON:0002020	gray matter of neuraxis	CNhs14550	locus coeruleus, adult, donor10258
UBERON:0001017	Central Nervous System	UBERON:0002020	gray matter of neuraxis	Cnhs14229	medial temporal gyrus, adult, donor10258
UBERON:0001017	Central Nervous System	UBERON:0002020	gray matter of neuraxis	CNhs10864	Astrocyte - cerebral cortex, donor1
UBERON:0001017	Central Nervous System	UBERON:0002020	gray matter of neuraxis	CNhs11960	Astrocyte - cerebral cortex, donor2
UBERON:0001017	Central Nervous System	UBERON:0002020	gray matter of neuraxis	CNhs12005	Astrocyte - cerebral cortex, donor3
UBERON:0001017	Central Nervous System	UBERON:0002021	occipital lobe	CNhs10643	occipital pole, adult, pool1
UBERON:0001017	Central Nervous System	UBERON:0002021	occipital lobe	CNhs11784	occipital lobe, fetal, donor1
UBERON:0001017	Central Nervous System	UBERON:0002021	occipital lobe	CNhs11787	occipital lobe, adult, donor1
UBERON:0001017	Central Nervous System	UBERON:0002021	occipital lobe	CNhs12320	occipital cortex, adult, donor10252
UBERON:0001017	Central Nervous System	UBERON:0002021	occipital lobe	CNhs13798	occipital cortex - adult, donor10196
UBERON:0001017	Central Nervous System	UBERON:0002021	occipital lobe	CNhs14073	occipital cortex, newborn, donor10223
UBERON:0001017	Central Nervous System	UBERON:0002022	insula	CNhs10646	insula, adult, pool1
UBERON:0001017	Central Nervous System	UBERON:0002028	hindbrain	CNhs10640	pons, adult, pool1
UBERON:0001017	Central Nervous System	UBERON:0002028	hindbrain	CNhs10645	medulla oblongata, adult, pool1
UBERON:0001017	Central Nervous System	UBERON:0002028	hindbrain	CNhs11795	cerebellum, adult, pool1
UBERON:0001017	Central Nervous System	UBERON:0002028	hindbrain	CNhs12315	medulla oblongata, adult, donor10252
UBERON:0001017	Central Nervous System	UBERON:0002028	hindbrain	CNhs12322	locus coeruleus, adult, donor10252
UBERON:0001017	Central Nervous System	UBERON:0002028	hindbrain	CNhs12323	cerebellum, adult, donor10252
UBERON:0001017	Central Nervous System	UBERON:0002028	hindbrain	CNhs13799	cerebellum - adult, donor10196
UBERON:0001017	Central Nervous System	UBERON:0002028	hindbrain	CNhs13800	medulla oblongata - adult, donor10196
UBERON:0001017	Central Nervous System	UBERON:0002028	hindbrain	CNhs13808	locus coeruleus - adult, donor10196
UBERON:0001017	Central Nervous System	UBERON:0002028	hindbrain	CNhs14075	cerebellum, newborn, donor10223
UBERON:0001017	Central Nervous System	UBERON:0002028	hindbrain	CNhs14079	medulla oblongata, newborn, donor10223
UBERON:0001017	Central Nervous System	UBERON:0002028	hindbrain	CNhs14080	locus coeruleus, newborn, donor10223
UBERON:0001017	Central Nervous System	UBERON:0002028	hindbrain	CNhs14550	locus coeruleus, adult, donor10258
UBERON:0001017	Central Nervous System	UBERON:0002028	hindbrain	CNhs11321	Astrocyte - cerebellum, donor1
UBERON:0001017	Central Nervous System	UBERON:0002028	hindbrain	CNhs12081	Astrocyte - cerebellum, donor2
UBERON:0001017	Central Nervous System	UBERON:0002028	hindbrain	CNhs12117	Astrocyte - cerebellum, donor3
UBERON:0001017	Central Nervous System	UBERON:0002037	cerebellum	CNhs11795	cerebellum, adult, pool1
UBERON:0001017	Central Nervous System	UBERON:0002037	cerebellum	CNhs12323	cerebellum, adult, donor10252
UBERON:0001017	Central Nervous System	UBERON:0002037	cerebellum	CNhs13799	cerebellum - adult, donor10196
UBERON:0001017	Central Nervous System	UBERON:0002037	cerebellum	CNhs14075	cerebellum, newborn, donor10223
UBERON:0001017	Central Nervous System	UBERON:0002037	cerebellum	CNhs11321	Astrocyte - cerebellum, donor1
UBERON:0001017	Central Nervous System	UBERON:0002037	cerebellum	CNhs12081	Astrocyte - cerebellum, donor2
UBERON:0001017	Central Nervous System	UBERON:0002037	cerebellum	CNhs12117	Astrocyte - cerebellum, donor3
UBERON:0001017	Central Nervous System	UBERON:0002038	substantia nigra	CNhs12318	substantia nigra, adult, donor10252
UBERON:0001017	Central Nervous System	UBERON:0002038	substantia nigra	CNhs13803	substantia nigra - adult, donor10196
UBERON:0001017	Central Nervous System	UBERON:0002038	substantia nigra	CNhs14076	substantia nigra, newborn, donor10223
UBERON:0001017	Central Nervous System	UBERON:0002038	substantia nigra	CNhs14224	substantia nigra, adult, donor10258
UBERON:0001017	Central Nervous System	UBERON:0002148	locus ceruleus	CNhs12322	locus coeruleus, adult, donor10252
UBERON:0001017	Central Nervous System	UBERON:0002148	locus ceruleus	CNhs13808	locus coeruleus - adult, donor10196
UBERON:0001017	Central Nervous System	UBERON:0002148	locus ceruleus	CNhs14080	locus coeruleus, newborn, donor10223
UBERON:0001017	Central Nervous System	UBERON:0002148	locus ceruleus	CNhs14550	locus coeruleus, adult, donor10258
UBERON:0001017	Central Nervous System	UBERON:0002240	spinal cord	CNhs11764	spinal cord, fetal, donor1
UBERON:0001017	Central Nervous System	UBERON:0002240	spinal cord	CNhs12227	spinal cord, adult, donor10252
UBERON:0001017	Central Nervous System	UBERON:0002240	spinal cord	CNhs13807	spinal cord - adult, donor10196
UBERON:0001017	Central Nervous System	UBERON:0002240	spinal cord	CNhs14077	spinal cord, newborn, donor10223
UBERON:0001017	Central Nervous System	UBERON:0002240	spinal cord	CNhs14222	spinal cord, adult, donor10258
UBERON:0001017	Central Nervous System	UBERON:0002298	brainstem	CNhs10640	pons, adult, pool1
UBERON:0001017	Central Nervous System	UBERON:0002298	brainstem	CNhs10645	medulla oblongata, adult, pool1
UBERON:0001017	Central Nervous System	UBERON:0002298	brainstem	CNhs12315	medulla oblongata, adult, donor10252
UBERON:0001017	Central Nervous System	UBERON:0002298	brainstem	CNhs12322	locus coeruleus, adult, donor10252
UBERON:0001017	Central Nervous System	UBERON:0002298	brainstem	CNhs13800	medulla oblongata - adult, donor10196
UBERON:0001017	Central Nervous System	UBERON:0002298	brainstem	CNhs13808	locus coeruleus - adult, donor10196
UBERON:0001017	Central Nervous System	UBERON:0002298	brainstem	CNhs14079	medulla oblongata, newborn, donor10223
UBERON:0001017	Central Nervous System	UBERON:0002298	brainstem	CNhs14080	locus coeruleus, newborn, donor10223
UBERON:0001017	Central Nervous System	UBERON:0002298	brainstem	CNhs14550	locus coeruleus, adult, donor10258
UBERON:0001017	Central Nervous System	UBERON:0002308	nucleus of brain	CNhs10644	nucleus accumbens, adult, pool1
UBERON:0001017	Central Nervous System	UBERON:0002308	nucleus of brain	CNhs13912	putamen, adult, donor10252
UBERON:0001017	Central Nervous System	UBERON:0002308	nucleus of brain	CNhs12318	substantia nigra, adult, donor10252
UBERON:0001017	Central Nervous System	UBERON:0002308	nucleus of brain	CNhs12319	globus pallidus, adult, donor10252
UBERON:0001017	Central Nervous System	UBERON:0002308	nucleus of brain	CNhs12321	caudate nucleus, adult, donor10252
UBERON:0001017	Central Nervous System	UBERON:0002308	nucleus of brain	CNhs12322	locus coeruleus, adult, donor10252
UBERON:0001017	Central Nervous System	UBERON:0002308	nucleus of brain	CNhs13801	globus pallidus - adult, donor10196
UBERON:0001017	Central Nervous System	UBERON:0002308	nucleus of brain	CNhs12324	putamen, adult, donor10196
UBERON:0001017	Central Nervous System	UBERON:0002308	nucleus of brain	CNhs13802	caudate nucleus - adult, donor10196
UBERON:0001017	Central Nervous System	UBERON:0002308	nucleus of brain	CNhs13803	substantia nigra - adult, donor10196
UBERON:0001017	Central Nervous System	UBERON:0002308	nucleus of brain	CNhs13808	locus coeruleus - adult, donor10196
UBERON:0001017	Central Nervous System	UBERON:0002308	nucleus of brain	CNhs14071	caudate nucleus, newborn, donor10223
UBERON:0001017	Central Nervous System	UBERON:0002308	nucleus of brain	CNhs14076	substantia nigra, newborn, donor10223
UBERON:0001017	Central Nervous System	UBERON:0002308	nucleus of brain	CNhs14080	locus coeruleus, newborn, donor10223
UBERON:0001017	Central Nervous System	UBERON:0002308	nucleus of brain	CNhs14082	globus pallidus, newborn, donor10223
UBERON:0001017	Central Nervous System	UBERON:0002308	nucleus of brain	CNhs14083	putamen, newborn, donor10223
UBERON:0001017	Central Nervous System	UBERON:0002308	nucleus of brain	CNhs14549	globus pallidus, adult, donor10258
UBERON:0001017	Central Nervous System	UBERON:0002308	nucleus of brain	CNhs14224	substantia nigra, adult, donor10258
UBERON:0001017	Central Nervous System	UBERON:0002308	nucleus of brain	Cnhs14225	putamen, adult, donor10258
UBERON:0001017	Central Nervous System	UBERON:0002308	nucleus of brain	CNhs14550	locus coeruleus, adult, donor10258
UBERON:0001017	Central Nervous System	UBERON:0002316	white matter	CNhs10649	corpus callosum, adult, pool1

UBERON:0001017	Central Nervous System	UBERON:0002336	corpus callosum	CNhs10649	corpus callosum, adult, pool1
UBERON:0001017	Central Nervous System	UBERON:0002360	meninx	CNhs10648	dura mater, adult, donor1
UBERON:0001017	Central Nervous System	UBERON:0002360	meninx	CNhs12840	cerebral meninges, adult
UBERON:0001017	Central Nervous System	UBERON:0002363	dura mater	CNhs10648	dura mater, adult, donor1
UBERON:0001017	Central Nervous System	UBERON:0002420	basal ganglion	CNhs10644	nucleus accumbens, adult, pool1
UBERON:0001017	Central Nervous System	UBERON:0002420	basal ganglion	CNhs12311	amygdala, adult, donor10252
UBERON:0001017	Central Nervous System	UBERON:0002420	basal ganglion	CNhs13912	putamen, adult, donor10252
UBERON:0001017	Central Nervous System	UBERON:0002420	basal ganglion	CNhs12318	substantia nigra, adult, donor10252
UBERON:0001017	Central Nervous System	UBERON:0002420	basal ganglion	CNhs12319	globus pallidus, adult, donor10252
UBERON:0001017	Central Nervous System	UBERON:0002420	basal ganglion	CNhs12321	caudate nucleus, adult, donor10252
UBERON:0001017	Central Nervous System	UBERON:0002420	basal ganglion	CNhs13793	amygdala - adult, donor10196
UBERON:0001017	Central Nervous System	UBERON:0002420	basal ganglion	CNhs13801	globus pallidus - adult, donor10196
UBERON:0001017	Central Nervous System	UBERON:0002420	basal ganglion	CNhs12324	putamen, adult, donor10196
UBERON:0001017	Central Nervous System	UBERON:0002420	basal ganglion	CNhs13802	caudate nucleus - adult, donor10196
UBERON:0001017	Central Nervous System	UBERON:0002420	basal ganglion	CNhs13803	substantia nigra - adult, donor10196
UBERON:0001017	Central Nervous System	UBERON:0002420	basal ganglion	CNhs14071	caudate nucleus, newborn, donor10223
UBERON:0001017	Central Nervous System	UBERON:0002420	basal ganglion	CNhs14076	substantia nigra, newborn, donor10223
UBERON:0001017	Central Nervous System	UBERON:0002420	basal ganglion	CNhs14078	amygdala, newborn, donor10223
UBERON:0001017	Central Nervous System	UBERON:0002420	basal ganglion	CNhs14082	globus pallidus, newborn, donor10223
UBERON:0001017	Central Nervous System	UBERON:0002420	basal ganglion	CNhs14083	putamen, newborn, donor10223
UBERON:0001017	Central Nervous System	UBERON:0002420	basal ganglion	CNhs14549	globus pallidus, adult, donor10258
UBERON:0001017	Central Nervous System	UBERON:0002420	basal ganglion	CNhs14224	substantia nigra, adult, donor10258
UBERON:0001017	Central Nervous System	UBERON:0002420	basal ganglion	Cnhs14225	putamen, adult, donor10258
UBERON:0001017	Central Nervous System	UBERON:0002421	hippocampal formation	CNhs12312	hippocampus, adult, donor10252
UBERON:0001017	Central Nervous System	UBERON:0002421	hippocampal formation	CNhs13795	hippocampus - adult, donor10196
UBERON:0001017	Central Nervous System	UBERON:0002421	hippocampal formation	CNhs14081	hippocampus, newborn, donor10223
UBERON:0001017	Central Nervous System	UBERON:0002421	hippocampal formation	CNhs14227	hippocampus, adult, donor10258
UBERON:0001017	Central Nervous System	UBERON:0002435	striatum	CNhs10644	nucleus accumbens, adult, pool1
UBERON:0001017	Central Nervous System	UBERON:0002435	striatum	CNhs13912	putamen, adult, donor10252
UBERON:0001017	Central Nervous System	UBERON:0002435	striatum	CNhs12321	caudate nucleus, adult, donor10252
UBERON:0001017	Central Nervous System	UBERON:0002435	striatum	CNhs12324	putamen, adult, donor10196
UBERON:0001017	Central Nervous System	UBERON:0002435	striatum	CNhs13802	caudate nucleus - adult, donor10196
UBERON:0001017	Central Nervous System	UBERON:0002435	striatum	CNhs14071	caudate nucleus, newborn, donor10223
UBERON:0001017	Central Nervous System	UBERON:0002435	striatum	CNhs14083	putamen, newborn, donor10223
UBERON:0001017	Central Nervous System	UBERON:0002435	striatum	Cnhs14225	putamen, adult, donor10258
UBERON:0001017	Central Nervous System	UBERON:0002437	cerebral white matter	CNhs10649	corpus callosum, adult, pool1
UBERON:0001017	Central Nervous System	UBERON:0002473	intercerebral commissure	CNhs10649	corpus callosum, adult, pool1
UBERON:0001017	Central Nervous System	UBERON:0002581	postcentral gyrus	CNhs10638	postcentral gyrus, adult, pool1
UBERON:0001017	Central Nervous System	UBERON:0002600	limbic lobe	CNhs12312	hippocampus, adult, donor10252
UBERON:0001017	Central Nervous System	UBERON:0002600	limbic lobe	CNhs13795	hippocampus - adult, donor10196
UBERON:0001017	Central Nervous System	UBERON:0002600	limbic lobe	CNhs14081	hippocampus, newborn, donor10223
UBERON:0001017	Central Nervous System	UBERON:0002600	limbic lobe	CNhs14227	hippocampus, adult, donor10258
UBERON:0001017	Central Nervous System	UBERON:0002616	regional part of brain	CNhs10637	temporal lobe, adult, pool1
UBERON:0001017	Central Nervous System	UBERON:0002616	regional part of brain	CNhs10638	postcentral gyrus, adult, pool1
UBERON:0001017	Central Nervous System	UBERON:0002616	regional part of brain	CNhs10640	pons, adult, pool1
UBERON:0001017	Central Nervous System	UBERON:0002616	regional part of brain	CNhs10641	parietal lobe, adult, pool1
UBERON:0001017	Central Nervous System	UBERON:0002616	regional part of brain	CNhs10642	paracentral gyrus, adult, pool1
UBERON:0001017	Central Nervous System	UBERON:0002616	regional part of brain	CNhs10643	occipital pole, adult, pool1
UBERON:0001017	Central Nervous System	UBERON:0002616	regional part of brain	CNhs10644	nucleus accumbens, adult, pool1
UBERON:0001017	Central Nervous System	UBERON:0002616	regional part of brain	CNhs10645	medulla oblongata, adult, pool1
UBERON:0001017	Central Nervous System	UBERON:0002616	regional part of brain	CNhs10646	insula, adult, pool1
UBERON:0001017	Central Nervous System	UBERON:0002616	regional part of brain	CNhs10647	frontal lobe, adult, pool1
UBERON:0001017	Central Nervous System	UBERON:0002616	regional part of brain	CNhs10649	corpus callosum, adult, pool1
UBERON:0001017	Central Nervous System	UBERON:0002616	regional part of brain	Cnhs11781	duodenum, fetal, donor1
UBERON:0001017	Central Nervous System	UBERON:0002616	regional part of brain	CNhs11782	parietal lobe, fetal, donor1
UBERON:0001017	Central Nervous System	UBERON:0002616	regional part of brain	CNhs11784	occipital lobe, fetal, donor1
UBERON:0001017	Central Nervous System	UBERON:0002616	regional part of brain	CNhs11787	occipital lobe, adult, donor1
UBERON:0001017	Central Nervous System	UBERON:0002616	regional part of brain	CNhs11795	cerebellum, adult, pool1
UBERON:0001017	Central Nervous System	UBERON:0002616	regional part of brain	CNhs12310	medial frontal gyrus, adult, donor10252
UBERON:0001017	Central Nervous System	UBERON:0002616	regional part of brain	CNhs12311	amygdala, adult, donor10252
UBERON:0001017	Central Nervous System	UBERON:0002616	regional part of brain	CNhs13912	putamen, adult, donor10252
UBERON:0001017	Central Nervous System	UBERON:0002616	regional part of brain	CNhs12312	hippocampus, adult, donor10252
UBERON:0001017	Central Nervous System	UBERON:0002616	regional part of brain	CNhs12314	thalamus, adult, donor10252
UBERON:0001017	Central Nervous System	UBERON:0002616	regional part of brain	CNhs12315	medulla oblongata, adult, donor10252
UBERON:0001017	Central Nervous System	UBERON:0002616	regional part of brain	CNhs12316	medial temporal gyrus, adult, donor10252
UBERON:0001017	Central Nervous System	UBERON:0002616	regional part of brain	CNhs12317	parietal lobe, adult, donor10252
UBERON:0001017	Central Nervous System	UBERON:0002616	regional part of brain	CNhs12318	substantia nigra, adult, donor10252
UBERON:0001017	Central Nervous System	UBERON:0002616	regional part of brain	CNhs12228	pineal gland, adult, donor10252
UBERON:0001017	Central Nervous System	UBERON:0002616	regional part of brain	CNhs12319	globus pallidus, adult, donor10252
UBERON:0001017	Central Nervous System	UBERON:0002616	regional part of brain	CNhs12229	pituitary gland, adult, donor10252
UBERON:0001017	Central Nervous System	UBERON:0002616	regional part of brain	CNhs12320	occipital cortex, adult, donor10252
UBERON:0001017	Central Nervous System	UBERON:0002616	regional part of brain	CNhs12321	caudate nucleus, adult, donor10252
UBERON:0001017	Central Nervous System	UBERON:0002616	regional part of brain	CNhs12322	locus coeruleus, adult, donor10252
UBERON:0001017	Central Nervous System	UBERON:0002616	regional part of brain	CNhs12323	cerebellum, adult, donor10252
UBERON:0001017	Central Nervous System	UBERON:0002616	regional part of brain	CNhs13793	amygdala - adult, donor10196
UBERON:0001017	Central Nervous System	UBERON:0002616	regional part of brain	CNhs13794	thalamus - adult, donor10196
UBERON:0001017	Central Nervous System	UBERON:0002616	regional part of brain	CNhs13795	hippocampus - adult, donor10196
UBERON:0001017	Central Nervous System	UBERON:0002616	regional part of brain	CNhs13796	medial frontal gyrus - adult, donor10196
UBERON:0001017	Central Nervous System	UBERON:0002616	regional part of brain	CNhs13797	parietal lobe - adult, donor10196
UBERON:0001017	Central Nervous System	UBERON:0002616	regional part of brain	CNhs13798	occipital cortex - adult, donor10196
UBERON:0001017	Central Nervous System	UBERON:0002616	regional part of brain	CNhs13799	cerebellum - adult, donor10196
UBERON:0001017	Central Nervous System	UBERON:0002616	regional part of brain	CNhs13800	medulla oblongata - adult, donor10196
UBERON:0001017	Central Nervous System	UBERON:0002616	regional part of brain	CNhs13801	globus pallidus - adult, donor10196
UBERON:0001017	Central Nervous System	UBERON:0002616	regional part of brain	CNhs12324	putamen, adult, donor10196
UBERON:0001017	Central Nervous System	UBERON:0002616	regional part of brain	CNhs13802	caudate nucleus - adult, donor10196
UBERON:0001017	Central Nervous System	UBERON:0002616	regional part of brain	CNhs13803	substantia nigra - adult, donor10196
UBERON:0001017	Central Nervous System	UBERON:0002616	regional part of brain	CNhs13804	pineal gland - adult, donor10196
UBERON:0001017	Central Nervous System	UBERON:0002616	regional part of brain	CNhs13805	pituitary gland - adult, donor10196
UBERON:0001017	Central Nervous System	UBERON:0002616	regional part of brain	CNhs13808	locus coeruleus - adult, donor10196
UBERON:0001017	Central Nervous System	UBERON:0002616	regional part of brain	CNhs13809	medial temporal gyrus - adult, donor10196
UBERON:0001017	Central Nervous System	UBERON:0002616	regional part of brain	CNhs12610	diencephalon, adult
UBERON:0001017	Central Nervous System	UBERON:0002616	regional part of brain	CNhs14069	medial frontal gyrus, newborn, donor10223
UBERON:0001017	Central Nervous System	UBERON:0002616	regional part of brain	CNhs14070	medial temporal gyrus, newborn, donor10223
UBERON:0001017	Central Nervous System	UBERON:0002616	regional part of brain	CNhs14071	caudate nucleus, newborn, donor10223
UBERON:0001017	Central Nervous System	UBERON:0002616	regional part of brain	CNhs14073	occipital cortex, newborn, donor10223
UBERON:0001017	Central Nervous System	UBERON:0002616	regional part of brain	CNhs14074	parietal lobe, newborn, donor10223
UBERON:0001017	Central Nervous System	UBERON:0002616	regional part of brain	CNhs14075	cerebellum, newborn, donor10223
UBERON:0001017	Central Nervous System	UBERON:0002616	regional part of brain	CNhs14076	substantia nigra, newborn, donor10223

UBERON:0001017	Central Nervous System	UBERON:0002616	regional part of brain	CNhs14078	amygdala, newborn, donor10223
UBERON:0001017	Central Nervous System	UBERON:0002616	regional part of brain	CNhs14079	medulla oblongata, newborn, donor10223
UBERON:0001017	Central Nervous System	UBERON:0002616	regional part of brain	CNhs14080	locus coeruleus, newborn, donor10223
UBERON:0001017	Central Nervous System	UBERON:0002616	regional part of brain	CNhs14081	hippocampus, newborn, donor10223
UBERON:0001017	Central Nervous System	UBERON:0002616	regional part of brain	CNhs14082	globus pallidus, newborn, donor10223
UBERON:0001017	Central Nervous System	UBERON:0002616	regional part of brain	CNhs14083	putamen, newborn, donor10223
UBERON:0001017	Central Nervous System	UBERON:0002616	regional part of brain	CNhs14084	thalamus, newborn, donor10223
UBERON:0001017	Central Nervous System	UBERON:0002616	regional part of brain	CNhs14549	globus pallidus, adult, donor10258
UBERON:0001017	Central Nervous System	UBERON:0002616	regional part of brain	CNhs14221	medial frontal gyrus, adult, donor10258
UBERON:0001017	Central Nervous System	UBERON:0002616	regional part of brain	Cnhs14223	thalamus, adult, donor10258
UBERON:0001017	Central Nervous System	UBERON:0002616	regional part of brain	CNhs14224	substantia nigra, adult, donor10258
UBERON:0001017	Central Nervous System	UBERON:0002616	regional part of brain	Cnhs14225	putamen, adult, donor10258
UBERON:0001017	Central Nervous System	UBERON:0002616	regional part of brain	CNhs14226	parietal cortex, adult, donor10258
UBERON:0001017	Central Nervous System	UBERON:0002616	regional part of brain	CNhs14227	hippocampus, adult, donor10258
UBERON:0001017	Central Nervous System	UBERON:0002616	regional part of brain	CNhs14550	locus coeruleus, adult, donor10258
UBERON:0001017	Central Nervous System	UBERON:0002616	regional part of brain	Cnhs14229	medial temporal gyrus, adult, donor10258
UBERON:0001017	Central Nervous System	UBERON:0002616	regional part of brain	CNhs14230	pineal gland, adult, donor10258
UBERON:0001017	Central Nervous System	UBERON:0002616	regional part of brain	CNhs14231	pituitary gland, adult, donor10258
UBERON:0001017	Central Nervous System	UBERON:0002616	regional part of brain	CNhs10864	Astrocyte - cerebral cortex, donor1
UBERON:0001017	Central Nervous System	UBERON:0002616	regional part of brain	CNhs11960	Astrocyte - cerebral cortex, donor2
UBERON:0001017	Central Nervous System	UBERON:0002616	regional part of brain	CNhs12005	Astrocyte - cerebral cortex, donor3
UBERON:0001017	Central Nervous System	UBERON:0002616	regional part of brain	CNhs11321	Astrocyte - cerebellum, donor1
UBERON:0001017	Central Nervous System	UBERON:0002616	regional part of brain	CNhs12081	Astrocyte - cerebellum, donor2
UBERON:0001017	Central Nervous System	UBERON:0002616	regional part of brain	CNhs12117	Astrocyte - cerebellum, donor3
UBERON:0001017	Central Nervous System	UBERON:0002619	regional part of cerebral cortex	CNhs10637	temporal lobe, adult, pool1
UBERON:0001017	Central Nervous System	UBERON:0002619	regional part of cerebral cortex	CNhs10638	postcentral gyrus, adult, pool1
UBERON:0001017	Central Nervous System	UBERON:0002619	regional part of cerebral cortex	CNhs10641	parietal lobe, adult, pool1
UBERON:0001017	Central Nervous System	UBERON:0002619	regional part of cerebral cortex	CNhs10643	occipital pole, adult, pool1
UBERON:0001017	Central Nervous System	UBERON:0002619	regional part of cerebral cortex	CNhs10646	insula, adult, pool1
UBERON:0001017	Central Nervous System	UBERON:0002619	regional part of cerebral cortex	CNhs10647	frontal lobe, adult, pool1
UBERON:0001017	Central Nervous System	UBERON:0002619	regional part of cerebral cortex	Cnhs11781	duodenum, fetal, donor1
UBERON:0001017	Central Nervous System	UBERON:0002619	regional part of cerebral cortex	CNhs11782	parietal lobe, fetal, donor1
UBERON:0001017	Central Nervous System	UBERON:0002619	regional part of cerebral cortex	CNhs11784	occipital lobe, fetal, donor1
UBERON:0001017	Central Nervous System	UBERON:0002619	regional part of cerebral cortex	CNhs11787	occipital lobe, adult, donor1
UBERON:0001017	Central Nervous System	UBERON:0002619	regional part of cerebral cortex	CNhs12310	medial frontal gyrus, adult, donor10252
UBERON:0001017	Central Nervous System	UBERON:0002619	regional part of cerebral cortex	CNhs12311	amygdala, adult, donor10252
UBERON:0001017	Central Nervous System	UBERON:0002619	regional part of cerebral cortex	CNhs12312	hippocampus, adult, donor10252
UBERON:0001017	Central Nervous System	UBERON:0002619	regional part of cerebral cortex	CNhs12316	medial temporal gyrus, adult, donor10252
UBERON:0001017	Central Nervous System	UBERON:0002619	regional part of cerebral cortex	CNhs12317	parietal lobe, adult, donor10252
UBERON:0001017	Central Nervous System	UBERON:0002619	regional part of cerebral cortex	CNhs12320	occipital cortex, adult, donor10252
UBERON:0001017	Central Nervous System	UBERON:0002619	regional part of cerebral cortex	CNhs13793	amygdala - adult, donor10196
UBERON:0001017	Central Nervous System	UBERON:0002619	regional part of cerebral cortex	CNhs13795	hippocampus - adult, donor10196
UBERON:0001017	Central Nervous System	UBERON:0002619	regional part of cerebral cortex	CNhs13796	medial frontal gyrus - adult, donor10196
UBERON:0001017	Central Nervous System	UBERON:0002619	regional part of cerebral cortex	CNhs13797	parietal lobe - adult, donor10196
UBERON:0001017	Central Nervous System	UBERON:0002619	regional part of cerebral cortex	CNhs13798	occipital cortex - adult, donor10196
UBERON:0001017	Central Nervous System	UBERON:0002619	regional part of cerebral cortex	CNhs13809	medial temporal gyrus - adult, donor10196
UBERON:0001017	Central Nervous System	UBERON:0002619	regional part of cerebral cortex	CNhs14069	medial frontal gyrus, newborn, donor10223
UBERON:0001017	Central Nervous System	UBERON:0002619	regional part of cerebral cortex	CNhs14070	medial temporal gyrus, newborn, donor10223
UBERON:0001017	Central Nervous System	UBERON:0002619	regional part of cerebral cortex	CNhs14073	occipital cortex, newborn, donor10223
UBERON:0001017	Central Nervous System	UBERON:0002619	regional part of cerebral cortex	CNhs14074	parietal lobe, newborn, donor10223
UBERON:0001017	Central Nervous System	UBERON:0002619	regional part of cerebral cortex	CNhs14078	amygdala, newborn, donor10223
UBERON:0001017	Central Nervous System	UBERON:0002619	regional part of cerebral cortex	CNhs14081	hippocampus, newborn, donor10223
UBERON:0001017	Central Nervous System	UBERON:0002619	regional part of cerebral cortex	CNhs14221	medial frontal gyrus, adult, donor10258
UBERON:0001017	Central Nervous System	UBERON:0002619	regional part of cerebral cortex	CNhs14226	parietal cortex, adult, donor10258
UBERON:0001017	Central Nervous System	UBERON:0002619	regional part of cerebral cortex	CNhs14227	hippocampus, adult, donor10258
UBERON:0001017	Central Nervous System	UBERON:0002619	regional part of cerebral cortex	Cnhs14229	medial temporal gyrus, adult, donor10258
UBERON:0001017	Central Nervous System	UBERON:0002680	regional part of metencephalon	CNhs10640	pons, adult, pool1
UBERON:0001017	Central Nervous System	UBERON:0002680	regional part of metencephalon	CNhs11795	cerebellum, adult, pool1
UBERON:0001017	Central Nervous System	UBERON:0002680	regional part of metencephalon	CNhs12322	locus coeruleus, adult, donor10252
UBERON:0001017	Central Nervous System	UBERON:0002680	regional part of metencephalon	CNhs12323	cerebellum, adult, donor10252
UBERON:0001017	Central Nervous System	UBERON:0002680	regional part of metencephalon	CNhs13799	cerebellum - adult, donor10196
UBERON:0001017	Central Nervous System	UBERON:0002680	regional part of metencephalon	CNhs13808	locus coeruleus - adult, donor10196
UBERON:0001017	Central Nervous System	UBERON:0002680	regional part of metencephalon	CNhs14075	cerebellum, newborn, donor10223
UBERON:0001017	Central Nervous System	UBERON:0002680	regional part of metencephalon	CNhs14080	locus coeruleus, newborn, donor10223
UBERON:0001017	Central Nervous System	UBERON:0002680	regional part of metencephalon	CNhs14550	locus coeruleus, adult, donor10258
UBERON:0001017	Central Nervous System	UBERON:0002680	regional part of metencephalon	CNhs11321	Astrocyte - cerebellum, donor1
UBERON:0001017	Central Nervous System	UBERON:0002680	regional part of metencephalon	CNhs12081	Astrocyte - cerebellum, donor2
UBERON:0001017	Central Nervous System	UBERON:0002680	regional part of metencephalon	CNhs12117	Astrocyte - cerebellum, donor3
UBERON:0001017	Central Nervous System	UBERON:0002702	middle frontal gyrus	CNhs12310	medial frontal gyrus, adult, donor10252
UBERON:0001017	Central Nervous System	UBERON:0002702	middle frontal gyrus	CNhs13796	medial frontal gyrus - adult, donor10196
UBERON:0001017	Central Nervous System	UBERON:0002702	middle frontal gyrus	CNhs14069	medial frontal gyrus, newborn, donor10223
UBERON:0001017	Central Nervous System	UBERON:0002702	middle frontal gyrus	CNhs14221	medial frontal gyrus, adult, donor10258
UBERON:0001017	Central Nervous System	UBERON:0002757	regional part of epithalamus	CNhs12228	pineal gland, adult, donor10252
UBERON:0001017	Central Nervous System	UBERON:0002757	regional part of epithalamus	CNhs13804	pineal gland - adult, donor10196
UBERON:0001017	Central Nervous System	UBERON:0002757	regional part of epithalamus	CNhs14230	pineal gland, adult, donor10258
UBERON:0001017	Central Nervous System	UBERON:0002771	middle temporal gyrus	CNhs12316	medial temporal gyrus, adult, donor10252
UBERON:0001017	Central Nervous System	UBERON:0002771	middle temporal gyrus	CNhs13809	medial temporal gyrus - adult, donor10196
UBERON:0001017	Central Nervous System	UBERON:0002771	middle temporal gyrus	CNhs14070	medial temporal gyrus, newborn, donor10223
UBERON:0001017	Central Nervous System	UBERON:0002771	middle temporal gyrus	Cnhs14229	medial temporal gyrus, adult, donor10258
UBERON:0001017	Central Nervous System	UBERON:0002780	regional part of forebrain	CNhs10637	temporal lobe, adult, pool1
UBERON:0001017	Central Nervous System	UBERON:0002780	regional part of forebrain	CNhs10638	postcentral gyrus, adult, pool1
UBERON:0001017	Central Nervous System	UBERON:0002780	regional part of forebrain	CNhs10641	parietal lobe, adult, pool1
UBERON:0001017	Central Nervous System	UBERON:0002780	regional part of forebrain	CNhs10642	paracentral gyrus, adult, pool1
UBERON:0001017	Central Nervous System	UBERON:0002780	regional part of forebrain	CNhs10643	occipital pole, adult, pool1
UBERON:0001017	Central Nervous System	UBERON:0002780	regional part of forebrain	CNhs10644	nucleus accumbens, adult, pool1
UBERON:0001017	Central Nervous System	UBERON:0002780	regional part of forebrain	CNhs10646	insula, adult, pool1
UBERON:0001017	Central Nervous System	UBERON:0002780	regional part of forebrain	CNhs10647	frontal lobe, adult, pool1
UBERON:0001017	Central Nervous System	UBERON:0002780	regional part of forebrain	CNhs10649	corpus callosum, adult, pool1
UBERON:0001017	Central Nervous System	UBERON:0002780	regional part of forebrain	Cnhs11781	duodenum, fetal, donor1
UBERON:0001017	Central Nervous System	UBERON:0002780	regional part of forebrain	CNhs11782	parietal lobe, fetal, donor1
UBERON:0001017	Central Nervous System	UBERON:0002780	regional part of forebrain	CNhs11784	occipital lobe, fetal, donor1
UBERON:0001017	Central Nervous System	UBERON:0002780	regional part of forebrain	CNhs11787	occipital lobe, adult, donor1
UBERON:0001017	Central Nervous System	UBERON:0002780	regional part of forebrain	CNhs12310	medial frontal gyrus, adult, donor10252
UBERON:0001017	Central Nervous System	UBERON:0002780	regional part of forebrain	CNhs12311	amygdala, adult, donor10252
UBERON:0001017	Central Nervous System	UBERON:0002780	regional part of forebrain	CNhs13912	putamen, adult, donor10252
UBERON:0001017	Central Nervous System	UBERON:0002780	regional part of forebrain	CNhs12312	hippocampus, adult, donor10252
UBERON:0001017	Central Nervous System	UBERON:0002780	regional part of forebrain	CNhs12314	thalamus, adult, donor10252
UBERON:0001017	Central Nervous System	UBERON:0002780	regional part of forebrain	CNhs12316	medial temporal gyrus, adult, donor10252

UBERON:0001017	Central Nervous System	UBERON:0002780	regional part of forebrain	CNhs12317	parietal lobe, adult, donor10252
UBERON:0001017	Central Nervous System	UBERON:0002780	regional part of forebrain	CNhs12318	substantia nigra, adult, donor10252
UBERON:0001017	Central Nervous System	UBERON:0002780	regional part of forebrain	CNhs12228	pineal gland, adult, donor10252
UBERON:0001017	Central Nervous System	UBERON:0002780	regional part of forebrain	CNhs12319	globus pallidus, adult, donor10252
UBERON:0001017	Central Nervous System	UBERON:0002780	regional part of forebrain	CNhs12229	pituitary gland, adult, donor10252
UBERON:0001017	Central Nervous System	UBERON:0002780	regional part of forebrain	CNhs12320	occipital cortex, adult, donor10252
UBERON:0001017	Central Nervous System	UBERON:0002780	regional part of forebrain	CNhs12321	caudate nucleus, adult, donor10252
UBERON:0001017	Central Nervous System	UBERON:0002780	regional part of forebrain	CNhs13793	amygdala - adult, donor10196
UBERON:0001017	Central Nervous System	UBERON:0002780	regional part of forebrain	CNhs13794	thalamus - adult, donor10196
UBERON:0001017	Central Nervous System	UBERON:0002780	regional part of forebrain	CNhs13795	hippocampus - adult, donor10196
UBERON:0001017	Central Nervous System	UBERON:0002780	regional part of forebrain	CNhs13796	medial frontal gyrus - adult, donor10196
UBERON:0001017	Central Nervous System	UBERON:0002780	regional part of forebrain	CNhs13797	parietal lobe - adult, donor10196
UBERON:0001017	Central Nervous System	UBERON:0002780	regional part of forebrain	CNhs13798	occipital cortex - adult, donor10196
UBERON:0001017	Central Nervous System	UBERON:0002780	regional part of forebrain	CNhs13801	globus pallidus - adult, donor10196
UBERON:0001017	Central Nervous System	UBERON:0002780	regional part of forebrain	CNhs12324	putamen, adult, donor10196
UBERON:0001017	Central Nervous System	UBERON:0002780	regional part of forebrain	CNhs13802	caudate nucleus - adult, donor10196
UBERON:0001017	Central Nervous System	UBERON:0002780	regional part of forebrain	CNhs13803	substantia nigra - adult, donor10196
UBERON:0001017	Central Nervous System	UBERON:0002780	regional part of forebrain	CNhs13804	pineal gland - adult, donor10196
UBERON:0001017	Central Nervous System	UBERON:0002780	regional part of forebrain	CNhs13805	pituitary gland - adult, donor10196
UBERON:0001017	Central Nervous System	UBERON:0002780	regional part of forebrain	CNhs13809	medial temporal gyrus - adult, donor10196
UBERON:0001017	Central Nervous System	UBERON:0002780	regional part of forebrain	CNhs12610	diencephalon, adult
UBERON:0001017	Central Nervous System	UBERON:0002780	regional part of forebrain	CNhs14069	medial frontal gyrus, newborn, donor10223
UBERON:0001017	Central Nervous System	UBERON:0002780	regional part of forebrain	CNhs14070	medial temporal gyrus, newborn, donor10223
UBERON:0001017	Central Nervous System	UBERON:0002780	regional part of forebrain	CNhs14071	caudate nucleus, newborn, donor10223
UBERON:0001017	Central Nervous System	UBERON:0002780	regional part of forebrain	CNhs14073	occipital cortex, newborn, donor10223
UBERON:0001017	Central Nervous System	UBERON:0002780	regional part of forebrain	CNhs14074	parietal lobe, newborn, donor10223
UBERON:0001017	Central Nervous System	UBERON:0002780	regional part of forebrain	CNhs14076	substantia nigra, newborn, donor10223
UBERON:0001017	Central Nervous System	UBERON:0002780	regional part of forebrain	CNhs14078	amygdala, newborn, donor10223
UBERON:0001017	Central Nervous System	UBERON:0002780	regional part of forebrain	CNhs14081	hippocampus, newborn, donor10223
UBERON:0001017	Central Nervous System	UBERON:0002780	regional part of forebrain	CNhs14082	globus pallidus, newborn, donor10223
UBERON:0001017	Central Nervous System	UBERON:0002780	regional part of forebrain	CNhs14083	putamen, newborn, donor10223
UBERON:0001017	Central Nervous System	UBERON:0002780	regional part of forebrain	CNhs14084	thalamus, newborn, donor10223
UBERON:0001017	Central Nervous System	UBERON:0002780	regional part of forebrain	CNhs14549	globus pallidus, adult, donor10258
UBERON:0001017	Central Nervous System	UBERON:0002780	regional part of forebrain	CNhs14221	medial frontal gyrus, adult, donor10258
UBERON:0001017	Central Nervous System	UBERON:0002780	regional part of forebrain	Cnhs14223	thalamus, adult, donor10258
UBERON:0001017	Central Nervous System	UBERON:0002780	regional part of forebrain	CNhs14224	substantia nigra, adult, donor10258
UBERON:0001017	Central Nervous System	UBERON:0002780	regional part of forebrain	Cnhs14225	putamen, adult, donor10258
UBERON:0001017	Central Nervous System	UBERON:0002780	regional part of forebrain	CNhs14226	parietal cortex, adult, donor10258
UBERON:0001017	Central Nervous System	UBERON:0002780	regional part of forebrain	CNhs14227	hippocampus, adult, donor10258
UBERON:0001017	Central Nervous System	UBERON:0002780	regional part of forebrain	Cnhs14229	medial temporal gyrus, adult, donor10258
UBERON:0001017	Central Nervous System	UBERON:0002780	regional part of forebrain	CNhs14230	pineal gland, adult, donor10258
UBERON:0001017	Central Nervous System	UBERON:0002780	regional part of forebrain	CNhs14231	pituitary gland, adult, donor10258
UBERON:0001017	Central Nervous System	UBERON:0002780	regional part of forebrain	CNhs10864	Astrocyte - cerebral cortex, donor1
UBERON:0001017	Central Nervous System	UBERON:0002780	regional part of forebrain	CNhs11960	Astrocyte - cerebral cortex, donor2
UBERON:0001017	Central Nervous System	UBERON:0002780	regional part of forebrain	CNhs12005	Astrocyte - cerebral cortex, donor3
UBERON:0001017	Central Nervous System	UBERON:0002784	regional part of diencephalon	CNhs12314	thalamus, adult, donor10252
UBERON:0001017	Central Nervous System	UBERON:0002784	regional part of diencephalon	CNhs12228	pineal gland, adult, donor10252
UBERON:0001017	Central Nervous System	UBERON:0002784	regional part of diencephalon	CNhs13794	thalamus - adult, donor10196
UBERON:0001017	Central Nervous System	UBERON:0002784	regional part of diencephalon	CNhs13804	pineal gland - adult, donor10196
UBERON:0001017	Central Nervous System	UBERON:0002784	regional part of diencephalon	CNhs14084	thalamus, newborn, donor10223
UBERON:0001017	Central Nervous System	UBERON:0002784	regional part of diencephalon	Cnhs14223	thalamus, adult, donor10258
UBERON:0001017	Central Nervous System	UBERON:0002784	regional part of diencephalon	CNhs14230	pineal gland, adult, donor10258
UBERON:0001017	Central Nervous System	UBERON:0002791	regional part of telencephalon	CNhs10637	temporal lobe, adult, pool1
UBERON:0001017	Central Nervous System	UBERON:0002791	regional part of telencephalon	CNhs10638	postcentral gyrus, adult, pool1
UBERON:0001017	Central Nervous System	UBERON:0002791	regional part of telencephalon	CNhs10641	parietal lobe, adult, pool1
UBERON:0001017	Central Nervous System	UBERON:0002791	regional part of telencephalon	CNhs10643	occipital pole, adult, pool1
UBERON:0001017	Central Nervous System	UBERON:0002791	regional part of telencephalon	CNhs10644	nucleus accumbens, adult, pool1
UBERON:0001017	Central Nervous System	UBERON:0002791	regional part of telencephalon	CNhs10646	insula, adult, pool1
UBERON:0001017	Central Nervous System	UBERON:0002791	regional part of telencephalon	CNhs10647	frontal lobe, adult, pool1
UBERON:0001017	Central Nervous System	UBERON:0002791	regional part of telencephalon	Cnhs11781	duodenum, fetal, donor1
UBERON:0001017	Central Nervous System	UBERON:0002791	regional part of telencephalon	CNhs11782	parietal lobe, fetal, donor1
UBERON:0001017	Central Nervous System	UBERON:0002791	regional part of telencephalon	CNhs11784	occipital lobe, fetal, donor1
UBERON:0001017	Central Nervous System	UBERON:0002791	regional part of telencephalon	CNhs11787	occipital lobe, adult, donor1
UBERON:0001017	Central Nervous System	UBERON:0002791	regional part of telencephalon	CNhs12310	medial frontal gyrus, adult, donor10252
UBERON:0001017	Central Nervous System	UBERON:0002791	regional part of telencephalon	CNhs12311	amygdala, adult, donor10252
UBERON:0001017	Central Nervous System	UBERON:0002791	regional part of telencephalon	CNhs13912	putamen, adult, donor10252
UBERON:0001017	Central Nervous System	UBERON:0002791	regional part of telencephalon	CNhs12312	hippocampus, adult, donor10252
UBERON:0001017	Central Nervous System	UBERON:0002791	regional part of telencephalon	CNhs12316	medial temporal gyrus, adult, donor10252
UBERON:0001017	Central Nervous System	UBERON:0002791	regional part of telencephalon	CNhs12317	parietal lobe, adult, donor10252
UBERON:0001017	Central Nervous System	UBERON:0002791	regional part of telencephalon	CNhs12318	substantia nigra, adult, donor10252
UBERON:0001017	Central Nervous System	UBERON:0002791	regional part of telencephalon	CNhs12319	globus pallidus, adult, donor10252
UBERON:0001017	Central Nervous System	UBERON:0002791	regional part of telencephalon	CNhs12320	occipital cortex, adult, donor10252
UBERON:0001017	Central Nervous System	UBERON:0002791	regional part of telencephalon	CNhs12321	caudate nucleus, adult, donor10252
UBERON:0001017	Central Nervous System	UBERON:0002791	regional part of telencephalon	CNhs13793	amygdala - adult, donor10196
UBERON:0001017	Central Nervous System	UBERON:0002791	regional part of telencephalon	CNhs13795	hippocampus - adult, donor10196
UBERON:0001017	Central Nervous System	UBERON:0002791	regional part of telencephalon	CNhs13796	medial frontal gyrus - adult, donor10196
UBERON:0001017	Central Nervous System	UBERON:0002791	regional part of telencephalon	CNhs13797	parietal lobe - adult, donor10196
UBERON:0001017	Central Nervous System	UBERON:0002791	regional part of telencephalon	CNhs13798	occipital cortex - adult, donor10196
UBERON:0001017	Central Nervous System	UBERON:0002791	regional part of telencephalon	CNhs13801	globus pallidus - adult, donor10196
UBERON:0001017	Central Nervous System	UBERON:0002791	regional part of telencephalon	CNhs12324	putamen, adult, donor10196
UBERON:0001017	Central Nervous System	UBERON:0002791	regional part of telencephalon	CNhs13802	caudate nucleus - adult, donor10196
UBERON:0001017	Central Nervous System	UBERON:0002791	regional part of telencephalon	CNhs13803	substantia nigra - adult, donor10196
UBERON:0001017	Central Nervous System	UBERON:0002791	regional part of telencephalon	CNhs13809	medial temporal gyrus - adult, donor10196
UBERON:0001017	Central Nervous System	UBERON:0002791	regional part of telencephalon	CNhs14069	medial frontal gyrus, newborn, donor10223
UBERON:0001017	Central Nervous System	UBERON:0002791	regional part of telencephalon	CNhs14070	medial temporal gyrus, newborn, donor10223
UBERON:0001017	Central Nervous System	UBERON:0002791	regional part of telencephalon	CNhs14071	caudate nucleus, newborn, donor10223
UBERON:0001017	Central Nervous System	UBERON:0002791	regional part of telencephalon	CNhs14073	occipital cortex, newborn, donor10223
UBERON:0001017	Central Nervous System	UBERON:0002791	regional part of telencephalon	CNhs14074	parietal lobe, newborn, donor10223
UBERON:0001017	Central Nervous System	UBERON:0002791	regional part of telencephalon	CNhs14076	substantia nigra, newborn, donor10223
UBERON:0001017	Central Nervous System	UBERON:0002791	regional part of telencephalon	CNhs14078	amygdala, newborn, donor10223
UBERON:0001017	Central Nervous System	UBERON:0002791	regional part of telencephalon	CNhs14081	hippocampus, newborn, donor10223
UBERON:0001017	Central Nervous System	UBERON:0002791	regional part of telencephalon	CNhs14082	globus pallidus, newborn, donor10223
UBERON:0001017	Central Nervous System	UBERON:0002791	regional part of telencephalon	CNhs14083	putamen, newborn, donor10223
UBERON:0001017	Central Nervous System	UBERON:0002791	regional part of telencephalon	CNhs14549	globus pallidus, adult, donor10258
UBERON:0001017	Central Nervous System	UBERON:0002791	regional part of telencephalon	CNhs14221	medial frontal gyrus, adult, donor10258
UBERON:0001017	Central Nervous System	UBERON:0002791	regional part of telencephalon	CNhs14224	substantia nigra, adult, donor10258
UBERON:0001017	Central Nervous System	UBERON:0002791	regional part of telencephalon	Cnhs14225	putamen, adult, donor10258
UBERON:0001017	Central Nervous System	UBERON:0002791	regional part of telencephalon	CNhs14226	parietal cortex, adult, donor10258

UBERON:0001017	Central Nervous System	UBERON:0002791	regional part of telencephalon	CNhs14227	hippocampus, adult, donor10258
UBERON:0001017	Central Nervous System	UBERON:0002791	regional part of telencephalon	Cnhs14229	medial temporal gyrus, adult, donor10258
UBERON:0001017	Central Nervous System	UBERON:0002791	regional part of telencephalon	CNhs10864	Astrocyte - cerebral cortex, donor1
UBERON:0001017	Central Nervous System	UBERON:0002791	regional part of telencephalon	CNhs11960	Astrocyte - cerebral cortex, donor2
UBERON:0001017	Central Nervous System	UBERON:0002791	regional part of telencephalon	CNhs12005	Astrocyte - cerebral cortex, donor3
UBERON:0001017	Central Nervous System	UBERON:0002902	occipital pole	CNhs10643	occipital pole, adult, pool1
UBERON:0001017	Central Nervous System	UBERON:0002950	regional part of midbrain	CNhs12318	substantia nigra, adult, donor10252
UBERON:0001017	Central Nervous System	UBERON:0002950	regional part of midbrain	CNhs13803	substantia nigra - adult, donor10196
UBERON:0001017	Central Nervous System	UBERON:0002950	regional part of midbrain	CNhs14076	substantia nigra, newborn, donor10223
UBERON:0001017	Central Nervous System	UBERON:0002950	regional part of midbrain	CNhs14224	substantia nigra, adult, donor10258
UBERON:0001017	Central Nervous System	UBERON:0003022	lobe parts of cerebral cortex	CNhs12312	hippocampus, adult, donor10252
UBERON:0001017	Central Nervous System	UBERON:0003022	lobe parts of cerebral cortex	CNhs13795	hippocampus - adult, donor10196
UBERON:0001017	Central Nervous System	UBERON:0003022	lobe parts of cerebral cortex	CNhs14081	hippocampus, newborn, donor10223
UBERON:0001017	Central Nervous System	UBERON:0003022	lobe parts of cerebral cortex	CNhs14227	hippocampus, adult, donor10258
UBERON:0001017	Central Nervous System	UBERON:0003296	gland of diencephalon	CNhs12228	pineal gland, adult, donor10252
UBERON:0001017	Central Nervous System	UBERON:0003296	gland of diencephalon	CNhs12229	pituitary gland, adult, donor10252
UBERON:0001017	Central Nervous System	UBERON:0003296	gland of diencephalon	CNhs13804	pineal gland - adult, donor10196
UBERON:0001017	Central Nervous System	UBERON:0003296	gland of diencephalon	CNhs13805	pituitary gland - adult, donor10196
UBERON:0001017	Central Nervous System	UBERON:0003296	gland of diencephalon	CNhs14230	pineal gland, adult, donor10258
UBERON:0001017	Central Nervous System	UBERON:0003296	gland of diencephalon	CNhs14231	pituitary gland, adult, donor10258
UBERON:0001017	Central Nervous System	UBERON:0003528	brain grey matter	CNhs10637	temporal lobe, adult, pool1
UBERON:0001017	Central Nervous System	UBERON:0003528	brain grey matter	CNhs10638	postcentral gyrus, adult, pool1
UBERON:0001017	Central Nervous System	UBERON:0003528	brain grey matter	CNhs10641	parietal lobe, adult, pool1
UBERON:0001017	Central Nervous System	UBERON:0003528	brain grey matter	CNhs10643	occipital pole, adult, pool1
UBERON:0001017	Central Nervous System	UBERON:0003528	brain grey matter	CNhs10644	nucleus accumbens, adult, pool1
UBERON:0001017	Central Nervous System	UBERON:0003528	brain grey matter	CNhs10646	insula, adult, pool1
UBERON:0001017	Central Nervous System	UBERON:0003528	brain grey matter	CNhs10647	frontal lobe, adult, pool1
UBERON:0001017	Central Nervous System	UBERON:0003528	brain grey matter	Cnhs11781	duodenum, fetal, donor1
UBERON:0001017	Central Nervous System	UBERON:0003528	brain grey matter	CNhs11782	parietal lobe, fetal, donor1
UBERON:0001017	Central Nervous System	UBERON:0003528	brain grey matter	CNhs11784	occipital lobe, fetal, donor1
UBERON:0001017	Central Nervous System	UBERON:0003528	brain grey matter	CNhs11787	occipital lobe, adult, donor1
UBERON:0001017	Central Nervous System	UBERON:0003528	brain grey matter	CNhs12310	medial frontal gyrus, adult, donor10252
UBERON:0001017	Central Nervous System	UBERON:0003528	brain grey matter	CNhs12311	amygdala, adult, donor10252
UBERON:0001017	Central Nervous System	UBERON:0003528	brain grey matter	CNhs13912	putamen, adult, donor10252
UBERON:0001017	Central Nervous System	UBERON:0003528	brain grey matter	CNhs12312	hippocampus, adult, donor10252
UBERON:0001017	Central Nervous System	UBERON:0003528	brain grey matter	CNhs12316	medial temporal gyrus, adult, donor10252
UBERON:0001017	Central Nervous System	UBERON:0003528	brain grey matter	CNhs12317	parietal lobe, adult, donor10252
UBERON:0001017	Central Nervous System	UBERON:0003528	brain grey matter	CNhs12318	substantia nigra, adult, donor10252
UBERON:0001017	Central Nervous System	UBERON:0003528	brain grey matter	CNhs12319	globus pallidus, adult, donor10252
UBERON:0001017	Central Nervous System	UBERON:0003528	brain grey matter	CNhs12320	occipital cortex, adult, donor10252
UBERON:0001017	Central Nervous System	UBERON:0003528	brain grey matter	CNhs12321	caudate nucleus, adult, donor10252
UBERON:0001017	Central Nervous System	UBERON:0003528	brain grey matter	CNhs12322	locus coeruleus, adult, donor10252
UBERON:0001017	Central Nervous System	UBERON:0003528	brain grey matter	CNhs13793	amygdala - adult, donor10196
UBERON:0001017	Central Nervous System	UBERON:0003528	brain grey matter	CNhs13795	hippocampus - adult, donor10196
UBERON:0001017	Central Nervous System	UBERON:0003528	brain grey matter	CNhs13796	medial frontal gyrus - adult, donor10196
UBERON:0001017	Central Nervous System	UBERON:0003528	brain grey matter	CNhs13797	parietal lobe - adult, donor10196
UBERON:0001017	Central Nervous System	UBERON:0003528	brain grey matter	CNhs13798	occipital cortex - adult, donor10196
UBERON:0001017	Central Nervous System	UBERON:0003528	brain grey matter	CNhs13801	globus pallidus - adult, donor10196
UBERON:0001017	Central Nervous System	UBERON:0003528	brain grey matter	CNhs12324	putamen, adult, donor10196
UBERON:0001017	Central Nervous System	UBERON:0003528	brain grey matter	CNhs13802	caudate nucleus - adult, donor10196
UBERON:0001017	Central Nervous System	UBERON:0003528	brain grey matter	CNhs13803	substantia nigra - adult, donor10196
UBERON:0001017	Central Nervous System	UBERON:0003528	brain grey matter	CNhs13808	locus coeruleus - adult, donor10196
UBERON:0001017	Central Nervous System	UBERON:0003528	brain grey matter	CNhs13809	medial temporal gyrus - adult, donor10196
UBERON:0001017	Central Nervous System	UBERON:0003528	brain grey matter	CNhs14069	medial frontal gyrus, newborn, donor10223
UBERON:0001017	Central Nervous System	UBERON:0003528	brain grey matter	CNhs14070	medial temporal gyrus, newborn, donor10223
UBERON:0001017	Central Nervous System	UBERON:0003528	brain grey matter	CNhs14071	caudate nucleus, newborn, donor10223
UBERON:0001017	Central Nervous System	UBERON:0003528	brain grey matter	CNhs14073	occipital cortex, newborn, donor10223
UBERON:0001017	Central Nervous System	UBERON:0003528	brain grey matter	CNhs14074	parietal lobe, newborn, donor10223
UBERON:0001017	Central Nervous System	UBERON:0003528	brain grey matter	CNhs14076	substantia nigra, newborn, donor10223
UBERON:0001017	Central Nervous System	UBERON:0003528	brain grey matter	CNhs14078	amygdala, newborn, donor10223
UBERON:0001017	Central Nervous System	UBERON:0003528	brain grey matter	CNhs14080	locus coeruleus, newborn, donor10223
UBERON:0001017	Central Nervous System	UBERON:0003528	brain grey matter	CNhs14081	hippocampus, newborn, donor10223
UBERON:0001017	Central Nervous System	UBERON:0003528	brain grey matter	CNhs14082	globus pallidus, newborn, donor10223
UBERON:0001017	Central Nervous System	UBERON:0003528	brain grey matter	CNhs14083	putamen, newborn, donor10223
UBERON:0001017	Central Nervous System	UBERON:0003528	brain grey matter	CNhs14549	globus pallidus, adult, donor10258
UBERON:0001017	Central Nervous System	UBERON:0003528	brain grey matter	CNhs14221	medial frontal gyrus, adult, donor10258
UBERON:0001017	Central Nervous System	UBERON:0003528	brain grey matter	CNhs14224	substantia nigra, adult, donor10258
UBERON:0001017	Central Nervous System	UBERON:0003528	brain grey matter	Cnhs14225	putamen, adult, donor10258
UBERON:0001017	Central Nervous System	UBERON:0003528	brain grey matter	CNhs14226	parietal cortex, adult, donor10258
UBERON:0001017	Central Nervous System	UBERON:0003528	brain grey matter	CNhs14227	hippocampus, adult, donor10258
UBERON:0001017	Central Nervous System	UBERON:0003528	brain grey matter	CNhs14550	locus coeruleus, adult, donor10258
UBERON:0001017	Central Nervous System	UBERON:0003528	brain grey matter	Cnhs14229	medial temporal gyrus, adult, donor10258
UBERON:0001017	Central Nervous System	UBERON:0003528	brain grey matter	CNhs10864	Astrocyte - cerebral cortex, donor1
UBERON:0001017	Central Nervous System	UBERON:0003528	brain grey matter	CNhs11960	Astrocyte - cerebral cortex, donor2
UBERON:0001017	Central Nervous System	UBERON:0003528	brain grey matter	CNhs12005	Astrocyte - cerebral cortex, donor3
UBERON:0001017	Central Nervous System	UBERON:0003544	brain white matter	CNhs10649	corpus callosum, adult, pool1
UBERON:0001017	Central Nervous System	UBERON:0004086	brain ventricle	CNhs11319	Fibroblast - Choroid Plexus, donor1
UBERON:0001017	Central Nervous System	UBERON:0004086	brain ventricle	CNhs12344	Fibroblast - Choroid Plexus, donor2
UBERON:0001017	Central Nervous System	UBERON:0004086	brain ventricle	CNhs12620	Fibroblast - Choroid Plexus, donor3
UBERON:0001017	Central Nervous System	UBERON:0004733	segmental subdivision of hindbrain	CNhs10640	pons, adult, pool1
UBERON:0001017	Central Nervous System	UBERON:0004733	segmental subdivision of hindbrain	CNhs10645	medulla oblongata, adult, pool1
UBERON:0001017	Central Nervous System	UBERON:0004733	segmental subdivision of hindbrain	CNhs11795	cerebellum, adult, pool1
UBERON:0001017	Central Nervous System	UBERON:0004733	segmental subdivision of hindbrain	CNhs12315	medulla oblongata, adult, donor10252
UBERON:0001017	Central Nervous System	UBERON:0004733	segmental subdivision of hindbrain	CNhs12322	locus coeruleus, adult, donor10252
UBERON:0001017	Central Nervous System	UBERON:0004733	segmental subdivision of hindbrain	CNhs12323	cerebellum, adult, donor10252
UBERON:0001017	Central Nervous System	UBERON:0004733	segmental subdivision of hindbrain	CNhs13799	cerebellum - adult, donor10196
UBERON:0001017	Central Nervous System	UBERON:0004733	segmental subdivision of hindbrain	CNhs13800	medulla oblongata - adult, donor10196
UBERON:0001017	Central Nervous System	UBERON:0004733	segmental subdivision of hindbrain	CNhs13808	locus coeruleus - adult, donor10196
UBERON:0001017	Central Nervous System	UBERON:0004733	segmental subdivision of hindbrain	CNhs14075	cerebellum, newborn, donor10223
UBERON:0001017	Central Nervous System	UBERON:0004733	segmental subdivision of hindbrain	CNhs14079	medulla oblongata, newborn, donor10223
UBERON:0001017	Central Nervous System	UBERON:0004733	segmental subdivision of hindbrain	CNhs14080	locus coeruleus, newborn, donor10223
UBERON:0001017	Central Nervous System	UBERON:0004733	segmental subdivision of hindbrain	CNhs14550	locus coeruleus, adult, donor10258
UBERON:0001017	Central Nervous System	UBERON:0004733	segmental subdivision of hindbrain	CNhs11321	Astrocyte - cerebellum, donor1
UBERON:0001017	Central Nervous System	UBERON:0004733	segmental subdivision of hindbrain	CNhs12081	Astrocyte - cerebellum, donor2
UBERON:0001017	Central Nervous System	UBERON:0004733	segmental subdivision of hindbrain	CNhs12117	Astrocyte - cerebellum, donor3
UBERON:0001017	Central Nervous System	UBERON:0005281	ventricular system of central nervous system	CNhs11319	Fibroblast - Choroid Plexus, donor1
UBERON:0001017	Central Nervous System	UBERON:0005281	ventricular system of central nervous system	CNhs12344	Fibroblast - Choroid Plexus, donor2
UBERON:0001017	Central Nervous System	UBERON:0005281	ventricular system of central nervous system	CNhs12620	Fibroblast - Choroid Plexus, donor3

UBERON:0001017	Central Nervous System	UBERON:0005282	ventricular system of brain	CNhs11319	Fibroblast - Choroid Plexus, donor1
UBERON:0001017	Central Nervous System	UBERON:0005282	ventricular system of brain	CNhs12344	Fibroblast - Choroid Plexus, donor2
UBERON:0001017	Central Nervous System	UBERON:0005282	ventricular system of brain	CNhs12620	Fibroblast - Choroid Plexus, donor3
UBERON:0001017	Central Nervous System	UBERON:0005284	brain vasculature	CNhs10863	Smooth Muscle Cells - Brain Vascular, donor1
UBERON:0001017	Central Nervous System	UBERON:0005284	brain vasculature	CNhs11900	Smooth Muscle Cells - Brain Vascular, donor2
UBERON:0001017	Central Nervous System	UBERON:0005284	brain vasculature	CNhs12004	Smooth Muscle Cells - Brain Vascular, donor3
UBERON:0001017	Central Nervous System	UBERON:0005284	brain vasculature	CNhs11319	Fibroblast - Choroid Plexus, donor1
UBERON:0001017	Central Nervous System	UBERON:0005284	brain vasculature	CNhs12344	Fibroblast - Choroid Plexus, donor2
UBERON:0001017	Central Nervous System	UBERON:0005284	brain vasculature	CNhs12620	Fibroblast - Choroid Plexus, donor3
UBERON:0001017	Central Nervous System	UBERON:0005290	myelencephalon	CNhs10645	medulla oblongata, adult, pool1
UBERON:0001017	Central Nervous System	UBERON:0005290	myelencephalon	CNhs12315	medulla oblongata, adult, donor10252
UBERON:0001017	Central Nervous System	UBERON:0005290	myelencephalon	CNhs13800	medulla oblongata - adult, donor10196
UBERON:0001017	Central Nervous System	UBERON:0005290	myelencephalon	CNhs14079	medulla oblongata, newborn, donor10223
UBERON:0001017	Central Nervous System	UBERON:0005340	dorsal telencephalic commissure	CNhs10649	corpus callosum, adult, pool1
UBERON:0001017	Central Nervous System	UBERON:0005382	dorsal striatum	CNhs13912	putamen, adult, donor10252
UBERON:0001017	Central Nervous System	UBERON:0005382	dorsal striatum	CNhs12321	caudate nucleus, adult, donor10252
UBERON:0001017	Central Nervous System	UBERON:0005382	dorsal striatum	CNhs12324	putamen, adult, donor10196
UBERON:0001017	Central Nervous System	UBERON:0005382	dorsal striatum	CNhs13802	caudate nucleus - adult, donor10196
UBERON:0001017	Central Nervous System	UBERON:0005382	dorsal striatum	CNhs14071	caudate nucleus, newborn, donor10223
UBERON:0001017	Central Nervous System	UBERON:0005382	dorsal striatum	CNhs14083	putamen, newborn, donor10223
UBERON:0001017	Central Nervous System	UBERON:0005382	dorsal striatum	Cnhs14225	putamen, adult, donor10258
UBERON:0001017	Central Nervous System	UBERON:0005383	caudate-putamen	CNhs13912	putamen, adult, donor10252
UBERON:0001017	Central Nervous System	UBERON:0005383	caudate-putamen	CNhs12321	caudate nucleus, adult, donor10252
UBERON:0001017	Central Nervous System	UBERON:0005383	caudate-putamen	CNhs12324	putamen, adult, donor10196
UBERON:0001017	Central Nervous System	UBERON:0005383	caudate-putamen	CNhs13802	caudate nucleus - adult, donor10196
UBERON:0001017	Central Nervous System	UBERON:0005383	caudate-putamen	CNhs14071	caudate nucleus, newborn, donor10223
UBERON:0001017	Central Nervous System	UBERON:0005383	caudate-putamen	CNhs14083	putamen, newborn, donor10223
UBERON:0001017	Central Nervous System	UBERON:0005383	caudate-putamen	Cnhs14225	putamen, adult, donor10258
UBERON:0001017	Central Nervous System	UBERON:0005403	ventral striatum	CNhs10644	nucleus accumbens, adult, pool1
UBERON:0001017	Central Nervous System	UBERON:0005408	circumventricular organ	CNhs12228	pineal gland, adult, donor10252
UBERON:0001017	Central Nervous System	UBERON:0005408	circumventricular organ	CNhs13804	pineal gland - adult, donor10196
UBERON:0001017	Central Nervous System	UBERON:0005408	circumventricular organ	CNhs14230	pineal gland, adult, donor10258
UBERON:0001017	Central Nervous System	UBERON:0005970	brain commissure	CNhs10649	corpus callosum, adult, pool1
UBERON:0001017	Central Nervous System	UBERON:0006331	brainstem nucleus	CNhs12322	locus coeruleus, adult, donor10252
UBERON:0001017	Central Nervous System	UBERON:0006331	brainstem nucleus	CNhs13808	locus coeruleus - adult, donor10196
UBERON:0001017	Central Nervous System	UBERON:0006331	brainstem nucleus	CNhs14080	locus coeruleus, newborn, donor10223
UBERON:0001017	Central Nervous System	UBERON:0006331	brainstem nucleus	CNhs14550	locus coeruleus, adult, donor10258
UBERON:0001017	Central Nervous System	UBERON:0006514	pallidum	CNhs12319	globus pallidus, adult, donor10252
UBERON:0001017	Central Nervous System	UBERON:0006514	pallidum	CNhs13801	globus pallidus - adult, donor10196
UBERON:0001017	Central Nervous System	UBERON:0006514	pallidum	CNhs14082	globus pallidus, newborn, donor10223
UBERON:0001017	Central Nervous System	UBERON:0006514	pallidum	CNhs14549	globus pallidus, adult, donor10258
UBERON:0001017	Central Nervous System	UBERON:0007190	paracentral gyrus	CNhs10642	paracentral gyrus, adult, pool1
UBERON:0001017	Central Nervous System	UBERON:0007245	nuclear complex of neuraxis	CNhs10644	nucleus accumbens, adult, pool1
UBERON:0001017	Central Nervous System	UBERON:0007245	nuclear complex of neuraxis	CNhs12311	amygdala, adult, donor10252
UBERON:0001017	Central Nervous System	UBERON:0007245	nuclear complex of neuraxis	CNhs13912	putamen, adult, donor10252
UBERON:0001017	Central Nervous System	UBERON:0007245	nuclear complex of neuraxis	CNhs12318	substantia nigra, adult, donor10252
UBERON:0001017	Central Nervous System	UBERON:0007245	nuclear complex of neuraxis	CNhs12319	globus pallidus, adult, donor10252
UBERON:0001017	Central Nervous System	UBERON:0007245	nuclear complex of neuraxis	CNhs12321	caudate nucleus, adult, donor10252
UBERON:0001017	Central Nervous System	UBERON:0007245	nuclear complex of neuraxis	CNhs13793	amygdala - adult, donor10196
UBERON:0001017	Central Nervous System	UBERON:0007245	nuclear complex of neuraxis	CNhs13801	globus pallidus - adult, donor10196
UBERON:0001017	Central Nervous System	UBERON:0007245	nuclear complex of neuraxis	CNhs12324	putamen, adult, donor10196
UBERON:0001017	Central Nervous System	UBERON:0007245	nuclear complex of neuraxis	CNhs13802	caudate nucleus - adult, donor10196
UBERON:0001017	Central Nervous System	UBERON:0007245	nuclear complex of neuraxis	CNhs13803	substantia nigra - adult, donor10196
UBERON:0001017	Central Nervous System	UBERON:0007245	nuclear complex of neuraxis	CNhs14071	caudate nucleus, newborn, donor10223
UBERON:0001017	Central Nervous System	UBERON:0007245	nuclear complex of neuraxis	CNhs14076	substantia nigra, newborn, donor10223
UBERON:0001017	Central Nervous System	UBERON:0007245	nuclear complex of neuraxis	CNhs14078	amygdala, newborn, donor10223
UBERON:0001017	Central Nervous System	UBERON:0007245	nuclear complex of neuraxis	CNhs14082	globus pallidus, newborn, donor10223
UBERON:0001017	Central Nervous System	UBERON:0007245	nuclear complex of neuraxis	CNhs14083	putamen, newborn, donor10223
UBERON:0001017	Central Nervous System	UBERON:0007245	nuclear complex of neuraxis	CNhs14549	globus pallidus, adult, donor10258
UBERON:0001017	Central Nervous System	UBERON:0007245	nuclear complex of neuraxis	CNhs14224	substantia nigra, adult, donor10258
UBERON:0001017	Central Nervous System	UBERON:0007245	nuclear complex of neuraxis	Cnhs14225	putamen, adult, donor10258
UBERON:0001017	Central Nervous System	UBERON:0009661	midbrain nucleus	CNhs12318	substantia nigra, adult, donor10252
UBERON:0001017	Central Nervous System	UBERON:0009661	midbrain nucleus	CNhs13803	substantia nigra - adult, donor10196
UBERON:0001017	Central Nervous System	UBERON:0009661	midbrain nucleus	CNhs14076	substantia nigra, newborn, donor10223
UBERON:0001017	Central Nervous System	UBERON:0009661	midbrain nucleus	CNhs14224	substantia nigra, adult, donor10258
UBERON:0001017	Central Nervous System	UBERON:0009662	hindbrain nucleus	CNhs12322	locus coeruleus, adult, donor10252
UBERON:0001017	Central Nervous System	UBERON:0009662	hindbrain nucleus	CNhs13808	locus coeruleus - adult, donor10196
UBERON:0001017	Central Nervous System	UBERON:0009662	hindbrain nucleus	CNhs14080	locus coeruleus, newborn, donor10223
UBERON:0001017	Central Nervous System	UBERON:0009662	hindbrain nucleus	CNhs14550	locus coeruleus, adult, donor10258
UBERON:0001017	Central Nervous System	UBERON:0009663	telencephalic nucleus	CNhs10644	nucleus accumbens, adult, pool1
UBERON:0001017	Central Nervous System	UBERON:0009663	telencephalic nucleus	CNhs13912	putamen, adult, donor10252
UBERON:0001017	Central Nervous System	UBERON:0009663	telencephalic nucleus	CNhs12318	substantia nigra, adult, donor10252
UBERON:0001017	Central Nervous System	UBERON:0009663	telencephalic nucleus	CNhs12319	globus pallidus, adult, donor10252
UBERON:0001017	Central Nervous System	UBERON:0009663	telencephalic nucleus	CNhs12321	caudate nucleus, adult, donor10252
UBERON:0001017	Central Nervous System	UBERON:0009663	telencephalic nucleus	CNhs13801	globus pallidus - adult, donor10196
UBERON:0001017	Central Nervous System	UBERON:0009663	telencephalic nucleus	CNhs12324	putamen, adult, donor10196
UBERON:0001017	Central Nervous System	UBERON:0009663	telencephalic nucleus	CNhs13802	caudate nucleus - adult, donor10196
UBERON:0001017	Central Nervous System	UBERON:0009663	telencephalic nucleus	CNhs13803	substantia nigra - adult, donor10196
UBERON:0001017	Central Nervous System	UBERON:0009663	telencephalic nucleus	CNhs14071	caudate nucleus, newborn, donor10223
UBERON:0001017	Central Nervous System	UBERON:0009663	telencephalic nucleus	CNhs14076	substantia nigra, newborn, donor10223
UBERON:0001017	Central Nervous System	UBERON:0009663	telencephalic nucleus	CNhs14082	globus pallidus, newborn, donor10223
UBERON:0001017	Central Nervous System	UBERON:0009663	telencephalic nucleus	CNhs14083	putamen, newborn, donor10223
UBERON:0001017	Central Nervous System	UBERON:0009663	telencephalic nucleus	CNhs14549	globus pallidus, adult, donor10258
UBERON:0001017	Central Nervous System	UBERON:0009663	telencephalic nucleus	CNhs14224	substantia nigra, adult, donor10258
UBERON:0001017	Central Nervous System	UBERON:0009663	telencephalic nucleus	Cnhs14225	putamen, adult, donor10258
UBERON:0001017	Central Nervous System	UBERON:0009899	pole of cerebral hemisphere	CNhs10643	occipital pole, adult, pool1
UBERON:0001017	Central Nervous System	UBERON:0010009	aggregate regional part of brain	CNhs10644	nucleus accumbens, adult, pool1
UBERON:0001017	Central Nervous System	UBERON:0010009	aggregate regional part of brain	CNhs12311	amygdala, adult, donor10252
UBERON:0001017	Central Nervous System	UBERON:0010009	aggregate regional part of brain	CNhs13912	putamen, adult, donor10252
UBERON:0001017	Central Nervous System	UBERON:0010009	aggregate regional part of brain	CNhs12318	substantia nigra, adult, donor10252
UBERON:0001017	Central Nervous System	UBERON:0010009	aggregate regional part of brain	CNhs12319	globus pallidus, adult, donor10252
UBERON:0001017	Central Nervous System	UBERON:0010009	aggregate regional part of brain	CNhs12321	caudate nucleus, adult, donor10252
UBERON:0001017	Central Nervous System	UBERON:0010009	aggregate regional part of brain	CNhs13793	amygdala - adult, donor10196
UBERON:0001017	Central Nervous System	UBERON:0010009	aggregate regional part of brain	CNhs13801	globus pallidus - adult, donor10196
UBERON:0001017	Central Nervous System	UBERON:0010009	aggregate regional part of brain	CNhs12324	putamen, adult, donor10196
UBERON:0001017	Central Nervous System	UBERON:0010009	aggregate regional part of brain	CNhs13802	caudate nucleus - adult, donor10196
UBERON:0001017	Central Nervous System	UBERON:0010009	aggregate regional part of brain	CNhs13803	substantia nigra - adult, donor10196
UBERON:0001017	Central Nervous System	UBERON:0010009	aggregate regional part of brain	CNhs14071	caudate nucleus, newborn, donor10223

UBERON:0001017	Central Nervous System	UBERON:0010009	aggregate regional part of brain	CNhs14076	substantia nigra, newborn, donor10223
UBERON:0001017	Central Nervous System	UBERON:0010009	aggregate regional part of brain	CNhs14078	amygdala, newborn, donor10223
UBERON:0001017	Central Nervous System	UBERON:0010009	aggregate regional part of brain	CNhs14082	globus pallidus, newborn, donor10223
UBERON:0001017	Central Nervous System	UBERON:0010009	aggregate regional part of brain	CNhs14083	putamen, newborn, donor10223
UBERON:0001017	Central Nervous System	UBERON:0010009	aggregate regional part of brain	CNhs14549	globus pallidus, adult, donor10258
UBERON:0001017	Central Nervous System	UBERON:0010009	aggregate regional part of brain	CNhs14224	substantia nigra, adult, donor10258
UBERON:0001017	Central Nervous System	UBERON:0010009	aggregate regional part of brain	Cnhs14225	putamen, adult, donor10258
UBERON:0001017	Central Nervous System	UBERON:0010011	collection of basal ganglia	CNhs10644	nucleus accumbens, adult, pool1
UBERON:0001017	Central Nervous System	UBERON:0010011	collection of basal ganglia	CNhs12311	amygdala, adult, donor10252
UBERON:0001017	Central Nervous System	UBERON:0010011	collection of basal ganglia	CNhs13912	putamen, adult, donor10252
UBERON:0001017	Central Nervous System	UBERON:0010011	collection of basal ganglia	CNhs12318	substantia nigra, adult, donor10252
UBERON:0001017	Central Nervous System	UBERON:0010011	collection of basal ganglia	CNhs12319	globus pallidus, adult, donor10252
UBERON:0001017	Central Nervous System	UBERON:0010011	collection of basal ganglia	CNhs12321	caudate nucleus, adult, donor10252
UBERON:0001017	Central Nervous System	UBERON:0010011	collection of basal ganglia	CNhs13793	amygdala - adult, donor10196
UBERON:0001017	Central Nervous System	UBERON:0010011	collection of basal ganglia	CNhs13801	globus pallidus - adult, donor10196
UBERON:0001017	Central Nervous System	UBERON:0010011	collection of basal ganglia	CNhs12324	putamen, adult, donor10196
UBERON:0001017	Central Nervous System	UBERON:0010011	collection of basal ganglia	CNhs13802	caudate nucleus - adult, donor10196
UBERON:0001017	Central Nervous System	UBERON:0010011	collection of basal ganglia	CNhs13803	substantia nigra - adult, donor10196
UBERON:0001017	Central Nervous System	UBERON:0010011	collection of basal ganglia	CNhs14071	caudate nucleus, newborn, donor10223
UBERON:0001017	Central Nervous System	UBERON:0010011	collection of basal ganglia	CNhs14076	substantia nigra, newborn, donor10223
UBERON:0001017	Central Nervous System	UBERON:0010011	collection of basal ganglia	CNhs14078	amygdala, newborn, donor10223
UBERON:0001017	Central Nervous System	UBERON:0010011	collection of basal ganglia	CNhs14082	globus pallidus, newborn, donor10223
UBERON:0001017	Central Nervous System	UBERON:0010011	collection of basal ganglia	CNhs14083	putamen, newborn, donor10223
UBERON:0001017	Central Nervous System	UBERON:0010011	collection of basal ganglia	CNhs14549	globus pallidus, adult, donor10258
UBERON:0001017	Central Nervous System	UBERON:0010011	collection of basal ganglia	CNhs14224	substantia nigra, adult, donor10258
UBERON:0001017	Central Nervous System	UBERON:0010011	collection of basal ganglia	Cnhs14225	putamen, adult, donor10258
UBERON:0001017	Central Nervous System	UBERON:0010134	secretory circumventricular organ	CNhs12228	pineal gland, adult, donor10252
UBERON:0001017	Central Nervous System	UBERON:0010134	secretory circumventricular organ	CNhs13804	pineal gland - adult, donor10196
UBERON:0001017	Central Nervous System	UBERON:0010134	secretory circumventricular organ	CNhs14230	pineal gland, adult, donor10258
UBERON:0001017	Central Nervous System	UBERON:0010225	thalamic complex	CNhs12314	thalamus, adult, donor10252
UBERON:0001017	Central Nervous System	UBERON:0010225	thalamic complex	CNhs13794	thalamus - adult, donor10196
UBERON:0001017	Central Nervous System	UBERON:0010225	thalamic complex	CNhs14084	thalamus, newborn, donor10223
UBERON:0001017	Central Nervous System	UBERON:0010225	thalamic complex	Cnhs14223	thalamus, adult, donor10258
UBERON:0001017	Central Nervous System	UBERON:0010743	meningeal cluster	CNhs10648	dura mater, adult, donor1
UBERON:0001017	Central Nervous System	UBERON:0010743	meningeal cluster	CNhs12840	cerebral meninges, adult
UBERON:0001017	Central Nervous System	UBERON:0011215	cell part cluster of neuraxis	CNhs10649	corpus callosum, adult, pool1
UBERON:0002204	Musculoskeletal System	UBERON:0000043	tendon	CNhs13435	achilles tendon, donor2
UBERON:0002204	Musculoskeletal System	UBERON:0000043	tendon	CNhs12639	tenocyte, donor1
UBERON:0002204	Musculoskeletal System	UBERON:0000043	tendon	CNhs12640	tenocyte, donor2
UBERON:0002204	Musculoskeletal System	UBERON:0000075	subdivision of skeletal system	CNhs12611	olfactory region, adult
UBERON:0002204	Musculoskeletal System	UBERON:0000075	subdivision of skeletal system	CNhs10846	Mesenchymal Stem Cells - Vertebral, donor1
UBERON:0002204	Musculoskeletal System	UBERON:0000075	subdivision of skeletal system	CNhs12493	Fibroblast - Periodontal Ligament, donor4 (PL29)
UBERON:0002204	Musculoskeletal System	UBERON:0000075	subdivision of skeletal system	CNhs10867	Fibroblast - Periodontal Ligament, donor1
UBERON:0002204	Musculoskeletal System	UBERON:0000075	subdivision of skeletal system	CNhs10876	Anulus Pulposus Cell, donor1
UBERON:0002204	Musculoskeletal System	UBERON:0000075	subdivision of skeletal system	CNhs10881	Nucleus Pulposus Cell, donor1
UBERON:0002204	Musculoskeletal System	UBERON:0000075	subdivision of skeletal system	CNhs11953	Fibroblast - Periodontal Ligament, donor5 (PL30)
UBERON:0002204	Musculoskeletal System	UBERON:0000075	subdivision of skeletal system	CNhs11962	Fibroblast - Periodontal Ligament, donor2
UBERON:0002204	Musculoskeletal System	UBERON:0000075	subdivision of skeletal system	CNhs11996	Fibroblast - Periodontal Ligament, donor6 (PLH3)
UBERON:0002204	Musculoskeletal System	UBERON:0000075	subdivision of skeletal system	CNhs11907	Fibroblast - Periodontal Ligament, donor3
UBERON:0002204	Musculoskeletal System	UBERON:0000075	subdivision of skeletal system	CNhs12019	Nucleus Pulposus Cell, donor2
UBERON:0002204	Musculoskeletal System	UBERON:0000075	subdivision of skeletal system	CNhs12063	Nucleus Pulposus Cell, donor3
UBERON:0002204	Musculoskeletal System	UBERON:0000075	subdivision of skeletal system	CNhs12064	Anulus Pulposus Cell, donor2
UBERON:0002204	Musculoskeletal System	UBERON:0000075	subdivision of skeletal system	CNhs13550	Mallassez-derived cells, donor2
UBERON:0002204	Musculoskeletal System	UBERON:0000075	subdivision of skeletal system	CNhs13551	Mallassez-derived cells, donor3
UBERON:0002204	Musculoskeletal System	UBERON:0000383	musculature of body	CNhs10629	skeletal muscle, adult, pool1
UBERON:0002204	Musculoskeletal System	UBERON:0000383	musculature of body	CNhs10630	small intestine, adult, pool1
UBERON:0002204	Musculoskeletal System	UBERON:0000383	musculature of body	CNhs11755	smooth muscle, adult, pool1
UBERON:0002204	Musculoskeletal System	UBERON:0000383	musculature of body	CNhs11776	skeletal muscle, fetal, donor1
UBERON:0002204	Musculoskeletal System	UBERON:0000383	musculature of body	CNhs11779	diaphragm, fetal, donor1
UBERON:0002204	Musculoskeletal System	UBERON:0000383	musculature of body	CNhs13444	eye - muscle inferior rectus, donor1
UBERON:0002204	Musculoskeletal System	UBERON:0000383	musculature of body	CNhs13454	skeletal muscle - soleus muscle, donor1
UBERON:0002204	Musculoskeletal System	UBERON:0000383	musculature of body	CNhs13435	achilles tendon, donor2
UBERON:0002204	Musculoskeletal System	UBERON:0000383	musculature of body	CNhs13441	eye - muscle superior, donor2
UBERON:0002204	Musculoskeletal System	UBERON:0000383	musculature of body	CNhs13442	eye - muscle lateral, donor2
UBERON:0002204	Musculoskeletal System	UBERON:0000383	musculature of body	CNhs13443	eye - muscle medial, donor2
UBERON:0002204	Musculoskeletal System	UBERON:0000383	musculature of body	CNhs10838	Smooth Muscle Cells - Aortic, donor0
UBERON:0002204	Musculoskeletal System	UBERON:0000383	musculature of body	CNhs10868	Smooth Muscle Cells - Colonic, donor1
UBERON:0002204	Musculoskeletal System	UBERON:0000383	musculature of body	CNhs10869	Skeletal Muscle Satellite Cells, donor1
UBERON:0002204	Musculoskeletal System	UBERON:0000383	musculature of body	CNhs10870	Myoblast, donor1
UBERON:0002204	Musculoskeletal System	UBERON:0000383	musculature of body	CNhs11083	Skeletal Muscle Cells, donor1
UBERON:0002204	Musculoskeletal System	UBERON:0000383	musculature of body	CNhs11084	Skeletal muscle cells differentiated into Myotubes - multinucleated, donor1
UBERON:0002204	Musculoskeletal System	UBERON:0000383	musculature of body	CNhs11085	Smooth Muscle Cells - Aortic, donor1
UBERON:0002204	Musculoskeletal System	UBERON:0000383	musculature of body	CNhs11963	Smooth Muscle Cells - Colonic, donor2
UBERON:0002204	Musculoskeletal System	UBERON:0000383	musculature of body	CNhs11964	Skeletal Muscle Satellite Cells, donor2
UBERON:0002204	Musculoskeletal System	UBERON:0000383	musculature of body	CNhs11965	Myoblast, donor2
UBERON:0002204	Musculoskeletal System	UBERON:0000383	musculature of body	CNhs11983	Skeletal Muscle Cells, donor2
UBERON:0002204	Musculoskeletal System	UBERON:0000383	musculature of body	CNhs11984	Skeletal muscle cells differentiated into Myotubes - multinucleated, donor2
UBERON:0002204	Musculoskeletal System	UBERON:0000383	musculature of body	CNhs11305	Smooth Muscle Cells - Aortic, donor2
UBERON:0002204	Musculoskeletal System	UBERON:0000383	musculature of body	CNhs12007	Smooth Muscle Cells - Colonic, donor3
UBERON:0002204	Musculoskeletal System	UBERON:0000383	musculature of body	CNhs12008	Skeletal Muscle Satellite Cells, donor3
UBERON:0002204	Musculoskeletal System	UBERON:0000383	musculature of body	CNhs11908	Myoblast, donor3
UBERON:0002204	Musculoskeletal System	UBERON:0000383	musculature of body	CNhs12040	Skeletal Muscle Cells, donor3
UBERON:0002204	Musculoskeletal System	UBERON:0000383	musculature of body	CNhs12041	Skeletal muscle cells differentiated into Myotubes - multinucleated, donor3
UBERON:0002204	Musculoskeletal System	UBERON:0000383	musculature of body	CNhs11309	Smooth Muscle Cells - Aortic, donor3
UBERON:0002204	Musculoskeletal System	UBERON:0000383	musculature of body	CNhs12053	Skeletal Muscle Cells, donor4
UBERON:0002204	Musculoskeletal System	UBERON:0000383	musculature of body	CNhs12056	Skeletal Muscle Cells, donor5
UBERON:0002204	Musculoskeletal System	UBERON:0000383	musculature of body	CNhs12060	Skeletal Muscle Cells, donor6
UBERON:0002204	Musculoskeletal System	UBERON:0000383	musculature of body	CNhs14183	Smooth muscle cells - airway, asthmatic, donor1
UBERON:0002204	Musculoskeletal System	UBERON:0000383	musculature of body	CNhs14184	Smooth muscle cells - airway, asthmatic, donor2
UBERON:0002204	Musculoskeletal System	UBERON:0000383	musculature of body	CNhs14186	Smooth muscle cells - airway, asthmatic, donor3
UBERON:0002204	Musculoskeletal System	UBERON:0000383	musculature of body	CNhs14187	Smooth muscle cells - airway, asthmatic, donor4
UBERON:0002204	Musculoskeletal System	UBERON:0000383	musculature of body	CNhs14188	Smooth muscle cells - airway, asthmatic, donor5
UBERON:0002204	Musculoskeletal System	UBERON:0000383	musculature of body	CNhs14189	Smooth muscle cells - airway, asthmatic, donor6
UBERON:0002204	Musculoskeletal System	UBERON:0000383	musculature of body	CNhs14190	Smooth muscle cells - airway, control, donor1
UBERON:0002204	Musculoskeletal System	UBERON:0000383	musculature of body	CNhs14191	Smooth muscle cells - airway, control, donor2
UBERON:0002204	Musculoskeletal System	UBERON:0000383	musculature of body	CNhs14192	Smooth muscle cells - airway, control, donor3
UBERON:0002204	Musculoskeletal System	UBERON:0000383	musculature of body	CNhs14193	Smooth muscle cells - airway, control, donor4
UBERON:0002204	Musculoskeletal System	UBERON:0000982	skeletal joint	CNhs13439	cruciate ligament, donor2

UBERON:0002204	Musculoskeletal System	UBERON:0000982	skeletal joint	CNhs10876	Anulus Pulposus Cell, donor1
UBERON:0002204	Musculoskeletal System	UBERON:0000982	skeletal joint	CNhs10881	Nucleus Pulposus Cell, donor1
UBERON:0002204	Musculoskeletal System	UBERON:0000982	skeletal joint	CNhs11068	Synoviocyte, donor1
UBERON:0002204	Musculoskeletal System	UBERON:0000982	skeletal joint	CNhs11992	Synoviocyte, donor2
UBERON:0002204	Musculoskeletal System	UBERON:0000982	skeletal joint	CNhs12019	Nucleus Pulposus Cell, donor2
UBERON:0002204	Musculoskeletal System	UBERON:0000982	skeletal joint	CNhs12050	Synoviocyte, donor3
UBERON:0002204	Musculoskeletal System	UBERON:0000982	skeletal joint	CNhs12063	Nucleus Pulposus Cell, donor3
UBERON:0002204	Musculoskeletal System	UBERON:0000982	skeletal joint	CNhs12064	Anulus Pulposus Cell, donor2
UBERON:0002204	Musculoskeletal System	UBERON:0001015	musculature	CNhs10629	skeletal muscle, adult, pool1
UBERON:0002204	Musculoskeletal System	UBERON:0001015	musculature	CNhs10630	small intestine, adult, pool1
UBERON:0002204	Musculoskeletal System	UBERON:0001015	musculature	CNhs11755	smooth muscle, adult, pool1
UBERON:0002204	Musculoskeletal System	UBERON:0001015	musculature	CNhs11776	skeletal muscle, fetal, donor1
UBERON:0002204	Musculoskeletal System	UBERON:0001015	musculature	CNhs11779	diaphragm, fetal, donor1
UBERON:0002204	Musculoskeletal System	UBERON:0001015	musculature	CNhs13444	eye - muscle inferior rectus, donor1
UBERON:0002204	Musculoskeletal System	UBERON:0001015	musculature	CNhs13454	skeletal muscle - soleus muscle, donor1
UBERON:0002204	Musculoskeletal System	UBERON:0001015	musculature	CNhs13441	eye - muscle superior, donor2
UBERON:0002204	Musculoskeletal System	UBERON:0001015	musculature	CNhs13442	eye - muscle lateral, donor2
UBERON:0002204	Musculoskeletal System	UBERON:0001015	musculature	CNhs13443	eye - muscle medial, donor2
UBERON:0002204	Musculoskeletal System	UBERON:0001015	musculature	CNhs10838	Smooth Muscle Cells - Aortic, donor0
UBERON:0002204	Musculoskeletal System	UBERON:0001015	musculature	CNhs10868	Smooth Muscle Cells - Colonic, donor1
UBERON:0002204	Musculoskeletal System	UBERON:0001015	musculature	CNhs10869	Skeletal Muscle Satellite Cells, donor1
UBERON:0002204	Musculoskeletal System	UBERON:0001015	musculature	CNhs10870	Myoblast, donor1
UBERON:0002204	Musculoskeletal System	UBERON:0001015	musculature	CNhs11083	Skeletal Muscle Cells, donor1
UBERON:0002204	Musculoskeletal System	UBERON:0001015	musculature	CNhs11084	Skeletal muscle cells differentiated into Myotubes - multinucleated, donor1
UBERON:0002204	Musculoskeletal System	UBERON:0001015	musculature	CNhs11085	Smooth Muscle Cells - Aortic, donor1
UBERON:0002204	Musculoskeletal System	UBERON:0001015	musculature	CNhs11963	Smooth Muscle Cells - Colonic, donor2
UBERON:0002204	Musculoskeletal System	UBERON:0001015	musculature	CNhs11964	Skeletal Muscle Satellite Cells, donor2
UBERON:0002204	Musculoskeletal System	UBERON:0001015	musculature	CNhs11965	Myoblast, donor2
UBERON:0002204	Musculoskeletal System	UBERON:0001015	musculature	CNhs11983	Skeletal Muscle Cells, donor2
UBERON:0002204	Musculoskeletal System	UBERON:0001015	musculature	CNhs11984	Skeletal muscle cells differentiated into Myotubes - multinucleated, donor2
UBERON:0002204	Musculoskeletal System	UBERON:0001015	musculature	CNhs11305	Smooth Muscle Cells - Aortic, donor2
UBERON:0002204	Musculoskeletal System	UBERON:0001015	musculature	CNhs12007	Smooth Muscle Cells - Colonic, donor3
UBERON:0002204	Musculoskeletal System	UBERON:0001015	musculature	CNhs12008	Skeletal Muscle Satellite Cells, donor3
UBERON:0002204	Musculoskeletal System	UBERON:0001015	musculature	CNhs11908	Myoblast, donor3
UBERON:0002204	Musculoskeletal System	UBERON:0001015	musculature	CNhs12040	Skeletal Muscle Cells, donor3
UBERON:0002204	Musculoskeletal System	UBERON:0001015	musculature	CNhs12041	Skeletal muscle cells differentiated into Myotubes - multinucleated, donor3
UBERON:0002204	Musculoskeletal System	UBERON:0001015	musculature	CNhs11309	Smooth Muscle Cells - Aortic, donor3
UBERON:0002204	Musculoskeletal System	UBERON:0001015	musculature	CNhs12053	Skeletal Muscle Cells, donor4
UBERON:0002204	Musculoskeletal System	UBERON:0001015	musculature	CNhs12056	Skeletal Muscle Cells, donor5
UBERON:0002204	Musculoskeletal System	UBERON:0001015	musculature	CNhs12060	Skeletal Muscle Cells, donor6
UBERON:0002204	Musculoskeletal System	UBERON:0001015	musculature	CNhs14183	Smooth muscle cells - airway, asthmatic, donor1
UBERON:0002204	Musculoskeletal System	UBERON:0001015	musculature	CNhs14184	Smooth muscle cells - airway, asthmatic, donor2
UBERON:0002204	Musculoskeletal System	UBERON:0001015	musculature	CNhs14186	Smooth muscle cells - airway, asthmatic, donor3
UBERON:0002204	Musculoskeletal System	UBERON:0001015	musculature	CNhs14187	Smooth muscle cells - airway, asthmatic, donor4
UBERON:0002204	Musculoskeletal System	UBERON:0001015	musculature	CNhs14188	Smooth muscle cells - airway, asthmatic, donor5
UBERON:0002204	Musculoskeletal System	UBERON:0001015	musculature	CNhs14189	Smooth muscle cells - airway, asthmatic, donor6
UBERON:0002204	Musculoskeletal System	UBERON:0001015	musculature	CNhs14190	Smooth muscle cells - airway, control, donor1
UBERON:0002204	Musculoskeletal System	UBERON:0001015	musculature	CNhs14191	Smooth muscle cells - airway, control, donor2
UBERON:0002204	Musculoskeletal System	UBERON:0001015	musculature	CNhs14192	Smooth muscle cells - airway, control, donor3
UBERON:0002204	Musculoskeletal System	UBERON:0001015	musculature	CNhs14193	Smooth muscle cells - airway, control, donor4
UBERON:0002204	Musculoskeletal System	UBERON:0001066	intervertebral disk	CNhs10876	Anulus Pulposus Cell, donor1
UBERON:0002204	Musculoskeletal System	UBERON:0001066	intervertebral disk	CNhs10881	Nucleus Pulposus Cell, donor1
UBERON:0002204	Musculoskeletal System	UBERON:0001066	intervertebral disk	CNhs12019	Nucleus Pulposus Cell, donor2
UBERON:0002204	Musculoskeletal System	UBERON:0001066	intervertebral disk	CNhs12063	Nucleus Pulposus Cell, donor3
UBERON:0002204	Musculoskeletal System	UBERON:0001066	intervertebral disk	CNhs12064	Anulus Pulposus Cell, donor2
UBERON:0002204	Musculoskeletal System	UBERON:0001103	diaphragm	CNhs11779	diaphragm, fetal, donor1
UBERON:0002204	Musculoskeletal System	UBERON:0001130	vertebral column	CNhs10846	Mesenchymal Stem Cells - Vertebral, donor1
UBERON:0002204	Musculoskeletal System	UBERON:0001130	vertebral column	CNhs10876	Anulus Pulposus Cell, donor1
UBERON:0002204	Musculoskeletal System	UBERON:0001130	vertebral column	CNhs10881	Nucleus Pulposus Cell, donor1
UBERON:0002204	Musculoskeletal System	UBERON:0001130	vertebral column	CNhs12019	Nucleus Pulposus Cell, donor2
UBERON:0002204	Musculoskeletal System	UBERON:0001130	vertebral column	CNhs12063	Nucleus Pulposus Cell, donor3
UBERON:0002204	Musculoskeletal System	UBERON:0001130	vertebral column	CNhs12064	Anulus Pulposus Cell, donor2
UBERON:0002204	Musculoskeletal System	UBERON:0001134	skeletal muscle tissue	CNhs10629	skeletal muscle, adult, pool1
UBERON:0002204	Musculoskeletal System	UBERON:0001134	skeletal muscle tissue	CNhs11776	skeletal muscle, fetal, donor1
UBERON:0002204	Musculoskeletal System	UBERON:0001134	skeletal muscle tissue	CNhs11779	diaphragm, fetal, donor1
UBERON:0002204	Musculoskeletal System	UBERON:0001134	skeletal muscle tissue	CNhs13444	eye - muscle inferior rectus, donor1
UBERON:0002204	Musculoskeletal System	UBERON:0001134	skeletal muscle tissue	CNhs13454	skeletal muscle - soleus muscle, donor1
UBERON:0002204	Musculoskeletal System	UBERON:0001134	skeletal muscle tissue	CNhs10869	Skeletal Muscle Satellite Cells, donor1
UBERON:0002204	Musculoskeletal System	UBERON:0001134	skeletal muscle tissue	CNhs10870	Myoblast, donor1
UBERON:0002204	Musculoskeletal System	UBERON:0001134	skeletal muscle tissue	CNhs11083	Skeletal Muscle Cells, donor1
UBERON:0002204	Musculoskeletal System	UBERON:0001134	skeletal muscle tissue	CNhs11084	Skeletal muscle cells differentiated into Myotubes - multinucleated, donor1
UBERON:0002204	Musculoskeletal System	UBERON:0001134	skeletal muscle tissue	CNhs11964	Skeletal Muscle Satellite Cells, donor2
UBERON:0002204	Musculoskeletal System	UBERON:0001134	skeletal muscle tissue	CNhs11965	Myoblast, donor2
UBERON:0002204	Musculoskeletal System	UBERON:0001134	skeletal muscle tissue	CNhs11983	Skeletal Muscle Cells, donor2
UBERON:0002204	Musculoskeletal System	UBERON:0001134	skeletal muscle tissue	CNhs11984	Skeletal muscle cells differentiated into Myotubes - multinucleated, donor2
UBERON:0002204	Musculoskeletal System	UBERON:0001134	skeletal muscle tissue	CNhs12008	Skeletal Muscle Satellite Cells, donor3
UBERON:0002204	Musculoskeletal System	UBERON:0001134	skeletal muscle tissue	CNhs11908	Myoblast, donor3
UBERON:0002204	Musculoskeletal System	UBERON:0001134	skeletal muscle tissue	CNhs12040	Skeletal Muscle Cells, donor3
UBERON:0002204	Musculoskeletal System	UBERON:0001134	skeletal muscle tissue	CNhs12041	Skeletal muscle cells differentiated into Myotubes - multinucleated, donor3
UBERON:0002204	Musculoskeletal System	UBERON:0001134	skeletal muscle tissue	CNhs12053	Skeletal Muscle Cells, donor4
UBERON:0002204	Musculoskeletal System	UBERON:0001134	skeletal muscle tissue	CNhs12056	Skeletal Muscle Cells, donor5
UBERON:0002204	Musculoskeletal System	UBERON:0001134	skeletal muscle tissue	CNhs12060	Skeletal Muscle Cells, donor6
UBERON:0002204	Musculoskeletal System	UBERON:0001135	smooth muscle tissue	CNhs10630	small intestine, adult, pool1
UBERON:0002204	Musculoskeletal System	UBERON:0001135	smooth muscle tissue	CNhs11755	smooth muscle, adult, pool1
UBERON:0002204	Musculoskeletal System	UBERON:0001135	smooth muscle tissue	CNhs10838	Smooth Muscle Cells - Aortic, donor0
UBERON:0002204	Musculoskeletal System	UBERON:0001135	smooth muscle tissue	CNhs10868	Smooth Muscle Cells - Colonic, donor1
UBERON:0002204	Musculoskeletal System	UBERON:0001135	smooth muscle tissue	CNhs11085	Smooth Muscle Cells - Aortic, donor1
UBERON:0002204	Musculoskeletal System	UBERON:0001135	smooth muscle tissue	CNhs11963	Smooth Muscle Cells - Colonic, donor2
UBERON:0002204	Musculoskeletal System	UBERON:0001135	smooth muscle tissue	CNhs11305	Smooth Muscle Cells - Aortic, donor2
UBERON:0002204	Musculoskeletal System	UBERON:0001135	smooth muscle tissue	CNhs12007	Smooth Muscle Cells - Colonic, donor3
UBERON:0002204	Musculoskeletal System	UBERON:0001135	smooth muscle tissue	CNhs11309	Smooth Muscle Cells - Aortic, donor3
UBERON:0002204	Musculoskeletal System	UBERON:0001135	smooth muscle tissue	CNhs14183	Smooth muscle cells - airway, asthmatic, donor1
UBERON:0002204	Musculoskeletal System	UBERON:0001135	smooth muscle tissue	CNhs14184	Smooth muscle cells - airway, asthmatic, donor2
UBERON:0002204	Musculoskeletal System	UBERON:0001135	smooth muscle tissue	CNhs14186	Smooth muscle cells - airway, asthmatic, donor3
UBERON:0002204	Musculoskeletal System	UBERON:0001135	smooth muscle tissue	CNhs14187	Smooth muscle cells - airway, asthmatic, donor4
UBERON:0002204	Musculoskeletal System	UBERON:0001135	smooth muscle tissue	CNhs14188	Smooth muscle cells - airway, asthmatic, donor5
UBERON:0002204	Musculoskeletal System	UBERON:0001135	smooth muscle tissue	CNhs14189	Smooth muscle cells - airway, asthmatic, donor6
UBERON:0002204	Musculoskeletal System	UBERON:0001135	smooth muscle tissue	CNhs14190	Smooth muscle cells - airway, control, donor1

UBERON:0002204	Musculoskeletal System	UBERON:0001135	smooth muscle tissue	CNhs14191	Smooth muscle cells - airway, control, donor2
UBERON:0002204	Musculoskeletal System	UBERON:0001135	smooth muscle tissue	CNhs14192	Smooth muscle cells - airway, control, donor3
UBERON:0002204	Musculoskeletal System	UBERON:0001135	smooth muscle tissue	CNhs14193	Smooth muscle cells - airway, control, donor4
UBERON:0002204	Musculoskeletal System	UBERON:0001383	muscle of leg	CNhs13454	skeletal muscle - soleus muscle, donor1
UBERON:0002204	Musculoskeletal System	UBERON:0001383	muscle of leg	CNhs13435	achilles tendon, donor2
UBERON:0002204	Musculoskeletal System	UBERON:0001389	soleus	CNhs13454	skeletal muscle - soleus muscle, donor1
UBERON:0002204	Musculoskeletal System	UBERON:0001434	skeletal system	CNhs12845	bone marrow, adult
UBERON:0002204	Musculoskeletal System	UBERON:0001434	skeletal system	CNhs12611	olfactory region, adult
UBERON:0002204	Musculoskeletal System	UBERON:0001434	skeletal system	CNhs13435	achilles tendon, donor2
UBERON:0002204	Musculoskeletal System	UBERON:0001434	skeletal system	CNhs13439	cruciate ligament, donor2
UBERON:0002204	Musculoskeletal System	UBERON:0001434	skeletal system	CNhs10846	Mesenchymal Stem Cells - Vertebral, donor1
UBERON:0002204	Musculoskeletal System	UBERON:0001434	skeletal system	CNhs12493	Fibroblast - Periodontal Ligament, donor4 (PL29)
UBERON:0002204	Musculoskeletal System	UBERON:0001434	skeletal system	CNhs10867	Fibroblast - Periodontal Ligament, donor1
UBERON:0002204	Musculoskeletal System	UBERON:0001434	skeletal system	CNhs10876	Anulus Pulposus Cell, donor1
UBERON:0002204	Musculoskeletal System	UBERON:0001434	skeletal system	CNhs10881	Nucleus Pulposus Cell, donor1
UBERON:0002204	Musculoskeletal System	UBERON:0001434	skeletal system	CNhs11068	Synoviocyte, donor1
UBERON:0002204	Musculoskeletal System	UBERON:0001434	skeletal system	CNhs11953	Fibroblast - Periodontal Ligament, donor5 (PL30)
UBERON:0002204	Musculoskeletal System	UBERON:0001434	skeletal system	CNhs11962	Fibroblast - Periodontal Ligament, donor2
UBERON:0002204	Musculoskeletal System	UBERON:0001434	skeletal system	CNhs11992	Synoviocyte, donor2
UBERON:0002204	Musculoskeletal System	UBERON:0001434	skeletal system	CNhs11996	Fibroblast - Periodontal Ligament, donor6 (PLH3)
UBERON:0002204	Musculoskeletal System	UBERON:0001434	skeletal system	CNhs11907	Fibroblast - Periodontal Ligament, donor3
UBERON:0002204	Musculoskeletal System	UBERON:0001434	skeletal system	CNhs12019	Nucleus Pulposus Cell, donor2
UBERON:0002204	Musculoskeletal System	UBERON:0001434	skeletal system	CNhs12050	Synoviocyte, donor3
UBERON:0002204	Musculoskeletal System	UBERON:0001434	skeletal system	CNhs12063	Nucleus Pulposus Cell, donor3
UBERON:0002204	Musculoskeletal System	UBERON:0001434	skeletal system	CNhs12064	Anulus Pulposus Cell, donor2
UBERON:0002204	Musculoskeletal System	UBERON:0001434	skeletal system	CNhs11316	Mesenchymal Stem Cells - bone marrow, donor4
UBERON:0002204	Musculoskeletal System	UBERON:0001434	skeletal system	CNhs11344	Mesenchymal Stem Cells - bone marrow, donor1
UBERON:0002204	Musculoskeletal System	UBERON:0001434	skeletal system	CNhs12100	Mesenchymal Stem Cells - bone marrow, donor2
UBERON:0002204	Musculoskeletal System	UBERON:0001434	skeletal system	CNhs12126	Mesenchymal Stem Cells - bone marrow, donor3
UBERON:0002204	Musculoskeletal System	UBERON:0001434	skeletal system	CNhs12366	mesenchymal precursor cell - bone marrow, donor1
UBERON:0002204	Musculoskeletal System	UBERON:0001434	skeletal system	CNhs12367	mesenchymal precursor cell - bone marrow, donor2
UBERON:0002204	Musculoskeletal System	UBERON:0001434	skeletal system	CNhs13098	mesenchymal precursor cell - bone marrow, donor3
UBERON:0002204	Musculoskeletal System	UBERON:0001434	skeletal system	CNhs13550	Mallassez-derived cells, donor2
UBERON:0002204	Musculoskeletal System	UBERON:0001434	skeletal system	CNhs13551	Mallassez-derived cells, donor3
UBERON:0002204	Musculoskeletal System	UBERON:0001468	intervertebral joint	CNhs10876	Anulus Pulposus Cell, donor1
UBERON:0002204	Musculoskeletal System	UBERON:0001468	intervertebral joint	CNhs10881	Nucleus Pulposus Cell, donor1
UBERON:0002204	Musculoskeletal System	UBERON:0001468	intervertebral joint	CNhs12019	Nucleus Pulposus Cell, donor2
UBERON:0002204	Musculoskeletal System	UBERON:0001468	intervertebral joint	CNhs12063	Nucleus Pulposus Cell, donor3
UBERON:0002204	Musculoskeletal System	UBERON:0001468	intervertebral joint	CNhs12064	Anulus Pulposus Cell, donor2
UBERON:0002204	Musculoskeletal System	UBERON:0001474	bone	CNhs12845	bone marrow, adult
UBERON:0002204	Musculoskeletal System	UBERON:0001474	bone	CNhs10846	Mesenchymal Stem Cells - Vertebral, donor1
UBERON:0002204	Musculoskeletal System	UBERON:0001474	bone	CNhs11316	Mesenchymal Stem Cells - bone marrow, donor4
UBERON:0002204	Musculoskeletal System	UBERON:0001474	bone	CNhs11344	Mesenchymal Stem Cells - bone marrow, donor1
UBERON:0002204	Musculoskeletal System	UBERON:0001474	bone	CNhs12100	Mesenchymal Stem Cells - bone marrow, donor2
UBERON:0002204	Musculoskeletal System	UBERON:0001474	bone	CNhs12126	Mesenchymal Stem Cells - bone marrow, donor3
UBERON:0002204	Musculoskeletal System	UBERON:0001474	bone	CNhs12366	mesenchymal precursor cell - bone marrow, donor1
UBERON:0002204	Musculoskeletal System	UBERON:0001474	bone	CNhs12367	mesenchymal precursor cell - bone marrow, donor2
UBERON:0002204	Musculoskeletal System	UBERON:0001474	bone	CNhs13098	mesenchymal precursor cell - bone marrow, donor3
UBERON:0002204	Musculoskeletal System	UBERON:0001485	knee joint	CNhs13439	cruciate ligament, donor2
UBERON:0002204	Musculoskeletal System	UBERON:0001577	muscle of face	CNhs13444	eye - muscle inferior rectus, donor1
UBERON:0002204	Musculoskeletal System	UBERON:0001577	muscle of face	CNhs13441	eye - muscle superior, donor2
UBERON:0002204	Musculoskeletal System	UBERON:0001577	muscle of face	CNhs13442	eye - muscle lateral, donor2
UBERON:0002204	Musculoskeletal System	UBERON:0001577	muscle of face	CNhs13443	eye - muscle medial, donor2
UBERON:0002204	Musculoskeletal System	UBERON:0001601	extra-ocular muscle	CNhs13444	eye - muscle inferior rectus, donor1
UBERON:0002204	Musculoskeletal System	UBERON:0001630	muscle organ	CNhs11779	diaphragm, fetal, donor1
UBERON:0002204	Musculoskeletal System	UBERON:0001630	muscle organ	CNhs13444	eye - muscle inferior rectus, donor1
UBERON:0002204	Musculoskeletal System	UBERON:0001630	muscle organ	CNhs13454	skeletal muscle - soleus muscle, donor1
UBERON:0002204	Musculoskeletal System	UBERON:0001630	muscle organ	CNhs13435	achilles tendon, donor2
UBERON:0002204	Musculoskeletal System	UBERON:0001630	muscle organ	CNhs13441	eye - muscle superior, donor2
UBERON:0002204	Musculoskeletal System	UBERON:0001630	muscle organ	CNhs13442	eye - muscle lateral, donor2
UBERON:0002204	Musculoskeletal System	UBERON:0001630	muscle organ	CNhs13443	eye - muscle medial, donor2
UBERON:0002204	Musculoskeletal System	UBERON:0001665	triceps surae	CNhs13454	skeletal muscle - soleus muscle, donor1
UBERON:0002204	Musculoskeletal System	UBERON:0001665	triceps surae	CNhs13435	achilles tendon, donor2
UBERON:0002204	Musculoskeletal System	UBERON:0001703	neurocranium	CNhs12611	olfactory region, adult
UBERON:0002204	Musculoskeletal System	UBERON:0001708	jaw	CNhs13550	Mallassez-derived cells, donor2
UBERON:0002204	Musculoskeletal System	UBERON:0001708	jaw	CNhs13551	Mallassez-derived cells, donor3
UBERON:0002204	Musculoskeletal System	UBERON:0001758	periodontium	CNhs12493	Fibroblast - Periodontal Ligament, donor4 (PL29)
UBERON:0002204	Musculoskeletal System	UBERON:0001758	periodontium	CNhs10867	Fibroblast - Periodontal Ligament, donor1
UBERON:0002204	Musculoskeletal System	UBERON:0001758	periodontium	CNhs11953	Fibroblast - Periodontal Ligament, donor5 (PL30)
UBERON:0002204	Musculoskeletal System	UBERON:0001758	periodontium	CNhs11962	Fibroblast - Periodontal Ligament, donor2
UBERON:0002204	Musculoskeletal System	UBERON:0001758	periodontium	CNhs11996	Fibroblast - Periodontal Ligament, donor6 (PLH3)
UBERON:0002204	Musculoskeletal System	UBERON:0001758	periodontium	CNhs11907	Fibroblast - Periodontal Ligament, donor3
UBERON:0002204	Musculoskeletal System	UBERON:0001774	muscle of trunk	CNhs11779	diaphragm, fetal, donor1
UBERON:0002204	Musculoskeletal System	UBERON:0001995	fibrocartilage	CNhs10876	Anulus Pulposus Cell, donor1
UBERON:0002204	Musculoskeletal System	UBERON:0001995	fibrocartilage	CNhs12064	Anulus Pulposus Cell, donor2
UBERON:0002204	Musculoskeletal System	UBERON:0002018	synovial membrane of synovial joint	CNhs11068	Synoviocyte, donor1
UBERON:0002204	Musculoskeletal System	UBERON:0002018	synovial membrane of synovial joint	CNhs11992	Synoviocyte, donor2
UBERON:0002204	Musculoskeletal System	UBERON:0002018	synovial membrane of synovial joint	CNhs12050	Synoviocyte, donor3
UBERON:0002204	Musculoskeletal System	UBERON:0002036	striated muscle tissue	CNhs10629	skeletal muscle, adult, pool1
UBERON:0002204	Musculoskeletal System	UBERON:0002036	striated muscle tissue	CNhs11776	skeletal muscle, fetal, donor1
UBERON:0002204	Musculoskeletal System	UBERON:0002036	striated muscle tissue	CNhs11779	diaphragm, fetal, donor1
UBERON:0002204	Musculoskeletal System	UBERON:0002036	striated muscle tissue	CNhs13444	eye - muscle inferior rectus, donor1
UBERON:0002204	Musculoskeletal System	UBERON:0002036	striated muscle tissue	CNhs13454	skeletal muscle - soleus muscle, donor1
UBERON:0002204	Musculoskeletal System	UBERON:0002036	striated muscle tissue	CNhs10869	Skeletal Muscle Satellite Cells, donor1
UBERON:0002204	Musculoskeletal System	UBERON:0002036	striated muscle tissue	CNhs10870	Myoblast, donor1
UBERON:0002204	Musculoskeletal System	UBERON:0002036	striated muscle tissue	CNhs11083	Skeletal Muscle Cells, donor1
UBERON:0002204	Musculoskeletal System	UBERON:0002036	striated muscle tissue	CNhs11084	Skeletal muscle cells differentiated into Myotubes - multinucleated, donor1
UBERON:0002204	Musculoskeletal System	UBERON:0002036	striated muscle tissue	CNhs11964	Skeletal Muscle Satellite Cells, donor2
UBERON:0002204	Musculoskeletal System	UBERON:0002036	striated muscle tissue	CNhs11965	Myoblast, donor2
UBERON:0002204	Musculoskeletal System	UBERON:0002036	striated muscle tissue	CNhs11983	Skeletal Muscle Cells, donor2
UBERON:0002204	Musculoskeletal System	UBERON:0002036	striated muscle tissue	CNhs11984	Skeletal muscle cells differentiated into Myotubes - multinucleated, donor2
UBERON:0002204	Musculoskeletal System	UBERON:0002036	striated muscle tissue	CNhs12008	Skeletal Muscle Satellite Cells, donor3
UBERON:0002204	Musculoskeletal System	UBERON:0002036	striated muscle tissue	CNhs11908	Myoblast, donor3
UBERON:0002204	Musculoskeletal System	UBERON:0002036	striated muscle tissue	CNhs12040	Skeletal Muscle Cells, donor3
UBERON:0002204	Musculoskeletal System	UBERON:0002036	striated muscle tissue	CNhs12041	Skeletal muscle cells differentiated into Myotubes - multinucleated, donor3
UBERON:0002204	Musculoskeletal System	UBERON:0002036	striated muscle tissue	CNhs12053	Skeletal Muscle Cells, donor4
UBERON:0002204	Musculoskeletal System	UBERON:0002036	striated muscle tissue	CNhs12056	Skeletal Muscle Cells, donor5
UBERON:0002204	Musculoskeletal System	UBERON:0002036	striated muscle tissue	CNhs12060	Skeletal Muscle Cells, donor6

UBERON:0002204	Musculoskeletal System	UBERON:0002111	artery smooth muscle tissue	CNhs10838	Smooth Muscle Cells - Aortic, donor0
UBERON:0002204	Musculoskeletal System	UBERON:0002111	artery smooth muscle tissue	CNhs11085	Smooth Muscle Cells - Aortic, donor1
UBERON:0002204	Musculoskeletal System	UBERON:0002111	artery smooth muscle tissue	CNhs11305	Smooth Muscle Cells - Aortic, donor2
UBERON:0002204	Musculoskeletal System	UBERON:0002111	artery smooth muscle tissue	CNhs11309	Smooth Muscle Cells - Aortic, donor3
UBERON:0002204	Musculoskeletal System	UBERON:0002204	musculoskeletal system	CNhs10629	skeletal muscle, adult, pool1
UBERON:0002204	Musculoskeletal System	UBERON:0002204	musculoskeletal system	CNhs10630	small intestine, adult, pool1
UBERON:0002204	Musculoskeletal System	UBERON:0002204	musculoskeletal system	CNhs11755	smooth muscle, adult, pool1
UBERON:0002204	Musculoskeletal System	UBERON:0002204	musculoskeletal system	CNhs11776	skeletal muscle, fetal, donor1
UBERON:0002204	Musculoskeletal System	UBERON:0002204	musculoskeletal system	CNhs11779	diaphragm, fetal, donor1
UBERON:0002204	Musculoskeletal System	UBERON:0002204	musculoskeletal system	CNhs12845	bone marrow, adult
UBERON:0002204	Musculoskeletal System	UBERON:0002204	musculoskeletal system	CNhs12611	olfactory region, adult
UBERON:0002204	Musculoskeletal System	UBERON:0002204	musculoskeletal system	CNhs13444	eye - muscle inferior rectus, donor1
UBERON:0002204	Musculoskeletal System	UBERON:0002204	musculoskeletal system	CNhs13454	skeletal muscle - soleus muscle, donor1
UBERON:0002204	Musculoskeletal System	UBERON:0002204	musculoskeletal system	CNhs13435	achilles tendon, donor2
UBERON:0002204	Musculoskeletal System	UBERON:0002204	musculoskeletal system	CNhs13439	cruciate ligament, donor2
UBERON:0002204	Musculoskeletal System	UBERON:0002204	musculoskeletal system	CNhs13441	eye - muscle superior, donor2
UBERON:0002204	Musculoskeletal System	UBERON:0002204	musculoskeletal system	CNhs13442	eye - muscle lateral, donor2
UBERON:0002204	Musculoskeletal System	UBERON:0002204	musculoskeletal system	CNhs13443	eye - muscle medial, donor2
UBERON:0002204	Musculoskeletal System	UBERON:0002204	musculoskeletal system	CNhs10838	Smooth Muscle Cells - Aortic, donor0
UBERON:0002204	Musculoskeletal System	UBERON:0002204	musculoskeletal system	CNhs10846	Mesenchymal Stem Cells - Vertebral, donor1
UBERON:0002204	Musculoskeletal System	UBERON:0002204	musculoskeletal system	CNhs12493	Fibroblast - Periodontal Ligament, donor4 (PL29)
UBERON:0002204	Musculoskeletal System	UBERON:0002204	musculoskeletal system	CNhs10867	Fibroblast - Periodontal Ligament, donor1
UBERON:0002204	Musculoskeletal System	UBERON:0002204	musculoskeletal system	CNhs10868	Smooth Muscle Cells - Colonic, donor1
UBERON:0002204	Musculoskeletal System	UBERON:0002204	musculoskeletal system	CNhs10869	Skeletal Muscle Satellite Cells, donor1
UBERON:0002204	Musculoskeletal System	UBERON:0002204	musculoskeletal system	CNhs10870	Myoblast, donor1
UBERON:0002204	Musculoskeletal System	UBERON:0002204	musculoskeletal system	CNhs10876	Anulus Pulposus Cell, donor1
UBERON:0002204	Musculoskeletal System	UBERON:0002204	musculoskeletal system	CNhs10881	Nucleus Pulposus Cell, donor1
UBERON:0002204	Musculoskeletal System	UBERON:0002204	musculoskeletal system	CNhs11083	Skeletal Muscle Cells, donor1
UBERON:0002204	Musculoskeletal System	UBERON:0002204	musculoskeletal system	CNhs11084	Skeletal muscle cells differentiated into Myotubes - multinucleated, donor1
UBERON:0002204	Musculoskeletal System	UBERON:0002204	musculoskeletal system	CNhs11085	Smooth Muscle Cells - Aortic, donor1
UBERON:0002204	Musculoskeletal System	UBERON:0002204	musculoskeletal system	CNhs11068	Synoviocyte, donor1
UBERON:0002204	Musculoskeletal System	UBERON:0002204	musculoskeletal system	CNhs11953	Fibroblast - Periodontal Ligament, donor5 (PL30)
UBERON:0002204	Musculoskeletal System	UBERON:0002204	musculoskeletal system	CNhs11962	Fibroblast - Periodontal Ligament, donor2
UBERON:0002204	Musculoskeletal System	UBERON:0002204	musculoskeletal system	CNhs11963	Smooth Muscle Cells - Colonic, donor2
UBERON:0002204	Musculoskeletal System	UBERON:0002204	musculoskeletal system	CNhs11964	Skeletal Muscle Satellite Cells, donor2
UBERON:0002204	Musculoskeletal System	UBERON:0002204	musculoskeletal system	CNhs11965	Myoblast, donor2
UBERON:0002204	Musculoskeletal System	UBERON:0002204	musculoskeletal system	CNhs11983	Skeletal Muscle Cells, donor2
UBERON:0002204	Musculoskeletal System	UBERON:0002204	musculoskeletal system	CNhs11984	Skeletal muscle cells differentiated into Myotubes - multinucleated, donor2
UBERON:0002204	Musculoskeletal System	UBERON:0002204	musculoskeletal system	CNhs11305	Smooth Muscle Cells - Aortic, donor2
UBERON:0002204	Musculoskeletal System	UBERON:0002204	musculoskeletal system	CNhs11992	Synoviocyte, donor2
UBERON:0002204	Musculoskeletal System	UBERON:0002204	musculoskeletal system	CNhs11996	Fibroblast - Periodontal Ligament, donor6 (PLH3)
UBERON:0002204	Musculoskeletal System	UBERON:0002204	musculoskeletal system	CNhs11907	Fibroblast - Periodontal Ligament, donor3
UBERON:0002204	Musculoskeletal System	UBERON:0002204	musculoskeletal system	CNhs12007	Smooth Muscle Cells - Colonic, donor3
UBERON:0002204	Musculoskeletal System	UBERON:0002204	musculoskeletal system	CNhs12008	Skeletal Muscle Satellite Cells, donor3
UBERON:0002204	Musculoskeletal System	UBERON:0002204	musculoskeletal system	CNhs11908	Myoblast, donor3
UBERON:0002204	Musculoskeletal System	UBERON:0002204	musculoskeletal system	CNhs12019	Nucleus Pulposus Cell, donor2
UBERON:0002204	Musculoskeletal System	UBERON:0002204	musculoskeletal system	CNhs12040	Skeletal Muscle Cells, donor3
UBERON:0002204	Musculoskeletal System	UBERON:0002204	musculoskeletal system	CNhs12041	Skeletal muscle cells differentiated into Myotubes - multinucleated, donor3
UBERON:0002204	Musculoskeletal System	UBERON:0002204	musculoskeletal system	CNhs11309	Smooth Muscle Cells - Aortic, donor3
UBERON:0002204	Musculoskeletal System	UBERON:0002204	musculoskeletal system	CNhs12050	Synoviocyte, donor3
UBERON:0002204	Musculoskeletal System	UBERON:0002204	musculoskeletal system	CNhs12053	Skeletal Muscle Cells, donor4
UBERON:0002204	Musculoskeletal System	UBERON:0002204	musculoskeletal system	CNhs12056	Skeletal Muscle Cells, donor5
UBERON:0002204	Musculoskeletal System	UBERON:0002204	musculoskeletal system	CNhs12060	Skeletal Muscle Cells, donor6
UBERON:0002204	Musculoskeletal System	UBERON:0002204	musculoskeletal system	CNhs12063	Nucleus Pulposus Cell, donor3
UBERON:0002204	Musculoskeletal System	UBERON:0002204	musculoskeletal system	CNhs12064	Anulus Pulposus Cell, donor2
UBERON:0002204	Musculoskeletal System	UBERON:0002204	musculoskeletal system	CNhs11316	Mesenchymal Stem Cells - bone marrow, donor4
UBERON:0002204	Musculoskeletal System	UBERON:0002204	musculoskeletal system	CNhs11344	Mesenchymal Stem Cells - bone marrow, donor1
UBERON:0002204	Musculoskeletal System	UBERON:0002204	musculoskeletal system	CNhs12100	Mesenchymal Stem Cells - bone marrow, donor2
UBERON:0002204	Musculoskeletal System	UBERON:0002204	musculoskeletal system	CNhs12126	Mesenchymal Stem Cells - bone marrow, donor3
UBERON:0002204	Musculoskeletal System	UBERON:0002204	musculoskeletal system	CNhs12366	mesenchymal precursor cell - bone marrow, donor1
UBERON:0002204	Musculoskeletal System	UBERON:0002204	musculoskeletal system	CNhs12367	mesenchymal precursor cell - bone marrow, donor2
UBERON:0002204	Musculoskeletal System	UBERON:0002204	musculoskeletal system	CNhs12639	tenocyte, donor1
UBERON:0002204	Musculoskeletal System	UBERON:0002204	musculoskeletal system	CNhs12640	tenocyte, donor2
UBERON:0002204	Musculoskeletal System	UBERON:0002204	musculoskeletal system	CNhs13098	mesenchymal precursor cell - bone marrow, donor3
UBERON:0002204	Musculoskeletal System	UBERON:0002204	musculoskeletal system	CNhs13550	Mallassez-derived cells, donor2
UBERON:0002204	Musculoskeletal System	UBERON:0002204	musculoskeletal system	CNhs13551	Mallassez-derived cells, donor3
UBERON:0002204	Musculoskeletal System	UBERON:0002204	musculoskeletal system	CNhs14183	Smooth muscle cells - airway, asthmatic, donor1
UBERON:0002204	Musculoskeletal System	UBERON:0002204	musculoskeletal system	CNhs14184	Smooth muscle cells - airway, asthmatic, donor2
UBERON:0002204	Musculoskeletal System	UBERON:0002204	musculoskeletal system	CNhs14186	Smooth muscle cells - airway, asthmatic, donor3
UBERON:0002204	Musculoskeletal System	UBERON:0002204	musculoskeletal system	CNhs14187	Smooth muscle cells - airway, asthmatic, donor4
UBERON:0002204	Musculoskeletal System	UBERON:0002204	musculoskeletal system	CNhs14188	Smooth muscle cells - airway, asthmatic, donor5
UBERON:0002204	Musculoskeletal System	UBERON:0002204	musculoskeletal system	CNhs14189	Smooth muscle cells - airway, asthmatic, donor6
UBERON:0002204	Musculoskeletal System	UBERON:0002204	musculoskeletal system	CNhs14190	Smooth muscle cells - airway, control, donor1
UBERON:0002204	Musculoskeletal System	UBERON:0002204	musculoskeletal system	CNhs14191	Smooth muscle cells - airway, control, donor2
UBERON:0002204	Musculoskeletal System	UBERON:0002204	musculoskeletal system	CNhs14192	Smooth muscle cells - airway, control, donor3
UBERON:0002204	Musculoskeletal System	UBERON:0002204	musculoskeletal system	CNhs14193	Smooth muscle cells - airway, control, donor4
UBERON:0002204	Musculoskeletal System	UBERON:0002209	fibrous joint	CNhs10876	Anulus Pulposus Cell, donor1
UBERON:0002204	Musculoskeletal System	UBERON:0002209	fibrous joint	CNhs10881	Nucleus Pulposus Cell, donor1
UBERON:0002204	Musculoskeletal System	UBERON:0002209	fibrous joint	CNhs12019	Nucleus Pulposus Cell, donor2
UBERON:0002204	Musculoskeletal System	UBERON:0002209	fibrous joint	CNhs12063	Nucleus Pulposus Cell, donor3
UBERON:0002204	Musculoskeletal System	UBERON:0002209	fibrous joint	CNhs12064	Anulus Pulposus Cell, donor2
UBERON:0002204	Musculoskeletal System	UBERON:0002213	cartilaginous joint	CNhs10876	Anulus Pulposus Cell, donor1
UBERON:0002204	Musculoskeletal System	UBERON:0002213	cartilaginous joint	CNhs10881	Nucleus Pulposus Cell, donor1
UBERON:0002204	Musculoskeletal System	UBERON:0002213	cartilaginous joint	CNhs12019	Nucleus Pulposus Cell, donor2
UBERON:0002204	Musculoskeletal System	UBERON:0002213	cartilaginous joint	CNhs12063	Nucleus Pulposus Cell, donor3
UBERON:0002204	Musculoskeletal System	UBERON:0002213	cartilaginous joint	CNhs12064	Anulus Pulposus Cell, donor2
UBERON:0002204	Musculoskeletal System	UBERON:0002216	symphysis	CNhs10876	Anulus Pulposus Cell, donor1
UBERON:0002204	Musculoskeletal System	UBERON:0002216	symphysis	CNhs10881	Nucleus Pulposus Cell, donor1
UBERON:0002204	Musculoskeletal System	UBERON:0002216	symphysis	CNhs12019	Nucleus Pulposus Cell, donor2
UBERON:0002204	Musculoskeletal System	UBERON:0002216	symphysis	CNhs12063	Nucleus Pulposus Cell, donor3
UBERON:0002204	Musculoskeletal System	UBERON:0002216	symphysis	CNhs12064	Anulus Pulposus Cell, donor2
UBERON:0002204	Musculoskeletal System	UBERON:0002217	synovial joint	CNhs13439	cruciate ligament, donor2
UBERON:0002204	Musculoskeletal System	UBERON:0002217	synovial joint	CNhs11068	Synoviocyte, donor1
UBERON:0002204	Musculoskeletal System	UBERON:0002217	synovial joint	CNhs11992	Synoviocyte, donor2
UBERON:0002204	Musculoskeletal System	UBERON:0002217	synovial joint	CNhs12050	Synoviocyte, donor3
UBERON:0002204	Musculoskeletal System	UBERON:0002241	chondrocranium	CNhs12611	olfactory region, adult
UBERON:0002204	Musculoskeletal System	UBERON:0002242	nucleus pulposus	CNhs10881	Nucleus Pulposus Cell, donor1
UBERON:0002204	Musculoskeletal System	UBERON:0002242	nucleus pulposus	CNhs12019	Nucleus Pulposus Cell, donor2

UBERON:0002204	Musculoskeletal System	UBERON:0002242	nucleus pulposus	CNhs12063	Nucleus Pulposus Cell, donor3
UBERON:0002204	Musculoskeletal System	UBERON:0002371	bone marrow	CNhs12845	bone marrow, adult
UBERON:0002204	Musculoskeletal System	UBERON:0002371	bone marrow	CNhs11316	Mesenchymal Stem Cells - bone marrow, donor4
UBERON:0002204	Musculoskeletal System	UBERON:0002371	bone marrow	CNhs11344	Mesenchymal Stem Cells - bone marrow, donor1
UBERON:0002204	Musculoskeletal System	UBERON:0002371	bone marrow	CNhs12100	Mesenchymal Stem Cells - bone marrow, donor2
UBERON:0002204	Musculoskeletal System	UBERON:0002371	bone marrow	CNhs12126	Mesenchymal Stem Cells - bone marrow, donor3
UBERON:0002204	Musculoskeletal System	UBERON:0002371	bone marrow	CNhs12366	mesenchymal precursor cell - bone marrow, donor1
UBERON:0002204	Musculoskeletal System	UBERON:0002371	bone marrow	CNhs12367	mesenchymal precursor cell - bone marrow, donor2
UBERON:0002204	Musculoskeletal System	UBERON:0002371	bone marrow	CNhs13098	mesenchymal precursor cell - bone marrow, donor3
UBERON:0002204	Musculoskeletal System	UBERON:0002376	muscle of head	CNhs13444	eye - muscle inferior rectus, donor1
UBERON:0002204	Musculoskeletal System	UBERON:0002376	muscle of head	CNhs13441	eye - muscle superior, donor2
UBERON:0002204	Musculoskeletal System	UBERON:0002376	muscle of head	CNhs13442	eye - muscle lateral, donor2
UBERON:0002204	Musculoskeletal System	UBERON:0002376	muscle of head	CNhs13443	eye - muscle medial, donor2
UBERON:0002204	Musculoskeletal System	UBERON:0002385	muscle tissue	CNhs10629	skeletal muscle, adult, pool1
UBERON:0002204	Musculoskeletal System	UBERON:0002385	muscle tissue	CNhs10630	small intestine, adult, pool1
UBERON:0002204	Musculoskeletal System	UBERON:0002385	muscle tissue	CNhs11755	smooth muscle, adult, pool1
UBERON:0002204	Musculoskeletal System	UBERON:0002385	muscle tissue	CNhs11776	skeletal muscle, fetal, donor1
UBERON:0002204	Musculoskeletal System	UBERON:0002385	muscle tissue	CNhs11779	diaphragm, fetal, donor1
UBERON:0002204	Musculoskeletal System	UBERON:0002385	muscle tissue	CNhs13444	eye - muscle inferior rectus, donor1
UBERON:0002204	Musculoskeletal System	UBERON:0002385	muscle tissue	CNhs13454	skeletal muscle - soleus muscle, donor1
UBERON:0002204	Musculoskeletal System	UBERON:0002385	muscle tissue	CNhs10838	Smooth Muscle Cells - Aortic, donor0
UBERON:0002204	Musculoskeletal System	UBERON:0002385	muscle tissue	CNhs10868	Smooth Muscle Cells - Colonic, donor1
UBERON:0002204	Musculoskeletal System	UBERON:0002385	muscle tissue	CNhs10869	Skeletal Muscle Satellite Cells, donor1
UBERON:0002204	Musculoskeletal System	UBERON:0002385	muscle tissue	CNhs10870	Myoblast, donor1
UBERON:0002204	Musculoskeletal System	UBERON:0002385	muscle tissue	CNhs11083	Skeletal Muscle Cells, donor1
UBERON:0002204	Musculoskeletal System	UBERON:0002385	muscle tissue	CNhs11084	Skeletal muscle cells differentiated into Myotubes - multinucleated, donor1
UBERON:0002204	Musculoskeletal System	UBERON:0002385	muscle tissue	CNhs11085	Smooth Muscle Cells - Aortic, donor1
UBERON:0002204	Musculoskeletal System	UBERON:0002385	muscle tissue	CNhs11963	Smooth Muscle Cells - Colonic, donor2
UBERON:0002204	Musculoskeletal System	UBERON:0002385	muscle tissue	CNhs11964	Skeletal Muscle Satellite Cells, donor2
UBERON:0002204	Musculoskeletal System	UBERON:0002385	muscle tissue	CNhs11965	Myoblast, donor2
UBERON:0002204	Musculoskeletal System	UBERON:0002385	muscle tissue	CNhs11983	Skeletal Muscle Cells, donor2
UBERON:0002204	Musculoskeletal System	UBERON:0002385	muscle tissue	CNhs11984	Skeletal muscle cells differentiated into Myotubes - multinucleated, donor2
UBERON:0002204	Musculoskeletal System	UBERON:0002385	muscle tissue	CNhs11305	Smooth Muscle Cells - Aortic, donor2
UBERON:0002204	Musculoskeletal System	UBERON:0002385	muscle tissue	CNhs12007	Smooth Muscle Cells - Colonic, donor3
UBERON:0002204	Musculoskeletal System	UBERON:0002385	muscle tissue	CNhs12008	Skeletal Muscle Satellite Cells, donor3
UBERON:0002204	Musculoskeletal System	UBERON:0002385	muscle tissue	CNhs11908	Myoblast, donor3
UBERON:0002204	Musculoskeletal System	UBERON:0002385	muscle tissue	CNhs12040	Skeletal Muscle Cells, donor3
UBERON:0002204	Musculoskeletal System	UBERON:0002385	muscle tissue	CNhs12041	Skeletal muscle cells differentiated into Myotubes - multinucleated, donor3
UBERON:0002204	Musculoskeletal System	UBERON:0002385	muscle tissue	CNhs11309	Smooth Muscle Cells - Aortic, donor3
UBERON:0002204	Musculoskeletal System	UBERON:0002385	muscle tissue	CNhs12053	Skeletal Muscle Cells, donor4
UBERON:0002204	Musculoskeletal System	UBERON:0002385	muscle tissue	CNhs12056	Skeletal Muscle Cells, donor5
UBERON:0002204	Musculoskeletal System	UBERON:0002385	muscle tissue	CNhs12060	Skeletal Muscle Cells, donor6
UBERON:0002204	Musculoskeletal System	UBERON:0002385	muscle tissue	CNhs14183	Smooth muscle cells - airway, asthmatic, donor1
UBERON:0002204	Musculoskeletal System	UBERON:0002385	muscle tissue	CNhs14184	Smooth muscle cells - airway, asthmatic, donor2
UBERON:0002204	Musculoskeletal System	UBERON:0002385	muscle tissue	CNhs14186	Smooth muscle cells - airway, asthmatic, donor3
UBERON:0002204	Musculoskeletal System	UBERON:0002385	muscle tissue	CNhs14187	Smooth muscle cells - airway, asthmatic, donor4
UBERON:0002204	Musculoskeletal System	UBERON:0002385	muscle tissue	CNhs14188	Smooth muscle cells - airway, asthmatic, donor5
UBERON:0002204	Musculoskeletal System	UBERON:0002385	muscle tissue	CNhs14189	Smooth muscle cells - airway, asthmatic, donor6
UBERON:0002204	Musculoskeletal System	UBERON:0002385	muscle tissue	CNhs14190	Smooth muscle cells - airway, control, donor1
UBERON:0002204	Musculoskeletal System	UBERON:0002385	muscle tissue	CNhs14191	Smooth muscle cells - airway, control, donor2
UBERON:0002204	Musculoskeletal System	UBERON:0002385	muscle tissue	CNhs14192	Smooth muscle cells - airway, control, donor3
UBERON:0002204	Musculoskeletal System	UBERON:0002385	muscle tissue	CNhs14193	Smooth muscle cells - airway, control, donor4
UBERON:0002204	Musculoskeletal System	UBERON:0002412	vertebra	CNhs10846	Mesenchymal Stem Cells - Vertebral, donor1
UBERON:0002204	Musculoskeletal System	UBERON:0002418	cartilage tissue	CNhs10876	Anulus Pulposus Cell, donor1
UBERON:0002204	Musculoskeletal System	UBERON:0002418	cartilage tissue	CNhs12064	Anulus Pulposus Cell, donor2
UBERON:0002204	Musculoskeletal System	UBERON:0002426	chest muscle	CNhs11779	diaphragm, fetal, donor1
UBERON:0002204	Musculoskeletal System	UBERON:0002513	endochondral bone	CNhs10846	Mesenchymal Stem Cells - Vertebral, donor1
UBERON:0002204	Musculoskeletal System	UBERON:0003112	olfactory region	CNhs12611	olfactory region, adult
UBERON:0002204	Musculoskeletal System	UBERON:0003128	cranium	CNhs12611	olfactory region, adult
UBERON:0002204	Musculoskeletal System	UBERON:0003129	skull	CNhs12611	olfactory region, adult
UBERON:0002204	Musculoskeletal System	UBERON:0003129	skull	CNhs12493	Fibroblast - Periodontal Ligament, donor4 (PL29)
UBERON:0002204	Musculoskeletal System	UBERON:0003129	skull	CNhs10867	Fibroblast - Periodontal Ligament, donor1
UBERON:0002204	Musculoskeletal System	UBERON:0003129	skull	CNhs11953	Fibroblast - Periodontal Ligament, donor5 (PL30)
UBERON:0002204	Musculoskeletal System	UBERON:0003129	skull	CNhs11962	Fibroblast - Periodontal Ligament, donor2
UBERON:0002204	Musculoskeletal System	UBERON:0003129	skull	CNhs11996	Fibroblast - Periodontal Ligament, donor6 (PLH3)
UBERON:0002204	Musculoskeletal System	UBERON:0003129	skull	CNhs11907	Fibroblast - Periodontal Ligament, donor3
UBERON:0002204	Musculoskeletal System	UBERON:0003269	skeletal muscle of eye	CNhs13444	eye - muscle inferior rectus, donor1
UBERON:0002204	Musculoskeletal System	UBERON:0003657	limb joint	CNhs13439	cruciate ligament, donor2
UBERON:0002204	Musculoskeletal System	UBERON:0003661	limb muscle	CNhs13454	skeletal muscle - soleus muscle, donor1
UBERON:0002204	Musculoskeletal System	UBERON:0003661	limb muscle	CNhs13435	achilles tendon, donor2
UBERON:0002204	Musculoskeletal System	UBERON:0003663	hindlimb muscle	CNhs13454	skeletal muscle - soleus muscle, donor1
UBERON:0002204	Musculoskeletal System	UBERON:0003663	hindlimb muscle	CNhs13435	achilles tendon, donor2
UBERON:0002204	Musculoskeletal System	UBERON:0003672	dentition	CNhs12493	Fibroblast - Periodontal Ligament, donor4 (PL29)
UBERON:0002204	Musculoskeletal System	UBERON:0003672	dentition	CNhs10867	Fibroblast - Periodontal Ligament, donor1
UBERON:0002204	Musculoskeletal System	UBERON:0003672	dentition	CNhs11953	Fibroblast - Periodontal Ligament, donor5 (PL30)
UBERON:0002204	Musculoskeletal System	UBERON:0003672	dentition	CNhs11962	Fibroblast - Periodontal Ligament, donor2
UBERON:0002204	Musculoskeletal System	UBERON:0003672	dentition	CNhs11996	Fibroblast - Periodontal Ligament, donor6 (PLH3)
UBERON:0002204	Musculoskeletal System	UBERON:0003672	dentition	CNhs11907	Fibroblast - Periodontal Ligament, donor3
UBERON:0002204	Musculoskeletal System	UBERON:0003701	calcaneal tendon	CNhs13435	achilles tendon, donor2
UBERON:0002204	Musculoskeletal System	UBERON:0003830	thoracic segment muscle	CNhs11779	diaphragm, fetal, donor1
UBERON:0002204	Musculoskeletal System	UBERON:0003831	respiratory system muscle	CNhs11779	diaphragm, fetal, donor1
UBERON:0002204	Musculoskeletal System	UBERON:0003840	hindlimb joint	CNhs13439	cruciate ligament, donor2
UBERON:0002204	Musculoskeletal System	UBERON:0003898	skeletal muscle of trunk	CNhs11779	diaphragm, fetal, donor1
UBERON:0002204	Musculoskeletal System	UBERON:0003899	skeletal muscle of head	CNhs13444	eye - muscle inferior rectus, donor1
UBERON:0002204	Musculoskeletal System	UBERON:0004174	leg joint	CNhs13439	cruciate ligament, donor2
UBERON:0002204	Musculoskeletal System	UBERON:0004178	aorta smooth muscle tissue	CNhs10838	Smooth Muscle Cells - Aortic, donor0
UBERON:0002204	Musculoskeletal System	UBERON:0004178	aorta smooth muscle tissue	CNhs11085	Smooth Muscle Cells - Aortic, donor1
UBERON:0002204	Musculoskeletal System	UBERON:0004178	aorta smooth muscle tissue	CNhs11305	Smooth Muscle Cells - Aortic, donor2
UBERON:0002204	Musculoskeletal System	UBERON:0004178	aorta smooth muscle tissue	CNhs11309	Smooth Muscle Cells - Aortic, donor3
UBERON:0002204	Musculoskeletal System	UBERON:0004225	respiratory system smooth muscle	CNhs14183	Smooth muscle cells - airway, asthmatic, donor1
UBERON:0002204	Musculoskeletal System	UBERON:0004225	respiratory system smooth muscle	CNhs14184	Smooth muscle cells - airway, asthmatic, donor2
UBERON:0002204	Musculoskeletal System	UBERON:0004225	respiratory system smooth muscle	CNhs14186	Smooth muscle cells - airway, asthmatic, donor3
UBERON:0002204	Musculoskeletal System	UBERON:0004225	respiratory system smooth muscle	CNhs14187	Smooth muscle cells - airway, asthmatic, donor4
UBERON:0002204	Musculoskeletal System	UBERON:0004225	respiratory system smooth muscle	CNhs14188	Smooth muscle cells - airway, asthmatic, donor5
UBERON:0002204	Musculoskeletal System	UBERON:0004225	respiratory system smooth muscle	CNhs14189	Smooth muscle cells - airway, asthmatic, donor6
UBERON:0002204	Musculoskeletal System	UBERON:0004225	respiratory system smooth muscle	CNhs14190	Smooth muscle cells - airway, control, donor1
UBERON:0002204	Musculoskeletal System	UBERON:0004225	respiratory system smooth muscle	CNhs14191	Smooth muscle cells - airway, control, donor2
UBERON:0002204	Musculoskeletal System	UBERON:0004225	respiratory system smooth muscle	CNhs14192	Smooth muscle cells - airway, control, donor3

UBERON:0002204	Musculoskeletal System	UBERON:0004225	respiratory system smooth muscle	CNhs14193	Smooth muscle cells - airway, control, donor4
UBERON:0002204	Musculoskeletal System	UBERON:0004237	blood vessel smooth muscle	CNhs10838	Smooth Muscle Cells - Aortic, donor0
UBERON:0002204	Musculoskeletal System	UBERON:0004237	blood vessel smooth muscle	CNhs11085	Smooth Muscle Cells - Aortic, donor1
UBERON:0002204	Musculoskeletal System	UBERON:0004237	blood vessel smooth muscle	CNhs11305	Smooth Muscle Cells - Aortic, donor2
UBERON:0002204	Musculoskeletal System	UBERON:0004237	blood vessel smooth muscle	CNhs11309	Smooth Muscle Cells - Aortic, donor3
UBERON:0002204	Musculoskeletal System	UBERON:0004256	hindlimb zeugopod muscle	CNhs13454	skeletal muscle - soleus muscle, donor1
UBERON:0002204	Musculoskeletal System	UBERON:0004256	hindlimb zeugopod muscle	CNhs13435	achilles tendon, donor2
UBERON:0002204	Musculoskeletal System	UBERON:0004277	eye muscle	CNhs13444	eye - muscle inferior rectus, donor1
UBERON:0002204	Musculoskeletal System	UBERON:0004277	eye muscle	CNhs13441	eye - muscle superior, donor2
UBERON:0002204	Musculoskeletal System	UBERON:0004277	eye muscle	CNhs13442	eye - muscle lateral, donor2
UBERON:0002204	Musculoskeletal System	UBERON:0004277	eye muscle	CNhs13443	eye - muscle medial, donor2
UBERON:0002204	Musculoskeletal System	UBERON:0004461	musculature of head	CNhs13444	eye - muscle inferior rectus, donor1
UBERON:0002204	Musculoskeletal System	UBERON:0004461	musculature of head	CNhs13441	eye - muscle superior, donor2
UBERON:0002204	Musculoskeletal System	UBERON:0004461	musculature of head	CNhs13442	eye - muscle lateral, donor2
UBERON:0002204	Musculoskeletal System	UBERON:0004461	musculature of head	CNhs13443	eye - muscle medial, donor2
UBERON:0002204	Musculoskeletal System	UBERON:0004473	musculature of face	CNhs13444	eye - muscle inferior rectus, donor1
UBERON:0002204	Musculoskeletal System	UBERON:0004473	musculature of face	CNhs13441	eye - muscle superior, donor2
UBERON:0002204	Musculoskeletal System	UBERON:0004473	musculature of face	CNhs13442	eye - muscle lateral, donor2
UBERON:0002204	Musculoskeletal System	UBERON:0004473	musculature of face	CNhs13443	eye - muscle medial, donor2
UBERON:0002204	Musculoskeletal System	UBERON:0004695	arterial system smooth muscle	CNhs10838	Smooth Muscle Cells - Aortic, donor0
UBERON:0002204	Musculoskeletal System	UBERON:0004695	arterial system smooth muscle	CNhs11085	Smooth Muscle Cells - Aortic, donor1
UBERON:0002204	Musculoskeletal System	UBERON:0004695	arterial system smooth muscle	CNhs11305	Smooth Muscle Cells - Aortic, donor2
UBERON:0002204	Musculoskeletal System	UBERON:0004695	arterial system smooth muscle	CNhs11309	Smooth Muscle Cells - Aortic, donor3
UBERON:0002204	Musculoskeletal System	UBERON:0004715	annulus fibrosus disci intervertebralis	CNhs10876	Anulus Pulposus Cell, donor1
UBERON:0002204	Musculoskeletal System	UBERON:0004715	annulus fibrosus disci intervertebralis	CNhs12064	Anulus Pulposus Cell, donor2
UBERON:0002204	Musculoskeletal System	UBERON:0004755	skeletal tissue	CNhs10876	Anulus Pulposus Cell, donor1
UBERON:0002204	Musculoskeletal System	UBERON:0004755	skeletal tissue	CNhs12064	Anulus Pulposus Cell, donor2
UBERON:0002204	Musculoskeletal System	UBERON:0004765	skeletal element	CNhs12845	bone marrow, adult
UBERON:0002204	Musculoskeletal System	UBERON:0004765	skeletal element	CNhs10846	Mesenchymal Stem Cells - Vertebral, donor1
UBERON:0002204	Musculoskeletal System	UBERON:0004765	skeletal element	CNhs10876	Anulus Pulposus Cell, donor1
UBERON:0002204	Musculoskeletal System	UBERON:0004765	skeletal element	CNhs10881	Nucleus Pulposus Cell, donor1
UBERON:0002204	Musculoskeletal System	UBERON:0004765	skeletal element	CNhs12019	Nucleus Pulposus Cell, donor2
UBERON:0002204	Musculoskeletal System	UBERON:0004765	skeletal element	CNhs12063	Nucleus Pulposus Cell, donor3
UBERON:0002204	Musculoskeletal System	UBERON:0004765	skeletal element	CNhs12064	Anulus Pulposus Cell, donor2
UBERON:0002204	Musculoskeletal System	UBERON:0004765	skeletal element	CNhs11316	Mesenchymal Stem Cells - bone marrow, donor4
UBERON:0002204	Musculoskeletal System	UBERON:0004765	skeletal element	CNhs11344	Mesenchymal Stem Cells - bone marrow, donor1
UBERON:0002204	Musculoskeletal System	UBERON:0004765	skeletal element	CNhs12100	Mesenchymal Stem Cells - bone marrow, donor2
UBERON:0002204	Musculoskeletal System	UBERON:0004765	skeletal element	CNhs12126	Mesenchymal Stem Cells - bone marrow, donor3
UBERON:0002204	Musculoskeletal System	UBERON:0004765	skeletal element	CNhs12366	mesenchymal precursor cell - bone marrow, donor1
UBERON:0002204	Musculoskeletal System	UBERON:0004765	skeletal element	CNhs12367	mesenchymal precursor cell - bone marrow, donor2
UBERON:0002204	Musculoskeletal System	UBERON:0004765	skeletal element	CNhs13098	mesenchymal precursor cell - bone marrow, donor3
UBERON:0002204	Musculoskeletal System	UBERON:0004765	skeletal element	CNhs13550	Mallassez-derived cells, donor2
UBERON:0002204	Musculoskeletal System	UBERON:0004765	skeletal element	CNhs13551	Mallassez-derived cells, donor3
UBERON:0002204	Musculoskeletal System	UBERON:0004770	articular system	CNhs13439	cruciate ligament, donor2
UBERON:0002204	Musculoskeletal System	UBERON:0004770	articular system	CNhs10876	Anulus Pulposus Cell, donor1
UBERON:0002204	Musculoskeletal System	UBERON:0004770	articular system	CNhs10881	Nucleus Pulposus Cell, donor1
UBERON:0002204	Musculoskeletal System	UBERON:0004770	articular system	CNhs11068	Synoviocyte, donor1
UBERON:0002204	Musculoskeletal System	UBERON:0004770	articular system	CNhs11992	Synoviocyte, donor2
UBERON:0002204	Musculoskeletal System	UBERON:0004770	articular system	CNhs12019	Nucleus Pulposus Cell, donor2
UBERON:0002204	Musculoskeletal System	UBERON:0004770	articular system	CNhs12050	Synoviocyte, donor3
UBERON:0002204	Musculoskeletal System	UBERON:0004770	articular system	CNhs12063	Nucleus Pulposus Cell, donor3
UBERON:0002204	Musculoskeletal System	UBERON:0004770	articular system	CNhs12064	Anulus Pulposus Cell, donor2
UBERON:0002204	Musculoskeletal System	UBERON:0004830	respiratory system skeletal muscle	CNhs11779	diaphragm, fetal, donor1
UBERON:0002204	Musculoskeletal System	UBERON:0006322	inferior rectus extraocular muscle	CNhs13444	eye - muscle inferior rectus, donor1
UBERON:0002204	Musculoskeletal System	UBERON:0006444	annulus fibrosus	CNhs10876	Anulus Pulposus Cell, donor1
UBERON:0002204	Musculoskeletal System	UBERON:0006444	annulus fibrosus	CNhs12064	Anulus Pulposus Cell, donor2
UBERON:0002204	Musculoskeletal System	UBERON:0006531	oculomotor muscle	CNhs13444	eye - muscle inferior rectus, donor1
UBERON:0002204	Musculoskeletal System	UBERON:0006533	rectus extraocular muscle	CNhs13444	eye - muscle inferior rectus, donor1
UBERON:0002204	Musculoskeletal System	UBERON:0006659	cruciate ligament of knee	CNhs13439	cruciate ligament, donor2
UBERON:0002204	Musculoskeletal System	UBERON:0007844	cartilaginous element	CNhs10876	Anulus Pulposus Cell, donor1
UBERON:0002204	Musculoskeletal System	UBERON:0007844	cartilaginous element	CNhs10881	Nucleus Pulposus Cell, donor1
UBERON:0002204	Musculoskeletal System	UBERON:0007844	cartilaginous element	CNhs12019	Nucleus Pulposus Cell, donor2
UBERON:0002204	Musculoskeletal System	UBERON:0007844	cartilaginous element	CNhs12063	Nucleus Pulposus Cell, donor3
UBERON:0002204	Musculoskeletal System	UBERON:0007844	cartilaginous element	CNhs12064	Anulus Pulposus Cell, donor2
UBERON:0002204	Musculoskeletal System	UBERON:0008001	irregular bone	CNhs10846	Mesenchymal Stem Cells - Vertebral, donor1
UBERON:0002204	Musculoskeletal System	UBERON:0008229	craniocervical region musculature	CNhs13444	eye - muscle inferior rectus, donor1
UBERON:0002204	Musculoskeletal System	UBERON:0008229	craniocervical region musculature	CNhs13441	eye - muscle superior, donor2
UBERON:0002204	Musculoskeletal System	UBERON:0008229	craniocervical region musculature	CNhs13442	eye - muscle lateral, donor2
UBERON:0002204	Musculoskeletal System	UBERON:0008229	craniocervical region musculature	CNhs13443	eye - muscle medial, donor2
UBERON:0002204	Musculoskeletal System	UBERON:0008846	skeletal ligament	CNhs13435	achilles tendon, donor2
UBERON:0002204	Musculoskeletal System	UBERON:0008846	skeletal ligament	CNhs13439	cruciate ligament, donor2
UBERON:0002204	Musculoskeletal System	UBERON:0008895	splanchnocranium	CNhs13550	Mallassez-derived cells, donor2
UBERON:0002204	Musculoskeletal System	UBERON:0008895	splanchnocranium	CNhs13551	Mallassez-derived cells, donor3
UBERON:0002204	Musculoskeletal System	UBERON:0010323	cranial skeletal system	CNhs12611	olfactory region, adult
UBERON:0002204	Musculoskeletal System	UBERON:0010323	cranial skeletal system	CNhs12493	Fibroblast - Periodontal Ligament, donor4 (PL29)
UBERON:0002204	Musculoskeletal System	UBERON:0010323	cranial skeletal system	CNhs10867	Fibroblast - Periodontal Ligament, donor1
UBERON:0002204	Musculoskeletal System	UBERON:0010323	cranial skeletal system	CNhs11953	Fibroblast - Periodontal Ligament, donor5 (PL30)
UBERON:0002204	Musculoskeletal System	UBERON:0010323	cranial skeletal system	CNhs11962	Fibroblast - Periodontal Ligament, donor2
UBERON:0002204	Musculoskeletal System	UBERON:0010323	cranial skeletal system	CNhs11996	Fibroblast - Periodontal Ligament, donor6 (PLH3)
UBERON:0002204	Musculoskeletal System	UBERON:0010323	cranial skeletal system	CNhs11907	Fibroblast - Periodontal Ligament, donor3
UBERON:0002204	Musculoskeletal System	UBERON:0010323	cranial skeletal system	CNhs13550	Mallassez-derived cells, donor2
UBERON:0002204	Musculoskeletal System	UBERON:0010323	cranial skeletal system	CNhs13551	Mallassez-derived cells, donor3
UBERON:0002204	Musculoskeletal System	UBERON:0010363	endochondral element	CNhs10846	Mesenchymal Stem Cells - Vertebral, donor1
UBERON:0002204	Musculoskeletal System	UBERON:0010890	pelvic complex muscle	CNhs13454	skeletal muscle - soleus muscle, donor1
UBERON:0002204	Musculoskeletal System	UBERON:0010890	pelvic complex muscle	CNhs13435	achilles tendon, donor2
UBERON:0002204	Musculoskeletal System	UBERON:0010913	vertebral element	CNhs10846	Mesenchymal Stem Cells - Vertebral, donor1
UBERON:0002204	Musculoskeletal System	UBERON:0010959	craniocervical muscle	CNhs13444	eye - muscle inferior rectus, donor1
UBERON:0002204	Musculoskeletal System	UBERON:0010959	craniocervical muscle	CNhs13441	eye - muscle superior, donor2
UBERON:0002204	Musculoskeletal System	UBERON:0010959	craniocervical muscle	CNhs13442	eye - muscle lateral, donor2
UBERON:0002204	Musculoskeletal System	UBERON:0010959	craniocervical muscle	CNhs13443	eye - muscle medial, donor2
UBERON:0002204	Musculoskeletal System	UBERON:0011088	ligament of knee joint	CNhs13439	cruciate ligament, donor2
UBERON:0002204	Musculoskeletal System	UBERON:0011134	nonsynovial joint	CNhs10876	Anulus Pulposus Cell, donor1
UBERON:0002204	Musculoskeletal System	UBERON:0011134	nonsynovial joint	CNhs10881	Nucleus Pulposus Cell, donor1
UBERON:0002204	Musculoskeletal System	UBERON:0011134	nonsynovial joint	CNhs12019	Nucleus Pulposus Cell, donor2
UBERON:0002204	Musculoskeletal System	UBERON:0011134	nonsynovial joint	CNhs12063	Nucleus Pulposus Cell, donor3
UBERON:0002204	Musculoskeletal System	UBERON:0011134	nonsynovial joint	CNhs12064	Anulus Pulposus Cell, donor2
UBERON:0002204	Musculoskeletal System	UBERON:0011135	intervertebral cartilage	CNhs10876	Anulus Pulposus Cell, donor1
UBERON:0002204	Musculoskeletal System	UBERON:0011135	intervertebral cartilage	CNhs10881	Nucleus Pulposus Cell, donor1

UBERON:0002204	Musculoskeletal System	UBERON:0011135	intervertebral cartilage	CNhs12019	Nucleus Pulposus Cell, donor2
UBERON:0002204	Musculoskeletal System	UBERON:0011135	intervertebral cartilage	CNhs12063	Nucleus Pulposus Cell, donor3
UBERON:0002204	Musculoskeletal System	UBERON:0011135	intervertebral cartilage	CNhs12064	Anulus Pulposus Cell, donor2
UBERON:0002204	Musculoskeletal System	UBERON:0011137	axial skeletal system	CNhs12611	olfactory region, adult
UBERON:0002204	Musculoskeletal System	UBERON:0011137	axial skeletal system	CNhs12493	Fibroblast - Periodontal Ligament, donor4 (PL29)
UBERON:0002204	Musculoskeletal System	UBERON:0011137	axial skeletal system	CNhs10867	Fibroblast - Periodontal Ligament, donor1
UBERON:0002204	Musculoskeletal System	UBERON:0011137	axial skeletal system	CNhs11953	Fibroblast - Periodontal Ligament, donor5 (PL30)
UBERON:0002204	Musculoskeletal System	UBERON:0011137	axial skeletal system	CNhs11962	Fibroblast - Periodontal Ligament, donor2
UBERON:0002204	Musculoskeletal System	UBERON:0011137	axial skeletal system	CNhs11996	Fibroblast - Periodontal Ligament, donor6 (PLH3)
UBERON:0002204	Musculoskeletal System	UBERON:0011137	axial skeletal system	CNhs11907	Fibroblast - Periodontal Ligament, donor3
UBERON:0002204	Musculoskeletal System	UBERON:0011137	axial skeletal system	CNhs13550	Mallassez-derived cells, donor2
UBERON:0002204	Musculoskeletal System	UBERON:0011137	axial skeletal system	CNhs13551	Mallassez-derived cells, donor3
UBERON:0002204	Musculoskeletal System	UBERON:0011138	post-cranial axial skeletal system	CNhs10846	Mesenchymal Stem Cells - Vertebral, donor1
UBERON:0002204	Musculoskeletal System	UBERON:0011138	post-cranial axial skeletal system	CNhs10876	Anulus Pulposus Cell, donor1
UBERON:0002204	Musculoskeletal System	UBERON:0011138	post-cranial axial skeletal system	CNhs10881	Nucleus Pulposus Cell, donor1
UBERON:0002204	Musculoskeletal System	UBERON:0011138	post-cranial axial skeletal system	CNhs12019	Nucleus Pulposus Cell, donor2
UBERON:0002204	Musculoskeletal System	UBERON:0011138	post-cranial axial skeletal system	CNhs12063	Nucleus Pulposus Cell, donor3
UBERON:0002204	Musculoskeletal System	UBERON:0011138	post-cranial axial skeletal system	CNhs12064	Anulus Pulposus Cell, donor2
UBERON:0002204	Musculoskeletal System	UBERON:0011139	synovial limb joint	CNhs13439	cruciate ligament, donor2
UBERON:0002204	Musculoskeletal System	UBERON:0011158	primary subdivision of skull	CNhs12611	olfactory region, adult
UBERON:0002204	Musculoskeletal System	UBERON:0011159	primary subdivision of cranial skeletal system	CNhs12611	olfactory region, adult
UBERON:0002204	Musculoskeletal System	UBERON:0011159	primary subdivision of cranial skeletal system	CNhs13550	Mallassez-derived cells, donor2
UBERON:0002204	Musculoskeletal System	UBERON:0011159	primary subdivision of cranial skeletal system	CNhs13551	Mallassez-derived cells, donor3
UBERON:0002405	Immune System	UBERON:0000029	lymph node	CNhs11788	lymph node, adult, donor1
UBERON:0002405	Immune System	UBERON:0001473	lymphatic vessel	CNhs10865	Endothelial Cells - Lymphatic, donor1
UBERON:0002405	Immune System	UBERON:0001473	lymphatic vessel	CNhs11901	Endothelial Cells - Lymphatic, donor2
UBERON:0002405	Immune System	UBERON:0001473	lymphatic vessel	CNhs11906	Endothelial Cells - Lymphatic, donor3
UBERON:0002405	Immune System	UBERON:0001473	lymphatic vessel	CNhs11322	Fibroblast - Lymphatic, donor1
UBERON:0002405	Immune System	UBERON:0001473	lymphatic vessel	CNhs12082	Fibroblast - Lymphatic, donor2
UBERON:0002405	Immune System	UBERON:0001473	lymphatic vessel	CNhs12118	Fibroblast - Lymphatic, donor3
UBERON:0002405	Immune System	UBERON:0001735	tonsillar ring	CNhs10654	tonsil, adult, pool1
UBERON:0002405	Immune System	UBERON:0001744	lymphoid tissue	CNhs10654	tonsil, adult, pool1
UBERON:0002405	Immune System	UBERON:0001961	mucosa-associated lymphoid tissue	CNhs10654	tonsil, adult, pool1
UBERON:0002405	Immune System	UBERON:0002106	spleen	CNhs10631	spleen, adult, pool1
UBERON:0002405	Immune System	UBERON:0002106	spleen	CNhs10651	spleen, fetal, pool1
UBERON:0002405	Immune System	UBERON:0002370	thymus	CNhs10633	thymus, adult, pool1
UBERON:0002405	Immune System	UBERON:0002370	thymus	CNhs10650	thymus, fetal, pool1
UBERON:0002405	Immune System	UBERON:0002371	bone marrow	CNhs12845	bone marrow, adult
UBERON:0002405	Immune System	UBERON:0002371	bone marrow	CNhs11316	Mesenchymal Stem Cells - bone marrow, donor4
UBERON:0002405	Immune System	UBERON:0002371	bone marrow	CNhs11344	Mesenchymal Stem Cells - bone marrow, donor1
UBERON:0002405	Immune System	UBERON:0002371	bone marrow	CNhs12100	Mesenchymal Stem Cells - bone marrow, donor2
UBERON:0002405	Immune System	UBERON:0002371	bone marrow	CNhs12126	Mesenchymal Stem Cells - bone marrow, donor3
UBERON:0002405	Immune System	UBERON:0002371	bone marrow	CNhs12366	mesenchymal precursor cell - bone marrow, donor1
UBERON:0002405	Immune System	UBERON:0002371	bone marrow	CNhs12367	mesenchymal precursor cell - bone marrow, donor2
UBERON:0002405	Immune System	UBERON:0002371	bone marrow	CNhs13098	mesenchymal precursor cell - bone marrow, donor3
UBERON:0002405	Immune System	UBERON:0002372	tonsil	CNhs10654	tonsil, adult, pool1
UBERON:0002405	Immune System	UBERON:0002405	immune system	CNhs10631	spleen, adult, pool1
UBERON:0002405	Immune System	UBERON:0002405	immune system	CNhs10633	thymus, adult, pool1
UBERON:0002405	Immune System	UBERON:0002405	immune system	CNhs10650	thymus, fetal, pool1
UBERON:0002405	Immune System	UBERON:0002405	immune system	CNhs10651	spleen, fetal, pool1
UBERON:0002405	Immune System	UBERON:0002405	immune system	CNhs10654	tonsil, adult, pool1
UBERON:0002405	Immune System	UBERON:0002405	immune system	CNhs11788	lymph node, adult, donor1
UBERON:0002405	Immune System	UBERON:0002405	immune system	CNhs12845	bone marrow, adult
UBERON:0002405	Immune System	UBERON:0002405	immune system	CNhs10865	Endothelial Cells - Lymphatic, donor1
UBERON:0002405	Immune System	UBERON:0002405	immune system	CNhs11901	Endothelial Cells - Lymphatic, donor2
UBERON:0002405	Immune System	UBERON:0002405	immune system	CNhs11906	Endothelial Cells - Lymphatic, donor3
UBERON:0002405	Immune System	UBERON:0002405	immune system	CNhs11316	Mesenchymal Stem Cells - bone marrow, donor4
UBERON:0002405	Immune System	UBERON:0002405	immune system	CNhs11322	Fibroblast - Lymphatic, donor1
UBERON:0002405	Immune System	UBERON:0002405	immune system	CNhs11344	Mesenchymal Stem Cells - bone marrow, donor1
UBERON:0002405	Immune System	UBERON:0002405	immune system	CNhs12082	Fibroblast - Lymphatic, donor2
UBERON:0002405	Immune System	UBERON:0002405	immune system	CNhs12100	Mesenchymal Stem Cells - bone marrow, donor2
UBERON:0002405	Immune System	UBERON:0002405	immune system	CNhs12118	Fibroblast - Lymphatic, donor3
UBERON:0002405	Immune System	UBERON:0002405	immune system	CNhs12126	Mesenchymal Stem Cells - bone marrow, donor3
UBERON:0002405	Immune System	UBERON:0002405	immune system	CNhs12366	mesenchymal precursor cell - bone marrow, donor1
UBERON:0002405	Immune System	UBERON:0002405	immune system	CNhs12367	mesenchymal precursor cell - bone marrow, donor2
UBERON:0002405	Immune System	UBERON:0002405	immune system	CNhs13098	mesenchymal precursor cell - bone marrow, donor3
UBERON:0002405	Immune System	UBERON:0002465	lymphoid system	CNhs10654	tonsil, adult, pool1
UBERON:0002405	Immune System	UBERON:0002465	lymphoid system	CNhs11788	lymph node, adult, donor1
UBERON:0002405	Immune System	UBERON:0002465	lymphoid system	CNhs10865	Endothelial Cells - Lymphatic, donor1
UBERON:0002405	Immune System	UBERON:0002465	lymphoid system	CNhs11901	Endothelial Cells - Lymphatic, donor2
UBERON:0002405	Immune System	UBERON:0002465	lymphoid system	CNhs11906	Endothelial Cells - Lymphatic, donor3
UBERON:0002405	Immune System	UBERON:0002465	lymphoid system	CNhs11322	Fibroblast - Lymphatic, donor1
UBERON:0002405	Immune System	UBERON:0002465	lymphoid system	CNhs12082	Fibroblast - Lymphatic, donor2
UBERON:0002405	Immune System	UBERON:0002465	lymphoid system	CNhs12118	Fibroblast - Lymphatic, donor3
UBERON:0002405	Immune System	UBERON:0004536	lymph vasculature	CNhs10865	Endothelial Cells - Lymphatic, donor1
UBERON:0002405	Immune System	UBERON:0004536	lymph vasculature	CNhs11901	Endothelial Cells - Lymphatic, donor2
UBERON:0002405	Immune System	UBERON:0004536	lymph vasculature	CNhs11906	Endothelial Cells - Lymphatic, donor3
UBERON:0002405	Immune System	UBERON:0004536	lymph vasculature	CNhs11322	Fibroblast - Lymphatic, donor1
UBERON:0002405	Immune System	UBERON:0004536	lymph vasculature	CNhs12082	Fibroblast - Lymphatic, donor2
UBERON:0002405	Immune System	UBERON:0004536	lymph vasculature	CNhs12118	Fibroblast - Lymphatic, donor3
UBERON:0002405	Immune System	UBERON:0005057	immune organ	CNhs10631	spleen, adult, pool1
UBERON:0002405	Immune System	UBERON:0005057	immune organ	CNhs10633	thymus, adult, pool1
UBERON:0002405	Immune System	UBERON:0005057	immune organ	CNhs10650	thymus, fetal, pool1
UBERON:0002405	Immune System	UBERON:0005057	immune organ	CNhs10651	spleen, fetal, pool1
UBERON:0002405	Immune System	UBERON:0006558	lymphatic part of lymphoid system	CNhs10865	Endothelial Cells - Lymphatic, donor1
UBERON:0002405	Immune System	UBERON:0006558	lymphatic part of lymphoid system	CNhs11901	Endothelial Cells - Lymphatic, donor2
UBERON:0002405	Immune System	UBERON:0006558	lymphatic part of lymphoid system	CNhs11906	Endothelial Cells - Lymphatic, donor3
UBERON:0002405	Immune System	UBERON:0006558	lymphatic part of lymphoid system	CNhs11322	Fibroblast - Lymphatic, donor1
UBERON:0002405	Immune System	UBERON:0006558	lymphatic part of lymphoid system	CNhs12082	Fibroblast - Lymphatic, donor2
UBERON:0002405	Immune System	UBERON:0006558	lymphatic part of lymphoid system	CNhs12118	Fibroblast - Lymphatic, donor3
