# Supplementary material for: EvoTol: a protein-sequence based evolutionary intolerance framework for disease-gene prioritization
Source: Nucleic Acids Res. 2014 Dec 29;43(5):e33. doi: 10.1093/nar/gku1322 (PMC4357693; doi:10.1093/nar/gku1322)
Supplement: SUPPLEMENTARY DATA [file Supplemental Table 3.pdf › Supp/Supplemental Table 3.pdf]

Supplemental Table 3. Proteins (and corresponding genes) associated with epilepsy, retrived from UniProt (keyword: "Epilepsy [KW-0887]". The UniProt keyword "KW-0887" refers to the following classification: "protein which, if defective, causes epilepsy, any of a group of disorders characterized by paroxysmal transient disturbances of the electrical activity of the brain that may be manifested as episodic impairment or loss of consciousness, abnormal motor phenomena, psychic or sensory disturbances, or perturbation of the autonomic nervous system. Epilepsy is classified as either symptomatic or idiopathic according to whether the cause is known or unknown. Both of these types can be classified into partial and generalized epilepsy, depending on whether the seizures are due to limited or to widespread brain lesions, respectively."

Entry	Entry name	Status	Protein names	Associated gene names	Organism	Length
Q15822	ACHA2_HUMAN	reviewed	Neuronal acetylcholine receptor subunit alpha-2	*CHRNA2*	Homo sapiens (Human)	529
P43681	ACHA4_HUMAN	reviewed	Neuronal acetylcholine receptor subunit alpha-4	*CHRNA4 NACRA4*	Homo sapiens (Human)	627
P17787	ACHB2_HUMAN	reviewed	Neuronal acetylcholine receptor subunit beta-2	*CHRNB2*	Homo sapiens (Human)	502
P49419	AL7A1_HUMAN	reviewed	Alpha-aminoadipic semialdehyde dehydrogenase (Alpha-AASA dehydrogenase) (EC 1.2.1.31) (Aldehyde dehydrogenase family 7 member A1) (EC 1.2.1.3) (Antiquitin-1) (Betaine aldehyde dehydrogenase) (EC 1.2.1.8) (Delta1-piperideine-6-carboxylate dehydrogenase) (P6c dehydrogenas	*ALDH7A1 ATQ1*	Homo sapiens (Human)	539
O43307	ARHG9_HUMAN	reviewed	Rho guanine nucleotide exchange factor 9 (Collybistin) (PEM-2 homolog) (Rac/Cdc42 guanine nucleotide exchange factor 9)	*ARHGEF9 ARHDH9 KIAA0424*	Homo sapiens (Human)	516
Q96QS3	ARX_HUMAN	reviewed	Homeobox protein ARX (Aristaless-related homeobox)	*ARX*	Homo sapiens (Human)	562
Q13510	ASAH1_HUMAN	reviewed	Acid ceramidase (AC) (ACDase) (Acid CDase) (EC 3.5.1.23) (Acylsphingosine deacylase) (N-acylsphingosine amidohydrolase) (Putative 32 kDa heart protein) (PHP32) [Cleaved into: Acid ceramidase subunit alpha; Acid ceramidase subunit beta]	*ASAH1 ASAH HSD-33 HSD33*	Homo sapiens (Human)	395
P16615	AT2A2_HUMAN	reviewed	Sarcoplasmic/endoplasmic reticulum calcium ATPase 2 (SERCA2) (SR Ca(2+)-ATPase 2) (EC 3.6.3.8) (Calcium pump 2) (Calcium-transporting ATPase sarcoplasmic reticulum type, slow twitch skeletal muscle isoform) (Endoplasmic reticulum class 1/2 Ca(2+) ATPase)	*ATP2A2 ATP2B*	Homo sapiens (Human)	1042
P54259	ATN1_HUMAN	reviewed	Atrophin-1 (Dentatorubral-pallidoluysian atrophy protein)	*ATN1 D12S755E DRPLA*	Homo sapiens (Human)	1190
Q6PJG6	BRAT1_HUMAN	reviewed	BRCA1-associated ATM activator 1 (BRCA1-associated protein required for ATM activation protein 1)	*BRAT1 BAAT1 C7orf27*	Homo sapiens (Human)	821
O95180	CAC1H_HUMAN	reviewed	Voltage-dependent T-type calcium channel subunit alpha-1H (Low-voltage-activated calcium channel alpha1 3.2 subunit) (Voltage-gated calcium channel subunit alpha Cav3.2)	*CACNA1H*	Homo sapiens (Human)	2353
O00305	CACB4_HUMAN	reviewed	Voltage-dependent L-type calcium channel subunit beta-4 (CAB4) (Calcium channel voltage-dependent subunit beta 4)	*CACNB4 CACNLB4*	Homo sapiens (Human)	520
P41180	CASR_HUMAN	reviewed	Extracellular calcium-sensing receptor (CaSR) (Parathyroid cell calcium-sensing receptor 1) (PCaR1)	*CASR GPRC2A PCAR1*	Homo sapiens (Human)	1078
Q8N4T0	CBPA6_HUMAN	reviewed	Carboxypeptidase A6 (EC 3.4.17.1) (Carboxypeptidase B)	*CPA6 CPAH*	Homo sapiens (Human)	437
O76039	CDKL5_HUMAN	reviewed	Cyclin-dependent kinase-like 5 (EC 2.7.11.22) (Serine/threonine-protein kinase 9)	*CDKL5 STK9*	Homo sapiens (Human)	1030
O14647	CHD2_HUMAN	reviewed	Chromodomain-helicase-DNA-binding protein 2 (CHD-2) (EC 3.6.4.12) (ATP-dependent helicase CHD2)	*CHD2*	Homo sapiens (Human)	1828
P51788	CLCN2_HUMAN	reviewed	Chloride channel protein 2 (ClC-2)	*CLCN2*	Homo sapiens (Human)	898
O75503	CLN5_HUMAN	reviewed	Ceroid-lipofuscinosis neuronal protein 5 (Protein CLN5)	*CLN5*	Homo sapiens (Human)	358
Q9UBY8	CLN8_HUMAN	reviewed	Protein CLN8	*CLN8 C8orf61*	Homo sapiens (Human)	286
Q9UHC6	CNTP2_HUMAN	reviewed	Contactin-associated protein-like 2 (Cell recognition molecule Caspr2)	*CNTNAP2 CASPR2 KIAA0868*	Homo sapiens (Human)	1331
Q02246	CNTN2_HUMAN	reviewed	Contactin-2 (Axonal glycoprotein TAG-1) (Axonin-1) (Transient axonal glycoprotein 1) (TAX-1)	*CNTN2 AXT TAG1 TAX1*	Homo sapiens (Human)	1040
P04080	CYTB_HUMAN	reviewed	Cystatin-B (CPI-B) (Liver thiol proteinase inhibitor) (Stefin-B)	*CSTB CST6 STFB*	Homo sapiens (Human)	98
O43602	DCX_HUMAN	reviewed	Neuronal migration protein doublecortin (Doublin) (Lissencephalin-X) (Lis-X)	*DCX DBCN LISX*	Homo sapiens (Human)	441
O75140	DEPD5_HUMAN	reviewed	DEP domain-containing protein 5	*DEPDC5 KIAA0645*	Homo sapiens (Human)	1603
Q5JVL4	EFHC1_HUMAN	reviewed	EF-hand domain-containing protein 1 (Myoclonin-1)	*EFHC1*	Homo sapiens (Human)	640
O95278	EPM2A_HUMAN	reviewed	Laforin (EC 3.1.3.16) (EC 3.1.3.48) (Lafora PTPase) (LAFPTPase)	*EPM2A*	Homo sapiens (Human)	331
P14867	GBRA1_HUMAN	reviewed	Gamma-aminobutyric acid receptor subunit alpha-1 (GABA(A) receptor subunit alpha-1)	*GABRA1*	Homo sapiens (Human)	456
P28472	GBRB3_HUMAN	reviewed	Gamma-aminobutyric acid receptor subunit beta-3 (GABA(A) receptor subunit beta-3)	*GABRB3*	Homo sapiens (Human)	473
O14764	GBRD_HUMAN	reviewed	Gamma-aminobutyric acid receptor subunit delta (GABA(A) receptor subunit delta)	*GABRD*	Homo sapiens (Human)	452
P18507	GBRG2_HUMAN	reviewed	Gamma-aminobutyric acid receptor subunit gamma-2 (GABA(A) receptor subunit gamma-2)	*GABRG2*	Homo sapiens (Human)	467
Q9H936	GHC1_HUMAN	reviewed	Mitochondrial glutamate carrier 1 (GC-1) (Glutamate/H(+) symporter 1) (Solute carrier family 25 member 22)	*SLC25A22 GC1*	Homo sapiens (Human)	323
O14653	GOSR2_HUMAN	reviewed	Golgi SNAP receptor complex member 2 (27 kDa Golgi SNARE protein) (Membrin)	*GOSR2 GS27*	Homo sapiens (Human)	212
P11166	GTR1_HUMAN	reviewed	Solute carrier family 2, facilitated glucose transporter member 1 (Glucose transporter type 1, erythrocyte/brain) (GLUT-1) (HepG2 glucose transporter)	*SLC2A1 GLUT1*	Homo sapiens (Human)	492
Q9Y5U9	IR3IP_HUMAN	reviewed	Immediate early response 3-interacting protein 1	*IER3IP1 HSPC039*	Homo sapiens (Human)	82
P78508	IRK10_HUMAN	reviewed	ATP-sensitive inward rectifier potassium channel 10 (ATP-dependent inwardly rectifying potassium channel Kir4.1) (Inward rectifier K(+) channel Kir1.2) (Potassium channel, inwardly rectifying subfamily J member 10)	*KCNJ10*	Homo sapiens (Human)	379
P15884	ITF2_HUMAN	reviewed	Transcription factor 4 (TCF-4) (Class B basic helix-loop-helix protein 19) (bHLHb19) (Immunoglobulin transcription factor 2) (ITF-2) (SL3-3 enhancer factor 2) (SEF-2)	*TCF4 BHLHB19 ITF2 SEF2*	Homo sapiens (Human)	667
Q12791	KCMA1_HUMAN	reviewed	Calcium-activated potassium channel subunit alpha-1 (BK channel) (BKCA alpha) (Calcium-activated potassium channel, subfamily M subunit alpha-1) (K(VCA)alpha) (KCa1.1) (Maxi K channel) (MaxiK) (Slo-alpha) (Slo1) (Slowpoke homolog) (Slo homolog) (hSlo)	*KCNMA1 KCNMA SLO*	Homo sapiens (Human)	1236
O43526	KCNQ2_HUMAN	reviewed	Potassium voltage-gated channel subfamily KQT member 2 (KQT-like 2) (Neuroblastoma-specific potassium channel subunit alpha KvLQT2) (Voltage-gated potassium channel subunit Kv7.2)	*KCNQ2*	Homo sapiens (Human)	872
O43525	KCNQ3_HUMAN	reviewed	Potassium voltage-gated channel subfamily KQT member 3 (KQT-like 3) (Potassium channel subunit alpha KvLQT3) (Voltage-gated potassium channel subunit Kv7.3)	*KCNQ3*	Homo sapiens (Human)	872
Q5JUK3	KCNT1_HUMAN	reviewed	Potassium channel subfamily T member 1 (KCa4.1)	*KCNT1 KIAA1422*	Homo sapiens (Human)	1230
Q96MP8	KCTD7_HUMAN	reviewed	BTB/POZ domain-containing protein KCTD7	*KCTD7*	Homo sapiens (Human)	289
O95970	LGI1_HUMAN	reviewed	Leucine-rich glioma-inactivated protein 1 (Epitempin-1)	*LGI1 EPT UNQ775/PRO1569*	Homo sapiens (Human)	557
Q06413	MEF2C_HUMAN	reviewed	Myocyte-specific enhancer factor 2C	*MEF2C*	Homo sapiens (Human)	473
P53779	MK10_HUMAN	reviewed	Mitogen-activated protein kinase 10 (MAP kinase 10) (MAPK 10) (EC 2.7.11.24) (MAP kinase p49 3F12) (Stress-activated protein kinase 1b) (SAPK1b) (Stress-activated protein kinase JNK3) (c-Jun N-terminal kinase 3)	*MAPK10 JNK3 JNK3A PRKM10 SAPK1B*	Homo sapiens (Human)	464
P17050	NAGAB_HUMAN	reviewed	Alpha-N-acetylgalactosaminidase (EC 3.2.1.49) (Alpha-galactosidase B)	*NAGA*	Homo sapiens (Human)	411
Q6VVB1	NHLC1_HUMAN	reviewed	E3 ubiquitin-protein ligase NHLRC1 (EC 6.3.2.-) (Malin) (NHL repeat-containing protein 1)	*NHLRC1 EPM2B*	Homo sapiens (Human)	395
Q12879	NMDE1_HUMAN	reviewed	Glutamate receptor ionotropic, NMDA 2A (GluN2A) (Glutamate [NMDA] receptor subunit epsilon-1) (N-methyl D-aspartate receptor subtype 2A) (NMDAR2A) (NR2A) (hNR2A)	*GRIN2A NMDAR2A*	Homo sapiens (Human)	1464
Q8TAB3	PCD19_HUMAN	reviewed	Protocadherin-19	*PCDH19 KIAA1313*	Homo sapiens (Human)	1148
Q8IWS0	PHF6_HUMAN	reviewed	PHD finger protein 6 (PHD-like zinc finger protein)	*PHF6 KIAA1823*	Homo sapiens (Human)	365
P37287	PIGA_HUMAN	reviewed	Phosphatidylinositol N-acetylglucosaminyltransferase subunit A (EC 2.4.1.198) (GlcNAc-PI synthesis protein) (Phosphatidylinositol-glycan biosynthesis class A protein) (PIG-A)	*PIGA*	Homo sapiens (Human)	484
O95427	PIGN_HUMAN	reviewed	GPI ethanolamine phosphate transferase 1 (EC 2.-.-.-) (MCD4 homolog) (Phosphatidylinositol-glycan biosynthesis class N protein) (PIG-N)	*PIGN MCD4*	Homo sapiens (Human)	931
Q969N2	PIGT_HUMAN	reviewed	GPI transamidase component PIG-T (Phosphatidylinositol-glycan biosynthesis class T protein)	*PIGT CGI-06 PSEC0163 UNQ716/PRO1379*	Homo sapiens (Human)	578
Q9NQ66	PLCB1_HUMAN	reviewed	1-phosphatidylinositol 4,5-bisphosphate phosphodiesterase beta-1 (EC 3.1.4.11) (PLC-154) (Phosphoinositide phospholipase C-beta-1) (Phospholipase C-I) (PLC-I) (Phospholipase C-beta-1) (PLC-beta-1)	*PLCB1 KIAA0581*	Homo sapiens (Human)	1216
Q9NVS9	PNPO_HUMAN	reviewed	Pyridoxine-5'-phosphate oxidase (EC 1.4.3.5) (Pyridoxamine-phosphate oxidase)	*PNPO*	Homo sapiens (Human)	261
Q7Z3G6	PRIC2_HUMAN	reviewed	Prickle-like protein 2	*PRICKLE2*	Homo sapiens (Human)	844
Q96MT3	PRIC1_HUMAN	reviewed	Prickle-like protein 1 (REST/NRSF-interacting LIM domain protein 1)	*PRICKLE1 RILP*	Homo sapiens (Human)	831
Q7Z6L0	PRRT2_HUMAN	reviewed	Proline-rich transmembrane protein 2 (Dispanin subfamily B member 3) (DSPB3)	*PRRT2*	Homo sapiens (Human)	340
P30566	PUR8_HUMAN	reviewed	Adenylosuccinate lyase (ASL) (EC 4.3.2.2) (Adenylosuccinase) (ASase)	*ADSL AMPS*	Homo sapiens (Human)	484
P31939	PUR9_HUMAN	reviewed	Bifunctional purine biosynthesis protein PURH [Includes: Phosphoribosylaminoimidazolecarboxamide formyltransferase (EC 2.1.2.3) (5-aminoimidazole-4-carboxamide ribonucleotide formyltransferase) (AICAR transformylase); IMP cyclohydrolase (EC 3.5.4.10) (ATIC) (IMP synthase) (Inosi	*ATIC PURH OK/SW-cl.86*	Homo sapiens (Human)	592
O75787	RENR_HUMAN	reviewed	Renin receptor (ATPase H(+)-transporting lysosomal accessory protein 2) (ATPase H(+)-transporting lysosomal-interacting protein 2) (ER-localized type I transmembrane adaptor) (Embryonic liver differentiation factor 10) (N14F) (Renin/prorenin receptor) (Vacuolar ATP synthase membrane	*ATP6AP2 ATP6IP2 CAPER ELDF10 HT028 MSTP009 PSEC0072*	Homo sapiens (Human)	350
Q9GZN7	ROGDI_HUMAN	reviewed	Protein rogdi homolog	*ROGDI*	Homo sapiens (Human)	287
Q99250	SCN2A_HUMAN	reviewed	Sodium channel protein type 2 subunit alpha (HBSC II) (Sodium channel protein brain II subunit alpha) (Sodium channel protein type II subunit alpha) (Voltage-gated sodium channel subunit alpha Nav1.2)	*SCN2A NAC2 SCN2A1 SCN2A2*	Homo sapiens (Human)	2005
P35498	SCN1A_HUMAN	reviewed	Sodium channel protein type 1 subunit alpha (Sodium channel protein brain I subunit alpha) (Sodium channel protein type I subunit alpha) (Voltage-gated sodium channel subunit alpha Nav1.1)	*SCN1A NAC1 SCN1*	Homo sapiens (Human)	2009
Q15858	SCN9A_HUMAN	reviewed	Sodium channel protein type 9 subunit alpha (Neuroendocrine sodium channel) (hNE-Na) (Peripheral sodium channel 1) (PN1) (Sodium channel protein type IX subunit alpha) (Voltage-gated sodium channel subunit alpha Nav1.7)	*SCN9A NENA*	Homo sapiens (Human)	1988
Q07699	SCN1B_HUMAN	reviewed	Sodium channel subunit beta-1	*SCN1B*	Homo sapiens (Human)	218
Q9UQD0	SCN8A_HUMAN	reviewed	Sodium channel protein type 8 subunit alpha (Sodium channel protein type VIII subunit alpha) (Voltage-gated sodium channel subunit alpha Nav1.6)	*SCN8A MED*	Homo sapiens (Human)	1980
Q14108	SCRB2_HUMAN	reviewed	Lysosome membrane protein 2 (85 kDa lysosomal membrane sialoglycoprotein) (LGP85) (CD36 antigen-like 2) (Lysosome membrane protein II) (LIMP II) (Scavenger receptor class B member 2) (CD antigen CD36)	*SCARB2 CD36L2 LIMP2 LIMPII*	Homo sapiens (Human)	478
Q11203	SIAT6_HUMAN	reviewed	CMP-N-acetylneuraminate-beta-1,4-galactoside alpha-2,3-sialyltransferase (EC 2.4.99.6) (Beta-galactoside alpha-2,3-sialyltransferase 3) (Alpha 2,3-ST 3) (Gal beta-1,3(4) GlcNAc alpha-2,3 sialyltransferase) (N-acetyllactosaminide alpha-2,3-sialyltransferase) (ST3Gal III) (ST3GalIII) (ST3N	*ST3GAL3 SIAT6*	Homo sapiens (Human)	375
Q9UNP4	SIAT9_HUMAN	reviewed	Lactosylceramide alpha-2,3-sialyltransferase (EC 2.4.99.9) (CMP-NeuAc:lactosylceramide alpha-2,3-sialyltransferase) (Ganglioside GM3 synthase) (ST3Gal V) (ST3GalV) (Sialyltransferase 9)	*ST3GAL5 SIAT9 UNQ2510/PRO5998*	Homo sapiens (Human)	418
Q92581	SL9A6_HUMAN	reviewed	Sodium/hydrogen exchanger 6 (Na(+)/H(+) exchanger 6) (NHE-6) (Solute carrier family 9 member 6)	*SLC9A6 KIAA0267 NHE6*	Homo sapiens (Human)	669
Q8TAD8	SNIP1_HUMAN	reviewed	Smad nuclear-interacting protein 1 (FHA domain-containing protein SNIP1)	*SNIP1*	Homo sapiens (Human)	396
Q13813	SPTN1_HUMAN	reviewed	Spectrin alpha chain, non-erythrocytic 1 (Alpha-II spectrin) (Fodrin alpha chain) (Spectrin, non-erythroid alpha subunit)	*SPTAN1 NEAS SPTA2*	Homo sapiens (Human)	2472
O60687	SRPX2_HUMAN	reviewed	Sushi repeat-containing protein SRPX2 (Sushi-repeat protein upregulated in leukemia)	*SRPX2 SRPUL*	Homo sapiens (Human)	465
P61764	STXB1_HUMAN	reviewed	Syntaxin-binding protein 1 (MUNC18-1) (N-Sec1) (Protein unc-18 homolog 1) (Unc18-1) (Protein unc-18 homolog A) (Unc-18A) (p67)	*STXBP1 UNC18A*	Homo sapiens (Human)	594
P17600	SYN1_HUMAN	reviewed	Synapsin-1 (Brain protein 4.1) (Synapsin I)	*SYN1*	Homo sapiens (Human)	705
Q5T011	SZT2_HUMAN	reviewed	Protein SZT2 (Seizure threshold 2 protein homolog)	*SZT2 C1orf84 KIAA0467*	Homo sapiens (Human)	3432
Q9ULP9	TBC24_HUMAN	reviewed	TBC1 domain family member 24	*TBC1D24 KIAA1171*	Homo sapiens (Human)	559
O14773	TPP1_HUMAN	reviewed	Tripeptidyl-peptidase 1 (TPP-1) (EC 3.4.14.9) (Cell growth-inhibiting gene 1 protein) (Lysosomal pepstatin-insensitive protease) (LPIC) (Tripeptidyl aminopeptidase) (Tripeptidyl-peptidase I) (TPP-I)	*TPP1 CLN2 GIG1 UNQ267/PRO304*	Homo sapiens (Human)	563
Q92574	TSC1_HUMAN	reviewed	Hamartin (Tuberous sclerosis 1 protein)	*TSC1 KIAA0243 TSC*	Homo sapiens (Human)	1164
Q96RL7	VP13A_HUMAN	reviewed	Vacuolar protein sorting-associated protein 13A (Chorea-acanthocytosis protein) (Chorein)	*VPS13A CHAC KIAA0986*	Homo sapiens (Human)	3174
O60315	ZEB2_HUMAN	reviewed	Zinc finger E-box-binding homeobox 2 (Smad-interacting protein 1) (SMADIP1) (Zinc finger homeobox protein 1b)	*ZEB2 KIAA0569 SIP1 ZFHX1B ZFX1B HRIHFB2411*	Homo sapiens (Human)	1214
